# HELIOS: High-speed sequence alignment in optics

**DOI:** 10.1371/journal.pcbi.1010665

**Published:** 2022-11-21

**Authors:** Ehsan Maleki, Saeedeh Akbari Rokn Abadi, Somayyeh Koohi

**Affiliations:** Department of Computer Engineering, Sharif University of Technology, Tehran, Iran; University of Virginia, UNITED STATES

## Abstract

In response to the imperfections of current sequence alignment methods, originated from the inherent serialism within their corresponding electrical systems, a few optical approaches for biological data comparison have been proposed recently. However, due to their low performance, raised from their inefficient coding scheme, this paper presents a novel all-optical high-throughput method for aligning DNA, RNA, and protein sequences, named HELIOS. The HELIOS method employs highly sophisticated operations to locate character matches, single or multiple mutations, and single or multiple indels within various biological sequences. On the other hand, the HELIOS optical architecture exploits high-speed processing and operational parallelism in optics, by adopting wavelength and polarization of optical beams. For evaluation, the functionality and accuracy of the HELIOS method are approved through behavioral and optical simulation studies, while its complexity and performance are estimated through analytical computation. The accuracy evaluations indicate that the HELIOS method achieves a precise pairwise alignment of two sequences, highly similar to those of Smith-Waterman, Needleman-Wunsch, BLAST, MUSCLE, ClustalW, ClustalΩ, T-Coffee, Kalign, and MAFFT. According to our performance evaluations, the HELIOS optical architecture outperforms all alternative electrical and optical algorithms in terms of processing time and memory requirement, relying on its highly sophisticated method and optical architecture. Moreover, the employed compact coding scheme highly escalates the number of input characters, and hence, it offers reduced time and space complexities, compared to the electrical and optical alternatives. It makes the HELIOS method and optical architecture highly applicable for biomedical applications.

## Author summary

The character-by-character alignment of two long biological sequences, i.e. DNA, RNA, and protein, is a tedious task, but essential for recognizing homologies, relationships, and variations. In this case, every alteration, including mutations (substitution), and indels (insertion or deletion) is vital and required for many biological developments like diagnosis, medicine, and vaccination. However, the applicability of current sequence alignment methods is limited, specifically in processing time and memory usage, due to their inherent serialism and imperfections of electrical systems, as well as inefficient coding schemes of optical approaches. It approximately leads to quadratic run-time and space requirements in terms of input sequence lengths, becoming an expensive and laborious process for the real-time alignment of large datasets. Hence, proposing a superior alignment method in terms of accuracy, performance, and applicability can promote biological research and developments. Here, we show that we can overcome the long-lasting and challenging problems in sequence alignment procedure by exploiting optics as a novel computing technology. In this manner, we propose a novel method and its optical architecture for alignment of DNA, RNA, and protein sequences by exploiting high-speed processing and operational parallelism in optics. As our simulation studies confirm, it provides an accurate sequence alignment with outperforming the most widely used electrical and optical alternatives in the terms of processing time and memory requirements.

This is a *PLOS Computational Biology* Methods paper.

## Introduction

Bioinformatics develops computation-intensive techniques to enhance theoretical and practical biological studies [1]. Pairwise sequence alignment as one of the key operations of bioinformatics compares two DNA, RNA, or protein sequences to recognize homology, similarity, and variation [2]. The character-by-character alignment of two long biological sequences is a tedious task, but essential to locate character matches, mutations (i.e., substitution), and indels (i.e., insertion or deletion) in favor of many biological developments [3].

In this manner, many existing sequence alignment methods consume considerable resources to perform an accurate sequence alignment [4]. For instance, Smith-Waterman [5] and Needleman-Wunsch [6] are based on dynamic programming (DP); BLAST [7], ClustalW [8], ClustalΩ [9] T-Coffee [10], and Kalign [11] utilize a heuristic search; MUSCLE [12] and MAFFT [13] are iterative methods which perform an FFT-based cross-correlation; MUMmer [14] relies on suffix trees; and HMM-based methods [15] are built upon a probabilistic model. Despite their accurate sequence alignment, their resource demands, specifically in time and space, are originated from their sequential natures [16]. Moreover, these methods suffer from various problems due to the imperfections of the electrical systems, such as high computational time and space, high power consumption, heat generation, slow response, etc. [17]. Furthermore, the rapid enlargement of biological datasets and the advancements of bioscience challenge them more than ever [18]. Although various parallel and distributed optimization methods [19] could moderate some of these problems, the electrical implementation of these algorithms enforces inherent serial computation and high memory requirements [20]. Specifically, these methods lead to high time and space requirements in terms of the input sequence lengths [21, 22], which severely limits their applicability. Hence, proposing a superior method in terms of speed, accuracy, and applicability is crucial for the real-time processing of large biological data.

Fortunately, the inherent benefit of optics and photonics [23], as a novel computing technology, provides high-speed operational parallelism and avoids the imperfections of electrical systems [24]. Accordingly, biophotonics develops optical techniques for biological developments [25]. In this manner, some methods have been accomplished recently, such as correlation-based methods [26, 27], Fourier-Transform-based algorithms [28, 29], HAWPOD [30], Moiré Technique [31–33], OptCAM [34], GAC [35], and SPOMF [36]. Some of them [26–29] address optical similarity measurement algorithms for sequence alignment by taking advantage of optical correlation and Fourier Transform. Despite their high-speed processing, these methods only measure the similarities and differences between the input sequences within specific zones, regardless of the exact location of the variations and their importance to biological developments [1]. On the other hand, some studies [30–35] have achieved high-speed optical approaches for pairwise DNA alignment, which are capable of locating the variations. However, their sequence coding assumptions limit the number of input characters to that of the DNA sequences, which makes them incapable of aligning RNA and protein sequences; and misses their specific outcomes in diagnosis, medicine, and vaccination [3]. Finally, it should be noted that these methods should adopt an efficient biological data encoding by an optical modulator to avoid utilizing a large number of pixels per character. Accordingly, proposing a comprehensive pairwise alignment method can promote biological research by character-by-character alignment of various kinds of biological sequences in a fast accurate process.

**Fig 1:**
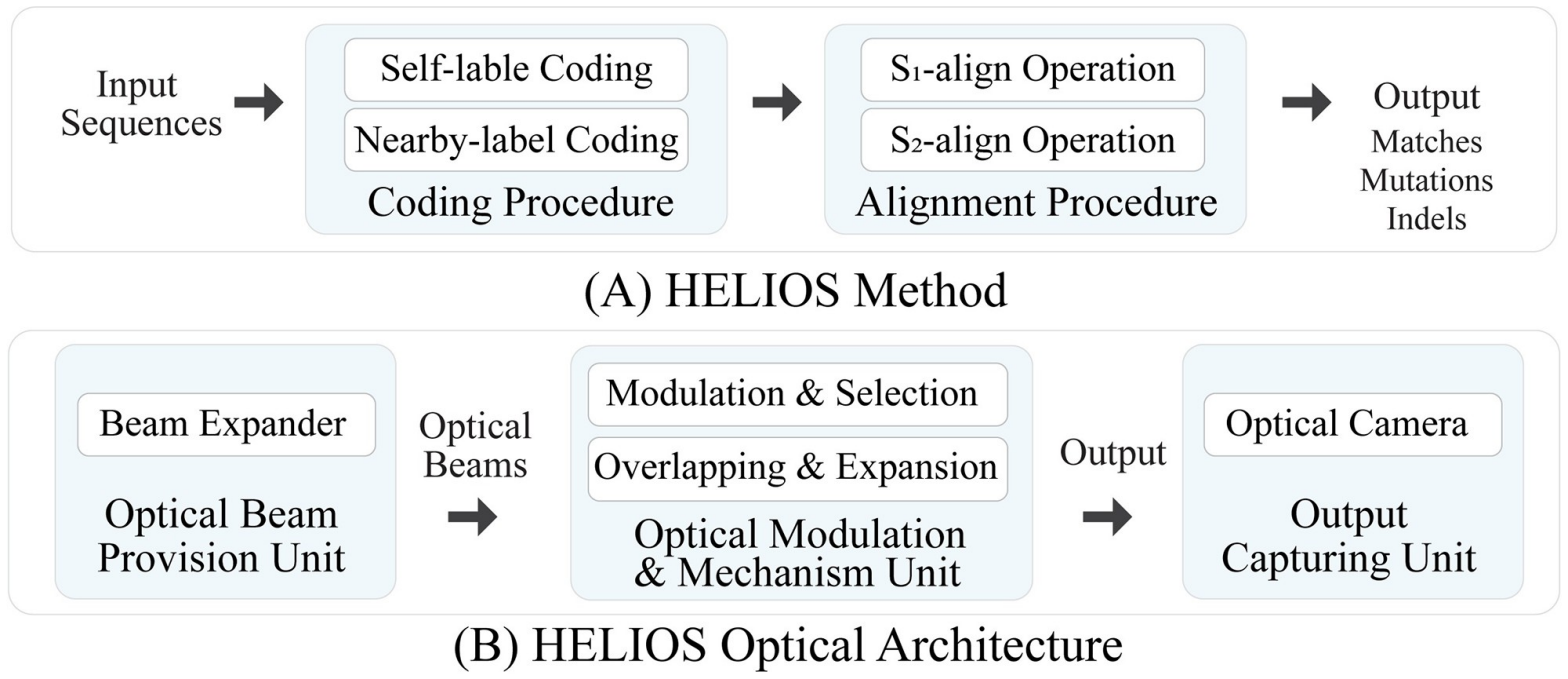
Block diagram illustration of the (A) HELIOS method and (B) HELIOS optical architecture. (A) the HELIOS method aligns two input sequences by performing the coding and alignment procedures to exactly locate character matches and variations; and (B) the HELIOS optical architecture executes the HELIOS method by performing the optical beam provision unit, the optical modulation and mechanism unit, and output capturing unit, utilizing inherent parallelism and high-speed processing in optics.

In this manner, we are motivated to propose an advanced ultra-fast all-optical method to accurately align any pair of biological sequences. The proposed method is named HELIOS, abbreviating High-speed sEquence aLIgnment in OpticS. By exploiting high-speed processing and operational parallelism in optics [24], the highly sophisticated HELIOS method avoids the problems of current sequence alignment methods, as well as the imperfections of their electrical implementations. On the other hand, adopting an efficient optical encoding of biological data, the HELIOS method outperforms the alternative optical methods in the case of time and space requirements. While the proposed method is discussed in two separate sections for more clarity, i.e., HELIOS method and HELIOS optical architecture, each one is manipulated to enhance the other one, and both form a single coherent system. The basic block illustration of the HELIOS method and the HELIOS optical architecture are presented in Fig 1A and 1B, respectively. Given the interdisciplinary nature of the HELIOS method, it can outperform many well-known alignment algorithms in terms of processing time with comparable accuracy, as verified by our comprehensive simulation studies and analytical computations. Finally, the main innovative and exclusive contributions of this paper are described as follows:

- Proposing an accurate pairwise sequence alignment method for DNAs, RNAs, and proteins.
- Designing an ultra-fast all-optical high-throughput architecture for the proposed method.
- Proposing an optical coding scheme utilizing wavelength and polarization of optical beams.

The organization of this manuscript is as follows. The *Method* section establishes the general concept of the HELIOS method, while its optical architecture is elaborated in the *Optical Architecture* section. Afterward, the *Discussion and Results* section discusses the functionality, accuracy, performance, and applicability of the HELIOS method and its optical architecture. Finally, the paper is concluded with future directions in the *Conclusion and Future Perspective* section.

## Method

Principally, the HELIOS method is composed of two main procedures to perform a parallel accurate pairwise sequence alignment, as illustrated in Fig 1A. It includes a) Coding procedure to code each character of input sequences with two parameters, and b) Alignment procedure to align two coded input sequences by performing two distinct operations in parallel. The detailed descriptions of the procedures are presented as follows.

### Coding procedure

Generally, the adopted coding scheme considerably affects the efficiency of the sequence alignment methods [2]. Thus, the coding procedure of the HELIOS method adopts a high compact distinct coding pattern to maximize parallelism and to achieve noise reduction. For this purpose, it codes every character of the input sequence according to two coding strategies: a) Self-label coding to code every character based on the character itself, and b) Nearby-label coding to provide a unique code for every character based on its nearby character.

First, the self-label coding provides a distinct code for every character within the input sequence based on the character itself. In this manner, the required number of distinct codes equals the number of nucleotides (four) in DNA and RNA or the number of amino acids (twenty) in protein sequences. Moreover, the nearby-label coding provides a distinct code for every character based on its nearby character. Specifically, it provides a unique code for each character based on its *k*^th^ previous character. So, by traversing the sequence, as the *k*^th^ previous character changes, the assigned code to the current character varies as well. Preserving locality information, it prevents data interference through the alignment procedure, as discussed in the *Alignment procedure* subsection. Same as the self-label coding, the required number of distinct codes are four, four, and twenty for coding DNA, RNA, and protein sequences, respectively.

Finally, to code the input sequence, it is traversed character-by-character, and every character is coded to an entry with two parameters according to both self-label and nearby-label coding schemes, described above. By putting together all coded entries, every sequence is represented as a one-dimensional (1D) vector with a size of 1 × length of the sequence (N). Here, we summarize the proposed coding procedure using Eq 1, formulating the coded pattern, and Eq 2, representing the adopted self-label and nearby-label coding schemes as follows:

$$\begin{array}{c} Code=[C_{self,i}\cup C_{nearby,i-k}]\;\forall \;1\leq i\leq N,and\;k>0 \end{array}$$ (1)

$$\begin{array}{c} C_{Scheme,j}=Offset_{Scheme}+\left(Step_{Scheme}\times V_{Ch_{j}}\right)\;\forall \;Scheme\in \left\{self,nearby\right\}\\ \forall \;1\leq j\leq N\;and\;\{\begin{array}{cc} 0\leq V_{Ch_{j}}\leq 19 & for\;\text{Protein}\\ 0\leq V_{Ch_{j}}\leq 4 & for\;\text{DNA}\\ 0\leq V_{Ch_{j}}\leq 4 & for\;\text{RNA} \end{array} \end{array}$$ (2)

where vector *Code* represents the coded pattern for each sequence; parameters *C*_*self*,*i*_ and *C*_*nearby*,*i*−*k*_ stand for the self-label and nearby-label coded values of the *i*^th^ character, based on the character itself and its *k*^th^ previous character, respectively. Moreover, the variable *N* represents the length of the sequence. Furthermore, variable *C*_*Scheme*,*j*_ calculates the coded value of character in position *j* within the sequence, according to coding strategy *Scheme* which is either the self-label or the nearby-label coding. In addition, the parameter *Offset*_*Scheme*_ defines the smallest value assumed for character coding, and *Step*_*Scheme*_ is the difference between two consequent code values. It is worth noting that in the case of the self-label and nearby-label coding schemes, both values of *Offset* and *Step* are independent and different. Finally, the variable *Ch*_*j*_ stands for the character in position *j* within the sequence, while the parameter *V*_*Chj*_ represents a preset value for every character within the range of 0 and the number of bases minus 1. For instance, it equals 0, 1, 2, and 3 for A, T, G, and C in DNA sequences, respectively.

**Fig 2:**
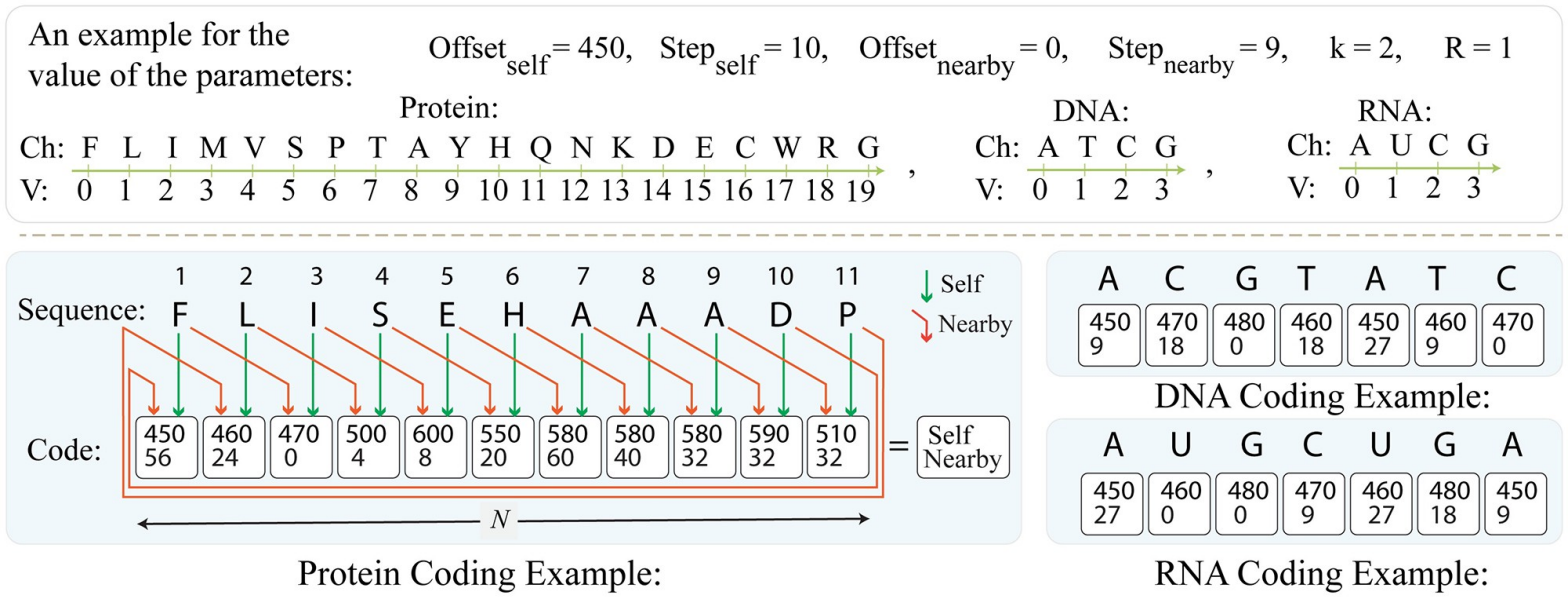
An example of the proposed coding scheme for DNA, RNA, and protein sequences. In this example, short DNA, RNA, and protein sequences are coded based on self-label and nearby-label coding schemes with preset values as follows: *Offset*_*self*_ = 450, *Step*_*self*_ = 10, *Offset*_*nearby*_ = 0, *Step*_*nearby*_ = 9, *k* = 2, and *R* = 1. The parameter *Ch*_*i*_ stands for the character positioned in location *i* as the current character in the self-label coding, and the *k*^th^ previous character in the nearby-label coding scheme. The parameter *V* represents a preset value between 0 to 19 for amino acids in the protein sequence and 0 to 3 for nucleotides in the DNA and the RNA sequences. Every character is coded with two values determined by the self-label and nearby-label coding schemes, as represented in its corresponding white block. For nearby-label coding of those characters positioned at the beginning of the sequence, the nearby-label coding wraps around the sequence and considers the desired nearby character at the end of the sequence.

According to the above discussions, the size of the coded pattern represents the length of the sequence. On the other hand, the value of each entry of the code vector indicates the corresponding character within the input sequence. These features enable random access to each character within the code vector, and hence, prevent information loss with no restriction on the length of the input sequence. Moreover, proposing a one-dimensional (1D) coding scheme enables a two-dimensional (2D) arrangement of various codded patterns for further parallel processing. Furthermore, considering the *k*^th^ previous character instead of the adjacent one in the proposed nearby-label coding scheme, it preserves the uniqueness of the code vector in the case of identical consecutive characters or pairs, like “AAAAAA” or “ACACAC”. Specifically, as the *k*^th^ previous character changes in traversing the input sequence, the assigned code to the identical consecutive characters varies as well. It prevents false character match through the alignment procedure. As parameter *k* can be set from 1 to the length of the input sequence (N), the input sequence is assumed as a circular sequence. Hence, the nearby-label coding scheme can wrap around from one end to the other, in the case of large k, to code each character based on any desired nearby character. In this case study and without loss of generality, we assume *k* equals *R* + 1, in which *R* is the number of the sequence shifts, discussed in the *Alignment procedure* subsection. Some examples of the proposed procedure for coding protein, DNA, and RNA sequences are presented in Fig 2. It should be noted that the presented values in Fig 2 are chosen according to the optical features and implementation choices, as discussed in the *Optical architecture* section.

### Alignment procedure

Once the input sequences are coded, the alignment procedure aligns two coded input sequences to determine their similarities and differences by locating character matches, mutations, and single or multiple indels. For this purpose, it performs two operations in parallel: a) S_1_-align operation to determine the state of characters (i.e., character matching, substitution, insertion, or deletion) within the first sequence, and b) S_2_-align operation to determine the state of characters within the second sequence. For simplicity, the first and second sequences are called S_1_ and S_2_ in the following, respectively.

#### S_1_-align operation

**Fig 3:**
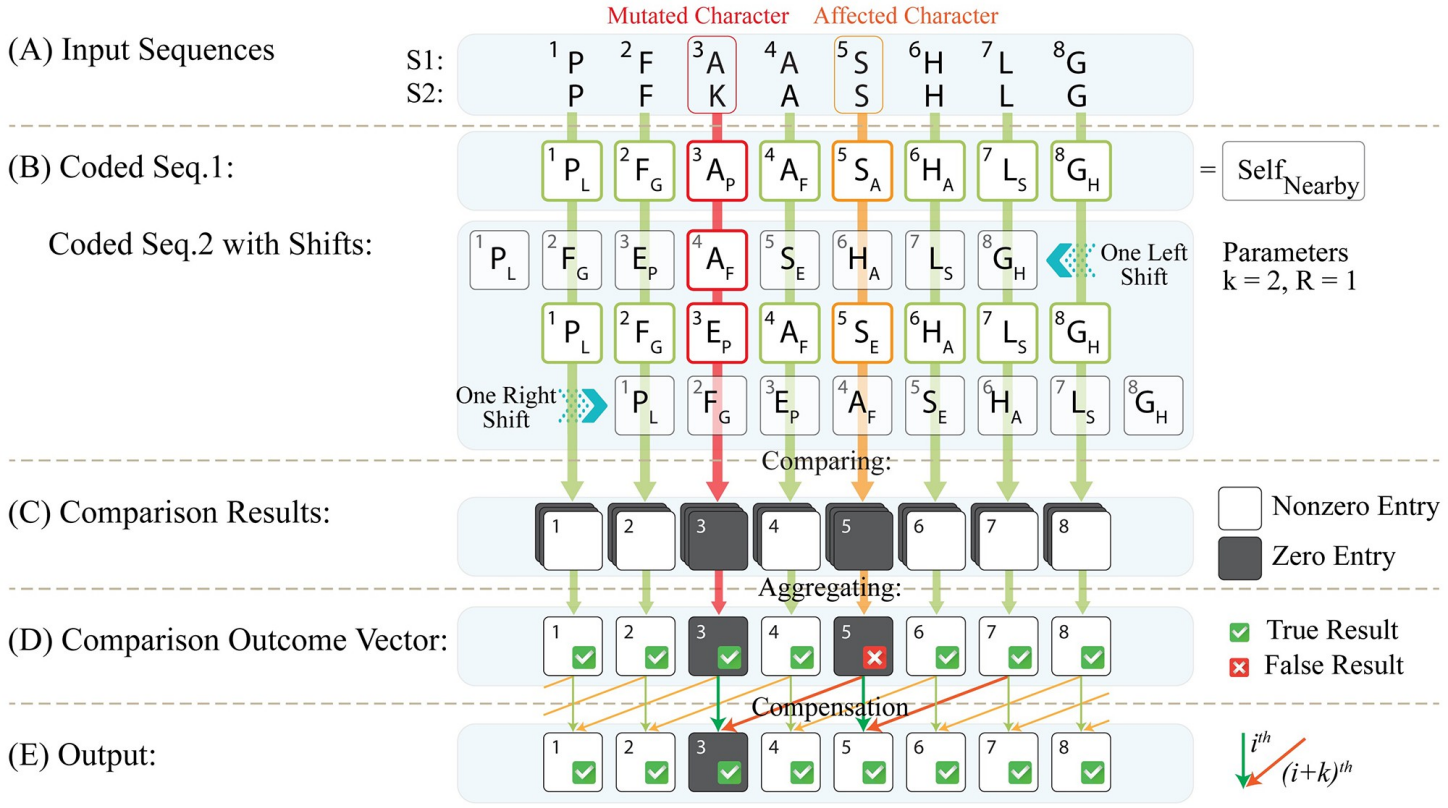
Step-by-step progress of the S_1_-align operation of the HELIOS method for optical sequence alignment. (A) Two input sequences, i.e S_1_ and S_2_, are given to the HELIOS method, assuming the third character (i.e. ‘A’ in S_1_ and E in S_2_) is mutated. (B) S_1_ and S_2_ are coded based on the proposed coding procedure, assuming *k* = 2 and *R* = 1. While the self-label codes of the third character are different for S_1_ and S_2_, the nearby-label codes of the fifth characters (i.e. ‘S’) are different as well, due to the mutated character, assuming *k* = 2. Afterward, the coded S_2_ is shifted one time horizontally towards the left and right of the coded S_2_ assuming R = 1. Then, the main S_1_ is compared with the main S_2_ and all its shifts, and hence, (C) the comparison results are presented for each comparison. (D) Next, the comparison output vector is formed by aggregating all the comparison results, where the matched characters result in nonzero entries. As represented with the zero entry, the mutated character in position 3 within the input sequences is successfully located due to the different self-label codes, while the 5^th^ character is false mismatched due to the different nearby-label codes. (E) To compensate for this false mismatch, the *i*^th^ entry of the output is determined according to aggregating the *i*^th^ and the (*i* + *k*)^th^ entries of the comparison outcome vector. Hence, the 5^th^ entry is recovered by the corresponding nonzero value at the 7^th^ entry; while proper detection of character mutation at the 3rd entry is not affected.

To specify the state of characters within S_1_, the S_1_-align operation determines whether every character in S_1_ corresponds to an identical character in S_2_; while in the case of character mutations (i.e., substitution) or indels (i.e., insertion or deletion), this correspondence does not exist. Moreover, while mutations only substitute the character itself; indels cause right-shifting or left-shifting of the rest of the sequence as well [2]. So, both character substitution and character-shifting should be addressed in the case of mutations and indels, respectively. In this manner, the S_1_-align operation shifts the coded S_2_ vector one to *R* times in the horizontal direction towards the left and right of the main S_2_ vector, as depicted in Fig 3B. Afterward, the main S_2_ vector and all its shifts are compared to the non-shifted S_1_ vector correspondingly. Performing this comparison, as shown in Fig 3C, a nonzero entry appears in the comparison results if the corresponding self-label and nearby-label codes of the input sequences are identical. Otherwise, the corresponding entry of the comparison results remains zero in the case of non-identical characters. It is worth noting that while the comparison of S_1_ and S_2_ enables the detection of matched characters and mutations, single or multiple indels are detected by comparing the S_1_ vector to the shifted S_2_ vectors. Finally, each entry of the comparison outcome vector is formed by aggregating corresponding entries of all vectors of the comparison results, which are resulted from comparing the non-shifted S_1_ vector with the main S_2_ vector and all its shifts. So, the comparison outcome vector is represented in a row, as depicted in Fig 3D. As a key advantage, distinct code assignment to similar characters within the input sequences by the nearby-label coding scheme prevents data interference through the horizontal shifting and comparing processes.

Summarizing the above discussion, we can conclude that the S_1_-align operation successfully locates character substitution (both single and multiple) and character insertion (both single and multiple) in S_1_ (i.e. character deletion from S_2_). However, specifying characters deletion from S_1_ requires a further comparative operation, named S_2_-align operation, as follows.

#### S_2_-align operation

**Fig 4:**
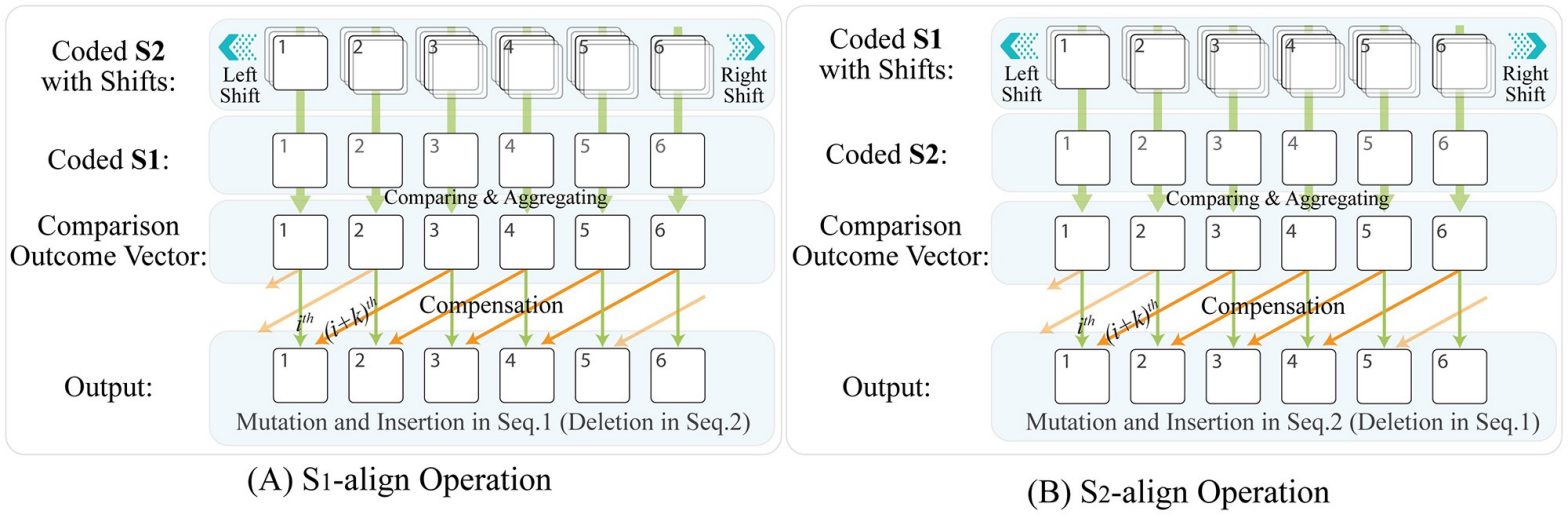
Side-by-side representation of the S_1_-align and S_2_-align operations of the HELIOS method. (A) As an overall view, the S_1_-align operation locates character substitutions, as well as character insertions in S_1_ (or character deletions from S_2_). For this purpose, it compares the main and all shifted S_2_ vectors with the S_1_ vector. Afterward, to produce the 1D output vector, the *i*^th^ entry of the output is determined according to the *i*^th^ and (*i* + *k*)^th^ entries of the comparison outcome vector. (B) Similarly, the S_2_-align operation compares the main and all shifted S_1_ vectors with the S_2_ vector to locate character substitutions, as well as character insertions in S_2_ (i.e. character deletions from S_1_).

As a complementary comparative operation, the S_2_-align operation determines the state of every character in S_2_ by finding its corresponding character in S_1_. For this purpose, the S_2_-align operation repeats the comparative S_1_-align operation, except that it shifts the S_1_ pattern (instead of S_2_) one to *R* times in the horizontal direction towards the left and right of the main S_1_ vector, as shown in Fig 4. Afterward, the main S_1_ vector and all its shifts are compared to the non-shifted S_2_ vector correspondingly. Thus, this operation successfully locates character mutations (both single and multiple), as well as character insertions (both single and multiple) in S_2_ (i.e. character deletions (both single and multiple) from S_1_). Specifically, character mutations and insertions are represented with zero entries in the comparison outcome vector.

It is worth noting that the number of consecutive indels (i.e. consecutive insertions or consecutive deletions) is assumed to not be larger than R, and hence, the value of R should be large enough to support all probable variations between two sequences. However, small values of R can be chosen in the case of aligning two similar sequences. Moreover, for aligning two input sequences with different lengths, the shorter one should slide all over the longer one to determine every probable variation, which results in a large value of R. As each sequence shifts in the horizontal direction towards the left and right of the other sequence, the parameter R varies in the range of [1, $\frac{N}{2}+1$]. However, the various choices of R (from 1 to $\frac{N}{2}+1$) do not affect the processing time and the speed, as discussed in more detail in the *Optical architecture* section.

#### Output vector production

Once the S_1_-align and S_2_-align operations are performed, every entry of the comparison outcome vector can be determined accordingly, as depicted in Fig 3. Specifically, as shown in Fig 3A and 3D, a mutation or an indel within the input sequence results in a zero value at the corresponding entry of the comparison outcome vector. For example, the 3^rd^ character (i.e. character ‘A’) within S_1_ is mutated against the character ‘E’ in S_2_. However, regarding the nearby-label coding scheme, the code of the *k*^th^ next character is also affected. Hence, assuming *k* = 2, the 5^th^ characters of the input sequences (i.e. characters ‘S’ of S_1_ and S_2_) are nearby-label coded differently as shown in Fig 3B. Consequently, this variation causes a false mismatch, as well as a false zero value at the 5^th^ entry of the comparison outcome vector as shown in Fig 3C and 3D, respectively.

To compensate for the false mismatched characters, the corresponding character is involved whose nearby-label code is determined based on the false mismatched characters, which is (*i* + *k*)^th^ charater (i.e. character ‘L’ at the 7^th^ entry of S_1_ and S_2_ in Fig 3A and 3D). In this manner to produce the final output, the *i*^th^ entry of the output is determined according to aggregating the *i*^th^ and (*i* + *k*)^th^ entries of the comparison outcome vector. For example, as depicted in Fig 3D and 3E, the false mismatch at the 5^th^ entry is recovered by the corresponding nonzero value at the 7^th^ entry; while proper detection of character mutation at the 3^rd^ entry is not affected.

As a final word, it should be noted that all aforementioned steps to produce the final output, i.e. shifting, comparing, aggregating, etc., are done in parallel with no hardware complexity, taking advantage of the inherent parallelism in optics.

#### Analysis and review of the output

As follows, we summarize the proposed alignment procedure: a) S_1_-align operation compares the mail and all shifted S_2_ vectors with the S_1_ vector to locate character substitutions, as well as character insertions in S_1_ (or deletions from S_2_), as depicted in Fig 4A and 4b) S_2_-align operation compares the main and all shifted S_1_ vectors with the S_2_ vector to locate character substitutions, as well as character insertions in S_2_ (or deletions from S_1_), as depicted in Fig 4B. To produce the output, the *i*^th^ entry of the output is determined according to the *i*^th^ and (*i* + *k*)^th^ entries of the comparison outcome vector. Producing a 1D vector for each comparison, performed by the S_1_-align or S_2_-align operations, the output can be arranged as a two-row matrix, as formulated in Eq 3; while Eq 4 calculates every entry of the output, as follows:

$$\begin{array}{c} Output=[\begin{array}{c} Out_{1,i}\vee Out_{1,i+k}\\ Out_{2,i}\vee Out_{2,i+k} \end{array}],\;\forall \;1\leq i\leq N,\;k>0,\;R>0 \end{array}$$ (3)

$$\begin{array}{c} Out_{row,j}=\overset{j+R}{\underset{x=j-R}{\vee }}A_{row,j,x}\;\forall \;row\in \left\{1,2\right\}\\ \forall \;A_{row,j,x}=\{\begin{array}{cc} 0 & \;\text{if}\;Code_{row,j}\neq Code_{\frac{2}{row},x}\\ 1 & \;\text{if}\;Code_{row,j}=Code_{\frac{2}{row},x} \end{array} \end{array}$$ (4)

where, *output* vector represents the output of the sequence alignment in two rows, the parameter *Out*_*row*,*i*_ represents its *i*^th^ entry as a result of comparing the *i*^th^ character of the input sequences, while *Out*_*row*,*i*+*k*_ compensates the probable false mismatching. Parameter *row* stands for the output rows’ indices, resulted from the S_1_-align or S_2_-align operations. Moreover, variable *Out*_*row*,*j*_ represents the comparison outcome for the *j*^th^ character by aggregating all corresponding entries, comparing the non-shifted vector of one sequence with the main and all shifted vectors of the other one as discussed in the *S_1_-align* and *S_2_-align operation* subsections. For this purpose, variable *A*_*row*,*j*, *x*_ represents the comparison result of two coded characters: *Out*_*row*,*j*_ as the *j*^th^ coded character within the non-shifted sequence, and *Code*_2/*row*, *x*_ as the *x*^th^ coded character within the main or shifts of the other sequence. For instance, for calculating *output* [1, 3], as the state of 3^rd^ entry of S_1_, the S_1_-align operation aggregates *out*_1,3_ and *out*_1,5_ based on Eq 3, assuming *k* = 2 and *R* = 1. While Eq 4 calculates *out*_1,3_, assuming *row* = 1 and *i* = 3, by accumulating the results of comparing codes of the 3^rd^ entry of S_1_ (i.e. *Code*_1,3_) with the 2^nd^, 3^rd^, and 4^th^ entries of S_2_ (i.e. *Code*_2,2_, *Code*_2,3_, *Code*_2,4_, respectively). Similar operation calculates *out*_1,5_. To finalize, the pseudo-code of the coding and alignment procedures is depicted in Algorithm 1.

**Algorithm 1** **Pseudo-code of the HELIOS method**, including the coding and alignment procedures.

**Require:** *S*_1_ ∧ *S*_2_ ∧ *R* ≥ 0 ∧ *k* > 0

 **for** each input sequence called *S*_*input*_ (*input* = 1 → 2) **do**

  **for** character *i* = 1 → *N* **do**

   $C_{self,i}⇐Offset_{self}+\left(step_{self}\times V_{Ch_{i}}\right)$

   $C_{nearby,i-k}⇐Offset_{nearby}+\left(step_{nearby}\times V_{Ch_{i-k}}\right)$

   *S*_*input*_.*Code*[*i*] ⇐ *C*_*self*_ ∪ *C*_*nearby*_

  **end for**

 **end for**

 **for** each operation called *row* = 1 → 2 **do**

  **for** entry *i* = 1 → *N* **do**

   **for** entry *j* = (*i*−*k*) → (*i* + *k*) **do**

    **if** *S*_*row*_.*Code*[*i*] = *S*_2/*row*_.*Code*[*j*] **then**

     *Out*[*row*, *i*] ⇐ 1

    **end if**

   **end for**

  **end for**

  **for** entry *i* = 1 → *N* **do**

   *Output*[*row*, *i*] ⇐ *Out*[*row*, *i*] ∨ *Out*[*row*, *i* + *k*]

  **end for**

 **end for**

**Fig 5:**
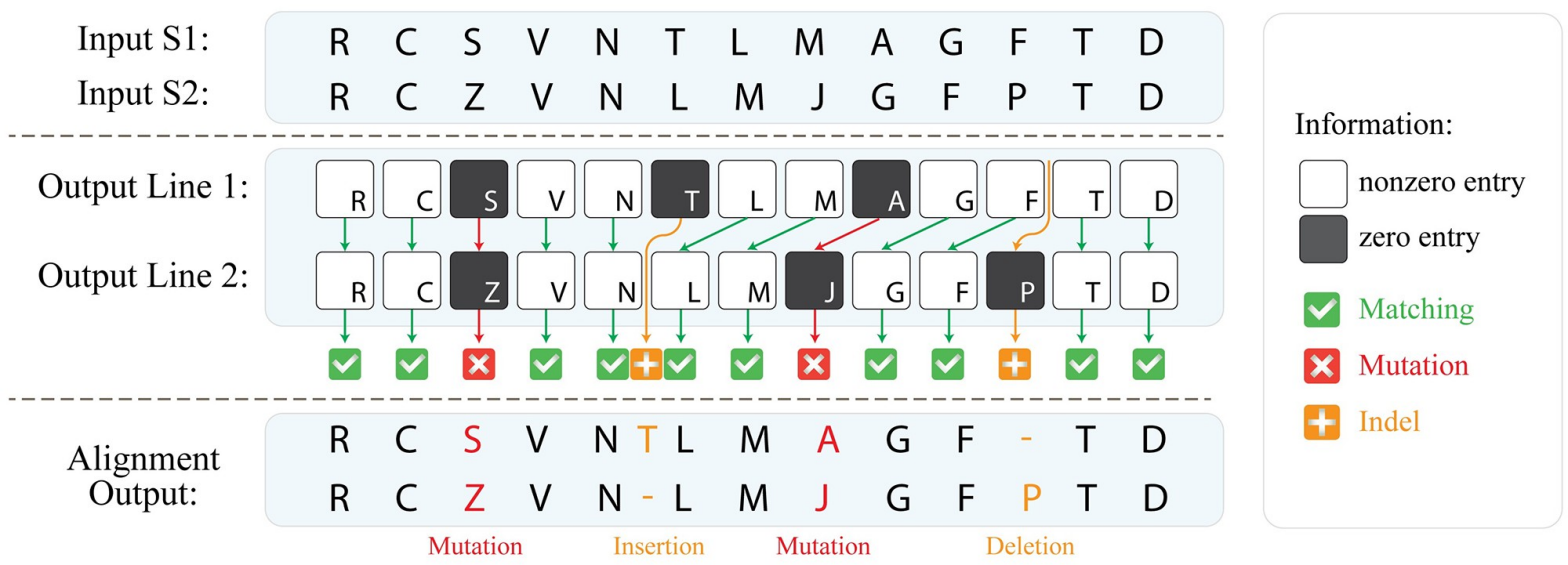
Output explanation of the HELIOS method. The output of the HELIOS method is represented with a two-row matrix, while the first and second rows are produced by the S_1_-align and S_2_-align operations, respectively. Moreover, each entry represents the alignment output of the characters at the corresponding position in the input sequences. By traversing the output from left to right, nonzero entries in both rows depict identical characters, i.e. character matching; while zero entries in both rows depict character mutation. Finally, a zero entry of a row along with a nonzero entry of the other one indicates indel, i.e. character insertion or deletion, and can be represented by a gap at the corresponding position of the sequence, containing the nonzero entry.

Performing the HELIOS method, the output appears as a two-row matrix, while each entry represents the alignment output of two characters at the corresponding position within the input sequences, as shown in Fig 5. By traversing the output from left to right, nonzero entries in both rows depict identical characters, i.e. character matching, at the corresponding position of the input sequences. On the other hand, zero entries in both rows depict character mutation at the corresponding position of the input sequences. Finally, a zero entry of a row along with a nonzero entry of the other one indicates indel, i.e. character insertion or deletion, and can be represented by a gap at the corresponding position of the sequence, containing the nonzero entry, as shown in Fig 5.

Summarizing the HELIOS method, we would like to emphasize that it exactly locates character matches, mutations, and single/multiple indels through the alignment procedure; while the coding procedure presents distinct coding patterns for input sequences and reduces the noises at the output vector, represented in a convenient form.

## Optical architecture

**Fig 6:**
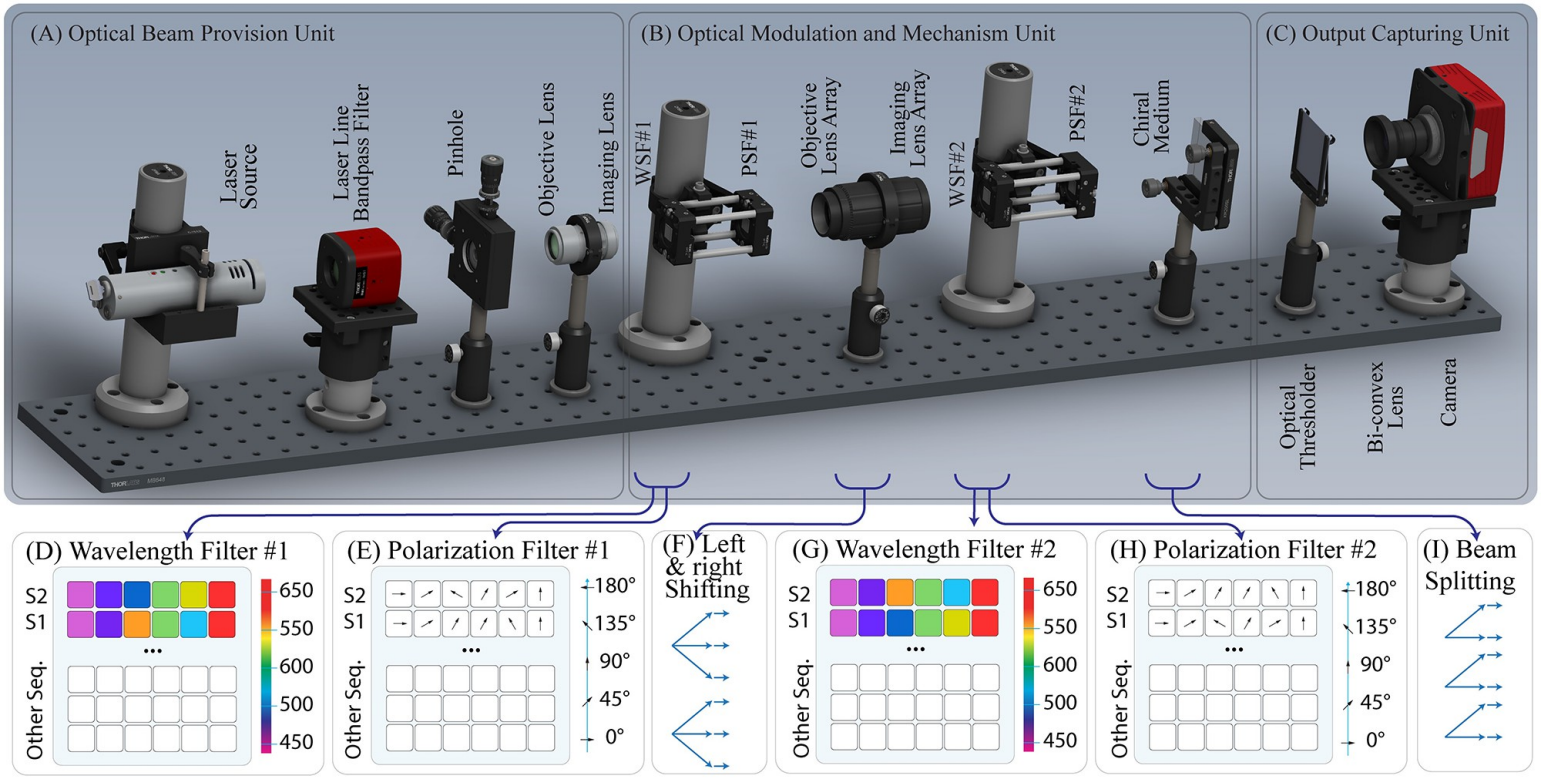
Schematic illustration of the HELIOS optical architecture. (A) The optical beam provision unit provides a collimated beam to feed the whole system. In this manner, the wideband laser beam, produced by a laser source, passes through the laser line bandpass filter and the pinhole to be cleaned. Afterward, the clean beam is diverged and collimated with passing through the objective and imaging lenses, respectively. Finally, the collimated beam is directed to the optical modulation and mechanism unit. (B) In the optical modulation and mechanism unit, passing collimated beam through WSF #1 modulates the wavelength of the optical beam based on the self-label coding of S_2_ and S_1_ on the first and second rows of a 2 × N pixels image, respectively; while PSF #1 performs their polarization selection based on their nearby-label coding scheme. Afterward, the objective and imaging lens arrays diverge and recollimate the optical beam through a horizontal direction to perform the shifting process of the alignment procedure. Moreover, WSF #2 and PSF #2 code S_1_ and S_2_ on the first and second rows of a 2 × N pixels image, respectively. By passing the expanded beams through WSF #2 and PSF #2, the proposed architecture compares the shifted coded S_2_ with S_1_ at the first row, implementing the S_1_-align operation, and compares the shifted coded S_1_ with S_2_ at the second row, implementing the S_2_ -align operation. Finally, each pixel is directed to two distinct pixels via a chiral medium to compensate for false mismatches. (C) Finally, in the output capturing unit, optical thresholdder eliminates wavelength cross-talks and speckle noises of the output before capturing. Afterward, the output is captured by a bi-convex lens and a charged-coupled device (CCD) camera.

Equivalent to the HELIOS method, the HELIOS optical architecture is developed to exploit the inherent parallelism and ultra-fast processing capabilities of optics, as depicted in Figs 1B and 6. The HELIOS optical architecture consists of three main units: a) Optical beam provision unit to prepare collimated beam to feed the proposed optical architecture, b) Optical modulation and mechanism unit to accomplish the coding and alignment procedures of the HELIOS method, and c) Output capturing unit to capture the final output of HELIOS optical architecture. The units are explained in more detail as follows.

### Optical beam provision unit

The optical beam provision unit provides a collimated optical beam to feed the whole optical system. In this manner, it employs a wideband unpolarized laser source [37], a laser line bandpass filter [38], a pinhole, and two lenses, as depicted in Fig 6A. For this purpose, the wideband laser generates an intense coherent monochromatic light beam in a wide spectral range; while the laser line bandpass filter transmits laser light with suppressing ambient light as well as lower intensity secondary laser lines. It improves contrast by only transmitting light within a specific wavelength range e.g. 450 to 650 nanometers in this case of study. Moreover, the thermal load is minimized on the blocking glass and the epoxy by facing the highly reflective side of the filter to the laser source. Afterward, the Galilean beam expander model [39] is employed to provide the collimated beam. It utilizes a pinhole and two lenses, including a) an objective lens, which is a bi-concave lens with a negative focal length (−*f*1) to diverge the beam, and b) an imaging lens, which is a plano-convex lens with a positive focal length (+ *f*2) to collimate the diverged beam; while c) a pinhole, placed at the focal point of the lenses, spatially filters the beams to reduce its high pulse energy density and to prevent arcing the air. The absence of the focal point between the lenses because of different signs of the focal lengths (−*f*1 + *f*2), avoids high energy density between the lenses, as well as results in a compact design, erect output, and elimination of the correction lens.

Summarizing the above discussion, the wideband laser beam passes through the laser line bandpass filter and the pinhole to be cleaned. Afterward, the clean beam is diverged and collimated by passing through the objective and imaging lenses, respectively. Finally, the collimated beam is directed to the optical modulation and mechanism unit to fill the aperture of the modulator cells with a proper amplitude, wavelength, and polarization of the optical beams.

### Optical modulation and mechanism unit

In the HELIOS optical architecture, the coding and alignment procedures of the HELIOS method are performed by transmitting collimated beams through the optical modulation and mechanism unit, as depicted in Fig 6B. In this unit, the self-label and nearby-label coding schemes are performed by modulating the wavelength and polarization of the optical beams, respectively. Specifically, it is performed by passing the collimated beam through electrically controlled spatial filters [40, 41]; while the S_1_-align and S_2_-align operations of the alignment procedure are simultaneously performed by expanding and overlapping the modulated beams.

#### Modulation approach

To perform the self-label coding, a wavelength selection approach is employed to modulate every character of the input sequences at a distinct wavelength. In this manner, a recently developed electrically controllable wavelength selective filter (WSF) is adopted [40], which is built upon a liquid crystal. The filter covers the spectral band in the range of [450–1000] nanometers with bandwidth less than 10 nanometers and throughput more than 80 percents. Employing electronically controlled liquid crystal, the filter transmits only a selected wavelength of light and excludes others at each pixel.

Besides, the nearby-label coding is implemented utilizing the polarization of the optical beams. In this manner, a proposed polarization-based spatial filter (PSF) is employed [41], which is built upon an S-waveplate. It modulates every character of the input sequence with a unique linear polarization. This filter operates by transmitting a specific polarization along an azimuth angle *θ* in the range of [0, 180] degrees; while rejecting other polarizations. As the S-waveplate is a polarization-sensitive element, the transmittance of the S-waveplate at each pixel can be controlled by adjusting a bias voltage on the waveplate. Hereupon, it passes the incident beam at a specific polarization at each pixel. So, this property enables us to electrically modulate the polarization of the optical beams.

**Fig 7:**
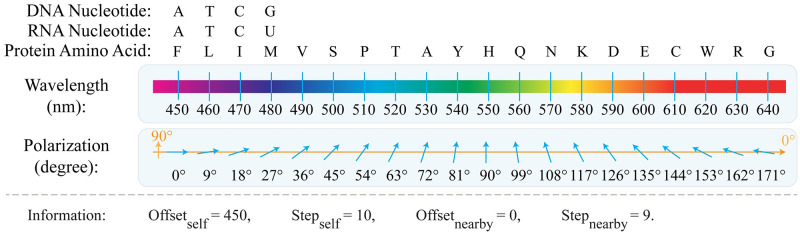
Modulation approach of the HELIOS Optical architecture, utilizing the wavelength and polarization of the optical beams. To implement the self-label coding through the wavelength modulation approach, every character of the input sequence is modulated with a distinct wavelength, within the spectral range of [450–650] nanometers with bandwidth of 10 nanometers. On the other hand, to implement the nearby-label coding through the polarization selection approach, every character of the input sequence is assigned to a specific polarization along a 9-degree azimuth angle in the range of 0 to 180 degrees. Each approach provides twenty distinct codes for protein sequences, while only four of them are employed for coding DNA and RNA sequences.

Therefore, the proposed architecture modulates specific wavelengths and polarizations of the unpolarized light beams, transmitted by the optical beam provision unit. Moreover, where a modulated beam crosses wavelength-selective and polarization-based spatial filters, it can only pass through the filters in the case of identical wavelengths and polarizations. Hence, it enables the comparison of two coded patterns in the optical architecture. Despite providing many distinct codes, in this study we assume modulation wavelength in the range of [450, 650] nanometers with 10 nanometers channel spacing, and linear polarization selection in the range of [0, 180] degree with angle variation of 9 degrees. This assumption provides the required orthogonal code sets for the self-label and nearby-label codings of DNA, RNA, and protein sequences, as depicted in Fig 7.

#### Mechanism of the unit

In the mechanism of the unit, at first, a space of 2 × N pixels is reserved on the WSF #1 and PSF #1, as depicted in Fig 6D and 6E, respectively. While the first row modulates all characters within S_2_ for performing the S_1_-align operation, the second row modulates all characters within S_1_ for performing the S_2_-align operation. Hence, transmitting collimated beam through WSF #1 and PSF #1 modulates wavelength and polarization of optical beams based on the self-label and nearby-label coding of the input sequences, respectively.

To realize shifting process of the modulated beams along the horizontal direction as discussed in the *alignment procedure* subsection, every modulated beam is expanded to multiple horizontal beams by two micro-lens arrays, as depicted in Fig 6B and 6F: a) an objective microlens array, composed of 2 × N bi-convex lenses with a negative focal length to diverge the modulated beams in the horizontal direction, and b) an imaging microlens array, composed of 2 × N bi-concave lenses with a positive focal length to recollimate the converged beams. It is worth noting that to implement the various numbers of sequence shifts, the objective and image lens arrays with focal lengths of different signs can be adopted to expand every modulated beam to the required range of horizontal pixels. In this process, the number of shifts, i.e. value of parameter R, does not affect the performance of the optical system, since expanding and recollimating the optical beam are performed in parallel.

Afterward, the recollimated modulated S_2_ and S_1_ beams (produced by WSF #1, PSF #1, and microlens arrays) are fed to WSF #2 and PSF #2. The WSF #2 and PSF #2 modulate the self-label and nearby-label codes of S_1_ and S_2_ on the first and the second rows of reserved 2 × N pixels, respectively, as depicted in Fig 6G and 6H. Hence, by passing the recollimated modulated beams through WSF #2 and PSF #2, the proposed architecture compares the shifted coded S_2_ with S_1_ at the first row, implementing the S_1_-align operation. Concurrently, it compares the shifted coded S_1_ with S_2_ at the second row, implementing the S_2_-align operation. Specifically, in the case that the crossed beam (modulated by PSF #1 and WSF #1) and the pixel on PSF #2 and WSF #2 have identical wavelength and polarization, respectively, the beam passes through the filters, indicating a non-blocking state, and so, a nonzero amplitude pixel appears at the comparison outcome. Otherwise, the optical beam fails to pass through WSF #2 or PSF #2, indicating a blocking state, and so, a zero amplitude pixel appears at the comparison outcome vector. In this manner, the presence of a pixel with nonzero amplitude at the output clarifies a matching state through the alignment procedure; while a zero amplitude pixel clarifies a mismatch (i.e. mutation or indel) correspondingly.

As discussed in the *Output vector production* subsection, false mismatches on the *k*^th^ next pixels to the right of mutated characters lead to false zero amplitude pixels at the output. Hence, the value of these pixels should be recovered to produce the proper output. In this manner, the property of double refraction of optical beams in optically active media is employed [42]. When a linearly-polarized beam of light enters an optically active medium, like a chiral liquid crystal, it is split into two separate beams of opposite circular polarizations, traveling at different speeds through the medium. Hence, the beams are refracted and diverged by an angle according to the different propagation speeds of the right-circularly and left-circularly polarized light beams [42]. So, every beam, entered the optically active medium, leaves it from two different locations with a specific distance apart. While light speed determines the angle of refraction, the desired distance between the two exit points can be set by the property and geometry of the optically active medium. Considering the double refraction property, every beam representing the *i*^th^ pixel is split into two beams at the *i*^th^ and the (*i* − *k*)^th^ pixels, as depicted in Fig 6I.

Summarizing the above discussion, the S_1_-align and S_2_-align operations of the alignment procedure are completely and concurrently performed by passing the collimated beams through the spaces of 2 × N pixels of the WSFs, PSFs, microlens arrays, and optically active media. It is worth noting that the inherent parallelism of optics enables multiple input sequences to be arranged on the aperture of the modulator cells and to be aligned through the HELIOS optical architecture simultaneously. Therefore, efficient use of coding space, as well as considerable speed-up can be achieved by the proposed optical architecture.

### Output capturing unit

Finally, optical thresholding is performed by an optical thresholder, shown in Fig 6C to provide a clean outcput. In this manner, it eliminates wavelength cross-talks and speckle noises of the output before capturing. At last, the output is converged to a proper aperture by a bi-convex lens to be captured by a charge-coupled device (CCD) camera [43], as depicted in Fig 6C. The resultant output, represented as 2 × N pixels image, includes nonzero and zero amplitudes, while each pixel represents the state of the corresponding character within the input sequences.

## Discussion and results

Comprehensively, various simulation approaches and numerical analyses are investigated to assess the HELIOS method and its optical architecture. At first, the functionality of the HELIOS method and its optical architecture is validated through investigating various simulation outputs. Next, the accuracy of the HELIOS method is inspected with comprehensive simulation studies and statistical analyses for various datasets. Afterward, the time and space complexities and the performance of the HELIOS optical architecture are estimated by analytical computation. For a comparative study, we consider various well-known algorithms, including BLAST [7], ClustalW [8], ClustalΩ [9], MUSCLE [12], T-Coffee [10], Kalign [11], MAFFT [13], Smith-waterman [5], Needleman-Wunsch [6] Nucmer4 [14], BLASR [44], BWA-MEM [45], Bowtie2 [46], Mauve [47], LASTZ [48], Moiré Technique [31], HAWPOD [30], and OptCAM [34]. Finally, some well-known applications are presented that can potentially benefit from the HELIOS method.

### Functional validation

**Fig 8:**
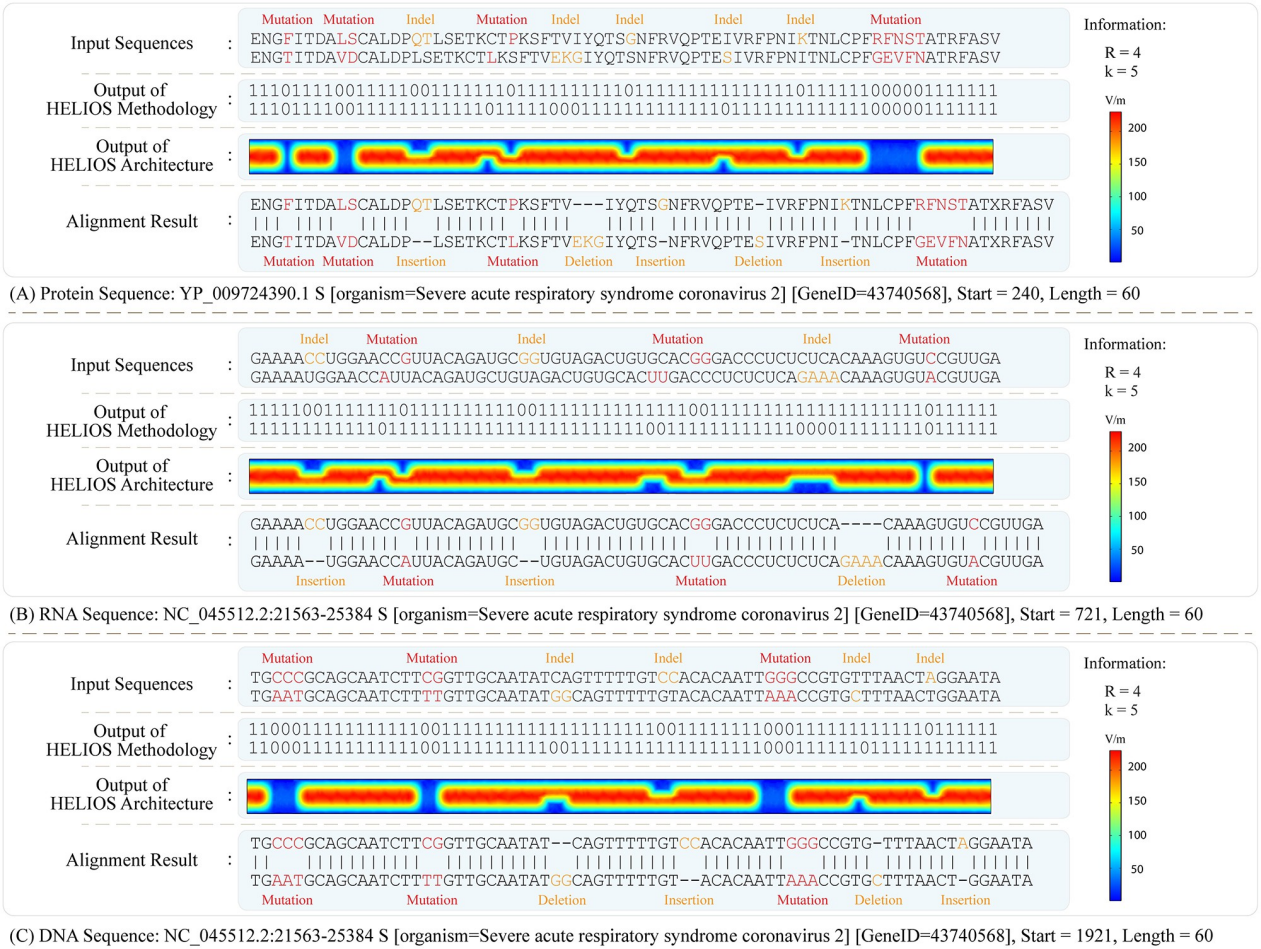
Simulation outputs of the HELIOS method and its optical architecture. In this case of study, the “Severe acute respiratory syndrome coronavirus 2” sequences [49] are aligned in the form of (A) Protein, (B) RNA, and (C) DNA; while some single and multiple mutations/indels are manually imposed to the sequence with varying distributions. For more clarity, only a small portion of each full-length alignment including 60 characters is shown in this figure, with the beginning at (A) character 240, (B) character 721, and (C) character 1921. In the coding and the alignment procedure, the parameters are set to *R* = 4 and *k* = 5. By investigating the outputs, two input sequences are successfully aligned in a two-line output by performing two consecutive procedures of the HELIOS method, as well as, passing optical beams through two units of the HELIOS optical architecture. As a result, the matches, mutations, and indels are detected and located accurately.

In order to evaluate the functionality of the HELIOS method and its optical architecture, the alignment outputs of numerous DNA, RNA, and protein sequences are investigated. In this manner, the HELIOS method and its optical architecture are simulated in MATLAB simulation tool and COMSOL Multiphysics software, respectively. As a case study, the “Severe acute respiratory syndrome coronavirus 2” sequences [49] are aligned and represented in the form of protein, RNA, and DNA, in Fig 8A–8C, respectively; while some single and multiple mutations/indels are manually imposed to the sequence with varying distributions. For more clarity, only a small portion of each full-length alignment, including 60 characters is represented in Fig 8. As shown in Fig 8, two input sequences are successfully aligned in a two-line output by performing two consecutive procedures of the HELIOS method, as well as, passing optical beams through two units of the HELIOS optical architecture. Moreover, the wavelength modulation within the range of 450 to 650 nanometers with 10 nanometers spacing, accompanied with polarization selection in the range of 0 to 180 degrees with 9-degree angle variation are performed for modulating optical beams in the HELIOS optical architecture. It is noteworthy that the crosstalk between neighboring pixels due to an electric field leakage, produced by filtering a specific wavelength and polarization, is negligible and does not affect our results [50].

Analyzing the simulation outputs, all the character matches, mutations, and indels are accurately detected and located within the protein, RNA, and DNA sequences. As depicted in Fig 8, the character matches are presented with the nonzero entries and high amplitude pixels within the outputs of the HELIOS method and its optical architecture, respectively; while the zero entries and zero-amplitude pixels represent mutations or indels. Eventually, investigating the simulation outputs verifies the accurate functionality of the HELIOS method in both levels of method and optical architecture.

### Accuracy evaluation

In order to comprehensively assess the accuracy of the HELIOS method, two statistical analyses are performed through simulations of various datasets: 1) Quantitative measurement of homology [51], and 2) Accuracy measurement of classification output [52], compared to the well-known algorithms.

#### Quantitative measurement of homology

To perform quantitative measurement of homology [51], the parameters Identity, Similarity, and Alignment Score of the HELIOS outputs are calculated through simulation studies, as reported in Tables 1–3, respectively, assuming the *“Nine ND5 protein sequences dataset”* [53]. While the Identity reports the number of exactly matched characters of two sequences (in percentage), the Similarity measures the resemblance of two compared sequences. Specifically, regarding the physicochemical properties, the amino acids are categorized into six groups with different similarity values; including GAVLI, FYW, STCM, KRH, DENQ, and P. As the third metric, the BLOSUM62 [54] substitution scoring matrix [54] is adopted to calculate the Alignment Score, with gap opening and extension penalties equal to -10 and -0.5, respectively.

**Table 1:** The parameter Identity of the HELIOS method in the quantitative measurement of homology. The Identity reports the number of exactly matched characters of two compared sequences (in percentage) aligned by the HELIOS method, assuming the *“Nine ND5 protein sequences dataset”* [53].

	Homo sapiens	Rattus norvegicus	Balaenoptera physalus	Balaenoptera musculus	Didelphis virginiana	Pan troglodytes	Pan paniscus	Gorilla gorilla	Mus musculus
Homo sapiens	100	64.1791	69.8176	69.3201	62.2924	93.3665	93.2007	90.2156	64.8425
Rattus norvegicus		100	66.8317	66.5017	60.7973	63.8474	64.0133	63.1841	78.2537
Balaenoptera physalus			100	96.5347	61.6279	70.1493	70.6468	69.3201	66.5017
Balaenoptera musculus				100	62.2924	69.9834	69.8176	68.9884	66.8317
Didelphis virginiana					100	62.4585	62.7907	60.9635	63.6213
Pan troglodytes						100	95.0249	90.7131	64.3449
Pan paniscus							100	91.0448	64.5108
Gorilla gorilla								100	64.3449
Mus musculus									100

**Table 2:** The parameter Similarity of the HELIOS method in the quantitative measurement of homology. The Similarity measures the resemblance of two compared sequences (in percentage) aligned by the HELIOS method. Various amino acids are categorized into six groups based on their physicochemical properties; including GAVLI, FYW, STCM, KRH, DENQ, and P. Moreover, the *“Nine ND5 protein sequences dataset”* [53] is assumed in this study.

	Homo sapiens	Rattus norvegicus	Balaenoptera physalus	Balaenoptera musculus	Didelphis virginiana	Pan troglodytes	Pan paniscus	Gorilla gorilla	Mus musculus
Homo sapiens	100	72.6368	77.7778	76.7828	72.5914	95.1907	95.8541	92.3715	73.9635
Rattus norvegicus		100	77.8878	77.2277	70.7641	72.9685	72.6368	72.9685	84.8435
Balaenoptera physalus			100	97.0297	75.4153	77.9436	78.2753	77.6119	77.3927
Balaenoptera musculus				100	75.2492	76.7828	76.7828	76.4511	78.0528
Didelphis virginiana					100	72.5914	74.0864	71.9269	73.0897
Pan troglodytes						100	96.5174	93.2007	74.1294
Pan paniscus							100	93.864	73.466
Gorilla gorilla								100	75.1244
Mus musculus									100

**Table 3:** The parameter Alignment Score of the HELIOS method in the quantitative measurement of homology. To calculate the Alignment Score of two compared sequences aligned by the HELIOS method, the BLOSUM62 substitution scoring matrix is adopted with gap opening and extension penalties equal to -10 and -0.5, respectively. Moreover, the *“Nine ND5 protein sequences dataset”* [53] is assumed in this study.

	Homo sapiens	Rattus norvegicus	Balaenoptera physalus	Balaenoptera musculus	Didelphis virginiana	Pan troglodytes	Pan paniscus	Gorilla gorilla	Mus musculus
Homo sapiens	3091	1831.5	1938.5	2003.5	1846	2884	2889	2781	1903.5
Rattus norvegicus		3139	1988	2038	1757	1796.5	1823.5	1768	2396
Balaenoptera physalus			3125	3020	1930	2034	2022.5	1992	1948.5
Balaenoptera musculus				3132	1921	2005	2037.5	1997.5	2005
Didelphis virginiana					3100	1825.5	1920	1784	1865.5
Pan troglodytes						3085	2942	2810	1874
Pan paniscus							3088	2804	1894
Gorilla gorilla								3086	1915.5
Mus musculus									3115

For a comparative study, the quantitative measurement of homology is performed by the HELIOS method and is compared to various well-known algorithms, including BLAST [7], ClustalW [8], ClustalΩ [9], MUSCLE [12], MAFFT [13], Kalign [11], T-Coffee [10], Smith-Waterman (SW) [5], and Needleman-Wunsch (NW) [6] algorithms. As an instance, the values of Identity, Similarity, and Alignment Score of the Smith-Waterman algorithm are reported in detail in Tables 4–6, respectively, to be compared to those of the HELIOS method. Additionally, we consider twelve different datasets for this evaluation, represented in S1 Text–S12 Text; while the input sequences of each dataset are represented in Table A3 in its corresponding file. Moreover, the quantitative measurement of homology of all aforementioned algorithms are reported in Tables A4-A33 in the S1 Text–S12 Text for twelve different datasets [53, 55–61]. By the way, as a brief report, the average value of each parameter, achieved by the aforementioned algorithms, are reported in Table 7 for the twelve datasets.

**Table 4:** The parameter Identity of the Smith-Waterman algorithm in the quantitative measurement of homology. The Identity reports the number of exactly matched characters of two compared sequences (in percentage) aligned by the HELIOS method, assuming the *“Nine ND5 protein sequences dataset”* [53].

	Homo sapiens	Rattus norvegicus	Balaenoptera physalus	Balaenoptera musculus	Didelphis virginiana	Pan troglodytes	Pan paniscus	Gorilla gorilla	Mus musculus
Homo sapiens	100	63.6816	68.4909	68.325	62.7907	93.3665	93.2007	90.2156	64.6766
Rattus norvegicus		100	66.3366	66.5017	60.9635	63.1841	63.1841	62.8524	78.2537
Balaenoptera physalus			100	96.5347	61.6279	68.6567	68.8226	67.33	65.5116
Balaenoptera musculus				100	61.794	68.6567	68.8226	67.4959	65.5116
Didelphis virginiana					100	62.4585	62.7907	60.9635	63.4551
Pan troglodytes						100	95.0249	90.7131	64.6766
Pan paniscus							100	90.8789	64.3449
Gorilla gorilla								100	64.0133
Mus musculus									100

**Table 5:** The parameter Similarity of the Smith-Waterman algorithm in the quantitative measurement of homology. The Similarity measures the resemblance of two compared sequences (in percentage) aligned by the HELIOS method. Various amino acids are categorized into six groups based on their physicochemical properties; including GAVLI, FYW, STCM, KRH, DENQ, and P. Moreover, the *“Nine ND5 protein sequences dataset”* [53] is assumed in this study.

	Homo sapiens	Rattus norvegicus	Balaenoptera physalus	Balaenoptera musculus	Didelphis virginiana	Pan troglodytes	Pan paniscus	Gorilla gorilla	Mus musculus
Homo sapiens	100	72.9685	77.7778	77.2803	74.0864	95.1907	95.8541	92.3715	75.1244
Rattus norvegicus		100	78.2178	77.3927	71.0963	73.1343	72.6368	74.461	85.0082
Balaenoptera physalus			100	97.0297	76.412	77.6119	77.7778	76.7828	77.7228
Balaenoptera musculus				100	75.9136	76.9486	77.1144	76.1194	77.5578
Didelphis virginiana					100	74.0864	74.5847	72.7575	73.0897
Pan troglodytes						100	96.5174	93.2007	75.4561
Pan paniscus							100	93.864	74.2952
Gorilla gorilla								100	75.6219
Mus musculus									100

**Table 6:** The parameter Alignment Score of the Smith-Waterman algorithm in the quantitative measurement of homology. To calculate the Alignment Score of two compared sequences aligned by the HELIOS method, the BLOSUM62 substitution scoring matrix is adopted with gap opening and extension penalties equal to -10 and -0.5, respectively. Moreover, the *“Nine ND5 protein sequences dataset”* [53] is assumed in this study.

	Homo sapiens	Rattus norvegicus	Balaenoptera physalus	Balaenoptera musculus	Didelphis virginiana	Pan troglodytes	Pan paniscus	Gorilla gorilla	Mus musculus
Homo sapiens	3091	1913	2157	2152	1940	2884	2889	2781	1951
Rattus norvegicus		3139	2066	2076	1870	1911	1925	1901	2474
Balaenoptera physalus			3125	3020	1963	2180	2185	2131	2059.5
Balaenoptera musculus				3132	1962.5	2171	2179	2128	2097
Didelphis virginiana					3100	1915	1949	1894.5	1950
Pan troglodytes						3085	2942	2810	1957.5
Pan paniscus							3088	2818	1984.5
Gorilla gorilla								3086	1967.5
Mus musculus									3115

**Table 7:** A brief report of the quantitative measurement of homology of measurement of the HELIOS method, compared to nine well-known algorithms, including SW, NW, BLAST, ClustalW, ClustalΩ, Muscle, T-Coffee, Kalign, and MAFFT. In this manner, the parameters Identity, Similarity, and Alignment Score are reported. The Identity reports the number of exactly matched characters of two compared sequences (in percentage), aligned by each algorithm. Moreover, the Similarity measures the resemblance of two compared sequences (in percentage), aligned by every aformentioned algorithm. Various amino acids are categorized into six groups based on their physicochemical properties; including GAVLI, FYW, STCM, KRH, DENQ, and P. Finally, to calculate the Alignment Score of two compared sequences, aligned by every aforementioned algorithm, the BLOSUM62 substitution scoring matrix is adopted with gap opening and extension penalties equal to -10 and -0.5, respectively. Twelve diferent datasets are considered for this study [53, 55–61].

Parameter	Method	Dataset											
		1^1^	2^2^	3^3^	4^4^	5^5^	6^6^	7^7^	8^8^	9^9^	10^10^	11^11^	12^12^
**The average of Identity (percentage)**	HELIOS	76.959	80.358	68.700	96.982	100	99.815	96.778	99.841	98.638	100	60.091	100
	SW	76.580	80.283	68.862	96.585	100	94.390	96.566	97.874	98.401	100	57.542	100
	NW	76.580	80.283	68.862	96.493	100	94.107	96.438	97.874	98.317	100	57.221	100
	BLAST	76.274	80.283	68.004	97.016	100	99.013	97.019	99.796	98.616	100	59.907	100
	MUSCLE	76.292	80.283	68.104	97.026	100	99.767	96.396	99.841	98.568	100	59.627	100
	ClustalW	76.292	80.283	68.021	97.110	100	99.744	96.484	99.841	98.532	100	56.930	100
	ClustalΩ	76.263	80.283	67.923	96.677	100	99.673	96.353	99.841	98.401	100	57.537	100
	T-Coffee	76.344	80.878	68.206	97.105	100	99.767	96.619	99.841	98.616	100	58.120	100
	Kalign	76.337	80.283	68.104	96.784	100	99.720	96.439	99.841	98.449	100	59.507	100
	MAFFT	76.388	80.283	68.104	97.357	100	99.767	96.916	99.841	98.616	100	60.746	100
**The average of Similarity (percentage)**	HELIOS	83.365	88.591	79.808	97.520	100	99.862	97.305	99.841	98.764	100	65.998	100
	SW	83.623	88.794	81.581	96.995	100	95.711	96.958	98.281	98.574	100	65.924	100
	NW	83.605	88.794	81.531	96.575	100	94.484	96.608	97.919	98.317	100	61.076	100
	BLAST	83.358	88.848	81.174	97.561	100	99.390	97.368	99.796	98.915	100	64.187	100
	MUSCLE	83.325	88.848	81.191	97.704	100	99.862	97.181	99.841	98.909	100	65.922	100
	ClustalW	83.295	88.848	81.108	97.572	100	99.838	97.134	99.841	98.742	100	62.916	100
	ClustalΩ	83.306	88.848	80.878	97.044	100	99.767	96.869	99.841	98.574	100	62.075	100
	T-Coffee	83.358	89.622	81.035	97.455	100	99.862	96.883	99.841	98.873	100	62.805	100
	Kalign	83.373	88.848	81.191	97.281	100	99.815	96.913	99.841	98.622	100	63.478	100
	MAFFT	83.424	88.848	81.386	97.880	100	99.862	97.401	99.841	98.957	100	66.591	100
**The average of Alignment Score**	HELIOS	2314.5	583.4	567.7	2155.3	2011	2274.0	750.5	3865.0	2900.3	5457.9	217.9	2776.7
	SW	2380.3	588.1	600.5	2146.9	2011	2114.1	748.6	3780.5	2897.2	5454.7	220.7	2764.0
	NW	2389.9	595.1	609.5	2190.4	2011	2153.3	759.9	3799.5	2929.8	5454.7	280.8	2764.0
	BLAST	2373.5	588.7	599.4	2155.9	2011	2247.9	747.9	3859.1	2906.1	5457.9	232.8	2776.7
	MUSCLE	2381.3	588.7	604.9	2156.9	2011	2275.3	746.7	3865.0	2904.1	5457.9	232.9	2776.7
	ClustalW	2381.0	588.7	603.5	2154.3	2011	2273.8	744.0	3865.0	2898.8	5457.9	206.8	2776.2
	ClustalΩ	2380.3	588.7	600.7	2146.8	2011	2274.1	745.7	3865.0	2897.2	5457.9	215.9	2776.7
	T-Coffee	2380.6	588.7	602.5	2157.1	2011	2275.3	746.2	3865.0	2903.8	5457.9	214.7	2776.7
	Kalign	2382.3	588.7	604.9	2150.9	2011	2273.2	746.2	3865.0	2900.1	5457.9	231.4	2776.7
	MAFFT	2383.6	588.7	605.5	2162.6	2011	2275.3	747.7	3865.0	2907.3	5457.9	238.7	2776.7

^1^ Dataset 1: *“Nine ND5 protein sequences dataset”* [53]. See S1 Text for the detailed results.

^2^ Dataset 2: *Nine beta globin protein sequences dataset* [53]. See S2 Text for the detailed results.

^3^ Dataset 3: *ND6 (NADH dehydrogenase subunit 6) proteins for eight different species* [55]. See S3 Text for the detailed results.

^4^ Dataset 4: *ENCODE Transcription Factor Targets Dataset, TFAP2A Gene Set* [56]. See S4 Text for the detailed results.

^5^ Dataset 5: *Hub Proteins Protein-Protein Interactions Dataset, AARR1 Gene Set* [57]. See S5 Text for the detailed results.

^6^ Dataset 6: *ChIP-X Enrichment Analysis Resource, CHEA Transcription Factor Targets Dataset, AP1M2 Gene set* [58]. See S6 Text for the detailed results.

^7^ Dataset 7: *ChIP-X Enrichment Analysis Resource, CHEA Transcription Factor Targets Dataset, AP1S2 Gene Set* [58]. See S7 Text for the detailed results.

^8^ Dataset 8: *Encyclopedia of DNA Elements Resource, ENCODE Transcription Factor Targets Dataset, SP1 Gene Set* [59]. See S8 Text for the detailed results.

^9^ Dataset 9: *Hub Proteins Resource, Protein-Protein Interactions Dataset, PTPN6 Gene Set* [57]. See S9 Text for the detailed results.

^10^ Dataset 10: *Kinase Enrichment Analysis Resource, KEA Substrates of Kinases Dataset, ULK Gene Set* [61]. See S10 Text for the detailed results.

^11^ Dataset 11: *Jaspar PWMs Resource, JASPAR Predicted Transcription Factor Targets Dataset, MAX Gene Set* [60]. See S11 Text for the detailed results.

^12^ Dataset 12: *Kinase Enrichment Analysis Resource, KEA Substrates of Kinases Dataset, YES1 Gene* Set [61]. See S12 Text for the detailed results.

Analyzing all data reported in Tables 1, 4, and 7, we can confirm that the HELIOS method detects and locates a bit more identical characters among the input sequences in most of the given datasets, and hence, it leads to higher values of the Identity compared to the SW and other well-known algorithms (as reported in S1 Text–S12 Text). The main reason can be stated as follows; since the HELIOS method inserts limited consecutive gaps freely (R gaps in maximum), it detects and locates more identical characters between two input sequences. Moreover, comparing Table 2 with 5 and considering Table 7, we can conclude that while the Similarity values, achieved by the HELIOS method are approximately equal to those of alternative algorithms for the most given datasets, while at the worst case it is small by only 2.28%, compared to T-Coffee for ND6 (NADH dehydrogenase subunit 6) protein of eight species dataset [55]. On the other hand, as reported in Tables 3, 6, and 7, the values of the Alignment Scores, calculated by the HELIOS method highly reach those of alternative algorithms in our case studies. Therefore, we can conclude that the HELIOS method performs a comparable accurate alignment against the alternative algorithms.

#### Accuracy measurement of classification output

The accuracy measurement of the classification output [52] of the HELIOS method is addressed by calculating the values of Sensitivity (SEN), Specificity (Spec), Accuracy (ACC), Positive Predictive Value (PPV), Negative Predictive Value (NPV), Matthew’s Coefficient Correlation (MCC), and Test’s Accuracy (F-Score) in the simulation studies, according to Eq 5 to Eq 11, respectively.

$$\begin{array}{c} SEN=\frac{TP}{TP+TN} \end{array}$$ (5)

$$\begin{array}{c} Spec=\frac{TN}{TN+FP} \end{array}$$ (6)

$$\begin{array}{c} ACC=\frac{TP+TN}{TP+TN+FP+FN} \end{array}$$ (7)

$$\begin{array}{c} PPV=\frac{TP}{TP+FP} \end{array}$$ (8)

$$\begin{array}{c} NPV=\frac{TN}{TN+FN} \end{array}$$ (9)

$$\begin{array}{c} MCC=\frac{TP\times TN-FP\times FN}{\sqrt{\left(TP+FP)(TP+FN)(TN+FP)(TN+FN\right)}} \end{array}$$ (10)

$$\begin{array}{c} FScore=\frac{2\times TP}{2\times TP+FP+FN} \end{array}$$ (11)

where, parameters TP, TN, FP, and FN stand for True Positive, True Negative, False Positive, and False Negative, respectively.

As a comparative study, the accuracy measurement of the classification output of the HELIOS method is accomplished, and the corresponding metrics (as formulated by Eqs 5 to 11) are calculated with considering Smith-Waterman [5], Needleman-Wunsch [6], ClustalW [8], ClustalΩ [9], BLAST [7], MUSCLE [12], T-Coffee [10], Kalign [11], and MAFFT [13] algorithms as the reference algorithms. As an instance, the values of SEN, Spec, ACC, PPV, NPV, MCC, and F-Score of the HELIOS method are reported with considering SW as the reference algorithm in Tables 8–14, respectively. It should be noted that the values of these metrics for other mentioned algorithms which are considered as the reference algorithm for various datasets are reported in Tables A34-A96 in S1 Text–S12 Text, respectively. By the way, as a brief report, the average value of each parameter considering the mentioned reference algorithms is reported in Table 15 for the same twelve datasets addressed in Table 7.

**Table 8:** The parameter Sensitivity (SEN) of the HELIOS method with referencing the Smith-Waterman algorithm in the accuracy measurement of classification output. The assumed dataset is the *“Nine ND5 protein sequences dataset”* [53] in this study.

	Homo sapiens	Rattus norvegicus	Balaenoptera physalus	Balaenoptera musculus	Didelphis virginiana	Pan troglodytes	Pan paniscus	Gorilla gorilla	Mus musculus
Homo sapiens	1	0.93012	0.88372	0.92359	0.91457	1	1	1	0.94324
Rattus norvegicus		1	0.93377	0.96026	0.86957	0.9103	0.92206	0.89535	0.9654
Balaenoptera physalus			1	1	0.89244	0.9204	0.90713	0.9204	0.90381
Balaenoptera musculus				1	0.88926	0.92703	0.93201	0.92869	0.95207
Didelphis virginiana					1	0.90285	0.95477	0.93467	0.93121
Pan troglodytes						1	1	1	0.91833
Pan paniscus							1	0.99337	0.93023
Gorilla gorilla								1	0.94676
Mus musculus									1

**Table 9:** The parameter Specification (Spec) of the HELIOS method with referencing the Smith-Waterman algorithm in the accuracy measurement of classification output. The assumed dataset is the *“Nine ND5 protein sequences dataset”* [53] in this study.

	Homo sapiens	Rattus norvegicus	Balaenoptera physalus	Balaenoptera musculus	Didelphis virginiana	Pan troglodytes	Pan paniscus	Gorilla gorilla	Mus musculus
Homo sapiens	1	0.99989	0.99987	0.99991	0.99988	1	1	1	0.99992
Rattus norvegicus		1	0.99991	0.99994	0.99981	0.9999	0.9999	0.99987	0.99996
Balaenoptera physalus			1	1	0.99983	0.99991	0.9999	0.99992	0.99986
Balaenoptera musculus				1	0.99983	0.99993	0.99993	0.99993	0.99995
Didelphis virginiana					1	0.99987	0.99993	0.99991	0.99992
Pan troglodytes						1	1	1	0.99989
Pan paniscus							1	0.99999	0.99992
Gorilla gorilla								1	0.99993
Mus musculus									1

**Table 10:** The parameter Accuracy (Acc) of the HELIOS method with referencing the Smith-Waterman algorithm in the accuracy measurement of classification output. The assumed dataset is the *“Nine ND5 protein sequences dataset”* [53] in this study.

	Homo sapiens	Rattus norvegicus	Balaenoptera physalus	Balaenoptera musculus	Didelphis virginiana	Pan troglodytes	Pan paniscus	Gorilla gorilla	Mus musculus
Homo sapiens	1	0.99978	0.99968	0.99979	0.99974	1	1	1	0.99983
Rattus norvegicus		1	0.9998	0.99988	0.9996	0.99975	0.99977	0.9997	0.99991
Balaenoptera physalus			1	1	0.99965	0.99978	0.99975	0.99978	0.99971
Balaenoptera musculus				1	0.99965	0.99981	0.99982	0.99981	0.99987
Didelphis virginiana					1	0.99971	0.99986	0.99981	0.99981
Pan troglodytes						1	1	1	0.99976
Pan paniscus							1	0.99998	0.9998
Gorilla gorilla								1	0.99984
Mus musculus									1

**Table 11:** The parameter Positive Predictive Value (PPV) of the HELIOS method with referencing the Smith-Waterman algorithm in the accuracy measurement of classification output. The assumed dataset is the *“Nine ND5 protein sequences dataset”* [53] in this study.

	Homo sapiens	Rattus norvegicus	Balaenoptera physalus	Balaenoptera musculus	Didelphis virginiana	Pan troglodytes	Pan paniscus	Gorilla gorilla	Mus musculus
Homo sapiens	1	0.93478	0.91883	0.94558	0.92386	1	1	1	0.95118
Rattus norvegicus		1	0.94472	0.96506	0.88435	0.93675	0.93919	0.9198	0.9783
Balaenoptera physalus			1	1	0.89545	0.94549	0.93825	0.9471	0.91597
Balaenoptera musculus				1	0.89527	0.95556	0.95904	0.954	0.96807
Didelphis virginiana					1	0.91823	0.9596	0.94737	0.94872
Pan troglodytes						1	1	1	0.93232
Pan paniscus							1	0.99667	0.94755
Gorilla gorilla								1	0.95791
Mus musculus									1

**Table 12:** The parameter Negative Predictive Value (NPV) of the HELIOS method with referencing the Smith-Waterman algorithm in the accuracy measurement of classification output. The assumed dataset is the *“Nine ND5 protein sequences dataset”* [53] in this study.

	Homo sapiens	Rattus norvegicus	Balaenoptera physalus	Balaenoptera musculus	Didelphis virginiana	Pan troglodytes	Pan paniscus	Gorilla gorilla	Mus musculus
Homo sapiens	1	0.99989	0.99981	0.99987	0.99986	1	1	1	0.99991
Rattus norvegicus		1	0.99989	0.99993	0.99979	0.99985	0.99987	0.99983	0.99994
Balaenoptera physalus			1	1	0.99982	0.99987	0.99985	0.99987	0.99984
Balaenoptera musculus				1	0.99982	0.99988	0.99989	0.99988	0.99992
Didelphis virginiana					1	0.99984	0.99993	0.99989	0.99989
Pan troglodytes						1	1	1	0.99987
Pan paniscus							1	0.99999	0.99989
Gorilla gorilla								1	0.99991
Mus musculus									1

**Table 13:** The parameter Matthew’s Coefficient Correlation (MCC) of the HELIOS method with referencing the Smith-Waterman algorithm in the accuracy measurement of classification output. The assumed dataset is the *“Nine ND5 protein sequences dataset”* [53] in this study.

	Homo sapiens	Rattus norvegicus	Balaenoptera physalus	Balaenoptera musculus	Didelphis virginiana	Pan troglodytes	Pan paniscus	Gorilla gorilla	Mus musculus
Homo sapiens	1	0.93234	0.90094	0.93441	0.91907	1	1	1	0.94711
Rattus norvegicus		1	0.93913	0.9626	0.87673	0.92331	0.93047	0.90734	0.97178
Balaenoptera physalus			1	1	0.89377	0.93275	0.92243	0.93355	0.90972
Balaenoptera musculus				1	0.89209	0.94109	0.94534	0.94117	0.95997
Didelphis virginiana					1	0.91036	0.95711	0.9409	0.93983
Pan troglodytes						1	1	1	0.92518
Pan paniscus							1	0.99501	0.93875
Gorilla gorilla								1	0.95224
Mus musculus									1

**Table 14:** The parameter Test’s Accuracy (F-Score) of the HELIOS method with referencing the Smith-Waterman algorithm in the accuracy measurement of classification output. The assumed dataset is the *“Nine ND5 protein sequences dataset”* [53] in this study.

	Homo sapiens	Rattus norvegicus	Balaenoptera physalus	Balaenoptera musculus	Didelphis virginiana	Pan troglodytes	Pan paniscus	Gorilla gorilla	Mus musculus
Homo sapiens	1	0.93244	0.90093	0.93445	0.91919	1	1	1	0.94719
Rattus norvegicus		1	0.93922	0.96266	0.8769	0.92334	0.93054	0.90741	0.97181
Balaenoptera physalus			1	1	0.89394	0.93277	0.92243	0.93356	0.90985
Balaenoptera musculus				1	0.89226	0.94108	0.94533	0.94118	0.96
Didelphis virginiana					1	0.91047	0.95718	0.94098	0.93988
Pan troglodytes						1	1	1	0.92527
Pan paniscus							1	0.99502	0.93881
Gorilla gorilla								1	0.9523
Mus musculus									1

**Table 15:** A brief report of the accuracy measurement of classification output of the HELIOS method with referencing well-known algorithms, including Smith-Waterman, Needleman-Wunsch, ClustalW, ClustalΩ, BLAST, Muscle T-Coffee, Kalign, MAFFT algorithms. The parameters SEN, Spec, ACC, PPV, NPV, MCC, and F-Score are averaged and reported. The twelve datasets [53, 55–61] are considered.

Parameter	Method	Dataset											
		1	2	3	4	5	6	7	8	9	10	11	12
**The average of the parameter SEN**	SW	0.94972	0.98565	0.87985	0.96481	1.00000	0.93868	0.98375	0.97558	0.98897	0.99841	0.59860	0.99666
	NW	0.79205	0.98565	0.61425	0.31215	1.00000	0.60910	0.99298	0.50587	0.47676	0.99841	0.57446	0.99666
	BLAST	0.95190	0.98914	0.87743	0.96788	1.00000	0.98681	0.95240	0.99773	0.99182	1.00000	0.59893	0.99933
	MUSCLE	0.95424	0.98675	0.88059	0.96513	1.00000	0.99862	0.97137	0.99955	0.98603	0.99873	0.57025	0.99933
	ClustalW	0.95491	0.98675	0.87992	0.96535	1.00000	0.99905	0.97238	1.00000	0.99086	0.99968	0.56972	0.99967
	ClustalΩ	0.95476	0.98822	0.88149	0.96481	1.00000	0.99811	0.97815	0.99955	0.98897	0.99952	0.58862	0.99933
	T-Coffee	0.95557	0.71116	0.42407	0.97446	1.00000	0.99953	0.96569	0.99955	0.98863	0.99936	0.58200	1.00000
	Kalign	0.95561	0.98914	0.88059	0.96661	1.00000	0.99862	0.97769	1.00000	0.98945	0.99936	0.58769	0.99933
	MAFFT	0.95613	0.98675	0.87897	0.96807	1.00000	0.99953	0.98137	0.99955	0.98643	0.99873	0.60765	0.99933
**The average of the parameter Spec**	SW	0.99994	0.99994	0.99940	0.99993	1.00000	0.99987	0.99991	0.99997	0.99998	1.00000	0.99689	0.99999
	NW	0.99968	0.99993	0.99786	0.99854	1.00000	0.99920	0.99997	0.99936	0.99913	1.00000	0.99392	0.99999
	BLAST	0.99994	0.99996	0.99938	0.99993	1.00000	0.99997	0.99984	1.00000	0.99999	1.00000	0.99684	1.00000
	MUSCLE	0.99995	0.99995	0.99941	0.99993	1.00000	1.00000	0.99982	1.00000	0.99998	1.00000	0.99667	1.00000
	ClustalW	0.99995	0.99995	0.99940	0.99993	1.00000	1.00000	0.99982	1.00000	0.99999	1.00000	0.99678	1.00000
	ClustalΩ	0.99995	0.99996	0.99941	0.99993	1.00000	1.00000	0.99985	1.00000	0.99998	1.00000	0.99675	1.00000
	T-Coffee	0.99995	0.99838	0.99680	0.99995	1.00000	1.00000	0.99992	1.00000	0.99998	1.00000	0.99672	1.00000
	Kalign	0.99995	0.99996	0.99941	0.99993	1.00000	1.00000	0.99985	1.00000	0.99998	1.00000	0.99680	1.00000
	MAFFT	0.9999	0.99995	0.99940	0.99993	1.00000	1.00000	0.99988	1.00000	0.99998	1.00000	0.99690	1.00000
**The average of the parameter Acc**	SW	0.99985	0.99984	0.99872	0.99985	1.00000	0.99975	0.99980	0.99994	0.99997	1.00000	0.99357	0.99999
	NW	0.99933	0.99983	0.99563	0.99683	1.00000	0.99817	0.99993	0.99868	0.99814	1.00000	0.98456	0.99999
	BLAST	0.99986	0.99989	0.99869	0.99986	1.00000	0.99995	0.99954	0.99999	0.99997	1.00000	0.99491	1.00000
	MUSCLE	0.99987	0.99986	0.99873	0.99986	1.00000	0.99999	0.99964	1.00000	0.99995	1.00000	0.99383	1.00000
	ClustalW	0.99987	0.99986	0.99872	0.99985	1.00000	1.00000	0.99965	1.00000	0.99997	1.00000	0.99325	1.00000
	ClustalΩ	0.99987	0.99988	0.99874	0.99985	1.00000	0.99999	0.99972	1.00000	0.99997	1.00000	0.99503	1.00000
	T-Coffee	0.99987	0.99643	0.99354	0.99989	1.00000	1.00000	0.99971	1.00000	0.99996	1.00000	0.99425	1.00000
	Kalign	0.99988	0.99989	0.99873	0.99986	1.00000	0.99999	0.99971	1.00000	0.99997	1.00000	0.99462	1.00000
	MAFFT	0.99988	0.99986	0.99871	0.99986	1.00000	1.00000	0.99976	1.00000	0.99996	1.00000	0.99444	1.00000
**The average of the parameter PPV**	SW	0.96055	0.99097	0.89123	0.96579	1.00000	0.93628	0.98489	0.97706	0.98989	0.99841	0.60935	0.99666
	NW	0.80181	0.99097	0.62125	0.31743	1.00000	0.61167	0.99603	0.50628	0.47677	0.99841	0.99031	0.99666
	BLAST	0.96273	0.99446	0.88875	0.96785	1.00000	0.98681	0.97368	0.99773	0.99994	1.00000	0.59979	0.99933
	MUSCLE	0.96636	0.99207	0.89298	0.96611	1.00000	0.99862	0.97082	0.99955	0.98496	0.99873	0.57311	0.99933
	ClustalW	0.96704	0.99207	0.89227	0.96579	1.00000	0.99882	0.97176	1.00000	0.99179	0.99968	0.57597	0.99961
	ClustalΩ	0.96689	0.99354	0.89387	0.96579	1.00000	0.99811	0.97671	0.99955	0.98989	0.99952	0.58637	0.99933
	T-Coffee	0.96771	0.74743	0.42829	0.97467	1.00000	0.99953	0.98719	0.99955	0.98754	0.99936	0.56966	1.00000
	Kalign	0.96775	0.99446	0.89298	0.96627	1.00000	0.99815	0.97626	1.00000	0.99038	0.99936	0.59457	0.99933
	MAFFT	0.96828	0.99207	0.89133	0.96760	1.00000	0.99953	0.98137	0.99955	0.98536	0.99873	0.60220	0.99933
**The average of the parameter NPV**	SW	0.99992	0.99990	0.99931	0.99993	1.00000	0.99988	0.99990	0.99997	0.99998	1.00000	0.99663	0.99999
	NW	0.99966	0.99990	0.99775	0.99828	1.00000	0.99897	0.99995	0.99932	0.99900	1.00000	0.99031	0.99999
	BLAST	0.99992	0.99993	0.99930	0.99993	1.00000	0.99997	0.99970	1.00000	0.99999	1.00000	0.99804	1.00000
	MUSCLE	0.99992	0.99991	0.99932	0.99993	1.00000	1.00000	0.99982	1.00000	0.99998	1.00000	0.99712	1.00000
	ClustalW	0.99993	0.99991	0.99931	0.99993	1.00000	1.00000	0.99983	1.00000	0.99998	1.00000	0.99642	1.00000
	ClustalΩ	0.99993	0.99992	0.99932	0.99993	1.00000	1.00000	0.99986	1.00000	0.99998	1.00000	0.99825	1.00000
	T-Coffee	0.99993	0.99803	0.99670	0.99995	1.00000	1.00000	0.99978	1.00000	0.99998	1.00000	0.99749	1.00000
	Kalign	0.99993	0.99993	0.99932	0.99993	1.00000	1.00000	0.99986	1.00000	0.99998	1.00000	0.99778	1.00000
	MAFFT	0.99993	0.99991	0.99931	0.99993	1.00000	1.00000	0.99988	1.00000	0.99998	1.00000	0.99751	1.00000
**The average of the parameter MMC**	SW	0.95503	0.98821	0.88485	0.96522	1.00000	0.93736	0.98422	0.97629	0.98941	0.99841	0.60062	0.99665
	NW	0.79657	0.98820	0.61553	0.31305	1.00000	0.60940	0.99446	0.50541	0.47583	0.99841	0.59308	0.99665
	BLAST	0.95722	0.99172	0.88238	0.96779	1.00000	0.98679	0.96267	0.99772	0.99186	1.00000	0.59684	0.99933
	MUSCLE	0.96020	0.98932	0.88609	0.96555	1.00000	0.99862	0.97091	0.99954	0.98547	0.99873	0.56850	0.99933
	ClustalW	0.96087	0.98932	0.88540	0.96549	1.00000	0.99893	0.97189	1.00000	0.99131	0.99968	0.56933	0.99964
	ClustalΩ	0.96072	0.99080	0.88699	0.96522	1.00000	0.99811	0.97728	0.99954	0.98941	0.99952	0.58520	0.99933
	T-Coffee	0.96155	0.72670	0.42291	0.97451	1.00000	0.99953	0.97615	0.99954	0.98806	0.99936	0.57253	1.00000
	Kalign	0.96159	0.99172	0.88609	0.96637	1.00000	0.99838	0.97682	1.00000	0.98989	0.99936	0.58843	0.99933
	MAFFT	0.96211	0.98932	0.88445	0.96776	1.00000	0.99953	0.98125	0.99954	0.98587	0.99873	0.60190	0.99933
**The average of the parameter F-Score**	SW	0.95508	0.98827	0.88544	0.96529	1.00000	0.93748	0.98432	0.97631	0.98943	0.99841	0.60374	0.99666
	NW	0.79687	0.98827	0.6170	0.31447	1.00000	0.61023	0.99448	0.50607	0.47676	0.99841	0.59697	0.99999
	BLAST	0.95726	0.99176	0.88299	0.96786	1.00000	0.98681	0.96275	0.99773	0.99188	1.00000	0.59915	0.99933
	MUSCLE	0.96023	0.98937	0.88667	0.96562	1.00000	0.99862	0.97108	0.99955	0.98549	0.99873	0.57149	0.99933
	ClustalW	0.96090	0.98937	0.88598	0.96556	1.00000	0.99894	0.97206	1.00000	0.99132	0.99968	0.57261	0.99964
	ClustalΩ	0.96075	0.99084	0.88757	0.96529	1.00000	0.99811	0.97741	0.99955	0.98943	0.99952	0.58742	0.99933
	T-Coffee	0.96157	0.72768	0.42615	0.97456	1.00000	0.99953	0.97615	0.99955	0.98808	0.99936	0.57497	1.00000
	Kalign	0.96161	0.99176	0.88667	0.96644	1.00000	0.99838	0.97695	1.00000	0.98991	0.99936	0.59093	0.99933
	MAFFT	0.9621	0.98937	0.88504	0.96783	1.00000	0.99953	0.98137	0.99955	0.98589	0.99873	0.60443	0.99933

As reported in Tables 8–14 with assuming SW as the reference algorithm, and then reported in Table 15, the values of SEN, Spec, ACC, PPV, NPV, MCC, and F-Score of the HELIOS method approximately reach the value of one for most of the comparative studies. The latter observation confirms that the simulation outputs by the HELIOS method are highly similar to those of SW, BLAST, MUSCLE, ClustalW, ClustalΩ, Kalign, and MAFFT; while there are some differences with the outputs of NW and T-Coffee. It is noteworthy that detection of more identical characters by the HELIOS method, and hence, the higher value of the Identity, as represented in Tables 1 and 7, results in the less value of the aforementioned parameters, as presented in Tables 8–15.

### Performance evaluation

For performance evaluation, at first, we analyze the time and space complexities of the HELIOS optical architecture by analytical computations, and afterward, the processing time and required memory of the HELIOS optical architecture is analytically estimated. Both analyses are compared to the well-known algorithms with various implementation assumptions.

First of all, it is worth noting that the ability to arrange numerous input sequences on the 2D aperture of the employed modulators empowers the HELIOS optical architecture to simultaneously align numerous sequences through each alignment process, as the result of the highly sophisticated HELIOS optical architecture along with the inherent parallel processing capability of optics. Moreover, relying on parallelism, it eliminates the need for large storage components, such as RAMs. Subsequently, its required memory only depends on the aperture size of the employed modulators. By the way, we consider the utilized space on the modulators to modulate the whole input sequences as the space complexity of the HELIOS optical architecture. Here, the aperture size of modulators directly impacts the time and space complexities. Specifically, they can be large enough to modulate a whole genome, or thousands of short sequences to be aligned concurrently. Moreover, regarding negligible light propagation delay through an optical architecture, the time complexity and processing time of numerous pairwise sequences alignments are also determined by the switching time of the modulators. Therefore, taking advantage of the HELIOS optical architecture, the input sequences are aligned at the speed of light, regardless of the number of sequence shifts (i.e. R) in the alignment procedure. In all, while these two key factors, i.e. the aperture size and switching times of the modulators, determine the time complexity and processing time of the HELIOS optical architecture; the utilized space on the modulators is considered as the space complexity of HELIOS optical architecture. In folowing case of studies, we consider two modulators of size *L* × *W* pixels, as L represents its length and W stands for its width, and assume the first and the second input sequences of lengths *N* and *M*, respectively, while *N* > *M*.

#### Time and space complexities

As the HELIOS method employs a simple coding scheme to align two sequences, which codes every character of input sequence by only two entries, it achieves the time complexity in order of $O(\frac{2NM}{L^{2}W})$ and space complexity in order of $O(\frac{2NM}{L}+N+M)$. However, employing large modulators for aligning short sequences leads to *O*(1) time complexity. To emphasize the superiority of optical computing, it should be noted that common electrical algorithms necessitate storage of large matrices, and hence, are inefficient for aligning a substantial number of long sequences. For instance, it reaches *O*(*MN*) for SW, NW, ClustalW, MUSCLE, and T-Coffee [21, 22]. As shown in Table 16, the time and space complexities of the HELIOS optical architecture are considerably less than those of alternative electrical algorithms.

**Table 16:** Time and space complexities of the HELIOS compared to well-known algorithms, performing a pairwise sequence alignment. In this table, N and M are the lengths of the first and the second input sequences, respectively, assuming N > M. Moreover, the parameter L × W is the size of the aperture of modulators, used in HELIOS optical architecture. As represented in this table, HELIOS offers a fraction of MN by L2 × W for time complexity, leading to O(1) in the case of large modulators.

Method	Time Complexity		Space Complexity	
	Detail	Overall	Detail	Overall
HELIOS Optical Architecture.	O(2NM/(*L*^2^ *W*))	O(1)	O(2NM/L)+O(N+M)	O((MN)
Smith-Waterman	O(2MN)+O(M+N)+O(N)	O(MN)	O(MN)+O(N)	O(MN)
Needleman-Wunsch	O(MN)+O(2(M+N))+O(N)	O(MN)	O(MN)+O(N)	O(MN)
ClustalW	O(MN)+O(M+N)	O(MN)	O(4MN)+O(2(M+N))	O(MN)
T-Coffee	O(MN)+O(M+N)	O(MN)	O(4MN)+O(2(M+N))	O(MN)
MAFFT	O(4MN)	O(MN)	O(MN)+O(N+M)	O(MN)
Moiré Technique	O(16NM/(*L*^2^))	O(MN)	O(16NM ×(*W*^2^))	O(MN)
HAWPOD	O(4NM/(*L*^2^))	O(MN)	O(4NM ×(*W*^2^))	O(MN)
OptCAM	O(16NM/(*L*^2^ *W*))	O(1)	O(32NM/L)+O(4(N+M))	O(MN)

On the other hand, a few recent proposed optical approaches, such as Moiré Technique [31], HAWPOD [30], and OptCAM [34] benefit from the parallelism and high-speed processing of optics. However, due to their exclusive coding assumptions, they can be adopted for neither RNAs nor proteins alignment. Moreover, these methods occupy a large number of pixels to code a DNA character which leads to inefficient resource utilization. While the OptCAM needs 8 pixels to code a single character, the HAWPOD and the Moiré Technique use two and four columns on a modulator (2 × W pixels), respectively. However, it is only two pixels for the HELIOS optical architecture. Moreover, while the output of the Moiré Technique is too noisy and practically useless for large input sequences, the outputs of the HELIOS method and its optical architecture are highly manipulated to be easy to understand. Finally, estimating the order of time complexity of Moiré Technique, HAWPOD, and OptCAM leads to $O(\frac{16NM}{L^{2}})$, $O(\frac{4NM}{L^{2}})$, and $O(\left(\frac{16NM}{L^{2}W}\right)$, respectively. Additionally, the estimations of the order of their space complexities are $O(16NM\times W^{2})$, $O(4NM\times W^{2})$, and $O(\frac{32NM}{L}+4\left(N+M\right))$, for Moiré Technique, HAWPOD, and OptCAM, respectively. According to these estimations, the HELIOS optical architecture outperforms the alternative optical approaches in terms of time and space complexities, alongside its wider applicability.

Considering the electrical implementation, the HELIOS method is simple enough to be executed on a typical computer. Specifically, in the case of time complexity, comparing each character of an input sequence with 2R+1 characters of the other one leads to time complexity in the order of *O*((2*R* + 1)(*M* + *N*)). In this regard, since the variable R is in the range of [1, $\frac{N}{2}+1$], the time complexity varies in the range of [*O*(3(*M* + *N*)), *O*(2*MN* + *N* + *M*)]. Additionally, in the case of space complexity, the required space for the coding and alignment procedures can be estimated as follow:

- *O*(2(*M* + *N*)) for coding procedure,
- *O*((2*R* + 1)(*M* + *N*)) for shifting and aligning two sequences; varying in [*O*(3(*M* + *N*)), *O*(2*MN* + *N* + *M*)] for R in the range of [1, $\frac{N}{2}+1$],
- *O*(*M* + *N*) for compensation of false mismatching and storing the final output.

Therefore, the space complexity of the HELIOS method, executed on an electrical computer is in the range of [*O*(6(*M* + *N*)), *O*(2*MN* + 4(*M* + *N*))]. Concluding the above discussion, we can state that the HELIOS method achieves linear to quadratic time and space complexities, running on an electrical computer.

#### Processing time comparison

For a comprehensive comparison of the processing time and the memory requirement, the following scenarios are considered. In this manner, the processing times and memory of the HELIOS optical architecture are analytically estimated and compared to A) Nucmer4 [14], Mauve [47], LASTZ match [48], LASTZ default [48], Moiré Technique [31], HAWPOD [30], and OptCAM [34] in the case of genome-to-genome alignment, as reported in [14], B) Nucmer4 [14], BLASR [44], BWA-MEM [45], Bowtie2 [46], Moiré Technique [31], HAWPOD [30], and OptCAM [34] in the case of reads alignment with a reference sequence considering Illumina and PacBio databases for Arabidopsis [62] and Human [63] reference genomes, as reported in [14], and finally, C) Smith-waterman [5], in the case of aligning various length of sequences with the SWISS-PROT dataset [64], reported in [65]. The analytical estimation of processing time of HELIOS optical architecture is performed by considering the aperture size and switching times of the modulators, as follows, according to Eq 12; while the memory requirement equals the aperture size of the modulators, according to Eq 13.

$$\begin{array}{c} P=2\times \frac{N\times M}{L^{2}\times W} \end{array}$$ (12)

$$\begin{array}{c} S=L\times W \end{array}$$ (13)

where, *P* stands for the processing time, and *S* represents the required memory to align two input sequences of length N and M. Moreover, L and W are the length and width of the modulators in pixels, respectively, and T stands for the switching time of the modulators.

In this manner, for the first and the second comparison scenarios, all reported timings are measured on a dual-CPU, 32-core AMD Opteron 6276 computer with 256 GB of DDR3 PC3–12800 RAM, using 32 parallel threads. Moreover, detailed information about assumed datasets in both scenarios are shown in Table 17 [14]. Finally, for the third comparison, an NVIDIA TESLA K40 GPU with 44.3 giga cell updates per second (GCUPS), and GTX 275 with 21 GCUPS [65] are employed. On the other hand, for the analytical estimation of the processing times of the HELIOS optical architecture, we consider a typical graphene-based modulator with an aperture size of 1024 × 1024 pixels and a 100 MHz switching rate [66], which can modulate 1,048,576 bases per 10 nanoseconds. It should be noted that the switching rate can be increased up to 4.5 THz, resulting in 0.22 picoseconds for the switching time, by employing a recently developed metamaterial-based modulator [67]. Additionally, the aperture size of the modulator can be enlarged as well, for example to 4096 × 4096 pixels to escalate the number of modulated bases per switch, which speeds up the process by 64 times.

**Table 17:** Detailed description of the employed datasets for the genome-to-genome alignment, and the reads alignment with a reference sequence. In these comparisons, the Illumina and PacBio data for Arabidopsis thaliana Ler-0 genome [68] are employed. Moreover, the human Illumina and PacBio reads are employed from the Ashkenazi child data set, which is available from the Genome in a Bottle project [69], NCBI SRA accession SRX847862. Furthermore, the reference genomes are the Arabidopsis thaliana Col-0 reference genome [62], human reference genome versionGRCH38.p7 [63], and the chimpanzee (pan troglodytes) genome [70], released in PanTro4, GenBank accession GCF0001515.6.

Reference	Genome size	Illumina			PacBio		
		number of reads	bases in reads	average read size	number of reads	bases in reads	average read size
Arabidopsis	120 MB	23 M	6919 M	300 bp	481 K	2748 M	5713 bp
Human	3.09 GB	264 M	39.1 G	300 bp	3.9	30.5 G	7821 bp
Chimp	3.31 GB	-					

A) For the first comparison scenario, the processing time of the HELIOS optical architecture is compared to that of Nucmer4, Mauve, LASTZ default, LASTZ match, Moiré Technique, HAWPOD, and OptCAM in the case of genome-to-genome alignment, as represented in Table 18.

At first, the reference assemblies of human genome, version GRCh38.p7 [63] (3.088 Gb) and chimpanzee genome, (release PanTro4, GenBank accession GCF_000001515.6) [70] (3.31 Gb) are aligned to one another, as represented in Table 17. It takes 3104 minutes with 66 GB memory for Nucmer4, and more than 2 days for Mauve, LASTZ default, and LASTZ match, as represented in Table 18. However, the HELIOS optical architecture performs this alignment in 190.5 seconds with 1 MB memory usage. On the other hand, it takes more than 4 days for Moiré Technique and HAWPOD, and 25.40 minutes for OptCAM, with 1MB memory requirements for all of them, assuming the same modulators.

**Table 18:** Processing time and memory requirement of genome-to-genome alignment for the HELIOS optical architecture, compared to Nucmer4, Mauve, LASTZ default, LASTZ match, OptCAM, HAWPOD, and Moiré Techniques. For these comparison scenarios, all reported timings for Nucmer4, Mauve, LASTZ default, and LASTZ match are measured on a dual-CPU, 32-core AMD Opteron 6276 computer with 256 GB of DDR3 PC3–12800 RAM, using 32 parallel threads. On the other hand, for the analytical estimation of the processing times of the HELIOS optical architecture, OptCAM, HAWPOD, and Moiré Technique, a typical graphene-based modulator is considered with an aperture size of 1024 × 1024 pixels and a 100 MHz switching rate. Moreover, the processing times of the HELIOS optical architecture are reported for more recently developed modulators with various aperture sizes, such as 1920 × 1080 and 4096 × 4096 pixels, as well as various switching rates, including 35 GHz [72] and 4.5 THz [67]. It should be noted that the processing times reported for Nucmer4, Mauve, LASTZ default, and LASTZ match include Wall time and CPU time, while the HELIOS method is implemented within its optical architecture, and hence, the corresponding wall time is assumed zero.

Method	Implementation	Human / Chimp		Arabidopsis		Tardigrade	
		Processing time	Memory	Processing time	Memory	Processing time	Memory
LASTZ default	A dual-CPU, 32-core AMD Opteron 6276 computer with 256 GB of DDR3 PC3–12800 RAM, using 32 parallel threads	>2 days	-	2135 min	1.3 GB	>2 days	-
LASTZ match		>2 days	-	132 min	0.6 GB	153 min	0.4 GB
Mauve		>2 days	-	79.6 min	3.3 GB	541 min	4.0 GB
Nucmer4		3104 min	66 GB	25.7 min	4.6 GB	30 min	4.9 GB
Moiré Technique	A graphene-based modulator with an aperture size of 1024 × 1024 pixels and a 100 MHz switching rate	>4 days	1 MB	63.17 min	1 MB	72.78 min	1 MB
HAWPOD		>4 days	1 MB	15.79 min	1 MB	18.19 min	1 MB
OptCAM		25.40 min	1 MB	3.70 sec	1 MB	4.26 sec	1 MB
HELIOS		190.5 sec	1 MB	0.462 sec	1 MB	0.533 sec	1 MB
	A graphene-based modulator with an aperture size of 4096 × 4096 pixels and a 100 MHz switching rate	2.976 sec	16 MB	7.2e-3 sec	16 MB	8.3e-3 sec	16 MB
	A graphene-based modulator with an aperture size 1920 × 1080 and a 35 GHz switching rate	0.146 sec	2 MB	3.56e-4 sec	2 MB	4.10e-4 sec	2 MB
	A metamaterial-based modulator with an aperture size of 1024 × 1024 pixels and a 4.5 THz switching rate	4.20e-3 sec	1 MB	1.02e-5 sec	1 MB	1.18e-5	1 MB

Secondly. for aligning two Arabidopsis species, the Arabidopsis lyrata assembly 1.0 [71] (207 Mb) is aligned to the Arabidopsis thaliana Col-0 reference genome [62] (120 Mb), as represented in Table 17. This process takes 25.7 minutes with 4.6 GB, 79.6 minutes with 3.3GB, 2135 minutes with 1.3 GB, 132 minutes with 0.6 GB for Nucmer4, Mauve, LASTZ default, and LASTZ match, respectively, as represented in Table 18. While it takes 63.17 minutes, 15.79 minutes, and 3.70 seconds as the processing time and 1 MB memory usage for Moiré Technique, HAWPOD, and OptCAM, respectively. As depicted in these comparisons, it can be clarified how the employed coding assumption can enhance the performance of an optical system, regardless of the high-speed processing and inherent parallelism of optics. Hence, relying on its compact coding scheme, the HELIOS optical architecture outperforms other optical alternatives, specifically in terms of the processing time. Finally, it is performed in 0.4627 seconds with 1MB memory usage by the HELIOS optical architecture.

Finally, aligning two assemblies of a microscopic animal, the tardigrade (Hypsibius dujardini), represented in two different studies [73, 74] is considered. In this manner, the assembly of Hd-Boothby [73] (212 Mb) is aligned with the assembly of Hd-Blaxter [74] (135 Mb). As represented in Table 18, it takes 30 minutes with 4.9 GB, 541 minutes with 4.0 GB, 153 minutes with 0.4 GB, and more than two days for Nucmer4, Mauve, LASTZ match, and LASTZ default, respectively. Moreover, it takes 72.78 minutes, 18.19 minutes, and 4.2647 seconds with 1 MB memory usage for Moiré Technique, HAWPOD, and OptCAM, respectively. Finally, the HELIOS optical architecture aligns them in 0.5331 seconds with 1 MB Memory usage.

It is noteworthy that the processing times reported for Nucmer4, Mauve, LASTZ default, and LASTZ match include Wall time and CPU time. While the HELIOS optical architecture performs the HELIOS method by light beam propagation through photonics components, and hence, it results in zero wall time. Taking advantages of the operational parallelism of optics, the processing time of the HELIOS optical architecture can be considerably reduced by employing larger and faster modulators, such as 1920 × 1080 and 4096 × 4096 pixels for their aperture size, as well as 35 GHz [72] and 4.5 THz [67] for their switching rate, as reported in Table 18.

B) For the second comparison scenario, the processing time of the HELIOS optical architecture is estimated and compared to that of Nucmer4 [14], Bowtie2 [46], BWA-MEM [45], and BLASR [44], as reported in Table 19. For this comparative study, we adopt PacBio SMRT and Illumina reads from Aradopsis thaliana Ler-0 [68] for aligning to the Arabidopsis thaliana Col-0 reference genomes [62]. Moreover, a subset of Illumina and PacBio reads from the publicly available Ashkenazi dataset, which is available from the Genome in a Bottle project [69], NCBI SRA accession SRX847862, is aligned to the human genome reference GRCh38.p7 [63]. Detailed information about these datasets is shown in Table 17 [14]. Finally, it should be noted that in these time comparisons, we reported the runtimes of the building alignment index and aligning process by Nucmer4, Bowtie2, BWA-MEM, and BLASR.

**Table 19:** Processing time and memory usage to align PacBio and Illumina reads to the Arabidopsis and Human reference genomes by HELIOS optical architecture, compared to BLASR, BWA-MEM, Bowtie2, Nucmer4, Moiré Technique, HAWPOD, and OptCAM. In this regard, the analytical estimation of the processing times of the HELIOS optical architecture, OptCAM, HAWPOD, and Moiré Technique, are reported by employing a typical graphene-based modulator with an aperture size of 1024 × 1024 pixels and a 100 MHz switching rate. In versus, the processing times of Nucmer4, Bowtie2, BWA-MEM, and BLASR are measured on a dual-CPU, 32-core AMD Opteron 6276 computer with 256 GB of DDR3 PC3–12800 RAM, using 32 parallel threads, as reported in [14]. The report includes the times to build the genome index and to align the sequences. Moreover, the processing times of HELIOS optical architecture are also reported for more recently developed modulators with various aperture sizes, such as 1920 × 1080 and 4096 × 4096 pixels, as well as various switching rates, including 35 GHz [72] and 4.5 THz [67].

Method	Implementation	PacBio				Illumina			
		Arabidopsis		Human		Arabidopsis		Human	
		Time	Memory	Time	Memory	Time	Memory	Time	Memory
BLASR	A dual-CPU, 32-core AMD Opteron 6276 computer with 256 GB of DDR3 PC3–12800 RAM, using 32 parallel threads	95 min	4065 MB	1720 min	77.3 GB	-	-	-	-
BWA-MEM		49 min	2162 MB	1569 min	12.2 GB	30 min	3360 MB	293 min	15.7 GB
Bowtie2		-	-	-	-	24 min	686 MB	214 min	22.6 GB
Nucmer4		24 min	95.2 GB	886 min	95.2 GB	29 min	1283 MB	182 min	90.6 GB
Moiré	A graphene-based modulator with an aperture size of 1024 × 1024 pixels and a 100 MHz switching rate	838 min	1 MB	2.39e5 min	1 MB	2.11e3 min	1 MB	3.07e5 min	1 MB
HAWPOD		209 min	1 MB	5.99e4 min	1 MB	528 min	1 MB	7.68e4 min	1 MB
OptCAM		49.13 sec	1 MB	234 min	1 MB	123.7 sec	1 MB	300 min	1 MB
HELIOS		6.14 sec	1 MB	29.25 min	1 MB	15.46 sec	1 MB	37.5 min	1 MB
	A graphene-based modulator with an aperture size of 4096 × 4096 pixels and a 100 MHz switching rate	9.6e-2 sec	16 MB	27.42 sec	16 MB	0.2416 sec	16 MB	35.16 sec	16 MB
	A graphene-based modulator with an aperture size 1920 × 1080 and a 35 GHz switching rate	4.70e-3 sec	2 MB	1.35 sec	2 MB	1.19e-2 sec	2 MB	1.73 sec	2 MB
	A metamaterial-based modulator with an aperture size of 1024 × 1024 pixels and a 4.5 THz switching rate	1.36e-4 sec	1 MB	3.90e-2 sec	1 MB	3.43e-4	1 MB	0.05 sec	1 MB

In order to align 481,000 PacBio reads (2748 Mbp) to the Arabidopsis reference genome (120 Mbp), reported in Table 17, the HELIOS optical architecture needs 6.1423 seconds with 1 MB memory, compared to 95 minutes, 49 minutes, and 24 minutes for BLASR, BWA-MEM, and Nucmer4, respectively, as reported in Table 19. On the other hand, the required times to perform this alignment are 838.5 minutes, 209 minutes, 49.1 seconds for Moiré Technique, HAWPOD, and OptCAM, respectively. Finally, the HELIOS optical architecture only needs 1MB memory usage, similar to Moiré Technique, HAWPOD, and OptCAM; while BLASR, BWA-MEM, and Nucmer4 require 4065 MB, 2162 MB, and 5743 MB memory, respectively.

Since the Arabidopsis genome is a small reference genome (120 Mbp), we consider aligning 3.9M PacBio reads (30.5 Gbp) with the Human reference genome (3.09 Gbp) as well, as shown in Table 17. As represented in Table 19, the HELIOS optical architecture achieves 29.25 minutes as the processing time, compared to 1720 minutes, 1569 minutes, 886 minutes, 239680 minutes, 59919 minutes, 234.06 minutes for BLASR, BWA-MEM, Nucmer4, Moiré Technique, HAWPOD, and OptCAM, respectively. Moreover, BLASR, BWA-MEM, and Nucmer4 require 77.3 GB, 12.2 GB, and 95.2 GB memory, respectively, against 1MB memory usage of Moiré Technique, HAWPOD, OptCAM, and HELIOS optical architecture.

As a similar comparative study, the processing times and memory requirement are calculated for the Illumina reads with Arabidopsis and Human reference genomes. For aligning 23 million Illumina reads (6919 Mbp) with Arabidopsis reference genome (120 Mbp), the HELIOS optical architecture requires 15.46 seconds, compared to 30 minutes, 24 minutes, 29 minutes, 2111.5 minutes, 527.87 minutes, and 123.72 seconds for BWA-MEM, Bowtie2, Nucmer4, Moiré Technique, HAWPOD, and OptCAM, respectively. Moreover, BWA-MEM, Bowtie2, and Nucmer4 require 3360 MB, 686 MB, and 1283 MB of memory, respectively, against 1MB for the mentioned optical approaches. On the other hand, to align 264 million Illumina reads (39.1 Gbp) with the Human reference genome (3.09 Gbp), the HELIOS requires 37.50 minutes, compared to 293 minutes, 214 minutes, 182 minutes, 307260 minutes, 76815 minutes, and 300 minutes for BWA-MEM, Bowtie2, Nucmer4, Moiré Technique, HAWPOD, and OptCAM, respectively. Moreover, BWA-MEM, Bowtie2, and Nucmer4 require 15.7 GB, 22.6 GB, and 90.6 GB of memory, respectively, against 1MB for the mentioned optical approaches as reported in Table 19.

C) For the third comparison scenario, the processing time of the HELIOS optical architecture is analytically estimated and compared with that of GPU implementation of Smith-waterman. In this manner, various lengths of query sequences are aligned to the SWISS-PROT database [64], which contains 392,768 sequences and a total of 141,218,456 characters. In this regard, Table 20 reports the processing time of Smith-waterman, executed on NVIDIA TESLA K40 GPU with 44.3 giga cell updates per second (GCUPS), and on GTX 275 with 21 GCUPS [65]. Moreover, this table reports the analytical estimation of the processing time of the HELIOS optical architecture, assuming various switching rates and aperture sizes of the modulators As an instance, the HELIOS optical architecture aligns the query P27895 with the length of 1000 characters to SWISS-PROT database, in 1.4914 microseconds, utilizing a 1024 × 1024-pixel and 100MHz modulator; while it requires 4.54 seconds and 8.6 seconds to be executed on NVIDIA TESLA K40 GPU and GTX 275, respectively. As presented in this table, the HELIOS optical architecture is faster than GPU-based implementation of Smith-waterman by approximately 3.04 million and 5.76 million times, considering implementation on NVIDIA TESLA K40 GPU and GTX 275, respectively.

**Table 20:** Processing time of the HELIOS optical architecture, compared to Smith-waterman, assuming the SWISS-PROT database [64]. In this manner, various lengths of query sequences, from 144 to 5478 bases are aligned to the SWISS-PROT database [64], which contains 392,768 sequences and a total of 141,218,456 characters. In this regard, the processing times of Smith-waterman are reported by executing on NVIDIA TESLA K40 GPU with 44.3 giga cell updates per second (GCUPS) and GTX 275 with 21 GCUPS [65]. On the other hand, the processing times of HELIOS optical architecture are reported for a modulator with 1024 × 1024 pixels aperture size and 100 MHz switching rate [66] as a default, as well as for more recently developed modulators with various aperture sizes, such as 1920 × 1080 and 4096 × 4096 pixels, as well as various switching rates, including 35 GHz [72] and 4.5 THz [67].

Query	Length (bp)	Smith-Waterman (sec)		HELIOS Optical Architecture (sec)			
		GPU K40	GPU GTX 275	1024 × 1024 pixels & 100 MHz	4096 × 4096 pixels & 100 MHz	1920 × 1080 pixels & 35 GHz	1024 × 1024 pixels & 4.5 THz
P02232	144	0.65	1.24	3.7878e-07	5.9184e-09	2.9187e-10	8.4173e-12
P05013	189	0.85	1.65	4.9715e-07	7.7679e-09	3.8308e-10	1.1048e-11
P14942	222	1.01	1.93	5.8395e-07	9.1242e-09	4.4997e-10	1.2977e-11
P07327	375	1.7	3.24	9.8640e-07	1.5412e-08	7.6008e-10	2.1920e-11
P01008	464	2.1	3.99	9.8640e-07	1.9070e-08	9.4047e-10	2.7122e-11
P03435	567	2.57	4.89	1.2205e-06	2.3304e-08	1.1492e-09	3.3143e-11
P27895	1000	4.54	8.6	1.4914e-06	4.1100e-08	2.0269e-09	5.8453e-11
P07756	1500	6.81	12.91	3.9456e-06	6.1650e-08	3.0403e-09	8.7680e-11
P04775	2005	9.13	17.27	5.2739e-06	8.2405e-08	4.0639e-09	1.1720e-10
P19096	2504	11.38	21.54	6.5865e-06	1.0291e-07	5.0753e-09	1.4637e-10
P28167	3005	13.68	25.88	7.9043e-06	1.2351e-07	6.0908e-09	1.7565e-10
P0C6B8	3564	16.12	30.67	9.3747e-06	1.4648e-07	7.2238e-09	2.0833e-10
P20930	4061	18.48	34.97	1.0682e-05	1.6691e-07	8.2311e-09	2.3738e-10
Q9UKN1	5478	24.79	47.15	1.4409e-05	2.2515e-07	1.1103e-08	3.2021e-10

As verified by all the comparative studies, represented in Tables 18–20 the HELIOS method outperforms all alternative electrical algorithms in the processing time and memory requirement for all of the implementation scenarios, as well as, for various lengths of query sequences. This supremacy relies on the highly sophisticated HELIOS method and its optical architecture, which takes advantages of high-speed processing and operational parallelism in optics. Furthermore, the employed compact coding assumption highly enhances the performance of the HELIOS optical architecture, compared to other optical alternatives, specifically in terms of processing time.

### A quick review of optical implementation challenges

While this research mainly focuses on the design of the HELIOS method and its architecture, we would like to provide a brief review of the common challenges associated with the successful integration of an optical system into a product. In this manner five challenges are considered as follows: a) alignment tolerance, b) imprecision in optical components, c) speckle noises, d) thermal tolerance, and e) dust, humidity, and contaminants. These challenges mostly belong to mechanical engineering and the physical sciences of light waves and materials. Addressing each challenge in detail requires standalone research that discusses various solutions and probable arising issues. While we provide a brief review of each challenge, researchers and specialists in those areas of knowledge can expertly solve them. In this manner, various considerable research studies have discussed the aforementioned challenges [75–84] by employing a wide range of knowledge, including optics, physics, mechanics, electrics, and software programming.

#### Alignment tolerances

In optical systems, while there have been a lot of investments in a near-perfect design and fabrication of optical components, the lack of perfect alignment of the component causes performance issues. By a perfect alignment, we mean that the optical elements should be placed exactly where the theoretical optical and mechanical design specifies. Any small deviations from the designed shape of optical components cause a big difference in optical systems. As components might be slightly misaligned in the optical systems, the quality of light transmission is directly impacted, and consequently, the accuracy of the output decays [85]. Advancements in optical technologies have reduced the alignment tolerances to the nanometer levels [75, 76]. Therefore, even a few micrometers of misalignment can result in the loss of final output. As out-of-tolerance components do not cause complete functional failure, the progressive degradation of optical systems makes determinations of failure points more difficult. As an instance, several types of deviations in lens positions that affect the performance of the optical system include spacing, decentering, and tilt, which are defined as follows. a) Spacing, the space between optical elements, b) Tilt, the angle of the lens’s optical axis with respect to the axis of the device system, and c) decentering, misalignment of the optical axis with respect to the other devices.

As a key feature, the HELIOS method and its optical architecture are designed to be as simple as possible to reduce the probable challenges in the implementation phase. Notably, this simplicity comes with producing precise sequence alignment, compared to other well-known counterparts. As described in the Section “Optical architecture”, the functional part of the optical architecture which implements the HELIOS method (subsection “Optical modulation and mechanism unit”), only consists of eight components, including two lenses, five filters, and a chiral medium which is simple in comparison to its optical counterparts (for example 22 components in HAWPOD). Meanwhile, in the other parts of the architecture, the optical beam provision unit provides a clean optical beam to feed the system, and the output capturing unit captures the results respectively (which existed in all optical systems). Additionally, the HELIOS optical architecture prevents rotating and reflecting the beam to avoid the alignment complexities that they cause (HAWPOD and Moiré Technique do rotation and reflection). It places all components in a straight line and makes the structure simple. Therefore, passing optical beams through a straight path highly increases the alignment tolerance of the system.

Fortunately, photonic device manufacturers have been developing new alignment techniques with a wide range of mechanical, electrical, and software systems to manipulate alignment tolerance between optical components. As a result, an automated alignment process by implementing the optimal positioning system architecture is serial kinematics [86]. Serial kinematics uses a single actuator to position an optical component in one direction in space. By moving each axis independently from the others and only when needed, the serial kinematics: a) aligns optical components with less motion-induced error, b) eliminates additional joints, c) is capable of individual motions, or step sizes, of less than 10 nanometers, d) provides travel for each component to hundreds of millimeters, e) increases design modularity, f) simplifies programming, g) increases the flexibility of the implemented system, and h) finds home references for each axis very simple.

It should be noted in the employed filters, the size of each pixel is far more than 10 nm, which makes the alignment error negligible. For instance, it is 20 × 20 μm for 4.5 THz modulator [67], 450 × 250 nanometers for 100MHz modulator [66], 4.24 × 4.24 millimeters for 35GHz modulator [72]. By employing the serial kinematics and benefiting from the simple straight design of the HELIOS optical architecture, the alignment tolerance of the proposed system is addressed with high precision (less than 10 nanometers), without complicated challenges.

#### Imprecision in the optical components

All optical components built with advanced technology require a high degree of precision to meet their expected properties. Sometimes optical components are required to operate reliably under increasingly tough conditions. While a near-perfect design and fabrication of optical components have been achieved in recent years, any tiny imprecision in the optical component causes failure in their properties and functions. Manufacturing high-precision optical designs at the micron-level tolerances require seamless integration between their fundamental materials of glass or plastic (for the optical design) and metal (for the optomechanics). In recent decades by increasing the availability of low-price and high-power lasers, innovative industries provide more efficient materials processing, smaller components, increasingly detailed inspection, and greater accuracy [77, 78]. As an example, in our case study, for the resolution of an imaging lens, the imaging lens with less than one arcminute decenter can achieve a resolution of around 128 lp/mm at 20% contrast. However, when 2 of the elements were relaxed to allow less than six arcminute decenter, the imaging lens could only achieve a resolution of around 86 lp/mm at 20% contrast.

#### Speckle noises

Speckle is a granular interference that appears in images and diffraction patterns, produced by objects that are rough on the scale of an optical wavelength [87]. It is caused by multiple forward and backward scattering of light waves. It is a ubiquitous phenomenon that inherently exists in laser-based optical systems and directly degrades the quality of the optical system. Speckle noise in the optical systems impairs both the visual quality of the output and the performance of automatic analyses. The presence of speckle noise often obscures subtle but important details, and thus, is detrimental to high-resolution imaging systems. It also affects the performance of automatic analysis methods intended for objective and accurate quantifications. Although the resolution, speed, and depth of optical imaging systems have been greatly enhanced recently [79, 80], their intrinsic problem (i.e. speckle noise) has not been completely solved.

As the first attempt to reduce the speckle noise in HELIOS optical architecture, the optical beam provision unit employs a wideband unpolarized laser source [37], a laser line bandpass filter [38], and a pinhole, as depicted in Fig 6A. While the wideband laser generates an intense coherent monochromatic light beam in a wide spectral range, the laser line bandpass filter transmits laser light by suppressing ambient light as well as lower intensity secondary laser lines. It improves contrast by only transmitting light within a specific wavelength range (e.g. 450 to 650 nanometers). Afterward, the pinhole, placed at the focal point of the lenses, spatially filters the beams to reduce the high pulse energy density of the beam and to prevent arcing the air. Removing ambient beams reduces occurring speckle noises in the rest of the architecture. As well as, a spatial filter as an optical thresholder is placed at the end of the optical modulation and mechanism unit. It eliminates wavelength cross-talks and speckle noises of the output before capturing, and increases the quality of the results.

As an additional approach, in the case of speckle noises, as rough surfaces act differently with different wavelengths, we can use various sets of wavelengths (with slightly different offsets and steps, explained in Section “Coding procedure”). In this manner, the HELIOS optical architecture once performs the sequence alignment with the employed example of coding schema, shown in Figs 2 and 7. Afterward, a new set of wavelengths and polarizations are chosen (with different offsets and steps), and then the alignment process is performed again. By doing the sequence alignment procedure at least three times and then comparing their outputs, the probable occurred noises, due to cross-talk, speckle, etc, can be determined and removed. It is worth noting that the three-time execution of the sequence alignment multiplies the processing times by three and causes performance overhead. However, this overhead is negligible with the considerably low processing time of HELIOS optical architecture, represented in Tables 18–20. Additionally, more pixels of the modulators can be adopted to modulate each code, for example, 2 × 2 pixels instead of one pixel. It makes the results more resistant to various kinds of noises, with negligible overhead performance.

#### Thermal tolerance

Although an on-desk optical architecture is placed on a desk with a temperature that remains between 20 and 25°C for its entire useful life, any considerable change in the temperature causes materials to expand and contract, which results in damage to optical devices [81, 82]. The thermal expansion can: a) change the optical distances, b) shift the lens spacing, and c) change the focal lengths of lenses as the lens glass expands or contracts. So, temperature changes may cause an optic to lose focus. More importantly, as commonly used metal housings and optical glasses are rigid materials, stresses generated by a small change in component dimensions can be very high. These thermally induced stresses can lead to the failure of the lens or housing, sheering the bond lines and falling out of bonded lenses, and frustrating closely fit lenses. Additionally, when the temperature changes rapidly, some components or certain areas of a component experience temperature variation faster than others. It can lead to thermally induced malformation of components. Even if thermally induced stresses are low enough not to cause damage, they can cause stress-induced birefringence in lenses, tending to blur randomly polarized light.

Fortunately, the cracking lenses and sheering bonded interfaces can be avoided entirely by using lens mounts. Basically, lens mounts handle: a) the differences in thermal expansion and contraction between lenses and housing, and b) enough clearance between components to expand or contract as required. It is worth noting that all optical structures also include a temperature managing system to keep the structure at the same temperature. Moreover, material selection is another way to avoid problems with thermal expansion. For instance, optical glasses and metals can have similar coefficients of thermal expansion.

Each component employed in HELIOS architecture is mounted in its customized mount by the manufacturer. As depicted in Fig 6, the employed lenses in HELIOS optical architecture are made of glass and mounted in metal mounts, they work accurately in the temperature range of -20 to 60 °C. On the other hand, the employed modulators with 4.5 THz [67] offer speed stability in a wide range of temperature variations, i.e. 25 − 145 °C. So, it could be concluded that the HELIOS optical architecture (with the employed components) works precisely in the temperature range of 25 − 60 °C.

#### Dust, humidity, contaminants

Despite many electrical and mechanical systems, any tiny condensation, dirt, or dust rapidly degrades the performance of the optical systems [88]. Dust and dirt reduce the clarity of the light, while condensation can completely blind the light. While the outer surfaces of the outermost component are easily cleaned, the surfaces inside an optical system are mostly impossible to clean. Sadly, an optical system can be contaminated by itself from the inside. Furthermore, some materials, such as adhesives, rubbers, and plastics, can outgas. The outgassed materials can permanently condense on optical surfaces and blur the light.

By sealing the optics, most of the contaminants like dust and dirt can be managed [83, 84]. Elastomeric seals retain adequate compression under all circumstances, and at all positions. Dispensed sealants and adhesives undergo careful process development to ensure every assembly is adequately sealed. Additionally, most optical components are made waterproof, similar to all other waterproof devices. System waterproofing also makes it air-tight. Moreover, to prevent internal condensation, the optical assembly is built in a dry environment, like a cold space or a de-humidified space, and it greatly reduces the likelihood of condensation appearing on the inside optical surfaces. To prevent outgassing materials from condensing on the inside surfaces, working temperature limits of the component and side-effects of working out of those temperatures should be examined, which is directly related to its thermal tolerance. In application with high-power lasers, the components reach fairly high service temperatures. In these applications, the intolerant materials including plastics, adhesives, and rubbers should be eliminated from the design completely.

By assembling the structure in a dry environment, sealing it, and making it waterproof, the HELIOS optical architecture avoids most of the contaminates and internal condensation. Additionally, while the HELIOS optical architecture uses moderate laser power [37], the materials of employed components are made of glass and metal, which are resistant to the applied power of the laser. It prevents the whole structure from outgassing.

### Target applications

While sequence alignment algorithms play a significant role in bioinformatics, so far in this paper, it has been discussed as the best candidate applications to benefit from the parallel processing capability of the HELIOS method. As discussed above, this method facilitates three major data alignment scenarios, including, a) pair-wise genome alignment, b) alignment of two large sets of sequences, and c) reads alignment with a reference genome. Besides, the HELIOS method can be adopted to speed up the comparative studies with comparable accuracy for various types of biological sequences. In this manner, some major examples of the HELIOS applications are listed as follow:

- Identification of homologous, orthologous, and paralogous.
- Comparing a gene and its products.
- Function prediction and locating key features (e.g. catalytic domains, disulphide bridges).
- Identifying shared domains, and duplicated regions.
- Accelerating research studies on declaring genetic impairment and its detrimental effects (for example in genetic diseases).
- Advancing research projects on declaring genetic improvement and its beneficial effects (for instance in crop improvement).
- Speeding up the clinical tests to detect genetic diseases (such as Sickle-Cell).

Summarizing the above discussions, key advantages of the HELIOS method originate from its methodology, including high accuracy, simplicity, convenient output, no need to further processing, etc.; while its practical advantages arise from the HELIOS optical architecture, including high-speed processing, parallelism, optimal power consumption, no need for storage components, etc.

## Conclusion and future perspective

In this paper, a novel all-optical high-throughput sequence alignment method is proposed for aligning DNA, RNA, and protein sequences. The proposed method and its optical architecture are named HELIOS method and HELIOS optical architecture, respectively. The HELIOS method consists of two procedures to perform an accurate sequence alignment: a) coding procedure, and b) alignment procedure. It exactly determines the state of every character within input sequences, i.e., character matches, mutations, and indels. Moreover, the HELIOS optical architecture employs both high-speed processing and operational parallelism in the optical domain, as well as avoids various problems of electrical systems. It is built upon two units: a) optical beam provision unit, and b) optical modulation and mechanism unit, adopting polarization and wavelength of the optical beams.

For evaluation, the functionality and accuracy of the HELIOS method and its optical architecture are approved through behavioral and optical simulation studies, respectively. Furthermore, the complexity and performance of the HELIOS method and its optical architecture are calculated by analytical estimations. The results of accuracy evaluation indicate that the HELIOS method achieves comparable homologous alignment between two sequences, in comparison with the well-known algorithms. As our simulation results confirm, the alignment outputs by the HELIOS method are highly similar to those of SW, BLAST, MUSCLE, ClustalW, ClustalΩ, Kalign, and MAFFT; while there are some differences with the outputs of NW and T-Coffee. Moreover, according to our performance evaluation, the employed compact coding scheme highly escalates the number of input characters, and hence, it offers reduced time and space complexities, compared to the electrical and optical alternatives. Specifically, relying on its highly sophisticated method and optical architecture, as well as high-speed processing and operational parallelism in optics, the HELIOS optical architecture outperforms all alternative electrical and optical algorithms in terms of processing time and memory requirement.

As future works, we plan to enrich the method and the corresponding optical architecture as follows. Regarding the HELIOS method, the detection of further biological variations beyond mutations and indels will be addressed, including duplication, inversion, and translocation. Regarding the optical architecture, the efficiency of the HELIOS method will be improved by increasing the resolution of coded patterns, employing the amplitude and phase modulations of the optical beams. The latter improvement can boost the parallel processing capability of the proposed architecture.

## Supporting information

S1 Text Including Tables A1-A96, assuming dataset 1: *“Nine ND5 protein sequences dataset”* [53]. (PDF)

S2 Text Including Tables A1-A96, assuming dataset 2: *Nine beta globin protein sequences dataset* [53]. (PDF)

S3 Text Including Tables A1-A96, assuming dataset 3: *ND6 (NADH dehydrogenase subunit 6) proteins for eight different species* [55]. (PDF)

S4 Text Including Tables A1-A96, assuming dataset 4: *ENCODE Transcription Factor Targets Dataset, TFAP2A Gene Set* [56]. (PDF)

S5 Text Including Tables A1-A96, assuming dataset 5: *Hub Proteins Protein-Protein Interactions Dataset, AARR1 Gene Set* [57]. (PDF)

S6 Text Including Tables A1-A96, assuming dataset 6: *ChIP-X Enrichment Analysis Resource, CHEA Transcription Factor Targets Dataset, AP1M2 Gene set* [58]. (PDF)

S7 Text Including Tables A1-A96, assuming dataset 7: *ChIP-X Enrichment Analysis Resource, CHEA Transcription Factor Targets Dataset, AP1S2 Gene Set* [58]. (PDF)

S8 Text Including Tables A1-A96, assuming dataset 8: *Encyclopedia of DNA Elements Resource, ENCODE Transcription Factor Targets Dataset, SP1 Gene Set* [59]. (PDF)

S9 Text Including Tables A1-A96, assuming dataset 9: *Hub Proteins Resource, Protein-Protein Interactions Dataset, PTPN6 Gene Set* [57]. (PDF)

S10 Text Including Tables A1-A96, assuming dataset 10: *Kinase Enrichment Analysis Resource, KEA Substrates of Kinases Dataset, ULK Gene Set* [61]. (PDF)

S11 Text Including Tables A1-A96, assuming dataset 11: *Jaspar PWMs Resource, JASPAR Predicted Transcription Factor Targets Dataset, MAX Gene Set* [60]. (PDF)

S12 Text Including Tables A1-A96, assuming dataset 12: *Kinase Enrichment Analysis Resource, KEA Substrates of Kinases Dataset, YES1 Gene* Set [61]. (PDF)

## References

[1] Lesk A. Introduction to bioinformatics1. Oxford university press; 2019.

[2] Haque W, Aravind A, Reddy B. Pairwise sequence alignment algorithms: a survey. In: Proceedings of the 2009 conference on Information Science, Technology and Applications; 2009. p. 96–103.

[3] Kulkarni S, Roy S. Clinical genomics. Academic Press; 2014.

[4] Blake JD, Cohen FE. Pairwise sequence alignment below the twilight zone. Journal of molecular biology. 2001;307(2):721–735. doi: 10.1006/jmbi.2001.4495 11254392

[5] Zou H, Tang S, Yu C, Fu H, Li Y, Tang W. asw: accelerating Smith–Waterman algorithm on coupled CPU–GPU architecture. International Journal of Parallel Programming. 2019;47(3):388–402. doi: 10.1007/s10766-018-0617-3

[6] Jararweh Y, Al-Ayyoub M, Fakirah M, Alawneh L, Gupta BB. Improving the performance of the needleman-wunsch algorithm using parallelization and vectorization techniques. Multimedia Tools and Applications. 2019;78(4):3961–3977. doi: 10.1007/s11042-017-5092-0

[7] Boratyn GM, Thierry-Mieg J, Thierry-Mieg D, Busby B, Madden TL. Magic-BLAST, an accurate RNA-seq aligner for long and short reads. BMC bioinformatics. 2019;20(1):1–19. doi: 10.1186/s12859-019-2996-x 31345161 PMC6659269

[8] Hung CL, Lin YS, Lin CY, Chung YC, Chung YF. CUDA ClustalW: An efficient parallel algorithm for progressive multiple sequence alignment on Multi-GPUs. Computational biology and chemistry. 2015;58:62–68. doi: 10.1016/j.compbiolchem.2015.05.004 26052076

[9] Sievers F, Higgins DG. Clustal Omega for making accurate alignments of many protein sequences. Protein Science. 2018;27(1):135–145. doi: 10.1002/pro.3290 28884485 PMC5734385

[10] Notredame C, Higgins DG, Heringa J. T-coffee: a novel method for fast and accurate multiple sequence alignment11Edited by J. Thornton. Journal of Molecular Biology. 2000;302(1):205–217. doi: 10.1006/jmbi.2000.4042 10964570

[11] Lassmann T. Kalign 3: multiple sequence alignment of large datasets; 2020. 10.1093/bioinformatics/btz795 PMC7703769 31665271

[12] Edgar RC. MUSCLE: multiple sequence alignment with high accuracy and high throughput. Nucleic Acids Research. 2004;32(5):1792–1797. doi: 10.1093/nar/gkh340 15034147 PMC390337

[13] Rozewicki J, Li S, Amada KM, Standley DM, Katoh K. MAFFT-DASH: integrated protein sequence and structural alignment. Nucleic acids research. 2019;47(W1):W5–W10. doi: 10.1093/nar/gkz342 31062021 PMC6602451

[14] Marçais G, Delcher AL, Phillippy AM, Coston R, Salzberg SL, Zimin A. MUMmer4: A fast and versatile genome alignment system. PLOS Computational Biology. 2018;14(1):1–14. doi: 10.1371/journal.pcbi.1005944 29373581 PMC5802927

[15] Eddy SR. A Probabilistic Model of Local Sequence Alignment That Simplifies Statistical Significance Estimation. PLOS Computational Biology. 2008;4(5):1–14. doi: 10.1371/journal.pcbi.1000069 18516236 PMC2396288

[16] Chowdhury B, Garai G. A review on multiple sequence alignment from the perspective of genetic algorithm. Genomics. 2017;109(5):419–431. doi: 10.1016/j.ygeno.2017.06.007 28669847

[17] Cathey JJ. Theory and problems of electronic devices and circuits; 2002.

[18] Pal S, Mondal S, Das G, Khatua S, Ghosh Z. Big data in biology: The hope and present-day challenges in it. Gene Reports. 2020; p. 100869. doi: 10.1016/j.genrep.2020.100869

[19] Díaz D, Esteban FJ, Hernández P, Caballero JA, Dorado G, Gálvez S. Parallelizing and optimizing a bioinformatics pairwise sequence alignment algorithm for many-core architecture. Parallel Computing. 2011;37(4-5):244–259. doi: 10.1016/j.parco.2011.03.003

[20] Chatterjee K, Joshi S. An Overview on High Performance Issues of Parallel Architectures. Internet Technologies and Application Research. 2013;V.1:11–17. doi: 10.12966/itar.09.01.2013

[21] Baichoo S, Ouzounis CA. Computational complexity of algorithms for sequence comparison, short-read assembly and genome alignment. Biosystems. 2017;156:72–85. doi: 10.1016/j.biosystems.2017.03.003 28392341

[22] Zhang Y, Chan JWT, Chin FYL, Ting HF, Ye D, Zhang F, et al. On the Complexity of Constrained Sequences Alignment Problems. In: 8th International Frontiers of Algorithmics Workshop, FAW 2014; 2014. p. 309–319.

[23] Saleh BE, Teich MC. Fundamentals of photonics. john Wiley & sons; 2019.

[24] Javidi B, Horner JL. Real-time optical information processing. Academic Press; 2012.

[25] Keiser G. Biophotonics. Springer; 2016.

[26] Curilem Saldías M, Villarroel Sassarini F, Muñoz Poblete C, Vargas Vásquez A, Maureira Butler I. Image correlation method for DNA sequence alignment. PloS one. 2012;7(6):e39221. doi: 10.1371/journal.pone.0039221 22761742 PMC3384675

[27] Notredame C. Recent Evolutions of Multiple Sequence Alignment Algorithms. PLOS Computational Biology. 2007;3(8):1–4. doi: 10.1371/journal.pcbi.0030123 17784778 PMC1963500

[28] Wen Z, He J, Huang SY. Topology-independent and global protein structure alignment through an FFT-based algorithm. Bioinformatics. 2020;36(2):478–486. 31384919 10.1093/bioinformatics/btz609

[29] Ping P, Zhu X, Wang L. Similarities/dissimilarities analysis of protein sequences based on PCA-FFT. Journal of biological systems. 2017;25(01):29–45. doi: 10.1142/S0218339017500024

[30] Maleki E, Babashah H, Koohi S, Kavehvash Z. All-optical DNA variant discovery utilizing extended DV-curve-based wavelength modulation. J Opt Soc Am A. 2018;35(11):1929–1940. doi: 10.1364/JOSAA.35.001929 30461853

[31] Tanida J, Nitta K, Yahata A. Spatially coded moire matching technique for genome information visualization. In: Optical Information Processing Technology. vol. 4929. International Society for Optics and Photonics; 2002. p. 26–33. doi: 10.1117/12.483210

[32] Niita K, Togo H, Yahata A, Tanida J. Genome information analysis using spatial coded moiré technique. In: Technical Digest. CLEO/Pacific Rim 2001. 4th Pacific Rim Conference on Lasers and Electro-Optics (Cat. No. 01TH8557). vol. 2. IEEE; 2001. p. II–II.

[33] Maleki E, Babashah H, Koohi S, Kavehvash Z. High-speed all-optical DNA local sequence alignment based on a three-dimensional artificial neural network. J Opt Soc Am A. 2017;34(7):1173–1186. doi: 10.1364/JOSAA.34.001173 29036127

[34] Maleki E, Koohi S, Kavehvash Z, Mashaghi A. OptCAM: An ultra-fast all-optical architecture for DNA variant discovery. Journal of Biophotonics. 2020;13(1):e201900227. doi: 10.1002/jbio.201900227 31397961

[35] Akbari Rokn Abadi S, Hashemi Dijujin N, Koohi S. Optical pattern generator for efficient bio-data encoding in a photonic sequence comparison architecture. PLOS ONE. 2021;16(1):1–27. doi: 10.1371/journal.pone.0245095 PMC7810328 33449928

[36] Brodzik AK. Phase-only filtering for the masses (of DNA data): A new approach to sequence alignment. IEEE transactions on signal processing. 2006;54(6):2456–2466. doi: 10.1109/TSP.2006.873717

[37] Silfvast WT. Laser fundamentals. Cambridge university press; 2004.

[38] Niraula M, Yoon JW, Magnusson R. Single-layer optical bandpass filter technology. Opt Lett. 2015;40(21):5062–5065. doi: 10.1364/OL.40.005062 26512519

[39] Sze JR, Wei AC. Compact beam expander based on planar structure to avoid inner focus. Optical Review. 2016;23(5):842–847. doi: 10.1007/s10043-016-0251-5

[40] Abuleil M, Abdulhalim I. Narrowband multispectral liquid crystal tunable filter. Opt Lett. 2016;41(9):1957–1960. doi: 10.1364/OL.41.001957 27128048

[41] Ram BSB, Senthilkumaran P, Sharma A. Polarization-based spatial filtering for directional and nondirectional edge enhancement using an S-waveplate. Appl Opt. 2017;56(11):3171–3178. doi: 10.1364/AO.56.003171 28414377

[42] Hess AJ, Poy G, Tai JSB, Žumer S, Smalyukh II. Control of light by topological solitons in soft chiral birefringent media. Physical Review X. 2020;10(3):031042. doi: 10.1103/PhysRevX.10.031042

[43] Feng Y, Zhang HF, Wang J, Xu YL, Chen JT, Yang DX, et al. Design of a Nonvacuum-Cooling Compact CCD Camera for Scientific Detection. IEEE Transactions on Nuclear Science. 2019;66(10):2286–2292. doi: 10.1109/TNS.2019.2937540

[44] Chaisson MJ, Tesler G. Mapping single molecule sequencing reads using basic local alignment with successive refinement (BLASR): application and theory. BMC bioinformatics. 2012;13(1):1–18. doi: 10.1186/1471-2105-13-238 22988817 PMC3572422

[45] Li H, Durbin R. Fast and accurate short read alignment with Burrows–Wheeler transform. bioinformatics. 2009;25(14):1754–1760. doi: 10.1093/bioinformatics/btp324 19451168 PMC2705234

[46] Langmead B, Salzberg SL. Fast gapped-read alignment with Bowtie 2. Nature methods. 2012;9(4):357–359. doi: 10.1038/nmeth.1923 22388286 PMC3322381

[47] Darling AC, Mau B, Blattner FR, Perna NT. Mauve: multiple alignment of conserved genomic sequence with rearrangements. Genome research. 2004;14(7):1394–1403. doi: 10.1101/gr.2289704 15231754 PMC442156

[48] Harris RS. Improved pairwise alignment of genomic DNA. The Pennsylvania State University; 2007.

[49] Lai CC, Shih TP, Ko WC, Tang HJ, Hsueh PR. Severe acute respiratory syndrome coronavirus 2 (SARS-CoV-2) and coronavirus disease-2019 (COVID-19): The epidemic and the challenges. International journal of antimicrobial agents. 2020;55(3):105924. doi: 10.1016/j.ijantimicag.2020.105924 32081636 PMC7127800

[50] Anzagira L, Fossum ER. Color filter array patterns for small-pixel image sensors with substantial cross talk. J Opt Soc Am A. 2015;32(1):28–34. doi: 10.1364/JOSAA.32.000028 26366487

[51] Moss DS, Jelaska S, Pongor S. Essays in bioinformatics. vol. 368. IOS Press; 2005.

[52] Hamada M, Kiryu H, Iwasaki W, Asai K. Generalized Centroid Estimators in Bioinformatics. PloS one. 2011;6:e16450. doi: 10.1371/journal.pone.0016450 21365017 PMC3041832

[53] Abo-Elkhier MM, Abd Elwahaab MA, Abo El Maaty MI. Measuring Similarity among Protein Sequences Using a New Descriptor. BioMed research international. 2019;2019. doi: 10.1155/2019/2796971 31886192 PMC6893242

[54] Mount DW. Using BLOSUM in Sequence Alignments. Cold Spring Harbor Protocols. 2008;2008(6):pdb.top39. doi: 10.1101/pdb.top39 21356855

[55] Xie Xl, Zheng Lf, Yu Y, Liang Lp, Guo Mc, Song J, et al. Protein sequence analysis based on hydropathy profile of amino acids. Journal of Zhejiang University Science B. 2012;13(2):152–158. doi: 10.1631/jzus.B1100052 22302429 PMC3274743

[56] Consortium EP, et al. A user’s guide to the encyclopedia of DNA elements (ENCODE). PLoS biol. 2011;9(4):e1001046. doi: 10.1371/journal.pbio.1001046 21526222 PMC3079585

[57] Chen EY, Tan CM, Kou Y, Duan Q, Wang Z, Meirelles GV, et al. Enrichr: interactive and collaborative HTML5 gene list enrichment analysis tool. BMC bioinformatics. 2013;14(1):1–14. doi: 10.1186/1471-2105-14-128 23586463 PMC3637064

[58] Lachmann A, Xu H, Krishnan J, Berger SI, Mazloom AR, Ma’ayan A. ChEA: transcription factor regulation inferred from integrating genome-wide ChIP-X experiments. Bioinformatics. 2010;26(19):2438–2444. doi: 10.1093/bioinformatics/btq466 20709693 PMC2944209

[59] Consortium EP, et al. The ENCODE (ENCyclopedia of DNA elements) project. Science. 2004;306(5696):636–640. doi: 10.1126/science.1105136 15499007

[60] Mathelier A, Zhao X, Zhang AW, Parcy F, Worsley-Hunt R, Arenillas DJ, et al. JASPAR 2014: an extensively expanded and updated open-access database of transcription factor binding profiles. Nucleic acids research. 2014;42(D1):D142–D147. doi: 10.1093/nar/gkt997 24194598 PMC3965086

[61] Lachmann A, Ma’ayan A. KEA: kinase enrichment analysis. Bioinformatics. 2009;25(5):684–686. doi: 10.1093/bioinformatics/btp026 19176546 PMC2647829

[62] Kaul S, Koo HL, Jenkins J, Rizzo M, Rooney T, Tallon LJ, et al. Analysis of the genome sequence of the flowering plant Arabidopsis thaliana. nature. 2000;408(6814):796–815. doi: 10.1038/35048692 11130711

[63] Schneider VA, Graves-Lindsay T, Howe K, Bouk N, Chen HC, Kitts PA, et al. Evaluation of GRCh38 and de novo haploid genome assemblies demonstrates the enduring quality of the reference assembly. Genome research. 2017;27(5):849–864. doi: 10.1101/gr.213611.116 28396521 PMC5411779

[64] Bairoch A, Apweiler R. The SWISS-PROT Protein Sequence Data Bank and Its New Supplement TREMBL. Nucleic Acids Research. 1996;24(1):21–25. doi: 10.1093/nar/24.1.21 8594581 PMC145613

[65] Yahya M, Hasan L, Ali SA. High-throughput Protein Sequence Alignment on Multi-core Systems. International Journal of Integrated Engineering. 2020;12(7):62–71. doi: 10.30880/ijie.2020.12.07.007

[66] Soref RA, De Leonardis F, Passaro VM. Tunable optical-microwave filters optimized for 100 MHz resolution. Optics express. 2018;26(14):18399–18411. doi: 10.1364/OE.26.018399 30114020

[67] Shrekenhamer D, Montoya J, Krishna S, Padilla WJ. Four-color Metamaterial absorber THz spatial light modulator. Advanced Optical Materials. 2013;1(12):905–909. doi: 10.1002/adom.201300265

[68] Lee H, Gurtowski J, Yoo S, Marcus S, McCombie WR, Schatz M. Error correction and assembly complexity of single molecule sequencing reads. BioRxiv. 2014; p. 006395.

[69] Zook JM, Catoe D, McDaniel J, Vang L, Spies N, Sidow A, et al. Extensive sequencing of seven human genomes to characterize benchmark reference materials. Scientific data. 2016;3(1):1–26. doi: 10.1038/sdata.2016.25 27271295 PMC4896128

[70] Mikkelsen T, Hillier L, Eichler E, Zody M, Jaffe D, Yang SP, et al. Initial sequence of the chimpanzee genome and comparison with the human genome. Nature. 2005;437(7055):69–87. doi: 10.1038/nature04072 16136131

[71] Hu TT, Pattyn P, Bakker EG, Cao J, Cheng JF, Clark RM, et al. The Arabidopsis lyrata genome sequence and the basis of rapid genome size change. Nature genetics. 2011;43(5):476–481. doi: 10.1038/ng.807 21478890 PMC3083492

[72] Dalir H, Xia Y, Wang Y, Zhang X. Athermal broadband graphene optical modulator with 35 GHz speed. ACS photonics. 2016; 3(9):1564–1568. doi: 10.1021/acsphotonics.6b00398

[73] Boothby TC, Tenlen JR, Smith FW, Wang JR, Patanella KA, Nishimura EO, et al. Evidence for extensive horizontal gene transfer from the draft genome of a tardigrade. Proceedings of the National Academy of Sciences. 2015; 112(52):15976–15981. doi: 10.1073/pnas.1510461112 26598659 PMC4702960

[74] Koutsovoulos G, Kumar S, Laetsch DR, Stevens L, Daub J, Conlon C, et al. No evidence for extensive horizontal gene transfer in the genome of the tardigrade Hypsibius dujardini. Proceedings of the National Academy of Sciences. 2016; 113(18):5053–5058. doi: 10.1073/pnas.1600338113 27035985 PMC4983863

[75] Boudreau RA, Boudreau SM. Passive micro-optical alignment methods. CRC Press; 2018.

[76] Hinrichs KM, Piotrowski JJ. Neural networks for faster optical alignment. Optical Engineering. 2020;59(7):074107. doi: 10.1117/1.OE.59.7.074107

[77] Zolfaghari A. Fabrication of Precise Optical Components Using Electroforming Process and Precision Molding. The Ohio State University. 2021.

[78] Mimura H, Takei Y, Kume T, Takeo Y, Motoyama H, Egawa S, et al. Fabrication of a precise ellipsoidal mirror for soft X-ray nanofocusing. Review of Scientific Instruments. 2018;89(9):093104. doi: 10.1063/1.5035323 30278763

[79] Mohan E, Rajesh A, Sunitha G, Konduru RM, Avanija J, Ganesh Babu L. A deep neural network learning-based speckle noise removal technique for enhancing the quality of synthetic-aperture radar images. Concurrency and Computation: Practice and Experience. 2021;33(13):e6239. doi: 10.1002/cpe.6239

[80] Jeon W, Jeong W, Son K, Yang H. Speckle noise reduction for digital holographic images using multi-scale convolutional neural networks. Optics letters. 2018;43(17):4240–4243. doi: 10.1364/OL.43.004240 30160761

[81] Shaaban KS, Alomairy S, Al-Buriahi M. Optical, thermal and radiation shielding properties of B2O3–NaF–PbO–BaO–La2O3 glasses. Journal of Materials Science: Materials in Electronics. 2021;32(21):26034–26048.

[82] Nakamura F, Suda S, Kurosu T, Ibusuki Y, Noriki A, Tamai I, et al. Analyzing Thermal Tolerance of Mirror-based Optical Redistribution for Co-packaged Optics. In: CLEO: Science and Innovations. Optica Publishing Group; 2022. p. SF3O–8.

[83] Watts B, Pilet N, Sarafimov B, Witte K, Raabe J. Controlling optics contamination at the PolLux STXM. Journal of Instrumentation. 2018;13(04):C04001. doi: 10.1088/1748-0221/13/04/C04001

[84] Wang Z, Chen C, Liu Q, Lou Y, Suo Z. Extrusion, slide, and rupture of an elastomeric seal. Journal of the Mechanics and Physics of Solids. 2017;99:289–303. doi: 10.1016/j.jmps.2016.12.007

[85] Wang C, Xing S, Xu M, Shi H, Wu X, Fu Q, et al. The Influence of Optical Alignment Error on Compression Coding Superresolution Imaging. Sensors. 2022;22(7):2717. doi: 10.3390/s22072717 35408330 PMC9003395

[86] Xuan JQ, Xu SH, et al. Review on kinematics calibration technology of serial robots. International journal of precision engineering and manufacturing. 2014;15(8):1759–1774. doi: 10.1007/s12541-014-0528-1

[87] Goodman JW. Speckle phenomena in optics: theory and applications. Roberts and Company Publishers; 2007.

[88] Brown A, Bernot D, Ogloza A, Olson K, Thomas J, Talghader J. Physical origin of early failure for contaminated optics. Scientific reports. 2019;9(1):1–9. doi: 10.1038/s41598-018-37337-5 30679675 PMC6346064

