# Supplementary material for: HELIOS: High-speed sequence alignment in optics
Source: PLoS Comput Biol. 2022 Nov 21;18(11):e1010665. doi: 10.1371/journal.pcbi.1010665 (PMC9678324; doi:10.1371/journal.pcbi.1010665)
Supplement: S1 Text — (PDF) [file pcbi.1010665.s001.pdf]

HELIOS: High-Speed Sequence Alignment in Optics: S1 Text

Ehsan Maleki[1], **Saeedeh Akbari Rokn Abadi**[1], **and Somayyeh Koohi**[1,*]

[1] *Department of Computer Engineering, Sharif University of Technology, Azadi Ave., Tehran, Iran.*
[*] *Corresponding author: koohi@sharif.edu*

Compiled October 20, 2022

In response to the imperfections of current sequence alignment methods, originated from the inherent serialism within their corresponding electrical systems, a few optical approaches for biological data comparison have been proposed recently. However, due to their low performance, raised from their inefficient coding scheme, this paper presents a novel all-optical high-throughput method for aligning DNA, RNA, and protein sequences, named HELIOS. The HELIOS method employs highly sophisticated operations to locate character matches, single or multiple mutations, and single or multiple indels within various biological sequences. On the other hand, the HELIOS optical architecture exploits high-speed processing and operational parallelism in optics, by adopting wavelength and polarization of optical beams. For evaluation, the functionality and accuracy of the HELIOS method are approved through behavioral and optical simulation studies, while its complexity and performance are estimated through analytical computation. The accuracy evaluations indicate that the HELIOS method achieves a precise pairwise alignment of two sequences, highly similar to those of Smith-Waterman, Needleman-Wunsch, BLAST, MUSCLE, ClustalW, ClustalΩ, T-Coffee, Kalign, and MAFFT. According to our performance evaluations, the HELIOS optical architecture outperforms all alternative electrical and optical algorithms in terms of processing time and memory requirement, relying on its highly sophisticated method and optical architecture. Moreover, the employed compact coding scheme highly escalates the number of input characters, and hence, it offers reduced time and space complexities, compared to the electrical and optical alternatives. It makes the HELIOS method and optical architecture highly applicable for biomedical applications.

1. ACCURACY EVALUATION

In order to comprehensively assess the accuracy of the HELIOS method, two statistical analyses are performed through simulation studies: 1) Quantitative measurement of homology [1], and 2) Accuracy measurement of classification output [2], compared to the well-known algorithms, briefly reported in Tables A1 and A2, respectively. In this study, the *Nine ND5 protein sequences dataset* [3] is assumed in this study, as represented in Table A3.

A. Quantitative measurement of homology

To perform quantitative measurement of homology [1], the parameters Identity, Similarity, and Alignment Score of the HELIOS outputs are calculated through simulation studies, as reported in Tables A4-A6, respectively, assuming the *Nine ND5 protein sequences dataset* [3]. While the Identity reports the number of exactly matched characters of two sequences (in percentage), the Similarity measures the resemblance of two compared sequences. Specifically, regarding the physicochemical properties, the amino acids are categorized into six groups with different Similarity values; including GAVLI, FYW, STCM, KRH, DENQ, and P. As the third metric, the BLOSUM62 [4] substitution scoring matrix [4] is adopted to calculate the Alignment Score, with gap opening and extension penalties equal to -10 and -0.5, respectively.

For a comparative study, the values of Identity, Similarity, and Alignment Score of the quantitative measurement of homology is performed by various well-known algorithms to be compared to the those of HELIOS method, assuming *Nine ND5 protein sequences dataset* [3]. It includes Smith-Waterman (SW) [5] reported in Tables A7-A9, Needleman-Wunsch (NW) [6] reported in Tables A10-A12, BLAST [7] reported in Tables A13-A15, ClustalW [8] reported in Tables A16-A18, Clustal-Omega [9] reported in Tables A19-A21, MUSCLE [9] reported in Tables A22-A24, T-Coffee [10] reported in Tables A25-A27, Kalign [11] reported in Tables A28-A30, and MAFFT [12] reported in Tables A31-A33.

B. Accuracy measurement of classification output

Afterward, the accuracy measurement of the classification output [2] of the HELIOS method is addressed by calculating the val-

Table A1. A brief report of the quantitative measurement of homology of the HELIOS method, compared to nine well-known algorithms, including SW, NW, BLAST, ClustalW, Clustal Omega, Muscle, T-Coffee, Kalign, and MAFFT. The parameters Identity, Similarity, and Alignment score are averaged and reported. The dataset used in this study is the *Nine ND5 protein sequences dataset* [3].

	HELIOS	SW	NW	BLAST	MUSCLE	ClustalW	ClustalΩ	T-Coffee	Kalign	MAFFT
Identity	76.959	76.580	76.580	76.274	76.292	76.292	76.263	76.344	76.337	76.388
Similarity	83.365	83.623	83.605	83.358	83.325	83.295	83.306	83.358	83.373	83.424
Alignment Score	2314.5	2380.3	2389.9	2373.5	2381.3	2381.0	2380.3	2380.6	2382.3	2383.6

Table A2. A brief report of the accuracy measurement of classification output of the HELIOS method with referencing well-known algorithms, including SW, NW, BLAST, ClustalW, Clustal Omega, Muscle, T-Coffee, Kalign, and MAFFT. The parameters SEN, Spec, Acc, PPV, NPV, MCC, and F-Score are averaged and reported. The dataset used in this study is the *Nine ND5 protein sequences dataset* [3].

	SW	NW	BLAST	MUSCLE	ClustalW	ClustalΩ	T-Coffee	Kalign	MAFFT
SEN	0.94972	0.79205	0.95190	0.95424	0.95491	0.95476	0.95557	0.95561	0.95613
Spec	0.99994	0.99968	0.99994	0.99995	0.99995	0.99995	0.99995	0.99995	0.99990
ACC	0.99985	0.99933	0.99986	0.99987	0.99987	0.99987	0.99987	0.99988	0.99988
PPV	0.96055	0.80181	0.96273	0.96636	0.96704	0.96689	0.96771	0.96775	0.96828
NPV	0.99992	0.99966	0.99992	0.99992	0.99993	0.99993	0.99993	0.99993	0.99993
MCC	0.95503	0.79657	0.95722	0.96020	0.96087	0.96072	0.96155	0.96159	0.96211
F-Score	0.95508	0.79687	0.95726	0.96023	0.96090	0.96075	0.96157	0.96161	0.96210

ues of Sensitivity (SEN), Specificity (Spec), Accuracy (ACC), Positive Predictive Value (PPV), Negative Predictive Value (NPV), Matthew's Coefficient Correlation (MCC), and Test's Accuracy (F-Score) in the simulation studies, according to Eq 5 to Eq 11, respectively.

As a comparative study, the accuracy measurement of the classification output of the HELIOS method is accomplished, assuming *Nine ND5 protein sequences dataset* [3], and the corresponding metrics are calculated with considering Smith-Waterman [5] reported in Tables A34-A40, Needleman-Wunsch [6] reported in Tables A41-A47, ClustalW [8] reported in Tables A55-A61, Clustal-Omega [9] reported in Tables A62-A68, BLAST [7] reported in Tables A48-A54, MUSCLE [13] reported in Tables A69-A75, T-Coffee [10] reported in Tables A76-A82, Kalign [11] reported in Tables A83-A89, and MAFFT [12] reported in Tables A90-A96.

REFERENCES

1. D. S. Moss, S. Jelaska, and S. Pongor, *Essays in bioinformatics*, vol. 368 (IOS Press, 2005).
2. M. Hamada, H. Kiryu, W. Iwasaki, and K. Asai, "Generalized centroid estimators in bioinformatics," PloS one **6**, e16450 (2011).
3. M. M. Abo-Elkhier, M. A. Abd Elwahaab, and M. I. Abo El Maaty, "Measuring similarity among protein sequences using a new descriptor," BioMed research international **2019** (2019).
4. D. W. Mount, "Using blosum in sequence alignments," Cold Spring Harb. Protoc. **2008**, pdb.top39 (2008).
5. H. Zou, S. Tang, C. Yu, H. Fu, Y. Li, and W. Tang, "asw: accelerating smith–waterman algorithm on coupled cpu–gpu architecture," Int. J. Parallel Program. **47**, 388–402 (2019).
6. Y. Jararweh, M. Al-Ayyoub, M. Fakirah, L. Alawneh, and B. B. Gupta, "Improving the performance of the needleman-wunsch algorithm using parallelization and vectorization techniques," Multimed. Tools Appl. **78**, 3961–3977 (2019).
7. G. M. Boratyn, J. Thierry-Mieg, D. Thierry-Mieg, B. Busby, and T. L. Madden, "Magic-blast, an accurate rna-seq aligner for long and short reads," BMC bioinformatics **20**, 1–19 (2019).
8. D. Díaz, F. J. Esteban, P. Hernández, J. A. Caballero, A. Guevara, G. Dorado, and S. Gálvez, "Mc64-clustalwp2: A highly-parallel hybrid strategy to align multiple sequences in many-core architectures," PLOS ONE **9**, 1–12 (2014).
9. F. Sievers and D. G. Higgins, "Clustal omega for making accurate alignments of many protein sequences," Protein Sci. **27**, 135–145 (2018).
10. C. Notredame, D. G. Higgins, and J. Heringa, "T-coffee: a novel method for fast and accurate multiple sequence alignment11edited by j. thornton," J. Mol. Biol. **302**, 205–217 (2000).
11. T. Lassmann, "Kalign 3: multiple sequence alignment of large datasets," (2020).
12. J. Rozewicki, S. Li, K. M. Amada, D. M. Standley, and K. Katoh, "Mafft-dash: integrated protein sequence and structural alignment," Nucleic acids research **47**, W5–W10 (2019).
13. R. C. Edgar, "MUSCLE: multiple sequence alignment with high accuracy and high throughput," Nucleic Acids Res. **32**, 1792–1797 (2004).

Table A3. The list of input sequences, assuming the *Nine ND5 protein sequences dataset* [3].

Name	Sequence
Homo sapiens	MTMHT TMTTL TLTSL IPPIL TTLVN PNKKN SYPHY VKSIV ASTFI ISLFP TTMFM CLDQE VIISN WHWAT TQTTQ LSLSF KLDYF SMMFI PVALF VTWSI MEFSL WYMNS DPNIN QFFKY LLIFL ITMLI LVTAN NLFQL FIGWE GVGIM SFLLI SWWYA RADAN TAAIQ AILYN RIGDI GFILA LAWFI LHSNS WDPQQ MALLN ANPSL TPLLG LLLAA AGKSA QLGLH PWLPS AMEGP TPVSA LLHSS TMVVA GIFLL IRFHP LAENS PLIQT LTLCL GAITT LFAAV CALTQ NDIKK IVAFS TSSQL GLMMV TIGIN QPHLA FLHIC THAFF KAMLF MCSGS IIHNL NNEQD IRKMG GLLKT MPLTS TSLTI GSLAL AGMPF LTGFY SKDHI IETAN MSYTN AWALS ITLIA TSLTS AYSTR MILLT LTGQP RFPTL TNINE NNPTL LNPIK RLAAG SLFAG FLITN NISPA SPFQT TIPLY LKLTA LAVTF LGLLT ALDLN YLTNK LKMKS PLCTF YFSNM LGFYP SITHR TIPYL GLLTS QNLPL LLLDL TWLEK LLPKT ISQHQ ISTSI ITSTQ KGMIK LYFLS FFFPL ILTLL LIT
Rattus norvegicus	MNMMT ILILM ILDLL TTPIT FSMTI LTKLM YFRHM LITSI KFSFL LSLLP LLLFF HHNTE YMITN WHWLT INSIK LTMSF KIDYF SILFL SVSLF VTWSI MQFSS WYMHS DPHIN RFIKY LMMFL NNMLI LTSAN NLFQL FIGWE GVGIM SFLLI GWWYG LADAN TAALQ AILYN RVGDI GFILA MTWFC LNMNS WELQQ IFLTN TNNNL VPLTG LLIAA TGKSA QFGLH PWLPS AMEGP TPVSA LLHSS TMVVA GIFLM IRFHP LTSNN STIMT AMLCL GAITT LFTAI CALTQ NDIKK IVAFS TSSQL GLMMV TLGIN QPYLA FLHIC THAFF KAMLF MCSGS IIHIL NDEQD IRKMG NMMKA MPFTS SCLII GSLAL TGMPF LTGFY SKDLI IEAIN TCNTN AWALM ITLIA TSMTA VYSMR IIYFV TMTKP RYSPL ITINE NNPNL INPIK RLALG SILAG FLISL NIPPT NIQIL TMPWH LKMTA LLITI LGFAI ALELN NLTLN LSMSK PTKLS SFSTS LGYYP PIMPR IIPQK TLNSS YKLSL NLLDL IWSEK TIPKS TSITQ TQLSK MMSNQ KGLIK LYFLS FLITI SLIFI LHTLN PEWFQ
Balaenoptera physalus	MNLFT SFTLL TLLIL TTPIM MSHTG SHVNN KYQSY VKNIV FCAFI TSLVP AMVYL HTNQE TLISN WHWIT IQTLK LTLSF KMDYF SLMFM PVALF ITWSI MEFSM WYMHS DPYIN QFFKY LLLFL ITMLI LVTAN NLFQL FIGWE GVGIM SFLLI GWWFG RTDAN TAALQ AILYN RIGDI GLLAS MAWFL SNMNT WDLEQ IFMLN QNPLN FPLMG LVLAA AGKSA QFGLH PWLPS AMEGP TPVSA LLHSS TMVVA GIFLL VRFYP LMENN KLIQT VTLCL GAITT LFTAI CALTQ NDIKK IIAFS TSSQL GLMMV TIGLN QPYLA FLHIC THAFF KAMLF LCSGS IIHNL NNEQD IRKMG GLFKA LPFTT TALII GCLAL TGMPF LTGFY SKDPI IEAAT SSYTN AWALL LTLIA TSLTA VYSTR IIFFA LLGQP RFPPS TTINE NNPLL INPIK RLLVG SIFAG FILSN SIPPM TTPLM TMPLH LKLTA LAMTT LGFII AFEIN LDTQN LKHKH PSNSF KFSTL LGYFP TIMHR LPPHL DLLMS QKLAT SLLDL TWLET ILPKT TALIQ LKAST LTSNQ QGLIK LYFLS FLITI TLSMI LFNYPE
Balaenoptera musculus	MNLFT SFVLL TLLIL FTPIM VSNTD PHKNN KYQSY VKNIV FCAFI TSLIP AMMYL HTNQE TLISN WHWIT IQTLK LTLSF KMDYF SLMFM PVALF ITWSI MEFSM WYMHS DPYIN QFFKY LLLFL ITMLI LVTAN NLFQL FIGWE GVGIM SFLLI GWWFG RTDAN TAALQ AILYN RIGDI GLLAS MAWFL SNMNT WDLQQ IFMLN QNPLN FPLMG LVLAA AGKSA QFGLH PWLPS AMEGP TPVSA LLHSS TMVVA GIFLL VRFYP LMENN KLIQT VTLCL GAITT LFTAI CALTQ NDIKK IIAFS TSSQL GLMMV TIGLN QPYLA FLHIC THAFF KAMLF LCSGS IIHNL NNEQD IRKMG GLFKA LPFTT TALII GCLAL TGMPF LTGFY SKDPI IEAAT SSYTN AWALL LTLTA TSLTA VYSTR IIFFA LLGQP RFPPS TTINE NNPLL INPIK RLLIG SIFAG FILSN SIPPV ITPLM TMPLH LKLTA LTMTT LGFII AFEIN LDTQN LKYTH PSNPF KFSTL LGYFP TIMHR LPPHL DLSMS QKLAT SLLDL TWLET TLPKT TALIQ LKAST LTSNQ QGLIK LYFLS FLITI TLSMI LFNCPE
Didelphis virginiana	MKVIN ISNTM SIMSI ILLIL PLLYN LLSLK KINFP LYCKN MIMLA FMMSL PSLLL FMYKG QESII TNWHW FSIHS FNISM SFKMD FFSII FIPIA LFVTW AILEF SLWYM HSDPN ISQFF KYLII FLLTM IILVS ANNLF QLFIG WEGVG IMSFL LIGWW YGRSD ANTAA LQAIL YNRIG DIGFM LTMAW LMLNC NSWDL QHIFS MNMHP IALLG LIIAA TGKSA QFSLH PWLPS AMEGP TPVSA LLHSS TMVVA GIFLL IRFHP MLENN KTMLT ITLCL GALTT LFTAM CAIMQ NDIKK IVAFS TSSQL GLMMV TVGLN QPHLA FLHIC THAFF KAMLF MCSGS IIHNL NDEQD IRKMG GLFYT LPITS SALMT GSLAL MGTPF LAGFY SKDSI IEAMN TSYTN SWALT ITLIA TSLTA IYSLR IIYYT LLGHP RFMTM SPLNE NNPNL INPII RLALG TIFAG FMLTT NMPPS YSITM TMPMF IKQMA LMVTT TGLMM GMELN SLTNK LMKSN NHTNN FLTML GFYTQ IMHRM QPLIS LFMGQ RIATM LIDMN WYEKA GPKGQ ANVHS KLSSL ISSSQ KGLMK MYFLS FLVSM IFIIL FT
Pan troglodytes	MTMYA TMTTL ALTSL IPPIL GALIN PNKKN SYPHY VKSII ASTFI ISLFP TTMFM CLDQE TIISN WHWAT TQTTQ LSLSF KLDYF SMTFI PVALF VTWSI MEFSL WYMDS DPNIN QFFKY LLIFL ITMLI LVTAN NLFQL FIGWE GVGIM SFLLI SWWYA RTDAN TAAIQ AILYN RIGDI GFVLA LAWFL LHSNS WDPQQ MILLS TNTDL TPLLG FLLAA AGKSA QLGLH PWLPS AMEGP TPVSA LLHSS TMVVA GIFLL IRFYP LAENN PLIQT LTLCL GAITT LFAAV CALTQ NDIKK IVAFS TSSQL GLMMV TIGIN QPHLA FLHIC THAFF KAMLF MCSGS IIHNL NNEQD IRKMG GLLKT MPLTS TSLTI GSLAL AGMPF LTGFY SKDLI IETAN MSYTN AWALS ITLIA TSLTS AYSTR MILLT LTGQP RFPTL TNINE NNPTL LNPIK RLTIG SLFAG FLITN NILPM STPQV TIPLY LKLTA LGVTS LGLLT ALDLN YLTSK LKMKS PLYTF HFSNM LGFYP NIMHR SIPYL GLLTS QNLPL LLLDL TWLEK LLPKT ISQYQ ISASI TTSTQ KGMIK LYFLS FFFPL ILTLL LIT
Pan paniscus	MTMYT TMTTL TLTPL ILPIL TTLIN PNKKN SYPHY VKSII ASTFI ISLFP TTMFM CLDQE AIISN WHWAT TQTTQ LSLSF KLDYF SMTFI PVALF VTWAI MEFSL WYMNS DPNIN QFFKY LLIFL ITMLI LVTAN NLFQL FIGWE GVGIM SFLLI SWWYA RTDAN TAAIQ AILYN RIGDI GFILA LAWFL LHSNS WDPQQ MVLLS TNPSL TPLLG FLLAA AGKSA QLGLH PWLPS AMEGP TPVSA LLHSS TMVVA GVFLL IRFHP LAENN PLIQT LTLCL GAITT LFAAI CALTQ NDIKK IVAFS TSSQL GLMMV TIGIN QPHLA FLHIC THAFF KAMLF MCSGS IIHNL NNEQD IRKMG GLLKT MPLTS TSLII GSLAL AGMPF LTGFY SKDLI IETAN MSYMN AWALS ITLIA TSLTS AYSTR MILLT LMGQP RFPTL TNINE NNPTL LNPIK RLTIG SLFAG FFITN NILPM STSQM TIPLY LKLTA LSVTF LGLLT ALDLN YLTNK LKMKS PPYTF YFSNM LGFYP NIMHR SIPYL GLLTS QNLPL LLLDL TWLEK LLPKT ISQYQ VSASI TTSTQ KGMIK LYFLS FLFPL ILTLL LIM
Gorilla gorilla	MTMYA TMTTL ALTSL IPPIL TTFIN PNKKS SYPHY VKSIV ASTFI ISLFP TTMFL CLDQE AIISS WHWAT TQTIQ LSLSF KLDYF SMMFI PVALF VTWSI MEFSL WYMNS DPNIN QFFKY LLIFL ITMLI LVTAN NLFQL FIGWE GVGIM SFLLI GWWYA RTDAN TAAVQ AILYN RIGDI GFILA LAWFL LHSNS WDPQQ MSLLN TNPNL IPLLG FLLAA AGKSA QLGLH PWLPS AMEGP TPVSA LLHSS TMVVA GVFLL IRFRH LAENN SLAQT LTLCL GAITT LFAAV CALTQ NDIKK IVAFS TSSQL GLMVA TIGIG QPHLA FLHIC THAFF KAMLF MCSGS IIHNL NNEQD IRKMG GLLKA MPLTS TSLAI GSLAL MGMPF LTGFY SKDLI IETAN MSHTN AWALS IILIA TSLTS AYSTR MILLT LTGQP RFPTF ANINE NYSTL LNPIK RLTIG SLFAG FFITN NILPT SVPQM TIPLY LKLTA LSITL LGLLT ALDLN YLTNK LKMKH PPHTF YFSNM LGFYP NITHR TIPYL GLLMS QNLPL LLLDL IWLEK LLPKT ISQHQ ISASI TTSTQ KGLIK LYFLS FFFPL LLILL LIT
Mus musculus	MNIFT TSILL IFILL LSPIL ISMSN LIKHI NFPLY TTTSI KFSFI ISLLP LLMFF HNNME YMITT WHWVT MNSME LKMSF KTDFF SILFT SVALF VTWSI MQFSS WYMHS DPNIN RFIKY LTLFL ITMLI LTSAN NMFQL FIGWE GVGIM SFLLI GWWYG RTDAN TAALQ AILYN RIGDI GFILA MVWFS LNMNS WELQQ IMFSN NNDNL IPLMG LLIAA TGKSA QFGLH PWLPS AMEGP TPVSA LLHSS TMVVA GIFLL VRFHP LTTNN NFILT TMLCL GALTT LFTAI CALTQ NDIKK IIAFS TSSQL GLMMV TLGMN QPHLA FLHIC THAFF KAMLF MCSGS IIHSL ADEQD IRKMG NITKI MPFTS SCLVI GSLAL TGMPF LTGFY SKDLI IEAIN TCNTN AWALL ITLIA TSMTA MYSMR IIYFV TMTKP RFPPL ISINE NDPDL MNPIK RLAFG SIFAG FVISY NIPPT SIPVL TMPWF LKTTA LIISV LGFLI ALELN NLTMK LSMNK ANPYS SFSTL LGFFP SIIHR ITPMK SLNLS LKTSL TLLDL IWLEK TIPKS TSTLH TNMTT LTTNQ KGLIK LYFMS FLINI ILIII LYSIN LE

Table A4. The parameter Identity of the HELIOS method in the quantitative measurement of homology, assuming the *Nine ND5 protein sequences dataset* [3].

	Homo sapiens	Rattus norvegicus	Balaenoptera physalus	Balaenoptera musculus	Didelphis virginiana	Pan troglodytes	Pan paniscus	Gorilla gorilla	Mus musculus
Homo sapiens	100	64.1791	69.8176	69.3201	62.2924	93.3665	93.2007	90.2156	64.8425
Rattus norvegicus		100	66.8317	66.5017	60.7973	63.8474	64.0133	63.1841	78.2537
Balaenoptera physalus			100	96.5347	61.6279	70.1493	70.6468	69.3201	66.5017
Balaenoptera musculus				100	62.2924	69.9834	69.8176	68.9884	66.8317
Didelphis virginiana					100	62.4585	62.7907	60.9635	63.6213
Pan troglodytes						100	95.0249	90.7131	64.3449
Pan paniscus							100	91.0448	64.5108
Gorilla gorilla								100	64.3449
Mus musculus									100

Table A5. The parameter Similarity of the HELIOS method in the quantitative measurement of homology, assuming the *Nine ND5 protein sequences dataset* [3].

	Homo sapiens	Rattus norvegicus	Balaenoptera physalus	Balaenoptera musculus	Didelphis virginiana	Pan troglodytes	Pan paniscus	Gorilla gorilla	Mus musculus
Homo sapiens	100	72.6368	77.7778	76.7828	72.5914	95.1907	95.8541	92.3715	73.9635
Rattus norvegicus		100	77.8878	77.2277	70.7641	72.9685	72.6368	72.9685	84.8435
Balaenoptera physalus			100	97.0297	75.4153	77.9436	78.2753	77.6119	77.3927
Balaenoptera musculus				100	75.2492	76.7828	76.7828	76.4511	78.0528
Didelphis virginiana					100	72.5914	74.0864	71.9269	73.0897
Pan troglodytes						100	96.5174	93.2007	74.1294
Pan paniscus							100	93.864	73.466
Gorilla gorilla								100	75.1244
Mus musculus									100

Table A6. The parameter Alignment Score of the HELIOS method in the quantitative measurement of homology, assuming the *Nine ND5 protein sequences dataset* [3].

	Homo sapiens	Rattus norvegicus	Balaenoptera physalus	Balaenoptera musculus	Didelphis virginiana	Pan troglodytes	Pan paniscus	Gorilla gorilla	Mus musculus
Homo sapiens	3091	1831.5	1938.5	2003.5	1846	2884	2889	2781	1903.5
Rattus norvegicus		3139	1988	2038	1757	1796.5	1823.5	1768	2396
Balaenoptera physalus			3125	3020	1930	2034	2022.5	1992	1948.5
Balaenoptera musculus				3132	1921	2005	2037.5	1997.5	2005
Didelphis virginiana					3100	1825.5	1920	1784	1865.5
Pan troglodytes						3085	2942	2810	1874
Pan paniscus							3088	2804	1894
Gorilla gorilla								3086	1915.5
Mus musculus									3115

Table A7. The parameter Identity of the Smith-Waterman in the quantitative measurement of homology, assuming the *Nine ND5 protein sequences dataset* [3].

	Homo sapiens	Rattus norvegicus	Balaenoptera physalus	Balaenoptera musculus	Didelphis virginiana	Pan troglodytes	Pan paniscus	Gorilla gorilla	Mus musculus
Homo sapiens	100	63.6816	68.4909	68.325	62.7907	93.3665	93.2007	90.2156	64.6766
Rattus norvegicus		100	66.3366	66.5017	60.9635	63.1841	63.1841	62.8524	78.2537
Balaenoptera physalus			100	96.5347	61.6279	68.6567	68.8226	67.33	65.5116
Balaenoptera musculus				100	61.794	68.6567	68.8226	67.4959	65.5116
Didelphis virginiana					100	62.4585	62.7907	60.9635	63.4551
Pan troglodytes						100	95.0249	90.7131	64.6766
Pan paniscus							100	90.8789	64.3449
Gorilla gorilla								100	64.0133
Mus musculus									100

Table A8. The parameter Similarity of the Smith-Waterman in the quantitative measurement of homology, assuming the *Nine ND5 protein sequences dataset* [3].

	Homo sapiens	Rattus norvegicus	Balaenoptera physalus	Balaenoptera musculus	Didelphis virginiana	Pan troglodytes	Pan paniscus	Gorilla gorilla	Mus musculus
Homo sapiens	100	72.9685	77.7778	77.2803	74.0864	95.1907	95.8541	92.3715	75.1244
Rattus norvegicus		100	78.2178	77.3927	71.0963	73.1343	72.6368	74.461	85.0082
Balaenoptera physalus			100	97.0297	76.412	77.6119	77.7778	76.7828	77.7228
Balaenoptera musculus				100	75.9136	76.9486	77.1144	76.1194	77.5578
Didelphis virginiana					100	74.0864	74.5847	72.7575	73.0897
Pan troglodytes						100	96.5174	93.2007	75.4561
Pan paniscus							100	93.864	74.2952
Gorilla gorilla								100	75.6219
Mus musculus									100

Table A9. The parameter Alignment Score of the Smith-Waterman in the quantitative measurement of homology, assuming the *Nine ND5 protein sequences dataset* [3].

	Homo sapiens	Rattus norvegicus	Balaenoptera physalus	Balaenoptera musculus	Didelphis virginiana	Pan troglodytes	Pan paniscus	Gorilla gorilla	Mus musculus
Homo sapiens	3091	1913	2157	2152	1940	2884	2889	2781	1951
Rattus norvegicus		3139	2066	2076	1870	1911	1925	1901	2474
Balaenoptera physalus			3125	3020	1963	2180	2185	2131	2059.5
Balaenoptera musculus				3132	1962.5	2171	2179	2128	2097
Didelphis virginiana					3100	1915	1949	1894.5	1950
Pan troglodytes						3085	2942	2810	1957.5
Pan paniscus							3088	2818	1984.5
Gorilla gorilla								3086	1967.5
Mus musculus									3115

Table A10. The parameter Identity of the Needleman-Wunsch in the quantitative measurement of homology, assuming the *Nine ND5 protein sequences dataset* [3].

	Homo sapiens	Rattus norvegicus	Balaenoptera physalus	Balaenoptera musculus	Didelphis virginiana	Pan troglodytes	Pan paniscus	Gorilla gorilla	Mus musculus
Homo sapiens	100	63.6816	68.4909	68.325	62.7907	93.3665	93.2007	90.2156	64.6766
Rattus norvegicus		100	66.3366	66.5017	60.9635	63.1841	63.1841	62.8524	78.2537
Balaenoptera physalus			100	96.5347	61.6279	68.6567	68.8226	67.33	65.5116
Balaenoptera musculus				100	61.794	68.6567	68.8226	67.4959	65.5116
Didelphis virginiana					100	62.4585	62.7907	60.9635	63.4551
Pan troglodytes						100	95.0249	90.7131	64.6766
Pan paniscus							100	90.8789	64.3449
Gorilla gorilla								100	64.0133
Mus musculus									100

Table A11. The parameter Similarity of the Needleman-Wunsch in the quantitative measurement of homology, assuming the *Nine ND5 protein sequences dataset* [3].

	Homo sapiens	Rattus norvegicus	Balaenoptera physalus	Balaenoptera musculus	Didelphis virginiana	Pan troglodytes	Pan paniscus	Gorilla gorilla	Mus musculus
Homo sapiens	100	72.9685	77.7778	77.2803	74.0864	95.1907	95.6882	92.3715	75.1244
Rattus norvegicus		100	78.2178	77.3927	71.0963	73.1343	72.471	74.461	85.0082
Balaenoptera physalus			100	97.0297	76.412	77.6119	77.7778	76.7828	77.7228
Balaenoptera musculus				100	75.9136	76.9486	77.1144	76.1194	77.5578
Didelphis virginiana					100	74.0864	74.5847	72.7575	73.0897
Pan troglodytes						100	96.3516	93.2007	75.4561
Pan paniscus							100	93.6982	74.1294
Gorilla gorilla								100	75.6219
Mus musculus									100

Table A12. The parameter Alignment Score of the Needleman-Wunsch in the quantitative measurement of homology, assuming the *Nine ND5 protein sequences dataset* [3].

	Homo sapiens	Rattus norvegicus	Balaenoptera physalus	Balaenoptera musculus	Didelphis virginiana	Pan troglodytes	Pan paniscus	Gorilla gorilla	Mus musculus
Homo sapiens	3091	1913	2168	2163	1962	2884	2890	2781	1962.5
Rattus norvegicus		3139	2077	2087	1894	1924	1942	1914	2485
Balaenoptera physalus			3125	3020	1985.5	2191	2198	2142	2075.5
Balaenoptera musculus				3132	1984.5	2182	2192	2139	2113
Didelphis virginiana					3100	1937	1971	1916.5	1975
Pan troglodytes						3085	2943	2810	1969
Pan paniscus							3088	2819	1998
Gorilla gorilla								3086	1979
Mus musculus									3115

Table A13. The parameter Identity of the BLAST in the quantitative measurement of homology, assuming the *Nine ND5 protein sequences dataset* [3].

	Homo sapiens	Rattus norvegicus	Balaenoptera physalus	Balaenoptera musculus	Didelphis virginiana	Pan troglodytes	Pan paniscus	Gorilla gorilla	Mus musculus
Homo sapiens	100	63.1841	68.325	68.1592	61.9601	93.3665	93.2007	90.0498	63.5158
Rattus norvegicus		100	65.6766	65.5116	60.6312	62.6866	63.1841	62.5207	78.2537
Balaenoptera physalus			100	96.5347	61.1296	68.6567	68.8226	67.33	65.3465
Balaenoptera musculus				100	60.9635	68.6567	68.8226	67.4959	65.6766
Didelphis virginiana					100	61.4618	62.1262	60.4651	62.2924
Pan troglodytes						100	95.0249	90.7131	63.1841
Pan paniscus							100	90.8789	63.5158
Gorilla gorilla								100	63.0182
Mus musculus									100

Table A14. The parameter Similarity of the BLAST in the quantitative measurement of homology, assuming the *Nine ND5 protein sequences dataset* [3].

	Homo sapiens	Rattus norvegicus	Balaenoptera physalus	Balaenoptera musculus	Didelphis virginiana	Pan troglodytes	Pan paniscus	Gorilla gorilla	Mus musculus
Homo sapiens	100	72.8027	77.7778	77.1144	72.7575	95.1907	95.8541	92.2056	73.7977
Rattus norvegicus		100	77.2277	76.7327	71.0963	72.9685	72.9685	74.1294	85.0082
Balaenoptera physalus			100	97.0297	76.412	77.6119	77.7778	76.7828	77.7228
Balaenoptera musculus				100	75.4153	76.9486	77.1144	76.1194	77.2277
Didelphis virginiana					100	73.0897	73.7542	72.093	72.7575
Pan troglodytes						100	96.5174	93.2007	73.7977
Pan paniscus							100	93.864	73.466
Gorilla gorilla								100	74.7927
Mus musculus									100

Table A15. The parameter Alignment Score of the BLAST in the quantitative measurement of homology, assuming the *Nine ND5 protein sequences dataset* [3].

	Homo sapiens	Rattus norvegicus	Balaenoptera physalus	Balaenoptera musculus	Didelphis virginiana	Pan troglodytes	Pan paniscus	Gorilla gorilla	Mus musculus
Homo sapiens	3091	1906	2161	2154	1903.5	2884	2889	2788	1932.5
Rattus norvegicus		3139	2059	2059	1868.5	1894	1912	1889	2474
Balaenoptera physalus			3125	3020	1959.5	2180	2185	2131	2078
Balaenoptera musculus				3132	1942.5	2171	2179	2128	2089
Didelphis virginiana					3100	1887.5	1917.5	1857.5	1959
Pan troglodytes						3085	2942	2810	1935.5
Pan paniscus							3088	2818	1945.5
Gorilla gorilla								3086	1939.5
Mus musculus									3115

Table A16. The parameter Identity of the ClustalW in the quantitative measurement of homology, assuming the *Nine ND5 protein sequences dataset* [3].

	Homo sapiens	Rattus norvegicus	Balaenoptera physalus	Balaenoptera musculus	Didelphis virginiana	Pan troglodytes	Pan paniscus	Gorilla gorilla	Mus musculus
Homo sapiens	100	63.1841	68.325	68.1592	62.2924	93.3665	93.2007	90.0498	63.8474
Rattus norvegicus		100	65.5116	65.6766	60.4651	62.8524	63.1841	62.5207	78.2537
Balaenoptera physalus			100	96.5347	60.9635	68.6567	68.8226	67.33	64.6865
Balaenoptera musculus				100	60.9635	68.6567	68.8226	67.4959	65.1815
Didelphis virginiana					100	61.794	62.6246	60.4651	61.9601
Pan troglodytes						100	95.0249	90.7131	63.5158
Pan paniscus							100	90.8789	63.8474
Gorilla gorilla								100	63.3499
Mus musculus									100

Table A17. The parameter Similarity of the ClustalW in the quantitative measurement of homology, assuming the *Nine ND5 protein sequences dataset* [3].

	Homo sapiens	Rattus norvegicus	Balaenoptera physalus	Balaenoptera musculus	Didelphis virginiana	Pan troglodytes	Pan paniscus	Gorilla gorilla	Mus musculus
Homo sapiens	100	72.471	77.7778	77.1144	72.9236	95.1907	95.8541	92.2056	73.9635
Rattus norvegicus		100	77.0627	76.7327	70.9302	72.8027	72.6368	74.1294	85.0082
Balaenoptera physalus			100	97.0297	75.9136	77.6119	77.7778	76.7828	76.8977
Balaenoptera musculus				100	75.0831	76.9486	77.1144	76.1194	77.2277
Didelphis virginiana					100	72.9236	73.9203	71.9269	71.7608
Pan troglodytes						100	96.5174	93.2007	74.2952
Pan paniscus							100	93.864	73.6318
Gorilla gorilla								100	74.9585
Mus musculus									100

Table A18. The parameter Alignment Score of the ClustalW in the quantitative measurement of homology, assuming the *Nine ND5 protein sequences dataset* [3].

	Homo sapiens	Rattus norvegicus	Balaenoptera physalus	Balaenoptera musculus	Didelphis virginiana	Pan troglodytes	Pan paniscus	Gorilla gorilla	Mus musculus
Homo sapiens	3091	1921	2161	2154	1932.5	2884	2889	2788	1963.5
Rattus norvegicus		3139	2069.5	2082.5	1876	1913	1925	1903	2474
Balaenoptera physalus			3125	3020	1968	2180	2185	2131	2066
Balaenoptera musculus				3132	1963	2171	2179	2128	2092
Didelphis virginiana					3100	1916.5	1952.5	1877.5	1942.5
Pan troglodytes						3085	2942	2810	1964.5
Pan paniscus							3088	2818	1975.5
Gorilla gorilla								3086	1968.5
Mus musculus									3115

Table A19. The parameter Identity of the ClustalΩ in the quantitative measurement of homology, assuming the *Nine ND5 protein sequences dataset* [3].

	Homo sapiens	Rattus norvegicus	Balaenoptera physalus	Balaenoptera musculus	Didelphis virginiana	Pan troglodytes	Pan paniscus	Gorilla gorilla	Mus musculus
Homo sapiens	100	63.1841	68.325	68.1592	62.2924	93.3665	93.2007	90.0498	63.8474
Rattus norvegicus		100	65.5116	65.6766	60.1329	62.8524	63.1841	62.5207	78.2537
Balaenoptera physalus			100	96.5347	60.7973	68.6567	68.8226	67.33	64.6865
Balaenoptera musculus				100	60.6312	68.6567	68.8226	67.4959	65.1815
Didelphis virginiana					100	61.6279	62.4585	60.4651	61.794
Pan troglodytes						100	95.0249	90.7131	63.5158
Pan paniscus							100	90.8789	63.8474
Gorilla gorilla								100	63.3499
Mus musculus									100

Table A20. The parameter Similarity of the ClustalΩ in the quantitative measurement of homology, assuming the *Nine ND5 protein sequences dataset* [3].

	Homo sapiens	Rattus norvegicus	Balaenoptera physalus	Balaenoptera musculus	Didelphis virginiana	Pan troglodytes	Pan paniscus	Gorilla gorilla	Mus musculus
Homo sapiens	100	72.471	77.7778	77.1144	72.9236	95.1907	95.8541	92.2056	73.9635
Rattus norvegicus		100	77.0627	76.7327	70.7641	72.8027	72.6368	74.1294	85.0082
Balaenoptera physalus			100	97.0297	75.9136	77.6119	77.7778	76.7828	76.8977
Balaenoptera musculus				100	74.9169	76.9486	77.1144	76.1194	77.2277
Didelphis virginiana					100	72.9236	73.9203	72.093	72.4252
Pan troglodytes						100	96.5174	93.2007	74.2952
Pan paniscus							100	93.864	73.6318
Gorilla gorilla								100	74.9585
Mus musculus									100

Table A21. The parameter Alignment Score of the ClustalΩ in the quantitative measurement of homology, assuming the *Nine ND5 protein sequences dataset* [3].

	Homo sapiens	Rattus norvegicus	Balaenoptera physalus	Balaenoptera musculus	Didelphis virginiana	Pan troglodytes	Pan paniscus	Gorilla gorilla	Mus musculus
Homo sapiens	3091	1921	2161	2154	1928.5	2884	2889	2788	1963.5
Rattus norvegicus		3139	2069.5	2082.5	1860	1913	1925	1903	2474
Balaenoptera physalus			3125	3020	1967	2180	2185	2131	2066
Balaenoptera musculus				3132	1957	2171	2179	2128	2092
Didelphis virginiana					3100	1910.5	1946.5	1877.5	1949.5
Pan troglodytes						3085	2942	2810	1964.5
Pan paniscus							3088	2818	1975.5
Gorilla gorilla								3086	1968.5
Mus musculus									3115

Table A22. The parameter Identity of the MUSCLE in the quantitative measurement of homology, assuming the *Nine ND5 protein sequences dataset* [3].

	Homo sapiens	Rattus norvegicus	Balaenoptera physalus	Balaenoptera musculus	Didelphis virginiana	Pan troglodytes	Pan paniscus	Gorilla gorilla	Mus musculus
Homo sapiens	100	63.1841	68.325	68.1592	62.2924	93.3665	93.2007	90.0498	63.8474
Rattus norvegicus		100	65.5116	65.6766	60.4651	62.8524	63.1841	62.5207	78.2537
Balaenoptera physalus			100	96.5347	60.9635	68.6567	68.8226	67.33	64.6865
Balaenoptera musculus				100	60.9635	68.6567	68.8226	67.4959	65.1815
Didelphis virginiana					100	61.794	62.6246	60.4651	61.9601
Pan troglodytes						100	95.0249	90.7131	63.5158
Pan paniscus							100	90.8789	63.8474
Gorilla gorilla								100	63.3499
Mus musculus									100

Table A23. The parameter Similarity of the MUSCLE in the quantitative measurement of homology, assuming the *Nine ND5 protein sequences dataset* [3].

	Homo sapiens	Rattus norvegicus	Balaenoptera physalus	Balaenoptera musculus	Didelphis virginiana	Pan troglodytes	Pan paniscus	Gorilla gorilla	Mus musculus
Homo sapiens	100	72.471	77.7778	77.1144	73.0897	95.1907	95.8541	92.2056	73.9635
Rattus norvegicus		100	77.0627	76.7327	71.0963	72.8027	72.6368	74.1294	85.0082
Balaenoptera physalus			100	97.0297	76.0797	77.6119	77.7778	76.7828	76.8977
Balaenoptera musculus				100	75.2492	76.9486	77.1144	76.1194	77.2277
Didelphis virginiana					100	73.0897	74.0864	72.093	71.9269
Pan troglodytes						100	96.5174	93.2007	74.2952
Pan paniscus							100	93.864	73.6318
Gorilla gorilla								100	74.9585
Mus musculus									100

Table A24. The parameter Alignment Score of the MUSCLE in the quantitative measurement of homology, assuming the *Nine ND5 protein sequences dataset* [3].

	Homo sapiens	Rattus norvegicus	Balaenoptera physalus	Balaenoptera musculus	Didelphis virginiana	Pan troglodytes	Pan paniscus	Gorilla gorilla	Mus musculus
Homo sapiens	3091	1921	2161	2154	1934.5	2884	2889	2788	1963.5
Rattus norvegicus		3139	2069.5	2082.5	1874	1913	1925	1903	2474
Balaenoptera physalus			3125	3020	1970	2180	2185	2131	2066
Balaenoptera musculus				3132	1965	2171	2179	2128	2092
Didelphis virginiana					3100	1918.5	1954.5	1879.5	1944.5
Pan troglodytes						3085	2942	2810	1964.5
Pan paniscus							3088	2818	1975.5
Gorilla gorilla								3086	1968.5
Mus musculus									3115

Table A25. The parameter Identity of the T-Coffee in the quantitative measurement of homology, assuming the *Nine ND5 protein sequences dataset* [3].

	Homo sapiens	Rattus norvegicus	Balaenoptera physalus	Balaenoptera musculus	Didelphis virginiana	Pan troglodytes	Pan paniscus	Gorilla gorilla	Mus musculus
Homo sapiens	100	63.0182	68.325	68.1592	62.7907	93.3665	93.2007	90.0498	63.8474
Rattus norvegicus		100	65.8416	66.0066	60.6312	62.6866	63.1841	62.3549	78.2537
Balaenoptera physalus			100	96.5347	60.9635	68.6567	68.8226	67.33	64.8515
Balaenoptera musculus				100	60.9635	68.6567	68.8226	67.4959	65.3465
Didelphis virginiana					100	62.1262	62.7907	60.9635	62.1262
Pan troglodytes						100	95.0249	90.7131	63.5158
Pan paniscus							100	90.8789	63.8474
Gorilla gorilla								100	63.3499
Mus musculus									100

Table A26. The parameter Similarity of the T-Coffee in the quantitative measurement of homology, assuming the *Nine ND5 protein sequences dataset* [3].

	Homo sapiens	Rattus norvegicus	Balaenoptera physalus	Balaenoptera musculus	Didelphis virginiana	Pan troglodytes	Pan paniscus	Gorilla gorilla	Mus musculus
Homo sapiens	100	72.3051	77.7778	77.1144	73.588	95.1907	95.8541	92.2056	73.7977
Rattus norvegicus		100	77.3927	77.0627	70.9302	72.6368	72.471	73.9635	85.0082
Balaenoptera physalus			100	97.0297	75.7475	77.6119	77.7778	76.7828	77.0627
Balaenoptera musculus				100	74.9169	76.9486	77.1144	76.1194	77.3927
Didelphis virginiana					100	73.588	74.5847	72.7575	72.4252
Pan troglodytes						100	96.5174	93.2007	74.1294
Pan paniscus							100	93.864	73.466
Gorilla gorilla								100	74.7927
Mus musculus									100

Table A27. The parameter Alignment Score of the T-Coffee in the quantitative measurement of homology, assuming the *Nine ND5 protein sequences dataset* [3].

	Homo sapiens	Rattus norvegicus	Balaenoptera physalus	Balaenoptera musculus	Didelphis virginiana	Pan troglodytes	Pan paniscus	Gorilla gorilla	Mus musculus
Homo sapiens	3091	1905.5	2160	2153	1949.5	2884	2889	2788	1961.5
Rattus norvegicus		3139	2075	2088	1850	1897.5	1914.5	1887.5	2474
Balaenoptera physalus			3125	3020	1957.5	2179	2185	2130	2073
Balaenoptera musculus				3132	1952.5	2170	2179	2127	2099
Didelphis virginiana					3100	1931.5	1961.5	1898.5	1952
Pan troglodytes						3085	2942	2810	1962.5
Pan paniscus							3088	2818	1974.5
Gorilla gorilla								3086	1966.5
Mus musculus									3115

Table A28. The parameter Identity of the Kalign in the quantitative measurement of homology, assuming the *Nine ND5 protein sequences dataset* [3].

	Homo sapiens	Rattus norvegicus	Balaenoptera physalus	Balaenoptera musculus	Didelphis virginiana	Pan troglodytes	Pan paniscus	Gorilla gorilla	Mus musculus
Homo sapiens	100	63.1841	68.325	68.1592	62.4585	93.3665	93.2007	90.0498	63.8474
Rattus norvegicus		100	65.5116	65.6766	60.7973	62.8524	63.1841	62.5207	78.2537
Balaenoptera physalus			100	96.5347	61.2957	68.6567	68.8226	67.33	64.6865
Balaenoptera musculus				100	61.2957	68.6567	68.8226	67.4959	65.1815
Didelphis virginiana					100	61.9601	62.6246	60.6312	62.4585
Pan troglodytes						100	95.0249	90.7131	63.5158
Pan paniscus							100	90.8789	63.8474
Gorilla gorilla								100	63.3499
Mus musculus									100

Table A29. The parameter Similarity of the Kalign in the quantitative measurement of homology, assuming the *Nine ND5 protein sequences dataset* [3].

	Homo sapiens	Rattus norvegicus	Balaenoptera physalus	Balaenoptera musculus	Didelphis virginiana	Pan troglodytes	Pan paniscus	Gorilla gorilla	Mus musculus
Homo sapiens	100	72.471	77.7778	77.1144	73.4219	95.1907	95.8541	92.2056	73.9635
Rattus norvegicus		100	77.0627	76.7327	71.0963	72.8027	72.6368	74.1294	85.0082
Balaenoptera physalus			100	97.0297	76.0797	77.6119	77.7778	76.7828	76.8977
Balaenoptera musculus				100	75.2492	76.9486	77.1144	76.1194	77.2277
Didelphis virginiana					100	73.4219	74.4186	72.4252	72.7575
Pan troglodytes						100	96.5174	93.2007	74.2952
Pan paniscus							100	93.864	73.6318
Gorilla gorilla								100	74.9585
Mus musculus									100

Table A30. The parameter Alignment Score of the Kalign in the quantitative measurement of homology, assuming the *Nine ND5 protein sequences dataset* [3].

	Homo sapiens	Rattus norvegicus	Balaenoptera physalus	Balaenoptera musculus	Didelphis virginiana	Pan troglodytes	Pan paniscus	Gorilla gorilla	Mus musculus
Homo sapiens	3091	1921	2161	2154	1943.5	2884	2889	2788	1963.5
Rattus norvegicus		3139	2069.5	2082.5	1870.5	1913	1925	1903	2474
Balaenoptera physalus			3125	3020	1971.5	2180	2185	2131	2066
Balaenoptera musculus				3132	1966.5	2171	2179	2128	2092
Didelphis virginiana					3100	1927.5	1957.5	1889.5	1961
Pan troglodytes						3085	2942	2810	1964.5
Pan paniscus							3088	2818	1975.5
Gorilla gorilla								3086	1968.5
Mus musculus									3115

Table A31. The parameter Identity of the MAFFT in the quantitative measurement of homology, assuming the *Nine ND5 protein sequences dataset* [3].

	Homo sapiens	Rattus norvegicus	Balaenoptera physalus	Balaenoptera musculus	Didelphis virginiana	Pan troglodytes	Pan paniscus	Gorilla gorilla	Mus musculus
Homo sapiens	100	63.1841	68.325	68.1592	62.6246	93.3665	93.2007	90.0498	63.8474
Rattus norvegicus		100	65.8416	66.0066	60.9635	62.8524	63.1841	62.5207	78.2537
Balaenoptera physalus			100	96.5347	61.4618	68.6567	68.8226	67.33	64.8515
Balaenoptera musculus				100	61.4618	68.6567	68.8226	67.4959	65.3465
Didelphis virginiana					100	62.1262	62.7907	60.7973	62.6246
Pan troglodytes						100	95.0249	90.7131	63.5158
Pan paniscus							100	90.8789	63.8474
Gorilla gorilla								100	63.3499
Mus musculus									100

Table A32. The parameter Similarity of the MAFFT in the quantitative measurement of homology, assuming the *Nine ND5 protein sequences dataset* [3].

	Homo sapiens	Rattus norvegicus	Balaenoptera physalus	Balaenoptera musculus	Didelphis virginiana	Pan troglodytes	Pan paniscus	Gorilla gorilla	Mus musculus
Homo sapiens	100	72.471	77.7778	77.1144	73.588	95.1907	95.8541	92.2056	73.9635
Rattus norvegicus		100	77.3927	77.0627	71.2625	72.8027	72.6368	74.1294	85.0082
Balaenoptera physalus			100	97.0297	76.2458	77.6119	77.7778	76.7828	77.0627
Balaenoptera musculus				100	75.4153	76.9486	77.1144	76.1194	77.3927
Didelphis virginiana					100	73.588	74.5847	72.5914	72.9236
Pan troglodytes						100	96.5174	93.2007	74.2952
Pan paniscus							100	93.864	73.6318
Gorilla gorilla								100	74.9585
Mus musculus									100

Table A33. The parameter Alignment Score of the MAFFT in the quantitative measurement of homology, assuming the *Nine ND5 protein sequences dataset* [3].

	Homo sapiens	Rattus norvegicus	Balaenoptera physalus	Balaenoptera musculus	Didelphis virginiana	Pan troglodytes	Pan paniscus	Gorilla gorilla	Mus musculus
Homo sapiens	3091	1921	2161	2154	1947.5	2884	2889	2788	1963.5
Rattus norvegicus		3139	2075	2088	1874.5	1913	1925	1903	2474
Balaenoptera physalus			3125	3020	1975.5	2180	2185	2131	2073
Balaenoptera musculus				3132	1970.5	2171	2179	2128	2099
Didelphis virginiana					3100	1931.5	1961.5	1893.5	1965
Pan troglodytes						3085	2942	2810	1964.5
Pan paniscus							3088	2818	1975.5
Gorilla gorilla								3086	1968.5
Mus musculus									3115

Table A34. The parameter Sensitivity (SEN) of the HELIOS method with referencing the Smith-Waterman in the accuracy measurement of classification output, assuming the *Nine ND5 protein sequences dataset* [3].

	Homo sapiens	Rattus norvegicus	Balaenoptera physalus	Balaenoptera musculus	Didelphis virginiana	Pan troglodytes	Pan paniscus	Gorilla gorilla	Mus musculus
Homo sapiens	1	0.93012	0.88372	0.92359	0.91457	1	1	1	0.94324
Rattus norvegicus		1	0.93377	0.96026	0.86957	0.9103	0.92206	0.89535	0.9654
Balaenoptera physalus			1	1	0.89244	0.9204	0.90713	0.9204	0.90381
Balaenoptera musculus				1	0.88926	0.92703	0.93201	0.92869	0.95207
Didelphis virginiana					1	0.90285	0.95477	0.93467	0.93121
Pan troglodytes						1	1	1	0.91833
Pan paniscus							1	0.99337	0.93023
Gorilla gorilla								1	0.94676
Mus musculus									1

Table A35. The parameter Specification (Spec) of the HELIOS method with referencing the Smith-Waterman in the accuracy measurement of classification output, assuming the *Nine ND5 protein sequences dataset* [3].

	Homo sapiens	Rattus norvegicus	Balaenoptera physalus	Balaenoptera musculus	Didelphis virginiana	Pan troglodytes	Pan paniscus	Gorilla gorilla	Mus musculus
Homo sapiens	1	0.99989	0.99987	0.99991	0.99988	1	1	1	0.99992
Rattus norvegicus		1	0.99991	0.99994	0.99981	0.9999	0.9999	0.99987	0.99996
Balaenoptera physalus			1	1	0.99983	0.99991	0.9999	0.99992	0.99986
Balaenoptera musculus				1	0.99983	0.99993	0.99993	0.99993	0.99995
Didelphis virginiana					1	0.99987	0.99993	0.99991	0.99992
Pan troglodytes						1	1	1	0.99989
Pan paniscus							1	0.99999	0.99992
Gorilla gorilla								1	0.99993
Mus musculus									1

Table A36. The parameter Accuracy (Acc) of the HELIOS method with referencing the Smith-Waterman in the accuracy measurement of classification output, assuming the *Nine ND5 protein sequences dataset* [3].

	Homo sapiens	Rattus norvegicus	Balaenoptera physalus	Balaenoptera musculus	Didelphis virginiana	Pan troglodytes	Pan paniscus	Gorilla gorilla	Mus musculus
Homo sapiens	1	0.99978	0.99968	0.99979	0.99974	1	1	1	0.99983
Rattus norvegicus		1	0.9998	0.99988	0.9996	0.99975	0.99977	0.9997	0.99991
Balaenoptera physalus			1	1	0.99965	0.99978	0.99975	0.99978	0.99971
Balaenoptera musculus				1	0.99965	0.99981	0.99982	0.99981	0.99987
Didelphis virginiana					1	0.99971	0.99986	0.99981	0.99981
Pan troglodytes						1	1	1	0.99976
Pan paniscus							1	0.99998	0.9998
Gorilla gorilla								1	0.99984
Mus musculus									1

Table A37. The parameter Positive Predictive Value (PPV) of the HELIOS method with referencing the Smith-Waterman in the accuracy measurement of classification output, assuming the *Nine ND5 protein sequences dataset* [3].

	Homo sapiens	Rattus norvegicus	Balaenoptera physalus	Balaenoptera musculus	Didelphis virginiana	Pan troglodytes	Pan paniscus	Gorilla gorilla	Mus musculus
Homo sapiens	1	0.93478	0.91883	0.94558	0.92386	1	1	1	0.95118
Rattus norvegicus		1	0.94472	0.96506	0.88435	0.93675	0.93919	0.9198	0.9783
Balaenoptera physalus			1	1	0.89545	0.94549	0.93825	0.9471	0.91597
Balaenoptera musculus				1	0.89527	0.95556	0.95904	0.954	0.96807
Didelphis virginiana					1	0.91823	0.9596	0.94737	0.94872
Pan troglodytes						1	1	1	0.93232
Pan paniscus							1	0.99667	0.94755
Gorilla gorilla								1	0.95791
Mus musculus									1

Table A38. The parameter Negative Predictive Value (NPV) of the HELIOS method with referencing the Smith-Waterman in the accuracy measurement of classification output, assuming the *Nine ND5 protein sequences dataset* [3].

	Homo sapiens	Rattus norvegicus	Balaenoptera physalus	Balaenoptera musculus	Didelphis virginiana	Pan troglodytes	Pan paniscus	Gorilla gorilla	Mus musculus
Homo sapiens	1	0.99989	0.99981	0.99987	0.99986	1	1	1	0.99991
Rattus norvegicus		1	0.99989	0.99993	0.99979	0.99985	0.99987	0.99983	0.99994
Balaenoptera physalus			1	1	0.99982	0.99987	0.99985	0.99987	0.99984
Balaenoptera musculus				1	0.99982	0.99988	0.99989	0.99988	0.99992
Didelphis virginiana					1	0.99984	0.99993	0.99989	0.99989
Pan troglodytes						1	1	1	0.99987
Pan paniscus							1	0.99999	0.99989
Gorilla gorilla								1	0.99991
Mus musculus									1

Table A39. The parameter Matthew's Coefficient Correlation (MCC) of the HELIOS method with referencing the Smith-Waterman in the accuracy measurement of classification output, assuming the *Nine ND5 protein sequences dataset* [3].

	Homo sapiens	Rattus norvegicus	Balaenoptera physalus	Balaenoptera musculus	Didelphis virginiana	Pan troglodytes	Pan paniscus	Gorilla gorilla	Mus musculus
Homo sapiens	1	0.93234	0.90094	0.93441	0.91907	1	1	1	0.94711
Rattus norvegicus		1	0.93913	0.9626	0.87673	0.92331	0.93047	0.90734	0.97178
Balaenoptera physalus			1	1	0.89377	0.93275	0.92243	0.93355	0.90972
Balaenoptera musculus				1	0.89209	0.94109	0.94534	0.94117	0.95997
Didelphis virginiana					1	0.91036	0.95711	0.9409	0.93983
Pan troglodytes						1	1	1	0.92518
Pan paniscus							1	0.99501	0.93875
Gorilla gorilla								1	0.95224
Mus musculus									1

Table A40. The parameter Test's Accuracy (F-Score) of the HELIOS method with referencing the Smith-Waterman in the accuracy measurement of classification output, assuming the *Nine ND5 protein sequences dataset* [3].

	Homo sapiens	Rattus norvegicus	Balaenoptera physalus	Balaenoptera musculus	Didelphis virginiana	Pan troglodytes	Pan paniscus	Gorilla gorilla	Mus musculus
Homo sapiens	1	0.93244	0.90093	0.93445	0.91919	1	1	1	0.94719
Rattus norvegicus		1	0.93922	0.96266	0.8769	0.92334	0.93054	0.90741	0.97181
Balaenoptera physalus			1	1	0.89394	0.93277	0.92243	0.93356	0.90985
Balaenoptera musculus				1	0.89226	0.94108	0.94533	0.94118	0.96
Didelphis virginiana					1	0.91047	0.95718	0.94098	0.93988
Pan troglodytes						1	1	1	0.92527
Pan paniscus							1	0.99502	0.93881
Gorilla gorilla								1	0.9523
Mus musculus									1

Table A41. The parameter Sensitivity (SEN) of the HELIOS method with referencing the Needleman-Wunsch in the accuracy measurement of classification output, assuming the *Nine ND5 protein sequences dataset* [3].

	Homo sapiens	Rattus norvegicus	Balaenoptera physalus	Balaenoptera musculus	Didelphis virginiana	Pan troglodytes	Pan paniscus	Gorilla gorilla	Mus musculus
Homo sapiens	1	0.93012	0.88667	0.92667	0.020134	1	1	1	0.94324
Rattus norvegicus		1	0.93377	0.96026	0.028428	0.9103	0.92512	0.89535	0.9654
Balaenoptera physalus			1	1	0.026891	0.92346	0.91015	0.92346	0.90833
Balaenoptera musculus				1	0.02521	0.93012	0.93511	0.93178	0.95349
Didelphis virginiana					1	0.018456	0.0083893	0.015101	0.018487
Pan troglodytes						1	1	1	0.91833
Pan paniscus							1	0.99336	0.93
Gorilla gorilla								1	0.94676
Mus musculus									1

Table A42. The parameter Specification (Spec) of the HELIOS method with referencing the Needleman-Wunsch in the accuracy measurement of classification output, assuming the *Nine ND5 protein sequences dataset* [3].

	Homo sapiens	Rattus norvegicus	Balaenoptera physalus	Balaenoptera musculus	Didelphis virginiana	Pan troglodytes	Pan paniscus	Gorilla gorilla	Mus musculus
Homo sapiens	1	0.99989	0.99988	0.99992	0.9984	1	1	1	0.99992
Rattus norvegicus		1	0.99991	0.99994	0.99842	0.9999	0.99991	0.99987	0.99996
Balaenoptera physalus			1	1	0.9984	0.99992	0.99991	0.99993	0.99987
Balaenoptera musculus				1	0.9984	0.99994	0.99994	0.99994	0.99995
Didelphis virginiana					1	0.99841	0.99837	0.99839	0.99841
Pan troglodytes						1	1	1	0.9999
Pan paniscus							1	0.99999	0.99991
Gorilla gorilla								1	0.99993
Mus musculus									1

Table A43. The parameter Accuracy (Acc) of the HELIOS method with referencing the Needleman-Wunsch in the accuracy measurement of classification output, assuming the *Nine ND5 protein sequences dataset* [3].

	Homo sapiens	Rattus norvegicus	Balaenoptera physalus	Balaenoptera musculus	Didelphis virginiana	Pan troglodytes	Pan paniscus	Gorilla gorilla	Mus musculus
Homo sapiens	1	0.99978	0.99969	0.9998	0.99678	1	1	1	0.99983
Rattus norvegicus		1	0.9998	0.99988	0.99682	0.99975	0.99979	0.9997	0.99991
Balaenoptera physalus			1	1	0.99679	0.9998	0.99976	0.9998	0.99972
Balaenoptera musculus				1	0.99679	0.99982	0.99983	0.99982	0.99987
Didelphis virginiana					1	0.99678	0.99673	0.99676	0.99679
Pan troglodytes						1	1	1	0.99977
Pan paniscus							1	0.99998	0.9998
Gorilla gorilla								1	0.99984
Mus musculus									1

Table A44. The parameter Positive Predictive Value (PPV) of the HELIOS method with referencing the Needleman-Wunsch in the accuracy measurement of classification output, assuming the *Nine ND5 protein sequences dataset* [3].

	Homo sapiens	Rattus norvegicus	Balaenoptera physalus	Balaenoptera musculus	Didelphis virginiana	Pan troglodytes	Pan paniscus	Gorilla gorilla	Mus musculus
Homo sapiens	1	0.93478	0.92522	0.95205	0.020443	1	1	1	0.95118
Rattus norvegicus		1	0.94472	0.96506	0.02906	0.93675	0.94558	0.9198	0.9783
Balaenoptera physalus			1	1	0.027073	0.95197	0.94148	0.95361	0.92217
Balaenoptera musculus				1	0.025467	0.96213	0.96233	0.96055	0.96959
Didelphis virginiana					1	0.018868	0.0084746	0.015385	0.018933
Pan troglodytes						1	1	1	0.93867
Pan paniscus							1	0.99667	0.94737
Gorilla gorilla								1	0.95791
Mus musculus									1

Table A45. The parameter Negative Predictive Value (NPV) of the HELIOS method with referencing the Needleman-Wunsch in the accuracy measurement of classification output, assuming the *Nine ND5 protein sequences dataset* [3].

	Homo sapiens	Rattus norvegicus	Balaenoptera physalus	Balaenoptera musculus	Didelphis virginiana	Pan troglodytes	Pan paniscus	Gorilla gorilla	Mus musculus
Homo sapiens	1	0.99989	0.99981	0.99988	0.99837	1	1	1	0.99991
Rattus norvegicus		1	0.99989	0.99993	0.99839	0.99985	0.99988	0.99983	0.99994
Balaenoptera physalus			1	1	0.99839	0.99987	0.99985	0.99987	0.99985
Balaenoptera musculus				1	0.99839	0.99988	0.99989	0.99989	0.99992
Didelphis virginiana					1	0.99837	0.99835	0.99836	0.99837
Pan troglodytes						1	1	1	0.99987
Pan paniscus							1	0.99999	0.99988
Gorilla gorilla								1	0.99991
Mus musculus									1

Table A46. The parameter Matthew's Coefficient Correlation (MCC) of the HELIOS method with referencing the Needleman-Wunsch in the accuracy measurement of classification output, assuming the *Nine ND5 protein sequences dataset* [3].

	Homo sapiens	Rattus norvegicus	Balaenoptera physalus	Balaenoptera musculus	Didelphis virginiana	Pan troglodytes	Pan paniscus	Gorilla gorilla	Mus musculus
Homo sapiens	1	0.93234	0.90558	0.93918	0.018673	1	1	1	0.94711
Rattus norvegicus		1	0.93913	0.9626	0.027149	0.92331	0.93519	0.90734	0.97178
Balaenoptera physalus			1	1	0.025376	0.93751	0.92556	0.93831	0.91508
Balaenoptera musculus				1	0.023733	0.9459	0.94854	0.94597	0.96144
Didelphis virginiana					1	0.017049	0.0067931	0.013621	0.017101
Pan troglodytes						1	1	1	0.92833
Pan paniscus							1	0.995	0.93854
Gorilla gorilla								1	0.95224
Mus musculus									1

Table A47. The parameter Test's Accuracy (F-Score) of the HELIOS method with referencing the Needleman-Wunsch in the accuracy measurement of classification output, assuming the *Nine ND5 protein sequences dataset* [3].

	Homo sapiens	Rattus norvegicus	Balaenoptera physalus	Balaenoptera musculus	Didelphis virginiana	Pan troglodytes	Pan paniscus	Gorilla gorilla	Mus musculus
Homo sapiens	1	0.93244	0.90553	0.93919	0.020287	1	1	1	0.94719
Rattus norvegicus		1	0.93922	0.96266	0.02874	0.92334	0.93524	0.90741	0.97181
Balaenoptera physalus			1	1	0.026981	0.9375	0.92555	0.93829	0.9152
Balaenoptera musculus				1	0.025338	0.94585	0.94852	0.94595	0.96147
Didelphis virginiana					1	0.01866	0.0084317	0.015241	0.018707
Pan troglodytes						1	1	1	0.92839
Pan paniscus							1	0.99501	0.9386
Gorilla gorilla								1	0.9523
Mus musculus									1

Table A48. The parameter Sensitivity (SEN) of the HELIOS method with referencing the BLAST in the accuracy measurement of classification output, assuming the *Nine ND5 protein sequences dataset* [3].

	Homo sapiens	Rattus norvegicus	Balaenoptera physalus	Balaenoptera musculus	Didelphis virginiana	Pan troglodytes	Pan paniscus	Gorilla gorilla	Mus musculus
Homo sapiens	1	0.92679	0.88391	0.93035	0.93456	1	1	0.99668	0.95008
Rattus norvegicus		1	0.94868	0.95861	0.87625	0.90349	0.90682	0.90682	0.9654
Balaenoptera physalus			1	1	0.90436	0.9204	0.90713	0.9204	0.9255
Balaenoptera musculus				1	0.9094	0.92703	0.93201	0.92869	0.94702
Didelphis virginiana					1	0.92953	0.95302	0.90604	0.91137
Pan troglodytes						1	1	1	0.94176
Pan paniscus							1	0.99337	0.9401
Gorilla gorilla								1	0.95008
Mus musculus									1

Table A49. The parameter Specification (Spec) of the HELIOS method with referencing the BLAST in the accuracy measurement of classification output, assuming the *Nine ND5 protein sequences dataset* [3].

	Homo sapiens	Rattus norvegicus	Balaenoptera physalus	Balaenoptera musculus	Didelphis virginiana	Pan troglodytes	Pan paniscus	Gorilla gorilla	Mus musculus
Homo sapiens	1	0.99989	0.99987	0.99993	0.99991	1	1	1	0.99994
Rattus norvegicus		1	0.99993	0.99994	0.99983	0.99989	0.99987	0.99989	0.99996
Balaenoptera physalus			1	1	0.99985	0.99991	0.9999	0.99992	0.9999
Balaenoptera musculus				1	0.99986	0.99993	0.99993	0.99993	0.99994
Didelphis virginiana					1	0.99991	0.99993	0.99986	0.99989
Pan troglodytes						1	1	1	0.99993
Pan paniscus							1	0.99999	0.99993
Gorilla gorilla								1	0.99994
Mus musculus									1

Table A50. The parameter Accuracy (Acc) of the HELIOS method with referencing the BLAST in the accuracy measurement of classification output, assuming the *Nine ND5 protein sequences dataset* [3].

	Homo sapiens	Rattus norvegicus	Balaenoptera physalus	Balaenoptera musculus	Didelphis virginiana	Pan troglodytes	Pan paniscus	Gorilla gorilla	Mus musculus
Homo sapiens	1	0.99977	0.99968	0.99981	0.9998	1	1	0.99999	0.99986
Rattus norvegicus		1	0.99985	0.99987	0.99962	0.99973	0.99972	0.99974	0.99991
Balaenoptera physalus			1	1	0.99969	0.99978	0.99975	0.99978	0.99978
Balaenoptera musculus				1	0.99971	0.99981	0.99982	0.99981	0.99985
Didelphis virginiana					1	0.99979	0.99985	0.99971	0.99975
Pan troglodytes						1	1	1	0.99984
Pan paniscus							1	0.99998	0.99983
Gorilla gorilla								1	0.99986
Mus musculus									1

Table A51. The parameter Positive Predictive Value (PPV) of the HELIOS method with referencing the BLAST in the accuracy measurement of classification output, assuming the *Nine ND5 protein sequences dataset* [3].

	Homo sapiens	Rattus norvegicus	Balaenoptera physalus	Balaenoptera musculus	Didelphis virginiana	Pan troglodytes	Pan paniscus	Gorilla gorilla	Mus musculus
Homo sapiens	1	0.93144	0.92055	0.95408	0.94247	1	1	0.99834	0.96128
Rattus norvegicus		1	0.9598	0.96339	0.89116	0.92821	0.92061	0.93003	0.9783
Balaenoptera physalus			1	1	0.90741	0.94549	0.93825	0.9471	0.9395
Balaenoptera musculus				1	0.91554	0.95556	0.95904	0.954	0.96134
Didelphis virginiana					1	0.94378	0.95623	0.91681	0.93162
Pan troglodytes						1	1	1	0.9577
Pan paniscus							1	0.99667	0.95601
Gorilla gorilla								1	0.96128
Mus musculus									1

Table A52. The parameter Negative Predictive Value (NPV) of the HELIOS method with referencing the BLAST in the accuracy measurement of classification output, assuming the *Nine ND5 protein sequences dataset* [3].

	Homo sapiens	Rattus norvegicus	Balaenoptera physalus	Balaenoptera musculus	Didelphis virginiana	Pan troglodytes	Pan paniscus	Gorilla gorilla	Mus musculus
Homo sapiens	1	0.99988	0.99981	0.99988	0.99989	1	1	0.99999	0.99992
Rattus norvegicus		1	0.99992	0.99993	0.9998	0.99984	0.99985	0.99985	0.99994
Balaenoptera physalus			1	1	0.99984	0.99987	0.99985	0.99987	0.99988
Balaenoptera musculus				1	0.99985	0.99988	0.99989	0.99988	0.99991
Didelphis virginiana					1	0.99988	0.99992	0.99985	0.99985
Pan troglodytes						1	1	1	0.9999
Pan paniscus							1	0.99999	0.9999
Gorilla gorilla								1	0.99992
Mus musculus									1

Table A53. The parameter Matthew's Coefficient Correlation (MCC) of the HELIOS method with referencing the BLAST in the accuracy measurement of classification output, assuming the *Nine ND5 protein sequences dataset* [3].

	Homo sapiens	Rattus norvegicus	Balaenoptera physalus	Balaenoptera musculus	Didelphis virginiana	Pan troglodytes	Pan paniscus	Gorilla gorilla	Mus musculus
Homo sapiens	1	0.92899	0.90189	0.94205	0.93841	1	1	0.99751	0.95559
Rattus norvegicus		1	0.95415	0.96094	0.88349	0.91563	0.91355	0.91822	0.97178
Balaenoptera physalus			1	1	0.90573	0.93275	0.92243	0.93355	0.93236
Balaenoptera musculus				1	0.91232	0.94109	0.94534	0.94117	0.95408
Didelphis virginiana					1	0.93653	0.95455	0.91126	0.92131
Pan troglodytes						1	1	1	0.94962
Pan paniscus							1	0.99501	0.94794
Gorilla gorilla								1	0.95559
Mus musculus									1

Table A54. The parameter Test's Accuracy (F-Score) of the HELIOS method with referencing the BLAST in the accuracy measurement of classification output, assuming the *Nine ND5 protein sequences dataset* [3].

	Homo sapiens	Rattus norvegicus	Balaenoptera physalus	Balaenoptera musculus	Didelphis virginiana	Pan troglodytes	Pan paniscus	Gorilla gorilla	Mus musculus
Homo sapiens	1	0.92911	0.90186	0.94207	0.9385	1	1	0.99751	0.95565
Rattus norvegicus		1	0.9542	0.961	0.88364	0.91568	0.91366	0.91828	0.97181
Balaenoptera physalus			1	1	0.90588	0.93277	0.92243	0.93356	0.93244
Balaenoptera musculus				1	0.91246	0.94108	0.94533	0.94118	0.95413
Didelphis virginiana					1	0.9366	0.95462	0.91139	0.92139
Pan troglodytes						1	1	1	0.94966
Pan paniscus							1	0.99502	0.94799
Gorilla gorilla								1	0.95565
Mus musculus									1

Table A55. The parameter Sensitivity (SEN) of the HELIOS method with referencing the ClustalW in the accuracy measurement of classification output, assuming the *Nine ND5 protein sequences dataset* [3].

	Homo sapiens	Rattus norvegicus	Balaenoptera physalus	Balaenoptera musculus	Didelphis virginiana	Pan troglodytes	Pan paniscus	Gorilla gorilla	Mus musculus
Homo sapiens	1	0.94196	0.88391	0.93035	0.93645	1	1	0.99668	0.96517
Rattus norvegicus		1	0.96205	0.97195	0.88462	0.91874	0.92206	0.90381	0.9654
Balaenoptera physalus			1	1	0.90301	0.9204	0.90713	0.9204	0.92574
Balaenoptera musculus				1	0.90803	0.92703	0.93201	0.92869	0.9538
Didelphis virginiana					1	0.92977	0.96488	0.90803	0.9097
Pan troglodytes						1	1	1	0.95025
Pan paniscus							1	0.99337	0.9403
Gorilla gorilla								1	0.96517
Mus musculus									1

Table A56. The parameter Specification (Spec) of the HELIOS method with referencing the ClustalW in the accuracy measurement of classification output, assuming the *Nine ND5 protein sequences dataset* [3].

	Homo sapiens	Rattus norvegicus	Balaenoptera physalus	Balaenoptera musculus	Didelphis virginiana	Pan troglodytes	Pan paniscus	Gorilla gorilla	Mus musculus
Homo sapiens	1	0.99992	0.99987	0.99993	0.99991	1	1	1	0.99997
Rattus norvegicus		1	0.99996	0.99997	0.99984	0.99992	0.9999	0.99989	0.99996
Balaenoptera physalus			1	1	0.99985	0.99991	0.9999	0.99992	0.99991
Balaenoptera musculus				1	0.99987	0.99993	0.99993	0.99993	0.99995
Didelphis virginiana					1	0.99991	0.99995	0.99987	0.99989
Pan troglodytes						1	1	1	0.99995
Pan paniscus							1	0.99999	0.99993
Gorilla gorilla								1	0.99997
Mus musculus									1

Table A57. The parameter Accuracy (Acc) of the HELIOS method with referencing the ClustalW in the accuracy measurement of classification output, assuming the *Nine ND5 protein sequences dataset* [3].

	Homo sapiens	Rattus norvegicus	Balaenoptera physalus	Balaenoptera musculus	Didelphis virginiana	Pan troglodytes	Pan paniscus	Gorilla gorilla	Mus musculus
Homo sapiens	1	0.99982	0.99968	0.99981	0.99981	1	1	0.99999	0.99991
Rattus norvegicus		1	0.9999	0.99992	0.99965	0.99978	0.99977	0.99973	0.99991
Balaenoptera physalus			1	1	0.99969	0.99978	0.99975	0.99978	0.99979
Balaenoptera musculus				1	0.99971	0.99981	0.99982	0.99981	0.99988
Didelphis virginiana					1	0.9998	0.9999	0.99972	0.99974
Pan troglodytes						1	1	1	0.99987
Pan paniscus							1	0.99998	0.99984
Gorilla gorilla								1	0.99991
Mus musculus									1

Table A58. The parameter Positive Predictive Value (PPV) of the HELIOS method with referencing the ClustalW in the accuracy measurement of classification output, assuming the *Nine ND5 protein sequences dataset* [3].

	Homo sapiens	Rattus norvegicus	Balaenoptera physalus	Balaenoptera musculus	Didelphis virginiana	Pan troglodytes	Pan paniscus	Gorilla gorilla	Mus musculus
Homo sapiens	1	0.94983	0.92055	0.95408	0.94755	1	1	0.99834	0.9798
Rattus norvegicus		1	0.97655	0.98003	0.89966	0.94701	0.93919	0.93003	0.9783
Balaenoptera physalus			1	1	0.90909	0.94549	0.93825	0.9471	0.94286
Balaenoptera musculus				1	0.91723	0.95556	0.95904	0.954	0.97143
Didelphis virginiana					1	0.94719	0.97138	0.9219	0.92991
Pan troglodytes						1	1	1	0.96954
Pan paniscus							1	0.99667	0.95939
Gorilla gorilla								1	0.9798
Mus musculus									1

Table A59. The parameter Negative Predictive Value (NPV) of the HELIOS method with referencing the ClustalW in the accuracy measurement of classification output, assuming the *Nine ND5 protein sequences dataset* [3].

	Homo sapiens	Rattus norvegicus	Balaenoptera physalus	Balaenoptera musculus	Didelphis virginiana	Pan troglodytes	Pan paniscus	Gorilla gorilla	Mus musculus
Homo sapiens	1	0.9999	0.99981	0.99988	0.9999	1	1	0.99999	0.99994
Rattus norvegicus		1	0.99994	0.99995	0.99981	0.99987	0.99987	0.99984	0.99994
Balaenoptera physalus			1	1	0.99984	0.99987	0.99985	0.99987	0.99988
Balaenoptera musculus				1	0.99985	0.99988	0.99989	0.99988	0.99992
Didelphis virginiana					1	0.99988	0.99994	0.99985	0.99985
Pan troglodytes						1	1	1	0.99992
Pan paniscus							1	0.99999	0.9999
Gorilla gorilla								1	0.99994
Mus musculus									1

Table A60. The parameter Matthew's Coefficient Correlation (MCC) of the HELIOS method with referencing the ClustalW in the accuracy measurement of classification output, assuming the *Nine ND5 protein sequences dataset* [3].

	Homo sapiens	Rattus norvegicus	Balaenoptera physalus	Balaenoptera musculus	Didelphis virginiana	Pan troglodytes	Pan paniscus	Gorilla gorilla	Mus musculus
Homo sapiens	1	0.9458	0.90189	0.94205	0.94189	1	1	0.99751	0.97241
Rattus norvegicus		1	0.96922	0.97594	0.89193	0.93266	0.93047	0.9167	0.97178
Balaenoptera physalus			1	1	0.90589	0.93275	0.92243	0.93355	0.93415
Balaenoptera musculus				1	0.91247	0.94109	0.94534	0.94117	0.96251
Didelphis virginiana					1	0.93834	0.96807	0.9148	0.91962
Pan troglodytes						1	1	1	0.95978
Pan paniscus							1	0.99501	0.94971
Gorilla gorilla								1	0.97241
Mus musculus									1

Table A61. The parameter Test's Accuracy (F-Score) of the HELIOS method with referencing the ClustalW in the accuracy measurement of classification output, assuming the *Nine ND5 protein sequences dataset* [3].

	Homo sapiens	Rattus norvegicus	Balaenoptera physalus	Balaenoptera musculus	Didelphis virginiana	Pan troglodytes	Pan paniscus	Gorilla gorilla	Mus musculus
Homo sapiens	1	0.94588	0.90186	0.94207	0.94197	1	1	0.99751	0.97243
Rattus norvegicus		1	0.96924	0.97597	0.89207	0.93266	0.93054	0.91674	0.97181
Balaenoptera physalus			1	1	0.90604	0.93277	0.92243	0.93356	0.93422
Balaenoptera musculus				1	0.91261	0.94108	0.94533	0.94118	0.96253
Didelphis virginiana					1	0.9384	0.96812	0.91491	0.9197
Pan troglodytes						1	1	1	0.9598
Pan paniscus							1	0.99502	0.94975
Gorilla gorilla								1	0.97243
Mus musculus									1

Table A62. The parameter Sensitivity (SEN) of the HELIOS method with referencing the ClustalΩ in the accuracy measurement of classification output, assuming the *Nine ND5 protein sequences dataset* [3].

	Homo sapiens	Rattus norvegicus	Balaenoptera physalus	Balaenoptera musculus	Didelphis virginiana	Pan troglodytes	Pan paniscus	Gorilla gorilla	Mus musculus
Homo sapiens	1	0.94196	0.88391	0.93035	0.93478	1	1	0.99668	0.96517
Rattus norvegicus		1	0.96205	0.97195	0.86957	0.91874	0.92206	0.90381	0.9654
Balaenoptera physalus			1	1	0.90803	0.9204	0.90713	0.9204	0.92574
Balaenoptera musculus				1	0.91304	0.92703	0.93201	0.92869	0.9538
Didelphis virginiana					1	0.92809	0.95987	0.91639	0.90803
Pan troglodytes						1	1	1	0.95025
Pan paniscus							1	0.99337	0.9403
Gorilla gorilla								1	0.96517
Mus musculus									1

Table A63. The parameter Specification (Spec) of the HELIOS method with referencing the ClustalΩ in the accuracy measurement of classification output, assuming the *Nine ND5 protein sequences dataset* [3].

	Homo sapiens	Rattus norvegicus	Balaenoptera physalus	Balaenoptera musculus	Didelphis virginiana	Pan troglodytes	Pan paniscus	Gorilla gorilla	Mus musculus
Homo sapiens	1	0.99992	0.99987	0.99993	0.99991	1	1	1	0.99997
Rattus norvegicus		1	0.99996	0.99997	0.99981	0.99992	0.9999	0.99989	0.99996
Balaenoptera physalus			1	1	0.99986	0.99991	0.9999	0.99992	0.99991
Balaenoptera musculus				1	0.99987	0.99993	0.99993	0.99993	0.99995
Didelphis virginiana					1	0.99991	0.99994	0.99989	0.99988
Pan troglodytes						1	1	1	0.99995
Pan paniscus							1	0.99999	0.99993
Gorilla gorilla								1	0.99997
Mus musculus									1

Table A64. The parameter Accuracy (Acc) of the HELIOS method with referencing the ClustalΩ in the accuracy measurement of classification output, assuming the *Nine ND5 protein sequences dataset* [3].

	Homo sapiens	Rattus norvegicus	Balaenoptera physalus	Balaenoptera musculus	Didelphis virginiana	Pan troglodytes	Pan paniscus	Gorilla gorilla	Mus musculus
Homo sapiens	1	0.99982	0.99968	0.99981	0.9998	1	1	0.99999	0.99991
Rattus norvegicus		1	0.9999	0.99992	0.9996	0.99978	0.99977	0.99973	0.99991
Balaenoptera physalus			1	1	0.99971	0.99978	0.99975	0.99978	0.99979
Balaenoptera musculus				1	0.99973	0.99981	0.99982	0.99981	0.99988
Didelphis virginiana					1	0.99979	0.99988	0.99975	0.99973
Pan troglodytes						1	1	1	0.99987
Pan paniscus							1	0.99998	0.99984
Gorilla gorilla								1	0.99991
Mus musculus									1

Table A65. The parameter Positive Predictive Value (PPV) of the HELIOS method with referencing the ClustalΩ in the accuracy measurement of classification output, assuming the *Nine ND5 protein sequences dataset* [3].

	Homo sapiens	Rattus norvegicus	Balaenoptera physalus	Balaenoptera musculus	Didelphis virginiana	Pan troglodytes	Pan paniscus	Gorilla gorilla	Mus musculus
Homo sapiens	1	0.94983	0.92055	0.95408	0.94585	1	1	0.99834	0.9798
Rattus norvegicus		1	0.97655	0.98003	0.88435	0.94701	0.93919	0.93003	0.9783
Balaenoptera physalus			1	1	0.91414	0.94549	0.93825	0.9471	0.94286
Balaenoptera musculus				1	0.9223	0.95556	0.95904	0.954	0.97143
Didelphis virginiana					1	0.94549	0.96633	0.93039	0.92821
Pan troglodytes						1	1	1	0.96954
Pan paniscus							1	0.99667	0.95939
Gorilla gorilla								1	0.9798
Mus musculus									1

Table A66. The parameter Negative Predictive Value (NPV) of the HELIOS method with referencing the ClustalΩ in the accuracy measurement of classification output, assuming the *Nine ND5 protein sequences dataset* [3].

	Homo sapiens	Rattus norvegicus	Balaenoptera physalus	Balaenoptera musculus	Didelphis virginiana	Pan troglodytes	Pan paniscus	Gorilla gorilla	Mus musculus
Homo sapiens	1	0.9999	0.99981	0.99988	0.99989	1	1	0.99999	0.99994
Rattus norvegicus		1	0.99994	0.99995	0.99979	0.99987	0.99987	0.99984	0.99994
Balaenoptera physalus			1	1	0.99985	0.99987	0.99985	0.99987	0.99988
Balaenoptera musculus				1	0.99986	0.99988	0.99989	0.99988	0.99992
Didelphis virginiana					1	0.99988	0.99993	0.99986	0.99985
Pan troglodytes						1	1	1	0.99992
Pan paniscus							1	0.99999	0.9999
Gorilla gorilla								1	0.99994
Mus musculus									1

Table A67. The parameter Matthew's Coefficient Correlation (MCC) of the HELIOS method with referencing the ClustalΩ in the accuracy measurement of classification output, assuming the *Nine ND5 protein sequences dataset* [3].

	Homo sapiens	Rattus norvegicus	Balaenoptera physalus	Balaenoptera musculus	Didelphis virginiana	Pan troglodytes	Pan paniscus	Gorilla gorilla	Mus musculus
Homo sapiens	1	0.9458	0.90189	0.94205	0.9402	1	1	0.99751	0.97241
Rattus norvegicus		1	0.96922	0.97594	0.87673	0.93266	0.93047	0.9167	0.97178
Balaenoptera physalus			1	1	0.91093	0.93275	0.92243	0.93355	0.93415
Balaenoptera musculus				1	0.91752	0.94109	0.94534	0.94117	0.96251
Didelphis virginiana					1	0.93665	0.96303	0.92324	0.91793
Pan troglodytes						1	1	1	0.95978
Pan paniscus							1	0.99501	0.94971
Gorilla gorilla								1	0.97241
Mus musculus									1

Table A68. The parameter Test's Accuracy (F-Score) of the HELIOS method with referencing the ClustalΩ in the accuracy measurement of classification output, assuming the *Nine ND5 protein sequences dataset* [3].

	Homo sapiens	Rattus norvegicus	Balaenoptera physalus	Balaenoptera musculus	Didelphis virginiana	Pan troglodytes	Pan paniscus	Gorilla gorilla	Mus musculus
Homo sapiens	1	0.94588	0.90186	0.94207	0.94029	1	1	0.99751	0.97243
Rattus norvegicus		1	0.96924	0.97597	0.8769	0.93266	0.93054	0.91674	0.97181
Balaenoptera physalus			1	1	0.91107	0.93277	0.92243	0.93356	0.93422
Balaenoptera musculus				1	0.91765	0.94108	0.94533	0.94118	0.96253
Didelphis virginiana					1	0.93671	0.96309	0.92334	0.91801
Pan troglodytes						1	1	1	0.9598
Pan paniscus							1	0.99502	0.94975
Gorilla gorilla								1	0.97243
Mus musculus									1

Table A69. The parameter Sensitivity (SEN) of the HELIOS method with referencing the MUSCLE in the accuracy measurement of classification output, assuming the *Nine ND5 protein sequences dataset* [3].

	Homo sapiens	Rattus norvegicus	Balaenoptera physalus	Balaenoptera musculus	Didelphis virginiana	Pan troglodytes	Pan paniscus	Gorilla gorilla	Mus musculus
Homo sapiens	1	0.94196	0.88391	0.93035	0.93144	1	1	0.99668	0.96517
Rattus norvegicus		1	0.96205	0.97195	0.8796	0.91874	0.92206	0.90381	0.9654
Balaenoptera physalus			1	1	0.89799	0.9204	0.90713	0.9204	0.92574
Balaenoptera musculus				1	0.90301	0.92703	0.93201	0.92869	0.9538
Didelphis virginiana					1	0.92475	0.95987	0.91304	0.90468
Pan troglodytes						1	1	1	0.95025
Pan paniscus							1	0.99337	0.9403
Gorilla gorilla								1	0.96517
Mus musculus									1

Table A70. The parameter Specification (Spec) of the HELIOS method with referencing the MUSCLE in the accuracy measurement of classification output, assuming the *Nine ND5 protein sequences dataset* [3].

	Homo sapiens	Rattus norvegicus	Balaenoptera physalus	Balaenoptera musculus	Didelphis virginiana	Pan troglodytes	Pan paniscus	Gorilla gorilla	Mus musculus
Homo sapiens	1	0.99992	0.99987	0.99993	0.99991	1	1	1	0.99997
Rattus norvegicus		1	0.99996	0.99997	0.99983	0.99992	0.9999	0.99989	0.99996
Balaenoptera physalus			1	1	0.99984	0.99991	0.9999	0.99992	0.99991
Balaenoptera musculus				1	0.99986	0.99993	0.99993	0.99993	0.99995
Didelphis virginiana					1	0.99991	0.99994	0.99988	0.99988
Pan troglodytes						1	1	1	0.99995
Pan paniscus							1	0.99999	0.99993
Gorilla gorilla								1	0.99997
Mus musculus									1

Table A71. The parameter Accuracy (Acc) of the HELIOS method with referencing the MUSCLE in the accuracy measurement of classification output, assuming the *Nine ND5 protein sequences dataset* [3].

	Homo sapiens	Rattus norvegicus	Balaenoptera physalus	Balaenoptera musculus	Didelphis virginiana	Pan troglodytes	Pan paniscus	Gorilla gorilla	Mus musculus
Homo sapiens	1	0.99982	0.99968	0.99981	0.99979	1	1	0.99999	0.99991
Rattus norvegicus		1	0.9999	0.99992	0.99964	0.99978	0.99977	0.99973	0.99991
Balaenoptera physalus			1	1	0.99968	0.99978	0.99975	0.99978	0.99979
Balaenoptera musculus				1	0.9997	0.99981	0.99982	0.99981	0.99988
Didelphis virginiana					1	0.99978	0.99988	0.99974	0.99972
Pan troglodytes						1	1	1	0.99987
Pan paniscus							1	0.99998	0.99984
Gorilla gorilla								1	0.99991
Mus musculus									1

Table A72. The parameter Positive Predictive Value (PPV) of the HELIOS method with referencing the MUSCLE in the accuracy measurement of classification output, assuming the *Nine ND5 protein sequences dataset* [3].

	Homo sapiens	Rattus norvegicus	Balaenoptera physalus	Balaenoptera musculus	Didelphis virginiana	Pan troglodytes	Pan paniscus	Gorilla gorilla	Mus musculus
Homo sapiens	1	0.94983	0.92055	0.95408	0.94247	1	1	0.99834	0.9798
Rattus norvegicus		1	0.97655	0.98003	0.89456	0.94701	0.93919	0.93003	0.9783
Balaenoptera physalus			1	1	0.90404	0.94549	0.93825	0.9471	0.94286
Balaenoptera musculus				1	0.91216	0.95556	0.95904	0.954	0.97143
Didelphis virginiana					1	0.94208	0.96633	0.92699	0.92479
Pan troglodytes						1	1	1	0.96954
Pan paniscus							1	0.99667	0.95939
Gorilla gorilla								1	0.9798
Mus musculus									1

Table A73. The parameter Negative Predictive Value (NPV) of the HELIOS method with referencing the MUSCLE in the accuracy measurement of classification output, assuming the *Nine ND5 protein sequences dataset* [3].

	Homo sapiens	Rattus norvegicus	Balaenoptera physalus	Balaenoptera musculus	Didelphis virginiana	Pan troglodytes	Pan paniscus	Gorilla gorilla	Mus musculus
Homo sapiens	1	0.9999	0.99981	0.99988	0.99989	1	1	0.99999	0.99994
Rattus norvegicus		1	0.99994	0.99995	0.9998	0.99987	0.99987	0.99984	0.99994
Balaenoptera physalus			1	1	0.99983	0.99987	0.99985	0.99987	0.99988
Balaenoptera musculus				1	0.99984	0.99988	0.99989	0.99988	0.99992
Didelphis virginiana					1	0.99988	0.99993	0.99986	0.99984
Pan troglodytes						1	1	1	0.99992
Pan paniscus							1	0.99999	0.9999
Gorilla gorilla								1	0.99994
Mus musculus									1

Table A74. The parameter Matthew's Coefficient Correlation (MCC) of the HELIOS method with referencing the MUSCLE in the accuracy measurement of classification output, assuming the *Nine ND5 protein sequences dataset* [3].

	Homo sapiens	Rattus norvegicus	Balaenoptera physalus	Balaenoptera musculus	Didelphis virginiana	Pan troglodytes	Pan paniscus	Gorilla gorilla	Mus musculus
Homo sapiens	1	0.9458	0.90189	0.94205	0.93683	1	1	0.99751	0.97241
Rattus norvegicus		1	0.96922	0.97594	0.88686	0.93266	0.93047	0.9167	0.97178
Balaenoptera physalus			1	1	0.90085	0.93275	0.92243	0.93355	0.93415
Balaenoptera musculus				1	0.90742	0.94109	0.94534	0.94117	0.96251
Didelphis virginiana					1	0.93326	0.96303	0.91986	0.91454
Pan troglodytes						1	1	1	0.95978
Pan paniscus							1	0.99501	0.94971
Gorilla gorilla								1	0.97241
Mus musculus									1

Table A75. The parameter Test's Accuracy (F-Score) of the HELIOS method with referencing the MUSCLE in the accuracy measurement of classification output, assuming the *Nine ND5 protein sequences dataset* [3].

	Homo sapiens	Rattus norvegicus	Balaenoptera physalus	Balaenoptera musculus	Didelphis virginiana	Pan troglodytes	Pan paniscus	Gorilla gorilla	Mus musculus
Homo sapiens	1	0.94588	0.90186	0.94207	0.93692	1	1	0.99751	0.97243
Rattus norvegicus		1	0.96924	0.97597	0.88702	0.93266	0.93054	0.91674	0.97181
Balaenoptera physalus			1	1	0.90101	0.93277	0.92243	0.93356	0.93422
Balaenoptera musculus				1	0.90756	0.94108	0.94533	0.94118	0.96253
Didelphis virginiana					1	0.93333	0.96309	0.91997	0.91462
Pan troglodytes						1	1	1	0.9598
Pan paniscus							1	0.99502	0.94975
Gorilla gorilla								1	0.97243
Mus musculus									1

Table A76. The parameter Sensitivity (SEN) of the HELIOS method with referencing the T-Coffee in the accuracy measurement of classification output, assuming the *Nine ND5 protein sequences dataset* [3].

	Homo sapiens	Rattus norvegicus	Balaenoptera physalus	Balaenoptera musculus	Didelphis virginiana	Pan troglodytes	Pan paniscus	Gorilla gorilla	Mus musculus
Homo sapiens	1	0.94196	0.88391	0.93035	0.93813	1	1	0.99668	0.96352
Rattus norvegicus		1	0.96535	0.97525	0.87625	0.91874	0.92206	0.90216	0.9654
Balaenoptera physalus			1	1	0.90803	0.9204	0.90713	0.9204	0.92904
Balaenoptera musculus				1	0.91304	0.92703	0.93201	0.92869	0.9538
Didelphis virginiana					1	0.93311	0.96656	0.92977	0.90468
Pan troglodytes						1	1	1	0.95191
Pan paniscus							1	0.99337	0.93864
Gorilla gorilla								1	0.96352
Mus musculus									1

Table A77. The parameter Specification (Spec) of the HELIOS method with referencing the T-Coffee in the accuracy measurement of classification output, assuming the *Nine ND5 protein sequences dataset* [3].

	Homo sapiens	Rattus norvegicus	Balaenoptera physalus	Balaenoptera musculus	Didelphis virginiana	Pan troglodytes	Pan paniscus	Gorilla gorilla	Mus musculus
Homo sapiens	1	0.99992	0.99987	0.99993	0.99992	1	1	1	0.99996
Rattus norvegicus		1	0.99997	0.99997	0.99983	0.99992	0.9999	0.99989	0.99996
Balaenoptera physalus			1	1	0.99986	0.99991	0.9999	0.99992	0.99991
Balaenoptera musculus				1	0.99987	0.99993	0.99993	0.99993	0.99995
Didelphis virginiana					1	0.99992	0.99996	0.99991	0.99988
Pan troglodytes						1	1	1	0.99995
Pan paniscus							1	0.99999	0.99993
Gorilla gorilla								1	0.99996
Mus musculus									1

Table A78. The parameter Accuracy (Acc) of the HELIOS method with referencing the T-Coffee in the accuracy measurement of classification output, assuming the *Nine ND5 protein sequences dataset* [3].

	Homo sapiens	Rattus norvegicus	Balaenoptera physalus	Balaenoptera musculus	Didelphis virginiana	Pan troglodytes	Pan paniscus	Gorilla gorilla	Mus musculus
Homo sapiens	1	0.99982	0.99968	0.99981	0.99982	1	1	0.99999	0.9999
Rattus norvegicus		1	0.99991	0.99993	0.99962	0.99978	0.99977	0.99973	0.99991
Balaenoptera physalus			1	1	0.99971	0.99978	0.99975	0.99978	0.9998
Balaenoptera musculus				1	0.99973	0.99981	0.99982	0.99981	0.99988
Didelphis virginiana					1	0.99981	0.9999	0.99979	0.99972
Pan troglodytes						1	1	1	0.99987
Pan paniscus							1	0.99998	0.99983
Gorilla gorilla								1	0.9999
Mus musculus									1

Table A79. The parameter Positive Predictive Value (PPV) of the HELIOS method with referencing the T-Coffee in the accuracy measurement of classification output, assuming the *Nine ND5 protein sequences dataset* [3].

	Homo sapiens	Rattus norvegicus	Balaenoptera physalus	Balaenoptera musculus	Didelphis virginiana	Pan troglodytes	Pan paniscus	Gorilla gorilla	Mus musculus
Homo sapiens	1	0.94983	0.92055	0.95408	0.94924	1	1	0.99834	0.97811
Rattus norvegicus		1	0.9799	0.98336	0.89116	0.94701	0.93919	0.92833	0.9783
Balaenoptera physalus			1	1	0.91414	0.94549	0.93825	0.9471	0.94622
Balaenoptera musculus				1	0.9223	0.95556	0.95904	0.954	0.97143
Didelphis virginiana					1	0.9506	0.97306	0.94397	0.92479
Pan troglodytes						1	1	1	0.97124
Pan paniscus							1	0.99667	0.9577
Gorilla gorilla								1	0.97811
Mus musculus									1

Table A80. The parameter Negative Predictive Value (NPV) of the HELIOS method with referencing the T-Coffee in the accuracy measurement of classification output, assuming the *Nine ND5 protein sequences dataset* [3].

	Homo sapiens	Rattus norvegicus	Balaenoptera physalus	Balaenoptera musculus	Didelphis virginiana	Pan troglodytes	Pan paniscus	Gorilla gorilla	Mus musculus
Homo sapiens	1	0.9999	0.99981	0.99988	0.9999	1	1	0.99999	0.99994
Rattus norvegicus		1	0.99994	0.99996	0.9998	0.99987	0.99987	0.99984	0.99994
Balaenoptera physalus			1	1	0.99985	0.99987	0.99985	0.99987	0.99988
Balaenoptera musculus				1	0.99986	0.99988	0.99989	0.99988	0.99992
Didelphis virginiana					1	0.99989	0.99994	0.99988	0.99984
Pan troglodytes						1	1	1	0.99992
Pan paniscus							1	0.99999	0.9999
Gorilla gorilla								1	0.99994
Mus musculus									1

Table A81. The parameter Matthew's Coefficient Correlation (MCC) of the HELIOS method with referencing the T-Coffee in the accuracy measurement of classification output, assuming the *Nine ND5 protein sequences dataset* [3].

	Homo sapiens	Rattus norvegicus	Balaenoptera physalus	Balaenoptera musculus	Didelphis virginiana	Pan troglodytes	Pan paniscus	Gorilla gorilla	Mus musculus
Homo sapiens	1	0.9458	0.90189	0.94205	0.94357	1	1	0.99751	0.97074
Rattus norvegicus		1	0.97255	0.97926	0.88349	0.93266	0.93047	0.91501	0.97178
Balaenoptera physalus			1	1	0.91093	0.93275	0.92243	0.93355	0.93749
Balaenoptera musculus				1	0.91752	0.94109	0.94534	0.94117	0.96251
Didelphis virginiana					1	0.94172	0.96975	0.93674	0.91454
Pan troglodytes						1	1	1	0.96146
Pan paniscus							1	0.99501	0.94804
Gorilla gorilla								1	0.97074
Mus musculus									1

Table A82. The parameter Test's Accuracy (F-Score) of the HELIOS method with referencing the T-Coffee in the accuracy measurement of classification output, assuming the *Nine ND5 protein sequences dataset* [3].

	Homo sapiens	Rattus norvegicus	Balaenoptera physalus	Balaenoptera musculus	Didelphis virginiana	Pan troglodytes	Pan paniscus	Gorilla gorilla	Mus musculus
Homo sapiens	1	0.94588	0.90186	0.94207	0.94365	1	1	0.99751	0.97076
Rattus norvegicus		1	0.97257	0.97929	0.88364	0.93266	0.93054	0.91505	0.97181
Balaenoptera physalus			1	1	0.91107	0.93277	0.92243	0.93356	0.93755
Balaenoptera musculus				1	0.91765	0.94108	0.94533	0.94118	0.96253
Didelphis virginiana					1	0.94177	0.9698	0.93682	0.91462
Pan troglodytes						1	1	1	0.96147
Pan paniscus							1	0.99502	0.94807
Gorilla gorilla								1	0.97076
Mus musculus									1

Table A83. The parameter Sensitivity (SEN) of the HELIOS method with referencing the Kalign in the accuracy measurement of classification output, assuming the *Nine ND5 protein sequences dataset* [3].

	Homo sapiens	Rattus norvegicus	Balaenoptera physalus	Balaenoptera musculus	Didelphis virginiana	Pan troglodytes	Pan paniscus	Gorilla gorilla	Mus musculus
Homo sapiens	1	0.94196	0.88391	0.93035	0.93813	1	1	0.99668	0.96517
Rattus norvegicus		1	0.96205	0.97195	0.8796	0.91874	0.92206	0.90381	0.9654
Balaenoptera physalus			1	1	0.9097	0.9204	0.90713	0.9204	0.92574
Balaenoptera musculus				1	0.91472	0.92703	0.93201	0.92869	0.9538
Didelphis virginiana					1	0.93311	0.96656	0.91973	0.91472
Pan troglodytes						1	1	1	0.95025
Pan paniscus							1	0.99337	0.9403
Gorilla gorilla								1	0.96517
Mus musculus									1

Table A84. The parameter Specification (Spec) of the HELIOS method with referencing the Kalign in the accuracy measurement of classification output, assuming the *Nine ND5 protein sequences dataset* [3].

	Homo sapiens	Rattus norvegicus	Balaenoptera physalus	Balaenoptera musculus	Didelphis virginiana	Pan troglodytes	Pan paniscus	Gorilla gorilla	Mus musculus
Homo sapiens	1	0.99992	0.99987	0.99993	0.99992	1	1	1	0.99997
Rattus norvegicus		1	0.99996	0.99997	0.99983	0.99992	0.9999	0.99989	0.99996
Balaenoptera physalus			1	1	0.99986	0.99991	0.9999	0.99992	0.99991
Balaenoptera musculus				1	0.99988	0.99993	0.99993	0.99993	0.99995
Didelphis virginiana					1	0.99992	0.99996	0.99989	0.9999
Pan troglodytes						1	1	1	0.99995
Pan paniscus							1	0.99999	0.99993
Gorilla gorilla								1	0.99997
Mus musculus									1

Table A85. The parameter Accuracy (Acc) of the HELIOS method with referencing the Kalign in the accuracy measurement of classification output, assuming the *Nine ND5 protein sequences dataset* [3].

	Homo sapiens	Rattus norvegicus	Balaenoptera physalus	Balaenoptera musculus	Didelphis virginiana	Pan troglodytes	Pan paniscus	Gorilla gorilla	Mus musculus
Homo sapiens	1	0.99982	0.99968	0.99981	0.99982	1	1	0.99999	0.99991
Rattus norvegicus		1	0.9999	0.99992	0.99964	0.99978	0.99977	0.99973	0.99991
Balaenoptera physalus			1	1	0.99971	0.99978	0.99975	0.99978	0.99979
Balaenoptera musculus				1	0.99974	0.99981	0.99982	0.99981	0.99988
Didelphis virginiana					1	0.99981	0.9999	0.99976	0.99976
Pan troglodytes						1	1	1	0.99987
Pan paniscus							1	0.99998	0.99984
Gorilla gorilla								1	0.99991
Mus musculus									1

Table A86. The parameter Positive Predictive Value (PPV) of the HELIOS method with referencing the Kalign in the accuracy measurement of classification output, assuming the *Nine ND5 protein sequences dataset* [3].

	Homo sapiens	Rattus norvegicus	Balaenoptera physalus	Balaenoptera musculus	Didelphis virginiana	Pan troglodytes	Pan paniscus	Gorilla gorilla	Mus musculus
Homo sapiens	1	0.94983	0.92055	0.95408	0.94924	1	1	0.99834	0.9798
Rattus norvegicus		1	0.97655	0.98003	0.89456	0.94701	0.93919	0.93003	0.9783
Balaenoptera physalus			1	1	0.91582	0.94549	0.93825	0.9471	0.94286
Balaenoptera musculus				1	0.92399	0.95556	0.95904	0.954	0.97143
Didelphis virginiana					1	0.9506	0.97306	0.93379	0.93504
Pan troglodytes						1	1	1	0.96954
Pan paniscus							1	0.99667	0.95939
Gorilla gorilla								1	0.9798
Mus musculus									1

Table A87. The parameter Negative Predictive Value (NPV) of the HELIOS method with referencing the Kalign in the accuracy measurement of classification output, assuming the *Nine ND5 protein sequences dataset* [3].

	Homo sapiens	Rattus norvegicus	Balaenoptera physalus	Balaenoptera musculus	Didelphis virginiana	Pan troglodytes	Pan paniscus	Gorilla gorilla	Mus musculus
Homo sapiens	1	0.9999	0.99981	0.99988	0.9999	1	1	0.99999	0.99994
Rattus norvegicus		1	0.99994	0.99995	0.9998	0.99987	0.99987	0.99984	0.99994
Balaenoptera physalus			1	1	0.99985	0.99987	0.99985	0.99987	0.99988
Balaenoptera musculus				1	0.99986	0.99988	0.99989	0.99988	0.99992
Didelphis virginiana					1	0.99989	0.99994	0.99987	0.99986
Pan troglodytes						1	1	1	0.99992
Pan paniscus							1	0.99999	0.9999
Gorilla gorilla								1	0.99994
Mus musculus									1

Table A88. The parameter Matthew's Coefficient Correlation (MCC) of the HELIOS method with referencing the Kalign in the accuracy measurement of classification output, assuming the *Nine ND5 protein sequences dataset* [3].

	Homo sapiens	Rattus norvegicus	Balaenoptera physalus	Balaenoptera musculus	Didelphis virginiana	Pan troglodytes	Pan paniscus	Gorilla gorilla	Mus musculus
Homo sapiens	1	0.9458	0.90189	0.94205	0.94357	1	1	0.99751	0.97241
Rattus norvegicus		1	0.96922	0.97594	0.88686	0.93266	0.93047	0.9167	0.97178
Balaenoptera physalus			1	1	0.91261	0.93275	0.92243	0.93355	0.93415
Balaenoptera musculus				1	0.91921	0.94109	0.94534	0.94117	0.96251
Didelphis virginiana					1	0.94172	0.96975	0.92661	0.9247
Pan troglodytes						1	1	1	0.95978
Pan paniscus							1	0.99501	0.94971
Gorilla gorilla								1	0.97241
Mus musculus									1

Table A89. The parameter Test's Accuracy (F-Score) of the HELIOS method with referencing the Kalign in the accuracy measurement of classification output, assuming the *Nine ND5 protein sequences dataset* [3].

	Homo sapiens	Rattus norvegicus	Balaenoptera physalus	Balaenoptera musculus	Didelphis virginiana	Pan troglodytes	Pan paniscus	Gorilla gorilla	Mus musculus
Homo sapiens	1	0.94588	0.90186	0.94207	0.94365	1	1	0.99751	0.97243
Rattus norvegicus		1	0.96924	0.97597	0.88702	0.93266	0.93054	0.91674	0.97181
Balaenoptera physalus			1	1	0.91275	0.93277	0.92243	0.93356	0.93422
Balaenoptera musculus				1	0.91933	0.94108	0.94533	0.94118	0.96253
Didelphis virginiana					1	0.94177	0.9698	0.92671	0.92477
Pan troglodytes						1	1	1	0.9598
Pan paniscus							1	0.99502	0.94975
Gorilla gorilla								1	0.97243
Mus musculus									1

Table A90. The parameter Sensitivity (SEN) of the HELIOS method with referencing the MAFFT in the accuracy measurement of classification output, assuming the *Nine ND5 protein sequences dataset* [3].

	Homo sapiens	Rattus norvegicus	Balaenoptera physalus	Balaenoptera musculus	Didelphis virginiana	Pan troglodytes	Pan paniscus	Gorilla gorilla	Mus musculus
Homo sapiens	1	0.94196	0.88391	0.93035	0.9398	1	1	0.99668	0.96517
Rattus norvegicus		1	0.96535	0.97525	0.88127	0.91874	0.92206	0.90381	0.9654
Balaenoptera physalus			1	1	0.91137	0.9204	0.90713	0.9204	0.92904
Balaenoptera musculus				1	0.91639	0.92703	0.93201	0.92869	0.9538
Didelphis virginiana					1	0.93478	0.96823	0.9214	0.91639
Pan troglodytes						1	1	1	0.95025
Pan paniscus							1	0.99337	0.9403
Gorilla gorilla								1	0.96517
Mus musculus									1

Table A91. The parameter Specification (Spec) of the HELIOS method with referencing the MAFFT in the accuracy measurement of classification output, assuming the *Nine ND5 protein sequences dataset* [3].

	Homo sapiens	Rattus norvegicus	Balaenoptera physalus	Balaenoptera musculus	Didelphis virginiana	Pan troglodytes	Pan paniscus	Gorilla gorilla	Mus musculus
Homo sapiens	1	0.99992	0.99987	0.99993	0.99992	1	1	1	0.99997
Rattus norvegicus		1	0.99997	0.99997	0.99983	0.99992	0.9999	0.99989	0.99996
Balaenoptera physalus			1	1	0.99987	0.99991	0.9999	0.99992	0.99991
Balaenoptera musculus				1	0.99988	0.99993	0.99993	0.99993	0.99995
Didelphis virginiana					1	0.99992	0.99996	0.9999	0.9999
Pan troglodytes						1	1	1	0.99995
Pan paniscus							1	0.99999	0.99993
Gorilla gorilla								1	0.99997
Mus musculus									1

Table A92. The parameter Accuracy (Acc) of the HELIOS method with referencing the MAFFT in the accuracy measurement of classification output, assuming the *Nine ND5 protein sequences dataset* [3].

	Homo sapiens	Rattus norvegicus	Balaenoptera physalus	Balaenoptera musculus	Didelphis virginiana	Pan troglodytes	Pan paniscus	Gorilla gorilla	Mus musculus
Homo sapiens	1	0.99982	0.99968	0.99981	0.99982	1	1	0.99999	0.99991
Rattus norvegicus		1	0.99991	0.99993	0.99964	0.99978	0.99977	0.99973	0.99991
Balaenoptera physalus			1	1	0.99972	0.99978	0.99975	0.99978	0.9998
Balaenoptera musculus				1	0.99974	0.99981	0.99982	0.99981	0.99988
Didelphis virginiana					1	0.99982	0.99991	0.99977	0.99976
Pan troglodytes						1	1	1	0.99987
Pan paniscus							1	0.99998	0.99984
Gorilla gorilla								1	0.99991
Mus musculus									1

Table A93. The parameter Positive Predictive Value (PPV) of the HELIOS method with referencing the MAFFT in the accuracy measurement of classification output, assuming the *Nine ND5 protein sequences dataset* [3].

	Homo sapiens	Rattus norvegicus	Balaenoptera physalus	Balaenoptera musculus	Didelphis virginiana	Pan troglodytes	Pan paniscus	Gorilla gorilla	Mus musculus
Homo sapiens	1	0.94983	0.92055	0.95408	0.95093	1	1	0.99834	0.9798
Rattus norvegicus		1	0.9799	0.98336	0.89626	0.94701	0.93919	0.93003	0.9783
Balaenoptera physalus			1	1	0.91751	0.94549	0.93825	0.9471	0.94622
Balaenoptera musculus				1	0.92568	0.95556	0.95904	0.954	0.97143
Didelphis virginiana					1	0.9523	0.97475	0.93548	0.93675
Pan troglodytes						1	1	1	0.96954
Pan paniscus							1	0.99667	0.95939
Gorilla gorilla								1	0.9798
Mus musculus									1

Table A94. The parameter Negative Predictive Value (NPV) of the HELIOS method with referencing the MAFFT in the accuracy measurement of classification output, assuming the *Nine ND5 protein sequences dataset* [3].

	Homo sapiens	Rattus norvegicus	Balaenoptera physalus	Balaenoptera musculus	Didelphis virginiana	Pan troglodytes	Pan paniscus	Gorilla gorilla	Mus musculus
Homo sapiens	1	0.9999	0.99981	0.99988	0.9999	1	1	0.99999	0.99994
Rattus norvegicus		1	0.99994	0.99996	0.99981	0.99987	0.99987	0.99984	0.99994
Balaenoptera physalus			1	1	0.99985	0.99987	0.99985	0.99987	0.99988
Balaenoptera musculus				1	0.99986	0.99988	0.99989	0.99988	0.99992
Didelphis virginiana					1	0.99989	0.99995	0.99987	0.99986
Pan troglodytes						1	1	1	0.99992
Pan paniscus							1	0.99999	0.9999
Gorilla gorilla								1	0.99994
Mus musculus									1

Table A95. The parameter Matthew's Coefficient Correlation (MCC) of the HELIOS method with referencing the MAFFT in the accuracy measurement of classification output, assuming the *Nine ND5 protein sequences dataset* [3].

	Homo sapiens	Rattus norvegicus	Balaenoptera physalus	Balaenoptera musculus	Didelphis virginiana	Pan troglodytes	Pan paniscus	Gorilla gorilla	Mus musculus
Homo sapiens	1	0.9458	0.90189	0.94205	0.94526	1	1	0.99751	0.97241
Rattus norvegicus		1	0.97255	0.97926	0.88855	0.93266	0.93047	0.9167	0.97178
Balaenoptera physalus			1	1	0.91429	0.93275	0.92243	0.93355	0.93749
Balaenoptera musculus				1	0.92089	0.94109	0.94534	0.94117	0.96251
Didelphis virginiana					1	0.94341	0.97144	0.9283	0.9264
Pan troglodytes						1	1	1	0.95978
Pan paniscus							1	0.99501	0.94971
Gorilla gorilla								1	0.97241
Mus musculus									1

Table A96. The parameter Test's Accuracy (F-Score) of the HELIOS method with referencing the MAFFT in the accuracy measurement of classification output, assuming the *Nine ND5 protein sequences dataset* [3].

	Homo sapiens	Rattus norvegicus	Balaenoptera physalus	Balaenoptera musculus	Didelphis virginiana	Pan troglodytes	Pan paniscus	Gorilla gorilla	Mus musculus
Homo sapiens	1	0.94588	0.90186	0.94207	0.94533	1	1	0.99751	0.97243
Rattus norvegicus		1	0.97257	0.97929	0.8887	0.93266	0.93054	0.91674	0.97181
Balaenoptera physalus			1	1	0.91443	0.93277	0.92243	0.93356	0.93755
Balaenoptera musculus				1	0.92101	0.94108	0.94533	0.94118	0.96253
Didelphis virginiana					1	0.94346	0.97148	0.92839	0.92646
Pan troglodytes						1	1	1	0.9598
Pan paniscus							1	0.99502	0.94975
Gorilla gorilla								1	0.97243
Mus musculus									1
