# Supplementary material for: HELIOS: High-speed sequence alignment in optics
Source: PLoS Comput Biol. 2022 Nov 21;18(11):e1010665. doi: 10.1371/journal.pcbi.1010665 (PMC9678324; doi:10.1371/journal.pcbi.1010665)
Supplement: S2 Text — (PDF) [file pcbi.1010665.s002.pdf]

HELIOS: High-Speed Sequence Alignment in Optics: S2 Text

EHSAN MALEKI[1], SAEEDEH AKBARI ROKN ABADI[1], AND SOMAYYEH KOOHI[1,*]

[1] Department of Computer Engineering, Sharif University of Technology, Azadi Ave., Tehran, Iran.
[*] Corresponding author: koohi@sharif.edu

Compiled October 20, 2022

In response to the imperfections of current sequence alignment methods, originated from the inherent serialism within their corresponding electrical systems, a few optical approaches for biological data comparison have been proposed recently. However, due to their low performance, raised from their inefficient coding scheme, this paper presents a novel all-optical high-throughput method for aligning DNA, RNA, and protein sequences, named HELIOS. The HELIOS method employs highly sophisticated operations to locate character matches, single or multiple mutations, and single or multiple indels within various biological sequences. On the other hand, the HELIOS optical architecture exploits high-speed processing and operational parallelism in optics, by adopting wavelength and polarization of optical beams. For evaluation, the functionality and accuracy of the HELIOS method are approved through behavioral and optical simulation studies, while its complexity and performance are estimated through analytical computation. The accuracy evaluations indicate that the HELIOS method achieves a precise pairwise alignment of two sequences, highly similar to those of Smith-Waterman, Needleman-Wunsch, BLAST, MUSCLE, ClustalW, ClustalΩ, T-Coffee, Kalign, and MAFFT. According to our performance evaluations, the HELIOS optical architecture outperforms all alternative electrical and optical algorithms in terms of processing time and memory requirement, relying on its highly sophisticated method and optical architecture. Moreover, the employed compact coding scheme highly escalates the number of input characters, and hence, it offers reduced time and space complexities, compared to the electrical and optical alternatives. It makes the HELIOS method and optical architecture highly applicable for biomedical applications.

1. ACCURACY EVALUATION

In order to comprehensively assess the accuracy of the HELIOS method, two statistical analyses are performed through simulation studies: 1) Quantitative measurement of homology [1], and 2) Accuracy measurement of classification output [2], compared to the well-known algorithms, briefly reported in Tables A1 and A2, respectively. In this manner, the *Nine beta globin protein sequences dataset* [3] is assumed in this study, as represented in Table A3.

A. Quantitative measurement of homology

To perform quantitative measurement of homology [1], the parameters Identity, Similarity, and Alignment Score of the HELIOS outputs are calculated through simulation studies, as reported in Tables A4-A6, respectively, assuming the *Nine beta globin protein sequences dataset* [3]. While the Identity reports the number of exactly matched characters of two sequences (in percentage), the Similarity measures the resemblance of two compared sequences. Specifically, regarding the physicochemical properties, the amino acids are categorized into six groups with different Similarity values; including GAVLI, FYW, STCM, KRH, DENQ, and P. As the third metric, the BLOSUM62 [4] substitution scoring matrix [4] is adopted to calculate the Alignment Score, with gap opening and extension penalties equal to -10 and -0.5, respectively.

For a comparative study, the values of Identity, Similarity, and Alignment Score of the quantitative measurement of homology is performed by various well-known algorithms to be compared to the those of HELIOS method, assuming *Nine beta globin protein sequences dataset* [3]. It includes Smith-Waterman (SW) [5] reported in Tables A7-A9, Needleman-Wunsch (NW) [6] reported in Tables A10-A12, BLAST [7] reported in Tables A13-A15, ClustalW [8] reported in Tables A16-A18, Clustal-Omega [9] reported in Tables A19-A21, MUSCLE [9] reported in Tables A22-A24, T-Coffee [10] reported in Tables A25-A27, Kalign [11] reported in Tables A28-A30, and MAFFT [12] reported in Tables A31-A33.

Table A1. A brief report of the quantitative measurement of homology of the HELIOS method, compared to nine well-known algorithms, including SW, NW, BLAST, ClustalW, Clustal Omega, Muscle, T-Coffee, Kalign, and MAFFT. The parameters Identity, Similarity, and Alignment score are averaged and reported. The dataset used in this study is the *Nine beta globin protein sequences dataset* [3].

	HELIOS	SW	NW	BLAST	MUSCLE	ClustalW	ClustalΩ	T-Coffee	Kalign	MAFFT
Identity	80.358	80.283	80.283	80.283	80.283	80.283	80.283	80.878	80.283	80.283
Similarity	88.591	88.794	88.794	88.848	88.848	88.848	88.848	89.622	88.848	88.848
Alignment Score	583.4	588.1	595.1	588.7	588.7	588.7	588.7	588.7	588.7	588.7

Table A2. A brief report of the accuracy measurement of classification output of the HELIOS method with referencing well-known algorithms, including SW, NW, BLAST, ClustalW, Clustal Omega, Muscle, T-Coffee, Kalign, and MAFFT. The parameters SEN, Spec, Acc, PPV, NPV, MCC, and F-Score are averaged and reported. The dataset used in this study is the *Nine beta globin protein sequences dataset* [3].

	SW	NW	BLAST	MUSCLE	ClustalW	ClustalΩ	T-Coffee	Kalign	MAFFT
SEN	0.98565	0.98565	0.98914	0.98675	0.98675	0.98822	0.71116	0.98914	0.98675
Spec	0.99994	0.99993	0.99996	0.99995	0.99995	0.99996	0.99838	0.99996	0.99995
ACC	0.99984	0.99983	0.99989	0.99986	0.99986	0.99988	0.99643	0.99989	0.99986
PPV	0.99097	0.99097	0.99446	0.99207	0.99207	0.99354	0.74743	0.99446	0.99207
NPV	0.99990	0.99990	0.99993	0.99991	0.99991	0.99992	0.99803	0.99993	0.99991
MCC	0.98821	0.98820	0.99172	0.98932	0.98932	0.99080	0.72670	0.99172	0.98932
F-Score	0.98827	0.98827	0.99176	0.98937	0.98937	0.99084	0.72768	0.99176	0.98937

B. Accuracy measurement of classification output

Afterward, the accuracy measurement of the classification output [2] of the HELIOS method is addressed by calculating the values of Sensitivity (SEN), Specificity (Spec), Accuracy (ACC), Positive Predictive Value (PPV), Negative Predictive Value (NPV), Matthew's Coefficient Correlation (MCC), and Test's Accuracy (F-Score) in the simulation studies, according to Eq 5 to Eq 11, respectively.

As a comparative study, the accuracy measurement of the classification output of the HELIOS method is accomplished, assuming *Nine beta globin protein sequences dataset* [3], and the corresponding metrics are calculated with considering Smith-Waterman [5] reported in Tables A34-A40, Needleman-Wunsch [6] reported in Tables A41-A47, ClustalW [8] reported in Tabls A55-A61, Clustal-Omega [9] reported in Tables A62-A68, BLAST [7] reported in Tablse A48-A54, MUSCLE [13] reported in Tables A69-A75, T-Coffee [10] reported in Tables A76-A82, Kalign [11] reported in Tables A83-A89, and MAFFT [12] reported in Tables A90-A96.

REFERENCES

1. D. S. Moss, S. Jelaska, and S. Pongor, *Essays in bioinformatics*, vol. 368 (IOS Press, 2005).
2. M. Hamada, H. Kiryu, W. Iwasaki, and K. Asai, "Generalized centroid estimators in bioinformatics," PloS one **6**, e16450 (2011).
3. M. M. Abo-Elkhier, M. A. Abd Elwahaab, and M. I. Abo El Maaty, "Measuring similarity among protein sequences using a new descriptor," BioMed research international **2019** (2019).
4. D. W. Mount, "Using blosum in sequence alignments," Cold Spring Harb. Protoc. **2008**, pdb.top39 (2008).
5. H. Zou, S. Tang, C. Yu, H. Fu, Y. Li, and W. Tang, "asw: accelerating smith–waterman algorithm on coupled cpu–gpu architecture," Int. J. Parallel Program. **47**, 388–402 (2019).
6. Y. Jararweh, M. Al-Ayyoub, M. Fakirah, L. Alawneh, and B. B. Gupta, "Improving the performance of the needleman-wunsch algorithm using parallelization and vectorization techniques," Multimed. Tools Appl. **78**, 3961–3977 (2019).
7. G. M. Boratyn, J. Thierry-Mieg, D. Thierry-Mieg, B. Busby, and T. L. Madden, "Magic-blast, an accurate rna-seq aligner for long and short reads," BMC bioinformatics **20**, 1–19 (2019).
8. D. Díaz, F. J. Esteban, P. Hernández, J. A. Caballero, A. Guevara, G. Dorado, and S. Gálvez, "Mc64-clustalwp2: A highly-parallel hybrid strategy to align multiple sequences in many-core architectures," PLOS ONE **9**, 1–12 (2014).
9. F. Sievers and D. G. Higgins, "Clustal omega for making accurate alignments of many protein sequences," Protein Sci. **27**, 135–145 (2018).
10. C. Notredame, D. G. Higgins, and J. Heringa, "T-coffee: a novel method for fast and accurate multiple sequence alignment11edited by j. thornton," J. Mol. Biol. **302**, 205–217 (2000).
11. T. Lassmann, "Kalign 3: multiple sequence alignment of large datasets," (2020).
12. J. Rozewicki, S. Li, K. M. Amada, D. M. Standley, and K. Katoh, "Mafft-dash: integrated protein sequence and structural alignment," Nucleic acids research **47**, W5–W10 (2019).
13. R. C. Edgar, "MUSCLE: multiple sequence alignment with high accuracy and high throughput," Nucleic Acids Res. **32**, 1792–1797 (2004).

Table A3. The list of input sequences, assuming the *Nine beta globin protein sequences dataset* [3].

Name	Sequence
Gorilla gorilla	MVHLT PEEKS AVTAL WGKVN VDEVG GEALG RLLVV YPWTQ RFFES FGDLS TPDAV MGNPK VKAHG KKVLG AFSDG LAHLD NLKGT FATLS ELHCD KLHVD PENFK LLGNV LVCVL AHHFG K
Pan troglodytes	MVHLT PEEKS AVTAL WGKVN VDEVG GEALG RLVSR LLVVY PWTQR FFESF GDLST PDAVM GNPKV KAHGK KVLGA FSDGL AHLDN LKGTF ATLSE LHCDK LHVDP ENFRL LGNVL VCVLA HHFGK
Homo sapiens	MVHLT PEEKS AVTAL WGKVN VDEVG GEALG RLLVV YPWTQ RFFES FGDLS TPDAV MGNPK VKAHG KKVLG AFSDG LAHLD NLKGT FATLS ELHCD KLHVD PENFR LLGNV LVCVL AHHFG KEFTP PVQAA YQKVV AGVAN ALAHKYH
Rattus norvegicus	MVHLT DAEKA TVSGL WGKVN PDNVG AEALG RLLVV YPWTQ RYFSK FGDLS SASAI MGNPQ VKAHG KKVIN AFNDG LKHLD NLKGT FAHLS ELHCD KLHVD PENFR LLGNM IVIVL GHHLG KEFTP SAQAA FQKVV AGVAS ALAHKYH
Mus musculus	MVHLT DAEKS AVSCL WAKVN PDEVG GEALG RLLVV YPWTQ RYFDS FGDLS SASAI MGNPK VKAHG KKVIT AFNEG LKNLD NLKGT FASLS ELHCD KLHVD PENFR LLGNA IVIVL GHHLG KDFTP AAQAA FQKVV AGVAT ALAHKYH
Taeniopygia guttata	MVQWT AEEKQ LITGL WGKVN VAECG GEALA RLLIV YPWTQ RFFAS FGNLS SPTAV LGNPK VQAHG KKVLT SFGEA VKNLD SIKNT FSQLS ELHCD KLHVD PENFR LLGDI LVVVL AAHFG KDFTP DCQAA WQKLV RVVAH ALARKYH
Cairina moschata	MVHWT AEEKQ LITGL WGKVN VADCG AEALA RLLIV YPWTQ RFFAS FGNLS SPTAI LGNPM VRAHG KKVLT SFGDA VKNLD NIKNT FAQLS ELHCD KLHVD PENFR LLGDI LIIVL AAHFT KDFTP DCQAA WQKLV RVVAH ALARKYH
Gallus gallus	MVHWT AEEKQ LITGL WGKVN VAECG AEALA RLLIV YPWTQ RFFAS FGNLS SPTAI LGNPM VRAHG KKVLT SFGDA VKNLD NIKNT FSQLS ELHCD KLHVD PENFR LLGDI LIIVL AAHFS KDFTP ECQAA WQKLV RVVAH ALARKYH
Didelphis virginiana	MVHLT SEEKN CITTI WSKVQ VDQTG GEALG RMLVV YPWTT RFFGS FGDLS SPGAV MSNSK VQAHG AKVLT SFGEA VKHLD NLKGT YAKLS ELHCD KLHVD PENFK MLGNI IVICL AEHFG KDFTP ECQVA WQKLV AGVAH ALAHKYH

Table A4. The parameter Identity of the HELIOS method in the quantitative measurement of homology, assuming the *Nine beta globin protein sequences dataset* [3].

	Gorilla gorilla	Pan troglodytes	Homo sapiens	Rattus norvegicus	Mus musculus	Taeniopygia guttata	Cairina moschata	Gallus gallus	Didelphis virginiana
Gorilla gorilla	100	99.1736	99.1736	76.8595	79.3388	71.0744	69.4215	69.4215	71.9008
Pan troglodytes		100	96.8	75.2	77.6	69.6	68	68	68.8
Homo sapiens			100	78.9116	80.2721	70.7483	69.3878	69.3878	71.4286
Rattus norvegicus				100	88.4354	63.9456	67.3469	66.6667	65.9864
Mus musculus					100	67.3469	66.6667	66.6667	69.3878
Taeniopygia guttata						100	91.8367	92.517	71.4286
Cairina moschata							100	97.2789	70.068
Gallus gallus								100	70.068
Didelphis virginiana									100

Table A5. The parameter Similarity of the HELIOS method in the quantitative measurement of homology, assuming the *Nine beta globin protein sequences dataset* [3].

	Gorilla gorilla	Pan troglodytes	Homo sapiens	Rattus norvegicus	Mus musculus	Taeniopygia guttata	Cairina moschata	Gallus gallus	Didelphis virginiana
Gorilla gorilla	100	100	100	85.9504	90.0826	82.6446	84.2975	83.4711	80.9917
Pan troglodytes		100	96.8	83.2	87.2	80	81.6	80.8	78.4
Homo sapiens			100	87.0748	90.4762	82.9932	82.9932	83.6735	82.3129
Rattus norvegicus				100	92.517	77.551	80.2721	79.5918	80.2721
Mus musculus					100	81.6327	82.9932	82.3129	81.6327
Taeniopygia guttata						100	95.9184	96.5986	84.3537
Cairina moschata							100	99.3197	83.6735
Gallus gallus								100	82.9932
Didelphis virginiana									100

Table A6. The parameter Alignment Score of the HELIOS method in the quantitative measurement of homology, assuming the *Nine beta globin protein sequences dataset* [3].

	Gorilla gorilla	Pan troglodytes	Homo sapiens	Rattus norvegicus	Mus musculus	Taeniopygia guttata	Cairina moschata	Gallus gallus	Didelphis virginiana
Gorilla gorilla	644	629.5	618.5	489.5	500.5	445.5	441.5	443.5	460.5
Pan troglodytes		661	610	481	492	437	433	435	433.5
Homo sapiens			780	630	637	556	533	554	583
Rattus norvegicus				773	686	471.5	501	500	540
Mus musculus					768	511	503	506	563
Taeniopygia guttata						774	730	734	576
Cairina moschata							778	763	541
Gallus gallus								775	545
Didelphis virginiana									786

Table A7. The parameter Identity of the Smith-Waterman in the quantitative measurement of homology, assuming the *Nine beta globin protein sequences dataset* [3].

	Gorilla gorilla	Pan troglodytes	Homo sapiens	Rattus norvegicus	Mus musculus	Taeniopygia guttata	Cairina moschata	Gallus gallus	Didelphis virginiana
Gorilla gorilla	100	99.1736	99.1736	76.8595	79.3388	71.0744	69.4215	69.4215	71.9008
Pan troglodytes		100	96.8	75.2	77.6	69.6	68	68	68.8
Homo sapiens			100	78.9116	80.2721	70.7483	69.3878	69.3878	71.4286
Rattus norvegicus				100	88.4354	63.2653	66.6667	65.9864	65.9864
Mus musculus					100	67.3469	66.6667	66.6667	69.3878
Taeniopygia guttata						100	91.8367	92.517	71.4286
Cairina moschata							100	97.2789	69.3878
Gallus gallus								100	69.3878
Didelphis virginiana									100

Table A8. The parameter Similarity of the Smith-Waterman in the quantitative measurement of homology, assuming the *Nine beta globin protein sequences dataset* [3].

	Gorilla gorilla	Pan troglodytes	Homo sapiens	Rattus norvegicus	Mus musculus	Taeniopygia guttata	Cairina moschata	Gallus gallus	Didelphis virginiana
Gorilla gorilla	100	100	100	85.9504	90.0826	82.6446	84.2975	83.4711	80.9917
Pan troglodytes		100	96.8	83.2	87.2	79.2	80.8	80	78.4
Homo sapiens			100	87.0748	90.4762	82.9932	84.3537	83.6735	82.3129
Rattus norvegicus				100	92.517	79.5918	81.6327	80.9524	80.2721
Mus musculus					100	82.9932	84.3537	83.6735	81.6327
Taeniopygia guttata						100	95.9184	96.5986	84.3537
Cairina moschata							100	99.3197	84.3537
Gallus gallus								100	83.6735
Didelphis virginiana									100

Table A9. The parameter Alignment Score of the Smith-Waterman in the quantitative measurement of homology, assuming the *Nine beta globin protein sequences dataset* [3].

	Gorilla gorilla	Pan troglodytes	Homo sapiens	Rattus norvegicus	Mus musculus	Taeniopygia guttata	Cairina moschata	Gallus gallus	Didelphis virginiana
Gorilla gorilla	644	629.5	618.5	489.5	500.5	445.5	441.5	443.5	460.5
Pan troglodytes		661	610	481	492	428.5	424.5	426.5	446
Homo sapiens			780	630	637	556	552	554	583
Rattus norvegicus				773	686	516	534	532	540
Mus musculus					768	530	522	525	563
Taeniopygia guttata						774	730	734	576
Cairina moschata							778	763	562
Gallus gallus								775	566
Didelphis virginiana									786

Table A10. The parameter Identity of the Needleman-Wunsch in the quantitative measurement of homology, assuming the *Nine beta globin protein sequences dataset* [3].

	Gorilla gorilla	Pan troglodytes	Homo sapiens	Rattus norvegicus	Mus musculus	Taeniopygia guttata	Cairina moschata	Gallus gallus	Didelphis virginiana
Gorilla gorilla	100	99.1736	99.1736	76.8595	79.3388	71.0744	69.4215	69.4215	71.9008
Pan troglodytes		100	96.8	75.2	77.6	69.6	68	68	68.8
Homo sapiens			100	78.9116	80.2721	70.7483	69.3878	69.3878	71.4286
Rattus norvegicus				100	88.4354	63.2653	66.6667	65.9864	65.9864
Mus musculus					100	67.3469	66.6667	66.6667	69.3878
Taeniopygia guttata						100	91.8367	92.517	71.4286
Cairina moschata							100	97.2789	69.3878
Gallus gallus								100	69.3878
Didelphis virginiana									100

Table A11. The parameter Similarity of the Needleman-Wunsch in the quantitative measurement of homology, assuming the *Nine beta globin protein sequences dataset* [3].

	Gorilla gorilla	Pan troglodytes	Homo sapiens	Rattus norvegicus	Mus musculus	Taeniopygia guttata	Cairina moschata	Gallus gallus	Didelphis virginiana
Gorilla gorilla	100	100	100	85.9504	90.0826	82.6446	84.2975	83.4711	80.9917
Pan troglodytes		100	96.8	83.2	87.2	79.2	80.8	80	78.4
Homo sapiens			100	87.0748	90.4762	82.9932	84.3537	83.6735	82.3129
Rattus norvegicus				100	92.517	79.5918	81.6327	80.9524	80.2721
Mus musculus					100	82.9932	84.3537	83.6735	81.6327
Taeniopygia guttata						100	95.9184	96.5986	84.3537
Cairina moschata							100	99.3197	84.3537
Gallus gallus								100	83.6735
Didelphis virginiana									100

Table A12. The parameter Alignment Score of the Needleman-Wunsch in the quantitative measurement of homology, assuming the *Nine beta globin protein sequences dataset* [3].

	Gorilla gorilla	Pan troglodytes	Homo sapiens	Rattus norvegicus	Mus musculus	Taeniopygia guttata	Cairina moschata	Gallus gallus	Didelphis virginiana
Gorilla gorilla	644	629.5	641	512	523	468	464	466	483
Pan troglodytes		661	632.5	503.5	514.5	451	447	449	468.5
Homo sapiens			780	630	637	556	552	554	583
Rattus norvegicus				773	686	516	534	532	540
Mus musculus					768	530	522	525	563
Taeniopygia guttata						774	730	734	576
Cairina moschata							778	763	562
Gallus gallus								775	566
Didelphis virginiana									786

Table A13. The parameter Identity of the BLAST in the quantitative measurement of homology, assuming the *Nine beta globin protein sequences dataset* [3].

	Gorilla gorilla	Pan troglodytes	Homo sapiens	Rattus norvegicus	Mus musculus	Taeniopygia guttata	Cairina moschata	Gallus gallus	Didelphis virginiana
Gorilla gorilla	100	99.1736	99.1736	76.8595	79.3388	71.0744	69.4215	69.4215	71.9008
Pan troglodytes		100	96.8	75.2	77.6	69.6	68	68	68.8
Homo sapiens			100	78.9116	80.2721	70.7483	69.3878	69.3878	71.4286
Rattus norvegicus				100	88.4354	63.2653	66.6667	65.9864	65.9864
Mus musculus					100	67.3469	66.6667	66.6667	69.3878
Taeniopygia guttata						100	91.8367	92.517	71.4286
Cairina moschata							100	97.2789	69.3878
Gallus gallus								100	69.3878
Didelphis virginiana									100

Table A14. The parameter Similarity of the BLAST in the quantitative measurement of homology, assuming the *Nine beta globin protein sequences dataset* [3].

	Gorilla gorilla	Pan troglodytes	Homo sapiens	Rattus norvegicus	Mus musculus	Taeniopygia guttata	Cairina moschata	Gallus gallus	Didelphis virginiana
Gorilla gorilla	100	100	100	85.9504	90.0826	82.6446	84.2975	83.4711	80.9917
Pan troglodytes		100	96.8	83.2	87.2	80	81.6	80.8	78.4
Homo sapiens			100	87.0748	90.4762	82.9932	84.3537	83.6735	82.3129
Rattus norvegicus				100	92.517	79.5918	81.6327	80.9524	80.2721
Mus musculus					100	82.9932	84.3537	83.6735	81.6327
Taeniopygia guttata						100	95.9184	96.5986	84.3537
Cairina moschata							100	99.3197	84.3537
Gallus gallus								100	83.6735
Didelphis virginiana									100

Table A15. The parameter Alignment Score of the BLAST in the quantitative measurement of homology, assuming the *Nine beta globin protein sequences dataset* [3].

	Gorilla gorilla	Pan troglodytes	Homo sapiens	Rattus norvegicus	Mus musculus	Taeniopygia guttata	Cairina moschata	Gallus gallus	Didelphis virginiana
Gorilla gorilla	644	629.5	618.5	489.5	500.5	445.5	441.5	443.5	460.5
Pan troglodytes		661	610	481	492	437	433	435	446
Homo sapiens			780	630	637	556	552	554	583
Rattus norvegicus				773	686	516	534	532	540
Mus musculus					768	530	522	525	563
Taeniopygia guttata						774	730	734	576
Cairina moschata							778	763	562
Gallus gallus								775	566
Didelphis virginiana									786

Table A16. The parameter Identity of the ClustalW in the quantitative measurement of homology, assuming the *Nine beta globin protein sequences dataset* [3].

	Gorilla gorilla	Pan troglodytes	Homo sapiens	Rattus norvegicus	Mus musculus	Taeniopygia guttata	Cairina moschata	Gallus gallus	Didelphis virginiana
Gorilla gorilla	100	99.1736	99.1736	76.8595	79.3388	71.0744	69.4215	69.4215	71.9008
Pan troglodytes		100	96.8	75.2	77.6	69.6	68	68	68.8
Homo sapiens			100	78.9116	80.2721	70.7483	69.3878	69.3878	71.4286
Rattus norvegicus				100	88.4354	63.2653	66.6667	65.9864	65.9864
Mus musculus					100	67.3469	66.6667	66.6667	69.3878
Taeniopygia guttata						100	91.8367	92.517	71.4286
Cairina moschata							100	97.2789	69.3878
Gallus gallus								100	69.3878
Didelphis virginiana									100

Table A17. The parameter Similarity of the ClustalW in the quantitative measurement of homology, assuming the *Nine beta globin protein sequences dataset* [3].

	Gorilla gorilla	Pan troglodytes	Homo sapiens	Rattus norvegicus	Mus musculus	Taeniopygia guttata	Cairina moschata	Gallus gallus	Didelphis virginiana
Gorilla gorilla	100	100	100	85.9504	90.0826	82.6446	84.2975	83.4711	80.9917
Pan troglodytes		100	96.8	83.2	87.2	80	81.6	80.8	78.4
Homo sapiens			100	87.0748	90.4762	82.9932	84.3537	83.6735	82.3129
Rattus norvegicus				100	92.517	79.5918	81.6327	80.9524	80.2721
Mus musculus					100	82.9932	84.3537	83.6735	81.6327
Taeniopygia guttata						100	95.9184	96.5986	84.3537
Cairina moschata							100	99.3197	84.3537
Gallus gallus								100	83.6735
Didelphis virginiana									100

Table A18. The parameter Alignment Score of the ClustalW in the quantitative measurement of homology, assuming the *Nine beta globin protein sequences dataset* [3].

	Gorilla gorilla	Pan troglodytes	Homo sapiens	Rattus norvegicus	Mus musculus	Taeniopygia guttata	Cairina moschata	Gallus gallus	Didelphis virginiana
Gorilla gorilla	644	629.5	618.5	489.5	500.5	445.5	441.5	443.5	460.5
Pan troglodytes		661	610	481	492	437	433	435	446
Homo sapiens			780	630	637	556	552	554	583
Rattus norvegicus				773	686	516	534	532	540
Mus musculus					768	530	522	525	563
Taeniopygia guttata						774	730	734	576
Cairina moschata							778	763	562
Gallus gallus								775	566
Didelphis virginiana									786

Table A19. The parameter Identity of the ClustalΩ in the quantitative measurement of homology, assuming the *Nine beta globin protein sequences dataset* [3].

	Gorilla gorilla	Pan troglodytes	Homo sapiens	Rattus norvegicus	Mus musculus	Taeniopygia guttata	Cairina moschata	Gallus gallus	Didelphis virginiana
Gorilla gorilla	100	99.1736	99.1736	76.8595	79.3388	71.0744	69.4215	69.4215	71.9008
Pan troglodytes		100	96.8	75.2	77.6	69.6	68	68	68.8
Homo sapiens			100	78.9116	80.2721	70.7483	69.3878	69.3878	71.4286
Rattus norvegicus				100	88.4354	63.2653	66.6667	65.9864	65.9864
Mus musculus					100	67.3469	66.6667	66.6667	69.3878
Taeniopygia guttata						100	91.8367	92.517	71.4286
Cairina moschata							100	97.2789	69.3878
Gallus gallus								100	69.3878
Didelphis virginiana									100

Table A20. The parameter Similarity of the ClustalΩ in the quantitative measurement of homology, assuming the *Nine beta globin protein sequences dataset* [3].

	Gorilla gorilla	Pan troglodytes	Homo sapiens	Rattus norvegicus	Mus musculus	Taeniopygia guttata	Cairina moschata	Gallus gallus	Didelphis virginiana
Gorilla gorilla	100	100	100	85.9504	90.0826	82.6446	84.2975	83.4711	80.9917
Pan troglodytes		100	96.8	83.2	87.2	80	81.6	80.8	78.4
Homo sapiens			100	87.0748	90.4762	82.9932	84.3537	83.6735	82.3129
Rattus norvegicus				100	92.517	79.5918	81.6327	80.9524	80.2721
Mus musculus					100	82.9932	84.3537	83.6735	81.6327
Taeniopygia guttata						100	95.9184	96.5986	84.3537
Cairina moschata							100	99.3197	84.3537
Gallus gallus								100	83.6735
Didelphis virginiana									100

Table A21. The parameter Alignment Score of the ClustalΩ in the quantitative measurement of homology, assuming the *Nine beta globin protein sequences dataset* [3].

	Gorilla gorilla	Pan troglodytes	Homo sapiens	Rattus norvegicus	Mus musculus	Taeniopygia guttata	Cairina moschata	Gallus gallus	Didelphis virginiana
Gorilla gorilla	644	629.5	618.5	489.5	500.5	445.5	441.5	443.5	460.5
Pan troglodytes		661	610	481	492	437	433	435	446
Homo sapiens			780	630	637	556	552	554	583
Rattus norvegicus				773	686	516	534	532	540
Mus musculus					768	530	522	525	563
Taeniopygia guttata						774	730	734	576
Cairina moschata							778	763	562
Gallus gallus								775	566
Didelphis virginiana									786

Table A22. The parameter Identity of the MUSCLE in the quantitative measurement of homology, assuming the *Nine beta globin protein sequences dataset* [3].

	Gorilla gorilla	Pan troglodytes	Homo sapiens	Rattus norvegicus	Mus musculus	Taeniopygia guttata	Cairina moschata	Gallus gallus	Didelphis virginiana
Gorilla gorilla	100	99.1736	99.1736	76.8595	79.3388	71.0744	69.4215	69.4215	71.9008
Pan troglodytes		100	96.8	75.2	77.6	69.6	68	68	68.8
Homo sapiens			100	78.9116	80.2721	70.7483	69.3878	69.3878	71.4286
Rattus norvegicus				100	88.4354	63.2653	66.6667	65.9864	65.9864
Mus musculus					100	67.3469	66.6667	66.6667	69.3878
Taeniopygia guttata						100	91.8367	92.517	71.4286
Cairina moschata							100	97.2789	69.3878
Gallus gallus								100	69.3878
Didelphis virginiana									100

Table A23. The parameter Similarity of the MUSCLE in the quantitative measurement of homology, assuming the *Nine beta globin protein sequences dataset* [3].

	Gorilla gorilla	Pan troglodytes	Homo sapiens	Rattus norvegicus	Mus musculus	Taeniopygia guttata	Cairina moschata	Gallus gallus	Didelphis virginiana
Gorilla gorilla	100	100	100	85.9504	90.0826	82.6446	84.2975	83.4711	80.9917
Pan troglodytes		100	96.8	83.2	87.2	80	81.6	80.8	78.4
Homo sapiens			100	87.0748	90.4762	82.9932	84.3537	83.6735	82.3129
Rattus norvegicus				100	92.517	79.5918	81.6327	80.9524	80.2721
Mus musculus					100	82.9932	84.3537	83.6735	81.6327
Taeniopygia guttata						100	95.9184	96.5986	84.3537
Cairina moschata							100	99.3197	84.3537
Gallus gallus								100	83.6735
Didelphis virginiana									100

Table A24. The parameter Alignment Score of the MUSCLE in the quantitative measurement of homology, assuming the *Nine beta globin protein sequences dataset* [3].

	Gorilla gorilla	Pan troglodytes	Homo sapiens	Rattus norvegicus	Mus musculus	Taeniopygia guttata	Cairina moschata	Gallus gallus	Didelphis virginiana
Gorilla gorilla	644	629.5	618.5	489.5	500.5	445.5	441.5	443.5	460.5
Pan troglodytes		661	610	481	492	437	433	435	446
Homo sapiens			780	630	637	556	552	554	583
Rattus norvegicus				773	686	516	534	532	540
Mus musculus					768	530	522	525	563
Taeniopygia guttata						774	730	734	576
Cairina moschata							778	763	562
Gallus gallus								775	566
Didelphis virginiana									786

Table A25. The parameter Identity of the T-Coffee in the quantitative measurement of homology, assuming the *Nine beta globin protein sequences dataset* [3].

	Gorilla gorilla	Pan troglodytes	Homo sapiens	Rattus norvegicus	Mus musculus	Taeniopygia guttata	Cairina moschata	Gallus gallus	Didelphis virginiana
Gorilla gorilla	121.4876	86.7769	85.9504	84.2975	97.5207	100	95.8678	84.2975	99.1736
Pan troglodytes		117.6	84	81.6	81.6	68.8	77.6	81.6	69.6
Homo sapiens			100	92.517	67.3469	59.1837	63.2653	91.8367	58.5034
Rattus norvegicus				100	66.6667	57.8231	65.9864	97.2789	57.1429
Mus musculus					100	65.9864	88.4354	66.6667	65.3061
Taeniopygia guttata						85.034	63.9456	57.8231	81.6327
Cairina moschata							100	66.6667	63.2653
Gallus gallus								100	57.1429
Didelphis virginiana									82.3129

Table A26. The parameter Similarity of the T-Coffee in the quantitative measurement of homology, assuming the *Nine beta globin protein sequences dataset* [3].

	Gorilla gorilla	Pan troglodytes	Homo sapiens	Rattus norvegicus	Mus musculus	Taeniopygia guttata	Cairina moschata	Gallus gallus	Didelphis virginiana
Gorilla gorilla	121.4876	100	100.8264	101.6529	109.9174	100	105.7851	102.4793	100
Pan troglodytes		117.6	99.2	98.4	96	78.4	94.4	99.2	78.4
Homo sapiens			100	96.5986	82.9932	68.0272	79.5918	95.9184	68.0272
Rattus norvegicus				100	83.6735	68.7075	80.9524	99.3197	68.7075
Mus musculus					100	74.1497	92.517	84.3537	74.1497
Taeniopygia guttata						85.034	70.7483	69.3878	82.3129
Cairina moschata							100	81.6327	70.7483
Gallus gallus								100	69.3878
Didelphis virginiana									82.3129

Table A27. The parameter Alignment Score of the T-Coffee in the quantitative measurement of homology, assuming the *Nine beta globin protein sequences dataset* [3].

	Gorilla gorilla	Pan troglodytes	Homo sapiens	Rattus norvegicus	Mus musculus	Taeniopygia guttata	Cairina moschata	Gallus gallus	Didelphis virginiana
Gorilla gorilla	780	583	556	554	637	610	630	552	618.5
Pan troglodytes		786	576	566	563	446	540	562	460.5
Homo sapiens			774	734	530	437	516	730	445.5
Rattus norvegicus				775	525	435	532	763	443.5
Mus musculus					768	492	686	522	500.5
Taeniopygia guttata						661	481	433	629.5
Cairina moschata							773	534	489.5
Gallus gallus								778	441.5
Didelphis virginiana									644

Table A28. The parameter Identity of the Kalign in the quantitative measurement of homology, assuming the *Nine beta globin protein sequences dataset* [3].

	Gorilla gorilla	Pan troglodytes	Homo sapiens	Rattus norvegicus	Mus musculus	Taeniopygia guttata	Cairina moschata	Gallus gallus	Didelphis virginiana
Gorilla gorilla	100	99.1736	99.1736	76.8595	79.3388	71.0744	69.4215	69.4215	71.9008
Pan troglodytes		100	96.8	75.2	77.6	69.6	68	68	68.8
Homo sapiens			100	78.9116	80.2721	70.7483	69.3878	69.3878	71.4286
Rattus norvegicus				100	88.4354	63.2653	66.6667	65.9864	65.9864
Mus musculus					100	67.3469	66.6667	66.6667	69.3878
Taeniopygia guttata						100	91.8367	92.517	71.4286
Cairina moschata							100	97.2789	69.3878
Gallus gallus								100	69.3878
Didelphis virginiana									100

Table A29. The parameter Similarity of the Kalign in the quantitative measurement of homology, assuming the *Nine beta globin protein sequences dataset* [3].

	Gorilla gorilla	Pan troglodytes	Homo sapiens	Rattus norvegicus	Mus musculus	Taeniopygia guttata	Cairina moschata	Gallus gallus	Didelphis virginiana
Gorilla gorilla	100	100	100	85.9504	90.0826	82.6446	84.2975	83.4711	80.9917
Pan troglodytes		100	96.8	83.2	87.2	80	81.6	80.8	78.4
Homo sapiens			100	87.0748	90.4762	82.9932	84.3537	83.6735	82.3129
Rattus norvegicus				100	92.517	79.5918	81.6327	80.9524	80.2721
Mus musculus					100	82.9932	84.3537	83.6735	81.6327
Taeniopygia guttata						100	95.9184	96.5986	84.3537
Cairina moschata							100	99.3197	84.3537
Gallus gallus								100	83.6735
Didelphis virginiana									100

Table A30. The parameter Alignment Score of the Kalign in the quantitative measurement of homology, assuming the *Nine beta globin protein sequences dataset* [3].

	Gorilla gorilla	Pan troglodytes	Homo sapiens	Rattus norvegicus	Mus musculus	Taeniopygia guttata	Cairina moschata	Gallus gallus	Didelphis virginiana
Gorilla gorilla	644	629.5	618.5	489.5	500.5	445.5	441.5	443.5	460.5
Pan troglodytes		661	610	481	492	437	433	435	446
Homo sapiens			780	630	637	556	552	554	583
Rattus norvegicus				773	686	516	534	532	540
Mus musculus					768	530	522	525	563
Taeniopygia guttata						774	730	734	576
Cairina moschata							778	763	562
Gallus gallus								775	566
Didelphis virginiana									786

Table A31. The parameter Identity of the MAFFT in the quantitative measurement of homology, assuming the *Nine beta globin protein sequences dataset* [3].

	Gorilla gorilla	Pan troglodytes	Homo sapiens	Rattus norvegicus	Mus musculus	Taeniopygia guttata	Cairina moschata	Gallus gallus	Didelphis virginiana
Gorilla gorilla	100	99.1736	99.1736	76.8595	79.3388	71.0744	69.4215	69.4215	71.9008
Pan troglodytes		100	96.8	75.2	77.6	69.6	68	68	68.8
Homo sapiens			100	78.9116	80.2721	70.7483	69.3878	69.3878	71.4286
Rattus norvegicus				100	88.4354	63.2653	66.6667	65.9864	65.9864
Mus musculus					100	67.3469	66.6667	66.6667	69.3878
Taeniopygia guttata						100	91.8367	92.517	71.4286
Cairina moschata							100	97.2789	69.3878
Gallus gallus								100	69.3878
Didelphis virginiana									100

Table A32. The parameter Similarity of the MAFFT in the quantitative measurement of homology, assuming the *Nine beta globin protein sequences dataset* [3].

	Gorilla gorilla	Pan troglodytes	Homo sapiens	Rattus norvegicus	Mus musculus	Taeniopygia guttata	Cairina moschata	Gallus gallus	Didelphis virginiana
Gorilla gorilla	100	100	100	85.9504	90.0826	82.6446	84.2975	83.4711	80.9917
Pan troglodytes		100	96.8	83.2	87.2	80	81.6	80.8	78.4
Homo sapiens			100	87.0748	90.4762	82.9932	84.3537	83.6735	82.3129
Rattus norvegicus				100	92.517	79.5918	81.6327	80.9524	80.2721
Mus musculus					100	82.9932	84.3537	83.6735	81.6327
Taeniopygia guttata						100	95.9184	96.5986	84.3537
Cairina moschata							100	99.3197	84.3537
Gallus gallus								100	83.6735
Didelphis virginiana									100

Table A33. The parameter Alignment Score of the MAFFT in the quantitative measurement of homology, assuming the *Nine beta globin protein sequences dataset* [3].

	Gorilla gorilla	Pan troglodytes	Homo sapiens	Rattus norvegicus	Mus musculus	Taeniopygia guttata	Cairina moschata	Gallus gallus	Didelphis virginiana
Gorilla gorilla	644	629.5	618.5	489.5	500.5	445.5	441.5	443.5	460.5
Pan troglodytes		661	610	481	492	437	433	435	446
Homo sapiens			780	630	637	556	552	554	583
Rattus norvegicus				773	686	516	534	532	540
Mus musculus					768	530	522	525	563
Taeniopygia guttata						774	730	734	576
Cairina moschata							778	763	562
Gallus gallus								775	566
Didelphis virginiana									786

Table A34. The parameter Sensitivity (SEN) of the HELIOS method with referencing the Smith-Waterman in the accuracy measurement of classification output, assuming the *Nine beta globin protein sequences dataset* [3].

	Gorilla gorilla	Pan troglodytes	Homo sapiens	Rattus norvegicus	Mus musculus	Taeniopygia guttata	Cairina moschata	Gallus gallus	Didelphis virginiana
Gorilla gorilla	1	0.98347	1	1	1	1	1	1	1
Pan troglodytes		1	0.98347	0.98347	0.98347	0.97521	0.96694	0.96694	0.96694
Homo sapiens			1	1	1	1	0.95918	1	1
Rattus norvegicus				1	1	0.91156	0.93197	0.93197	1
Mus musculus					1	0.95918	0.95918	0.95918	1
Taeniopygia guttata						1	1	1	1
Cairina moschata							1	1	0.96599
Gallus gallus								1	0.96599
Didelphis virginiana									1

Table A35. The parameter Specification (Spec) of the HELIOS method with referencing the Smith-Waterman in the accuracy measurement of classification output, assuming the *Nine beta globin protein sequences dataset* [3].

	Gorilla gorilla	Pan troglodytes	Homo sapiens	Rattus norvegicus	Mus musculus	Taeniopygia guttata	Cairina moschata	Gallus gallus	Didelphis virginiana
Gorilla gorilla	1	0.99987	1	1	1	1	1	1	1
Pan troglodytes		1	0.99989	0.99989	0.99989	0.99984	0.99978	0.99978	0.99984
Homo sapiens			1	1	1	1	0.99986	1	1
Rattus norvegicus				1	1	0.99972	0.99981	0.99981	1
Mus musculus					1	0.99986	0.99986	0.99986	1
Taeniopygia guttata						1	1	1	1
Cairina moschata							1	1	0.99986
Gallus gallus								1	0.99986
Didelphis virginiana									1

Table A36. The parameter Accuracy (Acc) of the HELIOS method with referencing the Smith-Waterman in the accuracy measurement of classification output, assuming the *Nine beta globin protein sequences dataset* [3].

	Gorilla gorilla	Pan troglodytes	Homo sapiens	Rattus norvegicus	Mus musculus	Taeniopygia guttata	Cairina moschata	Gallus gallus	Didelphis virginiana
Gorilla gorilla	1	0.99974	1	1	1	1	1	1	1
Pan troglodytes		1	0.99978	0.99978	0.99978	0.99967	0.99956	0.99956	0.99962
Homo sapiens			1	1	1	1	0.99958	1	1
Rattus norvegicus				1	1	0.99912	0.99935	0.99935	1
Mus musculus					1	0.99958	0.99958	0.99958	1
Taeniopygia guttata						1	1	1	1
Cairina moschata							1	1	0.99963
Gallus gallus								1	0.99963
Didelphis virginiana									1

Table A37. The parameter Positive Predictive Value (PPV) of the HELIOS method with referencing the Smith-Waterman in the accuracy measurement of classification output, assuming the *Nine beta globin protein sequences dataset* [3].

	Gorilla gorilla	Pan troglodytes	Homo sapiens	Rattus norvegicus	Mus musculus	Taeniopygia guttata	Cairina moschata	Gallus gallus	Didelphis virginiana
Gorilla gorilla	1	0.98347	1	1	1	1	1	1	1
Pan troglodytes		1	0.98347	0.98347	0.98347	0.97521	0.96694	0.96694	0.975
Homo sapiens			1	1	1	1	0.97917	1	1
Rattus norvegicus				1	1	0.95714	0.97163	0.97163	1
Mus musculus					1	0.97917	0.97917	0.97917	1
Taeniopygia guttata						1	1	1	1
Cairina moschata							1	1	0.97931
Gallus gallus								1	0.97931
Didelphis virginiana									1

Table A38. The parameter Negative Predictive Value (NPV) of the HELIOS method with referencing the Smith-Waterman in the accuracy measurement of classification output, assuming the *Nine beta globin protein sequences dataset* [3].

	Gorilla gorilla	Pan troglodytes	Homo sapiens	Rattus norvegicus	Mus musculus	Taeniopygia guttata	Cairina moschata	Gallus gallus	Didelphis virginiana
Gorilla gorilla	1	0.99987	1	1	1	1	1	1	1
Pan troglodytes		1	0.99989	0.99989	0.99989	0.99984	0.99978	0.99978	0.99978
Homo sapiens			1	1	1	1	0.99972	1	1
Rattus norvegicus				1	1	0.99939	0.99953	0.99953	1
Mus musculus					1	0.99972	0.99972	0.99972	1
Taeniopygia guttata						1	1	1	1
Cairina moschata							1	1	0.99977
Gallus gallus								1	0.99977
Didelphis virginiana									1

Table A39. The parameter Matthew's Coefficient Correlation (MCC) of the HELIOS method with referencing the Smith-Waterman in the accuracy measurement of classification output, assuming the *Nine beta globin protein sequences dataset* [3].

	Gorilla gorilla	Pan troglodytes	Homo sapiens	Rattus norvegicus	Mus musculus	Taeniopygia guttata	Cairina moschata	Gallus gallus	Didelphis virginiana
Gorilla gorilla	1	0.98334	1	1	1	1	1	1	1
Pan troglodytes		1	0.98336	0.98336	0.98336	0.97504	0.96672	0.96672	0.97077
Homo sapiens			1	1	1	1	0.96891	1	1
Rattus norvegicus				1	1	0.93364	0.95127	0.95127	1
Mus musculus					1	0.96891	0.96891	0.96891	1
Taeniopygia guttata						1	1	1	1
Cairina moschata							1	1	0.97244
Gallus gallus								1	0.97244
Didelphis virginiana									1

Table A40. The parameter Test's Accuracy (F-Score) of the HELIOS method with referencing the Smith-Waterman in the accuracy measurement of classification output, assuming the *Nine beta globin protein sequences dataset* [3].

	Gorilla gorilla	Pan troglodytes	Homo sapiens	Rattus norvegicus	Mus musculus	Taeniopygia guttata	Cairina moschata	Gallus gallus	Didelphis virginiana
Gorilla gorilla	1	0.98347	1	1	1	1	1	1	1
Pan troglodytes		1	0.98347	0.98347	0.98347	0.97521	0.96694	0.96694	0.97095
Homo sapiens			1	1	1	1	0.96907	1	1
Rattus norvegicus				1	1	0.9338	0.95139	0.95139	1
Mus musculus					1	0.96907	0.96907	0.96907	1
Taeniopygia guttata						1	1	1	1
Cairina moschata							1	1	0.9726
Gallus gallus								1	0.9726
Didelphis virginiana									1

Table A41. The parameter Sensitivity (SEN) of the HELIOS method with referencing the Needleman-Wunsch in the accuracy measurement of classification output, assuming the *Nine beta globin protein sequences dataset* [3].

	Gorilla gorilla	Pan troglodytes	Homo sapiens	Rattus norvegicus	Mus musculus	Taeniopygia guttata	Cairina moschata	Gallus gallus	Didelphis virginiana
Gorilla gorilla	1	0.98347	1	1	1	1	1	1	1
Pan troglodytes		1	0.98347	0.98347	0.98347	0.97521	0.96694	0.96694	0.96694
Homo sapiens			1	1	1	1	0.95918	1	1
Rattus norvegicus				1	1	0.91156	0.93197	0.93197	1
Mus musculus					1	0.95918	0.95918	0.95918	1
Taeniopygia guttata						1	1	1	1
Cairina moschata							1	1	0.96599
Gallus gallus								1	0.96599
Didelphis virginiana									1

Table A42. The parameter Specification (Spec) of the HELIOS method with referencing the Needleman-Wunsch in the accuracy measurement of classification output, assuming the *Nine beta globin protein sequences dataset* [3].

	Gorilla gorilla	Pan troglodytes	Homo sapiens	Rattus norvegicus	Mus musculus	Taeniopygia guttata	Cairina moschata	Gallus gallus	Didelphis virginiana
Gorilla gorilla	1	0.99987	1	1	1	1	1	1	1
Pan troglodytes		1	0.99987	0.99987	0.99987	0.9998	0.99973	0.99973	0.9998
Homo sapiens			1	1	1	1	0.99986	1	1
Rattus norvegicus				1	1	0.99972	0.99981	0.99981	1
Mus musculus					1	0.99986	0.99986	0.99986	1
Taeniopygia guttata						1	1	1	1
Cairina moschata							1	1	0.99986
Gallus gallus								1	0.99986
Didelphis virginiana									1

Table A43. The parameter Accuracy (Acc) of the HELIOS method with referencing the Needleman-Wunsch in the accuracy measurement of classification output, assuming the *Nine beta globin protein sequences dataset* [3].

	Gorilla gorilla	Pan troglodytes	Homo sapiens	Rattus norvegicus	Mus musculus	Taeniopygia guttata	Cairina moschata	Gallus gallus	Didelphis virginiana
Gorilla gorilla	1	0.99974	1	1	1	1	1	1	1
Pan troglodytes		1	0.99974	0.99974	0.99974	0.9996	0.99947	0.99947	0.99954
Homo sapiens			1	1	1	1	0.99958	1	1
Rattus norvegicus				1	1	0.99912	0.99935	0.99935	1
Mus musculus					1	0.99958	0.99958	0.99958	1
Taeniopygia guttata						1	1	1	1
Cairina moschata							1	1	0.99963
Gallus gallus								1	0.99963
Didelphis virginiana									1

Table A44. The parameter Positive Predictive Value (PPV) of the HELIOS method with referencing the Needleman-Wunsch in the accuracy measurement of classification output, assuming the *Nine beta globin protein sequences dataset* [3].

	Gorilla gorilla	Pan troglodytes	Homo sapiens	Rattus norvegicus	Mus musculus	Taeniopygia guttata	Cairina moschata	Gallus gallus	Didelphis virginiana
Gorilla gorilla	1	0.98347	1	1	1	1	1	1	1
Pan troglodytes		1	0.98347	0.98347	0.98347	0.97521	0.96694	0.96694	0.975
Homo sapiens			1	1	1	1	0.97917	1	1
Rattus norvegicus				1	1	0.95714	0.97163	0.97163	1
Mus musculus					1	0.97917	0.97917	0.97917	1
Taeniopygia guttata						1	1	1	1
Cairina moschata							1	1	0.97931
Gallus gallus								1	0.97931
Didelphis virginiana									1

Table A45. The parameter Negative Predictive Value (NPV) of the HELIOS method with referencing the Needleman-Wunsch in the accuracy measurement of classification output, assuming the *Nine beta globin protein sequences dataset* [3].

	Gorilla gorilla	Pan troglodytes	Homo sapiens	Rattus norvegicus	Mus musculus	Taeniopygia guttata	Cairina moschata	Gallus gallus	Didelphis virginiana
Gorilla gorilla	1	0.99987	1	1	1	1	1	1	1
Pan troglodytes		1	0.99987	0.99987	0.99987	0.9998	0.99973	0.99973	0.99973
Homo sapiens			1	1	1	1	0.99972	1	1
Rattus norvegicus				1	1	0.99939	0.99953	0.99953	1
Mus musculus					1	0.99972	0.99972	0.99972	1
Taeniopygia guttata						1	1	1	1
Cairina moschata							1	1	0.99977
Gallus gallus								1	0.99977
Didelphis virginiana									1

Table A46. The parameter Matthew's Coefficient Correlation (MCC) of the HELIOS method with referencing the Needleman-Wunsch in the accuracy measurement of classification output, assuming the *Nine beta globin protein sequences dataset* [3].

	Gorilla gorilla	Pan troglodytes	Homo sapiens	Rattus norvegicus	Mus musculus	Taeniopygia guttata	Cairina moschata	Gallus gallus	Didelphis virginiana
Gorilla gorilla	1	0.98334	1	1	1	1	1	1	1
Pan troglodytes		1	0.98334	0.98334	0.98334	0.97501	0.96668	0.96668	0.97073
Homo sapiens			1	1	1	1	0.96891	1	1
Rattus norvegicus				1	1	0.93364	0.95127	0.95127	1
Mus musculus					1	0.96891	0.96891	0.96891	1
Taeniopygia guttata						1	1	1	1
Cairina moschata							1	1	0.97244
Gallus gallus								1	0.97244
Didelphis virginiana									1

Table A47. The parameter Test's Accuracy (F-Score) of the HELIOS method with referencing the Needleman-Wunsch in the accuracy measurement of classification output, assuming the *Nine beta globin protein sequences dataset* [3].

	Gorilla gorilla	Pan troglodytes	Homo sapiens	Rattus norvegicus	Mus musculus	Taeniopygia guttata	Cairina moschata	Gallus gallus	Didelphis virginiana
Gorilla gorilla	1	0.98347	1	1	1	1	1	1	1
Pan troglodytes		1	0.98347	0.98347	0.98347	0.97521	0.96694	0.96694	0.97095
Homo sapiens			1	1	1	1	0.96907	1	1
Rattus norvegicus				1	1	0.9338	0.95139	0.95139	1
Mus musculus					1	0.96907	0.96907	0.96907	1
Taeniopygia guttata						1	1	1	1
Cairina moschata							1	1	0.9726
Gallus gallus								1	0.9726
Didelphis virginiana									1

Table A48. The parameter Sensitivity (SEN) of the HELIOS method with referencing the BLAST in the accuracy measurement of classification output, assuming the *Nine beta globin protein sequences dataset* [3].

	Gorilla gorilla	Pan troglodytes	Homo sapiens	Rattus norvegicus	Mus musculus	Taeniopygia guttata	Cairina moschata	Gallus gallus	Didelphis virginiana
Gorilla gorilla	1	1	1	1	1	1	1	1	1
Pan troglodytes		1	1	1	1	0.99174	1	1	0.97521
Homo sapiens			1	1	1	1	0.95918	1	1
Rattus norvegicus				1	1	0.91156	0.93197	0.93197	1
Mus musculus					1	0.95918	0.95918	0.95918	1
Taeniopygia guttata						1	1	1	1
Cairina moschata							1	1	0.96599
Gallus gallus								1	0.96599
Didelphis virginiana									1

Table A49. The parameter Specification (Spec) of the HELIOS method with referencing the BLAST in the accuracy measurement of classification output, assuming the *Nine beta globin protein sequences dataset* [3].

	Gorilla gorilla	Pan troglodytes	Homo sapiens	Rattus norvegicus	Mus musculus	Taeniopygia guttata	Cairina moschata	Gallus gallus	Didelphis virginiana
Gorilla gorilla	1	1	1	1	1	1	1	1	1
Pan troglodytes		1	1	1	1	0.99995	1	1	0.99989
Homo sapiens			1	1	1	1	0.99986	1	1
Rattus norvegicus				1	1	0.99972	0.99981	0.99981	1
Mus musculus					1	0.99986	0.99986	0.99986	1
Taeniopygia guttata						1	1	1	1
Cairina moschata							1	1	0.99986
Gallus gallus								1	0.99986
Didelphis virginiana									1

Table A50. The parameter Accuracy (Acc) of the HELIOS method with referencing the BLAST in the accuracy measurement of classification output, assuming the *Nine beta globin protein sequences dataset* [3].

	Gorilla gorilla	Pan troglodytes	Homo sapiens	Rattus norvegicus	Mus musculus	Taeniopygia guttata	Cairina moschata	Gallus gallus	Didelphis virginiana
Gorilla gorilla	1	1	1	1	1	1	1	1	1
Pan troglodytes		1	1	1	1	0.99989	1	1	0.99973
Homo sapiens			1	1	1	1	0.99958	1	1
Rattus norvegicus				1	1	0.99912	0.99935	0.99935	1
Mus musculus					1	0.99958	0.99958	0.99958	1
Taeniopygia guttata						1	1	1	1
Cairina moschata							1	1	0.99963
Gallus gallus								1	0.99963
Didelphis virginiana									1

Table A51. The parameter Positive Predictive Value (PPV) of the HELIOS method with referencing the BLAST in the accuracy measurement of classification output, assuming the *Nine beta globin protein sequences dataset* [3].

	Gorilla gorilla	Pan troglodytes	Homo sapiens	Rattus norvegicus	Mus musculus	Taeniopygia guttata	Cairina moschata	Gallus gallus	Didelphis virginiana
Gorilla gorilla	1	1	1	1	1	1	1	1	1
Pan troglodytes		1	1	1	1	0.99174	1	1	0.98333
Homo sapiens			1	1	1	1	0.97917	1	1
Rattus norvegicus				1	1	0.95714	0.97163	0.97163	1
Mus musculus					1	0.97917	0.97917	0.97917	1
Taeniopygia guttata						1	1	1	1
Cairina moschata							1	1	0.97931
Gallus gallus								1	0.97931
Didelphis virginiana									1

Table A52. The parameter Negative Predictive Value (NPV) of the HELIOS method with referencing the BLAST in the accuracy measurement of classification output, assuming the *Nine beta globin protein sequences dataset* [3].

	Gorilla gorilla	Pan troglodytes	Homo sapiens	Rattus norvegicus	Mus musculus	Taeniopygia guttata	Cairina moschata	Gallus gallus	Didelphis virginiana
Gorilla gorilla	1	1	1	1	1	1	1	1	1
Pan troglodytes		1	1	1	1	0.99995	1	1	0.99984
Homo sapiens			1	1	1	1	0.99972	1	1
Rattus norvegicus				1	1	0.99939	0.99953	0.99953	1
Mus musculus					1	0.99972	0.99972	0.99972	1
Taeniopygia guttata						1	1	1	1
Cairina moschata							1	1	0.99977
Gallus gallus								1	0.99977
Didelphis virginiana									1

Table A53. The parameter Matthew's Coefficient Correlation (MCC) of the HELIOS method with referencing the BLAST in the accuracy measurement of classification output, assuming the *Nine beta globin protein sequences dataset* [3].

	Gorilla gorilla	Pan troglodytes	Homo sapiens	Rattus norvegicus	Mus musculus	Taeniopygia guttata	Cairina moschata	Gallus gallus	Didelphis virginiana
Gorilla gorilla	1	1	1	1	1	1	1	1	1
Pan troglodytes		1	1	1	1	0.99168	1	1	0.97912
Homo sapiens			1	1	1	1	0.96891	1	1
Rattus norvegicus				1	1	0.93364	0.95127	0.95127	1
Mus musculus					1	0.96891	0.96891	0.96891	1
Taeniopygia guttata						1	1	1	1
Cairina moschata							1	1	0.97244
Gallus gallus								1	0.97244
Didelphis virginiana									1

Table A54. The parameter Test's Accuracy (F-Score) of the HELIOS method with referencing the BLAST in the accuracy measurement of classification output, assuming the *Nine beta globin protein sequences dataset* [3].

	Gorilla gorilla	Pan troglodytes	Homo sapiens	Rattus norvegicus	Mus musculus	Taeniopygia guttata	Cairina moschata	Gallus gallus	Didelphis virginiana
Gorilla gorilla	1	1	1	1	1	1	1	1	1
Pan troglodytes		1	1	1	1	0.99174	1	1	0.97925
Homo sapiens			1	1	1	1	0.96907	1	1
Rattus norvegicus				1	1	0.9338	0.95139	0.95139	1
Mus musculus					1	0.96907	0.96907	0.96907	1
Taeniopygia guttata						1	1	1	1
Cairina moschata							1	1	0.9726
Gallus gallus								1	0.9726
Didelphis virginiana									1

Table A55. The parameter Sensitivity (SEN) of the HELIOS method with referencing the ClustalW in the accuracy measurement of classification output, assuming the *Nine beta globin protein sequences dataset* [3].

	Gorilla gorilla	Pan troglodytes	Homo sapiens	Rattus norvegicus	Mus musculus	Taeniopygia guttata	Cairina moschata	Gallus gallus	Didelphis virginiana
Gorilla gorilla	1	0.98347	1	1	1	1	1	1	1
Pan troglodytes		1	0.98347	0.98347	0.98347	0.99174	0.98347	0.98347	0.96694
Homo sapiens			1	1	1	1	0.95918	1	1
Rattus norvegicus				1	1	0.91156	0.93197	0.93197	1
Mus musculus					1	0.95918	0.95918	0.95918	1
Taeniopygia guttata						1	1	1	1
Cairina moschata							1	1	0.96599
Gallus gallus								1	0.96599
Didelphis virginiana									1

Table A56. The parameter Specification (Spec) of the HELIOS method with referencing the ClustalW in the accuracy measurement of classification output, assuming the *Nine beta globin protein sequences dataset* [3].

	Gorilla gorilla	Pan troglodytes	Homo sapiens	Rattus norvegicus	Mus musculus	Taeniopygia guttata	Cairina moschata	Gallus gallus	Didelphis virginiana
Gorilla gorilla	1	0.99987	1	1	1	1	1	1	1
Pan troglodytes		1	0.99989	0.99989	0.99989	0.99995	0.99989	0.99989	0.99984
Homo sapiens			1	1	1	1	0.99986	1	1
Rattus norvegicus				1	1	0.99972	0.99981	0.99981	1
Mus musculus					1	0.99986	0.99986	0.99986	1
Taeniopygia guttata						1	1	1	1
Cairina moschata							1	1	0.99986
Gallus gallus								1	0.99986
Didelphis virginiana									1

Table A57. The parameter Accuracy (Acc) of the HELIOS method with referencing the ClustalW in the accuracy measurement of classification output, assuming the *Nine beta globin protein sequences dataset* [3].

	Gorilla gorilla	Pan troglodytes	Homo sapiens	Rattus norvegicus	Mus musculus	Taeniopygia guttata	Cairina moschata	Gallus gallus	Didelphis virginiana
Gorilla gorilla	1	0.99974	1	1	1	1	1	1	1
Pan troglodytes		1	0.99978	0.99978	0.99978	0.99989	0.99978	0.99978	0.99962
Homo sapiens			1	1	1	1	0.99958	1	1
Rattus norvegicus				1	1	0.99912	0.99935	0.99935	1
Mus musculus					1	0.99958	0.99958	0.99958	1
Taeniopygia guttata						1	1	1	1
Cairina moschata							1	1	0.99963
Gallus gallus								1	0.99963
Didelphis virginiana									1

Table A58. The parameter Positive Predictive Value (PPV) of the HELIOS method with referencing the ClustalW in the accuracy measurement of classification output, assuming the *Nine beta globin protein sequences dataset* [3].

	Gorilla gorilla	Pan troglodytes	Homo sapiens	Rattus norvegicus	Mus musculus	Taeniopygia guttata	Cairina moschata	Gallus gallus	Didelphis virginiana
Gorilla gorilla	1	0.98347	1	1	1	1	1	1	1
Pan troglodytes		1	0.98347	0.98347	0.98347	0.99174	0.98347	0.98347	0.975
Homo sapiens			1	1	1	1	0.97917	1	1
Rattus norvegicus				1	1	0.95714	0.97163	0.97163	1
Mus musculus					1	0.97917	0.97917	0.97917	1
Taeniopygia guttata						1	1	1	1
Cairina moschata							1	1	0.97931
Gallus gallus								1	0.97931
Didelphis virginiana									1

Table A59. The parameter Negative Predictive Value (NPV) of the HELIOS method with referencing the ClustalW in the accuracy measurement of classification output, assuming the *Nine beta globin protein sequences dataset* [3].

	Gorilla gorilla	Pan troglodytes	Homo sapiens	Rattus norvegicus	Mus musculus	Taeniopygia guttata	Cairina moschata	Gallus gallus	Didelphis virginiana
Gorilla gorilla	1	0.99987	1	1	1	1	1	1	1
Pan troglodytes		1	0.99989	0.99989	0.99989	0.99995	0.99989	0.99989	0.99978
Homo sapiens			1	1	1	1	0.99972	1	1
Rattus norvegicus				1	1	0.99939	0.99953	0.99953	1
Mus musculus					1	0.99972	0.99972	0.99972	1
Taeniopygia guttata						1	1	1	1
Cairina moschata							1	1	0.99977
Gallus gallus								1	0.99977
Didelphis virginiana									1

Table A60. The parameter Matthew's Coefficient Correlation (MCC) of the HELIOS method with referencing the ClustalW in the accuracy measurement of classification output, assuming the *Nine beta globin protein sequences dataset* [3].

	Gorilla gorilla	Pan troglodytes	Homo sapiens	Rattus norvegicus	Mus musculus	Taeniopygia guttata	Cairina moschata	Gallus gallus	Didelphis virginiana
Gorilla gorilla	1	0.98334	1	1	1	1	1	1	1
Pan troglodytes		1	0.98336	0.98336	0.98336	0.99168	0.98336	0.98336	0.97077
Homo sapiens			1	1	1	1	0.96891	1	1
Rattus norvegicus				1	1	0.93364	0.95127	0.95127	1
Mus musculus					1	0.96891	0.96891	0.96891	1
Taeniopygia guttata						1	1	1	1
Cairina moschata							1	1	0.97244
Gallus gallus								1	0.97244
Didelphis virginiana									1

Table A61. The parameter Test's Accuracy (F-Score) of the HELIOS method with referencing the ClustalW in the accuracy measurement of classification output, assuming the *Nine beta globin protein sequences dataset* [3].

	Gorilla gorilla	Pan troglodytes	Homo sapiens	Rattus norvegicus	Mus musculus	Taeniopygia guttata	Cairina moschata	Gallus gallus	Didelphis virginiana
Gorilla gorilla	1	0.98347	1	1	1	1	1	1	1
Pan troglodytes		1	0.98347	0.98347	0.98347	0.99174	0.98347	0.98347	0.97095
Homo sapiens			1	1	1	1	0.96907	1	1
Rattus norvegicus				1	1	0.9338	0.95139	0.95139	1
Mus musculus					1	0.96907	0.96907	0.96907	1
Taeniopygia guttata						1	1	1	1
Cairina moschata							1	1	0.9726
Gallus gallus								1	0.9726
Didelphis virginiana									1

Table A62. The parameter Sensitivity (SEN) of the HELIOS method with referencing the ClustalΩ in the accuracy measurement of classification output, assuming the *Nine beta globin protein sequences dataset* [3].

	Gorilla gorilla	Pan troglodytes	Homo sapiens	Rattus norvegicus	Mus musculus	Taeniopygia guttata	Cairina moschata	Gallus gallus	Didelphis virginiana
Gorilla gorilla	1	0.99174	1	1	1	1	1	1	1
Pan troglodytes		1	0.99174	0.99174	0.99174	1	0.99174	0.99174	0.97521
Homo sapiens			1	1	1	1	0.95918	1	1
Rattus norvegicus				1	1	0.91156	0.93197	0.93197	1
Mus musculus					1	0.95918	0.95918	0.95918	1
Taeniopygia guttata						1	1	1	1
Cairina moschata							1	1	0.96599
Gallus gallus								1	0.96599
Didelphis virginiana									1

Table A63. The parameter Specification (Spec) of the HELIOS method with referencing the ClustalΩ in the accuracy measurement of classification output, assuming the *Nine beta globin protein sequences dataset* [3].

	Gorilla gorilla	Pan troglodytes	Homo sapiens	Rattus norvegicus	Mus musculus	Taeniopygia guttata	Cairina moschata	Gallus gallus	Didelphis virginiana
Gorilla gorilla	1	0.99993	1	1	1	1	1	1	1
Pan troglodytes		1	0.99995	0.99995	0.99995	1	0.99995	0.99995	0.99989
Homo sapiens			1	1	1	1	0.99986	1	1
Rattus norvegicus				1	1	0.99972	0.99981	0.99981	1
Mus musculus					1	0.99986	0.99986	0.99986	1
Taeniopygia guttata						1	1	1	1
Cairina moschata							1	1	0.99986
Gallus gallus								1	0.99986
Didelphis virginiana									1

Table A64. The parameter Accuracy (Acc) of the HELIOS method with referencing the ClustalΩ in the accuracy measurement of classification output, assuming the *Nine beta globin protein sequences dataset* [3].

	Gorilla gorilla	Pan troglodytes	Homo sapiens	Rattus norvegicus	Mus musculus	Taeniopygia guttata	Cairina moschata	Gallus gallus	Didelphis virginiana
Gorilla gorilla	1	0.99987	1	1	1	1	1	1	1
Pan troglodytes		1	0.99989	0.99989	0.99989	1	0.99989	0.99989	0.99973
Homo sapiens			1	1	1	1	0.99958	1	1
Rattus norvegicus				1	1	0.99912	0.99935	0.99935	1
Mus musculus					1	0.99958	0.99958	0.99958	1
Taeniopygia guttata						1	1	1	1
Cairina moschata							1	1	0.99963
Gallus gallus								1	0.99963
Didelphis virginiana									1

Table A65. The parameter Positive Predictive Value (PPV) of the HELIOS method with referencing the ClustalΩ in the accuracy measurement of classification output, assuming the *Nine beta globin protein sequences dataset* [3].

	Gorilla gorilla	Pan troglodytes	Homo sapiens	Rattus norvegicus	Mus musculus	Taeniopygia guttata	Cairina moschata	Gallus gallus	Didelphis virginiana
Gorilla gorilla	1	0.99174	1	1	1	1	1	1	1
Pan troglodytes		1	0.99174	0.99174	0.99174	1	0.99174	0.99174	0.98333
Homo sapiens			1	1	1	1	0.97917	1	1
Rattus norvegicus				1	1	0.95714	0.97163	0.97163	1
Mus musculus					1	0.97917	0.97917	0.97917	1
Taeniopygia guttata						1	1	1	1
Cairina moschata							1	1	0.97931
Gallus gallus								1	0.97931
Didelphis virginiana									1

Table A66. The parameter Negative Predictive Value (NPV) of the HELIOS method with referencing the ClustalΩ in the accuracy measurement of classification output, assuming the *Nine beta globin protein sequences dataset* [3].

	Gorilla gorilla	Pan troglodytes	Homo sapiens	Rattus norvegicus	Mus musculus	Taeniopygia guttata	Cairina moschata	Gallus gallus	Didelphis virginiana
Gorilla gorilla	1	0.99993	1	1	1	1	1	1	1
Pan troglodytes		1	0.99995	0.99995	0.99995	1	0.99995	0.99995	0.99984
Homo sapiens			1	1	1	1	0.99972	1	1
Rattus norvegicus				1	1	0.99939	0.99953	0.99953	1
Mus musculus					1	0.99972	0.99972	0.99972	1
Taeniopygia guttata						1	1	1	1
Cairina moschata							1	1	0.99977
Gallus gallus								1	0.99977
Didelphis virginiana									1

Table A67. The parameter Matthew's Coefficient Correlation (MCC) of the HELIOS method with referencing the ClustalΩ in the accuracy measurement of classification output, assuming the *Nine beta globin protein sequences dataset* [3].

	Gorilla gorilla	Pan troglodytes	Homo sapiens	Rattus norvegicus	Mus musculus	Taeniopygia guttata	Cairina moschata	Gallus gallus	Didelphis virginiana
Gorilla gorilla	1	0.99167	1	1	1	1	1	1	1
Pan troglodytes		1	0.99168	0.99168	0.99168	1	0.99168	0.99168	0.97912
Homo sapiens			1	1	1	1	0.96891	1	1
Rattus norvegicus				1	1	0.93364	0.95127	0.95127	1
Mus musculus					1	0.96891	0.96891	0.96891	1
Taeniopygia guttata						1	1	1	1
Cairina moschata							1	1	0.97244
Gallus gallus								1	0.97244
Didelphis virginiana									1

Table A68. The parameter Test's Accuracy (F-Score) of the HELIOS method with referencing the ClustalΩ in the accuracy measurement of classification output, assuming the *Nine beta globin protein sequences dataset* [3].

	Gorilla gorilla	Pan troglodytes	Homo sapiens	Rattus norvegicus	Mus musculus	Taeniopygia guttata	Cairina moschata	Gallus gallus	Didelphis virginiana
Gorilla gorilla	1	0.99174	1	1	1	1	1	1	1
Pan troglodytes		1	0.99174	0.99174	0.99174	1	0.99174	0.99174	0.97925
Homo sapiens			1	1	1	1	0.96907	1	1
Rattus norvegicus				1	1	0.9338	0.95139	0.95139	1
Mus musculus					1	0.96907	0.96907	0.96907	1
Taeniopygia guttata						1	1	1	1
Cairina moschata							1	1	0.9726
Gallus gallus								1	0.9726
Didelphis virginiana									1

Table A69. The parameter Sensitivity (SEN) of the HELIOS method with referencing the MUSCLE in the accuracy measurement of classification output, assuming the *Nine beta globin protein sequences dataset* [3].

	Gorilla gorilla	Pan troglodytes	Homo sapiens	Rattus norvegicus	Mus musculus	Taeniopygia guttata	Cairina moschata	Gallus gallus	Didelphis virginiana
Gorilla gorilla	1	0.98347	1	1	1	1	1	1	1
Pan troglodytes		1	0.98347	0.98347	0.98347	0.99174	0.98347	0.98347	0.96694
Homo sapiens			1	1	1	1	0.95918	1	1
Rattus norvegicus				1	1	0.91156	0.93197	0.93197	1
Mus musculus					1	0.95918	0.95918	0.95918	1
Taeniopygia guttata						1	1	1	1
Cairina moschata							1	1	0.96599
Gallus gallus								1	0.96599
Didelphis virginiana									1

Table A70. The parameter Specification (Spec) of the HELIOS method with referencing the MUSCLE in the accuracy measurement of classification output, assuming the *Nine beta globin protein sequences dataset* [3].

	Gorilla gorilla	Pan troglodytes	Homo sapiens	Rattus norvegicus	Mus musculus	Taeniopygia guttata	Cairina moschata	Gallus gallus	Didelphis virginiana
Gorilla gorilla	1	0.99987	1	1	1	1	1	1	1
Pan troglodytes		1	0.99989	0.99989	0.99989	0.99995	0.99989	0.99989	0.99984
Homo sapiens			1	1	1	1	0.99986	1	1
Rattus norvegicus				1	1	0.99972	0.99981	0.99981	1
Mus musculus					1	0.99986	0.99986	0.99986	1
Taeniopygia guttata						1	1	1	1
Cairina moschata							1	1	0.99986
Gallus gallus								1	0.99986
Didelphis virginiana									1

Table A71. The parameter Accuracy (Acc) of the HELIOS method with referencing the MUSCLE in the accuracy measurement of classification output, assuming the *Nine beta globin protein sequences dataset* [3].

	Gorilla gorilla	Pan troglodytes	Homo sapiens	Rattus norvegicus	Mus musculus	Taeniopygia guttata	Cairina moschata	Gallus gallus	Didelphis virginiana
Gorilla gorilla	1	0.99974	1	1	1	1	1	1	1
Pan troglodytes		1	0.99978	0.99978	0.99978	0.99989	0.99978	0.99978	0.99962
Homo sapiens			1	1	1	1	0.99958	1	1
Rattus norvegicus				1	1	0.99912	0.99935	0.99935	1
Mus musculus					1	0.99958	0.99958	0.99958	1
Taeniopygia guttata						1	1	1	1
Cairina moschata							1	1	0.99963
Gallus gallus								1	0.99963
Didelphis virginiana									1

Table A72. The parameter Positive Predictive Value (PPV) of the HELIOS method with referencing the MUSCLE in the accuracy measurement of classification output, assuming the *Nine beta globin protein sequences dataset* [3].

	Gorilla gorilla	Pan troglodytes	Homo sapiens	Rattus norvegicus	Mus musculus	Taeniopygia guttata	Cairina moschata	Gallus gallus	Didelphis virginiana
Gorilla gorilla	1	0.98347	1	1	1	1	1	1	1
Pan troglodytes		1	0.98347	0.98347	0.98347	0.99174	0.98347	0.98347	0.975
Homo sapiens			1	1	1	1	0.97917	1	1
Rattus norvegicus				1	1	0.95714	0.97163	0.97163	1
Mus musculus					1	0.97917	0.97917	0.97917	1
Taeniopygia guttata						1	1	1	1
Cairina moschata							1	1	0.97931
Gallus gallus								1	0.97931
Didelphis virginiana									1

Table A73. The parameter Negative Predictive Value (NPV) of the HELIOS method with referencing the MUSCLE in the accuracy measurement of classification output, assuming the *Nine beta globin protein sequences dataset* [3].

	Gorilla gorilla	Pan troglodytes	Homo sapiens	Rattus norvegicus	Mus musculus	Taeniopygia guttata	Cairina moschata	Gallus gallus	Didelphis virginiana
Gorilla gorilla	1	0.99987	1	1	1	1	1	1	1
Pan troglodytes		1	0.99989	0.99989	0.99989	0.99995	0.99989	0.99989	0.99978
Homo sapiens			1	1	1	1	0.99972	1	1
Rattus norvegicus				1	1	0.99939	0.99953	0.99953	1
Mus musculus					1	0.99972	0.99972	0.99972	1
Taeniopygia guttata						1	1	1	1
Cairina moschata							1	1	0.99977
Gallus gallus								1	0.99977
Didelphis virginiana									1

Table A74. The parameter Matthew's Coefficient Correlation (MCC) of the HELIOS method with referencing the MUSCLE in the accuracy measurement of classification output, assuming the *Nine beta globin protein sequences dataset* [3].

	Gorilla gorilla	Pan troglodytes	Homo sapiens	Rattus norvegicus	Mus musculus	Taeniopygia guttata	Cairina moschata	Gallus gallus	Didelphis virginiana
Gorilla gorilla	1	0.98334	1	1	1	1	1	1	1
Pan troglodytes		1	0.98336	0.98336	0.98336	0.99168	0.98336	0.98336	0.97077
Homo sapiens			1	1	1	1	0.96891	1	1
Rattus norvegicus				1	1	0.93364	0.95127	0.95127	1
Mus musculus					1	0.96891	0.96891	0.96891	1
Taeniopygia guttata						1	1	1	1
Cairina moschata							1	1	0.97244
Gallus gallus								1	0.97244
Didelphis virginiana									1

Table A75. The parameter Test's Accuracy (F-Score) of the HELIOS method with referencing the MUSCLE in the accuracy measurement of classification output, assuming the *Nine beta globin protein sequences dataset* [3].

	Gorilla gorilla	Pan troglodytes	Homo sapiens	Rattus norvegicus	Mus musculus	Taeniopygia guttata	Cairina moschata	Gallus gallus	Didelphis virginiana
Gorilla gorilla	1	0.98347	1	1	1	1	1	1	1
Pan troglodytes		1	0.98347	0.98347	0.98347	0.99174	0.98347	0.98347	0.97095
Homo sapiens			1	1	1	1	0.96907	1	1
Rattus norvegicus				1	1	0.9338	0.95139	0.95139	1
Mus musculus					1	0.96907	0.96907	0.96907	1
Taeniopygia guttata						1	1	1	1
Cairina moschata							1	1	0.9726
Gallus gallus								1	0.9726
Didelphis virginiana									1

Table A76. The parameter Sensitivity (SEN) of the HELIOS method with referencing the T-Coffee in the accuracy measurement of classification output, assuming the *Nine beta globin protein sequences dataset* [3].

	Gorilla gorilla	Pan troglodytes	Homo sapiens	Rattus norvegicus	Mus musculus	Taeniopygia guttata	Cairina moschata	Gallus gallus	Didelphis virginiana
Gorilla gorilla	0.82313	0.21769	0.82313	0.82313	0.82313	0.2562	0.82313	0.82313	1
Pan troglodytes		0.85034	0.21769	0.21769	0.21769	0.2562	0.21769	0.21769	0.2562
Homo sapiens			1	1	1	0.2562	0.95918	1	1
Rattus norvegicus				1	1	0.19835	0.93197	0.93197	1
Mus musculus					1	0.2562	0.95918	0.95918	1
Taeniopygia guttata						1	0.2562	0.2562	0.2562
Cairina moschata							1	1	0.95868
Gallus gallus								1	0.95868
Didelphis virginiana									1

Table A77. The parameter Specification (Spec) of the HELIOS method with referencing the T-Coffee in the accuracy measurement of classification output, assuming the *Nine beta globin protein sequences dataset* [3].

	Gorilla gorilla	Pan troglodytes	Homo sapiens	Rattus norvegicus	Mus musculus	Taeniopygia guttata	Cairina moschata	Gallus gallus	Didelphis virginiana
Gorilla gorilla	1	0.99585	1	1	1	0.99507	1	1	1
Pan troglodytes		1	0.99585	0.99585	0.99585	0.99507	0.99585	0.99585	0.99496
Homo sapiens			1	1	1	0.99485	0.99986	1	1
Rattus norvegicus				1	1	0.99469	0.99981	0.99981	1
Mus musculus					1	0.99485	0.99986	0.99986	1
Taeniopygia guttata						1	0.99485	0.99485	0.994
Cairina moschata							1	1	0.99983
Gallus gallus								1	0.99983
Didelphis virginiana									1

Table A78. The parameter Accuracy (Acc) of the HELIOS method with referencing the T-Coffee in the accuracy measurement of classification output, assuming the *Nine beta globin protein sequences dataset* [3].

	Gorilla gorilla	Pan troglodytes	Homo sapiens	Rattus norvegicus	Mus musculus	Taeniopygia guttata	Cairina moschata	Gallus gallus	Didelphis virginiana
Gorilla gorilla	0.9988	0.99056	0.9988	0.9988	0.9988	0.9902	0.9988	0.9988	1
Pan troglodytes		0.99898	0.99056	0.99056	0.99056	0.9902	0.99056	0.99056	0.98994
Homo sapiens			1	1	1	0.98999	0.99958	1	1
Rattus norvegicus				1	1	0.98944	0.99935	0.99935	1
Mus musculus					1	0.98999	0.99958	0.99958	1
Taeniopygia guttata						1	0.98999	0.98999	0.9881
Cairina moschata							1	1	0.99955
Gallus gallus								1	0.99955
Didelphis virginiana									1

Table A79. The parameter Positive Predictive Value (PPV) of the HELIOS method with referencing the T-Coffee in the accuracy measurement of classification output, assuming the *Nine beta globin protein sequences dataset* [3].

	Gorilla gorilla	Pan troglodytes	Homo sapiens	Rattus norvegicus	Mus musculus	Taeniopygia guttata	Cairina moschata	Gallus gallus	Didelphis virginiana
Gorilla gorilla	1	0.26446	1	1	1	0.2562	1	1	1
Pan troglodytes		1	0.26446	0.26446	0.26446	0.2562	0.26446	0.26446	0.25833
Homo sapiens			1	1	1	0.248	0.97917	1	1
Rattus norvegicus				1	1	0.19835	0.97163	0.97163	1
Mus musculus					1	0.248	0.97917	0.97917	1
Taeniopygia guttata						1	0.248	0.248	0.2562
Cairina moschata							1	1	0.97479
Gallus gallus								1	0.97479
Didelphis virginiana									1

Table A80. The parameter Negative Predictive Value (NPV) of the HELIOS method with referencing the T-Coffee in the accuracy measurement of classification output, assuming the *Nine beta globin protein sequences dataset* [3].

	Gorilla gorilla	Pan troglodytes	Homo sapiens	Rattus norvegicus	Mus musculus	Taeniopygia guttata	Cairina moschata	Gallus gallus	Didelphis virginiana
Gorilla gorilla	0.99879	0.99465	0.99879	0.99879	0.99879	0.99507	0.99879	0.99879	1
Pan troglodytes		0.99898	0.99465	0.99465	0.99465	0.99507	0.99465	0.99465	0.99491
Homo sapiens			1	1	1	0.99507	0.99972	1	1
Rattus norvegicus				1	1	0.99469	0.99953	0.99953	1
Mus musculus					1	0.99507	0.99972	0.99972	1
Taeniopygia guttata						1	0.99507	0.99507	0.994
Cairina moschata							1	1	0.99972
Gallus gallus								1	0.99972
Didelphis virginiana									1

Table A81. The parameter Matthew's Coefficient Correlation (MCC) of the HELIOS method with referencing the T-Coffee in the accuracy measurement of classification output, assuming the *Nine beta globin protein sequences dataset* [3].

	Gorilla gorilla	Pan troglodytes	Homo sapiens	Rattus norvegicus	Mus musculus	Taeniopygia guttata	Cairina moschata	Gallus gallus	Didelphis virginiana
Gorilla gorilla	0.90672	0.23522	0.90672	0.90672	0.90672	0.25127	0.90672	0.90672	1
Pan troglodytes		0.92167	0.23522	0.23522	0.23522	0.25127	0.23522	0.23522	0.2522
Homo sapiens			1	1	1	0.24703	0.96891	1	1
Rattus norvegicus				1	1	0.19303	0.95127	0.95127	1
Mus musculus					1	0.24703	0.96891	0.96891	1
Taeniopygia guttata						1	0.24703	0.24703	0.2502
Cairina moschata							1	1	0.96647
Gallus gallus								1	0.96647
Didelphis virginiana									1

Table A82. The parameter Test's Accuracy (F-Score) of the HELIOS method with referencing the T-Coffee in the accuracy measurement of classification output, assuming the *Nine beta globin protein sequences dataset* [3].

	Gorilla gorilla	Pan troglodytes	Homo sapiens	Rattus norvegicus	Mus musculus	Taeniopygia guttata	Cairina moschata	Gallus gallus	Didelphis virginiana
Gorilla gorilla	0.90299	0.23881	0.90299	0.90299	0.90299	0.2562	0.90299	0.90299	1
Pan troglodytes		0.91912	0.23881	0.23881	0.23881	0.2562	0.23881	0.23881	0.25726
Homo sapiens			1	1	1	0.25203	0.96907	1	1
Rattus norvegicus				1	1	0.19835	0.95139	0.95139	1
Mus musculus					1	0.25203	0.96907	0.96907	1
Taeniopygia guttata						1	0.25203	0.25203	0.2562
Cairina moschata							1	1	0.96667
Gallus gallus								1	0.96667
Didelphis virginiana									1

Table A83. The parameter Sensitivity (SEN) of the HELIOS method with referencing the Kalign in the accuracy measurement of classification output, assuming the *Nine beta globin protein sequences dataset* [3].

	Gorilla gorilla	Pan troglodytes	Homo sapiens	Rattus norvegicus	Mus musculus	Taeniopygia guttata	Cairina moschata	Gallus gallus	Didelphis virginiana
Gorilla gorilla	1	1	1	1	1	1	1	1	1
Pan troglodytes		1	1	1	1	0.99174	1	1	0.97521
Homo sapiens			1	1	1	1	0.95918	1	1
Rattus norvegicus				1	1	0.91156	0.93197	0.93197	1
Mus musculus					1	0.95918	0.95918	0.95918	1
Taeniopygia guttata						1	1	1	1
Cairina moschata							1	1	0.96599
Gallus gallus								1	0.96599
Didelphis virginiana									1

Table A84. The parameter Specification (Spec) of the HELIOS method with referencing the Kalign in the accuracy measurement of classification output, assuming the *Nine beta globin protein sequences dataset* [3].

	Gorilla gorilla	Pan troglodytes	Homo sapiens	Rattus norvegicus	Mus musculus	Taeniopygia guttata	Cairina moschata	Gallus gallus	Didelphis virginiana
Gorilla gorilla	1	1	1	1	1	1	1	1	1
Pan troglodytes		1	1	1	1	0.99995	1	1	0.99989
Homo sapiens			1	1	1	1	0.99986	1	1
Rattus norvegicus				1	1	0.99972	0.99981	0.99981	1
Mus musculus					1	0.99986	0.99986	0.99986	1
Taeniopygia guttata						1	1	1	1
Cairina moschata							1	1	0.99986
Gallus gallus								1	0.99986
Didelphis virginiana									1

Table A85. The parameter Accuracy (Acc) of the HELIOS method with referencing the Kalign in the accuracy measurement of classification output, assuming the *Nine beta globin protein sequences dataset* [3].

	Gorilla gorilla	Pan troglodytes	Homo sapiens	Rattus norvegicus	Mus musculus	Taeniopygia guttata	Cairina moschata	Gallus gallus	Didelphis virginiana
Gorilla gorilla	1	1	1	1	1	1	1	1	1
Pan troglodytes		1	1	1	1	0.99989	1	1	0.99973
Homo sapiens			1	1	1	1	0.99958	1	1
Rattus norvegicus				1	1	0.99912	0.99935	0.99935	1
Mus musculus					1	0.99958	0.99958	0.99958	1
Taeniopygia guttata						1	1	1	1
Cairina moschata							1	1	0.99963
Gallus gallus								1	0.99963
Didelphis virginiana									1

Table A86. The parameter Positive Predictive Value (PPV) of the HELIOS method with referencing the Kalign in the accuracy measurement of classification output, assuming the *Nine beta globin protein sequences dataset* [3].

	Gorilla gorilla	Pan troglodytes	Homo sapiens	Rattus norvegicus	Mus musculus	Taeniopygia guttata	Cairina moschata	Gallus gallus	Didelphis virginiana
Gorilla gorilla	1	1	1	1	1	1	1	1	1
Pan troglodytes		1	1	1	1	0.99174	1	1	0.98333
Homo sapiens			1	1	1	1	0.97917	1	1
Rattus norvegicus				1	1	0.95714	0.97163	0.97163	1
Mus musculus					1	0.97917	0.97917	0.97917	1
Taeniopygia guttata						1	1	1	1
Cairina moschata							1	1	0.97931
Gallus gallus								1	0.97931
Didelphis virginiana									1

Table A87. The parameter Negative Predictive Value (NPV) of the HELIOS method with referencing the Kalign in the accuracy measurement of classification output, assuming the *Nine beta globin protein sequences dataset* [3].

	Gorilla gorilla	Pan troglodytes	Homo sapiens	Rattus norvegicus	Mus musculus	Taeniopygia guttata	Cairina moschata	Gallus gallus	Didelphis virginiana
Gorilla gorilla	1	1	1	1	1	1	1	1	1
Pan troglodytes		1	1	1	1	0.99995	1	1	0.99984
Homo sapiens			1	1	1	1	0.99972	1	1
Rattus norvegicus				1	1	0.99939	0.99953	0.99953	1
Mus musculus					1	0.99972	0.99972	0.99972	1
Taeniopygia guttata						1	1	1	1
Cairina moschata							1	1	0.99977
Gallus gallus								1	0.99977
Didelphis virginiana									1

Table A88. The parameter Matthew's Coefficient Correlation (MCC) of the HELIOS method with referencing the Kalign in the accuracy measurement of classification output, assuming the *Nine beta globin protein sequences dataset* [3].

	Gorilla gorilla	Pan troglodytes	Homo sapiens	Rattus norvegicus	Mus musculus	Taeniopygia guttata	Cairina moschata	Gallus gallus	Didelphis virginiana
Gorilla gorilla	1	1	1	1	1	1	1	1	1
Pan troglodytes		1	1	1	1	0.99168	1	1	0.97912
Homo sapiens			1	1	1	1	0.96891	1	1
Rattus norvegicus				1	1	0.93364	0.95127	0.95127	1
Mus musculus					1	0.96891	0.96891	0.96891	1
Taeniopygia guttata						1	1	1	1
Cairina moschata							1	1	0.97244
Gallus gallus								1	0.97244
Didelphis virginiana									1

Table A89. The parameter Test's Accuracy (F-Score) of the HELIOS method with referencing the Kalign in the accuracy measurement of classification output, assuming the *Nine beta globin protein sequences dataset* [3].

	Gorilla gorilla	Pan troglodytes	Homo sapiens	Rattus norvegicus	Mus musculus	Taeniopygia guttata	Cairina moschata	Gallus gallus	Didelphis virginiana
Gorilla gorilla	1	1	1	1	1	1	1	1	1
Pan troglodytes		1	1	1	1	0.99174	1	1	0.97925
Homo sapiens			1	1	1	1	0.96907	1	1
Rattus norvegicus				1	1	0.9338	0.95139	0.95139	1
Mus musculus					1	0.96907	0.96907	0.96907	1
Taeniopygia guttata						1	1	1	1
Cairina moschata							1	1	0.9726
Gallus gallus								1	0.9726
Didelphis virginiana									1

Table A90. The parameter Sensitivity (SEN) of the HELIOS method with referencing the MAFFT in the accuracy measurement of classification output, assuming the *Nine beta globin protein sequences dataset* [3].

	Gorilla gorilla	Pan troglodytes	Homo sapiens	Rattus norvegicus	Mus musculus	Taeniopygia guttata	Cairina moschata	Gallus gallus	Didelphis virginiana
Gorilla gorilla	1	0.98347	1	1	1	1	1	1	1
Pan troglodytes		1	0.98347	0.98347	0.98347	0.99174	0.98347	0.98347	0.96694
Homo sapiens			1	1	1	1	0.95918	1	1
Rattus norvegicus				1	1	0.91156	0.93197	0.93197	1
Mus musculus					1	0.95918	0.95918	0.95918	1
Taeniopygia guttata						1	1	1	1
Cairina moschata							1	1	0.96599
Gallus gallus								1	0.96599
Didelphis virginiana									1

Table A91. The parameter Specification (Spec) of the HELIOS method with referencing the MAFFT in the accuracy measurement of classification output, assuming the *Nine beta globin protein sequences dataset* [3].

	Gorilla gorilla	Pan troglodytes	Homo sapiens	Rattus norvegicus	Mus musculus	Taeniopygia guttata	Cairina moschata	Gallus gallus	Didelphis virginiana
Gorilla gorilla	1	0.99987	1	1	1	1	1	1	1
Pan troglodytes		1	0.99989	0.99989	0.99989	0.99995	0.99989	0.99989	0.99984
Homo sapiens			1	1	1	1	0.99986	1	1
Rattus norvegicus				1	1	0.99972	0.99981	0.99981	1
Mus musculus					1	0.99986	0.99986	0.99986	1
Taeniopygia guttata						1	1	1	1
Cairina moschata							1	1	0.99986
Gallus gallus								1	0.99986
Didelphis virginiana									1

Table A92. The parameter Accuracy (Acc) of the HELIOS method with referencing the MAFFT in the accuracy measurement of classification output, assuming the *Nine beta globin protein sequences dataset* [3].

	Gorilla gorilla	Pan troglodytes	Homo sapiens	Rattus norvegicus	Mus musculus	Taeniopygia guttata	Cairina moschata	Gallus gallus	Didelphis virginiana
Gorilla gorilla	1	0.99974	1	1	1	1	1	1	1
Pan troglodytes		1	0.99978	0.99978	0.99978	0.99989	0.99978	0.99978	0.99962
Homo sapiens			1	1	1	1	0.99958	1	1
Rattus norvegicus				1	1	0.99912	0.99935	0.99935	1
Mus musculus					1	0.99958	0.99958	0.99958	1
Taeniopygia guttata						1	1	1	1
Cairina moschata							1	1	0.99963
Gallus gallus								1	0.99963
Didelphis virginiana									1

Table A93. The parameter Positive Predictive Value (PPV) of the HELIOS method with referencing the MAFFT in the accuracy measurement of classification output, assuming the *Nine beta globin protein sequences dataset* [3].

	Gorilla gorilla	Pan troglodytes	Homo sapiens	Rattus norvegicus	Mus musculus	Taeniopygia guttata	Cairina moschata	Gallus gallus	Didelphis virginiana
Gorilla gorilla	1	0.98347	1	1	1	1	1	1	1
Pan troglodytes		1	0.98347	0.98347	0.98347	0.99174	0.98347	0.98347	0.975
Homo sapiens			1	1	1	1	0.97917	1	1
Rattus norvegicus				1	1	0.95714	0.97163	0.97163	1
Mus musculus					1	0.97917	0.97917	0.97917	1
Taeniopygia guttata						1	1	1	1
Cairina moschata							1	1	0.97931
Gallus gallus								1	0.97931
Didelphis virginiana									1

Table A94. The parameter Negative Predictive Value (NPV) of the HELIOS method with referencing the MAFFT in the accuracy measurement of classification output, assuming the *Nine beta globin protein sequences dataset* [3].

	Gorilla gorilla	Pan troglodytes	Homo sapiens	Rattus norvegicus	Mus musculus	Taeniopygia guttata	Cairina moschata	Gallus gallus	Didelphis virginiana
Gorilla gorilla	1	0.99987	1	1	1	1	1	1	1
Pan troglodytes		1	0.99989	0.99989	0.99989	0.99995	0.99989	0.99989	0.99978
Homo sapiens			1	1	1	1	0.99972	1	1
Rattus norvegicus				1	1	0.99939	0.99953	0.99953	1
Mus musculus					1	0.99972	0.99972	0.99972	1
Taeniopygia guttata						1	1	1	1
Cairina moschata							1	1	0.99977
Gallus gallus								1	0.99977
Didelphis virginiana									1

Table A95. The parameter Matthew's Coefficient Correlation (MCC) of the HELIOS method with referencing the MAFFT in the accuracy measurement of classification output, assuming the *Nine beta globin protein sequences dataset* [3].

	Gorilla gorilla	Pan troglodytes	Homo sapiens	Rattus norvegicus	Mus musculus	Taeniopygia guttata	Cairina moschata	Gallus gallus	Didelphis virginiana
Gorilla gorilla	1	0.98334	1	1	1	1	1	1	1
Pan troglodytes		1	0.98336	0.98336	0.98336	0.99168	0.98336	0.98336	0.97077
Homo sapiens			1	1	1	1	0.96891	1	1
Rattus norvegicus				1	1	0.93364	0.95127	0.95127	1
Mus musculus					1	0.96891	0.96891	0.96891	1
Taeniopygia guttata						1	1	1	1
Cairina moschata							1	1	0.97244
Gallus gallus								1	0.97244
Didelphis virginiana									1

Table A96. The parameter Test's Accuracy (F-Score) of the HELIOS method with referencing the MAFFT in the accuracy measurement of classification output, assuming the *Nine beta globin protein sequences dataset* [3].

	Gorilla gorilla	Pan troglodytes	Homo sapiens	Rattus norvegicus	Mus musculus	Taeniopygia guttata	Cairina moschata	Gallus gallus	Didelphis virginiana
Gorilla gorilla	1	0.98347	1	1	1	1	1	1	1
Pan troglodytes		1	0.98347	0.98347	0.98347	0.99174	0.98347	0.98347	0.97095
Homo sapiens			1	1	1	1	0.96907	1	1
Rattus norvegicus				1	1	0.9338	0.95139	0.95139	1
Mus musculus					1	0.96907	0.96907	0.96907	1
Taeniopygia guttata						1	1	1	1
Cairina moschata							1	1	0.9726
Gallus gallus								1	0.9726
Didelphis virginiana									1
