# Supplementary material for: HELIOS: High-speed sequence alignment in optics
Source: PLoS Comput Biol. 2022 Nov 21;18(11):e1010665. doi: 10.1371/journal.pcbi.1010665 (PMC9678324; doi:10.1371/journal.pcbi.1010665)
Supplement: S3 Text — (PDF) [file pcbi.1010665.s003.pdf]

HELIOS: High-Speed Sequence Alignment in Optics: S3 Text

EHSAN MALEKI[1], SAEEDEH AKBARI ROKN ABADI[1], AND SOMAYYEH KOOHI[1,*]

[1] Department of Computer Engineering, Sharif University of Technology, Azadi Ave., Tehran, Iran.
[*] Corresponding author: koohi@sharif.edu

Compiled October 20, 2022

In response to the imperfections of current sequence alignment methods, originated from the inherent serialism within their corresponding electrical systems, a few optical approaches for biological data comparison have been proposed recently. However, due to their low performance, raised from their inefficient coding scheme, this paper presents a novel all-optical high-throughput method for aligning DNA, RNA, and protein sequences, named HELIOS. The HELIOS method employs highly sophisticated operations to locate character matches, single or multiple mutations, and single or multiple indels within various biological sequences. On the other hand, the HELIOS optical architecture exploits high-speed processing and operational parallelism in optics, by adopting wavelength and polarization of optical beams. For evaluation, the functionality and accuracy of the HELIOS method are approved through behavioral and optical simulation studies, while its complexity and performance are estimated through analytical computation. The accuracy evaluations indicate that the HELIOS method achieves a precise pairwise alignment of two sequences, highly similar to those of Smith-Waterman, Needleman-Wunsch, BLAST, MUSCLE, ClustalW, ClustalΩ, T-Coffee, Kalign, and MAFFT. According to our performance evaluations, the HELIOS optical architecture outperforms all alternative electrical and optical algorithms in terms of processing time and memory requirement, relying on its highly sophisticated method and optical architecture. Moreover, the employed compact coding scheme highly escalates the number of input characters, and hence, it offers reduced time and space complexities, compared to the electrical and optical alternatives. It makes the HELIOS method and optical architecture highly applicable for biomedical applications.

1. ACCURACY EVALUATION

In order to comprehensively assess the accuracy of the HELIOS method, two statistical analyses are performed through simulation studies: 1) Quantitative measurement of homology [1], and 2) Accuracy measurement of classification output [2], compared to the well-known algorithms, briefly reported in Tables A1 and A2, respectively. In this manner, the *ND6 (NADH dehydrogenase subunit 6) protein of eight species dataset* [3] is assumed in this study, as represented in Table A3.

A. Quantitative measurement of homology

To perform quantitative measurement of homology [1], the parameters Identity, Similarity, and Alignment Score of the HELIOS outputs are calculated through simulation studies, as reported in Tables A4-A6, respectively, assuming the *ND6 (NADH dehydrogenase subunit 6) protein of eight species dataset* [3]. While the Identity reports the number of exactly matched characters of two sequences (in percentage), the Similarity measures the resemblance of two compared sequences. Specifically, regarding the physicochemical properties, the amino acids are categorized into six groups with different Similarity values; including GAVLI, FYW, STCM, KRH, DENQ, and P. As the third metric, the BLOSUM62 [4] substitution scoring matrix [4] is adopted to calculate the Alignment Score, with gap opening and extension penalties equal to -10 and -0.5, respectively.

For a comparative study, the values of Identity, Similarity, and Alignment Score of the quantitative measurement of homology is performed by various well-known algorithms to be compared to the those of HELIOS method, assuming *ND6 (NADH dehydrogenase subunit 6) protein of eight species dataset* [3]. It includes Smith-Waterman (SW) [5] reported in Tables A7-A9, Needleman-Wunsch (NW) [6] reported in Tables A10-A12, BLAST [7] reported in Tables A13-A15, ClustalW [8] reported in Tables A16-A18, Clustal-Omega [9] reported in Tables A19-A21, MUSCLE [9] reported in Tables A22-A24, T-Coffee [10] reported in Tables A25-A27, Kalign [11] reported in Tables A28-A30, and MAFFT [12] reported in Tables A31-A33.

Table A1. A brief report of the quantitative measurement of homology of the HELIOS method, compared to nine well-known algorithms, including SW, NW, BLAST, ClustalW, Clustal Omega, Muscle, T-Coffee, Kalign, and MAFFT. The parameters Identity, Similarity, and Alignment score are averaged and reported. The dataset used in this study is the *ND6 (NADH dehydrogenase subunit 6) protein of eight species dataset* [3].

	HELIOS	SW	NW	BLAST	MUSCLE	ClustalW	ClustalΩ	T-Coffee	Kalign	MAFFT
Identity	68.700	68.862	68.862	68.004	68.104	68.021	67.923	68.206	68.104	68.104
Similarity	79.808	81.581	81.531	81.174	81.191	81.108	80.878	81.035	81.191	81.386
Alignment Score	567.7	600.5	609.5	599.4	604.9	603.5	600.7	602.5	604.9	605.5

Table A2. A brief report of the accuracy measurement of classification output of the HELIOS method with referencing well-known algorithms, including SW, NW, BLAST, ClustalW, Clustal Omega, Muscle, T-Coffee, Kalign, and MAFFT. The parameters SEN, Spec, Acc, PPV, NPV, MCC, and F-Score are averaged and reported. The dataset used in this study is the *ND6 (NADH dehydrogenase subunit 6) protein of eight species dataset* [3].

	SW	NW	BLAST	MUSCLE	ClustalW	ClustalΩ	T-Coffee	Kalign	MAFFT
SEN	0.87985	0.61425	0.87743	0.88059	0.87992	0.88149	0.42407	0.88059	0.87897
Spec	0.99940	0.99786	0.99938	0.99941	0.99940	0.99941	0.99680	0.99941	0.99940
ACC	0.99872	0.99563	0.99869	0.99873	0.99872	0.99874	0.99354	0.99873	0.99871
PPV	0.89123	0.62125	0.88875	0.89298	0.89227	0.89387	0.42829	0.89298	0.89133
NPV	0.99931	0.99775	0.99930	0.99932	0.99931	0.99932	0.99670	0.99932	0.99931
MCC	0.88485	0.61553	0.88238	0.88609	0.88540	0.88699	0.42291	0.88609	0.88445
F-Score	0.88544	0.61700	0.88299	0.88667	0.88598	0.88757	0.42615	0.88667	0.88504

B. Accuracy measurement of classification output

Afterward, the accuracy measurement of the classification output [2] of the HELIOS method is addressed by calculating the values of Sensitivity (SEN), Specificity (Spec), Accuracy (ACC), Positive Predictive Value (PPV), Negative Predictive Value (NPV), Matthew's Coefficient Correlation (MCC), and Test's Accuracy (F-Score) in the simulation studies, according to Eq 5 to Eq 11, respectively.

As a comparative study, the accuracy measurement of the classification output of the HELIOS method is accomplished, assuming *ND6 (NADH dehydrogenase subunit 6) protein of eight species dataset* [3], and the corresponding metrics are calculated with considering Smith-Waterman [5] reported in Tables A34-A40, Needleman-Wunsch [6] reported in Tables A41-A47, ClustalW [8] reported in Tables A55-A61, Clustal-Omega [9] reported in Tables A62-A68, BLAST [7] reported in Tables A48-A54, MUSCLE [13] reported in Tables A69-A75, T-Coffee [10] reported in Tables A76-A82, Kalign [11] reported in Tables A83-A89, and MAFFT [12] reported in Tables A90-A96.

REFERENCES

1. D. S. Moss, S. Jelaska, and S. Pongor, *Essays in bioinformatics*, vol. 368 (IOS Press, 2005).
2. M. Hamada, H. Kiryu, W. Iwasaki, and K. Asai, "Generalized centroid estimators in bioinformatics," PloS one **6**, e16450 (2011).
3. X.-l. Xie, L.-f. Zheng, Y. Yu, L.-p. Liang, M.-c. Guo, J. Song, and Z.-f. Yuan, "Protein sequence analysis based on hydropathy profile of amino acids," J. Zhejiang Univ. Sci. B **13**, 152–158 (2012).
4. D. W. Mount, "Using blosum in sequence alignments," Cold Spring Harb. Protoc. **2008**, pdb.top39 (2008).
5. H. Zou, S. Tang, C. Yu, H. Fu, Y. Li, and W. Tang, "asw: accelerating smith–waterman algorithm on coupled cpu–gpu architecture," Int. J. Parallel Program. **47**, 388–402 (2019).
6. Y. Jararweh, M. Al-Ayyoub, M. Fakirah, L. Alawneh, and B. B. Gupta, "Improving the performance of the needleman-wunsch algorithm using parallelization and vectorization techniques," Multimed. Tools Appl. **78**, 3961–3977 (2019).
7. G. M. Boratyn, J. Thierry-Mieg, D. Thierry-Mieg, B. Busby, and T. L. Madden, "Magic-blast, an accurate rna-seq aligner for long and short reads," BMC bioinformatics **20**, 1–19 (2019).
8. D. Díaz, F. J. Esteban, P. Hernández, J. A. Caballero, A. Guevara, G. Dorado, and S. Gálvez, "Mc64-clustalwp2: A highly-parallel hybrid strategy to align multiple sequences in many-core architectures," PLOS ONE **9**, 1–12 (2014).
9. F. Sievers and D. G. Higgins, "Clustal omega for making accurate alignments of many protein sequences," Protein Sci. **27**, 135–145 (2018).
10. C. Notredame, D. G. Higgins, and J. Heringa, "T-coffee: a novel method for fast and accurate multiple sequence alignment11edited by j. thornton," J. Mol. Biol. **302**, 205–217 (2000).
11. T. Lassmann, "Kalign 3: multiple sequence alignment of large datasets," (2020).
12. J. Rozewicki, S. Li, K. M. Amada, D. M. Standley, and K. Katoh, "Mafft-dash: integrated protein sequence and structural alignment," Nucleic acids research **47**, W5–W10 (2019).
13. R. C. Edgar, "MUSCLE: multiple sequence alignment with high accuracy and high throughput," Nucleic Acids Res. **32**, 1792–1797 (2004).

Table A3. The list of input sequences, assuming the *ND6 (NADH dehydrogenase subunit 6) protein of eight species dataset* [3].

Name	Sequence
Homo sapiens	MMYAL FLLSV GLVMG FVGFS SKPSP IYGGL VLIVS GVVGC VIILN FGGGY MGLMV FLIYL GGMMV VFGYT TAMAI EEYPE AWGSG VEVLV SVLVG LAMEV GLVLW VKEYD GVVVV VNFNS VGSWM IYEGE GSGLI REDPI GAGAL YDYGR WLVVV TGWTL FVGVY IVIEI ARGN
Gorilla gorilla	MTYVL FLLSV GLVMG FVGFS SKPSP IYGGL VLIVS GVVGC AIILN CGGGY MGLMV FLIYL GGMMV VFGYT TAMAI GEYPE AWGSG VEVLV SVLVG LAMEV GLVLW VKEYD GVVVV VNFNN VGSWM IYEGE GSGLI REDPI GAGAL YDYGR WLVVV TGWTL FVGVY IVIEI ARGN
Pan troglodytes	MTYAL FLLSV SLVMG FVGFS SKPSP IYGGL VLIVS GVVGC AIILN YGGGY MGLMV FLIYL GGMMV VFGYT TAMAI EEYPE AWGSG VEVLV SVLVG LAMEV GLVLW VKGYD GMVVV VNFNS VGSWM IYEGE GPGLI REDPI GAGAL YDYGR WLVVV TGWTL FVGVY IVIEI ARGN
Osphranter robustus	MKMMV VFLFS ILLVF GFVAF ASKPS PVYGG LSLVV SGGLG CAIVV SLEDV FLGLI VFLIY LGGML VVFGY TAAMA TEEYP ESWVG NTVAL SMLLF TVIVE SAWYL MSGEV KVSMD IELFD IIGGY CVGQD YSGVS LLYGC GGWAL VLLGW ILFIT IYVVL EVVRG CN
Phoca vitulina	MMTYI VFILS IIFVV SFVGF SSKPS PIYGG LVLII SGAVG CGIVL SFGGS FLGLM VFLIY LGGML VVFGY TTAMA IEQYP EVWVS NKAVL GAFVM GLLSE LLLAC YILKD DEVDV VFEFN GMGDW VIYDT GDSGF FSEEA MGIAA LYSYG TWLVI VTGWS LLTGV LVIME VTRGN
Halichoerus grypus	MMTYI VFILS IIFVI SFVGF SSKPS PIYGG LVLII SGAVG CGIVL SFGGS FLGLM VFLIY LGGML VVFGY TTAMA TEQYP EVWVS NKAVL GAFVM GLLSE LLLAC YILKD DEVDA VFEFN GMGDW VIYDT GDSGF FSEEA MGIAA LYSYG TWLVI VTGWS LFIGV LVIME VTRGN
Rattus norvegicus	MTNYM FILSL LFLTG CLGLA LKPSP IYGGF GLIVS GCIGC LMVLG FGGSF LGLMV FLIYL GGMLV VFGYT TAMAT EEYPE TWGSN WFIFS FFVLG LFMEL VVFYL FSLNN KVELV DFDSL GDWLM YEIDD VGVML EGGIG VAAIY SCATW MMVVA GWSLF AGIFI IIEIT RD
Mus musculus	MNNYI FVLSS LFLVG CLGLA LKPSP IYGGL GLIVS GFVGC LMVLG FGGSF LGLMV FLIYL GGMLV VFGYT TAMAT EEYPE TWGSN WLILG FLVLG VIMEV FLICV LNYYD EVGVI NLDGL GDWLM YEVDD VGVML EGGIG VAAMY SCATW MMVVA GWSLF AGIFI IIEIT RD

Table A4. The parameter Identity of the HELIOS method in the quantitative measurement of homology, assuming the *ND6 (NADH dehydrogenase subunit 6) protein of eight species dataset* [3].

	Homo sapiens	Gorilla gorilla	Pan troglodytes	Osphranter robustus	Phoca vitulina	Halichoerus grypus	Rattus norvegicus	Mus musculus
Homo sapiens	100	96.5517	95.977	43.7126	59.1954	59.1954	52.3256	55.2326
Gorilla gorilla		100	95.4023	44.9102	57.4713	56.8966	50	54.0698
Pan troglodytes			100	43.1138	57.4713	56.8966	52.907	54.6512
Osphranter robustus				100	47.3054	47.9042	43.7126	40.1198
Phoca vitulina					100	97.1429	55.2326	56.3953
Halichoerus grypus						100	56.9767	59.3023
Rattus norvegicus							100	83.1395
Mus musculus								100

Table A5. The parameter Similarity of the HELIOS method in the quantitative measurement of homology, assuming the *ND6 (NADH dehydrogenase subunit 6) protein of eight species dataset* [3].

	Homo sapiens	Gorilla gorilla	Pan troglodytes	Osphranter robustus	Phoca vitulina	Halichoerus grypus	Rattus norvegicus	Mus musculus
Homo sapiens	100	98.2759	97.7011	61.0778	75.2874	77.0115	70.9302	72.093
Gorilla gorilla		100	95.977	62.8743	74.1379	74.7126	69.186	69.7674
Pan troglodytes			100	62.8743	75.2874	75.8621	70.3488	71.5116
Osphranter robustus				100	61.6766	64.0719	61.6766	57.485
Phoca vitulina					100	98.2857	70.3488	71.5116
Halichoerus grypus						100	71.5116	75
Rattus norvegicus							100	86.6279
Mus musculus								100

Table A6. The parameter Alignment Score of the HELIOS method in the quantitative measurement of homology, assuming the *ND6 (NADH dehydrogenase subunit 6) protein of eight species dataset* [3].

	Homo sapiens	Gorilla gorilla	Pan troglodytes	Osphranter robustus	Phoca vitulina	Halichoerus grypus	Rattus norvegicus	Mus musculus
Homo sapiens	898	866	864	230	512	521	436.5	438.5
Gorilla gorilla		904	855	245.5	495	495	436.5	411
Pan troglodytes			902	253	506	506	423.5	430.5
Osphranter robustus				854	228.5	262.5	213.5	198.5
Phoca vitulina					894	874	450	461
Halichoerus grypus						896	463.5	482
Rattus norvegicus							906	725.5
Mus musculus								902

Table A7. The parameter Identity of the Smith-Waterman in the quantitative measurement of homology, assuming the *ND6 (NADH dehydrogenase subunit 6) protein of eight species dataset* [3].

	Homo sapiens	Gorilla gorilla	Pan troglodytes	Osphranter robustus	Phoca vitulina	Halichoerus grypus	Rattus norvegicus	Mus musculus
Homo sapiens	100	96.5517	95.977	47.9042	58.6207	58.046	50.5814	52.907
Gorilla gorilla		100	95.4023	47.9042	58.046	57.4713	49.4186	51.7442
Pan troglodytes			100	47.9042	58.046	57.4713	50.5814	52.3256
Osphranter robustus				100	48.503	50.2994	44.9102	44.9102
Phoca vitulina					100	97.1429	54.6512	57.5581
Halichoerus grypus						100	55.814	58.1395
Rattus norvegicus							100	80.2326
Mus musculus								100

Table A8. The parameter Similarity of the Smith-Waterman in the quantitative measurement of homology, assuming the *ND6 (NADH dehydrogenase subunit 6) protein of eight species dataset* [3].

	Homo sapiens	Gorilla gorilla	Pan troglodytes	Osphranter robustus	Phoca vitulina	Halichoerus grypus	Rattus norvegicus	Mus musculus
Homo sapiens	100	98.2759	97.7011	68.2635	77.5862	78.1609	71.5116	72.093
Gorilla gorilla		100	95.977	67.6647	76.4368	77.0115	69.7674	70.9302
Pan troglodytes			100	67.6647	77.0115	77.5862	70.9302	71.5116
Osphranter robustus				100	68.2635	69.4611	65.2695	65.2695
Phoca vitulina					100	98.2857	72.6744	74.4186
Halichoerus grypus						100	74.4186	76.1628
Rattus norvegicus							100	86.6279
Mus musculus								100

Table A9. The parameter Alignment Score of the Smith-Waterman in the quantitative measurement of homology, assuming the *ND6 (NADH dehydrogenase subunit 6) protein of eight species dataset* [3].

	Homo sapiens	Gorilla gorilla	Pan troglodytes	Osphranter robustus	Phoca vitulina	Halichoerus grypus	Rattus norvegicus	Mus musculus
Homo sapiens	898	866	864	314.5	543	543	458	470
Gorilla gorilla		904	855	308.5	541	541	445	456
Pan troglodytes			902	324	534	534	462	468
Osphranter robustus				854	355.5	357.5	297.5	293
Phoca vitulina					894	874	484	515
Halichoerus grypus						896	492	523
Rattus norvegicus							906	746
Mus musculus								902

Table A10. The parameter Identity of the Needleman-Wunsch in the quantitative measurement of homology, assuming the *ND6 (NADH dehydrogenase subunit 6) protein of eight species dataset* [3].

	Homo sapiens	Gorilla gorilla	Pan troglodytes	Osphranter robustus	Phoca vitulina	Halichoerus grypus	Rattus norvegicus	Mus musculus
Homo sapiens	100	96.5517	95.977	47.9042	58.6207	58.046	50.5814	52.907
Gorilla gorilla		100	95.4023	47.9042	58.046	57.4713	49.4186	51.7442
Pan troglodytes			100	47.9042	58.046	57.4713	50.5814	52.3256
Osphranter robustus				100	48.503	50.2994	44.9102	44.9102
Phoca vitulina					100	97.1429	54.6512	57.5581
Halichoerus grypus						100	55.814	58.1395
Rattus norvegicus							100	80.2326
Mus musculus								100

Table A11. The parameter Similarity of the Needleman-Wunsch in the quantitative measurement of homology, assuming the *ND6 (NADH dehydrogenase subunit 6) protein of eight species dataset* [3].

	Homo sapiens	Gorilla gorilla	Pan troglodytes	Osphranter robustus	Phoca vitulina	Halichoerus grypus	Rattus norvegicus	Mus musculus
Homo sapiens	100	98.2759	97.7011	68.2635	77.5862	78.1609	71.5116	72.093
Gorilla gorilla		100	95.977	67.0659	76.4368	77.0115	69.7674	70.9302
Pan troglodytes			100	67.0659	77.0115	77.5862	70.9302	71.5116
Osphranter robustus				100	68.2635	69.4611	64.6707	65.2695
Phoca vitulina					100	98.2857	72.6744	74.4186
Halichoerus grypus						100	74.4186	76.1628
Rattus norvegicus							100	86.6279
Mus musculus								100

Table A12. The parameter Alignment Score of the Needleman-Wunsch in the quantitative measurement of homology, assuming the *ND6 (NADH dehydrogenase subunit 6) protein of eight species dataset* [3].

	Homo sapiens	Gorilla gorilla	Pan troglodytes	Osphranter robustus	Phoca vitulina	Halichoerus grypus	Rattus norvegicus	Mus musculus
Homo sapiens	898	866	864	336	553	553	469	481
Gorilla gorilla		904	855	331	551	551	456	467
Pan troglodytes			902	336	544	544	473	479
Osphranter robustus				854	366	370.5	324	320.5
Phoca vitulina					894	874	505	526
Halichoerus grypus						896	513	534
Rattus norvegicus							906	746
Mus musculus								902

Table A13. The parameter Identity of the BLAST in the quantitative measurement of homology, assuming the *ND6 (NADH dehydrogenase subunit 6) protein of eight species dataset* [3].

	Homo sapiens	Gorilla gorilla	Pan troglodytes	Osphranter robustus	Phoca vitulina	Halichoerus grypus	Rattus norvegicus	Mus musculus
Homo sapiens	100	96.5517	95.977	43.7126	58.6207	58.046	50	52.907
Gorilla gorilla		100	95.4023	43.7126	58.046	57.4713	48.2558	51.7442
Pan troglodytes			100	43.7126	57.4713	56.8966	48.8372	51.7442
Osphranter robustus				100	46.1078	47.9042	40.7186	43.1138
Phoca vitulina					100	97.1429	53.4884	57.5581
Halichoerus grypus						100	54.6512	58.1395
Rattus norvegicus							100	80.2326
Mus musculus								100

Table A14. The parameter Similarity of the BLAST in the quantitative measurement of homology, assuming the *ND6 (NADH dehydrogenase subunit 6) protein of eight species dataset* [3].

	Homo sapiens	Gorilla gorilla	Pan troglodytes	Osphranter robustus	Phoca vitulina	Halichoerus grypus	Rattus norvegicus	Mus musculus
Homo sapiens	100	98.2759	97.7011	68.8623	77.5862	78.1609	70.3488	72.093
Gorilla gorilla		100	95.977	68.2635	76.4368	77.0115	68.6047	70.9302
Pan troglodytes			100	68.2635	75.8621	76.4368	69.7674	70.9302
Osphranter robustus				100	67.0659	68.8623	61.0778	64.6707
Phoca vitulina					100	98.2857	71.5116	73.8372
Halichoerus grypus						100	73.2558	75.5814
Rattus norvegicus							100	86.6279
Mus musculus								100

Table A15. The parameter Alignment Score of the BLAST in the quantitative measurement of homology, assuming the *ND6 (NADH dehydrogenase subunit 6) protein of eight species dataset* [3].

	Homo sapiens	Gorilla gorilla	Pan troglodytes	Osphranter robustus	Phoca vitulina	Halichoerus grypus	Rattus norvegicus	Mus musculus
Homo sapiens	898	866	864	336.5	543	543	423	451
Gorilla gorilla		904	855	329.5	541	541	407	440
Pan troglodytes			902	333.5	540	540	418	446
Osphranter robustus				854	367.5	383.5	301.5	314.5
Phoca vitulina					894	874	487	512
Halichoerus grypus						896	497	522
Rattus norvegicus							906	746
Mus musculus								902

Table A16. The parameter Identity of the ClustalW in the quantitative measurement of homology, assuming the *ND6 (NADH dehydrogenase subunit 6) protein of eight species dataset* [3].

	Homo sapiens	Gorilla gorilla	Pan troglodytes	Osphranter robustus	Phoca vitulina	Halichoerus grypus	Rattus norvegicus	Mus musculus
Homo sapiens	100	96.5517	95.977	44.9102	58.6207	58.046	50	52.907
Gorilla gorilla		100	95.4023	44.9102	58.046	57.4713	48.8372	51.7442
Pan troglodytes			100	44.3114	57.4713	56.8966	49.4186	51.7442
Osphranter robustus				100	45.509	46.7066	40.7186	43.1138
Phoca vitulina					100	97.1429	54.0698	56.3953
Halichoerus grypus						100	55.2326	56.3953
Rattus norvegicus							100	80.2326
Mus musculus								100

Table A17. The parameter Similarity of the ClustalW in the quantitative measurement of homology, assuming the *ND6 (NADH dehydrogenase subunit 6) protein of eight species dataset* [3].

	Homo sapiens	Gorilla gorilla	Pan troglodytes	Osphranter robustus	Phoca vitulina	Halichoerus grypus	Rattus norvegicus	Mus musculus
Homo sapiens	100	98.2759	97.7011	68.8623	77.5862	78.1609	70.3488	72.093
Gorilla gorilla		100	95.977	68.2635	76.4368	77.0115	68.6047	70.9302
Pan troglodytes			100	67.6647	75.8621	76.4368	69.7674	70.9302
Osphranter robustus				100	66.4671	68.2635	61.6766	63.4731
Phoca vitulina					100	98.2857	71.5116	73.8372
Halichoerus grypus						100	73.2558	75.5814
Rattus norvegicus							100	86.6279
Mus musculus								100

Table A18. The parameter Alignment Score of the ClustalW in the quantitative measurement of homology, assuming the *ND6 (NADH dehydrogenase subunit 6) protein of eight species dataset* [3].

	Homo sapiens	Gorilla gorilla	Pan troglodytes	Osphranter robustus	Phoca vitulina	Halichoerus grypus	Rattus norvegicus	Mus musculus
Homo sapiens	898	866	864	349.5	543	543	442	470
Gorilla gorilla		904	855	343.5	541	541	429	456
Pan troglodytes			902	342.5	540	540	441	465
Osphranter robustus				854	368.5	382.5	312	314
Phoca vitulina					894	874	488	506
Halichoerus grypus						896	496	512
Rattus norvegicus							906	746
Mus musculus								902

Table A19. The parameter Identity of the ClustalΩ in the quantitative measurement of homology, assuming the *ND6 (NADH dehydrogenase subunit 6) protein of eight species dataset* [3].

	Homo sapiens	Gorilla gorilla	Pan troglodytes	Osphranter robustus	Phoca vitulina	Halichoerus grypus	Rattus norvegicus	Mus musculus
Homo sapiens	100	96.5517	95.977	44.9102	58.6207	58.046	50	52.907
Gorilla gorilla		100	95.4023	44.9102	58.046	57.4713	48.8372	51.7442
Pan troglodytes			100	44.3114	57.4713	56.8966	50	52.3256
Osphranter robustus				100	45.509	46.7066	40.1198	41.9162
Phoca vitulina					100	97.1429	52.907	55.814
Halichoerus grypus						100	54.0698	56.3953
Rattus norvegicus							100	80.2326
Mus musculus								100

Table A20. The parameter Similarity of the ClustalΩ in the quantitative measurement of homology, assuming the *ND6 (NADH dehydrogenase subunit 6) protein of eight species dataset* [3].

	Homo sapiens	Gorilla gorilla	Pan troglodytes	Osphranter robustus	Phoca vitulina	Halichoerus grypus	Rattus norvegicus	Mus musculus
Homo sapiens	100	98.2759	97.7011	68.2635	77.5862	78.1609	70.3488	72.093
Gorilla gorilla		100	95.977	67.6647	76.4368	77.0115	68.6047	70.9302
Pan troglodytes			100	67.0659	75.8621	76.4368	70.3488	71.5116
Osphranter robustus				100	66.4671	68.2635	60.479	61.6766
Phoca vitulina					100	98.2857	69.7674	73.2558
Halichoerus grypus						100	71.5116	75
Rattus norvegicus							100	86.6279
Mus musculus								100

Table A21. The parameter Alignment Score of the ClustalΩ in the quantitative measurement of homology, assuming the *ND6 (NADH dehydrogenase subunit 6) protein of eight species dataset* [3].

	Homo sapiens	Gorilla gorilla	Pan troglodytes	Osphranter robustus	Phoca vitulina	Halichoerus grypus	Rattus norvegicus	Mus musculus
Homo sapiens	898	866	864	345.5	543	543	442	470
Gorilla gorilla		904	855	339.5	541	541	429	456
Pan troglodytes			902	338.5	540	540	444	468
Osphranter robustus				854	362.5	376.5	297	292
Phoca vitulina					894	874	472	494
Halichoerus grypus						896	483	507
Rattus norvegicus							906	746
Mus musculus								902

Table A22. The parameter Identity of the MUSCLE in the quantitative measurement of homology, assuming the *ND6 (NADH dehydrogenase subunit 6) protein of eight species dataset* [3].

	Homo sapiens	Gorilla gorilla	Pan troglodytes	Osphranter robustus	Phoca vitulina	Halichoerus grypus	Rattus norvegicus	Mus musculus
Homo sapiens	100	96.5517	95.977	45.509	58.6207	58.046	50	52.907
Gorilla gorilla		100	95.4023	45.509	58.046	57.4713	48.8372	51.7442
Pan troglodytes			100	44.9102	57.4713	56.8966	49.4186	51.7442
Osphranter robustus				100	46.1078	47.3054	40.7186	43.1138
Phoca vitulina					100	97.1429	54.0698	56.3953
Halichoerus grypus						100	55.2326	56.3953
Rattus norvegicus							100	80.2326
Mus musculus								100

Table A23. The parameter Similarity of the MUSCLE in the quantitative measurement of homology, assuming the *ND6 (NADH dehydrogenase subunit 6) protein of eight species dataset* [3].

	Homo sapiens	Gorilla gorilla	Pan troglodytes	Osphranter robustus	Phoca vitulina	Halichoerus grypus	Rattus norvegicus	Mus musculus
Homo sapiens	100	98.2759	97.7011	69.4611	77.5862	78.1609	70.3488	72.093
Gorilla gorilla		100	95.977	68.8623	76.4368	77.0115	68.6047	70.9302
Pan troglodytes			100	68.2635	75.8621	76.4368	69.7674	70.9302
Osphranter robustus				100	67.0659	68.8623	61.6766	63.4731
Phoca vitulina					100	98.2857	71.5116	73.8372
Halichoerus grypus						100	73.2558	75.5814
Rattus norvegicus							100	86.6279
Mus musculus								100

Table A24. The parameter Alignment Score of the MUSCLE in the quantitative measurement of homology, assuming the *ND6 (NADH dehydrogenase subunit 6) protein of eight species dataset* [3].

	Homo sapiens	Gorilla gorilla	Pan troglodytes	Osphranter robustus	Phoca vitulina	Halichoerus grypus	Rattus norvegicus	Mus musculus
Homo sapiens	898	866	864	358.5	543	543	442	470
Gorilla gorilla		904	855	352.5	541	541	429	456
Pan troglodytes			902	351.5	540	540	441	465
Osphranter robustus				854	380.5	394.5	312	314
Phoca vitulina					894	874	488	506
Halichoerus grypus						896	496	512
Rattus norvegicus							906	746
Mus musculus								902

Table A25. The parameter Identity of the T-Coffee in the quantitative measurement of homology, assuming the *ND6 (NADH dehydrogenase subunit 6) protein of eight species dataset* [3].

	Homo sapiens	Gorilla gorilla	Pan troglodytes	Osphranter robustus	Phoca vitulina	Halichoerus grypus	Rattus norvegicus	Mus musculus
Homo sapiens	98.8506	52.8736	54.023	41.9162	48.8506	48.2759	80.2326	50
Gorilla gorilla		100.5747	97.7011	46.1078	57.4713	58.046	56.3953	59.3023
Pan troglodytes			100.5747	47.9042	56.8966	57.4713	56.9767	58.7209
Osphranter robustus				100	44.9102	45.509	44.3114	45.509
Phoca vitulina					99.4286	94.8571	51.7442	97.093
Halichoerus grypus						99.4286	51.7442	97.6744
Rattus norvegicus							100	52.907
Mus musculus								101.1628

Table A26. The parameter Similarity of the T-Coffee in the quantitative measurement of homology, assuming the *ND6 (NADH dehydrogenase subunit 6) protein of eight species dataset* [3].

	Homo sapiens	Gorilla gorilla	Pan troglodytes	Osphranter robustus	Phoca vitulina	Halichoerus grypus	Rattus norvegicus	Mus musculus
Homo sapiens	98.8506	69.5402	71.2644	62.8743	68.9655	67.8161	86.6279	70.3488
Gorilla gorilla		100.5747	98.8506	66.4671	75.8621	76.4368	73.8372	78.4884
Pan troglodytes			100.5747	68.2635	76.4368	77.0115	75.5814	79.0698
Osphranter robustus				100	67.0659	67.6647	62.8743	68.2635
Phoca vitulina					99.4286	95.4286	70.9302	98.8372
Halichoerus grypus						99.4286	70.9302	99.4186
Rattus norvegicus							100	72.093
Mus musculus								101.1628

Table A27. The parameter Alignment Score of the T-Coffee in the quantitative measurement of homology, assuming the *ND6 (NADH dehydrogenase subunit 6) protein of eight species dataset* [3].

	Homo sapiens	Gorilla gorilla	Pan troglodytes	Osphranter robustus	Phoca vitulina	Halichoerus grypus	Rattus norvegicus	Mus musculus
Homo sapiens	906	479	490	324	441	429	746	442
Gorilla gorilla		894	874	358	540	541	501	543
Pan troglodytes			896	377	540	541	514	543
Osphranter robustus				854	339	337	318	344
Phoca vitulina					902	855	465	864
Halichoerus grypus						904	456	866
Rattus norvegicus							902	470
Mus musculus								898

Table A28. The parameter Identity of the Kalign in the quantitative measurement of homology, assuming the *ND6 (NADH dehydrogenase subunit 6) protein of eight species dataset* [3].

	Homo sapiens	Gorilla gorilla	Pan troglodytes	Osphranter robustus	Phoca vitulina	Halichoerus grypus	Rattus norvegicus	Mus musculus
Homo sapiens	100	96.5517	95.977	45.509	58.6207	58.046	50	52.907
Gorilla gorilla		100	95.4023	45.509	58.046	57.4713	48.8372	51.7442
Pan troglodytes			100	44.9102	57.4713	56.8966	49.4186	51.7442
Osphranter robustus				100	46.1078	47.3054	40.7186	43.1138
Phoca vitulina					100	97.1429	54.0698	56.3953
Halichoerus grypus						100	55.2326	56.3953
Rattus norvegicus							100	80.2326
Mus musculus								100

Table A29. The parameter Similarity of the Kalign in the quantitative measurement of homology, assuming the *ND6 (NADH dehydrogenase subunit 6) protein of eight species dataset* [3].

	Homo sapiens	Gorilla gorilla	Pan troglodytes	Osphranter robustus	Phoca vitulina	Halichoerus grypus	Rattus norvegicus	Mus musculus
Homo sapiens	100	98.2759	97.7011	69.4611	77.5862	78.1609	70.3488	72.093
Gorilla gorilla		100	95.977	68.8623	76.4368	77.0115	68.6047	70.9302
Pan troglodytes			100	68.2635	75.8621	76.4368	69.7674	70.9302
Osphranter robustus				100	67.0659	68.8623	61.6766	63.4731
Phoca vitulina					100	98.2857	71.5116	73.8372
Halichoerus grypus						100	73.2558	75.5814
Rattus norvegicus							100	86.6279
Mus musculus								100

Table A30. The parameter Alignment Score of the Kalign in the quantitative measurement of homology, assuming the *ND6 (NADH dehydrogenase subunit 6) protein of eight species dataset* [3].

	Homo sapiens	Gorilla gorilla	Pan troglodytes	Osphranter robustus	Phoca vitulina	Halichoerus grypus	Rattus norvegicus	Mus musculus
Homo sapiens	898	866	864	358.5	543	543	442	470
Gorilla gorilla		904	855	352.5	541	541	429	456
Pan troglodytes			902	351.5	540	540	441	465
Osphranter robustus				854	380.5	394.5	312	314
Phoca vitulina					894	874	488	506
Halichoerus grypus						896	496	512
Rattus norvegicus							906	746
Mus musculus								902

Table A31. The parameter Identity of the MAFFT in the quantitative measurement of homology, assuming the *ND6 (NADH dehydrogenase subunit 6) protein of eight species dataset* [3].

	Homo sapiens	Gorilla gorilla	Pan troglodytes	Osphranter robustus	Phoca vitulina	Halichoerus grypus	Rattus norvegicus	Mus musculus
Homo sapiens	100	96.5517	95.977	45.509	58.6207	58.046	50	52.907
Gorilla gorilla		100	95.4023	45.509	58.046	57.4713	48.8372	51.7442
Pan troglodytes			100	44.9102	57.4713	56.8966	49.4186	51.7442
Osphranter robustus				100	46.1078	47.3054	40.7186	43.1138
Phoca vitulina					100	97.1429	54.0698	56.3953
Halichoerus grypus						100	55.2326	56.3953
Rattus norvegicus							100	80.2326
Mus musculus								100

Table A32. The parameter Similarity of the MAFFT in the quantitative measurement of homology, assuming the *ND6 (NADH dehydrogenase subunit 6) protein of eight species dataset* [3].

	Homo sapiens	Gorilla gorilla	Pan troglodytes	Osphranter robustus	Phoca vitulina	Halichoerus grypus	Rattus norvegicus	Mus musculus
Homo sapiens	100	98.2759	97.7011	69.4611	77.5862	78.1609	70.9302	72.6744
Gorilla gorilla		100	95.977	68.8623	76.4368	77.0115	69.186	71.5116
Pan troglodytes			100	68.2635	75.8621	76.4368	70.3488	71.5116
Osphranter robustus				100	67.0659	68.8623	62.2754	64.0719
Phoca vitulina					100	98.2857	72.093	74.4186
Halichoerus grypus						100	73.8372	76.1628
Rattus norvegicus							100	86.6279
Mus musculus								100

Table A33. The parameter Alignment Score of the MAFFT in the quantitative measurement of homology, assuming the *ND6 (NADH dehydrogenase subunit 6) protein of eight species dataset* [3].

	Homo sapiens	Gorilla gorilla	Pan troglodytes	Osphranter robustus	Phoca vitulina	Halichoerus grypus	Rattus norvegicus	Mus musculus
Homo sapiens	898	866	864	358.5	543	543	444	472
Gorilla gorilla		904	855	352.5	541	541	431	458
Pan troglodytes			902	351.5	540	540	443	467
Osphranter robustus				854	380.5	394.5	314	316
Phoca vitulina					894	874	490	508
Halichoerus grypus						896	498	514
Rattus norvegicus							906	746
Mus musculus								902

Table A34. The parameter Sensitivity (SEN) of the HELIOS method with referencing the Smith-Waterman in the accuracy measurement of classification output, assuming the *ND6 (NADH dehydrogenase subunit 6) protein of eight species dataset* [3].

	Homo sapiens	Gorilla gorilla	Pan troglodytes	Osphranter robustus	Phoca vitulina	Halichoerus grypus	Rattus norvegicus	Mus musculus
Homo sapiens	1	1	1	0.4878	0.94253	0.95402	0.96512	0.93605
Gorilla gorilla		1	1	0.4939	0.94253	0.94253	0.98256	0.88953
Pan troglodytes			1	0.5122	0.94798	0.94798	0.94767	0.90698
Osphranter robustus				1	0.59756	0.6	0.58537	0.57055
Phoca vitulina					1	1	0.88304	0.93023
Halichoerus grypus						1	0.85965	0.94767
Rattus norvegicus							1	0.90116
Mus musculus								1

Table A35. The parameter Specification (Spec) of the HELIOS method with referencing the Smith-Waterman in the accuracy measurement of classification output, assuming the *ND6 (NADH dehydrogenase subunit 6) protein of eight species dataset* [3].

	Homo sapiens	Gorilla gorilla	Pan troglodytes	Osphranter robustus	Phoca vitulina	Halichoerus grypus	Rattus norvegicus	Mus musculus
Homo sapiens	1	1	1	0.99742	0.9997	0.99977	0.99987	0.9998
Gorilla gorilla		1	1	0.99735	0.9997	0.9997	0.99993	0.9996
Pan troglodytes			1	0.99743	0.9997	0.9997	0.9998	0.99963
Osphranter robustus				1	0.99803	0.99794	0.9979	0.99755
Phoca vitulina					1	1	0.99947	0.9997
Halichoerus grypus						1	0.99933	0.99987
Rattus norvegicus							1	0.99949
Mus musculus								1

Table A36. The parameter Accuracy (Acc) of the HELIOS method with referencing the Smith-Waterman in the accuracy measurement of classification output, assuming the *ND6 (NADH dehydrogenase subunit 6) protein of eight species dataset* [3].

	Homo sapiens	Gorilla gorilla	Pan troglodytes	Osphranter robustus	Phoca vitulina	Halichoerus grypus	Rattus norvegicus	Mus musculus
Homo sapiens	1	1	1	0.99453	0.99938	0.99951	0.99967	0.99943
Gorilla gorilla		1	1	0.9945	0.99938	0.99938	0.99983	0.99896
Pan troglodytes			1	0.99464	0.99941	0.99941	0.9995	0.9991
Osphranter robustus				1	0.99577	0.99569	0.99554	0.99513
Phoca vitulina					1	1	0.9988	0.9993
Halichoerus grypus						1	0.99854	0.99957
Rattus norvegicus							1	0.99892
Mus musculus								1

Table A37. The parameter Positive Predictive Value (PPV) of the HELIOS method with referencing the Smith-Waterman in the accuracy measurement of classification output, assuming the *ND6 (NADH dehydrogenase subunit 6) protein of eight species dataset* [3].

	Homo sapiens	Gorilla gorilla	Pan troglodytes	Osphranter robustus	Phoca vitulina	Halichoerus grypus	Rattus norvegicus	Mus musculus
Homo sapiens	1	1	1	0.51948	0.94798	0.95954	0.97647	0.96407
Gorilla gorilla		1	1	0.51592	0.94798	0.94798	0.9883	0.92727
Pan troglodytes			1	0.53503	0.94798	0.94798	0.9645	0.93413
Osphranter robustus				1	0.63226	0.62264	0.61538	0.57055
Phoca vitulina					1	1	0.90419	0.94675
Halichoerus grypus						1	0.88024	0.97605
Rattus norvegicus							1	0.91176
Mus musculus								1

Table A38. The parameter Negative Predictive Value (NPV) of the HELIOS method with referencing the Smith-Waterman in the accuracy measurement of classification output, assuming the *ND6 (NADH dehydrogenase subunit 6) protein of eight species dataset* [3].

	Homo sapiens	Gorilla gorilla	Pan troglodytes	Osphranter robustus	Phoca vitulina	Halichoerus grypus	Rattus norvegicus	Mus musculus
Homo sapiens	1	1	1	0.99708	0.99967	0.99974	0.9998	0.99963
Gorilla gorilla		1	1	0.99711	0.99967	0.99967	0.9999	0.99936
Pan troglodytes			1	0.99718	0.9997	0.9997	0.9997	0.99946
Osphranter robustus				1	0.99772	0.99773	0.99762	0.99755
Phoca vitulina					1	1	0.99933	0.9996
Halichoerus grypus						1	0.9992	0.9997
Rattus norvegicus							1	0.99942
Mus musculus								1

Table A39. The parameter Matthew's Coefficient Correlation (MCC) of the HELIOS method with referencing the Smith-Waterman in the accuracy measurement of classification output, assuming the *ND6 (NADH dehydrogenase subunit 6) protein of eight species dataset* [3].

	Homo sapiens	Gorilla gorilla	Pan troglodytes	Osphranter robustus	Phoca vitulina	Halichoerus grypus	Rattus norvegicus	Mus musculus
Homo sapiens	1	1	1	0.50065	0.94494	0.95653	0.97061	0.94967
Gorilla gorilla		1	1	0.50203	0.94494	0.94494	0.98534	0.90769
Pan troglodytes			1	0.5208	0.94768	0.94768	0.9558	0.92
Osphranter robustus				1	0.61254	0.60905	0.59795	0.5681
Phoca vitulina					1	1	0.89295	0.9381
Halichoerus grypus						1	0.86915	0.96154
Rattus norvegicus							1	0.9059
Mus musculus								1

Table A40. The parameter Test's Accuracy (F-Score) of the HELIOS method with referencing the Smith-Waterman in the accuracy measurement of classification output, assuming the *ND6 (NADH dehydrogenase subunit 6) protein of eight species dataset* [3].

	Homo sapiens	Gorilla gorilla	Pan troglodytes	Osphranter robustus	Phoca vitulina	Halichoerus grypus	Rattus norvegicus	Mus musculus
Homo sapiens	1	1	1	0.50314	0.94524	0.95677	0.97076	0.94985
Gorilla gorilla		1	1	0.50467	0.94524	0.94524	0.98542	0.90801
Pan troglodytes			1	0.52336	0.94798	0.94798	0.95601	0.92035
Osphranter robustus				1	0.61442	0.61111	0.6	0.57055
Phoca vitulina					1	1	0.89349	0.93842
Halichoerus grypus						1	0.86982	0.96165
Rattus norvegicus							1	0.90643
Mus musculus								1

Table A41. The parameter Sensitivity (SEN) of the HELIOS method with referencing the Needleman-Wunsch in the accuracy measurement of classification output, assuming the *ND6 (NADH dehydrogenase subunit 6) protein of eight species dataset* [3].

	Homo sapiens	Gorilla gorilla	Pan troglodytes	Osphranter robustus	Phoca vitulina	Halichoerus grypus	Rattus norvegicus	Mus musculus
Homo sapiens	1	1	1	0	0.051724	0.04023	0.96491	0.93567
Gorilla gorilla		1	1	0.018519	0.051724	0.051724	0.98246	0.88889
Pan troglodytes			1	0.024691	0.046243	0.046243	0.94737	0.90643
Osphranter robustus				1	0.59756	0.60366	0.031447	0.056962
Phoca vitulina					1	1	0.058824	0.92982
Halichoerus grypus						1	0.029412	0.94737
Rattus norvegicus							1	0.90116
Mus musculus								1

Table A42. The parameter Specification (Spec) of the HELIOS method with referencing the Needleman-Wunsch in the accuracy measurement of classification output, assuming the *ND6 (NADH dehydrogenase subunit 6) protein of eight species dataset* [3].

	Homo sapiens	Gorilla gorilla	Pan troglodytes	Osphranter robustus	Phoca vitulina	Halichoerus grypus	Rattus norvegicus	Mus musculus
Homo sapiens	1	1	1	0.99455	0.99459	0.99452	0.99986	0.99979
Gorilla gorilla		1	1	0.99452	0.99459	0.99459	0.99993	0.99959
Pan troglodytes			1	0.99455	0.99455	0.99455	0.99979	0.99962
Osphranter robustus				1	0.998	0.99793	0.99458	0.99447
Phoca vitulina					1	1	0.9947	0.99969
Halichoerus grypus						1	0.99453	0.99986
Rattus norvegicus							1	0.99949
Mus musculus								1

Table A43. The parameter Accuracy (Acc) of the HELIOS method with referencing the Needleman-Wunsch in the accuracy measurement of classification output, assuming the *ND6 (NADH dehydrogenase subunit 6) protein of eight species dataset* [3].

	Homo sapiens	Gorilla gorilla	Pan troglodytes	Osphranter robustus	Phoca vitulina	Halichoerus grypus	Rattus norvegicus	Mus musculus
Homo sapiens	1	1	1	0.98876	0.98917	0.98903	0.99966	0.99942
Gorilla gorilla		1	1	0.98881	0.98917	0.98917	0.99983	0.99895
Pan troglodytes			1	0.98888	0.98913	0.98913	0.99949	0.99908
Osphranter robustus				1	0.99572	0.99568	0.98881	0.98889
Phoca vitulina					1	1	0.98929	0.99929
Halichoerus grypus						1	0.98895	0.99956
Rattus norvegicus							1	0.99892
Mus musculus								1

Table A44. The parameter Positive Predictive Value (PPV) of the HELIOS method with referencing the Needleman-Wunsch in the accuracy measurement of classification output, assuming the *ND6 (NADH dehydrogenase subunit 6) protein of eight species dataset* [3].

	Homo sapiens	Gorilla gorilla	Pan troglodytes	Osphranter robustus	Phoca vitulina	Halichoerus grypus	Rattus norvegicus	Mus musculus
Homo sapiens	1	1	1	0	0.052326	0.040698	0.97633	0.96386
Gorilla gorilla		1	1	0.019481	0.052326	0.052326	0.98824	0.92683
Pan troglodytes			1	0.025974	0.046512	0.046512	0.96429	0.93373
Osphranter robustus				1	0.63226	0.62658	0.033784	0.058065
Phoca vitulina					1	1	0.060606	0.94643
Halichoerus grypus						1	0.030303	0.9759
Rattus norvegicus							1	0.91176
Mus musculus								1

Table A45. The parameter Negative Predictive Value (NPV) of the HELIOS method with referencing the Needleman-Wunsch in the accuracy measurement of classification output, assuming the *ND6 (NADH dehydrogenase subunit 6) protein of eight species dataset* [3].

	Homo sapiens	Gorilla gorilla	Pan troglodytes	Osphranter robustus	Phoca vitulina	Halichoerus grypus	Rattus norvegicus	Mus musculus
Homo sapiens	1	1	1	0.99415	0.99452	0.99445	0.99979	0.99962
Gorilla gorilla		1	1	0.99423	0.99452	0.99452	0.9999	0.99935
Pan troglodytes			1	0.99426	0.99452	0.99452	0.99969	0.99945
Osphranter robustus				1	0.99769	0.99772	0.99417	0.99436
Phoca vitulina					1	1	0.99453	0.99959
Halichoerus grypus						1	0.99436	0.99969
Rattus norvegicus							1	0.99942
Mus musculus								1

Table A46. The parameter Matthew's Coefficient Correlation (MCC) of the HELIOS method with referencing the Needleman-Wunsch in the accuracy measurement of classification output, assuming the *ND6 (NADH dehydrogenase subunit 6) protein of eight species dataset* [3].

	Homo sapiens	Gorilla gorilla	Pan troglodytes	Osphranter robustus	Phoca vitulina	Halichoerus grypus	Rattus norvegicus	Mus musculus
Homo sapiens	1	1	1	-0.0056461	0.046576	0.034949	0.97043	0.94937
Gorilla gorilla		1	1	0.013368	0.046576	0.046576	0.98526	0.90713
Pan troglodytes			1	0.019735	0.040913	0.040913	0.95553	0.91952
Osphranter robustus				1	0.61251	0.61284	0.026973	0.051923
Phoca vitulina					1	1	0.054323	0.93773
Halichoerus grypus						1	0.024298	0.96131
Rattus norvegicus							1	0.9059
Mus musculus								1

Table A47. The parameter Test's Accuracy (F-Score) of the HELIOS method with referencing the Needleman-Wunsch in the accuracy measurement of classification output, assuming the *ND6 (NADH dehydrogenase subunit 6) protein of eight species dataset* [3].

	Homo sapiens	Gorilla gorilla	Pan troglodytes	Osphranter robustus	Phoca vitulina	Halichoerus grypus	Rattus norvegicus	Mus musculus
Homo sapiens	1	1	1	0	0.052023	0.040462	0.97059	0.94955
Gorilla gorilla		1	1	0.018987	0.052023	0.052023	0.98534	0.90746
Pan troglodytes			1	0.025316	0.046377	0.046377	0.95575	0.91988
Osphranter robustus				1	0.61442	0.61491	0.032573	0.057508
Phoca vitulina					1	1	0.059701	0.93805
Halichoerus grypus						1	0.029851	0.96142
Rattus norvegicus							1	0.90643
Mus musculus								1

Table A48. The parameter Sensitivity (SEN) of the HELIOS method with referencing the BLAST in the accuracy measurement of classification output, assuming the *ND6 (NADH dehydrogenase subunit 6) protein of eight species dataset* [3].

	Homo sapiens	Gorilla gorilla	Pan troglodytes	Osphranter robustus	Phoca vitulina	Halichoerus grypus	Rattus norvegicus	Mus musculus
Homo sapiens	1	1	1	0.6	0.94253	0.95402	0.87719	0.91228
Gorilla gorilla		1	1	0.60606	0.94253	0.94253	0.89474	0.87719
Pan troglodytes			1	0.62424	0.96552	0.96552	0.85965	0.8655
Osphranter robustus				1	0.5	0.54819	0.56442	0.55828
Phoca vitulina					1	1	0.94186	0.9186
Halichoerus grypus						1	0.88372	0.94186
Rattus norvegicus							1	0.90116
Mus musculus								1

Table A49. The parameter Specification (Spec) of the HELIOS method with referencing the BLAST in the accuracy measurement of classification output, assuming the *ND6 (NADH dehydrogenase subunit 6) protein of eight species dataset* [3].

	Homo sapiens	Gorilla gorilla	Pan troglodytes	Osphranter robustus	Phoca vitulina	Halichoerus grypus	Rattus norvegicus	Mus musculus
Homo sapiens	1	1	1	0.99806	0.9997	0.99977	0.99933	0.99963
Gorilla gorilla		1	1	0.99799	0.9997	0.9997	0.9994	0.9995
Pan troglodytes			1	0.9981	0.99983	0.99983	0.99926	0.99936
Osphranter robustus				1	0.99749	0.99766	0.99776	0.99748
Phoca vitulina					1	1	0.99983	0.99963
Halichoerus grypus						1	0.9995	0.99983
Rattus norvegicus							1	0.99949
Mus musculus								1

Table A50. The parameter Accuracy (Acc) of the HELIOS method with referencing the BLAST in the accuracy measurement of classification output, assuming the *ND6 (NADH dehydrogenase subunit 6) protein of eight species dataset* [3].

	Homo sapiens	Gorilla gorilla	Pan troglodytes	Osphranter robustus	Phoca vitulina	Halichoerus grypus	Rattus norvegicus	Mus musculus
Homo sapiens	1	1	1	0.9958	0.99938	0.99951	0.99863	0.99913
Gorilla gorilla		1	1	0.99577	0.99938	0.99938	0.9988	0.9988
Pan troglodytes			1	0.99597	0.99964	0.99964	0.99846	0.9986
Osphranter robustus				1	0.99466	0.99511	0.9953	0.99499
Phoca vitulina					1	1	0.9995	0.99917
Halichoerus grypus						1	0.99884	0.9995
Rattus norvegicus							1	0.99892
Mus musculus								1

Table A51. The parameter Positive Predictive Value (PPV) of the HELIOS method with referencing the BLAST in the accuracy measurement of classification output, assuming the *ND6 (NADH dehydrogenase subunit 6) protein of eight species dataset* [3].

	Homo sapiens	Gorilla gorilla	Pan troglodytes	Osphranter robustus	Phoca vitulina	Halichoerus grypus	Rattus norvegicus	Mus musculus
Homo sapiens	1	1	1	0.63871	0.94798	0.95954	0.88235	0.93413
Gorilla gorilla		1	1	0.63291	0.94798	0.94798	0.89474	0.90909
Pan troglodytes			1	0.6519	0.9711	0.9711	0.86982	0.88623
Osphranter robustus				1	0.53205	0.57233	0.58974	0.55828
Phoca vitulina					1	1	0.97006	0.93491
Halichoerus grypus						1	0.91018	0.97006
Rattus norvegicus							1	0.91176
Mus musculus								1

Table A52. The parameter Negative Predictive Value (NPV) of the HELIOS method with referencing the BLAST in the accuracy measurement of classification output, assuming the *ND6 (NADH dehydrogenase subunit 6) protein of eight species dataset* [3].

	Homo sapiens	Gorilla gorilla	Pan troglodytes	Osphranter robustus	Phoca vitulina	Halichoerus grypus	Rattus norvegicus	Mus musculus
Homo sapiens	1	1	1	0.99772	0.99967	0.99974	0.99929	0.9995
Gorilla gorilla		1	1	0.99775	0.99967	0.99967	0.9994	0.99929
Pan troglodytes			1	0.99785	0.9998	0.9998	0.99919	0.99923
Osphranter robustus				1	0.99714	0.99742	0.99751	0.99748
Phoca vitulina					1	1	0.99967	0.99953
Halichoerus grypus						1	0.99933	0.99967
Rattus norvegicus							1	0.99942
Mus musculus								1

Table A53. The parameter Matthew's Coefficient Correlation (MCC) of the HELIOS method with referencing the BLAST in the accuracy measurement of classification output, assuming the *ND6 (NADH dehydrogenase subunit 6) protein of eight species dataset* [3].

	Homo sapiens	Gorilla gorilla	Pan troglodytes	Osphranter robustus	Phoca vitulina	Halichoerus grypus	Rattus norvegicus	Mus musculus
Homo sapiens	1	1	1	0.61695	0.94494	0.95653	0.87908	0.92271
Gorilla gorilla		1	1	0.61721	0.94494	0.94494	0.89413	0.8924
Pan troglodytes			1	0.6359	0.96812	0.96812	0.86395	0.8751
Osphranter robustus				1	0.5131	0.55767	0.57458	0.55576
Phoca vitulina					1	1	0.95561	0.9263
Halichoerus grypus						1	0.89627	0.95561
Rattus norvegicus							1	0.9059
Mus musculus								1

Table A54. The parameter Test's Accuracy (F-Score) of the HELIOS method with referencing the BLAST in the accuracy measurement of classification output, assuming the *ND6 (NADH dehydrogenase subunit 6) protein of eight species dataset* [3].

	Homo sapiens	Gorilla gorilla	Pan troglodytes	Osphranter robustus	Phoca vitulina	Halichoerus grypus	Rattus norvegicus	Mus musculus
Homo sapiens	1	1	1	0.61875	0.94524	0.95677	0.87977	0.92308
Gorilla gorilla		1	1	0.6192	0.94524	0.94524	0.89474	0.89286
Pan troglodytes			1	0.63777	0.9683	0.9683	0.86471	0.87574
Osphranter robustus				1	0.51553	0.56	0.5768	0.55828
Phoca vitulina					1	1	0.95575	0.92669
Halichoerus grypus						1	0.89676	0.95575
Rattus norvegicus							1	0.90643
Mus musculus								1

Table A55. The parameter Sensitivity (SEN) of the HELIOS method with referencing the ClustalW in the accuracy measurement of classification output, assuming the *ND6 (NADH dehydrogenase subunit 6) protein of eight species dataset* [3].

	Homo sapiens	Gorilla gorilla	Pan troglodytes	Osphranter robustus	Phoca vitulina	Halichoerus grypus	Rattus norvegicus	Mus musculus
Homo sapiens	1	1	1	0.59394	0.94253	0.95402	0.88953	0.93023
Gorilla gorilla		1	1	0.6	0.94253	0.94253	0.90698	0.88372
Pan troglodytes			1	0.61818	0.96552	0.96552	0.87209	0.87791
Osphranter robustus				1	0.59036	0.63855	0.53374	0.55828
Phoca vitulina					1	1	0.91279	0.90116
Halichoerus grypus						1	0.85465	0.90116
Rattus norvegicus							1	0.90116
Mus musculus								1

Table A56. The parameter Specification (Spec) of the HELIOS method with referencing the ClustalW in the accuracy measurement of classification output, assuming the *ND6 (NADH dehydrogenase subunit 6) protein of eight species dataset* [3].

	Homo sapiens	Gorilla gorilla	Pan troglodytes	Osphranter robustus	Phoca vitulina	Halichoerus grypus	Rattus norvegicus	Mus musculus
Homo sapiens	1	1	1	0.99803	0.9997	0.99977	0.99943	0.99976
Gorilla gorilla		1	1	0.99796	0.9997	0.9997	0.9995	0.99956
Pan troglodytes			1	0.99806	0.99983	0.99983	0.99936	0.99946
Osphranter robustus				1	0.998	0.99818	0.99758	0.99748
Phoca vitulina					1	1	0.99967	0.99953
Halichoerus grypus						1	0.99933	0.9996
Rattus norvegicus							1	0.99949
Mus musculus								1

Table A57. The parameter Accuracy (Acc) of the HELIOS method with referencing the ClustalW in the accuracy measurement of classification output, assuming the *ND6 (NADH dehydrogenase subunit 6) protein of eight species dataset* [3].

	Homo sapiens	Gorilla gorilla	Pan troglodytes	Osphranter robustus	Phoca vitulina	Halichoerus grypus	Rattus norvegicus	Mus musculus
Homo sapiens	1	1	1	0.99573	0.99938	0.99951	0.9988	0.99937
Gorilla gorilla		1	1	0.9957	0.99938	0.99938	0.99896	0.9989
Pan troglodytes			1	0.9959	0.99964	0.99964	0.99863	0.99876
Osphranter robustus				1	0.99569	0.99613	0.99495	0.99499
Phoca vitulina					1	1	0.99917	0.99897
Halichoerus grypus						1	0.9985	0.99904
Rattus norvegicus							1	0.99892
Mus musculus								1

Table A58. The parameter Positive Predictive Value (PPV) of the HELIOS method with referencing the ClustalW in the accuracy measurement of classification output, assuming the *ND6 (NADH dehydrogenase subunit 6) protein of eight species dataset* [3].

	Homo sapiens	Gorilla gorilla	Pan troglodytes	Osphranter robustus	Phoca vitulina	Halichoerus grypus	Rattus norvegicus	Mus musculus
Homo sapiens	1	1	1	0.63226	0.94798	0.95954	0.9	0.95808
Gorilla gorilla		1	1	0.62658	0.94798	0.94798	0.91228	0.92121
Pan troglodytes			1	0.64557	0.9711	0.9711	0.88757	0.90419
Osphranter robustus				1	0.62821	0.66667	0.55769	0.55828
Phoca vitulina					1	1	0.94012	0.91716
Halichoerus grypus						1	0.88024	0.92814
Rattus norvegicus							1	0.91176
Mus musculus								1

Table A59. The parameter Negative Predictive Value (NPV) of the HELIOS method with referencing the ClustalW in the accuracy measurement of classification output, assuming the *ND6 (NADH dehydrogenase subunit 6) protein of eight species dataset* [3].

	Homo sapiens	Gorilla gorilla	Pan troglodytes	Osphranter robustus	Phoca vitulina	Halichoerus grypus	Rattus norvegicus	Mus musculus
Homo sapiens	1	1	1	0.99768	0.99967	0.99974	0.99936	0.9996
Gorilla gorilla		1	1	0.99772	0.99967	0.99967	0.99946	0.99933
Pan troglodytes			1	0.99782	0.9998	0.9998	0.99926	0.99929
Osphranter robustus				1	0.99766	0.99794	0.99734	0.99748
Phoca vitulina					1	1	0.9995	0.99943
Halichoerus grypus						1	0.99916	0.99943
Rattus norvegicus							1	0.99942
Mus musculus								1

Table A60. The parameter Matthew's Coefficient Correlation (MCC) of the HELIOS method with referencing the ClustalW in the accuracy measurement of classification output, assuming the *ND6 (NADH dehydrogenase subunit 6) protein of eight species dataset* [3].

	Homo sapiens	Gorilla gorilla	Pan troglodytes	Osphranter robustus	Phoca vitulina	Halichoerus grypus	Rattus norvegicus	Mus musculus
Homo sapiens	1	1	1	0.61066	0.94494	0.95653	0.89415	0.94374
Gorilla gorilla		1	1	0.61099	0.94494	0.94494	0.9091	0.90172
Pan troglodytes			1	0.62967	0.96812	0.96812	0.87911	0.89033
Osphranter robustus				1	0.60683	0.65052	0.54305	0.55576
Phoca vitulina					1	1	0.92594	0.90861
Halichoerus grypus						1	0.8666	0.91407
Rattus norvegicus							1	0.9059
Mus musculus								1

Table A61. The parameter Test's Accuracy (F-Score) of the HELIOS method with referencing the ClustalW in the accuracy measurement of classification output, assuming the *ND6 (NADH dehydrogenase subunit 6) protein of eight species dataset* [3].

	Homo sapiens	Gorilla gorilla	Pan troglodytes	Osphranter robustus	Phoca vitulina	Halichoerus grypus	Rattus norvegicus	Mus musculus
Homo sapiens	1	1	1	0.6125	0.94524	0.95677	0.89474	0.94395
Gorilla gorilla		1	1	0.613	0.94524	0.94524	0.90962	0.90208
Pan troglodytes			1	0.63158	0.9683	0.9683	0.87977	0.89086
Osphranter robustus				1	0.6087	0.65231	0.54545	0.55828
Phoca vitulina					1	1	0.92625	0.90909
Halichoerus grypus						1	0.86726	0.91445
Rattus norvegicus							1	0.90643
Mus musculus								1

Table A62. The parameter Sensitivity (SEN) of the HELIOS method with referencing the ClustalΩ in the accuracy measurement of classification output, assuming the *ND6 (NADH dehydrogenase subunit 6) protein of eight species dataset* [3].

	Homo sapiens	Gorilla gorilla	Pan troglodytes	Osphranter robustus	Phoca vitulina	Halichoerus grypus	Rattus norvegicus	Mus musculus
Homo sapiens	1	1	1	0.6	0.94253	0.95402	0.91279	0.93605
Gorilla gorilla		1	1	0.60606	0.94253	0.94253	0.93023	0.88953
Pan troglodytes			1	0.62424	0.96552	0.96552	0.89535	0.90116
Osphranter robustus				1	0.57229	0.62048	0.53374	0.53988
Phoca vitulina					1	1	0.91279	0.87791
Halichoerus grypus						1	0.85465	0.91279
Rattus norvegicus							1	0.90116
Mus musculus								1

Table A63. The parameter Specification (Spec) of the HELIOS method with referencing the ClustalΩ in the accuracy measurement of classification output, assuming the *ND6 (NADH dehydrogenase subunit 6) protein of eight species dataset* [3].

	Homo sapiens	Gorilla gorilla	Pan troglodytes	Osphranter robustus	Phoca vitulina	Halichoerus grypus	Rattus norvegicus	Mus musculus
Homo sapiens	1	1	1	0.99806	0.9997	0.99977	0.99956	0.9998
Gorilla gorilla		1	1	0.99799	0.9997	0.9997	0.99963	0.9996
Pan troglodytes			1	0.9981	0.99983	0.99983	0.9995	0.9996
Osphranter robustus				1	0.9979	0.99807	0.99758	0.99737
Phoca vitulina					1	1	0.99967	0.9994
Halichoerus grypus						1	0.99933	0.99967
Rattus norvegicus							1	0.99949
Mus musculus								1

Table A64. The parameter Accuracy (Acc) of the HELIOS method with referencing the ClustalΩ in the accuracy measurement of classification output, assuming the *ND6 (NADH dehydrogenase subunit 6) protein of eight species dataset* [3].

	Homo sapiens	Gorilla gorilla	Pan troglodytes	Osphranter robustus	Phoca vitulina	Halichoerus grypus	Rattus norvegicus	Mus musculus
Homo sapiens	1	1	1	0.9958	0.99938	0.99951	0.99906	0.99943
Gorilla gorilla		1	1	0.99577	0.99938	0.99938	0.99923	0.99896
Pan troglodytes			1	0.99597	0.99964	0.99964	0.9989	0.99903
Osphranter robustus				1	0.99548	0.99593	0.99495	0.99478
Phoca vitulina					1	1	0.99917	0.9987
Halichoerus grypus						1	0.9985	0.99917
Rattus norvegicus							1	0.99892
Mus musculus								1

Table A65. The parameter Positive Predictive Value (PPV) of the HELIOS method with referencing the ClustalΩ in the accuracy measurement of classification output, assuming the *ND6 (NADH dehydrogenase subunit 6) protein of eight species dataset* [3].

	Homo sapiens	Gorilla gorilla	Pan troglodytes	Osphranter robustus	Phoca vitulina	Halichoerus grypus	Rattus norvegicus	Mus musculus
Homo sapiens	1	1	1	0.63871	0.94798	0.95954	0.92353	0.96407
Gorilla gorilla		1	1	0.63291	0.94798	0.94798	0.93567	0.92727
Pan troglodytes			1	0.6519	0.9711	0.9711	0.91124	0.92814
Osphranter robustus				1	0.60897	0.6478	0.55769	0.53988
Phoca vitulina					1	1	0.94012	0.89349
Halichoerus grypus						1	0.88024	0.94012
Rattus norvegicus							1	0.91176
Mus musculus								1

Table A66. The parameter Negative Predictive Value (NPV) of the HELIOS method with referencing the ClustalΩ in the accuracy measurement of classification output, assuming the *ND6 (NADH dehydrogenase subunit 6) protein of eight species dataset* [3].

	Homo sapiens	Gorilla gorilla	Pan troglodytes	Osphranter robustus	Phoca vitulina	Halichoerus grypus	Rattus norvegicus	Mus musculus
Homo sapiens	1	1	1	0.99772	0.99967	0.99974	0.9995	0.99963
Gorilla gorilla		1	1	0.99775	0.99967	0.99967	0.9996	0.99936
Pan troglodytes			1	0.99785	0.9998	0.9998	0.9994	0.99943
Osphranter robustus				1	0.99756	0.99783	0.99734	0.99737
Phoca vitulina					1	1	0.9995	0.9993
Halichoerus grypus						1	0.99916	0.9995
Rattus norvegicus							1	0.99942
Mus musculus								1

Table A67. The parameter Matthew's Coefficient Correlation (MCC) of the HELIOS method with referencing the ClustalΩ in the accuracy measurement of classification output, assuming the *ND6 (NADH dehydrogenase subunit 6) protein of eight species dataset* [3].

	Homo sapiens	Gorilla gorilla	Pan troglodytes	Osphranter robustus	Phoca vitulina	Halichoerus grypus	Rattus norvegicus	Mus musculus
Homo sapiens	1	1	1	0.61695	0.94494	0.95653	0.91767	0.94967
Gorilla gorilla		1	1	0.61721	0.94494	0.94494	0.93256	0.90769
Pan troglodytes			1	0.6359	0.96812	0.96812	0.90271	0.91407
Osphranter robustus				1	0.58808	0.63195	0.54305	0.53725
Phoca vitulina					1	1	0.92594	0.88501
Halichoerus grypus						1	0.8666	0.92594
Rattus norvegicus							1	0.9059
Mus musculus								1

Table A68. The parameter Test's Accuracy (F-Score) of the HELIOS method with referencing the ClustalΩ in the accuracy measurement of classification output, assuming the *ND6 (NADH dehydrogenase subunit 6) protein of eight species dataset* [3].

	Homo sapiens	Gorilla gorilla	Pan troglodytes	Osphranter robustus	Phoca vitulina	Halichoerus grypus	Rattus norvegicus	Mus musculus
Homo sapiens	1	1	1	0.61875	0.94524	0.95677	0.91813	0.94985
Gorilla gorilla		1	1	0.6192	0.94524	0.94524	0.93294	0.90801
Pan troglodytes			1	0.63777	0.9683	0.9683	0.90323	0.91445
Osphranter robustus				1	0.59006	0.63385	0.54545	0.53988
Phoca vitulina					1	1	0.92625	0.88563
Halichoerus grypus						1	0.86726	0.92625
Rattus norvegicus							1	0.90643
Mus musculus								1

Table A69. The parameter Sensitivity (SEN) of the HELIOS method with referencing the MUSCLE in the accuracy measurement of classification output, assuming the *ND6 (NADH dehydrogenase subunit 6) protein of eight species dataset* [3].

	Homo sapiens	Gorilla gorilla	Pan troglodytes	Osphranter robustus	Phoca vitulina	Halichoerus grypus	Rattus norvegicus	Mus musculus
Homo sapiens	1	1	1	0.59394	0.94253	0.95402	0.88953	0.93023
Gorilla gorilla		1	1	0.6	0.94253	0.94253	0.90698	0.88372
Pan troglodytes			1	0.61818	0.96552	0.96552	0.87209	0.87791
Osphranter robustus				1	0.60241	0.6506	0.53988	0.55215
Phoca vitulina					1	1	0.91279	0.90116
Halichoerus grypus						1	0.85465	0.90116
Rattus norvegicus							1	0.90116
Mus musculus								1

Table A70. The parameter Specification (Spec) of the HELIOS method with referencing the MUSCLE in the accuracy measurement of classification output, assuming the *ND6 (NADH dehydrogenase subunit 6) protein of eight species dataset* [3].

	Homo sapiens	Gorilla gorilla	Pan troglodytes	Osphranter robustus	Phoca vitulina	Halichoerus grypus	Rattus norvegicus	Mus musculus
Homo sapiens	1	1	1	0.99803	0.9997	0.99977	0.99943	0.99976
Gorilla gorilla		1	1	0.99796	0.9997	0.9997	0.9995	0.99956
Pan troglodytes			1	0.99806	0.99983	0.99983	0.99936	0.99946
Osphranter robustus				1	0.99807	0.99824	0.99762	0.99744
Phoca vitulina					1	1	0.99967	0.99953
Halichoerus grypus						1	0.99933	0.9996
Rattus norvegicus							1	0.99949
Mus musculus								1

Table A71. The parameter Accuracy (Acc) of the HELIOS method with referencing the MUSCLE in the accuracy measurement of classification output, assuming the *ND6 (NADH dehydrogenase subunit 6) protein of eight species dataset* [3].

	Homo sapiens	Gorilla gorilla	Pan troglodytes	Osphranter robustus	Phoca vitulina	Halichoerus grypus	Rattus norvegicus	Mus musculus
Homo sapiens	1	1	1	0.99573	0.99938	0.99951	0.9988	0.99937
Gorilla gorilla		1	1	0.9957	0.99938	0.99938	0.99896	0.9989
Pan troglodytes			1	0.9959	0.99964	0.99964	0.99863	0.99876
Osphranter robustus				1	0.99583	0.99627	0.99502	0.99492
Phoca vitulina					1	1	0.99917	0.99897
Halichoerus grypus						1	0.9985	0.99904
Rattus norvegicus							1	0.99892
Mus musculus								1

Table A72. The parameter Positive Predictive Value (PPV) of the HELIOS method with referencing the MUSCLE in the accuracy measurement of classification output, assuming the *ND6 (NADH dehydrogenase subunit 6) protein of eight species dataset* [3].

	Homo sapiens	Gorilla gorilla	Pan troglodytes	Osphranter robustus	Phoca vitulina	Halichoerus grypus	Rattus norvegicus	Mus musculus
Homo sapiens	1	1	1	0.63226	0.94798	0.95954	0.9	0.95808
Gorilla gorilla		1	1	0.62658	0.94798	0.94798	0.91228	0.92121
Pan troglodytes			1	0.64557	0.9711	0.9711	0.88757	0.90419
Osphranter robustus				1	0.64103	0.67925	0.5641	0.55215
Phoca vitulina					1	1	0.94012	0.91716
Halichoerus grypus						1	0.88024	0.92814
Rattus norvegicus							1	0.91176
Mus musculus								1

Table A73. The parameter Negative Predictive Value (NPV) of the HELIOS method with referencing the MUSCLE in the accuracy measurement of classification output, assuming the *ND6 (NADH dehydrogenase subunit 6) protein of eight species dataset* [3].

	Homo sapiens	Gorilla gorilla	Pan troglodytes	Osphranter robustus	Phoca vitulina	Halichoerus grypus	Rattus norvegicus	Mus musculus
Homo sapiens	1	1	1	0.99768	0.99967	0.99974	0.99936	0.9996
Gorilla gorilla		1	1	0.99772	0.99967	0.99967	0.99946	0.99933
Pan troglodytes			1	0.99782	0.9998	0.9998	0.99926	0.99929
Osphranter robustus				1	0.99773	0.998	0.99737	0.99744
Phoca vitulina					1	1	0.9995	0.99943
Halichoerus grypus						1	0.99916	0.99943
Rattus norvegicus							1	0.99942
Mus musculus								1

Table A74. The parameter Matthew's Coefficient Correlation (MCC) of the HELIOS method with referencing the MUSCLE in the accuracy measurement of classification output, assuming the *ND6 (NADH dehydrogenase subunit 6) protein of eight species dataset* [3].

	Homo sapiens	Gorilla gorilla	Pan troglodytes	Osphranter robustus	Phoca vitulina	Halichoerus grypus	Rattus norvegicus	Mus musculus
Homo sapiens	1	1	1	0.61066	0.94494	0.95653	0.89415	0.94374
Gorilla gorilla		1	1	0.61099	0.94494	0.94494	0.9091	0.90172
Pan troglodytes			1	0.62967	0.96812	0.96812	0.87911	0.89033
Osphranter robustus				1	0.61932	0.6629	0.54936	0.54959
Phoca vitulina					1	1	0.92594	0.90861
Halichoerus grypus						1	0.8666	0.91407
Rattus norvegicus							1	0.9059
Mus musculus								1

Table A75. The parameter Test's Accuracy (F-Score) of the HELIOS method with referencing the MUSCLE in the accuracy measurement of classification output, assuming the *ND6 (NADH dehydrogenase subunit 6) protein of eight species dataset* [3].

	Homo sapiens	Gorilla gorilla	Pan troglodytes	Osphranter robustus	Phoca vitulina	Halichoerus grypus	Rattus norvegicus	Mus musculus
Homo sapiens	1	1	1	0.6125	0.94524	0.95677	0.89474	0.94395
Gorilla gorilla		1	1	0.613	0.94524	0.94524	0.90962	0.90208
Pan troglodytes			1	0.63158	0.9683	0.9683	0.87977	0.89086
Osphranter robustus				1	0.62112	0.66462	0.55172	0.55215
Phoca vitulina					1	1	0.92625	0.90909
Halichoerus grypus						1	0.86726	0.91445
Rattus norvegicus							1	0.90643
Mus musculus								1

Table A76. The parameter Sensitivity (SEN) of the HELIOS method with referencing the T-Coffee in the accuracy measurement of classification output, assuming the *ND6 (NADH dehydrogenase subunit 6) protein of eight species dataset* [3].

	Homo sapiens	Gorilla gorilla	Pan troglodytes	Osphranter robustus	Phoca vitulina	Halichoerus grypus	Rattus norvegicus	Mus musculus
Homo sapiens	1	0.005814	0.005814	0.59375	0.37209	0.36047	0.55814	0.59884
Gorilla gorilla		0.99429	0.99429	0.018405	0.0057471	0.0057471	0.063953	0.33908
Pan troglodytes			0.99429	0.02454	0.0057471	0.0057471	0.075581	0.38506
Osphranter robustus				1	0	0	0.59375	0.57407
Phoca vitulina					1	1	0.02907	0.04023
Halichoerus grypus						1	0.02907	0.028736
Rattus norvegicus							1	0.57558
Mus musculus								0.98851

Table A77. The parameter Specification (Spec) of the HELIOS method with referencing the T-Coffee in the accuracy measurement of classification output, assuming the *ND6 (NADH dehydrogenase subunit 6) protein of eight species dataset* [3].

	Homo sapiens	Gorilla gorilla	Pan troglodytes	Osphranter robustus	Phoca vitulina	Halichoerus grypus	Rattus norvegicus	Mus musculus
Homo sapiens	1	0.99429	0.99429	0.99797	0.9964	0.99634	0.99752	0.99788
Gorilla gorilla		1	1	0.99467	0.99435	0.99435	0.99465	0.9965
Pan troglodytes			1	0.9947	0.99435	0.99435	0.99479	0.9967
Osphranter robustus				1	0.99464	0.99453	0.99786	0.99758
Phoca vitulina					1	1	0.99456	0.99462
Halichoerus grypus						1	0.99456	0.99462
Rattus norvegicus							1	0.99761
Mus musculus								1

Table A78. The parameter Accuracy (Acc) of the HELIOS method with referencing the T-Coffee in the accuracy measurement of classification output, assuming the *ND6 (NADH dehydrogenase subunit 6) protein of eight species dataset* [3].

	Homo sapiens	Gorilla gorilla	Pan troglodytes	Osphranter robustus	Phoca vitulina	Halichoerus grypus	Rattus norvegicus	Mus musculus
Homo sapiens	1	0.98864	0.98864	0.99572	0.99282	0.99268	0.99496	0.99559
Gorilla gorilla		0.99997	0.99997	0.98922	0.9887	0.9887	0.98934	0.99274
Pan troglodytes			0.99997	0.98929	0.9887	0.9887	0.98953	0.9932
Osphranter robustus				1	0.98909	0.98899	0.99561	0.99522
Phoca vitulina					1	1	0.98901	0.98913
Halichoerus grypus						1	0.98901	0.98907
Rattus norvegicus							1	0.99519
Mus musculus								0.99993

Table A79. The parameter Positive Predictive Value (PPV) of the HELIOS method with referencing the T-Coffee in the accuracy measurement of classification output, assuming the *ND6 (NADH dehydrogenase subunit 6) protein of eight species dataset* [3].

	Homo sapiens	Gorilla gorilla	Pan troglodytes	Osphranter robustus	Phoca vitulina	Halichoerus grypus	Rattus norvegicus	Mus musculus
Homo sapiens	1	0.005814	0.005814	0.62092	0.37427	0.36257	0.56805	0.62048
Gorilla gorilla		1	1	0.018987	0.005814	0.005814	0.064327	0.35758
Pan troglodytes			1	0.025316	0.005814	0.005814	0.076923	0.4012
Osphranter robustus				1	0	0	0.60897	0.57055
Phoca vitulina					1	1	0.02994	0.04142
Halichoerus grypus						1	0.02994	0.02994
Rattus norvegicus							1	0.58235
Mus musculus								1

Table A80. The parameter Negative Predictive Value (NPV) of the HELIOS method with referencing the T-Coffee in the accuracy measurement of classification output, assuming the *ND6 (NADH dehydrogenase subunit 6) protein of eight species dataset* [3].

	Homo sapiens	Gorilla gorilla	Pan troglodytes	Osphranter robustus	Phoca vitulina	Halichoerus grypus	Rattus norvegicus	Mus musculus
Homo sapiens	1	0.99429	0.99429	0.99772	0.99637	0.9963	0.99742	0.99768
Gorilla gorilla		0.99997	0.99997	0.9945	0.99429	0.99429	0.99462	0.9962
Pan troglodytes			0.99997	0.99453	0.99429	0.99429	0.99469	0.99647
Osphranter robustus				1	0.9944	0.99439	0.99772	0.99761
Phoca vitulina					1	1	0.99439	0.99445
Halichoerus grypus						1	0.99439	0.99439
Rattus norvegicus							1	0.99755
Mus musculus								0.99993

Table A81. The parameter Matthew's Coefficient Correlation (MCC) of the HELIOS method with referencing the T-Coffee in the accuracy measurement of classification output, assuming the *ND6 (NADH dehydrogenase subunit 6) protein of eight species dataset* [3].

	Homo sapiens	Gorilla gorilla	Pan troglodytes	Osphranter robustus	Phoca vitulina	Halichoerus grypus	Rattus norvegicus	Mus musculus
Homo sapiens	1	0.00010024	0.00010024	0.60503	0.36957	0.35784	0.56054	0.60735
Gorilla gorilla		0.99712	0.99712	0.013276	9.966e-05	9.966e-05	0.058777	0.34456
Pan troglodytes			0.99712	0.019541	9.966e-05	9.966e-05	0.070987	0.38963
Osphranter robustus				1	-0.0054832	-0.0055363	0.59911	0.56991
Phoca vitulina					1	1	0.023975	0.035357
Halichoerus grypus						1	0.023975	0.023836
Rattus norvegicus							1	0.57654
Mus musculus								0.9942

Table A82. The parameter Test's Accuracy (F-Score) of the HELIOS method with referencing the T-Coffee in the accuracy measurement of classification output, assuming the *ND6 (NADH dehydrogenase subunit 6) protein of eight species dataset* [3].

	Homo sapiens	Gorilla gorilla	Pan troglodytes	Osphranter robustus	Phoca vitulina	Halichoerus grypus	Rattus norvegicus	Mus musculus
Homo sapiens	1	0.005814	0.005814	0.60703	0.37318	0.36152	0.56305	0.60947
Gorilla gorilla		0.99713	0.99713	0.018692	0.0057803	0.0057803	0.06414	0.34808
Pan troglodytes			0.99713	0.024922	0.0057803	0.0057803	0.076246	0.39296
Osphranter robustus				1	0	0	0.60127	0.57231
Phoca vitulina					1	1	0.029499	0.040816
Halichoerus grypus						1	0.029499	0.029326
Rattus norvegicus							1	0.57895
Mus musculus								0.99422

Table A83. The parameter Sensitivity (SEN) of the HELIOS method with referencing the Kalign in the accuracy measurement of classification output, assuming the *ND6 (NADH dehydrogenase subunit 6) protein of eight species dataset* [3].

	Homo sapiens	Gorilla gorilla	Pan troglodytes	Osphranter robustus	Phoca vitulina	Halichoerus grypus	Rattus norvegicus	Mus musculus
Homo sapiens	1	1	1	0.59394	0.94253	0.95402	0.88953	0.93023
Gorilla gorilla		1	1	0.6	0.94253	0.94253	0.90698	0.88372
Pan troglodytes			1	0.61818	0.96552	0.96552	0.87209	0.87791
Osphranter robustus				1	0.60241	0.6506	0.53988	0.55215
Phoca vitulina					1	1	0.91279	0.90116
Halichoerus grypus						1	0.85465	0.90116
Rattus norvegicus							1	0.90116
Mus musculus								1

Table A84. The parameter Specification (Spec) of the HELIOS method with referencing the Kalign in the accuracy measurement of classification output, assuming the *ND6 (NADH dehydrogenase subunit 6) protein of eight species dataset* [3].

	Homo sapiens	Gorilla gorilla	Pan troglodytes	Osphranter robustus	Phoca vitulina	Halichoerus grypus	Rattus norvegicus	Mus musculus
Homo sapiens	1	1	1	0.99803	0.9997	0.99977	0.99943	0.99976
Gorilla gorilla		1	1	0.99796	0.9997	0.9997	0.9995	0.99956
Pan troglodytes			1	0.99806	0.99983	0.99983	0.99936	0.99946
Osphranter robustus				1	0.99807	0.99824	0.99762	0.99744
Phoca vitulina					1	1	0.99967	0.99953
Halichoerus grypus						1	0.99933	0.9996
Rattus norvegicus							1	0.99949
Mus musculus								1

Table A85. The parameter Accuracy (Acc) of the HELIOS method with referencing the Kalign in the accuracy measurement of classification output, assuming the *ND6 (NADH dehydrogenase subunit 6) protein of eight species dataset* [3].

	Homo sapiens	Gorilla gorilla	Pan troglodytes	Osphranter robustus	Phoca vitulina	Halichoerus grypus	Rattus norvegicus	Mus musculus
Homo sapiens	1	1	1	0.99573	0.99938	0.99951	0.9988	0.99937
Gorilla gorilla		1	1	0.9957	0.99938	0.99938	0.99896	0.9989
Pan troglodytes			1	0.9959	0.99964	0.99964	0.99863	0.99876
Osphranter robustus				1	0.99583	0.99627	0.99502	0.99492
Phoca vitulina					1	1	0.99917	0.99897
Halichoerus grypus						1	0.9985	0.99904
Rattus norvegicus							1	0.99892
Mus musculus								1

Table A86. The parameter Positive Predictive Value (PPV) of the HELIOS method with referencing the Kalign in the accuracy measurement of classification output, assuming the *ND6 (NADH dehydrogenase subunit 6) protein of eight species dataset* [3].

	Homo sapiens	Gorilla gorilla	Pan troglodytes	Osphranter robustus	Phoca vitulina	Halichoerus grypus	Rattus norvegicus	Mus musculus
Homo sapiens	1	1	1	0.63226	0.94798	0.95954	0.9	0.95808
Gorilla gorilla		1	1	0.62658	0.94798	0.94798	0.91228	0.92121
Pan troglodytes			1	0.64557	0.9711	0.9711	0.88757	0.90419
Osphranter robustus				1	0.64103	0.67925	0.5641	0.55215
Phoca vitulina					1	1	0.94012	0.91716
Halichoerus grypus						1	0.88024	0.92814
Rattus norvegicus							1	0.91176
Mus musculus								1

Table A87. The parameter Negative Predictive Value (NPV) of the HELIOS method with referencing the Kalign in the accuracy measurement of classification output, assuming the *ND6 (NADH dehydrogenase subunit 6) protein of eight species dataset* [3].

	Homo sapiens	Gorilla gorilla	Pan troglodytes	Osphranter robustus	Phoca vitulina	Halichoerus grypus	Rattus norvegicus	Mus musculus
Homo sapiens	1	1	1	0.99768	0.99967	0.99974	0.99936	0.9996
Gorilla gorilla		1	1	0.99772	0.99967	0.99967	0.99946	0.99933
Pan troglodytes			1	0.99782	0.9998	0.9998	0.99926	0.99929
Osphranter robustus				1	0.99773	0.998	0.99737	0.99744
Phoca vitulina					1	1	0.9995	0.99943
Halichoerus grypus						1	0.99916	0.99943
Rattus norvegicus							1	0.99942
Mus musculus								1

Table A88. The parameter Matthew's Coefficient Correlation (MCC) of the HELIOS method with referencing the Kalign in the accuracy measurement of classification output, assuming the *ND6 (NADH dehydrogenase subunit 6) protein of eight species dataset* [3].

	Homo sapiens	Gorilla gorilla	Pan troglodytes	Osphranter robustus	Phoca vitulina	Halichoerus grypus	Rattus norvegicus	Mus musculus
Homo sapiens	1	1	1	0.61066	0.94494	0.95653	0.89415	0.94374
Gorilla gorilla		1	1	0.61099	0.94494	0.94494	0.9091	0.90172
Pan troglodytes			1	0.62967	0.96812	0.96812	0.87911	0.89033
Osphranter robustus				1	0.61932	0.6629	0.54936	0.54959
Phoca vitulina					1	1	0.92594	0.90861
Halichoerus grypus						1	0.8666	0.91407
Rattus norvegicus							1	0.9059
Mus musculus								1

Table A89. The parameter Test's Accuracy (F-Score) of the HELIOS method with referencing the Kalign in the accuracy measurement of classification output, assuming the *ND6 (NADH dehydrogenase subunit 6) protein of eight species dataset* [3].

	Homo sapiens	Gorilla gorilla	Pan troglodytes	Osphranter robustus	Phoca vitulina	Halichoerus grypus	Rattus norvegicus	Mus musculus
Homo sapiens	1	1	1	0.6125	0.94524	0.95677	0.89474	0.94395
Gorilla gorilla		1	1	0.613	0.94524	0.94524	0.90962	0.90208
Pan troglodytes			1	0.63158	0.9683	0.9683	0.87977	0.89086
Osphranter robustus				1	0.62112	0.66462	0.55172	0.55215
Phoca vitulina					1	1	0.92625	0.90909
Halichoerus grypus						1	0.86726	0.91445
Rattus norvegicus							1	0.90643
Mus musculus								1

Table A90. The parameter Sensitivity (SEN) of the HELIOS method with referencing the MAFFT in the accuracy measurement of classification output, assuming the *ND6 (NADH dehydrogenase subunit 6) protein of eight species dataset* [3].

	Homo sapiens	Gorilla gorilla	Pan troglodytes	Osphranter robustus	Phoca vitulina	Halichoerus grypus	Rattus norvegicus	Mus musculus
Homo sapiens	1	1	1	0.59394	0.94253	0.95402	0.88372	0.92442
Gorilla gorilla		1	1	0.6	0.94253	0.94253	0.90116	0.87791
Pan troglodytes			1	0.61818	0.96552	0.96552	0.86628	0.87209
Osphranter robustus				1	0.60241	0.6506	0.53988	0.55215
Phoca vitulina					1	1	0.90698	0.89535
Halichoerus grypus						1	0.84884	0.89535
Rattus norvegicus							1	0.90116
Mus musculus								1

Table A91. The parameter Specification (Spec) of the HELIOS method with referencing the MAFFT in the accuracy measurement of classification output, assuming the *ND6 (NADH dehydrogenase subunit 6) protein of eight species dataset* [3].

	Homo sapiens	Gorilla gorilla	Pan troglodytes	Osphranter robustus	Phoca vitulina	Halichoerus grypus	Rattus norvegicus	Mus musculus
Homo sapiens	1	1	1	0.99803	0.9997	0.99977	0.9994	0.99973
Gorilla gorilla		1	1	0.99796	0.9997	0.9997	0.99946	0.99953
Pan troglodytes			1	0.99806	0.99983	0.99983	0.99933	0.99943
Osphranter robustus				1	0.99807	0.99824	0.99762	0.99744
Phoca vitulina					1	1	0.99963	0.9995
Halichoerus grypus						1	0.9993	0.99957
Rattus norvegicus							1	0.99949
Mus musculus								1

Table A92. The parameter Accuracy (Acc) of the HELIOS method with referencing the MAFFT in the accuracy measurement of classification output, assuming the *ND6 (NADH dehydrogenase subunit 6) protein of eight species dataset* [3].

	Homo sapiens	Gorilla gorilla	Pan troglodytes	Osphranter robustus	Phoca vitulina	Halichoerus grypus	Rattus norvegicus	Mus musculus
Homo sapiens	1	1	1	0.61066	0.94494	0.95653	0.88827	0.9378
Gorilla gorilla		1	1	0.61099	0.94494	0.94494	0.90324	0.89575
Pan troglodytes			1	0.62967	0.96812	0.96812	0.87321	0.8844
Osphranter robustus				1	0.61932	0.6629	0.54936	0.54959
Phoca vitulina					1	1	0.92	0.90271
Halichoerus grypus						1	0.86067	0.90814
Rattus norvegicus							1	0.9059
Mus musculus								1

Table A93. The parameter Positive Predictive Value (PPV) of the HELIOS method with referencing the MAFFT in the accuracy measurement of classification output, assuming the *ND6 (NADH dehydrogenase subunit 6) protein of eight species dataset* [3].

	Homo sapiens	Gorilla gorilla	Pan troglodytes	Osphranter robustus	Phoca vitulina	Halichoerus grypus	Rattus norvegicus	Mus musculus
Homo sapiens	1	1	1	0.63226	0.94798	0.95954	0.89412	0.9521
Gorilla gorilla		1	1	0.62658	0.94798	0.94798	0.90643	0.91515
Pan troglodytes			1	0.64557	0.9711	0.9711	0.88166	0.8982
Osphranter robustus				1	0.64103	0.67925	0.5641	0.55215
Phoca vitulina					1	1	0.93413	0.91124
Halichoerus grypus						1	0.87425	0.92216
Rattus norvegicus							1	0.91176
Mus musculus								1

Table A94. The parameter Negative Predictive Value (NPV) of the HELIOS method with referencing the MAFFT in the accuracy measurement of classification output, assuming the *ND6 (NADH dehydrogenase subunit 6) protein of eight species dataset* [3].

	Homo sapiens	Gorilla gorilla	Pan troglodytes	Osphranter robustus	Phoca vitulina	Halichoerus grypus	Rattus norvegicus	Mus musculus
Homo sapiens	1	1	1	0.99768	0.99967	0.99974	0.99933	0.99956
Gorilla gorilla		1	1	0.99772	0.99967	0.99967	0.99943	0.99929
Pan troglodytes			1	0.99782	0.9998	0.9998	0.99923	0.99926
Osphranter robustus				1	0.99773	0.998	0.99737	0.99744
Phoca vitulina					1	1	0.99947	0.9994
Halichoerus grypus						1	0.99913	0.9994
Rattus norvegicus							1	0.99942
Mus musculus								1

Table A95. The parameter Matthew's Coefficient Correlation (MCC) of the HELIOS method with referencing the MAFFT in the accuracy measurement of classification output, assuming the *ND6 (NADH dehydrogenase subunit 6) protein of eight species dataset* [3].

	Homo sapiens	Gorilla gorilla	Pan troglodytes	Osphranter robustus	Phoca vitulina	Halichoerus grypus	Rattus norvegicus	Mus musculus
Homo sapiens	1	1	1	0.61066	0.94494	0.95653	0.88827	0.9378
Gorilla gorilla		1	1	0.61099	0.94494	0.94494	0.90324	0.89575
Pan troglodytes			1	0.62967	0.96812	0.96812	0.87321	0.8844
Osphranter robustus				1	0.61932	0.6629	0.54936	0.54959
Phoca vitulina					1	1	0.92	0.90271
Halichoerus grypus						1	0.86067	0.90814
Rattus norvegicus							1	0.9059
Mus musculus								1

Table A96. The parameter Test's Accuracy (F-Score) of the HELIOS method with referencing the MAFFT in the accuracy measurement of classification output, assuming the *ND6 (NADH dehydrogenase subunit 6) protein of eight species dataset* [3].

	Homo sapiens	Gorilla gorilla	Pan troglodytes	Osphranter robustus	Phoca vitulina	Halichoerus grypus	Rattus norvegicus	Mus musculus
Homo sapiens	1	1	1	0.6125	0.94524	0.95677	0.88889	0.93805
Gorilla gorilla		1	1	0.613	0.94524	0.94524	0.90379	0.89614
Pan troglodytes			1	0.63158	0.9683	0.9683	0.8739	0.88496
Osphranter robustus				1	0.62112	0.66462	0.55172	0.55215
Phoca vitulina					1	1	0.92035	0.90323
Halichoerus grypus						1	0.86136	0.90855
Rattus norvegicus							1	0.90643
Mus musculus								1
