# Supplementary material for: HELIOS: High-speed sequence alignment in optics
Source: PLoS Comput Biol. 2022 Nov 21;18(11):e1010665. doi: 10.1371/journal.pcbi.1010665 (PMC9678324; doi:10.1371/journal.pcbi.1010665)
Supplement: S4 Text — (PDF) [file pcbi.1010665.s004.pdf]

HELIOS: High-Speed Sequence Alignment in Optics: S4 Text

Ehsan Maleki[1], **Saeedeh Akbari Rokn Abadi**[1], **and Somayyeh Koohi**[1,*]

[1] *Department of Computer Engineering, Sharif University of Technology, Azadi Ave., Tehran, Iran.*
[*] *Corresponding author: koohi@sharif.edu*

Compiled October 20, 2022

In response to the imperfections of current sequence alignment methods, originated from the inherent serialism within their corresponding electrical systems, a few optical approaches for biological data comparison have been proposed recently. However, due to their low performance, raised from their inefficient coding scheme, this paper presents a novel all-optical high-throughput method for aligning DNA, RNA, and protein sequences, named HELIOS. The HELIOS method employs highly sophisticated operations to locate character matches, single or multiple mutations, and single or multiple indels within various biological sequences. On the other hand, the HELIOS optical architecture exploits high-speed processing and operational parallelism in optics, by adopting wavelength and polarization of optical beams. For evaluation, the functionality and accuracy of the HELIOS method are approved through behavioral and optical simulation studies, while its complexity and performance are estimated through analytical computation. The accuracy evaluations indicate that the HELIOS method achieves a precise pairwise alignment of two sequences, highly similar to those of Smith-Waterman, Needleman-Wunsch, BLAST, MUSCLE, ClustalW, ClustalΩ, T-Coffee, Kalign, and MAFFT. According to our performance evaluations, the HELIOS optical architecture outperforms all alternative electrical and optical algorithms in terms of processing time and memory requirement, relying on its highly sophisticated method and optical architecture. Moreover, the employed compact coding scheme highly escalates the number of input characters, and hence, it offers reduced time and space complexities, compared to the electrical and optical alternatives. It makes the HELIOS method and optical architecture highly applicable for biomedical applications.

1. ACCURACY EVALUATION

In order to comprehensively assess the accuracy of the HELIOS method, two statistical analyses are performed through simulation studies: 1) Quantitative measurement of homology [1], and 2) Accuracy measurement of classification output [2], compared to the well-known algorithms, briefly reported in Tables A1 and A2, respectively. In this manner, the *ENCODE Transcription Factor Targets Dataset, TFAP2A Gene Set* [3] is assumed in this study, as represented in Table A3.

A. Quantitative measurement of homology

To perform quantitative measurement of homology [1], the parameters Identity, Similarity, and Alignment Score of the HELIOS outputs are calculated through simulation studies, as reported in Tables A4-A6, respectively, assuming the *ENCODE Transcription Factor Targets Dataset, TFAP2A Gene Set* [3]. While the Identity reports the number of exactly matched characters of two sequences (in percentage), the Similarity measures the resemblance of two compared sequences. Specifically, regarding the physicochemical properties, the amino acids are categorized into six groups with different Similarity values; including GAVLI, FYW, STCM, KRH, DENQ, and P. As the third metric, the BLOSUM62 [4] substitution scoring matrix [4] is adopted to calculate the Alignment Score, with gap opening and extension penalties equal to -10 and -0.5, respectively.

For a comparative study, the values of Identity, Similarity, and Alignment Score of the quantitative measurement of homology is performed by various well-known algorithms to be compared to the those of HELIOS method, assuming *ENCODE Transcription Factor Targets Dataset, TFAP2A Gene Set* [3]. It includes Smith-Waterman (SW) [5] reported in Tables A7-A9, Needleman-Wunsch (NW) [6] reported in Tables A10-A12, BLAST [7] reported in Tables A13-A15, ClustalW [8] reported in Tables A16-A18, Clustal-Omega [9] reported in Tables A19-A21, MUSCLE [9] reported in Tables A22-A24, T-Coffee [10] reported in Tables A25-A27, Kalign [11] reported in Tables A28-A30, and MAFFT [12] reported in Tables A31-A33.

Table A1. A brief report of the quantitative measurement of homology of the HELIOS method, compared to nine well-known algorithms, including SW, NW, BLAST, ClustalW, Clustal Omega, Muscle, T-Coffee, Kalign, and MAFFT. The parameters Identity, Similarity, and Alignment score are averaged and reported. The dataset used in this study is the *ENCODE Transcription Factor Targets Dataset*, *TFAP2A Gene Set* [3].

	HELIOS	SW	NW	BLAST	MUSCLE	ClustalW	ClustalΩ	T-Coffee	Kalign	MAFFT
Identity	96.982	96.585	96.493	97.016	97.026	97.110	96.677	97.105	96.784	97.357
Similarity	97.520	96.995	96.575	97.561	97.704	97.572	97.044	97.455	97.281	97.880
Alignment Score	2155.3	2146.9	2190.4	2155.9	2156.9	2154.3	2146.8	2157.1	2150.9	2162.6

Table A2. A brief report of the accuracy measurement of classification output of the HELIOS method with referencing well-known algorithms, including SW, NW, BLAST, ClustalW, Clustal Omega, Muscle, T-Coffee, Kalign, and MAFFT. The parameters SEN, Spec, Acc, PPV, NPV, MCC, and F-Score are averaged and reported. The dataset used in this study is the *ENCODE Transcription Factor Targets Dataset*, *TFAP2A Gene Set* [3].

	SW	NW	BLAST	MUSCLE	ClustalW	ClustalΩ	T-Coffee	Kalign	MAFFT
SEN	0.96481	0.31215	0.96788	0.96513	0.96535	0.96481	0.97446	0.96661	0.96807
Spec	0.99993	0.99854	0.99993	0.99993	0.99993	0.99993	0.99995	0.99993	0.99993
ACC	0.99985	0.99683	0.99986	0.99986	0.99985	0.99985	0.99989	0.99986	0.99986
PPV	0.96579	0.31743	0.96785	0.96611	0.96579	0.96579	0.97467	0.96627	0.96760
NPV	0.99993	0.99828	0.99993	0.99993	0.99993	0.99993	0.99995	0.99993	0.99993
MCC	0.96522	0.31305	0.96779	0.96555	0.96549	0.96522	0.97451	0.96637	0.96776
F-Score	0.96529	0.31447	0.96786	0.96562	0.96556	0.96529	0.97456	0.96644	0.96783

B. Accuracy measurement of classification output

Afterward, the accuracy measurement of the classification output [2] of the HELIOS method is addressed by calculating the values of Sensitivity (SEN), Specificity (Spec), Accuracy (ACC), Positive Predictive Value (PPV), Negative Predictive Value (NPV), Matthew's Coefficient Correlation (MCC), and Test's Accuracy (F-Score) in the simulation studies, according to Eq 5 to Eq 11, respectively.

As a comparative study, the accuracy measurement of the classification output of the HELIOS method is accomplished, assuming *ENCODE Transcription Factor Targets Dataset*, *TFAP2A Gene Set* [3], and the corresponding metrics are calculated with considering Smith-Waterman [5] reported in Tables A34-A40, Needleman-Wunsch [6] reported in Tables A41-A47, ClustalW [8] reported in Tables A55-A61, Clustal-Omega [9] reported in Tables A62-A68, BLAST [7] reported in Tables A48-A54, MUSCLE [13] reported in Tables A69-A75, T-Coffee [10] reported in Tables A76-A82, Kalign [11] reported in Tables A83-A89, and MAFFT [12] reported in Tables A90-A96.

REFERENCES

1. D. S. Moss, S. Jelaska, and S. Pongor, *Essays in bioinformatics*, vol. 368 (IOS Press, 2005).
2. M. Hamada, H. Kiryu, W. Iwasaki, and K. Asai, "Generalized centroid estimators in bioinformatics," PloS one **6**, e16450 (2011).
3. E. P. Consortium *et al.*, "A user's guide to the encyclopedia of dna elements (encode)," PLoS biol **9**, e1001046 (2011).
4. D. W. Mount, "Using blosum in sequence alignments," Cold Spring Harb. Protoc. **2008**, pdb.top39 (2008).
5. H. Zou, S. Tang, C. Yu, H. Fu, Y. Li, and W. Tang, "asw: accelerating smith–waterman algorithm on coupled cpu–gpu architecture," Int. J. Parallel Program. **47**, 388–402 (2019).
6. Y. Jararweh, M. Al-Ayyoub, M. Fakirah, L. Alawneh, and B. B. Gupta, "Improving the performance of the needleman-wunsch algorithm using parallelization and vectorization techniques," Multimed. Tools Appl. **78**, 3961–3977 (2019).
7. G. M. Boratyn, J. Thierry-Mieg, D. Thierry-Mieg, B. Busby, and T. L. Madden, "Magic-blast, an accurate rna-seq aligner for long and short reads," BMC bioinformatics **20**, 1–19 (2019).
8. D. Díaz, F. J. Esteban, P. Hernández, J. A. Caballero, A. Guevara, G. Dorado, and S. Gálvez, "Mc64-clustalwp2: A highly-parallel hybrid strategy to align multiple sequences in many-core architectures," PLOS ONE **9**, 1–12 (2014).
9. F. Sievers and D. G. Higgins, "Clustal omega for making accurate alignments of many protein sequences," Protein Sci. **27**, 135–145 (2018).
10. C. Notredame, D. G. Higgins, and J. Heringa, "T-coffee: a novel method for fast and accurate multiple sequence alignment11edited by j. thornton," J. Mol. Biol. **302**, 205–217 (2000).
11. T. Lassmann, "Kalign 3: multiple sequence alignment of large datasets," (2020).
12. J. Rozewicki, S. Li, K. M. Amada, D. M. Standley, and K. Katoh, "Mafft-dash: integrated protein sequence and structural alignment," Nucleic acids research **47**, W5–W10 (2019).
13. R. C. Edgar, "MUSCLE: multiple sequence alignment with high accuracy and high throughput," Nucleic Acids Res. **32**, 1792–1797 (2004).

Table A3. The list of input sequences, assuming the *ENCODE Transcription Factor Targets Dataset, TFAP2A Gene Set* [3].

Name	Sequence
NP 001027451.1	MLVHS FSAMD RHDGT SNGTA RLPQL GTVGQ SPYTS APPLS HTPNA DFQPP YFPPP YQPIY PQSQD PYSHV NDPYS LNPLH AQPQP QHPGW PGQRQ SQESG LLHTH RGLPH QLSGL DPRRD YRRHE DLLHG PHALS SGLGD LSIHS LPHAI EEVPH VEDPG INIPD QTVIK KGPVS LSKSN SNAVS AIPIN KDNLF GGVVN PNEVF CSVPG RLSLL SSTSK YKVTV AEVQR RLSPP ECLNA SLLGG VLRRA KSKNG GRSLR EKLDK IGLNL PAGRR KAANV TLLTS LVEGE AVHLA RDFGY VCETE FPAKA VAEFL NRQHS DPNEQ VTRKN MLLAT KQICK EFTDL LAQDR SPLGN SRPNP ILEPG IQSCL THFNL ISHGF GSPAV CAAVT ALQNY LTEAL KAMDK MYLSN NPNSH TDNNA KSSDK EEKHR K
NP 001035890.1	MSILA KMGDW QDRHD GTSNG TARLP QLGTV GQSPY TSAPP LSHTP NADFQ PPYFP PPYQP IYPQS QDPYS HVNDP YSLNP LHAQP QPQHP GWPGQ RQSQE SGLLH THRGL PHQLS GLDPR RDYRR HEDLL HGPHA LSSGL GDLSI HSLPH AIEEV PHVED PGINI PDQTV IKKGP VSLSK SNSNA VSAIP INKDN LFGGV VNPNE VFCSV PGRLS LLSST SKYKV TVAEV QRRLS PPECL NASLL GGVLR RAKSK NGGRS LREKL DKIGL NLPAG RRKAA NVTLL TSLVE GEAVH LARDF GYVCE TEFPA KAVAE FLNRQ HSDPN EQVTR KNMLL ATKQI CKEFT DLLAQ DRSPL GNSRP NPILE PGIQS CLTHF NLISH GFGSP AVCAA VTALQ NYLTE ALKAM DKMYL SNNPN SHTDN NAKSS DKEEK HRK
NP 001358995.1	MKMLW KLTDN IKYED CEDRH DGTSN GTARL PQLGT VGQSP YTSAP PLSHT PNADF QPPYF PPPYQ PIYPQ SQDPY SHVND PYSLN PLHAQ PQPQH PGWPG QRQSQ ESGLL HTHRG LPHQL SGLDP RRDYR RHEDL LHGPH ALSSG LGDLS IHSLP HAIEE VPHVE DPGIN IPDQT VIKKG PVSLS KSNSN AVSAI PINKD NLFGG VVNPN EVFCS VPGRL SLLSS TSKYK VTVAE VQRRL SPPEC LNASL LGGVL RRAKS KNGGR SLREK LDKIG LNLPA GRRKA ANVTL LTSLV EGEAV HLARD FGYVC ETEFP AKAVA EFLNR QHSDP NEQVT RKNML LATKQ ICKEF TDLLA QDRSP LGNSR PNPIL EPGIQ SCLTH FNLIS HGFGS PAVCA AVTAL QNYLT EALKA MDKMY LSNNP NSHTD NNAKS SDKEE KHRK
XP 006715238.1	MEEPR SSGQP SPESQ TRLRS GGARG QTGPG SACRA ESPTS ARTRS QAAPT RYARP PLHPE DRHDG TSNGT ARLPQ LGTVG QSPYT SAPPL SHTPN ADFQP PYFPP PYQPI YPQSQ DPYSH VNDPY SLNPL HAQPQ PQHPG WPGQR QSQES GLLHT HRGLP HQLSG LDPRR DYRRH EDLLH GPHAL SSGLG DLSIH SLPHA IEEVP HVEDP GINIP DQTVI KKGPV SLSKS NSNAV SAIPI NKDNL FGGVV NPNEV FCSVP GRLSL LSSTS KYKVT VAEVQ RRLSP PECLN ASLLG GVLRR AKSKN GGRSL REKLD KIGLN LPAGR RKAAN VTLLT SLVEG EAVHL ARDFG YVCET EFPAK AVAEF LNRQH SDPNE QVTRK NMLLA TKQIC KEFTD LLAQD RSPLG NSRPN PILEP GIQSC LTHFN LISHG FGSPA VCAAV TALQN YLTEA LKAMD KMYLS NNPNS HTDNN AKSSD KEEKH RK
XP 011513135.1	MRRQV ATQLF MTRTV TTAPA TGRHG CPSWA LLNPL HAQPQ PQHPG WPGQR QSQES GLLHT HRGLP HQLSG LDPRR DYRRH EDLLH GPHAL SSGLG DLSIH SLPHA IEEVP HVEDP GINIP DQTVI KKGPV SLSKS NSNAV SAIPI NKDNL FGGVV NPNEV FCSVP GRLSL LSSTS KYKVT VAEVQ RRLSP PECLN ASLLG GVLRR AKSKN GGRSL REKLD KIGLN LPAGR RKAAN VTLLT SLVEG EAVHL ARDFG YVCET EFPAK AVAEF LNRQH SDPNE QVTRK NMLLA TKQIC KEFTD LLAQD RSPLG NSRPN PILEP GIQSC LTHFN LISHG FGSPA VCAAV TALQN YLTEA LKAMD KMYLS NNPNS HTDNN AKSSD KEEKH RK
XP 016866721.1	MGRAC RCPTT GVRKH THTRI APSTS SSQSA SVPGP GTDRE SGLGL LGKAG ADGKI AAAIN PSLPR SALCG AGTAA GGVNS WASAH ASAGS NPALY DQDRH DGTSN GTARL PQLGT VGQSP YTSAP PLSHT PNADF QPPYF PPPYQ PIYPQ SQDPY SHVND PYSLN PLHAQ PQPQH PGWPG QRQSQ ESGLL HTHRG LPHQL SGLDP RRDYR RHEDL LHGPH ALSSG LGDLS IHSLP HAIEE VPHVE DPGIN IPDQT VIKKG PVSLS KSNSN AVSAI PINKD NLFGG VVNPN EVFCS VPGRL SLLSS TSKYK VTVAE VQRRL SPPEC LNASL LGGVL RRAKS KNGGR SLREK LDKIG LNLPA GRRKA ANVTL LTSLV EGEAV HLARD FGYVC ETEFP AKAVA EFLNR QHSDP NEQVT RKNML LATKQ ICKEF TDLLA QDRSP LGNSR PNPIL EPGIQ SCLTH FNLIS HGFGS PAVCA AVTAL QNYLT EALKA MDKMY LSNNP NSHTD NNAKS SDKEE KHRK

Table A4. The parameter Identity of the HELIOS method in the quantitative measurement of homology, assuming the *ENCODE Transcription Factor Targets Dataset, TFAP2A Gene Set* [3].

	NP 001027451.1	NP 001035890.1	NP 001358995.1	XP 006715238.1	XP 011513135.1	XP 016866721.1
NP 001027451.1	100	98.1439	98.3759	98.1439	92.7649	98.6079
NP 001035890.1		100	98.3834	97.9215	93.2817	97.9215
NP 001358995.1			100	96.5831	92.5065	96.3554
XP 006715238.1				100	93.0233	90.0415
XP 011513135.1					100	94.5736
XP 016866721.1						100

Table A5. The parameter Similarity of the HELIOS method in the quantitative measurement of homology, assuming the *ENCODE Transcription Factor Targets Dataset, TFAP2A Gene Set* [3].

	NP 001027451.1	NP 001035890.1	NP 001358995.1	XP 006715238.1	XP 011513135.1	XP 016866721.1
NP 001027451.1	100	98.8399	98.6079	98.3759	93.5401	99.0719
NP 001035890.1		100	98.3834	98.3834	95.0904	98.6143
NP 001358995.1			100	97.2665	93.2817	97.2665
XP 006715238.1				100	94.5736	91.2863
XP 011513135.1					100	95.3488
XP 016866721.1						100

Table A6. The parameter Alignment Score of the HELIOS method in the quantitative measurement of homology, assuming the *ENCODE Transcription Factor Targets Dataset, TFAP2A Gene Set* [3].

	NP 001027451.1	NP 001035890.1	NP 001358995.1	XP 006715238.1	XP 011513135.1	XP 016866721.1
NP 001027451.1	2284	2226.5	2217	2199	1800	2174
NP 001035890.1		2299	2238.5	2205	1816.5	2189.5
NP 001358995.1			2337	2200	1797.5	2179.5
XP 006715238.1				2555	1799	2190
XP 011513135.1					2029	1779.5
XP 016866721.1						2746

Table A7. The parameter Identity of the Smith-Waterman algorithm in the quantitative measurement of homology, assuming the *ENCODE Transcription Factor Targets Dataset, TFAP2A Gene Set* [3].

	NP 001027451.1	NP 001035890.1	NP 001358995.1	XP 006715238.1	XP 011513135.1	XP 016866721.1
NP 001027451.1	100	98.1439	97.9118	97.9118	92.2481	97.9118
NP 001035890.1		100	97.9215	97.4596	92.2481	97.6905
NP 001358995.1			100	96.3554	92.2481	96.1276
XP 006715238.1				100	92.2481	89.6266
XP 011513135.1					100	92.2481
XP 016866721.1						100

Table A8. The parameter Similarity of the Smith-Waterman algorithm in the quantitative measurement of homology, assuming the *ENCODE Transcription Factor Targets Dataset, TFAP2A Gene Set* [3].

	NP 001027451.1	NP 001035890.1	NP 001358995.1	XP 006715238.1	XP 011513135.1	XP 016866721.1
NP 001027451.1	100	98.8399	98.3759	98.1439	92.5065	97.9118
NP 001035890.1		100	98.6143	98.3834	92.5065	98.3834
NP 001358995.1			100	96.5831	92.5065	96.8109
XP 006715238.1				100	92.5065	92.3237
XP 011513135.1					100	92.5065
XP 016866721.1						100

Table A9. The parameter Alignment Score of the Smith-Waterman algorithm in the quantitative measurement of homology, assuming the *ENCODE Transcription Factor Targets Dataset, TFAP2A Gene Set* [3].

	NP 001027451.1	NP 001035890.1	NP 001358995.1	XP 006715238.1	XP 011513135.1	XP 016866721.1
NP 001027451.1	2284	2226.5	2215.5	2193	1789.5	2173.5
NP 001035890.1		2299	2231.5	2191	1788.5	2171.5
NP 001358995.1			2337	2187	1785.5	2168.5
XP 006715238.1				2555	1764	2204
XP 011513135.1					2029	1745.5
XP 016866721.1						2746

Table A10. The parameter Identity of the Needleman-Wunsch algorithm in the quantitative measurement of homology, assuming the *ENCODE Transcription Factor Targets Dataset, TFAP2A Gene Set* [3].

	NP 001027451.1	NP 001035890.1	NP 001358995.1	XP 006715238.1	XP 011513135.1	XP 016866721.1
NP 001027451.1	100	97.9118	97.9118	97.9118	91.9897	97.9118
NP 001035890.1		100	97.9215	97.4596	91.9897	97.6905
NP 001358995.1			100	96.3554	91.9897	96.1276
XP 006715238.1				100	91.9897	89.2116
XP 011513135.1					100	91.9897
XP 016866721.1						100

Table A11. The parameter Similarity of the Needleman-Wunsch algorithm in the quantitative measurement of homology, assuming the *ENCODE Transcription Factor Targets Dataset, TFAP2A Gene Set* [3].

	NP 001027451.1	NP 001035890.1	NP 001358995.1	XP 006715238.1	XP 011513135.1	XP 016866721.1
NP 001027451.1	100	97.9118	97.9118	97.9118	91.9897	97.9118
NP 001035890.1		100	98.1524	97.6905	91.9897	97.6905
NP 001358995.1			100	96.3554	91.9897	96.3554
XP 006715238.1				100	91.9897	90.249
XP 011513135.1					100	91.9897
XP 016866721.1						100

Table A12. The parameter Alignment Score of the Needleman-Wunsch algorithm in the quantitative measurement of homology, assuming the *ENCODE Transcription Factor Targets Dataset, TFAP2A Gene Set* [3].

	NP 001027451.1	NP 001035890.1	NP 001358995.1	XP 006715238.1	XP 011513135.1	XP 016866721.1
NP 001027451.1	2284	2240	2240	2240	1863	2240
NP 001035890.1		2299	2248	2242	1863	2245
NP 001358995.1			2337	2245	1863	2242
XP 006715238.1				2555	1863	2253
XP 011513135.1					2029	1863
XP 016866721.1						2746

Table A13. The parameter Identity of the BLAST in the quantitative measurement of homology, assuming the *ENCODE Transcription Factor Targets Dataset, TFAP2A Gene Set* [3].

	NP 001027451.1	NP 001035890.1	NP 001358995.1	XP 006715238.1	XP 011513135.1	XP 016866721.1
NP 001027451.1	100	98.1439	98.3759	97.9118	93.5401	98.3759
NP 001035890.1		100	98.3834	97.6905	93.5401	98.1524
NP 001358995.1			100	96.5831	93.5401	96.8109
XP 006715238.1				100	93.5401	89.2116
XP 011513135.1					100	93.5401
XP 016866721.1						100

Table A14. The parameter Similarity of the BLAST in the quantitative measurement of homology, assuming the *ENCODE Transcription Factor Targets Dataset, TFAP2A Gene Set* [3].

	NP 001027451.1	NP 001035890.1	NP 001358995.1	XP 006715238.1	XP 011513135.1	XP 016866721.1
NP 001027451.1	100	98.8399	98.8399	97.9118	94.5736	98.8399
NP 001035890.1		100	98.8453	98.1524	94.5736	98.8453
NP 001358995.1			100	97.2665	94.5736	97.7221
XP 006715238.1				100	94.5736	90.6639
XP 011513135.1					100	94.5736
XP 016866721.1						100

Table A15. The parameter Alignment Score of the BLAST in the quantitative measurement of homology, assuming the *ENCODE Transcription Factor Targets Dataset, TFAP2A Gene Set* [3].

	NP 001027451.1	NP 001035890.1	NP 001358995.1	XP 006715238.1	XP 011513135.1	XP 016866721.1
NP 001027451.1	2284	2226.5	2221	2173	1820.5	2170.5
NP 001035890.1		2299	2244.5	2193.5	1819.5	2182.5
NP 001358995.1			2337	2200	1816.5	2185
XP 006715238.1				2555	1795	2200.5
XP 011513135.1					2029	1776.5
XP 016866721.1						2746

Table A16. The parameter Identity of the ClustalW in the quantitative measurement of homology, assuming the *ENCODE Transcription Factor Targets Dataset*, *TFAP2A Gene Set* [3].

	NP 001027451.1	NP 001035890.1	NP 001358995.1	XP 006715238.1	XP 011513135.1	XP 016866721.1
NP 001027451.1	100	98.1439	97.9118	97.9118	94.5736	97.9118
NP 001035890.1		100	97.9215	97.4596	94.3152	97.6905
NP 001358995.1			100	96.3554	94.3152	96.1276
XP 006715238.1				100	94.3152	90.0415
XP 011513135.1					100	94.3152
XP 016866721.1						100

Table A17. The parameter Similarity of the ClustalW in the quantitative measurement of homology, assuming the *ENCODE Transcription Factor Targets Dataset*, *TFAP2A Gene Set* [3].

	NP 001027451.1	NP 001035890.1	NP 001358995.1	XP 006715238.1	XP 011513135.1	XP 016866721.1
NP 001027451.1	100	98.8399	98.3759	98.1439	95.0904	97.9118
NP 001035890.1		100	98.3834	98.3834	94.832	98.3834
NP 001358995.1			100	97.0387	94.832	96.8109
XP 006715238.1				100	94.832	92.3237
XP 011513135.1					100	94.832
XP 016866721.1						100

Table A18. The parameter Alignment Score of the ClustalW in the quantitative measurement of homology, assuming the *ENCODE Transcription Factor Targets Dataset*, *TFAP2A Gene Set* [3].

	NP 001027451.1	NP 001035890.1	NP 001358995.1	XP 006715238.1	XP 011513135.1	XP 016866721.1
NP 001027451.1	2284	2226.5	2219.5	2193	1824.5	2173.5
NP 001035890.1		2299	2220	2191	1818.5	2171.5
NP 001358995.1			2337	2184.5	1813.5	2159
XP 006715238.1				2555	1792	2228.5
XP 011513135.1					2029	1775.5
XP 016866721.1						2746

Table A19. The parameter Identity of the ClustalΩ in the quantitative measurement of homology, assuming the *ENCODE Transcription Factor Targets Dataset*, *TFAP2A Gene Set* [3].

	NP 001027451.1	NP 001035890.1	NP 001358995.1	XP 006715238.1	XP 011513135.1	XP 016866721.1
NP 001027451.1	100	98.1439	97.9118	97.9118	92.5065	97.9118
NP 001035890.1		100	97.9215	97.4596	92.5065	97.6905
NP 001358995.1			100	96.3554	92.5065	96.3554
XP 006715238.1				100	92.5065	90.0415
XP 011513135.1					100	92.5065
XP 016866721.1						100

Table A20. The parameter Similarity of the ClustalΩ in the quantitative measurement of homology, assuming the *ENCODE Transcription Factor Targets Dataset*, *TFAP2A Gene Set* [3].

	NP 001027451.1	NP 001035890.1	NP 001358995.1	XP 006715238.1	XP 011513135.1	XP 016866721.1
NP 001027451.1	100	98.8399	98.3759	98.1439	92.7649	97.9118
NP 001035890.1		100	98.6143	98.3834	92.7649	98.1524
NP 001358995.1			100	96.5831	92.7649	96.5831
XP 006715238.1				100	92.7649	92.5311
XP 011513135.1					100	92.7649
XP 016866721.1						100

Table A21. The parameter Alignment Score of the ClustalΩ in the quantitative measurement of homology, assuming the *ENCODE Transcription Factor Targets Dataset, TFAP2A Gene Set* [3].

	NP 001027451.1	NP 001035890.1	NP 001358995.1	XP 006715238.1	XP 011513135.1	XP 016866721.1
NP 001027451.1	2284	2226.5	2215.5	2183.5	1794	2165
NP 001035890.1		2299	2231.5	2184.5	1793	2163
NP 001358995.1			2337	2177.5	1790	2167
XP 006715238.1				2555	1768.5	2225
XP 011513135.1					2029	1750
XP 016866721.1						2746

Table A22. The parameter Identity of the MUSCLE in the quantitative measurement of homology, assuming the *ENCODE Transcription Factor Targets Dataset, TFAP2A Gene Set* [3].

	NP 001027451.1	NP 001035890.1	NP 001358995.1	XP 006715238.1	XP 011513135.1	XP 016866721.1
NP 001027451.1	100	97.9118	97.9118	97.9118	93.7984	98.3759
NP 001035890.1		100	98.3834	97.6905	93.5401	98.3834
NP 001358995.1			100	96.5831	93.5401	96.8109
XP 006715238.1				100	93.5401	89.6266
XP 011513135.1					100	93.5401
XP 016866721.1						100

Table A23. The parameter Similarity of the MUSCLE in the quantitative measurement of homology, assuming the *ENCODE Transcription Factor Targets Dataset, TFAP2A Gene Set* [3].

	NP 001027451.1	NP 001035890.1	NP 001358995.1	XP 006715238.1	XP 011513135.1	XP 016866721.1
NP 001027451.1	100	98.6079	98.3759	98.1439	95.0904	98.6079
NP 001035890.1		100	98.6143	98.6143	94.832	98.8453
NP 001358995.1			100	97.7221	94.832	97.7221
XP 006715238.1				100	94.832	92.1162
XP 011513135.1					100	94.832
XP 016866721.1						100

Table A24. The parameter Alignment Score of the MUSCLE in the quantitative measurement of homology, assuming the *ENCODE Transcription Factor Targets Dataset, TFAP2A Gene Set* [3].

	NP 001027451.1	NP 001035890.1	NP 001358995.1	XP 006715238.1	XP 011513135.1	XP 016866721.1
NP 001027451.1	2284	2218	2214	2182.5	1822.5	2179
NP 001035890.1		2299	2243.5	2195.5	1816.5	2195
NP 001358995.1			2337	2195.5	1811.5	2185
XP 006715238.1				2555	1790	2224.5
XP 011513135.1					2029	1773.5
XP 016866721.1						2746

Table A25. The parameter Identity of the T-Coffee in the quantitative measurement of homology, assuming the *ENCODE Transcription Factor Targets Dataset, TFAP2A Gene Set* [3].

	NP 001027451.1	NP 001035890.1	NP 001358995.1	XP 006715238.1	XP 011513135.1	XP 016866721.1
NP 001027451.1	100	98.1439	98.1439	98.1439	93.7984	98.1439
NP 001035890.1		100	98.3834	97.6905	93.7984	97.9215
NP 001358995.1			100	96.5831	93.7984	96.3554
XP 006715238.1				100	93.7984	90.4564
XP 011513135.1					100	94.0568
XP 016866721.1						100

Table A26. The parameter Similarity of the T-Coffee in the quantitative measurement of homology, assuming the *ENCODE Transcription Factor Targets Dataset, TFAP2A Gene Set* [3].

	NP 001027451.1	NP 001035890.1	NP 001358995.1	XP 006715238.1	XP 011513135.1	XP 016866721.1
NP 001027451.1	100	98.3759	98.1439	98.1439	94.0568	98.6079
NP 001035890.1		100	98.6143	98.3834	94.3152	98.6143
NP 001358995.1			100	96.8109	94.3152	97.4943
XP 006715238.1				100	94.0568	92.3237
XP 011513135.1					100	94.3152
XP 016866721.1						100

Table A27. The parameter Alignment Score of the T-Coffee in the quantitative measurement of homology, assuming the *ENCODE Transcription Factor Targets Dataset, TFAP2A Gene Set* [3].

	NP 001027451.1	NP 001035890.1	NP 001358995.1	XP 006715238.1	XP 011513135.1	XP 016866721.1
NP 001027451.1	2284	2227.5	2219.5	2198	1816.5	2177.5
NP 001035890.1		2299	2245.5	2196	1816.5	2174
NP 001358995.1			2337	2192	1815.5	2164
XP 006715238.1				2555	1796	2230.5
XP 011513135.1					2029	1780.5
XP 016866721.1						2746

Table A28. The parameter Identity of the Kalign in the quantitative measurement of homology, assuming the *ENCODE Transcription Factor Targets Dataset, TFAP2A Gene Set* [3].

	NP 001027451.1	NP 001035890.1	NP 001358995.1	XP 006715238.1	XP 011513135.1	XP 016866721.1
NP 001027451.1	100	97.9118	97.9118	97.9118	92.7649	97.9118
NP 001035890.1		100	98.3834	97.4596	92.7649	98.3834
NP 001358995.1			100	96.3554	92.7649	96.5831
XP 006715238.1				100	92.7649	89.834
XP 011513135.1					100	92.7649
XP 016866721.1						100

Table A29. The parameter Similarity of the Kalign in the quantitative measurement of homology, assuming the *ENCODE Transcription Factor Targets Dataset, TFAP2A Gene Set* [3].

	NP 001027451.1	NP 001035890.1	NP 001358995.1	XP 006715238.1	XP 011513135.1	XP 016866721.1
NP 001027451.1	100	97.9118	98.3759	98.1439	93.7984	97.9118
NP 001035890.1		100	98.6143	98.3834	93.7984	98.6143
NP 001358995.1			100	96.8109	93.7984	97.0387
XP 006715238.1				100	93.7984	92.1162
XP 011513135.1					100	93.7984
XP 016866721.1						100

Table A30. The parameter Alignment Score of the Kalign in the quantitative measurement of homology, assuming the *ENCODE Transcription Factor Targets Dataset, TFAP2A Gene Set* [3].

	NP 001027451.1	NP 001035890.1	NP 001358995.1	XP 006715238.1	XP 011513135.1	XP 016866721.1
NP 001027451.1	2284	2205	2205	2193	1802.5	2173.5
NP 001035890.1		2299	2243.5	2184.5	1801.5	2187
NP 001358995.1			2337	2184.5	1798.5	2176
XP 006715238.1				2555	1777	2230.5
XP 011513135.1					2029	1758.5
XP 016866721.1						2746

Table A31. The parameter Identity of the MAFFT in the quantitative measurement of homology, assuming the *ENCODE Transcription Factor Targets Dataset, TFAP2A Gene Set* [3].

	NP 001027451.1	NP 001035890.1	NP 001358995.1	XP 006715238.1	XP 011513135.1	XP 016866721.1
NP 001027451.1	100	98.1439	98.1439	98.6079	94.3152	98.6079
NP 001035890.1		100	98.3834	98.1524	94.3152	98.6143
NP 001358995.1			100	96.8109	94.3152	96.8109
XP 006715238.1				100	94.3152	90.6639
XP 011513135.1					100	94.3152
XP 016866721.1						100

Table A32. The parameter Similarity of the MAFFT in the quantitative measurement of homology, assuming the *ENCODE Transcription Factor Targets Dataset, TFAP2A Gene Set* [3].

	NP 001027451.1	NP 001035890.1	NP 001358995.1	XP 006715238.1	XP 011513135.1	XP 016866721.1
NP 001027451.1	100	98.1439	98.6079	98.8399	95.3488	99.0719
NP 001035890.1		100	98.6143	98.6143	95.3488	99.0762
NP 001358995.1			100	97.4943	95.3488	97.9499
XP 006715238.1				100	95.3488	92.3237
XP 011513135.1					100	95.3488
XP 016866721.1						100

Table A33. The parameter Alignment Score of the MAFFT in the quantitative measurement of homology, assuming the *ENCODE Transcription Factor Targets Dataset, TFAP2A Gene Set* [3].

	NP 001027451.1	NP 001035890.1	NP 001358995.1	XP 006715238.1	XP 011513135.1	XP 016866721.1
NP 001027451.1	2284	2227.5	2225.5	2202.5	1829	2190
NP 001035890.1		2299	2243.5	2202.5	1828	2198
NP 001358995.1			2337	2190	1825	2176.5
XP 006715238.1				2555	1803.5	2239.5
XP 011513135.1					2029	1785
XP 016866721.1						2746

Table A34. The parameter Sensitivity (SEN) of the HELIOS method with referencing the Smith-Waterman algorithm in the accuracy measurement of classification output, assuming the *ENCODE Transcription Factor Targets Dataset, TFAP2A Gene Set* [3].

	NP 001027451.1	NP 001035890.1	NP 001358995.1	XP 006715238.1	XP 011513135.1	XP 016866721.1
NP 001027451.1	1	0.97912	0.97912	0.99768	0.9199	0.97912
NP 001035890.1		1	0.9746	0.9746	0.9199	0.97691
NP 001358995.1			1	0.96355	0.9199	0.96128
XP 006715238.1				1	0.9199	0.87552
XP 011513135.1					1	0.9199
XP 016866721.1						1

Table A35. The parameter Specification (Spec) of the HELIOS method with referencing the Smith-Waterman algorithm in the accuracy measurement of classification output, assuming the *ENCODE Transcription Factor Targets Dataset, TFAP2A Gene Set* [3].

	NP 001027451.1	NP 001035890.1	NP 001358995.1	XP 006715238.1	XP 011513135.1	XP 016866721.1
NP 001027451.1	1	0.99995	0.99995	1	0.99981	0.99996
NP 001035890.1		1	0.99994	0.99995	0.99981	0.99996
NP 001358995.1			1	0.99992	0.99982	0.99993
XP 006715238.1				1	0.99983	0.99977
XP 011513135.1					1	0.99988
XP 016866721.1						1

Table A36. The parameter Accuracy (Acc) of the HELIOS method with referencing the Smith-Waterman algorithm in the accuracy measurement of classification output, assuming the *ENCODE Transcription Factor Targets Dataset, TFAP2A Gene Set* [3].

	NP 001027451.1	NP 001035890.1	NP 001358995.1	XP 006715238.1	XP 011513135.1	XP 016866721.1
NP 001027451.1	1	0.9999	0.9999	0.99999	0.99963	0.99992
NP 001035890.1		1	0.99988	0.99989	0.99963	0.99991
NP 001358995.1			1	0.99985	0.99964	0.99985
XP 006715238.1				1	0.99967	0.99953
XP 011513135.1					1	0.99973
XP 016866721.1						1

Table A37. The parameter Positive Predictive Value (PPV) of the HELIOS method with referencing the Smith-Waterman algorithm in the accuracy measurement of classification output, assuming the *ENCODE Transcription Factor Targets Dataset, TFAP2A Gene Set* [3].

	NP 001027451.1	NP 001035890.1	NP 001358995.1	XP 006715238.1	XP 011513135.1	XP 016866721.1
NP 001027451.1	1	0.97912	0.97912	0.99768	0.9199	0.97912
NP 001035890.1		1	0.9746	0.9746	0.9199	0.97691
NP 001358995.1			1	0.96355	0.9199	0.96128
XP 006715238.1				1	0.9199	0.87917
XP 011513135.1					1	0.93684
XP 016866721.1						1

Table A38. The parameter Negative Predictive Value (NPV) of the HELIOS method with referencing the Smith-Waterman algorithm in the accuracy measurement of classification output, assuming the *ENCODE Transcription Factor Targets Dataset, TFAP2A Gene Set* [3].

	NP 001027451.1	NP 001035890.1	NP 001358995.1	XP 006715238.1	XP 011513135.1	XP 016866721.1
NP 001027451.1	1	0.99995	0.99995	1	0.99981	0.99996
NP 001035890.1		1	0.99994	0.99995	0.99981	0.99996
NP 001358995.1			1	0.99992	0.99982	0.99993
XP 006715238.1				1	0.99983	0.99976
XP 011513135.1					1	0.99985
XP 016866721.1						1

Table A39. The parameter Matthew's Coefficient Correlation (MCC) of the HELIOS method with referencing the Smith-Waterman algorithm in the accuracy measurement of classification output, assuming the *ENCODE Transcription Factor Targets Dataset, TFAP2A Gene Set* [3].

	NP 001027451.1	NP 001035890.1	NP 001358995.1	XP 006715238.1	XP 011513135.1	XP 016866721.1
NP 001027451.1	1	0.97907	0.97907	0.99767	0.91971	0.97908
NP 001035890.1		1	0.97454	0.97454	0.91971	0.97686
NP 001358995.1			1	0.96348	0.91971	0.9612
XP 006715238.1				1	0.91973	0.8771
XP 011513135.1					1	0.92819
XP 016866721.1						1

Table A40. The parameter Test's Accuracy (F-Score) of the HELIOS method with referencing the Smith-Waterman algorithm in the accuracy measurement of classification output, assuming the *ENCODE Transcription Factor Targets Dataset, TFAP2A Gene Set* [3].

	NP 001027451.1	NP 001035890.1	NP 001358995.1	XP 006715238.1	XP 011513135.1	XP 016866721.1
NP 001027451.1	1	0.97912	0.97912	0.99768	0.9199	0.97912
NP 001035890.1		1	0.9746	0.9746	0.9199	0.97691
NP 001358995.1			1	0.96355	0.9199	0.96128
XP 006715238.1				1	0.9199	0.87734
XP 011513135.1					1	0.92829
XP 016866721.1						1

Table A41. The parameter Sensitivity (SEN) of the HELIOS method with referencing the Needleman-Wunsch algorithm in the accuracy measurement of classification output, assuming the *ENCODE Transcription Factor Targets Dataset, TFAP2A Gene Set* [3].

	NP 001027451.1	NP 001035890.1	NP 001358995.1	XP 006715238.1	XP 011513135.1	XP 016866721.1
NP 001027451.1	1	0.021277	0	0.0023697	0.044944	0.014184
NP 001035890.1		1	0.025701	0.026005	0.087079	0.023641
NP 001358995.1			1	0.037825	0.087079	0.040189
XP 006715238.1				1	0.087079	0.035242
XP 011513135.1					1	0.022472
XP 016866721.1						1

Table A42. The parameter Specification (Spec) of the HELIOS method with referencing the Needleman-Wunsch algorithm in the accuracy measurement of classification output, assuming the *ENCODE Transcription Factor Targets Dataset, TFAP2A Gene Set* [3].

	NP 001027451.1	NP 001035890.1	NP 001358995.1	XP 006715238.1	XP 011513135.1	XP 016866721.1
NP 001027451.1	1	0.99769	0.99767	0.99792	0.99766	0.99816
NP 001035890.1		1	0.99775	0.99797	0.99779	0.99817
NP 001358995.1			1	0.99796	0.99784	0.99817
XP 006715238.1				1	0.99818	0.99806
XP 011513135.1					1	0.99835
XP 016866721.1						1

Table A43. The parameter Accuracy (Acc) of the HELIOS method with referencing the Needleman-Wunsch algorithm in the accuracy measurement of classification output, assuming the *ENCODE Transcription Factor Targets Dataset, TFAP2A Gene Set* [3].

	NP 001027451.1	NP 001035890.1	NP 001358995.1	XP 006715238.1	XP 011513135.1	XP 016866721.1
NP 001027451.1	1	0.99538	0.99531	0.99556	0.99498	0.99583
NP 001035890.1		1	0.99548	0.99567	0.99523	0.99586
NP 001358995.1			1	0.99569	0.99528	0.99591
XP 006715238.1				1	0.99562	0.99595
XP 011513135.1					1	0.99561
XP 016866721.1						1

Table A44. The parameter Positive Predictive Value (PPV) of the HELIOS method with referencing the Needleman-Wunsch algorithm in the accuracy measurement of classification output, assuming the *ENCODE Transcription Factor Targets Dataset, TFAP2A Gene Set* [3].

	NP 001027451.1	NP 001035890.1	NP 001358995.1	XP 006715238.1	XP 011513135.1	XP 016866721.1
NP 001027451.1	1	0.021378	0	0.0026954	0.051282	0.01791
NP 001035890.1		1	0.026066	0.029412	0.1	0.029674
NP 001358995.1			1	0.042105	0.10197	0.049563
XP 006715238.1				1	0.11877	0.038369
XP 011513135.1					1	0.036866
XP 016866721.1						1

Table A45. The parameter Negative Predictive Value (NPV) of the HELIOS method with referencing the Needleman-Wunsch algorithm in the accuracy measurement of classification output, assuming the *ENCODE Transcription Factor Targets Dataset, TFAP2A Gene Set* [3].

	NP 001027451.1	NP 001035890.1	NP 001358995.1	XP 006715238.1	XP 011513135.1	XP 016866721.1
NP 001027451.1	1	0.99768	0.99762	0.99763	0.99731	0.99767
NP 001035890.1		1	0.99772	0.99769	0.99743	0.99769
NP 001358995.1			1	0.99772	0.99743	0.99773
XP 006715238.1				1	0.99743	0.99788
XP 011513135.1					1	0.99725
XP 016866721.1						1

Table A46. The parameter Matthew's Coefficient Correlation (MCC) of the HELIOS method with referencing the Needleman-Wunsch algorithm in the accuracy measurement of classification output, assuming the *ENCODE Transcription Factor Targets Dataset, TFAP2A Gene Set* [3].

	NP 001027451.1	NP 001035890.1	NP 001358995.1	XP 006715238.1	XP 011513135.1	XP 016866721.1
NP 001027451.1	1	0.019013	-0.0023526	0.00030611	0.045499	0.013864
NP 001035890.1		1	0.023618	0.02549	0.090934	0.024428
NP 001358995.1			1	0.037752	0.091876	0.042593
XP 006715238.1				1	0.099536	0.034744
XP 011513135.1					1	0.02665
XP 016866721.1						1

Table A47. The parameter Test's Accuracy (F-Score) of the HELIOS method with referencing the Needleman-Wunsch algorithm in the accuracy measurement of classification output, assuming the *ENCODE Transcription Factor Targets Dataset, TFAP2A Gene Set* [3].

	NP 001027451.1	NP 001035890.1	NP 001358995.1	XP 006715238.1	XP 011513135.1	XP 016866721.1
NP 001027451.1	1	0.021327	0	0.0025221	0.047904	0.015831
NP 001035890.1		1	0.025882	0.027604	0.093093	0.026316
NP 001358995.1			1	0.039851	0.093939	0.044386
XP 006715238.1				1	0.10049	0.036739
XP 011513135.1					1	0.027923
XP 016866721.1						1

Table A48. The parameter Sensitivity (SEN) of the HELIOS method with referencing the BLAST in the accuracy measurement of classification output, assuming the *ENCODE Transcription Factor Targets Dataset, TFAP2A Gene Set* [3].

	NP 001027451.1	NP 001035890.1	NP 001358995.1	XP 006715238.1	XP 011513135.1	XP 016866721.1
NP 001027451.1	1	0.97912	0.98376	0.98144	0.92228	0.97912
NP 001035890.1		1	0.98383	0.98383	0.92228	0.97691
NP 001358995.1			1	1	0.92228	0.96128
XP 006715238.1				1	0.92228	0.8847
XP 011513135.1					1	0.92228
XP 016866721.1						1

Table A49. The parameter Specification (Spec) of the HELIOS method with referencing the BLAST in the accuracy measurement of classification output, assuming the *ENCODE Transcription Factor Targets Dataset, TFAP2A Gene Set* [3].

	NP 001027451.1	NP 001035890.1	NP 001358995.1	XP 006715238.1	XP 011513135.1	XP 016866721.1
NP 001027451.1	1	0.99995	0.99996	0.99996	0.99981	0.99996
NP 001035890.1		1	0.99996	0.99997	0.99981	0.99996
NP 001358995.1			1	1	0.99982	0.99993
XP 006715238.1				1	0.99983	0.99977
XP 011513135.1					1	0.99988
XP 016866721.1						1

Table A50. The parameter Accuracy (Acc) of the HELIOS method with referencing the BLAST in the accuracy measurement of classification output, assuming the *ENCODE Transcription Factor Targets Dataset, TFAP2A Gene Set* [3].

	NP 001027451.1	NP 001035890.1	NP 001358995.1	XP 006715238.1	XP 011513135.1	XP 016866721.1
NP 001027451.1	1	0.9999	0.99993	0.99992	0.99963	0.99992
NP 001035890.1		1	0.99993	0.99993	0.99964	0.99991
NP 001358995.1			1	1	0.99964	0.99985
XP 006715238.1				1	0.99967	0.99955
XP 011513135.1					1	0.99973
XP 016866721.1						1

Table A51. The parameter Positive Predictive Value (PPV) of the HELIOS method with referencing the BLAST in the accuracy measurement of classification output, assuming the *ENCODE Transcription Factor Targets Dataset, TFAP2A Gene Set* [3].

	NP 001027451.1	NP 001035890.1	NP 001358995.1	XP 006715238.1	XP 011513135.1	XP 016866721.1
NP 001027451.1	1	0.97912	0.98376	0.98144	0.9199	0.97912
NP 001035890.1		1	0.98383	0.98383	0.9199	0.97691
NP 001358995.1			1	1	0.9199	0.96128
XP 006715238.1				1	0.9199	0.87917
XP 011513135.1					1	0.93684
XP 016866721.1						1

Table A52. The parameter Negative Predictive Value (NPV) of the HELIOS method with referencing the BLAST in the accuracy measurement of classification output, assuming the *ENCODE Transcription Factor Targets Dataset, TFAP2A Gene Set* [3].

	NP 001027451.1	NP 001035890.1	NP 001358995.1	XP 006715238.1	XP 011513135.1	XP 016866721.1
NP 001027451.1	1	0.99995	0.99996	0.99996	0.99982	0.99996
NP 001035890.1		1	0.99996	0.99997	0.99982	0.99996
NP 001358995.1			1	1	0.99982	0.99993
XP 006715238.1				1	0.99984	0.99978
XP 011513135.1					1	0.99985
XP 016866721.1						1

Table A53. The parameter Matthew's Coefficient Correlation (MCC) of the HELIOS method with referencing the BLAST in the accuracy measurement of classification output, assuming the *ENCODE Transcription Factor Targets Dataset, TFAP2A Gene Set* [3].

	NP 001027451.1	NP 001035890.1	NP 001358995.1	XP 006715238.1	XP 011513135.1	XP 016866721.1
NP 001027451.1	1	0.97907	0.98372	0.9814	0.9209	0.97908
NP 001035890.1		1	0.9838	0.9838	0.92091	0.97686
NP 001358995.1			1	1	0.92091	0.9612
XP 006715238.1				1	0.92092	0.8817
XP 011513135.1					1	0.9294
XP 016866721.1						1

Table A54. The parameter Test's Accuracy (F-Score) of the HELIOS method with referencing the BLAST in the accuracy measurement of classification output, assuming the *ENCODE Transcription Factor Targets Dataset, TFAP2A Gene Set* [3].

	NP 001027451.1	NP 001035890.1	NP 001358995.1	XP 006715238.1	XP 011513135.1	XP 016866721.1
NP 001027451.1	1	0.97912	0.98376	0.98144	0.92109	0.97912
NP 001035890.1		1	0.98383	0.98383	0.92109	0.97691
NP 001358995.1			1	1	0.92109	0.96128
XP 006715238.1				1	0.92109	0.88192
XP 011513135.1					1	0.9295
XP 016866721.1						1

Table A55. The parameter Sensitivity (SEN) of the HELIOS method with referencing the ClustalW in the accuracy measurement of classification output, assuming the *ENCODE Transcription Factor Targets Dataset, TFAP2A Gene Set* [3].

	NP 001027451.1	NP 001035890.1	NP 001358995.1	XP 006715238.1	XP 011513135.1	XP 016866721.1
NP 001027451.1	1	0.97912	0.98368	0.99768	0.9199	0.97912
NP 001035890.1		1	0.9814	0.9746	0.9199	0.97691
NP 001358995.1			1	0.96355	0.9199	0.96128
XP 006715238.1				1	0.9199	0.87552
XP 011513135.1					1	0.9199
XP 016866721.1						1

Table A56. The parameter Specification (Spec) of the HELIOS method with referencing the ClustalW in the accuracy measurement of classification output, assuming the *ENCODE Transcription Factor Targets Dataset, TFAP2A Gene Set* [3].

	NP 001027451.1	NP 001035890.1	NP 001358995.1	XP 006715238.1	XP 011513135.1	XP 016866721.1
NP 001027451.1	1	0.99995	0.99995	1	0.99981	0.99996
NP 001035890.1		1	0.99994	0.99995	0.99981	0.99996
NP 001358995.1			1	0.99992	0.99982	0.99993
XP 006715238.1				1	0.99983	0.99977
XP 011513135.1					1	0.99988
XP 016866721.1						1

Table A57. The parameter Accuracy (Acc) of the HELIOS method with referencing the ClustalW in the accuracy measurement of classification output, assuming the *ENCODE Transcription Factor Targets Dataset, TFAP2A Gene Set* [3].

	NP 001027451.1	NP 001035890.1	NP 001358995.1	XP 006715238.1	XP 011513135.1	XP 016866721.1
NP 001027451.1	1	0.9999	0.99992	0.99999	0.99963	0.99992
NP 001035890.1		1	0.9999	0.99989	0.99963	0.99991
NP 001358995.1			1	0.99985	0.99964	0.99985
XP 006715238.1				1	0.99967	0.99953
XP 011513135.1					1	0.99973
XP 016866721.1						1

Table A58. The parameter Positive Predictive Value (PPV) of the HELIOS method with referencing the ClustalW in the accuracy measurement of classification output, assuming the *ENCODE Transcription Factor Targets Dataset*, *TFAP2A Gene Set* [3].

	NP 001027451.1	NP 001035890.1	NP 001358995.1	XP 006715238.1	XP 011513135.1	XP 016866721.1
NP 001027451.1	1	0.97912	0.97912	0.99768	0.9199	0.97912
NP 001035890.1		1	0.9746	0.9746	0.9199	0.97691
NP 001358995.1			1	0.96355	0.9199	0.96128
XP 006715238.1				1	0.9199	0.87917
XP 011513135.1					1	0.93684
XP 016866721.1						1

Table A59. The parameter Negative Predictive Value (NPV) of the HELIOS method with referencing the ClustalW in the accuracy measurement of classification output, assuming the *ENCODE Transcription Factor Targets Dataset*, *TFAP2A Gene Set* [3].

	NP 001027451.1	NP 001035890.1	NP 001358995.1	XP 006715238.1	XP 011513135.1	XP 016866721.1
NP 001027451.1	1	0.99995	0.99996	1	0.99981	0.99996
NP 001035890.1		1	0.99996	0.99995	0.99981	0.99996
NP 001358995.1			1	0.99992	0.99982	0.99993
XP 006715238.1				1	0.99983	0.99976
XP 011513135.1					1	0.99985
XP 016866721.1						1

Table A60. The parameter Matthew's Coefficient Correlation (MCC) of the HELIOS method with referencing the ClustalW in the accuracy measurement of classification output, assuming the *ENCODE Transcription Factor Targets Dataset*, *TFAP2A Gene Set* [3].

	NP 001027451.1	NP 001035890.1	NP 001358995.1	XP 006715238.1	XP 011513135.1	XP 016866721.1
NP 001027451.1	1	0.97907	0.98136	0.99767	0.91971	0.97908
NP 001035890.1		1	0.97794	0.97454	0.91971	0.97686
NP 001358995.1			1	0.96348	0.91971	0.9612
XP 006715238.1				1	0.91973	0.8771
XP 011513135.1					1	0.92819
XP 016866721.1						1

Table A61. The parameter Test's Accuracy (F-Score) of the HELIOS method with referencing the ClustalW in the accuracy measurement of classification output, assuming the *ENCODE Transcription Factor Targets Dataset*, *TFAP2A Gene Set* [3].

	NP 001027451.1	NP 001035890.1	NP 001358995.1	XP 006715238.1	XP 011513135.1	XP 016866721.1
NP 001027451.1	1	0.97912	0.9814	0.99768	0.9199	0.97912
NP 001035890.1		1	0.97798	0.9746	0.9199	0.97691
NP 001358995.1			1	0.96355	0.9199	0.96128
XP 006715238.1				1	0.9199	0.87734
XP 011513135.1					1	0.92829
XP 016866721.1						1

Table A62. The parameter Sensitivity (SEN) of the HELIOS method with referencing the ClustalΩ in the accuracy measurement of classification output, assuming the *ENCODE Transcription Factor Targets Dataset*, *TFAP2A Gene Set* [3].

	NP 001027451.1	NP 001035890.1	NP 001358995.1	XP 006715238.1	XP 011513135.1	XP 016866721.1
NP 001027451.1	1	0.97912	0.97912	0.99768	0.9199	0.97912
NP 001035890.1		1	0.9746	0.9746	0.9199	0.97691
NP 001358995.1			1	0.96355	0.9199	0.96128
XP 006715238.1				1	0.9199	0.87552
XP 011513135.1					1	0.9199
XP 016866721.1						1

Table A63. The parameter Specification (Spec) of the HELIOS method with referencing the ClustalΩ in the accuracy measurement of classification output, assuming the *ENCODE Transcription Factor Targets Dataset*, *TFAP2A Gene Set* [3].

	NP 001027451.1	NP 001035890.1	NP 001358995.1	XP 006715238.1	XP 011513135.1	XP 016866721.1
NP 001027451.1	1	0.99995	0.99995	1	0.99981	0.99996
NP 001035890.1		1	0.99994	0.99995	0.99981	0.99996
NP 001358995.1			1	0.99992	0.99982	0.99993
XP 006715238.1				1	0.99983	0.99977
XP 011513135.1					1	0.99988
XP 016866721.1						1

Table A64. The parameter Accuracy (Acc) of the HELIOS method with referencing the ClustalΩ in the accuracy measurement of classification output, assuming the *ENCODE Transcription Factor Targets Dataset*, *TFAP2A Gene Set* [3].

	NP 001027451.1	NP 001035890.1	NP 001358995.1	XP 006715238.1	XP 011513135.1	XP 016866721.1
NP 001027451.1	1	0.9999	0.9999	0.99999	0.99963	0.99992
NP 001035890.1		1	0.99988	0.99989	0.99963	0.99991
NP 001358995.1			1	0.99985	0.99964	0.99985
XP 006715238.1				1	0.99967	0.99953
XP 011513135.1					1	0.99973
XP 016866721.1						1

Table A65. The parameter Positive Predictive Value (PPV) of the HELIOS method with referencing the ClustalΩ in the accuracy measurement of classification output, assuming the *ENCODE Transcription Factor Targets Dataset*, *TFAP2A Gene Set* [3].

	NP 001027451.1	NP 001035890.1	NP 001358995.1	XP 006715238.1	XP 011513135.1	XP 016866721.1
NP 001027451.1	1	0.97912	0.97912	0.99768	0.9199	0.97912
NP 001035890.1		1	0.9746	0.9746	0.9199	0.97691
NP 001358995.1			1	0.96355	0.9199	0.96128
XP 006715238.1				1	0.9199	0.87917
XP 011513135.1					1	0.93684
XP 016866721.1						1

Table A66. The parameter Negative Predictive Value (NPV) of the HELIOS method with referencing the ClustalΩ in the accuracy measurement of classification output, assuming the *ENCODE Transcription Factor Targets Dataset*, *TFAP2A Gene Set* [3].

	NP 001027451.1	NP 001035890.1	NP 001358995.1	XP 006715238.1	XP 011513135.1	XP 016866721.1
NP 001027451.1	1	0.99995	0.99995	1	0.99981	0.99996
NP 001035890.1		1	0.99994	0.99995	0.99981	0.99996
NP 001358995.1			1	0.99992	0.99982	0.99993
XP 006715238.1				1	0.99983	0.99976
XP 011513135.1					1	0.99985
XP 016866721.1						1

Table A67. The parameter Matthew's Coefficient Correlation (MCC) of the HELIOS method with referencing the ClustalΩ in the accuracy measurement of classification output, assuming the *ENCODE Transcription Factor Targets Dataset*, *TFAP2A Gene Set* [3].

	NP 001027451.1	NP 001035890.1	NP 001358995.1	XP 006715238.1	XP 011513135.1	XP 016866721.1
NP 001027451.1	1	0.97907	0.97907	0.99767	0.91971	0.97908
NP 001035890.1		1	0.97454	0.97454	0.91971	0.97686
NP 001358995.1			1	0.96348	0.91971	0.9612
XP 006715238.1				1	0.91973	0.8771
XP 011513135.1					1	0.92819
XP 016866721.1						1

Table A68. The parameter Test's Accuracy (F-Score) of the HELIOS method with referencing the ClustalΩ in the accuracy measurement of classification output, assuming the *ENCODE Transcription Factor Targets Dataset, TFAP2A Gene Set* [3].

	NP 001027451.1	NP 001035890.1	NP 001358995.1	XP 006715238.1	XP 011513135.1	XP 016866721.1
NP 001027451.1	1	0.97912	0.97912	0.99768	0.9199	0.97912
NP 001035890.1		1	0.9746	0.9746	0.9199	0.97691
NP 001358995.1			1	0.96355	0.9199	0.96128
XP 006715238.1				1	0.9199	0.87734
XP 011513135.1					1	0.92829
XP 016866721.1						1

Table A69. The parameter Sensitivity (SEN) of the HELIOS method with referencing the MUSCLE in the accuracy measurement of classification output, assuming the *ENCODE Transcription Factor Targets Dataset, TFAP2A Gene Set* [3].

	NP 001027451.1	NP 001035890.1	NP 001358995.1	XP 006715238.1	XP 011513135.1	XP 016866721.1
NP 001027451.1	1	0.97912	0.97912	0.98144	0.9199	0.97912
NP 001035890.1		1	0.99769	0.9746	0.9199	0.97691
NP 001358995.1			1	0.96355	0.9199	0.96128
XP 006715238.1				1	0.9199	0.87552
XP 011513135.1					1	0.9199
XP 016866721.1						1

Table A70. The parameter Specification (Spec) of the HELIOS method with referencing the MUSCLE in the accuracy measurement of classification output, assuming the *ENCODE Transcription Factor Targets Dataset, TFAP2A Gene Set* [3].

	NP 001027451.1	NP 001035890.1	NP 001358995.1	XP 006715238.1	XP 011513135.1	XP 016866721.1
NP 001027451.1	1	0.99995	0.99995	0.99996	0.99981	0.99996
NP 001035890.1		1	0.99999	0.99995	0.99981	0.99996
NP 001358995.1			1	0.99992	0.99982	0.99993
XP 006715238.1				1	0.99983	0.99977
XP 011513135.1					1	0.99988
XP 016866721.1						1

Table A71. The parameter Accuracy (Acc) of the HELIOS method with referencing the MUSCLE in the accuracy measurement of classification output, assuming the *ENCODE Transcription Factor Targets Dataset, TFAP2A Gene Set* [3].

	NP 001027451.1	NP 001035890.1	NP 001358995.1	XP 006715238.1	XP 011513135.1	XP 016866721.1
NP 001027451.1	1	0.9999	0.9999	0.99992	0.99963	0.99992
NP 001035890.1		1	0.99999	0.99989	0.99963	0.99991
NP 001358995.1			1	0.99985	0.99964	0.99985
XP 006715238.1				1	0.99967	0.99953
XP 011513135.1					1	0.99973
XP 016866721.1						1

Table A72. The parameter Positive Predictive Value (PPV) of the HELIOS method with referencing the MUSCLE in the accuracy measurement of classification output, assuming the *ENCODE Transcription Factor Targets Dataset, TFAP2A Gene Set* [3].

	NP 001027451.1	NP 001035890.1	NP 001358995.1	XP 006715238.1	XP 011513135.1	XP 016866721.1
NP 001027451.1	1	0.97912	0.97912	0.98144	0.9199	0.97912
NP 001035890.1		1	0.99769	0.9746	0.9199	0.97691
NP 001358995.1			1	0.96355	0.9199	0.96128
XP 006715238.1				1	0.9199	0.87917
XP 011513135.1					1	0.93684
XP 016866721.1						1

Table A73. The parameter Negative Predictive Value (NPV) of the HELIOS method with referencing the MUSCLE in the accuracy measurement of classification output, assuming the *ENCODE Transcription Factor Targets Dataset*, *TFAP2A Gene Set* [3].

	NP 001027451.1	NP 001035890.1	NP 001358995.1	XP 006715238.1	XP 011513135.1	XP 016866721.1
NP 001027451.1	1	0.99995	0.99995	0.99996	0.99981	0.99996
NP 001035890.1		1	0.99999	0.99995	0.99981	0.99996
NP 001358995.1			1	0.99992	0.99982	0.99993
XP 006715238.1				1	0.99983	0.99976
XP 011513135.1					1	0.99985
XP 016866721.1						1

Table A74. The parameter Matthew's Coefficient Correlation (MCC) of the HELIOS method with referencing the MUSCLE in the accuracy measurement of classification output, assuming the *ENCODE Transcription Factor Targets Dataset*, *TFAP2A Gene Set* [3].

	NP 001027451.1	NP 001035890.1	NP 001358995.1	XP 006715238.1	XP 011513135.1	XP 016866721.1
NP 001027451.1	1	0.97907	0.97907	0.9814	0.91971	0.97908
NP 001035890.1		1	0.99769	0.97454	0.91971	0.97686
NP 001358995.1			1	0.96348	0.91971	0.9612
XP 006715238.1				1	0.91973	0.8771
XP 011513135.1					1	0.92819
XP 016866721.1						1

Table A75. The parameter Test's Accuracy (F-Score) of the HELIOS method with referencing the MUSCLE in the accuracy measurement of classification output, assuming the *ENCODE Transcription Factor Targets Dataset*, *TFAP2A Gene Set* [3].

	NP 001027451.1	NP 001035890.1	NP 001358995.1	XP 006715238.1	XP 011513135.1	XP 016866721.1
NP 001027451.1	1	0.97912	0.97912	0.98144	0.9199	0.97912
NP 001035890.1		1	0.99769	0.9746	0.9199	0.97691
NP 001358995.1			1	0.96355	0.9199	0.96128
XP 006715238.1				1	0.9199	0.87734
XP 011513135.1					1	0.92829
XP 016866721.1						1

Table A76. The parameter Sensitivity (SEN) of the HELIOS method with referencing the T-Coffee in the accuracy measurement of classification output, assuming the *ENCODE Transcription Factor Targets Dataset*, *TFAP2A Gene Set* [3].

	NP 001027451.1	NP 001035890.1	NP 001358995.1	XP 006715238.1	XP 011513135.1	XP 016866721.1
NP 001027451.1	1	0.99072	0.97912	0.99072	0.93766	0.99072
NP 001035890.1		1	0.99076	0.99076	0.93782	0.99076
NP 001358995.1			1	0.98633	0.93782	0.97494
XP 006715238.1				1	0.93782	0.88981
XP 011513135.1					1	0.93782
XP 016866721.1						1

Table A77. The parameter Specification (Spec) of the HELIOS method with referencing the T-Coffee in the accuracy measurement of classification output, assuming the *ENCODE Transcription Factor Targets Dataset*, *TFAP2A Gene Set* [3].

	NP 001027451.1	NP 001035890.1	NP 001358995.1	XP 006715238.1	XP 011513135.1	XP 016866721.1
NP 001027451.1	1	0.99998	0.99995	0.99998	0.99984	0.99998
NP 001035890.1		1	0.99998	0.99998	0.99985	0.99998
NP 001358995.1			1	0.99997	0.99985	0.99995
XP 006715238.1				1	0.99987	0.99979
XP 011513135.1					1	0.99991
XP 016866721.1						1

Table A78. The parameter Accuracy (Acc) of the HELIOS method with referencing the T-Coffee in the accuracy measurement of classification output, assuming the *ENCODE Transcription Factor Targets Dataset, TFAP2A Gene Set* [3].

	NP 001027451.1	NP 001035890.1	NP 001358995.1	XP 006715238.1	XP 011513135.1	XP 016866721.1
NP 001027451.1	1	0.99996	0.9999	0.99996	0.9997	0.99996
NP 001035890.1		1	0.99996	0.99996	0.99971	0.99996
NP 001358995.1			1	0.99994	0.99971	0.9999
XP 006715238.1				1	0.99974	0.99958
XP 011513135.1					1	0.99979
XP 016866721.1						1

Table A79. The parameter Positive Predictive Value (PPV) of the HELIOS method with referencing the T-Coffee in the accuracy measurement of classification output, assuming the *ENCODE Transcription Factor Targets Dataset, TFAP2A Gene Set* [3].

	NP 001027451.1	NP 001035890.1	NP 001358995.1	XP 006715238.1	XP 011513135.1	XP 016866721.1
NP 001027451.1	1	0.99072	0.97912	0.99072	0.93282	0.99072
NP 001035890.1		1	0.99076	0.99076	0.9354	0.99076
NP 001358995.1			1	0.98633	0.9354	0.97494
XP 006715238.1				1	0.9354	0.89167
XP 011513135.1					1	0.95263
XP 016866721.1						1

Table A80. The parameter Negative Predictive Value (NPV) of the HELIOS method with referencing the T-Coffee in the accuracy measurement of classification output, assuming the *ENCODE Transcription Factor Targets Dataset, TFAP2A Gene Set* [3].

	NP 001027451.1	NP 001035890.1	NP 001358995.1	XP 006715238.1	XP 011513135.1	XP 016866721.1
NP 001027451.1	1	0.99998	0.99995	0.99998	0.99986	0.99998
NP 001035890.1		1	0.99998	0.99998	0.99986	0.99998
NP 001358995.1			1	0.99997	0.99986	0.99995
XP 006715238.1				1	0.99987	0.99979
XP 011513135.1					1	0.99988
XP 016866721.1						1

Table A81. The parameter Matthew's Coefficient Correlation (MCC) of the HELIOS method with referencing the T-Coffee in the accuracy measurement of classification output, assuming the *ENCODE Transcription Factor Targets Dataset, TFAP2A Gene Set* [3].

	NP 001027451.1	NP 001035890.1	NP 001358995.1	XP 006715238.1	XP 011513135.1	XP 016866721.1
NP 001027451.1	1	0.9907	0.97907	0.9907	0.93509	0.9907
NP 001035890.1		1	0.99074	0.99074	0.93646	0.99074
NP 001358995.1			1	0.9863	0.93647	0.97489
XP 006715238.1				1	0.93648	0.89053
XP 011513135.1					1	0.94509
XP 016866721.1						1

Table A82. The parameter Test's Accuracy (F-Score) of the HELIOS method with referencing the T-Coffee in the accuracy measurement of classification output, assuming the *ENCODE Transcription Factor Targets Dataset, TFAP2A Gene Set* [3].

	NP 001027451.1	NP 001035890.1	NP 001358995.1	XP 006715238.1	XP 011513135.1	XP 016866721.1
NP 001027451.1	1	0.99072	0.97912	0.99072	0.93523	0.99072
NP 001035890.1		1	0.99076	0.99076	0.93661	0.99076
NP 001358995.1			1	0.98633	0.93661	0.97494
XP 006715238.1				1	0.93661	0.89074
XP 011513135.1					1	0.94517
XP 016866721.1						1

Table A83. The parameter Sensitivity (SEN) of the HELIOS method with referencing the Kalign in the accuracy measurement of classification output, assuming the *ENCODE Transcription Factor Targets Dataset*, *TFAP2A Gene Set* [3].

	NP 001027451.1	NP 001035890.1	NP 001358995.1	XP 006715238.1	XP 011513135.1	XP 016866721.1
NP 001027451.1	1	0.99061	0.97912	0.99768	0.91969	0.97912
NP 001035890.1		1	0.99769	0.9746	0.91969	0.97691
NP 001358995.1			1	0.96575	0.91969	0.96347
XP 006715238.1				1	0.91969	0.87552
XP 011513135.1					1	0.91969
XP 016866721.1						1

Table A84. The parameter Specification (Spec) of the HELIOS method with referencing the Kalign in the accuracy measurement of classification output, assuming the *ENCODE Transcription Factor Targets Dataset*, *TFAP2A Gene Set* [3].

	NP 001027451.1	NP 001035890.1	NP 001358995.1	XP 006715238.1	XP 011513135.1	XP 016866721.1
NP 001027451.1	1	0.99995	0.99995	1	0.99981	0.99996
NP 001035890.1		1	0.99999	0.99995	0.99981	0.99996
NP 001358995.1			1	0.99992	0.99981	0.99993
XP 006715238.1				1	0.99983	0.99977
XP 011513135.1					1	0.99988
XP 016866721.1						1

Table A85. The parameter Accuracy (Acc) of the HELIOS method with referencing the Kalign in the accuracy measurement of classification output, assuming the *ENCODE Transcription Factor Targets Dataset*, *TFAP2A Gene Set* [3].

	NP 001027451.1	NP 001035890.1	NP 001358995.1	XP 006715238.1	XP 011513135.1	XP 016866721.1
NP 001027451.1	1	0.99993	0.9999	0.99999	0.99962	0.99992
NP 001035890.1		1	0.99999	0.99989	0.99962	0.99991
NP 001358995.1			1	0.99985	0.99963	0.99986
XP 006715238.1				1	0.99966	0.99953
XP 011513135.1					1	0.99972
XP 016866721.1						1

Table A86. The parameter Positive Predictive Value (PPV) of the HELIOS method with referencing the Kalign in the accuracy measurement of classification output, assuming the *ENCODE Transcription Factor Targets Dataset*, *TFAP2A Gene Set* [3].

	NP 001027451.1	NP 001035890.1	NP 001358995.1	XP 006715238.1	XP 011513135.1	XP 016866721.1
NP 001027451.1	1	0.97912	0.97912	0.99768	0.91731	0.97912
NP 001035890.1		1	0.99769	0.9746	0.91731	0.97691
NP 001358995.1			1	0.96355	0.91731	0.96128
XP 006715238.1				1	0.91731	0.87917
XP 011513135.1					1	0.93421
XP 016866721.1						1

Table A87. The parameter Negative Predictive Value (NPV) of the HELIOS method with referencing the Kalign in the accuracy measurement of classification output, assuming the *ENCODE Transcription Factor Targets Dataset*, *TFAP2A Gene Set* [3].

	NP 001027451.1	NP 001035890.1	NP 001358995.1	XP 006715238.1	XP 011513135.1	XP 016866721.1
NP 001027451.1	1	0.99998	0.99995	1	0.99981	0.99996
NP 001035890.1		1	0.99999	0.99995	0.99981	0.99996
NP 001358995.1			1	0.99993	0.99982	0.99993
XP 006715238.1				1	0.99983	0.99976
XP 011513135.1					1	0.99985
XP 016866721.1						1

Table A88. The parameter Matthew's Coefficient Correlation (MCC) of the HELIOS method with referencing the Kalign in the accuracy measurement of classification output, assuming the *ENCODE Transcription Factor Targets Dataset, TFAP2A Gene Set* [3].

	NP 001027451.1	NP 001035890.1	NP 001358995.1	XP 006715238.1	XP 011513135.1	XP 016866721.1
NP 001027451.1	1	0.98481	0.97907	0.99767	0.91831	0.97908
NP 001035890.1		1	0.99769	0.97454	0.91831	0.97686
NP 001358995.1			1	0.96458	0.91831	0.9623
XP 006715238.1				1	0.91833	0.8771
XP 011513135.1					1	0.92678
XP 016866721.1						1

Table A89. The parameter Test's Accuracy (F-Score) of the HELIOS method with referencing the Kalign in the accuracy measurement of classification output, assuming the *ENCODE Transcription Factor Targets Dataset, TFAP2A Gene Set* [3].

	NP 001027451.1	NP 001035890.1	NP 001358995.1	XP 006715238.1	XP 011513135.1	XP 016866721.1
NP 001027451.1	1	0.98483	0.97912	0.99768	0.9185	0.97912
NP 001035890.1		1	0.99769	0.9746	0.9185	0.97691
NP 001358995.1			1	0.96465	0.9185	0.96237
XP 006715238.1				1	0.9185	0.87734
XP 011513135.1					1	0.92689
XP 016866721.1						1

Table A90. The parameter Sensitivity (SEN) of the HELIOS method with referencing the MAFFT in the accuracy measurement of classification output, assuming the *ENCODE Transcription Factor Targets Dataset, TFAP2A Gene Set* [3].

	NP 001027451.1	NP 001035890.1	NP 001358995.1	XP 006715238.1	XP 011513135.1	XP 016866721.1
NP 001027451.1	1	0.99764	0.9814	0.98376	0.92487	0.98144
NP 001035890.1		1	0.99769	0.97691	0.92487	0.97921
NP 001358995.1			1	0.96583	0.92487	0.96355
XP 006715238.1				1	0.92487	0.87759
XP 011513135.1					1	0.92487
XP 016866721.1						1

Table A91. The parameter Specification (Spec) of the HELIOS method with referencing the MAFFT in the accuracy measurement of classification output, assuming the *ENCODE Transcription Factor Targets Dataset, TFAP2A Gene Set* [3].

	NP 001027451.1	NP 001035890.1	NP 001358995.1	XP 006715238.1	XP 011513135.1	XP 016866721.1
NP 001027451.1	1	0.99996	0.99995	0.99997	0.99982	0.99996
NP 001035890.1		1	0.99999	0.99995	0.99982	0.99996
NP 001358995.1			1	0.99993	0.99982	0.99993
XP 006715238.1				1	0.99984	0.99977
XP 011513135.1					1	0.99989
XP 016866721.1						1

Table A92. The parameter Accuracy (Acc) of the HELIOS method with referencing the MAFFT in the accuracy measurement of classification output, assuming the *ENCODE Transcription Factor Targets Dataset, TFAP2A Gene Set* [3].

	NP 001027451.1	NP 001035890.1	NP 001358995.1	XP 006715238.1	XP 011513135.1	XP 016866721.1
NP 001027451.1	1	0.99995	0.99991	0.99993	0.99965	0.99993
NP 001035890.1		1	0.99999	0.9999	0.99965	0.99992
NP 001358995.1			1	0.99986	0.99965	0.99986
XP 006715238.1				1	0.99968	0.99954
XP 011513135.1					1	0.99974
XP 016866721.1						1

Table A93. The parameter Positive Predictive Value (PPV) of the HELIOS method with referencing the MAFFT in the accuracy measurement of classification output, assuming the *ENCODE Transcription Factor Targets Dataset*, *TFAP2A Gene Set* [3].

	NP 001027451.1	NP 001035890.1	NP 001358995.1	XP 006715238.1	XP 011513135.1	XP 016866721.1
NP 001027451.1	1	0.98144	0.97912	0.98376	0.92248	0.98144
NP 001035890.1		1	0.99769	0.97691	0.92248	0.97921
NP 001358995.1			1	0.96583	0.92248	0.96355
XP 006715238.1				1	0.92248	0.88125
XP 011513135.1					1	0.93947
XP 016866721.1						1

Table A94. The parameter Negative Predictive Value (NPV) of the HELIOS method with referencing the MAFFT in the accuracy measurement of classification output, assuming the *ENCODE Transcription Factor Targets Dataset*, *TFAP2A Gene Set* [3].

	NP 001027451.1	NP 001035890.1	NP 001358995.1	XP 006715238.1	XP 011513135.1	XP 016866721.1
NP 001027451.1	1	0.99999	0.99996	0.99997	0.99983	0.99996
NP 001035890.1		1	0.99999	0.99995	0.99983	0.99996
NP 001358995.1			1	0.99993	0.99983	0.99993
XP 006715238.1				1	0.99984	0.99976
XP 011513135.1					1	0.99986
XP 016866721.1						1

Table A95. The parameter Matthew's Coefficient Correlation (MCC) of the HELIOS method with referencing the MAFFT in the accuracy measurement of classification output, assuming the *ENCODE Transcription Factor Targets Dataset*, *TFAP2A Gene Set* [3].

	NP 001027451.1	NP 001035890.1	NP 001358995.1	XP 006715238.1	XP 011513135.1	XP 016866721.1
NP 001027451.1	1	0.98948	0.98021	0.98372	0.9235	0.9814
NP 001035890.1		1	0.99769	0.97686	0.9235	0.97917
NP 001358995.1			1	0.96576	0.9235	0.96348
XP 006715238.1				1	0.92352	0.87919
XP 011513135.1					1	0.93201
XP 016866721.1						1

Table A96. The parameter Test's Accuracy (F-Score) of the HELIOS method with referencing the MAFFT in the accuracy measurement of classification output, assuming the *ENCODE Transcription Factor Targets Dataset*, *TFAP2A Gene Set* [3].

	NP 001027451.1	NP 001035890.1	NP 001358995.1	XP 006715238.1	XP 011513135.1	XP 016866721.1
NP 001027451.1	1	0.98947	0.98026	0.98376	0.92367	0.98144
NP 001035890.1		1	0.99769	0.97691	0.92367	0.97921
NP 001358995.1			1	0.96583	0.92367	0.96355
XP 006715238.1				1	0.92367	0.87942
XP 011513135.1					1	0.93211
XP 016866721.1						1
