# Supplementary material for: HELIOS: High-speed sequence alignment in optics
Source: PLoS Comput Biol. 2022 Nov 21;18(11):e1010665. doi: 10.1371/journal.pcbi.1010665 (PMC9678324; doi:10.1371/journal.pcbi.1010665)
Supplement: S7 Text — (PDF) [file pcbi.1010665.s007.pdf]

HELIOS: High-Speed Sequence Alignment in Optics: S7 Text

Ehsan Maleki[1], Saeedeh Akbari Rokn Abadi[1], and Somayyeh Koohi[1,*]

[1] Department of Computer Engineering, Sharif University of Technology, Azadi Ave., Tehran, Iran.
[*] Corresponding author: koohi@sharif.edu

Compiled October 20, 2022

In response to the imperfections of current sequence alignment methods, originated from the inherent serialism within their corresponding electrical systems, a few optical approaches for biological data comparison have been proposed recently. However, due to their low performance, raised from their inefficient coding scheme, this paper presents a novel all-optical high-throughput method for aligning DNA, RNA, and protein sequences, named HELIOS. The HELIOS method employs highly sophisticated operations to locate character matches, single or multiple mutations, and single or multiple indels within various biological sequences. On the other hand, the HELIOS optical architecture exploits high-speed processing and operational parallelism in optics, by adopting wavelength and polarization of optical beams. For evaluation, the functionality and accuracy of the HELIOS method are approved through behavioral and optical simulation studies, while its complexity and performance are estimated through analytical computation. The accuracy evaluations indicate that the HELIOS method achieves a precise pairwise alignment of two sequences, highly similar to those of Smith-Waterman, Needleman-Wunsch, BLAST, MUSCLE, ClustalW, ClustalΩ, T-Coffee, Kalign, and MAFFT. According to our performance evaluations, the HELIOS optical architecture outperforms all alternative electrical and optical algorithms in terms of processing time and memory requirement, relying on its highly sophisticated method and optical architecture. Moreover, the employed compact coding scheme highly escalates the number of input characters, and hence, it offers reduced time and space complexities, compared to the electrical and optical alternatives. It makes the HELIOS method and optical architecture highly applicable for biomedical applications.

1. ACCURACY EVALUATION

In order to comprehensively assess the accuracy of the HELIOS method, two statistical analyses are performed through simulation studies: 1) Quantitative measurement of homology [1], and 2) Accuracy measurement of classification output [2], compared to the well-known algorithms, briefly reported in Tables A1 and A2, respectively. In this manner, the *ChIP-X Enrichment Analysis Resource, CHEA Transcription Factor Targets Dataset, AP1S2 Gene Set* [3] is assumed in this study, as represented in Table A3.

A. Quantitative measurement of homology

To perform quantitative measurement of homology [1], the parameters Identity, Similarity, and Alignment Score of the HELIOS outputs are calculated through simulation studies, as reported in Tables A4-A6, respectively, assuming the *ChIP-X Enrichment Analysis Resource, CHEA Transcription Factor Targets Dataset, AP1S2 Gene Set* [3]. While the Identity reports the number of exactly matched characters of two sequences (in percentage), the Similarity measures the resemblance of two compared sequences. Specifically, regarding the physicochemical properties, the amino acids are categorized into six groups with different Similarity values; including GAVLI, FYW, STCM, KRH, DENQ, and P. As the third metric, the BLOSUM62 [4] substitution scoring matrix [4] is adopted to calculate the Alignment Score, with gap opening and extension penalties equal to -10 and -0.5, respectively.

For a comparative study, the values of Identity, Similarity, and Alignment Score of the quantitative measurement of homology is performed by various well-known algorithms to be compared to the those of HELIOS method, assuming *ChIP-X Enrichment Analysis Resource, CHEA Transcription Factor Targets Dataset, AP1S2 Gene Set* [3]. It includes Smith-Waterman (SW) [5] reported in Tables A7-A9, Needleman-Wunsch (NW) [6] reported in Tables A10-A12, BLAST [7] reported in Tables A13-A15, ClustalW [8] reported in Tables A16-A18, Clustal-Omega [9] reported in Tables A19-A21, MUSCLE [9] reported in Tables A22-A24, T-Coffee [10] reported in Tables A25-A27, Kalign [11] reported in Tables A28-A30, and MAFFT [12] reported in Tables A31-A33.

Table A1. A brief report of the quantitative measurement of homology of the HELIOS method, compared to nine well-known algorithms, including SW, NW, BLAST, ClustalW, Clustal Omega, Muscle, T-Coffee, Kalign, and MAFFT. The parameters Identity, Similarity, and Alignment score are averaged and reported. The dataset used in this study is the *ChIP-X Enrichment Analysis Resource, CHEA Transcription Factor Targets Dataset, AP1S2 Gene Set* [3].

	HELIOS	SW	NW	BLAST	MUSCLE	ClustalW	ClustalΩ	T-Coffee	Kalign	MAFFT
Identity	96.778	96.566	96.438	97.019	96.396	96.484	96.353	96.619	96.439	96.916
Similarity	97.305	96.958	96.608	97.368	97.181	97.134	96.869	96.883	96.913	97.401
Alignment Score	750.5	748.6	759.9	747.9	746.7	744.0	745.7	746.2	746.2	747.7

Table A2. A brief report of the accuracy measurement of classification output of the HELIOS method with referencing well-known algorithms, including SW, NW, BLAST, ClustalW, Clustal Omega, Muscle, T-Coffee, Kalign, and MAFFT. The parameters SEN, Spec, Acc, PPV, NPV, MCC, and F-Score are averaged and reported. The dataset used in this study is the *ChIP-X Enrichment Analysis Resource, CHEA Transcription Factor Targets Dataset, AP1S2 Gene Set* [3].

	SW	NW	BLAST	MUSCLE	ClustalW	ClustalΩ	T-Coffee	Kalign	MAFFT
SEN	0.98375	0.99298	0.95240	0.97137	0.97238	0.97815	0.96569	0.97769	0.98137
Spec	0.99991	0.99997	0.99984	0.99982	0.99982	0.99985	0.99992	0.99985	0.99988
ACC	0.99980	0.99993	0.99954	0.99964	0.99965	0.99972	0.99971	0.99971	0.99976
PPV	0.98489	0.99603	0.97368	0.97082	0.97176	0.97671	0.98719	0.97626	0.98137
NPV	0.99990	0.99995	0.99970	0.99982	0.99983	0.99986	0.99978	0.99986	0.99988
MCC	0.98422	0.99446	0.96267	0.97091	0.97189	0.97728	0.97615	0.97682	0.98125
F-Score	0.98432	0.99448	0.96275	0.97108	0.97206	0.97741	0.97615	0.97695	0.98137

B. Accuracy measurement of classification output

Afterward, the accuracy measurement of the classification output [2] of the HELIOS method is addressed by calculating the values of Sensitivity (SEN), Specificity (Spec), Accuracy (ACC), Positive Predictive Value (PPV), Negative Predictive Value (NPV), Matthew's Coefficient Correlation (MCC), and Test's Accuracy (F-Score) in the simulation studies, according to Eq 5 to Eq 11, respectively.

As a comparative study, the accuracy measurement of the classification output of the HELIOS method is accomplished, assuming *ChIP-X Enrichment Analysis Resource, CHEA Transcription Factor Targets Dataset, AP1S2 Gene Set* [3], and the corresponding metrics are calculated with considering Smith-Waterman [5] reported in Tables A34-A40, Needleman-Wunsch [6] reported in Tables A41-A47, ClustalW [8] reported in Tables A55-A61, Clustal-Omega [9] reported in Tables A62-A68, BLAST [7] reported in Tables A48-A54, MUSCLE [13] reported in Tables A69-A75, T-Coffee [10] reported in Tables A76-A82, Kalign [11] reported in Tables A83-A89, and MAFFT [12] reported in Tables A90-A96.

REFERENCES

1. D. S. Moss, S. Jelaska, and S. Pongor, *Essays in bioinformatics*, vol. 368 (IOS Press, 2005).
2. M. Hamada, H. Kiryu, W. Iwasaki, and K. Asai, "Generalized centroid estimators in bioinformatics," PloS one **6**, e16450 (2011).
3. A. Lachmann, H. Xu, J. Krishnan, S. I. Berger, A. R. Mazloom, and A. Ma'ayan, "Chea: transcription factor regulation inferred from integrating genome-wide chip-x experiments," Bioinformatics **26**, 2438–2444 (2010).
4. D. W. Mount, "Using blosum in sequence alignments," Cold Spring Harb. Protoc. **2008**, pdb.top39 (2008).
5. H. Zou, S. Tang, C. Yu, H. Fu, Y. Li, and W. Tang, "asw: accelerating smith–waterman algorithm on coupled cpu–gpu architecture," Int. J. Parallel Program. **47**, 388–402 (2019).
6. Y. Jararweh, M. Al-Ayyoub, M. Fakirah, L. Alawneh, and B. B. Gupta, "Improving the performance of the needleman-wunsch algorithm using parallelization and vectorization techniques," Multimed. Tools Appl. **78**, 3961–3977 (2019).
7. G. M. Boratyn, J. Thierry-Mieg, D. Thierry-Mieg, B. Busby, and T. L. Madden, "Magic-blast, an accurate rna-seq aligner for long and short reads," BMC bioinformatics **20**, 1–19 (2019).
8. D. Díaz, F. J. Esteban, P. Hernández, J. A. Caballero, A. Guevara, G. Dorado, and S. Gálvez, "Mc64-clustalwp2: A highly-parallel hybrid strategy to align multiple sequences in many-core architectures," PLOS ONE **9**, 1–12 (2014).
9. F. Sievers and D. G. Higgins, "Clustal omega for making accurate alignments of many protein sequences," Protein Sci. **27**, 135–145 (2018).
10. C. Notredame, D. G. Higgins, and J. Heringa, "T-coffee: a novel method for fast and accurate multiple sequence alignment11edited by j. thornton," J. Mol. Biol. **302**, 205–217 (2000).
11. T. Lassmann, "Kalign 3: multiple sequence alignment of large datasets," (2020).
12. J. Rozewicki, S. Li, K. M. Amada, D. M. Standley, and K. Katoh, "Mafft-dash: integrated protein sequence and structural alignment," Nucleic acids research **47**, W5–W10 (2019).
13. R. C. Edgar, "MUSCLE: multiple sequence alignment with high accuracy and high throughput," Nucleic Acids Res. **32**, 1792–1797 (2004).

Table A3. The list of input sequences, assuming the *ChIP-X Enrichment Analysis Resource, CHEA Transcription Factor Targets Dataset, AP1S2 Gene Set* [3].

Name	Sequence
NP 001259000.1	MQFML LFSRQ GKLRL QKWYV PLSDK EKKKI TRELV QTVLA RKPKM CSFLE WRDLK IVYKR YASLY FCCAI EDQDN ELITL EIIHR YVELL DKYFG SVCEL DIIFN FEKAY FILDE FLLGG EVQET SKKNV LKAIE QADLL QEDAK EAETP RSVLE EIGLT
NP 003907.3	MQFML LFSRQ GKLRL QKWYV PLSDK EKKKI TRELV QTVLA RKPKM CSFLE WRDLK IVYKR YASLY FCCAI EDQDN ELITL EIIHR YVELL DKYFG SVCEL DIIFN FEKAY FILDE FLLGG EVQET SKKNV LKAIE QADLL QEEAE TPRSV LEEIG LT
NP 001355923.1	MQFML LFSRQ GKLRL QKWYV PLSDK EKKKI TRELV QTVLA RKPKM CSFLE WRDLK IVYKR YASLY FCCAI EDQDN ELITL EIIHR YVELL DKYFG SVCEL DIIFN FEKAY FILDE FLLGG EVQET SKKNV LKAIE QADLL QEPRH EYFNV PVY
NP 001355936.1	MQFML LFSRQ GKLRL QKWYV PLSDK EKKKI TRELV QTVLA RKPKM CSFLE WRDLK IVYKR YASLY FCCAI EDQDN ELITL EIIHR YVELL DKYFG SVCEL DIIFN FEKAY FILDE FLLGG EVQET SKKNV LKAIE QADLL QEKTE TMYHS KSFIG FKKAY
NP 001355937.1	MQFML LFSRQ GKLRL QKWYV PLSDK EKKKI TRELV QTVLA RKPKM CSFLE WRDLK IVYKR YASLY FCCAI EDQDN ELITL EIIHR YVELL DKYFG SVCEL DIIFN FEKAY FILDE FLLGG EVQET SKKNV LKAIE QADLL QESFF SS

Table A4. The parameter Identity of the HELIOS method in the quantitative measurement of homology, assuming the *ChIP-X Enrichment Analysis Resource, CHEA Transcription Factor Targets Dataset, AP1S2 Gene Set* [3].

	NP 001259000.1	NP 003907.3	NP 001355923.1	NP 001355936.1	NP 001355937.1
NP 001259000.1	100	100	94.1176	91.25	96.5986
NP 003907.3		100	94.1176	92.9936	96.5986
NP 001355923.1			100	92.8105	96.5986
NP 001355936.1				100	96.5986
NP 001355937.1					100

Table A5. The parameter Similarity of the HELIOS method in the quantitative measurement of homology, assuming the *ChIP-X Enrichment Analysis Resource, CHEA Transcription Factor Targets Dataset, AP1S2 Gene Set* [3].

	NP 001259000.1	NP 003907.3	NP 001355923.1	NP 001355936.1	NP 001355937.1
NP 001259000.1	100	100	96.0784	91.875	96.5986
NP 003907.3		100	96.0784	92.9936	97.2789
NP 001355923.1			100	94.1176	96.5986
NP 001355936.1				100	97.9592
NP 001355937.1					100

Table A6. The parameter Alignment Score of the HELIOS method in the quantitative measurement of homology, assuming the *ChIP-X Enrichment Analysis Resource, CHEA Transcription Factor Targets Dataset, AP1S2 Gene Set* [3].

	NP 001259000.1	NP 003907.3	NP 001355923.1	NP 001355936.1	NP 001355937.1
NP 001259000.1	819	793	724	716	712
NP 003907.3		804	725.5	729	712.5
NP 001355923.1			798	717	712.5
NP 001355936.1				828	711
NP 001355937.1					756

Table A7. The parameter Identity of the Smith-Waterman algorithm in the quantitative measurement of homology, assuming the *ChIP-X Enrichment Analysis Resource, CHEA Transcription Factor Targets Dataset, AP1S2 Gene Set* [3].

	NP 001259000.1	NP 003907.3	NP 001355923.1	NP 001355936.1	NP 001355937.1
NP 001259000.1	100	100	93.4641	89.375	96.5986
NP 003907.3		100	93.4641	92.9936	96.5986
NP 001355923.1			100	92.8105	96.5986
NP 001355936.1				100	96.5986
NP 001355937.1					100

Table A8. The parameter Similarity of the Smith-Waterman algorithm in the quantitative measurement of homology, assuming the *ChIP-X Enrichment Analysis Resource, CHEA Transcription Factor Targets Dataset, AP1S2 Gene Set* [3].

	NP 001259000.1	NP 003907.3	NP 001355923.1	NP 001355936.1	NP 001355937.1
NP 001259000.1	100	100	94.1176	91.25	96.5986
NP 003907.3		100	93.4641	92.9936	97.2789
NP 001355923.1			100	94.1176	96.5986
NP 001355936.1				100	97.9592
NP 001355937.1					100

Table A9. The parameter Alignment Score of the Smith-Waterman algorithm in the quantitative measurement of homology, assuming the *ChIP-X Enrichment Analysis Resource, CHEA Transcription Factor Targets Dataset, AP1S2 Gene Set* [3].

	NP 001259000.1	NP 003907.3	NP 001355923.1	NP 001355936.1	NP 001355937.1
NP 001259000.1	819	793	709	719	712
NP 003907.3		804	709.5	729	712.5
NP 001355923.1			798	717	712.5
NP 001355936.1				828	711
NP 001355937.1					756

Table A10. The parameter Identity of the Needleman-Wunsch algorithm in the quantitative measurement of homology, assuming the *ChIP-X Enrichment Analysis Resource, CHEA Transcription Factor Targets Dataset, AP1S2 Gene Set* [3].

	NP 001259000.1	NP 003907.3	NP 001355923.1	NP 001355936.1	NP 001355937.1
NP 001259000.1	100	100	93.4641	89.375	96.5986
NP 003907.3		100	92.8105	91.7197	96.5986
NP 001355923.1			100	92.8105	96.5986
NP 001355936.1				100	96.5986
NP 001355937.1					100

Table A11. The parameter Similarity of the Needleman-Wunsch algorithm in the quantitative measurement of homology, assuming the *ChIP-X Enrichment Analysis Resource, CHEA Transcription Factor Targets Dataset, AP1S2 Gene Set* [3].

	NP 001259000.1	NP 003907.3	NP 001355923.1	NP 001355936.1	NP 001355937.1
NP 001259000.1	100	100	94.1176	90.625	96.5986
NP 003907.3		100	92.8105	91.7197	96.5986
NP 001355923.1			100	93.4641	96.5986
NP 001355936.1				100	96.5986
NP 001355937.1					100

Table A12. The parameter Alignment Score of the Needleman-Wunsch algorithm in the quantitative measurement of homology, assuming the *ChIP-X Enrichment Analysis Resource, CHEA Transcription Factor Targets Dataset, AP1S2 Gene Set* [3].

	NP 001259000.1	NP 003907.3	NP 001355923.1	NP 001355936.1	NP 001355937.1
NP 001259000.1	819	793	734	732	732
NP 003907.3		804	732	743	732
NP 001355923.1			798	732	732
NP 001355936.1				828	732
NP 001355937.1					756

Table A13. The parameter Identity of the BLAST in the quantitative measurement of homology, assuming the *ChIP-X Enrichment Analysis Resource, CHEA Transcription Factor Targets Dataset, AP1S2 Gene Set* [3].

	NP 001259000.1	NP 003907.3	NP 001355923.1	NP 001355936.1	NP 001355937.1
NP 001259000.1	100	91.7197	95.4248	88.75	106.8027
NP 003907.3		97.4522	93.4641	91.0828	97.9592
NP 001355923.1			104.5752	93.4641	99.3197
NP 001355936.1				91.875	96.5986
NP 001355937.1					106.8027

Table A14. The parameter Similarity of the BLAST in the quantitative measurement of homology, assuming the *ChIP-X Enrichment Analysis Resource, CHEA Transcription Factor Targets Dataset, AP1S2 Gene Set* [3].

	NP 001259000.1	NP 003907.3	NP 001355923.1	NP 001355936.1	NP 001355937.1
NP 001259000.1	100	93.6306	95.4248	88.75	106.8027
NP 003907.3		97.4522	94.1176	91.7197	100
NP 001355923.1			104.5752	93.4641	99.3197
NP 001355936.1				91.875	96.5986
NP 001355937.1					106.8027

Table A15. The parameter Alignment Score of the BLAST in the quantitative measurement of homology, assuming the *ChIP-X Enrichment Analysis Resource, CHEA Transcription Factor Targets Dataset, AP1S2 Gene Set* [3].

	NP 001259000.1	NP 003907.3	NP 001355923.1	NP 001355936.1	NP 001355937.1
NP 001259000.1	819	724	718	699.5	793
NP 003907.3		798	704.5	718	725.5
NP 001355923.1			828	701.5	729
NP 001355936.1				756	701
NP 001355937.1					804

Table A16. The parameter Identity of the ClustalW in the quantitative measurement of homology, assuming the *ChIP-X Enrichment Analysis Resource, CHEA Transcription Factor Targets Dataset, AP1S2 Gene Set* [3].

	NP 001259000.1	NP 003907.3	NP 001355923.1	NP 001355936.1	NP 001355937.1
NP 001259000.1	100	100	92.8105	89.375	96.5986
NP 003907.3		100	92.8105	91.0828	96.5986
NP 001355923.1			100	94.1176	96.5986
NP 001355936.1				100	97.2789
NP 001355937.1					100

Table A17. The parameter Similarity of the ClustalW in the quantitative measurement of homology, assuming the *ChIP-X Enrichment Analysis Resource, CHEA Transcription Factor Targets Dataset, AP1S2 Gene Set* [3].

	NP 001259000.1	NP 003907.3	NP 001355923.1	NP 001355936.1	NP 001355937.1
NP 001259000.1	100	100	94.1176	91.25	97.2789
NP 003907.3		100	94.1176	92.9936	97.2789
NP 001355923.1			100	95.4248	96.5986
NP 001355936.1				100	97.9592
NP 001355937.1					100

Table A18. The parameter Alignment Score of the ClustalW in the quantitative measurement of homology, assuming the *ChIP-X Enrichment Analysis Resource, CHEA Transcription Factor Targets Dataset, AP1S2 Gene Set* [3].

	NP 001259000.1	NP 003907.3	NP 001355923.1	NP 001355936.1	NP 001355937.1
NP 001259000.1	819	793	699.5	719	701.5
NP 003907.3		804	701	708	702
NP 001355923.1			798	713.5	705
NP 001355936.1				828	712.5
NP 001355937.1					756

Table A19. The parameter Identity of the ClustalΩ in the quantitative measurement of homology, assuming the *ChIP-X Enrichment Analysis Resource, CHEA Transcription Factor Targets Dataset, AP1S2 Gene Set* [3].

	NP 001259000.1	NP 003907.3	NP 001355923.1	NP 001355936.1	NP 001355937.1
NP 001259000.1	100	99.3631	93.4641	89.375	96.5986
NP 003907.3		100	92.8105	91.0828	96.5986
NP 001355923.1			100	92.8105	96.5986
NP 001355936.1				100	96.5986
NP 001355937.1					100

Table A20. The parameter Similarity of the ClustalΩ in the quantitative measurement of homology, assuming the *ChIP-X Enrichment Analysis Resource, CHEA Transcription Factor Targets Dataset, AP1S2 Gene Set* [3].

	NP 001259000.1	NP 003907.3	NP 001355923.1	NP 001355936.1	NP 001355937.1
NP 001259000.1	100	100	94.1176	91.25	96.5986
NP 003907.3		100	92.8105	92.9936	96.5986
NP 001355923.1			100	94.1176	96.5986
NP 001355936.1				100	97.9592
NP 001355937.1					100

Table A21. The parameter Alignment Score of the ClustalΩ in the quantitative measurement of homology, assuming the *ChIP-X Enrichment Analysis Resource, CHEA Transcription Factor Targets Dataset, AP1S2 Gene Set* [3].

	NP 001259000.1	NP 003907.3	NP 001355923.1	NP 001355936.1	NP 001355937.1
NP 001259000.1	819	790	709	719	712
NP 003907.3		804	695	710	706
NP 001355923.1			798	717	712.5
NP 001355936.1				828	711
NP 001355937.1					756

Table A22. The parameter Identity of the MUSCLE in the quantitative measurement of homology, assuming the *ChIP-X Enrichment Analysis Resource, CHEA Transcription Factor Targets Dataset, AP1S2 Gene Set* [3].

	NP 001259000.1	NP 003907.3	NP 001355923.1	NP 001355936.1	NP 001355937.1
NP 001259000.1	100	99.3631	93.4641	89.375	95.9184
NP 003907.3		100	92.8105	91.0828	96.5986
NP 001355923.1			100	93.4641	96.5986
NP 001355936.1				100	97.2789
NP 001355937.1					100

Table A23. The parameter Similarity of the MUSCLE in the quantitative measurement of homology, assuming the *ChIP-X Enrichment Analysis Resource, CHEA Transcription Factor Targets Dataset, AP1S2 Gene Set* [3].

	NP 001259000.1	NP 003907.3	NP 001355923.1	NP 001355936.1	NP 001355937.1
NP 001259000.1	100	99.3631	95.4248	91.25	96.5986
NP 003907.3		100	94.1176	92.9936	97.2789
NP 001355923.1			100	94.7712	97.2789
NP 001355936.1				100	98.6395
NP 001355937.1					100

Table A24. The parameter Alignment Score of the MUSCLE in the quantitative measurement of homology, assuming the *ChIP-X Enrichment Analysis Resource, CHEA Transcription Factor Targets Dataset, AP1S2 Gene Set* [3].

	NP 001259000.1	NP 003907.3	NP 001355923.1	NP 001355936.1	NP 001355937.1
NP 001259000.1	819	789	716	719	697.5
NP 003907.3		804	703	708	712.5
NP 001355923.1			798	723	714
NP 001355936.1				828	713.5
NP 001355937.1					756

Table A25. The parameter Identity of the T-Coffee in the quantitative measurement of homology, assuming the *ChIP-X Enrichment Analysis Resource, CHEA Transcription Factor Targets Dataset, AP1S2 Gene Set* [3].

	NP 001259000.1	NP 003907.3	NP 001355923.1	NP 001355936.1	NP 001355937.1
NP 001259000.1	100	91.7197	94.1176	88.75	104.0816
NP 003907.3		97.4522	93.4641	90.4459	97.2789
NP 001355923.1			104.5752	92.8105	99.3197
NP 001355936.1				91.875	96.5986
NP 001355937.1					106.8027

Table A26. The parameter Similarity of the T-Coffee in the quantitative measurement of homology, assuming the *ChIP-X Enrichment Analysis Resource, CHEA Transcription Factor Targets Dataset, AP1S2 Gene Set* [3].

	NP 001259000.1	NP 003907.3	NP 001355923.1	NP 001355936.1	NP 001355937.1
NP 001259000.1	100	92.3567	94.1176	88.75	104.7619
NP 003907.3		97.4522	94.1176	90.4459	97.2789
NP 001355923.1			104.5752	94.1176	99.3197
NP 001355936.1				91.875	97.2789
NP 001355937.1					106.8027

Table A27. The parameter Alignment Score of the T-Coffee in the quantitative measurement of homology, assuming the *ChIP-X Enrichment Analysis Resource, CHEA Transcription Factor Targets Dataset, AP1S2 Gene Set* [3].

	NP 001259000.1	NP 003907.3	NP 001355923.1	NP 001355936.1	NP 001355937.1
NP 001259000.1	819	704.5	706	712	772
NP 003907.3		798	721	712.5	709.5
NP 001355923.1			828	711	728
NP 001355936.1				756	712.5
NP 001355937.1					804

Table A28. The parameter Identity of the Kalign in the quantitative measurement of homology, assuming the *ChIP-X Enrichment Analysis Resource, CHEA Transcription Factor Targets Dataset*, *AP1S2 Gene Set* [3].

	NP 001259000.1	NP 003907.3	NP 001355923.1	NP 001355936.1	NP 001355937.1
NP 001259000.1	100	100	93.4641	89.375	96.5986
NP 003907.3		100	93.4641	91.0828	96.5986
NP 001355923.1			100	92.8105	96.5986
NP 001355936.1				100	96.5986
NP 001355937.1					100

Table A29. The parameter Similarity of the Kalign in the quantitative measurement of homology, assuming the *ChIP-X Enrichment Analysis Resource, CHEA Transcription Factor Targets Dataset*, *AP1S2 Gene Set* [3].

	NP 001259000.1	NP 003907.3	NP 001355923.1	NP 001355936.1	NP 001355937.1
NP 001259000.1	100	100	94.1176	91.25	96.5986
NP 003907.3		100	93.4641	92.9936	96.5986
NP 001355923.1			100	94.1176	96.5986
NP 001355936.1				100	97.9592
NP 001355937.1					100

Table A30. The parameter Alignment Score of the Kalign in the quantitative measurement of homology, assuming the *ChIP-X Enrichment Analysis Resource, CHEA Transcription Factor Targets Dataset*, *AP1S2 Gene Set* [3].

	NP 001259000.1	NP 003907.3	NP 001355923.1	NP 001355936.1	NP 001355937.1
NP 001259000.1	819	793	709	719	712
NP 003907.3		804	701	708	706
NP 001355923.1			798	717	712.5
NP 001355936.1				828	711
NP 001355937.1					756

Table A31. The parameter Identity of the MAFFT in the quantitative measurement of homology, assuming the *ChIP-X Enrichment Analysis Resource, CHEA Transcription Factor Targets Dataset*, *AP1S2 Gene Set* [3].

	NP 001259000.1	NP 003907.3	NP 001355923.1	NP 001355936.1	NP 001355937.1
NP 001259000.1	100	100	93.4641	91.25	97.2789
NP 003907.3		100	93.4641	92.9936	97.2789
NP 001355923.1			100	93.4641	97.2789
NP 001355936.1				100	97.2789
NP 001355937.1					100

Table A32. The parameter Similarity of the MAFFT in the quantitative measurement of homology, assuming the *ChIP-X Enrichment Analysis Resource, CHEA Transcription Factor Targets Dataset*, *AP1S2 Gene Set* [3].

	NP 001259000.1	NP 003907.3	NP 001355923.1	NP 001355936.1	NP 001355937.1
NP 001259000.1	100	100	94.1176	91.875	97.9592
NP 003907.3		100	94.1176	93.6306	97.9592
NP 001355923.1			100	94.7712	97.9592
NP 001355936.1				100	98.6395
NP 001355937.1					100

Table A33. The parameter Alignment Score of the MAFFT in the quantitative measurement of homology, assuming the *ChIP-X Enrichment Analysis Resource, CHEA Transcription Factor Targets Dataset, AP1S2 Gene Set* [3].

	NP 001259000.1	NP 003907.3	NP 001355923.1	NP 001355936.1	NP 001355937.1
NP 001259000.1	819	793	701.5	716	702.5
NP 003907.3		804	712.5	727	704
NP 001355923.1			798	723	718
NP 001355936.1				828	713.5
NP 001355937.1					756

Table A34. The parameter Sensitivity (SEN) of the HELIOS method with referencing the Smith-Waterman algorithm in the accuracy measurement of classification output, assuming the *ChIP-X Enrichment Analysis Resource, CHEA Transcription Factor Targets Dataset, AP1S2 Gene Set* [3].

	NP 001259000.1	NP 003907.3	NP 001355923.1	NP 001355936.1	NP 001355937.1
NP 001259000.1	1	1	0.9281	0.9	1
NP 003907.3		1	0.9281	1	1
NP 001355923.1			1	1	1
NP 001355936.1				1	1
NP 001355937.1					1

Table A35. The parameter Specification (Spec) of the HELIOS method with referencing the Smith-Waterman algorithm in the accuracy measurement of classification output, assuming the *ChIP-X Enrichment Analysis Resource, CHEA Transcription Factor Targets Dataset, AP1S2 Gene Set* [3].

	NP 001259000.1	NP 003907.3	NP 001355923.1	NP 001355936.1	NP 001355937.1
NP 001259000.1	1	1	0.99955	0.99949	1
NP 003907.3		1	0.99954	1	1
NP 001355923.1			1	1	1
NP 001355936.1				1	1
NP 001355937.1					1

Table A36. The parameter Accuracy (Acc) of the HELIOS method with referencing the Smith-Waterman algorithm in the accuracy measurement of classification output, assuming the *ChIP-X Enrichment Analysis Resource, CHEA Transcription Factor Targets Dataset, AP1S2 Gene Set* [3].

	NP 001259000.1	NP 003907.3	NP 001355923.1	NP 001355936.1	NP 001355937.1
NP 001259000.1	1	1	0.9991	0.99887	1
NP 003907.3		1	0.99908	1	1
NP 001355923.1			1	1	1
NP 001355936.1				1	1
NP 001355937.1					1

Table A37. The parameter Positive Predictive Value (PPV) of the HELIOS method with referencing the Smith-Waterman algorithm in the accuracy measurement of classification output, assuming the *ChIP-X Enrichment Analysis Resource, CHEA Transcription Factor Targets Dataset, AP1S2 Gene Set* [3].

	NP 001259000.1	NP 003907.3	NP 001355923.1	NP 001355936.1	NP 001355937.1
NP 001259000.1	1	1	0.9281	0.9172	1
NP 003907.3		1	0.9281	1	1
NP 001355923.1			1	1	1
NP 001355936.1				1	1
NP 001355937.1					1

Table A38. The parameter Negative Predictive Value (NPV) of the HELIOS method with referencing the Smith-Waterman algorithm in the accuracy measurement of classification output, assuming the *ChIP-X Enrichment Analysis Resource, CHEA Transcription Factor Targets Dataset, AP1S2 Gene Set* [3].

	NP 001259000.1	NP 003907.3	NP 001355923.1	NP 001355936.1	NP 001355937.1
NP 001259000.1	1	1	0.99955	0.99937	1
NP 003907.3		1	0.99954	1	1
NP 001355923.1			1	1	1
NP 001355936.1				1	1
NP 001355937.1					1

Table A39. The parameter Matthew's Coefficient Correlation (MCC) of the HELIOS method with referencing the Smith-Waterman algorithm in the accuracy measurement of classification output, assuming the *ChIP-X Enrichment Analysis Resource, CHEA Transcription Factor Targets Dataset, AP1S2 Gene Set* [3].

	NP 001259000.1	NP 003907.3	NP 001355923.1	NP 001355936.1	NP 001355937.1
NP 001259000.1	1	1	0.92765	0.90799	1
NP 003907.3		1	0.92764	1	1
NP 001355923.1			1	1	1
NP 001355936.1				1	1
NP 001355937.1					1

Table A40. The parameter Test's Accuracy (F-Score) of the HELIOS method with referencing the Smith-Waterman algorithm in the accuracy measurement of classification output, assuming the *ChIP-X Enrichment Analysis Resource, CHEA Transcription Factor Targets Dataset, AP1S2 Gene Set* [3].

	NP 001259000.1	NP 003907.3	NP 001355923.1	NP 001355936.1	NP 001355937.1
NP 001259000.1	1	1	0.9281	0.90852	1
NP 003907.3		1	0.9281	1	1
NP 001355923.1			1	1	1
NP 001355936.1				1	1
NP 001355937.1					1

Table A41. The parameter Sensitivity (SEN) of the HELIOS method with referencing the Needleman-Wunsch algorithm in the accuracy measurement of classification output, assuming the *ChIP-X Enrichment Analysis Resource, CHEA Transcription Factor Targets Dataset, AP1S2 Gene Set* [3].

	NP 001259000.1	NP 003907.3	NP 001355923.1	NP 001355936.1	NP 001355937.1
NP 001259000.1	1	1	0.9726	0.92208	1
NP 003907.3		1	1	1	1
NP 001355923.1			1	1	1
NP 001355936.1				1	1
NP 001355937.1					1

Table A42. The parameter Specification (Spec) of the HELIOS method with referencing the Needleman-Wunsch algorithm in the accuracy measurement of classification output, assuming the *ChIP-X Enrichment Analysis Resource, CHEA Transcription Factor Targets Dataset, AP1S2 Gene Set* [3].

	NP 001259000.1	NP 003907.3	NP 001355923.1	NP 001355936.1	NP 001355937.1
NP 001259000.1	1	1	1	0.99962	1
NP 003907.3		1	1	1	1
NP 001355923.1			1	1	1
NP 001355936.1				1	1
NP 001355937.1					1

Table A43. The parameter Accuracy (Acc) of the HELIOS method with referencing the Needleman-Wunsch algorithm in the accuracy measurement of classification output, assuming the *ChIP-X Enrichment Analysis Resource, CHEA Transcription Factor Targets Dataset, AP1S2 Gene Set* [3].

	NP 001259000.1	NP 003907.3	NP 001355923.1	NP 001355936.1	NP 001355937.1
NP 001259000.1	1	1	0.99981	0.99911	1
NP 003907.3		1	1	1	1
NP 001355923.1			1	1	1
NP 001355936.1				1	1
NP 001355937.1					1

Table A44. The parameter Positive Predictive Value (PPV) of the HELIOS method with referencing the Needleman-Wunsch algorithm in the accuracy measurement of classification output, assuming the *ChIP-X Enrichment Analysis Resource, CHEA Transcription Factor Targets Dataset, AP1S2 Gene Set* [3].

	NP 001259000.1	NP 003907.3	NP 001355923.1	NP 001355936.1	NP 001355937.1
NP 001259000.1	1	1	1	0.9404	1
NP 003907.3		1	1	1	1
NP 001355923.1			1	1	1
NP 001355936.1				1	1
NP 001355937.1					1

Table A45. The parameter Negative Predictive Value (NPV) of the HELIOS method with referencing the Needleman-Wunsch algorithm in the accuracy measurement of classification output, assuming the *ChIP-X Enrichment Analysis Resource, CHEA Transcription Factor Targets Dataset, AP1S2 Gene Set* [3].

	NP 001259000.1	NP 003907.3	NP 001355923.1	NP 001355936.1	NP 001355937.1
NP 001259000.1	1	1	0.99981	0.99949	1
NP 003907.3		1	1	1	1
NP 001355923.1			1	1	1
NP 001355936.1				1	1
NP 001355937.1					1

Table A46. The parameter Matthew's Coefficient Correlation (MCC) of the HELIOS method with referencing the Needleman-Wunsch algorithm in the accuracy measurement of classification output, assuming the *ChIP-X Enrichment Analysis Resource, CHEA Transcription Factor Targets Dataset, AP1S2 Gene Set* [3].

	NP 001259000.1	NP 003907.3	NP 001355923.1	NP 001355936.1	NP 001355937.1
NP 001259000.1	1	1	0.98611	0.93075	1
NP 003907.3		1	1	1	1
NP 001355923.1			1	1	1
NP 001355936.1				1	1
NP 001355937.1					1

Table A47. The parameter Test's Accuracy (F-Score) of the HELIOS method with referencing the Needleman-Wunsch algorithm in the accuracy measurement of classification output, assuming the *ChIP-X Enrichment Analysis Resource, CHEA Transcription Factor Targets Dataset, AP1S2 Gene Set* [3].

	NP 001259000.1	NP 003907.3	NP 001355923.1	NP 001355936.1	NP 001355937.1
NP 001259000.1	1	1	0.98611	0.93115	1
NP 003907.3		1	1	1	1
NP 001355923.1			1	1	1
NP 001355936.1				1	1
NP 001355937.1					1

Table A48. The parameter Sensitivity (SEN) of the HELIOS method with referencing the BLAST in the accuracy measurement of classification output, assuming the *ChIP-X Enrichment Analysis Resource, CHEA Transcription Factor Targets Dataset, AP1S2 Gene Set* [3].

	NP 001259000.1	NP 003907.3	NP 001355923.1	NP 001355936.1	NP 001355937.1
NP 001259000.1	1	0.9281	0.90446	0.96599	0.90446
NP 003907.3		1	0.9281	0.96599	0.9281
NP 001355923.1			0.95625	0.96599	0.93631
NP 001355936.1				1	0.96599
NP 001355937.1					0.93631

Table A49. The parameter Specification (Spec) of the HELIOS method with referencing the BLAST in the accuracy measurement of classification output, assuming the *ChIP-X Enrichment Analysis Resource, CHEA Transcription Factor Targets Dataset, AP1S2 Gene Set* [3].

	NP 001259000.1	NP 003907.3	NP 001355923.1	NP 001355936.1	NP 001355937.1
NP 001259000.1	1	0.99955	0.99957	0.99979	0.9998
NP 003907.3		1	0.99971	0.99978	0.99979
NP 001355923.1			1	0.99979	1
NP 001355936.1				1	0.99978
NP 001355937.1					1

Table A50. The parameter Accuracy (Acc) of the HELIOS method with referencing the BLAST in the accuracy measurement of classification output, assuming the *ChIP-X Enrichment Analysis Resource, CHEA Transcription Factor Targets Dataset, AP1S2 Gene Set* [3].

	NP 001259000.1	NP 003907.3	NP 001355923.1	NP 001355936.1	NP 001355937.1
NP 001259000.1	1	0.9991	0.99898	0.99957	0.9992
NP 003907.3		1	0.99926	0.99956	0.99933
NP 001355923.1			0.99973	0.99957	0.9996
NP 001355936.1				1	0.99957
NP 001355937.1					0.99959

Table A51. The parameter Positive Predictive Value (PPV) of the HELIOS method with referencing the BLAST in the accuracy measurement of classification output, assuming the *ChIP-X Enrichment Analysis Resource, CHEA Transcription Factor Targets Dataset, AP1S2 Gene Set* [3].

	NP 001259000.1	NP 003907.3	NP 001355923.1	NP 001355936.1	NP 001355937.1
NP 001259000.1	1	0.9281	0.9281	0.96599	0.96599
NP 003907.3		1	0.95302	0.96599	0.96599
NP 001355923.1			1	0.96599	1
NP 001355936.1				1	0.96599
NP 001355937.1					1

Table A52. The parameter Negative Predictive Value (NPV) of the HELIOS method with referencing the BLAST in the accuracy measurement of classification output, assuming the *ChIP-X Enrichment Analysis Resource, CHEA Transcription Factor Targets Dataset, AP1S2 Gene Set* [3].

	NP 001259000.1	NP 003907.3	NP 001355923.1	NP 001355936.1	NP 001355937.1
NP 001259000.1	1	0.99955	0.99941	0.99979	0.9994
NP 003907.3		1	0.99955	0.99978	0.99954
NP 001355923.1			0.99972	0.99979	0.9996
NP 001355936.1				1	0.99978
NP 001355937.1					0.99959

Table A53. The parameter Matthew's Coefficient Correlation (MCC) of the HELIOS method with referencing the BLAST in the accuracy measurement of classification output, assuming the *ChIP-X Enrichment Analysis Resource, CHEA Transcription Factor Targets Dataset, AP1S2 Gene Set* [3].

	NP 001259000.1	NP 003907.3	NP 001355923.1	NP 001355936.1	NP 001355937.1
NP 001259000.1	1	0.92765	0.9157	0.96577	0.93432
NP 003907.3		1	0.94011	0.96576	0.94652
NP 001355923.1			0.97775	0.96577	0.96744
NP 001355936.1				1	0.96577
NP 001355937.1					0.96743

Table A54. The parameter Test's Accuracy (F-Score) of the HELIOS method with referencing the BLAST in the accuracy measurement of classification output, assuming the *ChIP-X Enrichment Analysis Resource, CHEA Transcription Factor Targets Dataset, AP1S2 Gene Set* [3].

	NP 001259000.1	NP 003907.3	NP 001355923.1	NP 001355936.1	NP 001355937.1
NP 001259000.1	1	0.9281	0.91613	0.96599	0.93421
NP 003907.3		1	0.9404	0.96599	0.94667
NP 001355923.1			0.97764	0.96599	0.96711
NP 001355936.1				1	0.96599
NP 001355937.1					0.96711

Table A55. The parameter Sensitivity (SEN) of the HELIOS method with referencing the ClustalW in the accuracy measurement of classification output, assuming the *ChIP-X Enrichment Analysis Resource, CHEA Transcription Factor Targets Dataset, AP1S2 Gene Set* [3].

	NP 001259000.1	NP 003907.3	NP 001355923.1	NP 001355936.1	NP 001355937.1
NP 001259000.1	1	1	0.95425	0.9	0.97279
NP 003907.3		1	0.96053	0.90446	1
NP 001355923.1			1	0.93464	0.98621
NP 001355936.1				1	0.97279
NP 001355937.1					1

Table A56. The parameter Specification (Spec) of the HELIOS method with referencing the ClustalW in the accuracy measurement of classification output, assuming the *ChIP-X Enrichment Analysis Resource, CHEA Transcription Factor Targets Dataset, AP1S2 Gene Set* [3].

	NP 001259000.1	NP 003907.3	NP 001355923.1	NP 001355936.1	NP 001355937.1
NP 001259000.1	1	1	0.99971	0.99949	0.99983
NP 003907.3		1	0.99971	0.9994	0.99996
NP 001355923.1			1	0.99959	0.99982
NP 001355936.1				1	0.99983
NP 001355937.1					1

Table A57. The parameter Accuracy (Acc) of the HELIOS method with referencing the ClustalW in the accuracy measurement of classification output, assuming the *ChIP-X Enrichment Analysis Resource, CHEA Transcription Factor Targets Dataset, AP1S2 Gene Set* [3].

	NP 001259000.1	NP 003907.3	NP 001355923.1	NP 001355936.1	NP 001355937.1
NP 001259000.1	1	1	0.99943	0.99887	0.99966
NP 003907.3		1	0.99946	0.99881	0.99996
NP 001355923.1			1	0.99918	0.99973
NP 001355936.1				1	0.99966
NP 001355937.1					1

Table A58. The parameter Positive Predictive Value (PPV) of the HELIOS method with referencing the ClustalW in the accuracy measurement of classification output, assuming the *ChIP-X Enrichment Analysis Resource, CHEA Transcription Factor Targets Dataset, AP1S2 Gene Set* [3].

	NP 001259000.1	NP 003907.3	NP 001355923.1	NP 001355936.1	NP 001355937.1
NP 001259000.1	1	1	0.95425	0.9172	0.97279
NP 003907.3		1	0.95425	0.90446	0.9932
NP 001355923.1			1	0.93464	0.97279
NP 001355936.1				1	0.97279
NP 001355937.1					1

Table A59. The parameter Negative Predictive Value (NPV) of the HELIOS method with referencing the ClustalW in the accuracy measurement of classification output, assuming the *ChIP-X Enrichment Analysis Resource, CHEA Transcription Factor Targets Dataset, AP1S2 Gene Set* [3].

	NP 001259000.1	NP 003907.3	NP 001355923.1	NP 001355936.1	NP 001355937.1
NP 001259000.1	1	1	0.99971	0.99937	0.99983
NP 003907.3		1	0.99975	0.9994	1
NP 001355923.1			1	0.99959	0.99991
NP 001355936.1				1	0.99983
NP 001355937.1					1

Table A60. The parameter Matthew's Coefficient Correlation (MCC) of the HELIOS method with referencing the ClustalW in the accuracy measurement of classification output, assuming the *ChIP-X Enrichment Analysis Resource, CHEA Transcription Factor Targets Dataset, AP1S2 Gene Set* [3].

	NP 001259000.1	NP 003907.3	NP 001355923.1	NP 001355936.1	NP 001355937.1
NP 001259000.1	1	1	0.95396	0.90799	0.97262
NP 003907.3		1	0.95711	0.90386	0.99657
NP 001355923.1			1	0.93423	0.97934
NP 001355936.1				1	0.97262
NP 001355937.1					1

Table A61. The parameter Test's Accuracy (F-Score) of the HELIOS method with referencing the ClustalW in the accuracy measurement of classification output, assuming the *ChIP-X Enrichment Analysis Resource, CHEA Transcription Factor Targets Dataset, AP1S2 Gene Set* [3].

	NP 001259000.1	NP 003907.3	NP 001355923.1	NP 001355936.1	NP 001355937.1
NP 001259000.1	1	1	0.95425	0.90852	0.97279
NP 003907.3		1	0.95738	0.90446	0.99659
NP 001355923.1			1	0.93464	0.97945
NP 001355936.1				1	0.97279
NP 001355937.1					1

Table A62. The parameter Sensitivity (SEN) of the HELIOS method with referencing the ClustalΩ in the accuracy measurement of classification output, assuming the *ChIP-X Enrichment Analysis Resource, CHEA Transcription Factor Targets Dataset, AP1S2 Gene Set* [3].

	NP 001259000.1	NP 003907.3	NP 001355923.1	NP 001355936.1	NP 001355937.1
NP 001259000.1	1	0.99363	0.9281	0.9	1
NP 003907.3		1	0.94667	0.91083	0.99306
NP 001355923.1			1	1	1
NP 001355936.1				1	1
NP 001355937.1					1

Table A63. The parameter Specification (Spec) of the HELIOS method with referencing the ClustalΩ in the accuracy measurement of classification output, assuming the *ChIP-X Enrichment Analysis Resource, CHEA Transcription Factor Targets Dataset, AP1S2 Gene Set* [3].

	NP 001259000.1	NP 003907.3	NP 001355923.1	NP 001355936.1	NP 001355937.1
NP 001259000.1	1	0.99996	0.99955	0.99949	1
NP 003907.3		1	0.99954	0.99944	0.99983
NP 001355923.1			1	1	1
NP 001355936.1				1	1
NP 001355937.1					1

Table A64. The parameter Accuracy (Acc) of the HELIOS method with referencing the ClustalΩ in the accuracy measurement of classification output, assuming the *ChIP-X Enrichment Analysis Resource, CHEA Transcription Factor Targets Dataset, AP1S2 Gene Set* [3].

	NP 001259000.1	NP 003907.3	NP 001355923.1	NP 001355936.1	NP 001355937.1
NP 001259000.1	1	0.99992	0.9991	0.99887	1
NP 003907.3		1	0.99921	0.99889	0.99978
NP 001355923.1			1	1	1
NP 001355936.1				1	1
NP 001355937.1					1

Table A65. The parameter Positive Predictive Value (PPV) of the HELIOS method with referencing the ClustalΩ in the accuracy measurement of classification output, assuming the *ChIP-X Enrichment Analysis Resource, CHEA Transcription Factor Targets Dataset, AP1S2 Gene Set* [3].

	NP 001259000.1	NP 003907.3	NP 001355923.1	NP 001355936.1	NP 001355937.1
NP 001259000.1	1	0.99363	0.9281	0.9172	1
NP 003907.3		1	0.9281	0.91083	0.97279
NP 001355923.1			1	1	1
NP 001355936.1				1	1
NP 001355937.1					1

Table A66. The parameter Negative Predictive Value (NPV) of the HELIOS method with referencing the ClustalΩ in the accuracy measurement of classification output, assuming the *ChIP-X Enrichment Analysis Resource, CHEA Transcription Factor Targets Dataset, AP1S2 Gene Set* [3].

	NP 001259000.1	NP 003907.3	NP 001355923.1	NP 001355936.1	NP 001355937.1
NP 001259000.1	1	0.99363	0.9281	0.9172	1
NP 003907.3		1	0.9281	0.91083	0.97279
NP 001355923.1			1	1	1
NP 001355936.1				1	1
NP 001355937.1					1

Table A67. The parameter Matthew's Coefficient Correlation (MCC) of the HELIOS method with referencing the ClustalΩ in the accuracy measurement of classification output, assuming the *ChIP-X Enrichment Analysis Resource, CHEA Transcription Factor Targets Dataset, AP1S2 Gene Set* [3].

	NP 001259000.1	NP 003907.3	NP 001355923.1	NP 001355936.1	NP 001355937.1
NP 001259000.1	1	0.99359	0.92765	0.90799	1
NP 003907.3		1	0.93694	0.91027	0.98276
NP 001355923.1			1	1	1
NP 001355936.1				1	1
NP 001355937.1					1

Table A68. The parameter Test's Accuracy (F-Score) of the HELIOS method with referencing the ClustalΩ in the accuracy measurement of classification output, assuming the *ChIP-X Enrichment Analysis Resource, CHEA Transcription Factor Targets Dataset, AP1S2 Gene Set* [3].

	NP 001259000.1	NP 003907.3	NP 001355923.1	NP 001355936.1	NP 001355937.1
NP 001259000.1	1	0.99363	0.9281	0.90852	1
NP 003907.3		1	0.93729	0.91083	0.98282
NP 001355923.1			1	1	1
NP 001355936.1				1	1
NP 001355937.1					1

Table A69. The parameter Sensitivity (SEN) of the HELIOS method with referencing the MUSCLE in the accuracy measurement of classification output, assuming the *ChIP-X Enrichment Analysis Resource, CHEA Transcription Factor Targets Dataset, AP1S2 Gene Set* [3].

	NP 001259000.1	NP 003907.3	NP 001355923.1	NP 001355936.1	NP 001355937.1
NP 001259000.1	1	0.99363	0.95425	0.9	0.95918
NP 003907.3		1	0.96667	0.89809	1
NP 001355923.1			1	0.97386	0.96575
NP 001355936.1				1	0.95918
NP 001355937.1					1

Table A70. The parameter Specification (Spec) of the HELIOS method with referencing the MUSCLE in the accuracy measurement of classification output, assuming the *ChIP-X Enrichment Analysis Resource, CHEA Transcription Factor Targets Dataset, AP1S2 Gene Set* [3].

	NP 001259000.1	NP 003907.3	NP 001355923.1	NP 001355936.1	NP 001355937.1
NP 001259000.1	1	0.99996	0.99971	0.99949	0.99974
NP 003907.3		1	0.99966	0.99936	1
NP 001355923.1			1	0.99984	0.99973
NP 001355936.1				1	0.99974
NP 001355937.1					1

Table A71. The parameter Accuracy (Acc) of the HELIOS method with referencing the MUSCLE in the accuracy measurement of classification output, assuming the *ChIP-X Enrichment Analysis Resource, CHEA Transcription Factor Targets Dataset, AP1S2 Gene Set* [3].

	NP 001259000.1	NP 003907.3	NP 001355923.1	NP 001355936.1	NP 001355937.1
NP 001259000.1	1	0.99992	0.99943	0.99887	0.99949
NP 003907.3		1	0.99946	0.99873	1
NP 001355923.1			1	0.99967	0.99951
NP 001355936.1				1	0.99949
NP 001355937.1					1

Table A72. The parameter Positive Predictive Value (PPV) of the HELIOS method with referencing the MUSCLE in the accuracy measurement of classification output, assuming the *ChIP-X Enrichment Analysis Resource, CHEA Transcription Factor Targets Dataset, AP1S2 Gene Set* [3].

	NP 001259000.1	NP 003907.3	NP 001355923.1	NP 001355936.1	NP 001355937.1
NP 001259000.1	1	0.99363	0.95425	0.9172	0.95918
NP 003907.3		1	0.94771	0.89809	1
NP 001355923.1			1	0.97386	0.95918
NP 001355936.1				1	0.95918
NP 001355937.1					1

Table A73. The parameter Negative Predictive Value (NPV) of the HELIOS method with referencing the MUSCLE in the accuracy measurement of classification output, assuming the *ChIP-X Enrichment Analysis Resource, CHEA Transcription Factor Targets Dataset, AP1S2 Gene Set* [3].

	NP 001259000.1	NP 003907.3	NP 001355923.1	NP 001355936.1	NP 001355937.1
NP 001259000.1	1	0.99996	0.99971	0.99937	0.99974
NP 003907.3		1	0.99979	0.99936	1
NP 001355923.1			1	0.99984	0.99978
NP 001355936.1				1	0.99974
NP 001355937.1					1

Table A74. The parameter Matthew's Coefficient Correlation (MCC) of the HELIOS method with referencing the MUSCLE in the accuracy measurement of classification output, assuming the *ChIP-X Enrichment Analysis Resource, CHEA Transcription Factor Targets Dataset, AP1S2 Gene Set* [3].

	NP 001259000.1	NP 003907.3	NP 001355923.1	NP 001355936.1	NP 001355937.1
NP 001259000.1	1	0.99359	0.95396	0.90799	0.95893
NP 003907.3		1	0.95687	0.89745	1
NP 001355923.1			1	0.97369	0.96222
NP 001355936.1				1	0.95893
NP 001355937.1					1

Table A75. The parameter Test's Accuracy (F-Score) of the HELIOS method with referencing the MUSCLE in the accuracy measurement of classification output, assuming the *ChIP-X Enrichment Analysis Resource, CHEA Transcription Factor Targets Dataset, AP1S2 Gene Set* [3].

	NP 001259000.1	NP 003907.3	NP 001355923.1	NP 001355936.1	NP 001355937.1
NP 001259000.1	1	0.99363	0.95425	0.90852	0.95918
NP 003907.3		1	0.9571	0.89809	1
NP 001355923.1			1	0.97386	0.96246
NP 001355936.1				1	0.95918
NP 001355937.1					1

Table A76. The parameter Sensitivity (SEN) of the HELIOS method with referencing the T-Coffee in the accuracy measurement of classification output, assuming the *ChIP-X Enrichment Analysis Resource, CHEA Transcription Factor Targets Dataset, AP1S2 Gene Set* [3].

	NP 001259000.1	NP 003907.3	NP 001355923.1	NP 001355936.1	NP 001355937.1
NP 001259000.1	1	0.96078	0.90446	0.96599	0.93631
NP 003907.3		1	0.9281	1	0.96078
NP 001355923.1			0.95625	1	0.93631
NP 001355936.1				1	1
NP 001355937.1					0.93631

Table A77. The parameter Specification (Spec) of the HELIOS method with referencing the T-Coffee in the accuracy measurement of classification output, assuming the *ChIP-X Enrichment Analysis Resource, CHEA Transcription Factor Targets Dataset, AP1S2 Gene Set* [3].

	NP 001259000.1	NP 003907.3	NP 001355923.1	NP 001355936.1	NP 001355937.1
NP 001259000.1	1	0.99975	0.99957	0.99979	1
NP 003907.3		1	0.99971	1	1
NP 001355923.1			1	1	1
NP 001355936.1				1	1
NP 001355937.1					1

Table A78. The parameter Accuracy (Acc) of the HELIOS method with referencing the T-Coffee in the accuracy measurement of classification output, assuming the *ChIP-X Enrichment Analysis Resource, CHEA Transcription Factor Targets Dataset, AP1S2 Gene Set* [3].

	NP 001259000.1	NP 003907.3	NP 001355923.1	NP 001355936.1	NP 001355937.1
NP 001259000.1	1	0.99951	0.99898	0.99957	0.9996
NP 003907.3		1	0.99926	1	0.99975
NP 001355923.1			0.99973	1	0.9996
NP 001355936.1				1	1
NP 001355937.1					0.99959

Table A79. The parameter Positive Predictive Value (PPV) of the HELIOS method with referencing the T-Coffee in the accuracy measurement of classification output, assuming the *ChIP-X Enrichment Analysis Resource, CHEA Transcription Factor Targets Dataset, AP1S2 Gene Set* [3].

	NP 001259000.1	NP 003907.3	NP 001355923.1	NP 001355936.1	NP 001355937.1
NP 001259000.1	1	0.96078	0.9281	0.96599	1
NP 003907.3		1	0.95302	1	1
NP 001355923.1			1	1	1
NP 001355936.1				1	1
NP 001355937.1					1

Table A80. The parameter Negative Predictive Value (NPV) of the HELIOS method with referencing the T-Coffee in the accuracy measurement of classification output, assuming the *ChIP-X Enrichment Analysis Resource, CHEA Transcription Factor Targets Dataset, AP1S2 Gene Set* [3].

	NP 001259000.1	NP 003907.3	NP 001355923.1	NP 001355936.1	NP 001355937.1
NP 001259000.1	1	0.99975	0.99941	0.99979	0.9996
NP 003907.3		1	0.99955	1	0.99975
NP 001355923.1			0.99972	1	0.9996
NP 001355936.1				1	1
NP 001355937.1					0.99959

Table A81. The parameter Matthew's Coefficient Correlation (MCC) of the HELIOS method with referencing the T-Coffee in the accuracy measurement of classification output, assuming the *ChIP-X Enrichment Analysis Resource, CHEA Transcription Factor Targets Dataset, AP1S2 Gene Set* [3].

	NP 001259000.1	NP 003907.3	NP 001355923.1	NP 001355936.1	NP 001355937.1
NP 001259000.1	1	0.96054	0.9157	0.96577	0.96744
NP 003907.3		1	0.94011	1	0.98007
NP 001355923.1			0.97775	1	0.96744
NP 001355936.1				1	1
NP 001355937.1					0.96743

Table A82. The parameter Test's Accuracy (F-Score) of the HELIOS method with referencing the T-Coffee in the accuracy measurement of classification output, assuming the *ChIP-X Enrichment Analysis Resource, CHEA Transcription Factor Targets Dataset, AP1S2 Gene Set* [3].

	NP 001259000.1	NP 003907.3	NP 001355923.1	NP 001355936.1	NP 001355937.1
NP 001259000.1	1	0.96078	0.91613	0.96599	0.96711
NP 003907.3		1	0.9404	1	0.98
NP 001355923.1			0.97764	1	0.96711
NP 001355936.1				1	1
NP 001355937.1					0.96711

Table A83. The parameter Sensitivity (SEN) of the HELIOS method with referencing the Kalign in the accuracy measurement of classification output, assuming the *ChIP-X Enrichment Analysis Resource, CHEA Transcription Factor Targets Dataset, AP1S2 Gene Set* [3].

	NP 001259000.1	NP 003907.3	NP 001355923.1	NP 001355936.1	NP 001355937.1
NP 001259000.1	1	1	0.9281	0.9	1
NP 003907.3		1	0.94667	0.90446	0.98611
NP 001355923.1			1	1	1
NP 001355936.1				1	1
NP 001355937.1					1

Table A84. The parameter Specification (Spec) of the HELIOS method with referencing the Kalign in the accuracy measurement of classification output, assuming the *ChIP-X Enrichment Analysis Resource, CHEA Transcription Factor Targets Dataset, AP1S2 Gene Set* [3].

	NP 001259000.1	NP 003907.3	NP 001355923.1	NP 001355936.1	NP 001355937.1
NP 001259000.1	1	1	0.99955	0.99949	1
NP 003907.3		1	0.99954	0.9994	0.99978
NP 001355923.1			1	1	1
NP 001355936.1				1	1
NP 001355937.1					1

Table A85. The parameter Accuracy (Acc) of the HELIOS method with referencing the Kalign in the accuracy measurement of classification output, assuming the *ChIP-X Enrichment Analysis Resource, CHEA Transcription Factor Targets Dataset, AP1S2 Gene Set* [3].

	NP 001259000.1	NP 003907.3	NP 001355923.1	NP 001355936.1	NP 001355937.1
NP 001259000.1	1	1	0.9991	0.99887	1
NP 003907.3		1	0.99921	0.99881	0.9997
NP 001355923.1			1	1	1
NP 001355936.1				1	1
NP 001355937.1					1

Table A86. The parameter Positive Predictive Value (PPV) of the HELIOS method with referencing the Kalign in the accuracy measurement of classification output, assuming the *ChIP-X Enrichment Analysis Resource, CHEA Transcription Factor Targets Dataset, AP1S2 Gene Set* [3].

	NP 001259000.1	NP 003907.3	NP 001355923.1	NP 001355936.1	NP 001355937.1
NP 001259000.1	1	1	0.9281	0.9172	1
NP 003907.3		1	0.9281	0.90446	0.96599
NP 001355923.1			1	1	1
NP 001355936.1				1	1
NP 001355937.1					1

Table A87. The parameter Negative Predictive Value (NPV) of the HELIOS method with referencing the Kalign in the accuracy measurement of classification output, assuming the *ChIP-X Enrichment Analysis Resource, CHEA Transcription Factor Targets Dataset, AP1S2 Gene Set* [3].

	NP 001259000.1	NP 003907.3	NP 001355923.1	NP 001355936.1	NP 001355937.1
NP 001259000.1	1	1	0.99955	0.99937	1
NP 003907.3		1	0.99966	0.9994	0.99991
NP 001355923.1			1	1	1
NP 001355936.1				1	1
NP 001355937.1					1

Table A88. The parameter Matthew's Coefficient Correlation (MCC) of the HELIOS method with referencing the Kalign in the accuracy measurement of classification output, assuming the *ChIP-X Enrichment Analysis Resource, CHEA Transcription Factor Targets Dataset, AP1S2 Gene Set* [3].

	NP 001259000.1	NP 003907.3	NP 001355923.1	NP 001355936.1	NP 001355937.1
NP 001259000.1	1	1	0.92765	0.90799	1
NP 003907.3		1	0.93694	0.90386	0.97584
NP 001355923.1			1	1	1
NP 001355936.1				1	1
NP 001355937.1					1

Table A89. The parameter Test's Accuracy (F-Score) of the HELIOS method with referencing the Kalign in the accuracy measurement of classification output, assuming the *ChIP-X Enrichment Analysis Resource, CHEA Transcription Factor Targets Dataset, AP1S2 Gene Set* [3].

	NP 001259000.1	NP 003907.3	NP 001355923.1	NP 001355936.1	NP 001355937.1
NP 001259000.1	1	1	0.9281	0.90852	1
NP 003907.3		1	0.93729	0.90446	0.97595
NP 001355923.1			1	1	1
NP 001355936.1				1	1
NP 001355937.1					1

Table A90. The parameter Sensitivity (SEN) of the HELIOS method with referencing the MAFFT in the accuracy measurement of classification output, assuming the *ChIP-X Enrichment Analysis Resource, CHEA Transcription Factor Targets Dataset, AP1S2 Gene Set* [3].

	NP 001259000.1	NP 003907.3	NP 001355923.1	NP 001355936.1	NP 001355937.1
NP 001259000.1	1	1	0.94118	1	0.96599
NP 003907.3		1	0.94118	0.98726	0.96599
NP 001355923.1			1	0.98693	0.96599
NP 001355936.1				1	0.96599
NP 001355937.1					1

Table A91. The parameter Specification (Spec) of the HELIOS method with referencing the MAFFT in the accuracy measurement of classification output, assuming the *ChIP-X Enrichment Analysis Resource, CHEA Transcription Factor Targets Dataset, AP1S2 Gene Set* [3].

	NP 001259000.1	NP 003907.3	NP 001355923.1	NP 001355936.1	NP 001355937.1
NP 001259000.1	1	1	0.99963	1	0.99979
NP 003907.3		1	0.99962	0.99992	0.99978
NP 001355923.1			1	0.99992	0.99978
NP 001355936.1				1	0.99979
NP 001355937.1					1

Table A92. The parameter Accuracy (Acc) of the HELIOS method with referencing the MAFFT in the accuracy measurement of classification output, assuming the *ChIP-X Enrichment Analysis Resource, CHEA Transcription Factor Targets Dataset, AP1S2 Gene Set* [3].

	NP 001259000.1	NP 003907.3	NP 001355923.1	NP 001355936.1	NP 001355937.1
NP 001259000.1	1	1	0.99926	1	0.99957
NP 003907.3		1	0.99925	0.99984	0.99957
NP 001355923.1			1	0.99984	0.99956
NP 001355936.1				1	0.99957
NP 001355937.1					1

Table A93. The parameter Positive Predictive Value (PPV) of the HELIOS method with referencing the MAFFT in the accuracy measurement of classification output, assuming the *ChIP-X Enrichment Analysis Resource, CHEA Transcription Factor Targets Dataset, AP1S2 Gene Set* [3].

	NP 001259000.1	NP 003907.3	NP 001355923.1	NP 001355936.1	NP 001355937.1
NP 001259000.1	1	1	0.94118	1	0.96599
NP 003907.3		1	0.94118	0.98726	0.96599
NP 001355923.1			1	0.98693	0.96599
NP 001355936.1				1	0.96599
NP 001355937.1					1

Table A94. The parameter Negative Predictive Value (NPV) of the HELIOS method with referencing the MAFFT in the accuracy measurement of classification output, assuming the *ChIP-X Enrichment Analysis Resource, CHEA Transcription Factor Targets Dataset, AP1S2 Gene Set* [3].

	NP 001259000.1	NP 003907.3	NP 001355923.1	NP 001355936.1	NP 001355937.1
NP 001259000.1	1	1	0.99963	1	0.99979
NP 003907.3		1	0.99962	0.99992	0.99978
NP 001355923.1			1	0.99992	0.99978
NP 001355936.1				1	0.99979
NP 001355937.1					1

Table A95. The parameter Matthew's Coefficient Correlation (MCC) of the HELIOS method with referencing the MAFFT in the accuracy measurement of classification output, assuming the *ChIP-X Enrichment Analysis Resource, CHEA Transcription Factor Targets Dataset, AP1S2 Gene Set* [3].

	NP 001259000.1	NP 003907.3	NP 001355923.1	NP 001355936.1	NP 001355937.1
NP 001259000.1	1	1	0.94081	1	0.96577
NP 003907.3		1	0.9408	0.98718	0.96577
NP 001355923.1			1	0.98685	0.96576
NP 001355936.1				1	0.96577
NP 001355937.1					1

Table A96. The parameter Test's Accuracy (F-Score) of the HELIOS method with referencing the MAFFT in the accuracy measurement of classification output, assuming the *ChIP-X Enrichment Analysis Resource, CHEA Transcription Factor Targets Dataset, AP1S2 Gene Set* [3].

	NP 001259000.1	NP 003907.3	NP 001355923.1	NP 001355936.1	NP 001355937.1
NP 001259000.1	1	1	0.94118	1	0.96599
NP 003907.3		1	0.94118	0.98726	0.96599
NP 001355923.1			1	0.98693	0.96599
NP 001355936.1				1	0.96599
NP 001355937.1					1
