# Supplementary material for: HELIOS: High-speed sequence alignment in optics
Source: PLoS Comput Biol. 2022 Nov 21;18(11):e1010665. doi: 10.1371/journal.pcbi.1010665 (PMC9678324; doi:10.1371/journal.pcbi.1010665)
Supplement: S8 Text — (PDF) [file pcbi.1010665.s008.pdf]

HELIOS: High-Speed Sequence Alignment in Optics: S8 Text

EHSAN MALEKI[1], SAEEDEH AKBARI ROKN ABADI[1], AND SOMAYYEH KOOHI[1,*]

[1] *Department of Computer Engineering, Sharif University of Technology, Azadi Ave., Tehran, Iran.*
[*] *Corresponding author: koohi@sharif.edu*

Compiled October 20, 2022

In response to the imperfections of current sequence alignment methods, originated from the inherent serialism within their corresponding electrical systems, a few optical approaches for biological data comparison have been proposed recently. However, due to their low performance, raised from their inefficient coding scheme, this paper presents a novel all-optical high-throughput method for aligning DNA, RNA, and protein sequences, named HELIOS. The HELIOS method employs highly sophisticated operations to locate character matches, single or multiple mutations, and single or multiple indels within various biological sequences. On the other hand, the HELIOS optical architecture exploits high-speed processing and operational parallelism in optics, by adopting wavelength and polarization of optical beams. For evaluation, the functionality and accuracy of the HELIOS method are approved through behavioral and optical simulation studies, while its complexity and performance are estimated through analytical computation. The accuracy evaluations indicate that the HELIOS method achieves a precise pairwise alignment of two sequences, highly similar to those of Smith-Waterman, Needleman-Wunsch, BLAST, MUSCLE, ClustalW, ClustalΩ, T-Coffee, Kalign, and MAFFT. According to our performance evaluations, the HELIOS optical architecture outperforms all alternative electrical and optical algorithms in terms of processing time and memory requirement, relying on its highly sophisticated method and optical architecture. Moreover, the employed compact coding scheme highly escalates the number of input characters, and hence, it offers reduced time and space complexities, compared to the electrical and optical alternatives. It makes the HELIOS method and optical architecture highly applicable for biomedical applications.

1. ACCURACY EVALUATION

In order to comprehensively assess the accuracy of the HELIOS method, two statistical analyses are performed through simulation studies: 1) Quantitative measurement of homology [1], and 2) Accuracy measurement of classification output [2], compared to the well-known algorithms, briefly reported in Tables A1 and A2, respectively. In this manner, the *Encyclopedia of DNA Elements Resource, ENCODE Transcription Factor Targets Dataset, SP1 Gene Set* [3] is assumed in this study, as represented in Table A3.

A. Quantitative measurement of homology

To perform quantitative measurement of homology [1], the parameters Identity, Similarity, and Alignment Score of the HELIOS outputs are calculated through simulation studies, as reported in Tables A4-A6, respectively, assuming the *Encyclopedia of DNA Elements Resource, ENCODE Transcription Factor Targets Dataset, SP1 Gene Set* [3]. While the Identity reports the number of exactly matched characters of two sequences (in percentage), the Similarity measures the resemblance of two compared sequences. Specifically, regarding the physicochemical properties, the amino acids are categorized into six groups with different Similarity values; including GAVLI, FYW, STCM, KRH, DENQ, and P. As the third metric, the BLOSUM62 [4] substitution scoring matrix [4] is adopted to calculate the Alignment Score, with gap opening and extension penalties equal to -10 and -0.5, respectively.

For a comparative study, the values of Identity, Similarity, and Alignment Score of the quantitative measurement of homology is performed by various well-known algorithms to be compared to the those of HELIOS method, assuming *Encyclopedia of DNA Elements Resource, ENCODE Transcription Factor Targets Dataset, SP1 Gene Set* [3]. It includes Smith-Waterman (SW) [5] reported in Tables A7-A9, Needleman-Wunsch (NW) [6] reported in Tables A10-A12, BLAST [7] reported in Tables A13-A15, ClustalW [8] reported in Tables A16-A18, Clustal-Omega [9] reported in Tables A19-A21, MUSCLE [9] reported in Tables A22-A24, T-Coffee [10] reported in Tables A25-A27, Kalign [11] reported in Tables A28-A30, and MAFFT [12] reported in Tables A31-A33.

Table A1. A brief report of the quantitative measurement of homology of the HELIOS method, compared to nine well-known algorithms, including SW, NW, BLAST, ClustalW, Clustal Omega, Muscle, T-Coffee, Kalign, and MAFFT. The parameters Identity, Similarity, and Alignment score are averaged and reported. The dataset used in this study is the *Encyclopedia of DNA Elements Resource, ENCODE Transcription Factor Targets Dataset, SP1 Gene Set* [3].

	HELIOS	SW	NW	BLAST	MUSCLE	ClustalW	ClustalΩ	T-Coffee	Kalign	MAFFT
Identity	99.841	97.874	97.874	99.796	99.841	99.841	99.841	99.841	99.841	99.841
Similarity	99.841	98.281	97.919	99.796	99.841	99.841	99.841	99.841	99.841	99.841
Alignment Score	3865.0	3780.5	3799.5	3859.1	3865.0	3865.0	3865.0	3865.0	3865.0	3865.0

Table A2. A brief report of the accuracy measurement of classification output of the HELIOS method with referencing well-known algorithms, including SW, NW, BLAST, ClustalW, Clustal Omega, Muscle, T-Coffee, Kalign, and MAFFT. The parameters SEN, Spec, Acc, PPV, NPV, MCC, and F-Score are averaged and reported. The dataset used in this study is the *Encyclopedia of DNA Elements Resource, ENCODE Transcription Factor Targets Dataset, SP1 Gene Set* [3].

	SW	NW	BLAST	MUSCLE	ClustalW	ClustalΩ	T-Coffee	Kalign	MAFFT
SEN	0.97558	0.50587	0.99773	0.99955	1.00000	0.99955	0.99955	1.00000	0.99955
Spec	0.99997	0.99936	1.00000	1.00000	1.00000	1.00000	1.00000	1.00000	1.00000
ACC	0.99994	0.99868	0.99999	1.00000	1.00000	1.00000	1.00000	1.00000	1.00000
PPV	0.97706	0.50628	0.99773	0.99955	1.00000	0.99955	0.99955	1.00000	0.99955
NPV	0.99997	0.99932	1.00000	1.00000	1.00000	1.00000	1.00000	1.00000	1.00000
MCC	0.97629	0.50541	0.99772	0.99954	1.00000	0.99954	0.99954	1.00000	0.99954
F-Score	0.97631	0.50607	0.99773	0.99955	1.00000	0.99955	0.99955	1.00000	0.99955

B. Accuracy measurement of classification output

Afterward, the accuracy measurement of the classification output [2] of the HELIOS method is addressed by calculating the values of Sensitivity (SEN), Specificity (Spec), Accuracy (ACC), Positive Predictive Value (PPV), Negative Predictive Value (NPV), Matthew's Coefficient Correlation (MCC), and Test's Accuracy (F-Score) in the simulation studies, according to according to Eq 5 to Eq 11, respectively.

As a comparative study, the accuracy measurement of the classification output of the HELIOS method is accomplished, assuming *Encyclopedia of DNA Elements Resource, ENCODE Transcription Factor Targets Dataset, SP1 Gene Set* [3], and the corresponding metrics are calculated with considering Smith-Waterman [5] reported in Tables A34-A40, Needleman-Wunsch [6] reported in Tables A41-A47, ClustalW [8] reported in Tables A55-A61, Clustal-Omega [9] reported in Tables A62-A68, BLAST [7] reported in Tables A48-A54, MUSCLE [13] reported in Tables A69-A75, T-Coffee [10] reported in Tables A76-A82, Kalign [11] reported in Tables A83-A89, and MAFFT [12] reported in Tables A90-A96.

REFERENCES

1. D. S. Moss, S. Jelaska, and S. Pongor, *Essays in bioinformatics*, vol. 368 (IOS Press, 2005).
2. M. Hamada, H. Kiryu, W. Iwasaki, and K. Asai, "Generalized centroid estimators in bioinformatics," PloS one **6**, e16450 (2011).
3. E. P. Consortium *et al.*, "The encode (encyclopedia of dna elements) project," Science **306**, 636–640 (2004).
4. D. W. Mount, "Using blosum in sequence alignments," Cold Spring Harb. Protoc. **2008**, pdb.top39 (2008).
5. H. Zou, S. Tang, C. Yu, H. Fu, Y. Li, and W. Tang, "asw: accelerating smith–waterman algorithm on coupled cpu–gpu architecture," Int. J. Parallel Program. **47**, 388–402 (2019).
6. Y. Jararweh, M. Al-Ayyoub, M. Fakirah, L. Alawneh, and B. B. Gupta, "Improving the performance of the needleman-wunsch algorithm using parallelization and vectorization techniques," Multimed. Tools Appl. **78**, 3961–3977 (2019).
7. G. M. Boratyn, J. Thierry-Mieg, D. Thierry-Mieg, B. Busby, and T. L. Madden, "Magic-blast, an accurate rna-seq aligner for long and short reads," BMC bioinformatics **20**, 1–19 (2019).
8. D. Díaz, F. J. Esteban, P. Hernández, J. A. Caballero, A. Guevara, G. Dorado, and S. Gálvez, "Mc64-clustalwp2: A highly-parallel hybrid strategy to align multiple sequences in many-core architectures," PLOS ONE **9**, 1–12 (2014).
9. F. Sievers and D. G. Higgins, "Clustal omega for making accurate alignments of many protein sequences," Protein Sci. **27**, 135–145 (2018).
10. C. Notredame, D. G. Higgins, and J. Heringa, "T-coffee: a novel method for fast and accurate multiple sequence alignment11edited by j. thornton," J. Mol. Biol. **302**, 205–217 (2000).
11. T. Lassmann, "Kalign 3: multiple sequence alignment of large datasets," (2020).
12. J. Rozewicki, S. Li, K. M. Amada, D. M. Standley, and K. Katoh, "Mafft-dash: integrated protein sequence and structural alignment," Nucleic acids research **47**, W5–W10 (2019).
13. R. C. Edgar, "MUSCLE: multiple sequence alignment with high accuracy and high throughput," Nucleic Acids Res. **32**, 1792–1797 (2004).

Table A3. The list of input sequences, assuming the *Encyclopedia of DNA Elements Resource, ENCODE Transcription Factor Targets Dataset, SP1 Gene Set* [3].

Name	Sequence
NP 001238754.1	MSDQD HSMDE MTAVV KIEKG VGGNN GGNGN GGGAF SQARS SSTGS SSSTG GGGQG ANGWQ IISSS SGATP TSKEQ SGSST NGSNG SESSK NRTVS GGQYV VAAAP NLQNQ QVLTG LPGVM PNIQY QVIPQ FQTVD GQQLQ FAATG AQVQQ DGSGQ IQIIP GANQQ IITNR GSGGN IIAAM PNLLQ QAVPL QGLAN NVLSG QTQYV TNVPV ALNGN ITLLP VNSVS AATLT PSSQA VTISS SGSQE SGSQP VTSGT TISSA SLVSS QASSS SFFTN ANSYS TTTTT SNMGI MNFTT SGSSG TNSQG QTPQR VSGLQ GSDAL NIQQN QTSGG SLQAG QQKEG EQNQQ TQQQQ ILIQP QLVQG GQALQ ALQAA PLSGQ TFTTQ AISQE TLQNL QLQAV PNSGP IIIRT PTVGP NGQVS WQTLQ LQNLQ VQNPQ AQTIT LAPMQ GVSLG QTSSS NTTLT PIASA ASIPA GTVTV NAAQL SSMPG LQTIN LSALG TSGIQ VHPIQ GLPLA IANAP GDHGA QLGLH GAGGD GIHDD TAGGE EGENS PDAQP QAGRR TRREA CTCPY CKDSE GRGSG DPGKK KQHIC HIQGC GKVYG KTSHL RAHLR WHTGE RPFMC TWSYC GKRFT RSDEL QRHKR THTGE KKFAC PECPK RFMRS DHLSK HIKTH QNKKG GPGVA LSVGT LPLDS GAGSE GSGTA TPSAL ITTNM VAMEA ICPEG IARLA NSGIN VMQVA DLQSI NISGN GF
NP 003100.1	MDEMT AVVKI EKGVG GNNGG NGNGG GAFSQ ARSSS TGSSS STGGG GQESQ PSPLA LLAAT CSRIE SPNEN SNNSQ GPSQS GGTGE LDLTA TQLSQ GANGW QIISS SSGAT PTSKE QSGSS TNGSN GSESS KNRTV SGGQY VVAAA PNLQN QQVLT GLPGV MPNIQ YQVIP QFQTV DGQQL QFAAT GAQVQ QDGSG QIQII PGANQ QIITN RGSGG NIIAA MPNLL QQAVP LQGLA NNVLS GQTQY VTNVP VALNG NITLL PVNSV SAATL TPSSQ AVTIS SSGSQ ESGSQ PVTSG TTISS ASLVS SQASS SSFFT NANSY STTTT TSNMG IMNFT TSGSS GTNSQ GQTPQ RVSGL QGSDA LNIQQ NQTSG GSLQA GQQKE GEQNQ QTQQQ QILIQ PQLVQ GGQAL QALQA APLSG QTFTT QAISQ ETLQN LQLQA VPNSG PIIIR TPTVG PNGQV SWQTL QLQNL QVQNP QAQTI TLAPM QGVSL GQTSS SNTTL TPIAS AASIP AGTVT VNAAQ LSSMP GLQTI NLSAL GTSGI QVHPI QGLPL AIANA PGDHG AQLGL HGAGG DGIHD DTAGG EEGEN SPDAQ PQAGR RTRRE ACTCP YCKDS EGRGS GDPGK KKQHI CHIQG CGKVY GKTSH LRAHL RWHTG ERPFM CTWSY CGKRF TRSDE LQRHK RTHTG EKKFA CPECP KRFMR SDHLS KHIKT HQNKK GGPGV ALSVG TLPLD SGAGS EGSGT ATPSA LITTN MVAME AICPE GIARL ANSGI NVMQV ADLQS INISG NGF
NP 612482.2	MSDQD HSMDE MTAVV KIEKG VGGNN GGNGN GGGAF SQARS SSTGS SSSTG GGGQE SQPSP LALLA ATCSR IESPN ENSNN SQGPS QSGGT GELDL TATQL SQGAN GWQII SSSSG ATPTS KEQSG SSTNG SNGSE SSKNR TVSGG QYVVA AAPNL QNQQV LTGLP GVMPN IQYQV IPQFQ TVDGQ QLQFA ATGAQ VQQDG SGQIQ IIPGA NQQII TNRGS GGNII AAMPN LLQQA VPLQG LANNV LSGQT QYVTN VPVAL NGNIT LLPVN SVSAA TLTPS SQAVT ISSSG SQESG SQPVT SGTTI SSASL VSSQA SSSSF FTNAN SYSTT TTTSN MGIMN FTTSG SSGTN SQGQT PQRVS GLQGS DALNI QQNQT SGGSL QAGQQ KEGEQ NQQTQ QQQIL IQPQL VQGGQ ALQAL QAAPL SGQTF TTQAI SQETL QNLQL QAVPN SGPII IRTPT VGPNG QVSWQ TLQLQ NLQVQ NPQAQ TITLA PMQGV SLGQT SSSNT TLTPI ASAAS IPAGT VTVNA AQLSS MPGLQ TINLS ALGTS GIQVH PIQGL PLAIA NAPGD HGAQL GLHGA GGDGI HDDTA GGEEG ENSPD AQPQA GRRTR REACT CPYCK DSEGR GSGDP GKKKQ HICHI QGCGK VYGKT SHLRA HLRWH TGERP FMCTW SYCGK RFTRS DELQR HKRTH TGEKK FACPE CPKRF MRSDH LSKHI KTHQN KKGGP GVALS VGTLP LDSGA GSEGS GTATP SALIT TNMVA MEAIC PEGIA RLANS GINVM QVADL QSINI SGNGF

Table A4. The parameter Identity of the HELIOS method in the quantitative measurement of homology, assuming the *Encyclopedia of DNA Elements Resource, ENCODE Transcription Factor Targets Dataset, SP1 Gene Set* [3].

	NP 001238754.1	NP 003100.1	NP 612482.2
NP 001238754.1	100	99.0502	100
NP 003100.1		100	100
NP 612482.2			100

Table A5. The parameter Similarity of the HELIOS method in the quantitative measurement of homology, assuming the *Encyclopedia of DNA Elements Resource, ENCODE Transcription Factor Targets Dataset, SP1 Gene Set* [3].

	NP 001238754.1	NP 003100.1	NP 612482.2
NP 001238754.1	100	99.0502	100
NP 003100.1		100	100
NP 612482.2			100

Table A6. The parameter Alignment Score of the HELIOS method in the quantitative measurement of homology, assuming the *Encyclopedia of DNA Elements Resource, ENCODE Transcription Factor Targets Dataset, SP1 Gene Set* [3].

	NP 001238754.1	NP 003100.1	NP 612482.2
NP 001238754.1	3779	3694.5	3745.5
NP 003100.1		3982	3969
NP 612482.2			4020

Table A7. The parameter Identity of the Smith-Waterman algorithm in the quantitative measurement of homology, assuming the *Encyclopedia of DNA Elements Resource, ENCODE Transcription Factor Targets Dataset, SP1 Gene Set* [3].

	NP 001238754.1	NP 003100.1	NP 612482.2
NP 001238754.1	100	93.6228	93.6228
NP 003100.1		100	100
NP 612482.2			100

Table A8. The parameter Similarity of the Smith-Waterman algorithm in the quantitative measurement of homology, assuming the *Encyclopedia of DNA Elements Resource, ENCODE Transcription Factor Targets Dataset, SP1 Gene Set* [3].

	NP 001238754.1	NP 003100.1	NP 612482.2
NP 001238754.1	100	94.844	94.844
NP 003100.1		100	100
NP 612482.2			100

Table A9. The parameter Alignment Score of the Smith-Waterman algorithm in the quantitative measurement of homology, assuming the *Encyclopedia of DNA Elements Resource, ENCODE Transcription Factor Targets Dataset, SP1 Gene Set* [3].

	NP 001238754.1	NP 003100.1	NP 612482.2
NP 001238754.1	3779	3468.5	3465
NP 003100.1		3982	3969
NP 612482.2			4020

Table A10. The parameter Identity of the Needleman-Wunsch algorithm in the quantitative measurement of homology, assuming the *Encyclopedia of DNA Elements Resource, ENCODE Transcription Factor Targets Dataset, SP1 Gene Set* [3].

	NP 001238754.1	NP 003100.1	NP 612482.2
NP 001238754.1	100	93.6228	93.6228
NP 003100.1		100	100
NP 612482.2			100

Table A11. The parameter Similarity of the Needleman-Wunsch algorithm in the quantitative measurement of homology, assuming the *Encyclopedia of DNA Elements Resource, ENCODE Transcription Factor Targets Dataset, SP1 Gene Set* [3].

	NP 001238754.1	NP 003100.1	NP 612482.2
NP 001238754.1	100	93.7585	93.7585
NP 003100.1		100	100
NP 612482.2			100

Table A12. The parameter Alignment Score of the Needleman-Wunsch algorithm in the quantitative measurement of homology, assuming the *Encyclopedia of DNA Elements Resource, ENCODE Transcription Factor Targets Dataset, SP1 Gene Set* [3].

	NP 001238754.1	NP 003100.1	NP 612482.2
NP 001238754.1	3779	3517	3517
NP 003100.1		3982	3982
NP 612482.2			4020

Table A13. The parameter Identity of the BLAST in the quantitative measurement of homology, assuming the *Encyclopedia of DNA Elements Resource, ENCODE Transcription Factor Targets Dataset, SP1 Gene Set* [3].

	NP 001238754.1	NP 003100.1	NP 612482.2
NP 001238754.1	100	98.9145	99.8643
NP 003100.1		100	100
NP 612482.2			100

Table A14. The parameter Similarity of the BLAST in the quantitative measurement of homology, assuming the *Encyclopedia of DNA Elements Resource, ENCODE Transcription Factor Targets Dataset, SP1 Gene Set* [3].

	NP 001238754.1	NP 003100.1	NP 612482.2
NP 001238754.1	100	98.9145	99.8643
NP 003100.1		100	100
NP 612482.2			100

Table A15. The parameter Alignment Score of the BLAST in the quantitative measurement of homology, assuming the *Encyclopedia of DNA Elements Resource, ENCODE Transcription Factor Targets Dataset, SP1 Gene Set* [3].

	NP 001238754.1	NP 003100.1	NP 612482.2
NP 001238754.1	3779	3677	3728
NP 003100.1		3982	3969
NP 612482.2			4020

Table A16. The parameter Identity of the ClustalW in the quantitative measurement of homology, assuming the *Encyclopedia of DNA Elements Resource, ENCODE Transcription Factor Targets Dataset, SP1 Gene Set* [3].

	NP 001238754.1	NP 003100.1	NP 612482.2
NP 001238754.1	100	99.0502	100
NP 003100.1		100	100
NP 612482.2			100

Table A17. The parameter Similarity of the ClustalW in the quantitative measurement of homology, assuming the *Encyclopedia of DNA Elements Resource, ENCODE Transcription Factor Targets Dataset, SP1 Gene Set* [3].

	NP 001238754.1	NP 003100.1	NP 612482.2
NP 001238754.1	100	99.0502	100
NP 003100.1		100	100
NP 612482.2			100

Table A18. The parameter Alignment Score of the ClustalW in the quantitative measurement of homology, assuming the *Encyclopedia of DNA Elements Resource, ENCODE Transcription Factor Targets Dataset, SP1 Gene Set* [3].

	NP 001238754.1	NP 003100.1	NP 612482.2
NP 001238754.1	3779	3694.5	3745.5
NP 003100.1		3982	3969
NP 612482.2			4020

Table A19. The parameter Identity of the ClustalΩ in the quantitative measurement of homology, assuming the *Encyclopedia of DNA Elements Resource, ENCODE Transcription Factor Targets Dataset, SP1 Gene Set* [3].

	NP 001238754.1	NP 003100.1	NP 612482.2
NP 001238754.1	100	99.0502	100
NP 003100.1		100	100
NP 612482.2			100

Table A20. The parameter Similarity of the ClustalΩ in the quantitative measurement of homology, assuming the *Encyclopedia of DNA Elements Resource, ENCODE Transcription Factor Targets Dataset, SP1 Gene Set* [3].

	NP 001238754.1	NP 003100.1	NP 612482.2
NP 001238754.1	100	99.0502	100
NP 003100.1		100	100
NP 612482.2			100

Table A21. The parameter Alignment Score of the ClustalΩ in the quantitative measurement of homology, assuming the *Encyclopedia of DNA Elements Resource, ENCODE Transcription Factor Targets Dataset, SP1 Gene Set* [3].

	NP 001238754.1	NP 003100.1	NP 612482.2
NP 001238754.1	3779	3694.5	3745.5
NP 003100.1		3982	3969
NP 612482.2			4020

Table A22. The parameter Identity of the MUSCLE in the quantitative measurement of homology, assuming the *Encyclopedia of DNA Elements Resource, ENCODE Transcription Factor Targets Dataset, SP1 Gene Set* [3].

	NP 001238754.1	NP 003100.1	NP 612482.2
NP 001238754.1	100	99.0502	100
NP 003100.1		100	100
NP 612482.2			100

Table A23. The parameter Similarity of the MUSCLE in the quantitative measurement of homology, assuming the *Encyclopedia of DNA Elements Resource, ENCODE Transcription Factor Targets Dataset, SP1 Gene Set* [3].

	NP 001238754.1	NP 003100.1	NP 612482.2
NP 001238754.1	100	99.0502	100
NP 003100.1		100	100
NP 612482.2			100

Table A24. The parameter Alignment Score of the MUSCLE in the quantitative measurement of homology, assuming the *Encyclopedia of DNA Elements Resource, ENCODE Transcription Factor Targets Dataset, SP1 Gene Set* [3].

	NP 001238754.1	NP 003100.1	NP 612482.2
NP 001238754.1	3779	3694.5	3745.5
NP 003100.1		3982	3969
NP 612482.2			4020

Table A25. The parameter Identity of the T-Coffee in the quantitative measurement of homology, assuming the *Encyclopedia of DNA Elements Resource, ENCODE Transcription Factor Targets Dataset, SP1 Gene Set* [3].

	NP 001238754.1	NP 003100.1	NP 612482.2
NP 001238754.1	100	99.0502	100
NP 003100.1		100	100
NP 612482.2			100

Table A26. The parameter Similarity of the T-Coffee in the quantitative measurement of homology, assuming the *Encyclopedia of DNA Elements Resource, ENCODE Transcription Factor Targets Dataset, SP1 Gene Set* [3].

	NP 001238754.1	NP 003100.1	NP 612482.2
NP 001238754.1	100	99.0502	100
NP 003100.1		100	100
NP 612482.2			100

Table A27. The parameter Alignment Score of the T-Coffee in the quantitative measurement of homology, assuming the *Encyclopedia of DNA Elements Resource, ENCODE Transcription Factor Targets Dataset, SP1 Gene Set* [3].

	NP 001238754.1	NP 003100.1	NP 612482.2
NP 001238754.1	3779	3694.5	3745.5
NP 003100.1		3982	3969
NP 612482.2			4020

Table A28. The parameter Identity of the Kalign in the quantitative measurement of homology, assuming the *Encyclopedia of DNA Elements Resource, ENCODE Transcription Factor Targets Dataset, SP1 Gene Set* [3].

	NP 001238754.1	NP 003100.1	NP 612482.2
NP 001238754.1	100	99.0502	100
NP 003100.1		100	100
NP 612482.2			100

Table A29. The parameter Similarity of the Kalign in the quantitative measurement of homology, assuming the *Encyclopedia of DNA Elements Resource, ENCODE Transcription Factor Targets Dataset, SP1 Gene Set* [3].

	NP 001238754.1	NP 003100.1	NP 612482.2
NP 001238754.1	100	99.0502	100
NP 003100.1		100	100
NP 612482.2			100

Table A30. The parameter Alignment Score of the Kalign in the quantitative measurement of homology, assuming the *Encyclopedia of DNA Elements Resource, ENCODE Transcription Factor Targets Dataset, SP1 Gene Set* [3].

	NP 001238754.1	NP 003100.1	NP 612482.2
NP 001238754.1	3779	3694.5	3745.5
NP 003100.1		3982	3969
NP 612482.2			4020

Table A31. The parameter Identity of the MAFFT in the quantitative measurement of homology, assuming the *Encyclopedia of DNA Elements Resource, ENCODE Transcription Factor Targets Dataset, SP1 Gene Set* [3].

	NP 001238754.1	NP 003100.1	NP 612482.2
NP 001238754.1	100	99.0502	100
NP 003100.1		100	100
NP 612482.2			100

Table A32. The parameter Similarity of the MAFFT in the quantitative measurement of homology, assuming the *Encyclopedia of DNA Elements Resource, ENCODE Transcription Factor Targets Dataset, SP1 Gene Set* [3].

	NP 001238754.1	NP 003100.1	NP 612482.2
NP 001238754.1	100	99.0502	100
NP 003100.1		100	100
NP 612482.2			100

Table A33. The parameter Alignment Score of the MAFFT in the quantitative measurement of homology, assuming the *Encyclopedia of DNA Elements Resource, ENCODE Transcription Factor Targets Dataset, SP1 Gene Set* [3].

	NP 001238754.1	NP 003100.1	NP 612482.2
NP 001238754.1	3779	3694.5	3745.5
NP 003100.1		3982	3969
NP 612482.2			4020

Table A34. The parameter Sensitivity (SEN) of the HELIOS method with referencing the Smith-Waterman algorithm in the accuracy measurement of classification output, assuming the *Encyclopedia of DNA Elements Resource, ENCODE Transcription Factor Targets Dataset, SP1 Gene Set* [3].

	NP 001238754.1	NP 003100.1	NP 612482.2
NP 001238754.1	1	0.92673	0.92673
NP 003100.1		1	1
NP 612482.2			1

Table A35. The parameter Specification (Spec) of the HELIOS method with referencing the Smith-Waterman algorithm in the accuracy measurement of classification output, assuming the *Encyclopedia of DNA Elements Resource, ENCODE Transcription Factor Targets Dataset, SP1 Gene Set* [3].

	NP 001238754.1	NP 003100.1	NP 612482.2
NP 001238754.1	1	0.99992	0.99991
NP 003100.1		1	1
NP 612482.2			1

Table A36. The parameter Accuracy (Acc) of the HELIOS method with referencing the Smith-Waterman algorithm in the accuracy measurement of classification output, assuming the *Encyclopedia of DNA Elements Resource, ENCODE Transcription Factor Targets Dataset, SP1 Gene Set* [3].

	NP 001238754.1	NP 003100.1	NP 612482.2
NP 001238754.1	1	0.99982	0.99981
NP 003100.1		1	1
NP 612482.2			1

Table A37. The parameter Positive Predictive Value (PPV) of the HELIOS method with referencing the Smith-Waterman algorithm in the accuracy measurement of classification output, assuming the *Encyclopedia of DNA Elements Resource, ENCODE Transcription Factor Targets Dataset, SP1 Gene Set* [3].

	NP 001238754.1	NP 003100.1	NP 612482.2
NP 001238754.1	1	0.93562	0.92673
NP 003100.1		1	1
NP 612482.2			1

Table A38. The parameter Negative Predictive Value (NPV) of the HELIOS method with referencing the Smith-Waterman algorithm in the accuracy measurement of classification output, assuming the *Encyclopedia of DNA Elements Resource, ENCODE Transcription Factor Targets Dataset, SP1 Gene Set* [3].

	NP 001238754.1	NP 003100.1	NP 612482.2
NP 001238754.1	1	0.99991	0.99991
NP 003100.1		1	1
NP 612482.2			1

Table A39. The parameter Matthew's Coefficient Correlation (MCC) of the HELIOS method with referencing the Smith-Waterman algorithm in the accuracy measurement of classification output, assuming the *Encyclopedia of DNA Elements Resource, ENCODE Transcription Factor Targets Dataset, SP1 Gene Set* [3].

	NP 001238754.1	NP 003100.1	NP 612482.2
NP 001238754.1	1	0.93107	0.92664
NP 003100.1		1	1
NP 612482.2			1

Table A40. The parameter Test's Accuracy (F-Score) of the HELIOS method with referencing the Smith-Waterman algorithm in the accuracy measurement of classification output, assuming the *Encyclopedia of DNA Elements Resource, ENCODE Transcription Factor Targets Dataset, SP1 Gene Set* [3].

	NP 001238754.1	NP 003100.1	NP 612482.2
NP 001238754.1	1	0.93115	0.92673
NP 003100.1		1	1
NP 612482.2			1

Table A41. The parameter Sensitivity (SEN) of the HELIOS method with referencing the Needleman-Wunsch algorithm in the accuracy measurement of classification output, assuming the *Encyclopedia of DNA Elements Resource, ENCODE Transcription Factor Targets Dataset, SP1 Gene Set* [3].

	NP 001238754.1	NP 003100.1	NP 612482.2
NP 001238754.1	1	0	0.035211
NP 003100.1		1	0
NP 612482.2			1

Table A42. The parameter Specification (Spec) of the HELIOS method with referencing the Needleman-Wunsch algorithm in the accuracy measurement of classification output, assuming the *Encyclopedia of DNA Elements Resource, ENCODE Transcription Factor Targets Dataset, SP1 Gene Set* [3].

	NP 001238754.1	NP 003100.1	NP 612482.2
NP 001238754.1	1	0.99868	0.99873
NP 003100.1		1	0.99872
NP 612482.2			1

Table A43. The parameter Accuracy (Acc) of the HELIOS method with referencing the Needleman-Wunsch algorithm in the accuracy measurement of classification output, assuming the *Encyclopedia of DNA Elements Resource, ENCODE Transcription Factor Targets Dataset, SP1 Gene Set* [3].

	NP 001238754.1	NP 003100.1	NP 612482.2
NP 001238754.1	1	0.99728	0.99738
NP 003100.1		1	0.99744
NP 612482.2			1

Table A44. The parameter Positive Predictive Value (PPV) of the HELIOS method with referencing the Needleman-Wunsch algorithm in the accuracy measurement of classification output, assuming the *Encyclopedia of DNA Elements Resource, ENCODE Transcription Factor Targets Dataset, SP1 Gene Set* [3].

	NP 001238754.1	NP 003100.1	NP 612482.2
NP 001238754.1	1	0	0.037707
NP 003100.1		1	0
NP 612482.2			1

Table A45. The parameter Negative Predictive Value (NPV) of the HELIOS method with referencing the Needleman-Wunsch algorithm in the accuracy measurement of classification output, assuming the *Encyclopedia of DNA Elements Resource, ENCODE Transcription Factor Targets Dataset, SP1 Gene Set* [3].

	NP 001238754.1	NP 003100.1	NP 612482.2
NP 001238754.1	1	0.99859	0.99864
NP 003100.1		1	0.99871
NP 612482.2			1

Table A46. The parameter Matthew's Coefficient Correlation (MCC) of the HELIOS method with referencing the Needleman-Wunsch algorithm in the accuracy measurement of classification output, assuming the *Encyclopedia of DNA Elements Resource, ENCODE Transcription Factor Targets Dataset, SP1 Gene Set* [3].

	NP 001238754.1	NP 003100.1	NP 612482.2
NP 001238754.1	1	-0.001361	0.035127
NP 003100.1		1	-0.0012812
NP 612482.2			1

Table A47. The parameter Test's Accuracy (F-Score) of the HELIOS method with referencing the Needleman-Wunsch algorithm in the accuracy measurement of classification output, assuming the *Encyclopedia of DNA Elements Resource, ENCODE Transcription Factor Targets Dataset, SP1 Gene Set* [3].

	NP 001238754.1	NP 003100.1	NP 612482.2
NP 001238754.1	1	0	0.036417
NP 003100.1		1	0
NP 612482.2			1

Table A48. The parameter Sensitivity (SEN) of the HELIOS method with referencing the BLAST in the accuracy measurement of classification output, assuming the *Encyclopedia of DNA Elements Resource, ENCODE Transcription Factor Targets Dataset, SP1 Gene Set* [3].

	NP 001238754.1	NP 003100.1	NP 612482.2
NP 001238754.1	1	0.99315	0.99322
NP 003100.1		1	1
NP 612482.2			1

Table A49. The parameter Specification (Spec) of the HELIOS method with referencing the BLAST in the accuracy measurement of classification output, assuming the *Encyclopedia of DNA Elements Resource, ENCODE Transcription Factor Targets Dataset, SP1 Gene Set* [3].

	NP 001238754.1	NP 003100.1	NP 612482.2
NP 001238754.1	1	0.99999	0.99999
NP 003100.1		1	1
NP 612482.2			1

Table A50. The parameter Accuracy (Acc) of the HELIOS method with referencing the BLAST in the accuracy measurement of classification output, assuming the *Encyclopedia of DNA Elements Resource, ENCODE Transcription Factor Targets Dataset, SP1 Gene Set* [3].

	NP 001238754.1	NP 003100.1	NP 612482.2
NP 001238754.1	1	0.99998	0.99998
NP 003100.1		1	1
NP 612482.2			1

Table A51. The parameter Positive Predictive Value (PPV) of the HELIOS method with referencing the BLAST in the accuracy measurement of classification output, assuming the *Encyclopedia of DNA Elements Resource, ENCODE Transcription Factor Targets Dataset, SP1 Gene Set* [3].

	NP 001238754.1	NP 003100.1	NP 612482.2
NP 001238754.1	1	0.99315	0.99322
NP 003100.1		1	1
NP 612482.2			1

Table A52. The parameter Negative Predictive Value (NPV) of the HELIOS method with referencing the BLAST in the accuracy measurement of classification output, assuming the *Encyclopedia of DNA Elements Resource, ENCODE Transcription Factor Targets Dataset, SP1 Gene Set* [3].

	NP 001238754.1	NP 003100.1	NP 612482.2
NP 001238754.1	1	0.99999	0.99999
NP 003100.1		1	1
NP 612482.2			1

Table A53. The parameter Matthew's Coefficient Correlation (MCC) of the HELIOS method with referencing the BLAST in the accuracy measurement of classification output, assuming the *Encyclopedia of DNA Elements Resource, ENCODE Transcription Factor Targets Dataset, SP1 Gene Set* [3].

	NP 001238754.1	NP 003100.1	NP 612482.2
NP 001238754.1	1	0.99314	0.99321
NP 003100.1		1	1
NP 612482.2			1

Table A54. The parameter Test's Accuracy (F-Score) of the HELIOS method with referencing the BLAST in the accuracy measurement of classification output, assuming the *Encyclopedia of DNA Elements Resource, ENCODE Transcription Factor Targets Dataset, SP1 Gene Set* [3].

	NP 001238754.1	NP 003100.1	NP 612482.2
NP 001238754.1	1	0.99315	0.99322
NP 003100.1		1	1
NP 612482.2			1

Table A55. The parameter Sensitivity (SEN) of the HELIOS method with referencing the ClustalW in the accuracy measurement of classification output, assuming the *Encyclopedia of DNA Elements Resource, ENCODE Transcription Factor Targets Dataset, SP1 Gene Set* [3].

	NP 001238754.1	NP 003100.1	NP 612482.2
NP 001238754.1	1	1	1
NP 003100.1		1	1
NP 612482.2			1

Table A56. The parameter Specification (Spec) of the HELIOS method with referencing the ClustalW in the accuracy measurement of classification output, assuming the *Encyclopedia of DNA Elements Resource, ENCODE Transcription Factor Targets Dataset, SP1 Gene Set* [3].

	NP 001238754.1	NP 003100.1	NP 612482.2
NP 001238754.1	1	1	1
NP 003100.1		1	1
NP 612482.2			1

Table A57. The parameter Accuracy (Acc) of the HELIOS method with referencing the ClustalW in the accuracy measurement of classification output, assuming the *Encyclopedia of DNA Elements Resource, ENCODE Transcription Factor Targets Dataset, SP1 Gene Set* [3].

	NP 001238754.1	NP 003100.1	NP 612482.2
NP 001238754.1	1	1	1
NP 003100.1		1	1
NP 612482.2			1

Table A58. The parameter Positive Predictive Value (PPV) of the HELIOS method with referencing the ClustalW in the accuracy measurement of classification output, assuming the *Encyclopedia of DNA Elements Resource, ENCODE Transcription Factor Targets Dataset, SP1 Gene Set* [3].

	NP 001238754.1	NP 003100.1	NP 612482.2
NP 001238754.1	1	1	1
NP 003100.1		1	1
NP 612482.2			1

Table A59. The parameter Negative Predictive Value (NPV) of the HELIOS method with referencing the ClustalW in the accuracy measurement of classification output, assuming the *Encyclopedia of DNA Elements Resource, ENCODE Transcription Factor Targets Dataset, SP1 Gene Set* [3].

	NP 001238754.1	NP 003100.1	NP 612482.2
NP 001238754.1	1	1	1
NP 003100.1		1	1
NP 612482.2			1

Table A60. The parameter Matthew's Coefficient Correlation (MCC) of the HELIOS method with referencing the ClustalW in the accuracy measurement of classification output, assuming the *Encyclopedia of DNA Elements Resource, ENCODE Transcription Factor Targets Dataset, SP1 Gene Set* [3].

	NP 001238754.1	NP 003100.1	NP 612482.2
NP 001238754.1	1	1	1
NP 003100.1		1	1
NP 612482.2			1

Table A61. The parameter Test's Accuracy (F-Score) of the HELIOS method with referencing the ClustalW in the accuracy measurement of classification output, assuming the *Encyclopedia of DNA Elements Resource, ENCODE Transcription Factor Targets Dataset, SP1 Gene Set* [3].

	NP 001238754.1	NP 003100.1	NP 612482.2
NP 001238754.1	1	1	1
NP 003100.1		1	1
NP 612482.2			1

Table A62. The parameter Sensitivity (SEN) of the HELIOS method with referencing the ClustalΩ in the accuracy measurement of classification output, assuming the *Encyclopedia of DNA Elements Resource, ENCODE Transcription Factor Targets Dataset, SP1 Gene Set* [3].

	NP 001238754.1	NP 003100.1	NP 612482.2
NP 001238754.1	1	0.99863	0.99864
NP 003100.1		1	1
NP 612482.2			1

Table A63. The parameter Specification (Spec) of the HELIOS method with referencing the ClustalΩ in the accuracy measurement of classification output, assuming the *Encyclopedia of DNA Elements Resource, ENCODE Transcription Factor Targets Dataset, SP1 Gene Set* [3].

	NP 001238754.1	NP 003100.1	NP 612482.2
NP 001238754.1	1	1	1
NP 003100.1		1	1
NP 612482.2			1

Table A64. The parameter Accuracy (Acc) of the HELIOS method with referencing the ClustalΩ in the accuracy measurement of classification output, assuming the *Encyclopedia of DNA Elements Resource, ENCODE Transcription Factor Targets Dataset, SP1 Gene Set* [3].

	NP 001238754.1	NP 003100.1	NP 612482.2
NP 001238754.1	1	1	1
NP 003100.1		1	1
NP 612482.2			1

Table A65. The parameter Positive Predictive Value (PPV) of the HELIOS method with referencing the ClustalΩ in the accuracy measurement of classification output, assuming the *Encyclopedia of DNA Elements Resource, ENCODE Transcription Factor Targets Dataset, SP1 Gene Set* [3].

	NP 001238754.1	NP 003100.1	NP 612482.2
NP 001238754.1	1	0.99863	0.99864
NP 003100.1		1	1
NP 612482.2			1

Table A66. The parameter Negative Predictive Value (NPV) of the HELIOS method with referencing the ClustalΩ in the accuracy measurement of classification output, assuming the *Encyclopedia of DNA Elements Resource, ENCODE Transcription Factor Targets Dataset, SP1 Gene Set* [3].

	NP 001238754.1	NP 003100.1	NP 612482.2
NP 001238754.1	1	1	1
NP 003100.1		1	1
NP 612482.2			1

Table A67. The parameter Matthew's Coefficient Correlation (MCC) of the HELIOS method with referencing the ClustalΩ in the accuracy measurement of classification output, assuming the *Encyclopedia of DNA Elements Resource, ENCODE Transcription Factor Targets Dataset, SP1 Gene Set* [3].

	NP 001238754.1	NP 003100.1	NP 612482.2
NP 001238754.1	1	0.99863	0.99864
NP 003100.1		1	1
NP 612482.2			1

Table A68. The parameter Test's Accuracy (F-Score) of the HELIOS method with referencing the ClustalΩ in the accuracy measurement of classification output, assuming the *Encyclopedia of DNA Elements Resource, ENCODE Transcription Factor Targets Dataset, SP1 Gene Set* [3].

	NP 001238754.1	NP 003100.1	NP 612482.2
NP 001238754.1	1	0.99863	0.99864
NP 003100.1		1	1
NP 612482.2			1

Table A69. The parameter Sensitivity (SEN) of the HELIOS method with referencing the MUSCLE in the accuracy measurement of classification output, assuming the *Encyclopedia of DNA Elements Resource, ENCODE Transcription Factor Targets Dataset, SP1 Gene Set* [3].

	NP 001238754.1	NP 003100.1	NP 612482.2
NP 001238754.1	1	0.99863	0.99864
NP 003100.1		1	1
NP 612482.2			1

Table A70. The parameter Specification (Spec) of the HELIOS method with referencing the MUSCLE in the accuracy measurement of classification output, assuming the *Encyclopedia of DNA Elements Resource, ENCODE Transcription Factor Targets Dataset, SP1 Gene Set* [3].

	NP 001238754.1	NP 003100.1	NP 612482.2
NP 001238754.1	1	1	1
NP 003100.1		1	1
NP 612482.2			1

Table A71. The parameter Accuracy (Acc) of the HELIOS method with referencing the MUSCLE in the accuracy measurement of classification output, assuming the *Encyclopedia of DNA Elements Resource, ENCODE Transcription Factor Targets Dataset, SP1 Gene Set* [3].

	NP 001238754.1	NP 003100.1	NP 612482.2
NP 001238754.1	1	1	1
NP 003100.1		1	1
NP 612482.2			1

Table A72. The parameter Positive Predictive Value (PPV) of the HELIOS method with referencing the MUSCLE in the accuracy measurement of classification output, assuming the *Encyclopedia of DNA Elements Resource, ENCODE Transcription Factor Targets Dataset, SP1 Gene Set* [3].

	NP 001238754.1	NP 003100.1	NP 612482.2
NP 001238754.1	1	0.99863	0.99864
NP 003100.1		1	1
NP 612482.2			1

Table A73. The parameter Negative Predictive Value (NPV) of the HELIOS method with referencing the MUSCLE in the accuracy measurement of classification output, assuming the *Encyclopedia of DNA Elements Resource, ENCODE Transcription Factor Targets Dataset, SP1 Gene Set* [3].

	NP 001238754.1	NP 003100.1	NP 612482.2
NP 001238754.1	1	1	1
NP 003100.1		1	1
NP 612482.2			1

Table A74. The parameter Matthew's Coefficient Correlation (MCC) of the HELIOS method with referencing the MUSCLE in the accuracy measurement of classification output, assuming the *Encyclopedia of DNA Elements Resource, ENCODE Transcription Factor Targets Dataset, SP1 Gene Set* [3].

	NP 001238754.1	NP 003100.1	NP 612482.2
NP 001238754.1	1	0.99863	0.99864
NP 003100.1		1	1
NP 612482.2			1

Table A75. The parameter Test's Accuracy (F-Score) of the HELIOS method with referencing the MUSCLE in the accuracy measurement of classification output, assuming the *Encyclopedia of DNA Elements Resource, ENCODE Transcription Factor Targets Dataset, SP1 Gene Set* [3].

	NP 001238754.1	NP 003100.1	NP 612482.2
NP 001238754.1	1	0.99863	0.99864
NP 003100.1		1	1
NP 612482.2			1

Table A76. The parameter Sensitivity (SEN) of the HELIOS method with referencing the T-Coffee in the accuracy measurement of classification output, assuming the *Encyclopedia of DNA Elements Resource, ENCODE Transcription Factor Targets Dataset, SP1 Gene Set* [3].

	NP 001238754.1	NP 003100.1	NP 612482.2
NP 001238754.1	1	0.99863	0.99864
NP 003100.1		1	1
NP 612482.2			1

Table A77. The parameter Specification (Spec) of the HELIOS method with referencing the T-Coffee in the accuracy measurement of classification output, assuming the *Encyclopedia of DNA Elements Resource, ENCODE Transcription Factor Targets Dataset, SP1 Gene Set* [3].

	NP 001238754.1	NP 003100.1	NP 612482.2
NP 001238754.1	1	1	1
NP 003100.1		1	1
NP 612482.2			1

Table A78. The parameter Accuracy (Acc) of the HELIOS method with referencing the T-Coffee in the accuracy measurement of classification output, assuming the *Encyclopedia of DNA Elements Resource, ENCODE Transcription Factor Targets Dataset, SP1 Gene Set* [3].

	NP 001238754.1	NP 003100.1	NP 612482.2
NP 001238754.1	1	1	1
NP 003100.1		1	1
NP 612482.2			1

Table A79. The parameter Positive Predictive Value (PPV) of the HELIOS method with referencing the T-Coffee in the accuracy measurement of classification output, assuming the *Encyclopedia of DNA Elements Resource, ENCODE Transcription Factor Targets Dataset, SP1 Gene Set* [3].

	NP 001238754.1	NP 003100.1	NP 612482.2
NP 001238754.1	1	0.99863	0.99864
NP 003100.1		1	1
NP 612482.2			1

Table A80. The parameter Negative Predictive Value (NPV) of the HELIOS method with referencing the T-Coffee in the accuracy measurement of classification output, assuming the *Encyclopedia of DNA Elements Resource, ENCODE Transcription Factor Targets Dataset, SP1 Gene Set* [3].

	NP 001238754.1	NP 003100.1	NP 612482.2
NP 001238754.1	1	1	1
NP 003100.1		1	1
NP 612482.2			1

Table A81. The parameter Matthew's Coefficient Correlation (MCC) of the HELIOS method with referencing the T-Coffee in the accuracy measurement of classification output, assuming the *Encyclopedia of DNA Elements Resource, ENCODE Transcription Factor Targets Dataset, SP1 Gene Set* [3].

	NP 001238754.1	NP 003100.1	NP 612482.2
NP 001238754.1	1	0.99863	0.99864
NP 003100.1		1	1
NP 612482.2			1

Table A82. The parameter Test's Accuracy (F-Score) of the HELIOS method with referencing the T-Coffee in the accuracy measurement of classification output, assuming the *Encyclopedia of DNA Elements Resource, ENCODE Transcription Factor Targets Dataset, SP1 Gene Set* [3].

	NP 001238754.1	NP 003100.1	NP 612482.2
NP 001238754.1	1	0.99863	0.99864
NP 003100.1		1	1
NP 612482.2			1

Table A83. The parameter Sensitivity (SEN) of the HELIOS method with referencing the Kalign in the accuracy measurement of classification output, assuming the *Encyclopedia of DNA Elements Resource, ENCODE Transcription Factor Targets Dataset, SP1 Gene Set* [3].

	NP 001238754.1	NP 003100.1	NP 612482.2
NP 001238754.1	1	1	1
NP 003100.1		1	1
NP 612482.2			1

Table A84. The parameter Specification (Spec) of the HELIOS method with referencing the Kalign in the accuracy measurement of classification output, assuming the *Encyclopedia of DNA Elements Resource, ENCODE Transcription Factor Targets Dataset, SP1 Gene Set* [3].

	NP 001238754.1	NP 003100.1	NP 612482.2
NP 001238754.1	1	1	1
NP 003100.1		1	1
NP 612482.2			1

Table A85. The parameter Accuracy (Acc) of the HELIOS method with referencing the Kalign in the accuracy measurement of classification output, assuming the *Encyclopedia of DNA Elements Resource, ENCODE Transcription Factor Targets Dataset, SP1 Gene Set* [3].

	NP 001238754.1	NP 003100.1	NP 612482.2
NP 001238754.1	1	1	1
NP 003100.1		1	1
NP 612482.2			1

Table A86. The parameter Positive Predictive Value (PPV) of the HELIOS method with referencing the Kalign in the accuracy measurement of classification output, assuming the *Encyclopedia of DNA Elements Resource, ENCODE Transcription Factor Targets Dataset, SP1 Gene Set* [3].

	NP 001238754.1	NP 003100.1	NP 612482.2
NP 001238754.1	1	1	1
NP 003100.1		1	1
NP 612482.2			1

Table A87. The parameter Negative Predictive Value (NPV) of the HELIOS method with referencing the Kalign in the accuracy measurement of classification output, assuming the *Encyclopedia of DNA Elements Resource, ENCODE Transcription Factor Targets Dataset, SP1 Gene Set* [3].

	NP 001238754.1	NP 003100.1	NP 612482.2
NP 001238754.1	1	1	1
NP 003100.1		1	1
NP 612482.2			1

Table A88. The parameter Matthew's Coefficient Correlation (MCC) of the HELIOS method with referencing the Kalign in the accuracy measurement of classification output, assuming the *Encyclopedia of DNA Elements Resource, ENCODE Transcription Factor Targets Dataset, SP1 Gene Set* [3].

	NP 001238754.1	NP 003100.1	NP 612482.2
NP 001238754.1	1	1	1
NP 003100.1		1	1
NP 612482.2			1

Table A89. The parameter Test's Accuracy (F-Score) of the HELIOS method with referencing the Kalign in the accuracy measurement of classification output, assuming the *Encyclopedia of DNA Elements Resource, ENCODE Transcription Factor Targets Dataset, SP1 Gene Set* [3].

	NP 001238754.1	NP 003100.1	NP 612482.2
NP 001238754.1	1	1	1
NP 003100.1		1	1
NP 612482.2			1

Table A90. The parameter Sensitivity (SEN) of the HELIOS method with referencing the MAFFT in the accuracy measurement of classification output, assuming the *Encyclopedia of DNA Elements Resource, ENCODE Transcription Factor Targets Dataset, SP1 Gene Set* [3].

	NP 001238754.1	NP 003100.1	NP 612482.2
NP 001238754.1	1	0.99863	0.99864
NP 003100.1		1	1
NP 612482.2			1

Table A91. The parameter Specification (Spec) of the HELIOS method with referencing the MAFFT in the accuracy measurement of classification output, assuming the *Encyclopedia of DNA Elements Resource, ENCODE Transcription Factor Targets Dataset, SP1 Gene Set* [3].

	NP 001238754.1	NP 003100.1	NP 612482.2
NP 001238754.1	1	1	1
NP 003100.1		1	1
NP 612482.2			1

Table A92. The parameter Accuracy (Acc) of the HELIOS method with referencing the MAFFT in the accuracy measurement of classification output, assuming the *Encyclopedia of DNA Elements Resource, ENCODE Transcription Factor Targets Dataset, SP1 Gene Set* [3].

	NP 001238754.1	NP 003100.1	NP 612482.2
NP 001238754.1	1	1	1
NP 003100.1		1	1
NP 612482.2			1

Table A93. The parameter Positive Predictive Value (PPV) of the HELIOS method with referencing the MAFFT in the accuracy measurement of classification output, assuming the *Encyclopedia of DNA Elements Resource, ENCODE Transcription Factor Targets Dataset, SP1 Gene Set* [3].

	NP 001238754.1	NP 003100.1	NP 612482.2
NP 001238754.1	1	0.99863	0.99864
NP 003100.1		1	1
NP 612482.2			1

Table A94. The parameter Negative Predictive Value (NPV) of the HELIOS method with referencing the MAFFT in the accuracy measurement of classification output, assuming the *Encyclopedia of DNA Elements Resource, ENCODE Transcription Factor Targets Dataset, SP1 Gene Set* [3].

	NP 001238754.1	NP 003100.1	NP 612482.2
NP 001238754.1	1	1	1
NP 003100.1		1	1
NP 612482.2			1

Table A95. The parameter Matthew's Coefficient Correlation (MCC) of the HELIOS method with referencing the MAFFT in the accuracy measurement of classification output, assuming the *Encyclopedia of DNA Elements Resource, ENCODE Transcription Factor Targets Dataset, SP1 Gene Set* [3].

	NP 001238754.1	NP 003100.1	NP 612482.2
NP 001238754.1	1	0.99863	0.99864
NP 003100.1		1	1
NP 612482.2			1

Table A96. The parameter Test's Accuracy (F-Score) of the HELIOS method with referencing the MAFFT in the accuracy measurement of classification output, assuming the *Encyclopedia of DNA Elements Resource, ENCODE Transcription Factor Targets Dataset, SP1 Gene Set* [3].

	NP 001238754.1	NP 003100.1	NP 612482.2
NP 001238754.1	1	0.99863	0.99864
NP 003100.1		1	1
NP 612482.2			1
