# Supplementary material for: HELIOS: High-speed sequence alignment in optics
Source: PLoS Comput Biol. 2022 Nov 21;18(11):e1010665. doi: 10.1371/journal.pcbi.1010665 (PMC9678324; doi:10.1371/journal.pcbi.1010665)
Supplement: S9 Text — (PDF) [file pcbi.1010665.s009.pdf]

HELIOS: High-Speed Sequence Alignment in Optics: S9 Text

EHSAN MALEKI[1], SAEEDEH AKBARI ROKN ABADI[1], AND SOMAYYEH KOOHI[1,*]

[1] *Department of Computer Engineering, Sharif University of Technology, Azadi Ave., Tehran, Iran.*
[*] *Corresponding author: koohi@sharif.edu*

Compiled October 20, 2022

In response to the imperfections of current sequence alignment methods, originated from the inherent serialism within their corresponding electrical systems, a few optical approaches for biological data comparison have been proposed recently. However, due to their low performance, raised from their inefficient coding scheme, this paper presents a novel all-optical high-throughput method for aligning DNA, RNA, and protein sequences, named HELIOS. The HELIOS method employs highly sophisticated operations to locate character matches, single or multiple mutations, and single or multiple indels within various biological sequences. On the other hand, the HELIOS optical architecture exploits high-speed processing and operational parallelism in optics, by adopting wavelength and polarization of optical beams. For evaluation, the functionality and accuracy of the HELIOS method are approved through behavioral and optical simulation studies, while its complexity and performance are estimated through analytical computation. The accuracy evaluations indicate that the HELIOS method achieves a precise pairwise alignment of two sequences, highly similar to those of Smith-Waterman, Needleman-Wunsch, BLAST, MUSCLE, ClustalW, ClustalΩ, T-Coffee, Kalign, and MAFFT. According to our performance evaluations, the HELIOS optical architecture outperforms all alternative electrical and optical algorithms in terms of processing time and memory requirement, relying on its highly sophisticated method and optical architecture. Moreover, the employed compact coding scheme highly escalates the number of input characters, and hence, it offers reduced time and space complexities, compared to the electrical and optical alternatives. It makes the HELIOS method and optical architecture highly applicable for biomedical applications.

1. ACCURACY EVALUATION

In order to comprehensively assess the accuracy of the HELIOS method, two statistical analyses are performed through simulation studies: 1) Quantitative measurement of homology [1], and 2) Accuracy measurement of classification output [2], compared to the well-known algorithms, briefly reported in Tables A1 and A2, respectively. In this manner, the *Hub Proteins Resource, Protein-Protein Interactions Dataset, PTPN6 Gene Set* [3] is assumed in this study, as represented in Table A3.

A. Quantitative measurement of homology

To perform quantitative measurement of homology [1], the parameters Identity, Similarity, and Alignment Score of the HELIOS outputs are calculated through simulation studies, as reported in Tables A4-A6, respectively, assuming the *Hub Proteins Resource, Protein-Protein Interactions Dataset, PTPN6 Gene Set* [3]. While the Identity reports the number of exactly matched characters of two sequences (in percentage), the Similarity measures the resemblance of two compared sequences. Specifically, regarding the physicochemical properties, the amino acids are categorized into six groups with different Similarity values; including GAVLI, FYW, STCM, KRH, DENQ, and P. As the third metric, the BLOSUM62 [4] substitution scoring matrix [4] is adopted to calculate the Alignment Score, with gap opening and extension penalties equal to -10 and -0.5, respectively.

For a comparative study, the values of Identity, Similarity, and Alignment Score of the quantitative measurement of homology is performed by various well-known algorithms to be compared to the those of HELIOS method, assuming *Hub Proteins Resource, Protein-Protein Interactions Dataset, PTPN6 Gene Set* [3]. It includes Smith-Waterman (SW) [5] reported in Tables A7-A9, Needleman-Wunsch (NW) [6] reported in Tables A10-A12, BLAST [7] reported in Tables A13-A15, ClustalW [8] reported in Tables A16-A18, Clustal-Omega [9] reported in Tables A19-A21, MUSCLE [9] reported in Tables A22-A24, T-Coffee [10] reported in Tables A25-A27, Kalign [11] reported in Tables A28-A30, and MAFFT [12] reported in Tables A31-A33.

Table A1. A brief report of the quantitative measurement of homology of the HELIOS method, compared to nine well-known algorithms, including SW, NW, BLAST, ClustalW, Clustal Omega, Muscle, T-Coffee, Kalign, and MAFFT. The parameters Identity, Similarity, and Alignment score are averaged and reported. The dataset used in this study is the *Hub Proteins Resource, Protein-Protein Interactions Dataset, PTPN6 Gene Set* [3].

	HELIOS	SW	NW	BLAST	MUSCLE	ClustalW	ClustalΩ	T-Coffee	Kalign	MAFFT
Identity	98.638	98.401	98.317	98.616	98.568	98.532	98.401	98.616	98.449	98.616
Similarity	98.764	98.574	98.317	98.915	98.909	98.742	98.574	98.873	98.622	98.957
Alignment Score	2900.3	2897.2	2929.8	2906.1	2904.1	2898.8	2897.2	2903.8	2900.1	2907.3

Table A2. A brief report of the accuracy measurement of classification output of the HELIOS method with referencing well-known algorithms, including SW, NW, BLAST, ClustalW, Clustal Omega, Muscle, T-Coffee, Kalign, and MAFFT. The parameters SEN, Spec, Acc, PPV, NPV, MCC, and F-Score are averaged and reported. The dataset used in this study is the *Hub Proteins Resource, Protein-Protein Interactions Dataset, PTPN6 Gene Set* [3].

	SW	NW	BLAST	MUSCLE	ClustalW	ClustalΩ	T-Coffee	Kalign	MAFFT
SEN	0.98897	0.47676	0.99182	0.98603	0.99086	0.98897	0.98863	0.98945	0.98643
Spec	0.99998	0.99913	0.99999	0.99998	0.99999	0.99998	0.99998	0.99998	0.99998
ACC	0.99997	0.99814	0.99997	0.99995	0.99997	0.99997	0.99996	0.99997	0.99996
PPV	0.98989	0.47677	0.99994	0.98496	0.99179	0.98989	0.98754	0.99038	0.98536
NPV	0.99998	0.99900	0.99999	0.99998	0.99998	0.99998	0.99998	0.99998	0.99998
MCC	0.98941	0.47583	0.99186	0.98547	0.99131	0.98941	0.98806	0.98989	0.98587
F-Score	0.98943	0.47676	0.99188	0.98549	0.99132	0.98943	0.98808	0.98991	0.98589

B. Accuracy measurement of classification output

Afterward, the accuracy measurement of the classification output [2] of the HELIOS method is addressed by calculating the values of Sensitivity (SEN), Specificity (Spec), Accuracy (ACC), Positive Predictive Value (PPV), Negative Predictive Value (NPV), Matthew's Coefficient Correlation (MCC), and Test's Accuracy (F-Score) in the simulation studies, according to Eq 5 to Eq 11, respectively.

As a comparative study, the accuracy measurement of the classification output of the HELIOS method is accomplished, assuming *Hub Proteins Resource, Protein-Protein Interactions Dataset, PTPN6 Gene Set* [3], and the corresponding metrics are calculated with considering Smith-Waterman [5] reported in Tables A34-A40, Needleman-Wunsch [6] reported in Tables A41-A47, ClustalW [8] reported in Tables A55-A61, Clustal-Omega [9] reported in Tables A62-A68, BLAST [7] reported in Tables A48-A54, MUSCLE [13] reported in Tables A69-A75, T-Coffee [10] reported in Tables A76-A82, Kalign [11] reported in Tables A83-A89, and MAFFT [12] reported in Tables A90-A96.

REFERENCES

1. D. S. Moss, S. Jelaska, and S. Pongor, *Essays in bioinformatics*, vol. 368 (IOS Press, 2005).
2. M. Hamada, H. Kiryu, W. Iwasaki, and K. Asai, "Generalized centroid estimators in bioinformatics," PloS one **6**, e16450 (2011).
3. E. Y. Chen, C. M. Tan, Y. Kou, Q. Duan, Z. Wang, G. V. Meirelles, N. R. Clark, and A. Ma'ayan, "Enrichr: interactive and collaborative html5 gene list enrichment analysis tool," BMC bioinformatics **14**, 1–14 (2013).
4. D. W. Mount, "Using blosum in sequence alignments," Cold Spring Harb. Protoc. **2008**, pdb.top39 (2008).
5. H. Zou, S. Tang, C. Yu, H. Fu, Y. Li, and W. Tang, "asw: accelerating smith–waterman algorithm on coupled cpu–gpu architecture," Int. J. Parallel Program. **47**, 388–402 (2019).
6. Y. Jararweh, M. Al-Ayyoub, M. Fakirah, L. Alawneh, and B. B. Gupta, "Improving the performance of the needleman-wunsch algorithm using parallelization and vectorization techniques," Multimed. Tools Appl. **78**, 3961–3977 (2019).
7. G. M. Boratyn, J. Thierry-Mieg, D. Thierry-Mieg, B. Busby, and T. L. Madden, "Magic-blast, an accurate rna-seq aligner for long and short reads," BMC bioinformatics **20**, 1–19 (2019).
8. D. Díaz, F. J. Esteban, P. Hernández, J. A. Caballero, A. Guevara, G. Dorado, and S. Gálvez, "Mc64-clustalwp2: A highly-parallel hybrid strategy to align multiple sequences in many-core architectures," PLOS ONE **9**, 1–12 (2014).
9. F. Sievers and D. G. Higgins, "Clustal omega for making accurate alignments of many protein sequences," Protein Sci. **27**, 135–145 (2018).
10. C. Notredame, D. G. Higgins, and J. Heringa, "T-coffee: a novel method for fast and accurate multiple sequence alignment11edited by j. thornton," J. Mol. Biol. **302**, 205–217 (2000).
11. T. Lassmann, "Kalign 3: multiple sequence alignment of large datasets," (2020).
12. J. Rozewicki, S. Li, K. M. Amada, D. M. Standley, and K. Katoh, "Mafft-dash: integrated protein sequence and structural alignment," Nucleic acids research **47**, W5–W10 (2019).
13. R. C. Edgar, "MUSCLE: multiple sequence alignment with high accuracy and high throughput," Nucleic Acids Res. **32**, 1792–1797 (2004).

Table A3. The list of input sequences, assuming the *Hub Proteins Resource, Protein-Protein Interactions Dataset, PTPN6 Gene Set* [3].

Name	Sequence
NP 002822.2	MEVTA DQPRW VSHHH PAVLN GQHPD THHPG LSHSY MDAAQ YPLPE EVDVL FNIDG QGNHV PPYYG NSVRA TVQRY PPTHH GSQVC RPPLL HGSLP WLDGG KALGS HHTAS PWNLS PFSKT SIHHG SPGPL SVYPP ASSSS LSGGH ASPHL FTFPP TPPKD VSPDP SLSTP GSAGS ARQDE KECLK YQVPL PDSMK LESSH SRGSM TALGG ASSST HHPIT TYPPY VPEYS SGLFP PSSLL GGSPT GFGCK SRPKA RSSTE GRECV NCGAT STPLW RRDGT GHYLC NACGL YHKMN GQNRP LIKPK RRLSA ARRAG TSCAN CQTTT TTLWR RNANG DPVCN ACGLY YKLHN INRPL TMKKE GIQTR NRKMS SKSKK CKKVH DSLED FPKNS SFNPA ALSRH MSSLS HISPF SHSSH MLTTP TPMHP PSSLS FGPHH PSSMV TAMG
NP 536858.1	MLSRG WFHRD LSGLD AETLL KGRGV HGSFL ARPSR KNQGD FSLSV RVGDQ VTHIR IQNSG DFYDL YGGEK FATLT ELVEY YTQQQ GVLQD RDGTI IHLKY PLNCS DPTSE RWYHG HMSGG QAETL LQAKG EPWTF LVRES LSQPG DFVLS VLSDQ PKAGP GSPLR VTHIK VMCEG GRYTV GGLET FDSLT DLVEH FKKTG IEEAS GAFVY LRQPY YATRV NAADI ENRVL ELNKK QESED TAKAG FWEEF ESLQK QEVKN LHQRL EGQRP ENKGK NRYKN ILPFD HSRVI LQGRD SNIPG SDYIN ANYIK NQLLG PDENA KTYIA SQGCL EATVN DFWQM AWQEN SRVIV MTTRE VEKGR NKCVP YWPEV GMQRA YGPYS VTNCG EHDTT EYKLR TLQVS PLDNG DLIRE IWHYQ YLSWP DHGVP SEPGG VLSFL DQINQ RQESL PHAGP IIVHC SAGIG RTGTI IVIDM LMENI STKGL DCDID IQKTI QMVRA QRSGM VQTEA QYKFI YVAIA QFIET TKKKL EVLQS QKGQE SEYGN ITYPP AMKNA HAKAS RTSSK HKEDV YENLH TKNKR EEKVK KQRSA DKEKS KGSLK RK
NP 536859.1	MVRWF HRDLS GLDAE TLLKG RGVHG SFLAR PSRKN QGDFS LSVRV GDQVT HIRIQ NSGDF YDLYG GEKFA TLTEL VEYYT QQQGV LQDRD GTIIH LKYPL NCSDP TSERW YHGHM SGGQA ETLLQ AKGEP WTFLV RESLS QPGDF VLSVL SDQPK AGPGS PLRVT HIKVM CEGGR YTVGG LETFD SLTDL VEHFK KTGIE EASGA FVYLR QPYYA TRVNA ADIEN RVLEL NKKQE SEDTA KAGFW EEFES LQKQE VKNLH QRLEG QRPEN KGKNR YKNIL PFDHS RVILQ GRDSN IPGSD YINAN YIKNQ LLGPD ENAKT YIASQ GCLEA TVNDF WQMAW QENSR VIVMT TREVE KGRNK CVPYW PEVGM QRAYG PYSVT NCGEH DTTEY KLRTL QVSPL DNGDL IREIW HYQYL SWPDH GVPSE PGGVL SFLDQ INQRQ ESLPH AGPII VHCSA GIGRT GTIIV IDMLM ENIST KGLDC DIDIQ KTIQM VRAQR SGMVQ TEAQY KFIYV AIAQF IETTK KKLEV LQSQK GQESE YGNIT YPPAM KNAHA KASRT SSKSL ESSAG TVAAS PVRRG GQRGL PVPGP PVLSP DLHQL PVLAP LHPAA DTRRM CMRTC TLRTR GRRK
XP 006719057.1	MSGGQ AETLL QAKGE PWTFL VRESL SQPGD FVLSV LSDQP KAGPG SPLRV THIKV MCEGG RYTVG GLETF DSLTD LVEHF KKTGI EEASG AFVYL RQPYY ATRVN AADIE NRVLE LNKKQ ESEDT AKAGF WEEFE SLQKQ EVKNL HQRLE GQRPE NKGKN RYKNI LPFDH SRVIL QGRDS NIPGS DYINA NYIKN QLLGP DENAK TYIAS QGCLE ATVND FWQMA WQENS RVIVM TTREV EKGRN KCVPY WPEVG MQRAY GPYSV TNCGE HDTTE YKLRT LQVSP LDNGD LIREI WHYQY LSWPD HGVPS EPGGV LSFLD QINQR QESLP HAGPI IVHCS AGIGR TGTII VIDML MENIS TKGLD CDIDI QKTIQ MVRAQ RSGMV QTEAQ YKFIY VAIAQ FIETT KKKLE VLQSQ KGQES EYGNI TYPPA MKNAH AKASR TSSKH KEDVY ENLHT KNKRE EKVKK QRSAD KEKSK GSLKR K
XP 011519290.1	MLSRG WFHRD LSGLD AETLL KGRGV HGSFL ARPSR KNQGD FSLSV RVGDQ VTHIR IQNSG DFYDL YGGEK FATLT ELVEY YTQQQ GVLQD RDGTI IHLKY PLNCS DPTSE RWYHG HMSGG QAETL LQAKG EPWTF LVRES LSQPG DFVLS VLSDQ PKAGP GSPLR VTHIK VMCEG GRYTV GGLET FDSLT DLVEH FKKTG IEEAS GAFVY LRQPY YATRV NAADI ENRVL ELNKK QESED TAKAG FWEEF ESLQK QEVKN LHQRL EGQRP ENKGK NRYKN ILPFD HSRVI LQGRD SNIPG SDYIN ANYIK NQLLG PDENA KTYIA SQGCL EATVN DFWQM AWQEN SRVIV MTTRE VEKGR NKCVP YWPEV GMQRA YGPYS VTNCG EHDTT EYKLR TLQVS PLDNG DLIRE IWHYQ YLSWP DHGVP SEPGG VLSFL DQINQ RQESL PHAGP IIVHC SAGIG RTGTI IVIDM LMENI STKGL DCDID IQKTI QMVRA QRSGM VQTEA QYKFI YVAIA QFIET TKKKL EVLQS QKGQE SEYGN ITYPP AMKNA HAKAS RTSSK HKEDV YENLH TKNKR EEKVK KQRSA DKEKS KGSLK RK
XP 02 43048 74.1	MVRWF HRDLS GLDAE TLLKG RGVHG SFLAR PSRKN QGDFS LSVRV GDQVT HIRIQ NSGDF YDLYG GEKFA TLTEL VEYYT QQQGV LQDRD GTIIH LKYPL NCSDP TSERW YHGHM SGGQA ETLLQ AKGEP WTFLV RESLS QPGDF VLSVL SDQPK AGPGS PLRVT HIKVM CEGGR YTVGG LETFD SLTDL VEHFK KTGIE EASGA FVYLR QPYYA TRVNA ADIEN RVLEL NKKQE SEDTA KAGFW EEFES LQKQE VKNLH QRLEG QRPEN KGKNR YKNIL PFDHS RVILQ GRDSN IPGSD YINAN YIKNQ LLGPD ENAKT YIASQ GCLEA TVNDF WQMAW QENSR VIVMT TREVE KGRNK CVPYW PEVGM QRAYG PYSVT NCGEH DTTEY KLRTL QVSPL DNGDL IREIW HYQYL SWPDH GVPSE PGGVL SFLDQ INQRQ ESLPH AGPII VHCSA GIGRT GTIIV IDMLM ENIST KGLDC DIDIQ KTIQM VRAQR SGMVQ TEAQY KFIYV AIAQF IETTK KKLEV LQSQK GQESE YGNIT YPPAM KNAHA KASRT SSKHK EDVYE NLHTK NKREE KVKKQ RSADK EKSKG SLKRK

Table A4. The parameter Identity of the HELIOS method in the quantitative measurement of homology, assuming the *Hub Proteins Resource, Protein-Protein Interactions Dataset, PTPN6 Gene Set* [3].

	NP 002822.2	NP 536858.1	NP 536859.1	XP 006719057.1	XP 011519290.1	XP 024304874.1
NP 002822.2	100	99.8319	94.7899	100	99.8319	100
NP 536858.1		100	94.3049	100	100	99.8319
NP 536859.1			100	93.5551	94.4724	94.958
XP 006719057.1				100	100	100
XP 011519290.1					100	99.8319
XP 024304874.1						100

Table A5. The parameter Similarity of the HELIOS method in the quantitative measurement of homology, assuming the *Hub Proteins Resource, Protein-Protein Interactions Dataset, PTPN6 Gene Set* [3].

	NP 002822.2	NP 536858.1	NP 536859.1	XP 006719057.1	XP 011519290.1	XP 024304874.1
NP 002822.2	100	99.8319	95.2941	100	99.8319	100
NP 536858.1		100	94.8074	100	100	99.8319
NP 536859.1			100	94.1788	94.9749	95.2941
XP 006719057.1				100	100	100
XP 011519290.1					100	100
XP 024304874.1						100

Table A6. The parameter Alignment Score of the HELIOS method in the quantitative measurement of homology, assuming the *Hub Proteins Resource, Protein-Protein Interactions Dataset, PTPN6 Gene Set* [3].

	NP 002822.2	NP 536858.1	NP 536859.1	XP 006719057.1	XP 011519290.1	XP 024304874.1
NP 002822.2	3146	3120	2914.5	2462.5	3120	3146
NP 536858.1		3156	2888.5	2461.5	3156	3120
NP 536859.1			3302	2230	2896.5	2909
XP 006719057.1				2529	2461.5	2462.5
XP 011519290.1					3156	3123
XP 024304874.1						3146

Table A7. The parameter Identity of the Smith-Waterman algorithm in the quantitative measurement of homology, assuming the *Hub Proteins Resource, Protein-Protein Interactions Dataset, PTPN6 Gene Set* [3].

	NP 002822.2	NP 536858.1	NP 536859.1	XP 006719057.1	XP 011519290.1	XP 024304874.1
NP 002822.2	100	99.4958	94.2857	100	99.4958	100
NP 536858.1		100	93.4673	100	100	99.4958
NP 536859.1			100	92.9314	93.4673	94.2857
XP 006719057.1				100	100	100
XP 011519290.1					100	99.4958
XP 024304874.1						100

Table A8. The parameter Similarity of the Smith-Waterman algorithm in the quantitative measurement of homology, assuming the *Hub Proteins Resource, Protein-Protein Interactions Dataset, PTPN6 Gene Set* [3].

	NP 002822.2	NP 536858.1	NP 536859.1	XP 006719057.1	XP 011519290.1	XP 024304874.1
NP 002822.2	100	99.6639	94.7899	100	99.6639	100
NP 536858.1		100	94.1374	100	100	99.6639
NP 536859.1			100	93.5551	94.1374	94.7899
XP 006719057.1				100	100	100
XP 011519290.1					100	99.6639
XP 024304874.1						100

Table A9. The parameter Alignment Score of the Smith-Waterman algorithm in the quantitative measurement of homology, assuming the *Hub Proteins Resource, Protein-Protein Interactions Dataset, PTPN6 Gene Set* [3].

	NP 002822.2	NP 536858.1	NP 536859.1	XP 006719057.1	XP 011519290.1	XP 024304874.1
NP 002822.2	3146	3115.5	2908	2462.5	3115.5	3146
NP 536858.1		3156	2877.5	2461.5	3156	3115.5
NP 536859.1			3302	2224.5	2877.5	2908
XP 006719057.1				2529	2461.5	2462.5
XP 011519290.1					3156	3115.5
XP 024304874.1						3146

Table A10. The parameter Identity of the Needleman-Wunsch algorithm in the quantitative measurement of homology, assuming the *Hub Proteins Resource, Protein-Protein Interactions Dataset, PTPN6 Gene Set* [3].

	NP 002822.2	NP 536858.1	NP 536859.1	XP 006719057.1	XP 011519290.1	XP 024304874.1
NP 002822.2	100	99.4958	93.9496	100	99.4958	100
NP 536858.1		100	93.1323	100	100	99.4958
NP 536859.1			100	92.5156	93.1323	93.9496
XP 006719057.1				100	100	100
XP 011519290.1					100	99.4958
XP 024304874.1						100

Table A11. The parameter Similarity of the Needleman-Wunsch algorithm in the quantitative measurement of homology, assuming the *Hub Proteins Resource, Protein-Protein Interactions Dataset, PTPN6 Gene Set* [3].

	NP 002822.2	NP 536858.1	NP 536859.1	XP 006719057.1	XP 011519290.1	XP 024304874.1
NP 002822.2	100	99.4958	93.9496	100	99.4958	100
NP 536858.1		100	93.1323	100	100	99.4958
NP 536859.1			100	92.5156	93.1323	93.9496
XP 006719057.1				100	100	100
XP 011519290.1					100	99.4958
XP 024304874.1						100

Table A12. The parameter Alignment Score of the Needleman-Wunsch algorithm in the quantitative measurement of homology, assuming the *Hub Proteins Resource, Protein-Protein Interactions Dataset, PTPN6 Gene Set* [3].

	NP 002822.2	NP 536858.1	NP 536859.1	XP 006719057.1	XP 011519290.1	XP 024304874.1
NP 002822.2	3146	3132	2958	2529	3132	3146
NP 536858.1		3156	2944	2529	3156	3132
NP 536859.1			3302	2341	2944	2958
XP 006719057.1				2529	2529	2529
XP 011519290.1					3156	3132
XP 024304874.1						3146

Table A13. The parameter Identity of the BLAST in the quantitative measurement of homology, assuming the *Hub Proteins Resource, Protein-Protein Interactions Dataset, PTPN6 Gene Set* [3].

	NP 002822.2	NP 536858.1	NP 536859.1	XP 006719057.1	XP 011519290.1	XP 024304874.1
NP 002822.2	100	99.6639	94.958	100	99.6639	100
NP 536858.1		100	94.3049	100	100	99.6639
NP 536859.1			100	93.763	94.3049	94.958
XP 006719057.1				100	100	100
XP 011519290.1					100	99.6639
XP 024304874.1						100

Table A14. The parameter Similarity of the BLAST in the quantitative measurement of homology, assuming the *Hub Proteins Resource, Protein-Protein Interactions Dataset, PTPN6 Gene Set* [3].

	NP 002822.2	NP 536858.1	NP 536859.1	XP 006719057.1	XP 011519290.1	XP 024304874.1
NP 002822.2	100	99.8319	95.9664	100	99.8319	100
NP 536858.1		100	95.4774	100	100	99.8319
NP 536859.1			100	95.0104	95.4774	95.9664
XP 006719057.1				100	100	100
XP 011519290.1					100	99.8319
XP 024304874.1						100

Table A15. The parameter Alignment Score of the BLAST in the quantitative measurement of homology, assuming the *Hub Proteins Resource, Protein-Protein Interactions Dataset, PTPN6 Gene Set* [3].

	NP 002822.2	NP 536858.1	NP 536859.1	XP 006719057.1	XP 011519290.1	XP 024304874.1
NP 002822.2	3146	3126.5	2932	2462.5	3126.5	3146
NP 536858.1		3156	2912.5	2461.5	3156	3126.5
NP 536859.1			3302	2248.5	2912.5	2932
XP 006719057.1				2529	2461.5	2462.5
XP 011519290.1					3156	3126.5
XP 024304874.1						3146

Table A16. The parameter Identity of the ClustalW in the quantitative measurement of homology, assuming the *Hub Proteins Resource, Protein-Protein Interactions Dataset, PTPN6 Gene Set* [3].

	NP 002822.2	NP 536858.1	NP 536859.1	XP 006719057.1	XP 011519290.1	XP 024304874.1
NP 002822.2	100	99.6639	94.6218	100	99.6639	100
NP 536858.1		100	93.9698	100	100	99.6639
NP 536859.1			100	93.3472	93.9698	94.6218
XP 006719057.1				100	100	100
XP 011519290.1					100	99.6639
XP 024304874.1						100

Table A17. The parameter Similarity of the ClustalW in the quantitative measurement of homology, assuming the *Hub Proteins Resource, Protein-Protein Interactions Dataset, PTPN6 Gene Set* [3].

	NP 002822.2	NP 536858.1	NP 536859.1	XP 006719057.1	XP 011519290.1	XP 024304874.1
NP 002822.2	100	99.6639	95.4622	100	99.6639	100
NP 536858.1		100	94.8074	100	100	99.6639
NP 536859.1			100	94.3867	94.8074	95.4622
XP 006719057.1				100	100	100
XP 011519290.1					100	99.6639
XP 024304874.1						100

Table A18. The parameter Alignment Score of the ClustalW in the quantitative measurement of homology, assuming the *Hub Proteins Resource, Protein-Protein Interactions Dataset, PTPN6 Gene Set* [3].

	NP 002822.2	NP 536858.1	NP 536859.1	XP 006719057.1	XP 011519290.1	XP 024304874.1
NP 002822.2	3146	3117	2913	2462.5	3117	3146
NP 536858.1		3156	2884	2461.5	3156	3117
NP 536859.1			3302	2229.5	2884	2913
XP 006719057.1				2529	2461.5	2462.5
XP 011519290.1					3156	3117
XP 024304874.1						3146

Table A19. The parameter Identity of the ClustalΩ in the quantitative measurement of homology, assuming the *Hub Proteins Resource, Protein-Protein Interactions Dataset, PTPN6 Gene Set* [3].

	NP 002822.2	NP 536858.1	NP 536859.1	XP 006719057.1	XP 011519290.1	XP 024304874.1
NP 002822.2	100	99.4958	94.2857	100	99.4958	100
NP 536858.1		100	93.4673	100	100	99.4958
NP 536859.1			100	92.9314	93.4673	94.2857
XP 006719057.1				100	100	100
XP 011519290.1					100	99.4958
XP 024304874.1						100

Table A20. The parameter Similarity of the ClustalΩ in the quantitative measurement of homology, assuming the *Hub Proteins Resource, Protein-Protein Interactions Dataset, PTPN6 Gene Set* [3].

	NP 002822.2	NP 536858.1	NP 536859.1	XP 006719057.1	XP 011519290.1	XP 024304874.1
NP 002822.2	100	99.6639	94.7899	100	99.6639	100
NP 536858.1		100	94.1374	100	100	99.6639
NP 536859.1			100	93.5551	94.1374	94.7899
XP 006719057.1				100	100	100
XP 011519290.1					100	99.6639
XP 024304874.1						100

Table A21. The parameter Alignment Score of the ClustalΩ in the quantitative measurement of homology, assuming the *Hub Proteins Resource, Protein-Protein Interactions Dataset, PTPN6 Gene Set* [3].

	NP 002822.2	NP 536858.1	NP 536859.1	XP 006719057.1	XP 011519290.1	XP 024304874.1
NP 002822.2	3146	3115.5	2908	2462.5	3115.5	3146
NP 536858.1		3156	2877.5	2461.5	3156	3115.5
NP 536859.1			3302	2224.5	2877.5	2908
XP 006719057.1				2529	2461.5	2462.5
XP 011519290.1					3156	3115.5
XP 024304874.1						3146

Table A22. The parameter Identity of the MUSCLE in the quantitative measurement of homology, assuming the *Hub Proteins Resource, Protein-Protein Interactions Dataset, PTPN6 Gene Set* [3].

	NP 002822.2	NP 536858.1	NP 536859.1	XP 006719057.1	XP 011519290.1	XP 024304874.1
NP 002822.2	100	99.4958	94.958	100	99.4958	100
NP 536858.1		100	94.1374	100	100	99.4958
NP 536859.1			100	93.763	94.1374	94.958
XP 006719057.1				100	100	100
XP 011519290.1					100	99.4958
XP 024304874.1						100

Table A23. The parameter Similarity of the MUSCLE in the quantitative measurement of homology, assuming the *Hub Proteins Resource, Protein-Protein Interactions Dataset, PTPN6 Gene Set* [3].

	NP 002822.2	NP 536858.1	NP 536859.1	XP 006719057.1	XP 011519290.1	XP 024304874.1
NP 002822.2	100	99.6639	96.1345	100	99.6639	100
NP 536858.1		100	95.4774	100	100	99.6639
NP 536859.1			100	95.2183	95.4774	96.1345
XP 006719057.1				100	100	100
XP 011519290.1					100	99.6639
XP 024304874.1						100

Table A24. The parameter Alignment Score of the MUSCLE in the quantitative measurement of homology, assuming the *Hub Proteins Resource, Protein-Protein Interactions Dataset, PTPN6 Gene Set* [3].

	NP 002822.2	NP 536858.1	NP 536859.1	XP 006719057.1	XP 011519290.1	XP 024304874.1
NP 002822.2	3146	3115.5	2937	2462.5	3115.5	3146
NP 536858.1		3156	2906.5	2461.5	3156	3115.5
NP 536859.1			3302	2253.5	2906.5	2937
XP 006719057.1				2529	2461.5	2462.5
XP 011519290.1					3156	3115.5
XP 024304874.1						3146

Table A25. The parameter Identity of the T-Coffee in the quantitative measurement of homology, assuming the *Hub Proteins Resource, Protein-Protein Interactions Dataset, PTPN6 Gene Set* [3].

	NP 002822.2	NP 536858.1	NP 536859.1	XP 006719057.1	XP 011519290.1	XP 024304874.1
NP 002822.2	100	99.6639	94.958	100	99.6639	100
NP 536858.1		100	94.3049	100	100	99.6639
NP 536859.1			100	93.763	94.3049	94.958
XP 006719057.1				100	100	100
XP 011519290.1					100	99.6639
XP 024304874.1						100

Table A26. The parameter Similarity of the T-Coffee in the quantitative measurement of homology, assuming the *Hub Proteins Resource, Protein-Protein Interactions Dataset, PTPN6 Gene Set* [3].

	NP 002822.2	NP 536858.1	NP 536859.1	XP 006719057.1	XP 011519290.1	XP 024304874.1
NP 002822.2	100	99.8319	95.7983	100	99.8319	100
NP 536858.1		100	95.3099	100	100	99.8319
NP 536859.1			100	94.8025	95.3099	95.7983
XP 006719057.1				100	100	100
XP 011519290.1					100	99.8319
XP 024304874.1						100

Table A27. The parameter Alignment Score of the T-Coffee in the quantitative measurement of homology, assuming the *Hub Proteins Resource, Protein-Protein Interactions Dataset, PTPN6 Gene Set* [3].

	NP 002822.2	NP 536858.1	NP 536859.1	XP 006719057.1	XP 011519290.1	XP 024304874.1
NP 002822.2	3146	3125.5	2923.5	2462.5	3125.5	3146
NP 536858.1		3156	2903	2461.5	3156	3125.5
NP 536859.1			3302	2240	2903	2923.5
XP 006719057.1				2529	2461.5	2462.5
XP 011519290.1					3156	3125.5
XP 024304874.1						3146

Table A28. The parameter Identity of the Kalign in the quantitative measurement of homology, assuming the *Hub Proteins Resource, Protein-Protein Interactions Dataset, PTPN6 Gene Set* [3].

	NP 002822.2	NP 536858.1	NP 536859.1	XP 006719057.1	XP 011519290.1	XP 024304874.1
NP 002822.2	100	99.6639	94.2857	100	99.6639	100
NP 536858.1		100	93.6348	100	100	99.6639
NP 536859.1			100	92.9314	93.6348	94.2857
XP 006719057.1				100	100	100
XP 011519290.1					100	99.6639
XP 024304874.1						100

Table A29. The parameter Similarity of the Kalign in the quantitative measurement of homology, assuming the *Hub Proteins Resource, Protein-Protein Interactions Dataset, PTPN6 Gene Set* [3].

	NP 002822.2	NP 536858.1	NP 536859.1	XP 006719057.1	XP 011519290.1	XP 024304874.1
NP 002822.2	100	99.8319	94.7899	100	99.8319	100
NP 536858.1		100	94.3049	100	100	99.8319
NP 536859.1			100	93.5551	94.3049	94.7899
XP 006719057.1				100	100	100
XP 011519290.1					100	99.8319
XP 024304874.1						100

Table A30. The parameter Alignment Score of the Kalign in the quantitative measurement of homology, assuming the *Hub Proteins Resource, Protein-Protein Interactions Dataset, PTPN6 Gene Set* [3].

	NP 002822.2	NP 536858.1	NP 536859.1	XP 006719057.1	XP 011519290.1	XP 024304874.1
NP 002822.2	3146	3125.5	2908	2462.5	3125.5	3146
NP 536858.1		3156	2887.5	2461.5	3156	3125.5
NP 536859.1			3302	2224.5	2887.5	2908
XP 006719057.1				2529	2461.5	2462.5
XP 011519290.1					3156	3125.5
XP 024304874.1						3146

Table A31. The parameter Identity of the MAFFT in the quantitative measurement of homology, assuming the *Hub Proteins Resource, Protein-Protein Interactions Dataset, PTPN6 Gene Set* [3].

	NP 002822.2	NP 536858.1	NP 536859.1	XP 006719057.1	XP 011519290.1	XP 024304874.1
NP 002822.2	100	99.6639	94.958	100	99.6639	100
NP 536858.1		100	94.3049	100	100	99.6639
NP 536859.1			100	93.763	94.3049	94.958
XP 006719057.1				100	100	100
XP 011519290.1					100	99.6639
XP 024304874.1						100

Table A32. The parameter Similarity of the MAFFT in the quantitative measurement of homology, assuming the *Hub Proteins Resource, Protein-Protein Interactions Dataset, PTPN6 Gene Set* [3].

	NP 002822.2	NP 536858.1	NP 536859.1	XP 006719057.1	XP 011519290.1	XP 024304874.1
NP 002822.2	100	99.8319	96.1345	100	99.8319	100
NP 536858.1		100	95.6449	100	100	99.8319
NP 536859.1			100	95.2183	95.6449	96.1345
XP 006719057.1				100	100	100
XP 011519290.1					100	99.8319
XP 024304874.1						100

Table A33. The parameter Alignment Score of the MAFFT in the quantitative measurement of homology, assuming the *Hub Proteins Resource, Protein-Protein Interactions Dataset, PTPN6 Gene Set* [3].

	NP 002822.2	NP 536858.1	NP 536859.1	XP 006719057.1	XP 011519290.1	XP 024304874.1
NP 002822.2	3146	3126.5	2937	2462.5	3126.5	3146
NP 536858.1		3156	2917.5	2461.5	3156	3126.5
NP 536859.1			3302	2253.5	2917.5	2937
XP 006719057.1				2529	2461.5	2462.5
XP 011519290.1					3156	3126.5
XP 024304874.1						3146

Table A34. The parameter Sensitivity (SEN) of the HELIOS method with referencing the Smith-Waterman algorithm in the accuracy measurement of classification output, assuming the *Hub Proteins Resource, Protein-Protein Interactions Dataset, PTPN6 Gene Set* [3].

	NP 002822.2	NP 536858.1	NP 536859.1	XP 006719057.1	XP 011519290.1	XP 024304874.1
NP 002822.2	1	0.99496	0.96807	1	0.99496	1
NP 536858.1		1	0.96303	1	1	0.99496
NP 536859.1			1	0.93971	0.96639	0.95126
XP 006719057.1				1	1	1
XP 011519290.1					1	0.99496
XP 024304874.1						1

Table A35. The parameter Specification (Spec) of the HELIOS method with referencing the Smith-Waterman algorithm in the accuracy measurement of classification output, assuming the *Hub Proteins Resource, Protein-Protein Interactions Dataset, PTPN6 Gene Set* [3].

	NP 002822.2	NP 536858.1	NP 536859.1	XP 006719057.1	XP 011519290.1	XP 024304874.1
NP 002822.2	1	0.99999	0.99996	1	0.99999	1
NP 536858.1		1	0.99995	1	1	0.99999
NP 536859.1			1	0.9999	0.99995	0.99993
XP 006719057.1				1	1	1
XP 011519290.1					1	0.99999
XP 024304874.1						1

Table A36. The parameter Accuracy (Acc) of the HELIOS method with referencing the Smith-Waterman algorithm in the accuracy measurement of classification output, assuming the *Hub Proteins Resource, Protein-Protein Interactions Dataset, PTPN6 Gene Set* [3].

	NP 002822.2	NP 536858.1	NP 536859.1	XP 006719057.1	XP 011519290.1	XP 024304874.1
NP 002822.2	1	0.99998	0.99991	1	0.99998	1
NP 536858.1		1	0.99989	1	1	0.99998
NP 536859.1			1	0.99981	0.9999	0.99985
XP 006719057.1				1	1	1
XP 011519290.1					1	0.99998
XP 024304874.1						1

Table A37. The parameter Positive Predictive Value (PPV) of the HELIOS method with referencing the Smith-Waterman algorithm in the accuracy measurement of classification output, assuming the *Hub Proteins Resource, Protein-Protein Interactions Dataset, PTPN6 Gene Set* [3].

	NP 002822.2	NP 536858.1	NP 536859.1	XP 006719057.1	XP 011519290.1	XP 024304874.1
NP 002822.2	1	0.99496	0.97297	1	0.99496	1
NP 536858.1		1	0.96791	1	1	0.99496
NP 536859.1			1	0.93971	0.97128	0.95608
XP 006719057.1				1	1	1
XP 011519290.1					1	0.99496
XP 024304874.1						1

Table A38. The parameter Negative Predictive Value (NPV) of the HELIOS method with referencing the Smith-Waterman algorithm in the accuracy measurement of classification output, assuming the *Hub Proteins Resource, Protein-Protein Interactions Dataset, PTPN6 Gene Set* [3].

	NP 002822.2	NP 536858.1	NP 536859.1	XP 006719057.1	XP 011519290.1	XP 024304874.1
NP 002822.2	1	0.99999	0.99995	1	0.99999	1
NP 536858.1		1	0.99994	1	1	0.99999
NP 536859.1			1	0.9999	0.99995	0.99992
XP 006719057.1				1	1	1
XP 011519290.1					1	0.99999
XP 024304874.1						1

Table A39. The parameter Matthew's Coefficient Correlation (MCC) of the HELIOS method with referencing the Smith-Waterman algorithm in the accuracy measurement of classification output, assuming the *Hub Proteins Resource, Protein-Protein Interactions Dataset, PTPN6 Gene Set* [3].

	NP 002822.2	NP 536858.1	NP 536859.1	XP 006719057.1	XP 011519290.1	XP 024304874.1
NP 002822.2	1	0.99495	0.97047	1	0.99495	1
NP 536858.1		1	0.96541	1	1	0.99495
NP 536859.1			1	0.93961	0.96878	0.95359
XP 006719057.1				1	1	1
XP 011519290.1					1	0.99495
XP 024304874.1						1

Table A40. The parameter Test's Accuracy (F-Score) of the HELIOS method with referencing the Smith-Waterman algorithm in the accuracy measurement of classification output, assuming the *Hub Proteins Resource, Protein-Protein Interactions Dataset, PTPN6 Gene Set* [3].

	NP 002822.2	NP 536858.1	NP 536859.1	XP 006719057.1	XP 011519290.1	XP 024304874.1
NP 002822.2	1	0.99496	0.97051	1	0.99496	1
NP 536858.1		1	0.96546	1	1	0.99496
NP 536859.1			1	0.93971	0.96883	0.95366
XP 006719057.1				1	1	1
XP 011519290.1					1	0.99496
XP 024304874.1						1

Table A41. The parameter Sensitivity (SEN) of the HELIOS method with referencing the Needleman-Wunsch algorithm in the accuracy measurement of classification output, assuming the *Hub Proteins Resource, Protein-Protein Interactions Dataset, PTPN6 Gene Set* [3].

	NP 002822.2	NP 536858.1	NP 536859.1	XP 006719057.1	XP 011519290.1	XP 024304874.1
NP 002822.2	1	0.0016892	1	0	0.0016892	1
NP 536858.1		1	0.0017921	0	1	0.0016892
NP 536859.1			1	0	0.0017921	1
XP 006719057.1				1	0	0
XP 011519290.1					1	0.0033784
XP 024304874.1						1

Table A42. The parameter Specification (Spec) of the HELIOS method with referencing the Needleman-Wunsch algorithm in the accuracy measurement of classification output, assuming the *Hub Proteins Resource, Protein-Protein Interactions Dataset, PTPN6 Gene Set* [3].

	NP 002822.2	NP 536858.1	NP 536859.1	XP 006719057.1	XP 011519290.1	XP 024304874.1
NP 002822.2	1	0.99832	1	0.99841	0.99832	1
NP 536858.1		1	0.99821	0.99842	1	0.99832
NP 536859.1			1	0.99833	0.99821	1
XP 006719057.1				1	0.99842	0.99841
XP 011519290.1					1	0.99832
XP 024304874.1						1

Table A43. The parameter Accuracy (Acc) of the HELIOS method with referencing the Needleman-Wunsch algorithm in the accuracy measurement of classification output, assuming the *Hub Proteins Resource, Protein-Protein Interactions Dataset, PTPN6 Gene Set* [3].

	NP 002822.2	NP 536858.1	NP 536859.1	XP 006719057.1	XP 011519290.1	XP 024304874.1
NP 002822.2	1	0.99663	1	0.99633	0.99663	1
NP 536858.1		1	0.99643	0.99634	1	0.99663
NP 536859.1			1	0.9961	0.99643	1
XP 006719057.1				1	0.99634	0.99633
XP 011519290.1					1	0.99664
XP 024304874.1						1

Table A44. The parameter Positive Predictive Value (PPV) of the HELIOS method with referencing the Needleman-Wunsch algorithm in the accuracy measurement of classification output, assuming the *Hub Proteins Resource, Protein-Protein Interactions Dataset, PTPN6 Gene Set* [3].

	NP 002822.2	NP 536858.1	NP 536859.1	XP 006719057.1	XP 011519290.1	XP 024304874.1
NP 002822.2	1	0.0016949	1	0	0.0016949	1
NP 536858.1		1	0.0017986	0	1	0.0016949
NP 536859.1			1	0	0.0017986	1
XP 006719057.1				1	0	0
XP 011519290.1					1	0.0033898
XP 024304874.1						1

Table A45. The parameter Negative Predictive Value (NPV) of the HELIOS method with referencing the Needleman-Wunsch algorithm in the accuracy measurement of classification output, assuming the *Hub Proteins Resource, Protein-Protein Interactions Dataset, PTPN6 Gene Set* [3].

	NP 002822.2	NP 536858.1	NP 536859.1	XP 006719057.1	XP 011519290.1	XP 024304874.1
NP 002822.2	1	0.99831	1	0.99792	0.99831	1
NP 536858.1		1	0.99821	0.99792	1	0.99831
NP 536859.1			1	0.99776	0.99821	1
XP 006719057.1				1	0.99792	0.99792
XP 011519290.1					1	0.99831
XP 024304874.1						1

Table A46. The parameter Matthew's Coefficient Correlation (MCC) of the HELIOS method with referencing the Needleman-Wunsch algorithm in the accuracy measurement of classification output, assuming the *Hub Proteins Resource, Protein-Protein Interactions Dataset, PTPN6 Gene Set* [3].

	NP 002822.2	NP 536858.1	NP 536859.1	XP 006719057.1	XP 011519290.1	XP 024304874.1
NP 002822.2	1	5.726e-06	1	-0.0018193	5.726e-06	1
NP 536858.1		1	6.4464e-06	-0.0018144	1	5.726e-06
NP 536859.1			1	-0.0019347	6.4464e-06	1
XP 006719057.1				1	-0.0018144	-0.0018193
XP 011519290.1					1	0.0017006
XP 024304874.1						1

Table A47. The parameter Test's Accuracy (F-Score) of the HELIOS method with referencing the Needleman-Wunsch algorithm in the accuracy measurement of classification output, assuming the *Hub Proteins Resource, Protein-Protein Interactions Dataset, PTPN6 Gene Set* [3].

	NP 002822.2	NP 536858.1	NP 536859.1	XP 006719057.1	XP 011519290.1	XP 024304874.1
NP 002822.2	1	0.001692	1	0	0.001692	1
NP 536858.1		1	0.0017953	0	1	0.001692
NP 536859.1			1	0	0.0017953	1
XP 006719057.1				1	0	0
XP 011519290.1					1	0.0033841
XP 024304874.1						1

Table A48. The parameter Sensitivity (SEN) of the HELIOS method with referencing the BLAST in the accuracy measurement of classification output, assuming the *Hub Proteins Resource, Protein-Protein Interactions Dataset, PTPN6 Gene Set* [3].

	NP 002822.2	NP 536858.1	NP 536859.1	XP 006719057.1	XP 011519290.1	XP 024304874.1
NP 002822.2	1	0.99664	0.9747	1	0.99664	1
NP 536858.1		1	0.97133	1	1	0.99664
NP 536859.1			1	0.94781	0.97976	0.96627
XP 006719057.1				1	1	1
XP 011519290.1					1	0.99832
XP 024304874.1						1

Table A49. The parameter Specification (Spec) of the HELIOS method with referencing the BLAST in the accuracy measurement of classification output, assuming the *Hub Proteins Resource, Protein-Protein Interactions Dataset, PTPN6 Gene Set* [3].

	NP 002822.2	NP 536858.1	NP 536859.1	XP 006719057.1	XP 011519290.1	XP 024304874.1
NP 002822.2	1	0.99999	0.99996	1	0.99999	1
NP 536858.1		1	0.99996	1	1	0.99999
NP 536859.1			1	0.99991	0.99997	0.99995
XP 006719057.1				1	1	1
XP 011519290.1					1	1
XP 024304874.1						1

Table A50. The parameter Accuracy (Acc) of the HELIOS method with referencing the BLAST in the accuracy measurement of classification output, assuming the *Hub Proteins Resource, Protein-Protein Interactions Dataset, PTPN6 Gene Set* [3].

	NP 002822.2	NP 536858.1	NP 536859.1	XP 006719057.1	XP 011519290.1	XP 024304874.1
NP 002822.2	1	0.99999	0.99992	1	0.99999	1
NP 536858.1		1	0.99991	1	1	0.99999
NP 536859.1			1	0.99983	0.99994	0.99989
XP 006719057.1				1	1	1
XP 011519290.1					1	0.99999
XP 024304874.1						1

Table A51. The parameter Positive Predictive Value (PPV) of the HELIOS method with referencing the BLAST in the accuracy measurement of classification output, assuming the *Hub Proteins Resource, Protein-Protein Interactions Dataset, PTPN6 Gene Set* [3].

	NP 002822.2	NP 536858.1	NP 536859.1	XP 006719057.1	XP 011519290.1	XP 024304874.1
NP 002822.2	1	0.99664	0.97635	1	0.99664	1
NP 536858.1		1	0.97297	1	1	0.99664
NP 536859.1			1	0.94387	0.98142	0.96791
XP 006719057.1				1	1	1
XP 011519290.1					1	0.99832
XP 024304874.1						1

Table A52. The parameter Negative Predictive Value (NPV) of the HELIOS method with referencing the BLAST in the accuracy measurement of classification output, assuming the *Hub Proteins Resource, Protein-Protein Interactions Dataset, PTPN6 Gene Set* [3].

	NP 002822.2	NP 536858.1	NP 536859.1	XP 006719057.1	XP 011519290.1	XP 024304874.1
NP 002822.2	1	0.99999	0.99996	1	0.99999	1
NP 536858.1		1	0.99995	1	1	0.99999
NP 536859.1			1	0.99992	0.99997	0.99995
XP 006719057.1				1	1	1
XP 011519290.1					1	1
XP 024304874.1						1

Table A53. The parameter Matthew's Coefficient Correlation (MCC) of the HELIOS method with referencing the BLAST in the accuracy measurement of classification output, assuming the *Hub Proteins Resource, Protein-Protein Interactions Dataset, PTPN6 Gene Set* [3].

	NP 002822.2	NP 536858.1	NP 536859.1	XP 006719057.1	XP 011519290.1	XP 024304874.1
NP 002822.2	1	0.99663	0.97549	1	0.99663	1
NP 536858.1		1	0.97211	1	1	0.99663
NP 536859.1			1	0.94575	0.98056	0.96704
XP 006719057.1				1	1	1
XP 011519290.1					1	0.99832
XP 024304874.1						1

Table A54. The parameter Test's Accuracy (F-Score) of the HELIOS method with referencing the BLAST in the accuracy measurement of classification output, assuming the *Hub Proteins Resource, Protein-Protein Interactions Dataset, PTPN6 Gene Set* [3].

	NP 002822.2	NP 536858.1	NP 536859.1	XP 006719057.1	XP 011519290.1	XP 024304874.1
NP 002822.2	1	0.99664	0.97553	1	0.99664	1
NP 536858.1		1	0.97215	1	1	0.99664
NP 536859.1			1	0.94583	0.98059	0.96709
XP 006719057.1				1	1	1
XP 011519290.1					1	0.99832
XP 024304874.1						1

Table A55. The parameter Sensitivity (SEN) of the HELIOS method with referencing the ClustalW in the accuracy measurement of classification output, assuming the *Hub Proteins Resource, Protein-Protein Interactions Dataset, PTPN6 Gene Set* [3].

	NP 002822.2	NP 536858.1	NP 536859.1	XP 006719057.1	XP 011519290.1	XP 024304874.1
NP 002822.2	1	0.99832	0.96807	1	0.99832	1
NP 536858.1		1	0.96639	1	1	0.99832
NP 536859.1			1	0.95426	0.96975	0.95798
XP 006719057.1				1	1	1
XP 011519290.1					1	0.99664
XP 024304874.1						1

Table A56. The parameter Specification (Spec) of the HELIOS method with referencing the ClustalW in the accuracy measurement of classification output, assuming the *Hub Proteins Resource, Protein-Protein Interactions Dataset, PTPN6 Gene Set* [3].

	NP 002822.2	NP 536858.1	NP 536859.1	XP 006719057.1	XP 011519290.1	XP 024304874.1
NP 002822.2	1	1	0.99996	1	1	1
NP 536858.1		1	0.99995	1	1	1
NP 536859.1			1	0.99993	0.99996	0.99994
XP 006719057.1				1	1	1
XP 011519290.1					1	0.99999
XP 024304874.1						1

Table A57. The parameter Accuracy (Acc) of the HELIOS method with referencing the ClustalW in the accuracy measurement of classification output, assuming the *Hub Proteins Resource, Protein-Protein Interactions Dataset, PTPN6 Gene Set* [3].

	NP 002822.2	NP 536858.1	NP 536859.1	XP 006719057.1	XP 011519290.1	XP 024304874.1
NP 002822.2	1	0.99999	0.99991	1	0.99999	1
NP 536858.1		1	0.9999	1	1	0.99999
NP 536859.1			1	0.99985	0.99991	0.99987
XP 006719057.1				1	1	1
XP 011519290.1					1	0.99999
XP 024304874.1						1

Table A58. The parameter Positive Predictive Value (PPV) of the HELIOS method with referencing the ClustalW in the accuracy measurement of classification output, assuming the *Hub Proteins Resource, Protein-Protein Interactions Dataset, PTPN6 Gene Set* [3].

	NP 002822.2	NP 536858.1	NP 536859.1	XP 006719057.1	XP 011519290.1	XP 024304874.1
NP 002822.2	1	0.99832	0.97297	1	0.99832	1
NP 536858.1		1	0.97128	1	1	0.99832
NP 536859.1			1	0.95426	0.97466	0.96284
XP 006719057.1				1	1	1
XP 011519290.1					1	0.99664
XP 024304874.1						1

Table A59. The parameter Negative Predictive Value (NPV) of the HELIOS method with referencing the ClustalW in the accuracy measurement of classification output, assuming the *Hub Proteins Resource, Protein-Protein Interactions Dataset, PTPN6 Gene Set* [3].

	NP 002822.2	NP 536858.1	NP 536859.1	XP 006719057.1	XP 011519290.1	XP 024304874.1
NP 002822.2	1	1	0.99995	1	1	1
NP 536858.1		1	0.99995	1	1	1
NP 536859.1			1	0.99993	0.99995	0.99993
XP 006719057.1				1	1	1
XP 011519290.1					1	0.99999
XP 024304874.1						1

Table A60. The parameter Matthew's Coefficient Correlation (MCC) of the HELIOS method with referencing the ClustalW in the accuracy measurement of classification output, assuming the *Hub Proteins Resource, Protein-Protein Interactions Dataset, PTPN6 Gene Set* [3].

	NP 002822.2	NP 536858.1	NP 536859.1	XP 006719057.1	XP 011519290.1	XP 024304874.1
NP 002822.2	1	0.99832	0.97047	1	0.99832	1
NP 536858.1		1	0.96878	1	1	0.99832
NP 536859.1			1	0.95419	0.97216	0.96034
XP 006719057.1				1	1	1
XP 011519290.1					1	0.99663
XP 024304874.1						1

Table A61. The parameter Test's Accuracy (F-Score) of the HELIOS method with referencing the ClustalW in the accuracy measurement of classification output, assuming the *Hub Proteins Resource, Protein-Protein Interactions Dataset, PTPN6 Gene Set* [3].

	NP 002822.2	NP 536858.1	NP 536859.1	XP 006719057.1	XP 011519290.1	XP 024304874.1
NP 002822.2	1	0.99832	0.97051	1	0.99832	1
NP 536858.1		1	0.96883	1	1	0.99832
NP 536859.1			1	0.95426	0.9722	0.9604
XP 006719057.1				1	1	1
XP 011519290.1					1	0.99664
XP 024304874.1						1

Table A62. The parameter Sensitivity (SEN) of the HELIOS method with referencing the ClustalΩ in the accuracy measurement of classification output, assuming the *Hub Proteins Resource, Protein-Protein Interactions Dataset, PTPN6 Gene Set* [3].

	NP 002822.2	NP 536858.1	NP 536859.1	XP 006719057.1	XP 011519290.1	XP 024304874.1
NP 002822.2	1	0.99496	0.96807	1	0.99496	1
NP 536858.1		1	0.96303	1	1	0.99496
NP 536859.1			1	0.93971	0.96639	0.95126
XP 006719057.1				1	1	1
XP 011519290.1					1	0.99496
XP 024304874.1						1

Table A63. The parameter Specification (Spec) of the HELIOS method with referencing the ClustalΩ in the accuracy measurement of classification output, assuming the *Hub Proteins Resource, Protein-Protein Interactions Dataset, PTPN6 Gene Set* [3].

	NP 002822.2	NP 536858.1	NP 536859.1	XP 006719057.1	XP 011519290.1	XP 024304874.1
NP 002822.2	1	0.99999	0.99996	1	0.99999	1
NP 536858.1		1	0.99995	1	1	0.99999
NP 536859.1			1	0.9999	0.99995	0.99993
XP 006719057.1				1	1	1
XP 011519290.1					1	0.99999
XP 024304874.1						1

Table A64. The parameter Accuracy (Acc) of the HELIOS method with referencing the ClustalΩ in the accuracy measurement of classification output, assuming the *Hub Proteins Resource, Protein-Protein Interactions Dataset, PTPN6 Gene Set* [3].

	NP 002822.2	NP 536858.1	NP 536859.1	XP 006719057.1	XP 011519290.1	XP 024304874.1
NP 002822.2	1	0.99998	0.99991	1	0.99998	1
NP 536858.1		1	0.99989	1	1	0.99998
NP 536859.1			1	0.99981	0.9999	0.99985
XP 006719057.1				1	1	1
XP 011519290.1					1	0.99998
XP 024304874.1						1

Table A65. The parameter Positive Predictive Value (PPV) of the HELIOS method with referencing the ClustalΩ in the accuracy measurement of classification output, assuming the *Hub Proteins Resource, Protein-Protein Interactions Dataset, PTPN6 Gene Set* [3].

	NP 002822.2	NP 536858.1	NP 536859.1	XP 006719057.1	XP 011519290.1	XP 024304874.1
NP 002822.2	1	0.99496	0.97297	1	0.99496	1
NP 536858.1		1	0.96791	1	1	0.99496
NP 536859.1			1	0.93971	0.97128	0.95608
XP 006719057.1				1	1	1
XP 011519290.1					1	0.99496
XP 024304874.1						1

Table A66. The parameter Negative Predictive Value (NPV) of the HELIOS method with referencing the ClustalΩ in the accuracy measurement of classification output, assuming the *Hub Proteins Resource, Protein-Protein Interactions Dataset, PTPN6 Gene Set* [3].

	NP 002822.2	NP 536858.1	NP 536859.1	XP 006719057.1	XP 011519290.1	XP 024304874.1
NP 002822.2	1	0.99999	0.99995	1	0.99999	1
NP 536858.1		1	0.99994	1	1	0.99999
NP 536859.1			1	0.9999	0.99995	0.99992
XP 006719057.1				1	1	1
XP 011519290.1					1	0.99999
XP 024304874.1						1

Table A67. The parameter Matthew's Coefficient Correlation (MCC) of the HELIOS method with referencing the ClustalΩ in the accuracy measurement of classification output, assuming the *Hub Proteins Resource, Protein-Protein Interactions Dataset, PTPN6 Gene Set* [3].

	NP 002822.2	NP 536858.1	NP 536859.1	XP 006719057.1	XP 011519290.1	XP 024304874.1
NP 002822.2	1	0.99495	0.97047	1	0.99495	1
NP 536858.1		1	0.96541	1	1	0.99495
NP 536859.1			1	0.93961	0.96878	0.95359
XP 006719057.1				1	1	1
XP 011519290.1					1	0.99495
XP 024304874.1						1

Table A68. The parameter Test's Accuracy (F-Score) of the HELIOS method with referencing the ClustalΩ in the accuracy measurement of classification output, assuming the *Hub Proteins Resource, Protein-Protein Interactions Dataset, PTPN6 Gene Set* [3].

	NP 002822.2	NP 536858.1	NP 536859.1	XP 006719057.1	XP 011519290.1	XP 024304874.1
NP 002822.2	1	0.99496	0.97051	1	0.99496	1
NP 536858.1		1	0.96546	1	1	0.99496
NP 536859.1			1	0.93971	0.96883	0.95366
XP 006719057.1				1	1	1
XP 011519290.1					1	0.99496
XP 024304874.1						1

Table A69. The parameter Sensitivity (SEN) of the HELIOS method with referencing the MUSCLE in the accuracy measurement of classification output, assuming the *Hub Proteins Resource, Protein-Protein Interactions Dataset, PTPN6 Gene Set* [3].

	NP 002822.2	NP 536858.1	NP 536859.1	XP 006719057.1	XP 011519290.1	XP 024304874.1
NP 002822.2	1	0.99496	0.94915	1	0.99496	1
NP 536858.1		1	0.94407	1	1	0.99496
NP 536859.1			1	0.93697	0.94746	0.94915
XP 006719057.1				1	1	1
XP 011519290.1					1	0.99496
XP 024304874.1						1

Table A70. The parameter Specification (Spec) of the HELIOS method with referencing the MUSCLE in the accuracy measurement of classification output, assuming the *Hub Proteins Resource, Protein-Protein Interactions Dataset, PTPN6 Gene Set* [3].

	NP 002822.2	NP 536858.1	NP 536859.1	XP 006719057.1	XP 011519290.1	XP 024304874.1
NP 002822.2	1	0.99999	0.99991	1	0.99999	1
NP 536858.1		1	0.99991	1	1	0.99999
NP 536859.1			1	0.99988	0.99991	0.99991
XP 006719057.1				1	1	1
XP 011519290.1					1	0.99999
XP 024304874.1						1

Table A71. The parameter Accuracy (Acc) of the HELIOS method with referencing the MUSCLE in the accuracy measurement of classification output, assuming the *Hub Proteins Resource, Protein-Protein Interactions Dataset, PTPN6 Gene Set* [3].

	NP 002822.2	NP 536858.1	NP 536859.1	XP 006719057.1	XP 011519290.1	XP 024304874.1
NP 002822.2	1	0.99998	0.99983	1	0.99998	1
NP 536858.1		1	0.99982	1	1	0.99998
NP 536859.1			1	0.99978	0.99983	0.99983
XP 006719057.1				1	1	1
XP 011519290.1					1	0.99998
XP 024304874.1						1

Table A72. The parameter Positive Predictive Value (PPV) of the HELIOS method with referencing the MUSCLE in the accuracy measurement of classification output, assuming the *Hub Proteins Resource, Protein-Protein Interactions Dataset, PTPN6 Gene Set* [3].

	NP 002822.2	NP 536858.1	NP 536859.1	XP 006719057.1	XP 011519290.1	XP 024304874.1
NP 002822.2	1	0.99496	0.94595	1	0.99496	1
NP 536858.1		1	0.94088	1	1	0.99496
NP 536859.1			1	0.92723	0.94426	0.94595
XP 006719057.1				1	1	1
XP 011519290.1					1	0.99496
XP 024304874.1						1

Table A73. The parameter Negative Predictive Value (NPV) of the HELIOS method with referencing the MUSCLE in the accuracy measurement of classification output, assuming the *Hub Proteins Resource, Protein-Protein Interactions Dataset, PTPN6 Gene Set* [3].

	NP 002822.2	NP 536858.1	NP 536859.1	XP 006719057.1	XP 011519290.1	XP 024304874.1
NP 002822.2	1	0.99999	0.99992	1	0.99999	1
NP 536858.1		1	0.99991	1	1	0.99999
NP 536859.1			1	0.9999	0.99992	0.99992
XP 006719057.1				1	1	1
XP 011519290.1					1	0.99999
XP 024304874.1						1

Table A74. The parameter Matthew's Coefficient Correlation (MCC) of the HELIOS method with referencing the MUSCLE in the accuracy measurement of classification output, assuming the *Hub Proteins Resource, Protein-Protein Interactions Dataset, PTPN6 Gene Set* [3].

	NP 002822.2	NP 536858.1	NP 536859.1	XP 006719057.1	XP 011519290.1	XP 024304874.1
NP 002822.2	1	0.99495	0.94746	1	0.99495	1
NP 536858.1		1	0.94238	1	1	0.99495
NP 536859.1			1	0.93198	0.94577	0.94746
XP 006719057.1				1	1	1
XP 011519290.1					1	0.99495
XP 024304874.1						1

Table A75. The parameter Test's Accuracy (F-Score) of the HELIOS method with referencing the MUSCLE in the accuracy measurement of classification output, assuming the *Hub Proteins Resource, Protein-Protein Interactions Dataset, PTPN6 Gene Set* [3].

	NP 002822.2	NP 536858.1	NP 536859.1	XP 006719057.1	XP 011519290.1	XP 024304874.1
NP 002822.2	1	0.99496	0.94755	1	0.99496	1
NP 536858.1		1	0.94247	1	1	0.99496
NP 536859.1			1	0.93208	0.94585	0.94755
XP 006719057.1				1	1	1
XP 011519290.1					1	0.99496
XP 024304874.1						1

Table A76. The parameter Sensitivity (SEN) of the HELIOS method with referencing the T-Coffee in the accuracy measurement of classification output, assuming the *Hub Proteins Resource, Protein-Protein Interactions Dataset, PTPN6 Gene Set* [3].

	NP 002822.2	NP 536858.1	NP 536859.1	XP 006719057.1	XP 011519290.1	XP 024304874.1
NP 002822.2	1	0.99664	0.95763	1	0.99664	1
NP 536858.1		1	0.95424	1	1	0.99664
NP 536859.1			1	0.94748	0.95593	0.95763
XP 006719057.1				1	1	1
XP 011519290.1					1	0.99832
XP 024304874.1						1

Table A77. The parameter Specification (Spec) of the HELIOS method with referencing the T-Coffee in the accuracy measurement of classification output, assuming the *Hub Proteins Resource, Protein-Protein Interactions Dataset, PTPN6 Gene Set* [3].

	NP 002822.2	NP 536858.1	NP 536859.1	XP 006719057.1	XP 011519290.1	XP 024304874.1
NP 002822.2	1	0.99999	0.99993	1	0.99999	1
NP 536858.1		1	0.99992	1	1	0.99999
NP 536859.1			1	0.9999	0.99992	0.99993
XP 006719057.1				1	1	1
XP 011519290.1					1	1
XP 024304874.1						1

Table A78. The parameter Accuracy (Acc) of the HELIOS method with referencing the T-Coffee in the accuracy measurement of classification output, assuming the *Hub Proteins Resource, Protein-Protein Interactions Dataset, PTPN6 Gene Set* [3].

	NP 002822.2	NP 536858.1	NP 536859.1	XP 006719057.1	XP 011519290.1	XP 024304874.1
NP 002822.2	1	0.99999	0.99986	1	0.99999	1
NP 536858.1		1	0.99985	1	1	0.99999
NP 536859.1			1	0.99982	0.99986	0.99986
XP 006719057.1				1	1	1
XP 011519290.1					1	0.99999
XP 024304874.1						1

Table A79. The parameter Positive Predictive Value (PPV) of the HELIOS method with referencing the T-Coffee in the accuracy measurement of classification output, assuming the *Hub Proteins Resource, Protein-Protein Interactions Dataset, PTPN6 Gene Set* [3].

	NP 002822.2	NP 536858.1	NP 536859.1	XP 006719057.1	XP 011519290.1	XP 024304874.1
NP 002822.2	1	0.99664	0.95439	1	0.99664	1
NP 536858.1		1	0.95101	1	1	0.99664
NP 536859.1			1	0.93763	0.9527	0.95439
XP 006719057.1				1	1	1
XP 011519290.1					1	0.99832
XP 024304874.1						1

Table A80. The parameter Negative Predictive Value (NPV) of the HELIOS method with referencing the T-Coffee in the accuracy measurement of classification output, assuming the *Hub Proteins Resource, Protein-Protein Interactions Dataset, PTPN6 Gene Set* [3].

	NP 002822.2	NP 536858.1	NP 536859.1	XP 006719057.1	XP 011519290.1	XP 024304874.1
NP 002822.2	1	0.99999	0.99993	1	0.99999	1
NP 536858.1		1	0.99993	1	1	0.99999
NP 536859.1			1	0.99992	0.99993	0.99993
XP 006719057.1				1	1	1
XP 011519290.1					1	1
XP 024304874.1						1

Table A81. The parameter Matthew's Coefficient Correlation (MCC) of the HELIOS method with referencing the T-Coffee in the accuracy measurement of classification output, assuming the *Hub Proteins Resource, Protein-Protein Interactions Dataset, PTPN6 Gene Set* [3].

	NP 002822.2	NP 536858.1	NP 536859.1	XP 006719057.1	XP 011519290.1	XP 024304874.1
NP 002822.2	1	0.99663	0.95594	1	0.99663	1
NP 536858.1		1	0.95255	1	1	0.99663
NP 536859.1			1	0.94245	0.95424	0.95594
XP 006719057.1				1	1	1
XP 011519290.1					1	0.99832
XP 024304874.1						1

Table A82. The parameter Test's Accuracy (F-Score) of the HELIOS method with referencing the T-Coffee in the accuracy measurement of classification output, assuming the *Hub Proteins Resource, Protein-Protein Interactions Dataset, PTPN6 Gene Set* [3].

	NP 002822.2	NP 536858.1	NP 536859.1	XP 006719057.1	XP 011519290.1	XP 024304874.1
NP 002822.2	1	0.99664	0.95601	1	0.99664	1
NP 536858.1		1	0.95262	1	1	0.99664
NP 536859.1			1	0.94253	0.95431	0.95601
XP 006719057.1				1	1	1
XP 011519290.1					1	0.99832
XP 024304874.1						1

Table A83. The parameter Sensitivity (SEN) of the HELIOS method with referencing the Kalign in the accuracy measurement of classification output, assuming the *Hub Proteins Resource, Protein-Protein Interactions Dataset, PTPN6 Gene Set* [3].

	NP 002822.2	NP 536858.1	NP 536859.1	XP 006719057.1	XP 011519290.1	XP 024304874.1
NP 002822.2	1	0.99664	0.96807	1	0.99664	1
NP 536858.1		1	0.96471	1	1	0.99664
NP 536859.1			1	0.93971	0.96639	0.95126
XP 006719057.1				1	1	1
XP 011519290.1					1	0.99832
XP 024304874.1						1

Table A84. The parameter Specification (Spec) of the HELIOS method with referencing the Kalign in the accuracy measurement of classification output, assuming the *Hub Proteins Resource, Protein-Protein Interactions Dataset, PTPN6 Gene Set* [3].

	NP 002822.2	NP 536858.1	NP 536859.1	XP 006719057.1	XP 011519290.1	XP 024304874.1
NP 002822.2	1	0.99999	0.99996	1	0.99999	1
NP 536858.1		1	0.99995	1	1	0.99999
NP 536859.1			1	0.9999	0.99995	0.99993
XP 006719057.1				1	1	1
XP 011519290.1					1	1
XP 024304874.1						1

Table A85. The parameter Accuracy (Acc) of the HELIOS method with referencing the Kalign in the accuracy measurement of classification output, assuming the *Hub Proteins Resource, Protein-Protein Interactions Dataset, PTPN6 Gene Set* [3].

	NP 002822.2	NP 536858.1	NP 536859.1	XP 006719057.1	XP 011519290.1	XP 024304874.1
NP 002822.2	1	0.99999	0.99991	1	0.99999	1
NP 536858.1		1	0.9999	1	1	0.99999
NP 536859.1			1	0.99981	0.9999	0.99985
XP 006719057.1				1	1	1
XP 011519290.1					1	0.99999
XP 024304874.1						1

Table A86. The parameter Positive Predictive Value (PPV) of the HELIOS method with referencing the Kalign in the accuracy measurement of classification output, assuming the *Hub Proteins Resource, Protein-Protein Interactions Dataset, PTPN6 Gene Set* [3].

	NP 002822.2	NP 536858.1	NP 536859.1	XP 006719057.1	XP 011519290.1	XP 024304874.1
NP 002822.2	1	0.99664	0.97297	1	0.99664	1
NP 536858.1		1	0.96959	1	1	0.99664
NP 536859.1			1	0.93971	0.97128	0.95608
XP 006719057.1				1	1	1
XP 011519290.1					1	0.99832
XP 024304874.1						1

Table A87. The parameter Negative Predictive Value (NPV) of the HELIOS method with referencing the Kalign in the accuracy measurement of classification output, assuming the *Hub Proteins Resource, Protein-Protein Interactions Dataset, PTPN6 Gene Set* [3].

	NP 002822.2	NP 536858.1	NP 536859.1	XP 006719057.1	XP 011519290.1	XP 024304874.1
NP 002822.2	1	0.99999	0.99995	1	0.99999	1
NP 536858.1		1	0.99994	1	1	0.99999
NP 536859.1			1	0.9999	0.99995	0.99992
XP 006719057.1				1	1	1
XP 011519290.1					1	1
XP 024304874.1						1

Table A88. The parameter Matthew's Coefficient Correlation (MCC) of the HELIOS method with referencing the Kalign in the accuracy measurement of classification output, assuming the *Hub Proteins Resource, Protein-Protein Interactions Dataset, PTPN6 Gene Set* [3].

	NP 002822.2	NP 536858.1	NP 536859.1	XP 006719057.1	XP 011519290.1	XP 024304874.1
NP 002822.2	1	0.99663	0.97047	1	0.99663	1
NP 536858.1		1	0.96709	1	1	0.99663
NP 536859.1			1	0.93961	0.96878	0.95359
XP 006719057.1				1	1	1
XP 011519290.1					1	0.99832
XP 024304874.1						1

Table A89. The parameter Test's Accuracy (F-Score) of the HELIOS method with referencing the Kalign in the accuracy measurement of classification output, assuming the *Hub Proteins Resource, Protein-Protein Interactions Dataset, PTPN6 Gene Set* [3].

	NP 002822.2	NP 536858.1	NP 536859.1	XP 006719057.1	XP 011519290.1	XP 024304874.1
NP 002822.2	1	0.99664	0.97051	1	0.99664	1
NP 536858.1		1	0.96714	1	1	0.99664
NP 536859.1			1	0.93971	0.96883	0.95366
XP 006719057.1				1	1	1
XP 011519290.1					1	0.99832
XP 024304874.1						1

Table A90. The parameter Sensitivity (SEN) of the HELIOS method with referencing the MAFFT in the accuracy measurement of classification output, assuming the *Hub Proteins Resource, Protein-Protein Interactions Dataset, PTPN6 Gene Set* [3].

	NP 002822.2	NP 536858.1	NP 536859.1	XP 006719057.1	XP 011519290.1	XP 024304874.1
NP 002822.2	1	0.99664	0.94915	1	0.99664	1
NP 536858.1		1	0.94576	1	1	0.99664
NP 536859.1			1	0.93697	0.94576	0.94915
XP 006719057.1				1	1	1
XP 011519290.1					1	0.99832
XP 024304874.1						1

Table A91. The parameter Specification (Spec) of the HELIOS method with referencing the MAFFT in the accuracy measurement of classification output, assuming the *Hub Proteins Resource, Protein-Protein Interactions Dataset, PTPN6 Gene Set* [3].

	NP 002822.2	NP 536858.1	NP 536859.1	XP 006719057.1	XP 011519290.1	XP 024304874.1
NP 002822.2	1	0.99999	0.99991	1	0.99999	1
NP 536858.1		1	0.99991	1	1	0.99999
NP 536859.1			1	0.99988	0.99991	0.99991
XP 006719057.1				1	1	1
XP 011519290.1					1	1
XP 024304874.1						1

Table A92. The parameter Accuracy (Acc) of the HELIOS method with referencing the MAFFT in the accuracy measurement of classification output, assuming the *Hub Proteins Resource, Protein-Protein Interactions Dataset, PTPN6 Gene Set* [3].

	NP 002822.2	NP 536858.1	NP 536859.1	XP 006719057.1	XP 011519290.1	XP 024304874.1
NP 002822.2	1	0.99999	0.99983	1	0.99999	1
NP 536858.1		1	0.99982	1	1	0.99999
NP 536859.1			1	0.99978	0.99982	0.99983
XP 006719057.1				1	1	1
XP 011519290.1					1	0.99999
XP 024304874.1						1

Table A93. The parameter Positive Predictive Value (PPV) of the HELIOS method with referencing the MAFFT in the accuracy measurement of classification output, assuming the *Hub Proteins Resource, Protein-Protein Interactions Dataset, PTPN6 Gene Set* [3].

	NP 002822.2	NP 536858.1	NP 536859.1	XP 006719057.1	XP 011519290.1	XP 024304874.1
NP 002822.2	1	0.99664	0.94595	1	0.99664	1
NP 536858.1		1	0.94257	1	1	0.99664
NP 536859.1			1	0.92723	0.94257	0.94595
XP 006719057.1				1	1	1
XP 011519290.1					1	0.99832
XP 024304874.1						1

Table A94. The parameter Negative Predictive Value (NPV) of the HELIOS method with referencing the MAFFT in the accuracy measurement of classification output, assuming the *Hub Proteins Resource, Protein-Protein Interactions Dataset, PTPN6 Gene Set* [3].

	NP 002822.2	NP 536858.1	NP 536859.1	XP 006719057.1	XP 011519290.1	XP 024304874.1
NP 002822.2	1	0.99999	0.99992	1	0.99999	1
NP 536858.1		1	0.99991	1	1	0.99999
NP 536859.1			1	0.9999	0.99991	0.99992
XP 006719057.1				1	1	1
XP 011519290.1					1	1
XP 024304874.1						1

Table A95. The parameter Matthew's Coefficient Correlation (MCC) of the HELIOS method with referencing the MAFFT in the accuracy measurement of classification output, assuming the *Hub Proteins Resource, Protein-Protein Interactions Dataset, PTPN6 Gene Set* [3].

	NP 002822.2	NP 536858.1	NP 536859.1	XP 006719057.1	XP 011519290.1	XP 024304874.1
NP 002822.2	1	0.99663	0.94746	1	0.99663	1
NP 536858.1		1	0.94408	1	1	0.99663
NP 536859.1			1	0.93198	0.94408	0.94746
XP 006719057.1				1	1	1
XP 011519290.1					1	0.99832
XP 024304874.1						1

Table A96. The parameter Test's Accuracy (F-Score) of the HELIOS method with referencing the MAFFT in the accuracy measurement of classification output, assuming the *Hub Proteins Resource, Protein-Protein Interactions Dataset, PTPN6 Gene Set* [3].

	NP 002822.2	NP 536858.1	NP 536859.1	XP 006719057.1	XP 011519290.1	XP 024304874.1
NP 002822.2	1	0.99664	0.94755	1	0.99664	1
NP 536858.1		1	0.94416	1	1	0.99664
NP 536859.1			1	0.93208	0.94416	0.94755
XP 006719057.1				1	1	1
XP 011519290.1					1	0.99832
XP 024304874.1						1
