# Supplementary material for: HELIOS: High-speed sequence alignment in optics
Source: PLoS Comput Biol. 2022 Nov 21;18(11):e1010665. doi: 10.1371/journal.pcbi.1010665 (PMC9678324; doi:10.1371/journal.pcbi.1010665)
Supplement: S11 Text — (PDF) [file pcbi.1010665.s011.pdf]

HELIOS: High-Speed Sequence Alignment in Optics: S11 Text

EHSAN MALEKI[1], SAEEDEH AKBARI ROKN ABADI[1], AND SOMAYYEH KOOHI[1,*]

[1] Department of Computer Engineering, Sharif University of Technology, Azadi Ave., Tehran, Iran.
[*] Corresponding author: koohi@sharif.edu

Compiled October 20, 2022

In response to the imperfections of current sequence alignment methods, originated from the inherent serialism within their corresponding electrical systems, a few optical approaches for biological data comparison have been proposed recently. However, due to their low performance, raised from their inefficient coding scheme, this paper presents a novel all-optical high-throughput method for aligning DNA, RNA, and protein sequences, named HELIOS. The HELIOS method employs highly sophisticated operations to locate character matches, single or multiple mutations, and single or multiple indels within various biological sequences. On the other hand, the HELIOS optical architecture exploits high-speed processing and operational parallelism in optics, by adopting wavelength and polarization of optical beams. For evaluation, the functionality and accuracy of the HELIOS method are approved through behavioral and optical simulation studies, while its complexity and performance are estimated through analytical computation. The accuracy evaluations indicate that the HELIOS method achieves a precise pairwise alignment of two sequences, highly similar to those of Smith-Waterman, Needleman-Wunsch, BLAST, MUSCLE, ClustalW, ClustalΩ, T-Coffee, Kalign, and MAFFT. According to our performance evaluations, the HELIOS optical architecture outperforms all alternative electrical and optical algorithms in terms of processing time and memory requirement, relying on its highly sophisticated method and optical architecture. Moreover, the employed compact coding scheme highly escalates the number of input characters, and hence, it offers reduced time and space complexities, compared to the electrical and optical alternatives. It makes the HELIOS method and optical architecture highly applicable for biomedical applications.

1. ACCURACY EVALUATION

In order to comprehensively assess the accuracy of the HELIOS method, two statistical analyses are performed through simulation studies: 1) Quantitative measurement of homology [1], and 2) Accuracy measurement of classification output [2], compared to the well-known algorithms, briefly reported in Tables A1 and A2, respectively. In this manner, the *Jaspar PWMs Resource, JASPAR Predicted Transcription Factor Targets Dataset, MAX Gene Set* [3] is assumed in this study, as represented in Table A3.

A. Quantitative measurement of homology

To perform quantitative measurement of homology [1], the parameters Identity, Similarity, and Alignment Score of the HELIOS outputs are calculated through simulation studies, as reported in Tables A4-A6, respectively, assuming the *Jaspar PWMs Resource, JASPAR Predicted Transcription Factor Targets Dataset, MAX Gene Set* [3]. While the Identity reports the number of exactly matched characters of two sequences (in percentage), the Similarity measures the resemblance of two compared sequences. Specifically, regarding the physicochemical properties, the amino acids are categorized into six groups with different Similarity values; including GAVLI, FYW, STCM, KRH, DENQ, and P. As the third metric, the BLOSUM62 [4] substitution scoring matrix [4] is adopted to calculate the Alignment Score, with gap opening and extension penalties equal to -10 and -0.5, respectively.

For a comparative study, the values of Identity, Similarity, and Alignment Score of the quantitative measurement of homology is performed by various well-known algorithms to be compared to the those of HELIOS method, assuming *Jaspar PWMs Resource, JASPAR Predicted Transcription Factor Targets Dataset, MAX Gene Set* [3]. It includes Smith-Waterman (SW) [5] reported in Tables A7-A9, Needleman-Wunsch (NW) [6] reported in Tables A10-A12, BLAST [7] reported in Tables A13-A15, ClustalW [8] reported in Tables A16-A18, Clustal-Omega [9] reported in Tables A19-A21, MUSCLE [9] reported in Tables A22-A24, T-Coffee [10] reported in Tables A25-A27, Kalign [11] reported in Tables A28-A30, and MAFFT [12] reported in Tables A31-A33.

Table A1. A brief report of the quantitative measurement of homology of the HELIOS method, compared to nine well-known algorithms, including SW, NW, BLAST, ClustalW, Clustal Omega, Muscle, T-Coffee, Kalign, and MAFFT. The parameters Identity, Similarity, and Alignment score are averaged and reported. The dataset used in this study is the *Jaspar PWMs Resource, JASPAR Predicted Transcription Factor Targets Dataset, MAX Gene Set* [3].

	HELIOS	SW	NW	BLAST	MUSCLE	ClustalW	ClustalΩ	T-Coffee	Kalign	MAFFT
Identity	60.091	57.542	57.221	59.907	59.627	56.930	57.537	58.120	59.507	60.746
Similarity	65.998	65.924	61.076	64.187	65.922	62.916	62.075	62.805	63.478	66.591
Alignment Score	217.9	220.7	280.8	232.8	232.9	206.8	215.9	214.7	231.4	238.7

Table A2. A brief report of the accuracy measurement of classification output of the HELIOS method with referencing well-known algorithms, including SW, NW, BLAST, ClustalW, Clustal Omega, Muscle, T-Coffee, Kalign, and MAFFT. The parameters SEN, Spec, Acc, PPV, NPV, MCC, and F-Score are averaged and reported. The dataset used in this study is the *Jaspar PWMs Resource, JASPAR Predicted Transcription Factor Targets Dataset, MAX Gene Set* [3].

	SW	NW	BLAST	MUSCLE	ClustalW	ClustalΩ	T-Coffee	Kalign	MAFFT
SEN	0.59860	0.57446	0.59893	0.57025	0.56972	0.58862	0.58200	0.58769	0.60765
Spec	0.99689	0.99392	0.99684	0.99667	0.99678	0.99675	0.99672	0.99680	0.99690
ACC	0.99357	0.98456	0.99491	0.99383	0.99325	0.99503	0.99425	0.99462	0.99444
PPV	0.60935	0.99031	0.59979	0.57311	0.57597	0.58637	0.56966	0.59457	0.60220
NPV	0.99663	0.99031	0.99804	0.99712	0.99642	0.99825	0.99749	0.99778	0.99751
MCC	0.60062	0.59308	0.59684	0.56850	0.56933	0.58520	0.57253	0.58843	0.60190
F-Score	0.60374	0.59697	0.59915	0.57149	0.57261	0.58742	0.57497	0.59093	0.60443

B. Accuracy measurement of classification output

Afterward, the accuracy measurement of the classification output [2] of the HELIOS method is addressed by calculating the values of Sensitivity (SEN), Specificity (Spec), Accuracy (ACC), Positive Predictive Value (PPV), Negative Predictive Value (NPV), Matthew's Coefficient Correlation (MCC), and Test's Accuracy (F-Score) in the simulation studies, according to Eq 5 to Eq 11, respectively.

As a comparative study, the accuracy measurement of the classification output of the HELIOS method is accomplished, assuming *Jaspar PWMs Resource, JASPAR Predicted Transcription Factor Targets Dataset, MAX Gene Set* [3], and the corresponding metrics are calculated with considering Smith-Waterman [5] reported in Tables A34-A40, Needleman-Wunsch [6] reported in Tables A41-A47, ClustalW [8] reported in Tables A55-A61, Clustal-Omega [9] reported in Tables A62-A68, BLAST [7] reported in Tables A48-A54, MUSCLE [13] reported in Tables A69-A75, T-Coffee [10] reported in Tables A76-A82, Kalign [11] reported in Tables A83-A89, and MAFFT [12] reported in Tables A90-A96.

REFERENCES

1. D. S. Moss, S. Jelaska, and S. Pongor, *Essays in bioinformatics*, vol. 368 (IOS Press, 2005).
2. M. Hamada, H. Kiryu, W. Iwasaki, and K. Asai, "Generalized centroid estimators in bioinformatics," PloS one **6**, e16450 (2011).
3. A. Mathelier, X. Zhao, A. W. Zhang, F. Parcy, R. Worsley-Hunt, D. J. Arenillas, S. Buchman, C.-y. Chen, A. Chou, H. Ienasescu *et al.*, "Jaspar 2014: an extensively expanded and updated open-access database of transcription factor binding profiles," Nucleic acids research **42**, D142–D147 (2014).
4. D. W. Mount, "Using blosum in sequence alignments," Cold Spring Harb. Protoc. **2008**, pdb.top39 (2008).
5. H. Zou, S. Tang, C. Yu, H. Fu, Y. Li, and W. Tang, "asw: accelerating smith–waterman algorithm on coupled cpu–gpu architecture," Int. J. Parallel Program. **47**, 388–402 (2019).
6. Y. Jararweh, M. Al-Ayyoub, M. Fakirah, L. Alawneh, and B. B. Gupta, "Improving the performance of the needleman-wunsch algorithm using parallelization and vectorization techniques," Multimed. Tools Appl. **78**, 3961–3977 (2019).
7. G. M. Boratyn, J. Thierry-Mieg, D. Thierry-Mieg, B. Busby, and T. L. Madden, "Magic-blast, an accurate rna-seq aligner for long and short reads," BMC bioinformatics **20**, 1–19 (2019).
8. D. Díaz, F. J. Esteban, P. Hernández, J. A. Caballero, A. Guevara, G. Dorado, and S. Gálvez, "Mc64-clustalwp2: A highly-parallel hybrid strategy to align multiple sequences in many-core architectures," PLOS ONE **9**, 1–12 (2014).
9. F. Sievers and D. G. Higgins, "Clustal omega for making accurate alignments of many protein sequences," Protein Sci. **27**, 135–145 (2018).
10. C. Notredame, D. G. Higgins, and J. Heringa, "T-coffee: a novel method for fast and accurate multiple sequence alignment11edited by j. thornton," J. Mol. Biol. **302**, 205–217 (2000).
11. T. Lassmann, "Kalign 3: multiple sequence alignment of large datasets," (2020).
12. J. Rozewicki, S. Li, K. M. Amada, D. M. Standley, and K. Katoh, "Mafft-dash: integrated protein sequence and structural alignment," Nucleic acids research **47**, W5–W10 (2019).
13. R. C. Edgar, "MUSCLE: multiple sequence alignment with high accuracy and high throughput," Nucleic Acids Res. **32**, 1792–1797 (2004).

Table A3. The list of input sequences, assuming the *Jaspar PWMs Resource, JASPAR Predicted Transcription Factor Targets Dataset, MAX Gene Set* [3].

Name	Sequence
NP 001257997.1	MSDND DIEVE SDADK RAHHN ALERK RRDHI KDSFH SLRDS VPSLQ GEKLY FLFWK LCTPV LHRQS LMQKC HTFIS SYQVH KKKECK I
NP 001257998.1	MSDND DIEVE SDADK RAHHN ALERK RRDHI KDSFH SLRDS VPSLQ GEKGT KMKLT LPPVF PYEHL PFPTV FCHG
NP 001307344.1	MLFWS SKGKA RADQV LCSWG IHFSS SLMED ASKRK FRALE KARSS AQLQT NYPSS DNSLY TNAKG STISA FDGGS DSSSE SEPEE PQSRK KLRME AS
NP 002373.3	MSDND DIEVE SDEEQ PRFQS AADKR AHHNA LERKR RDHIK DSFHS LRDSV PSLQG EKASR AQILD KATEY IQYMR RKNHT HQQDI DDLKR QNALL EQQVR ALEKA RSSAQ LQTNY PSSDN SLYTN AKGST ISAFD GGSDS SSESE PEEPQ SRKKL RMEAS
NP 660087.1	MSDND DIEVE SDADK RAHHN ALERK RRDHI KDSFH SLRDS VPSLQ GEKAS RAQIL DKATE YIQYM RRKNH THQQD IDDLK RQNAL LEQQV RALEK ARSSA QLQTN YPSSD NSLYT NAKGS TISAF DGGSD SSSES EPEEP QSRKK LRMEA S
NP 660088.1	MSDND DIEVE SDEEQ PRFQS AADKR AHHNA LERKR RDHIK DSFHS LRDSV PSLQG EKASR AQILD KATEY IQYMR RKNHT HQQDI DDLKR QNALL EQQGE SES
NP 660089.1	MSDND DIEVE SDEEQ PRFQS AADKR AHHNA LERKR RDHIK DSFHS LRDSV PSLQG EKLYF LFWKL CTPVL HRQSL MQKCH TFISS YQVHK KKECK I
NP 932061.1	MSDND DIEVE SDEEQ PRFQS AADKR AHHNA LERKR RDHIK DSFHS LRDSV PSLQG EKGTK MKLTL PPVFP YEHLP FPTVF CHG
XP 011535075.1	MSDND DIEVE SDEEQ PRFQS AADKR AHHNA LERKR RDHIK DSFHS LRDSV PSLQG EKASR AQILD KATEY IQYMR RKNHT HQQDI DDLKR QNALL EQQGA LPAVG ITPGI RCLKR ALRSL EWNVL CKYQR LQVFR DSEPS PLSMV VQSGQ PMG
XP 016876801.1	MLFWS SKGKA RADQV LCSWG IHFSS SLMED ASKRK FRALE KARSS AQLQT NYPSS DNSLY TNAKG STISA FDGGS DSSSE SEPEE PQSRK KLRME AS
XP 016876802.1	MLFWS SKGKA RADQV LCSWG IHFSS SLMED ASKRK FRALE KARSS AQLQT NYPSS DNSLY TNAKG STISA FDGGS DSSSE SEPEE PQSRK KLRME AS

Table A4. The parameter Identity of the HELIOS method in the quantitative measurement of homology, assuming the *Jaspar PWMs Resource, JASPAR Predicted Transcription Factor Targets Dataset, MAX Gene Set* [3].

	NP 001257997.1	NP 001257998.1	NP 001307344.1	NP 002373.3	NP 660087.1	NP 660088.1	NP 660089.1	NP 932061.1	XP 011535075.1	XP 016876801.1	XP 016876802.1
NP 001257997.1	100	74.3243	13.7931	58.6207	58.6207	58.6207	100	63.8554	58.6207	13.7931	13.7931
NP 001257998.1		100	13.5135	64.8649	64.8649	64.8649	72.973	100	64.8649	13.5135	12.1622
NP 001307344.1			100	68.0412	69.0722	12.3711	14.5833	14.4578	12.3711	100	100
NP 002373.3				100	100	98.0583	63.5417	68.6747	71.8954	68.0412	68.0412
NP 660087.1					100	89.3204	54.1667	18.0723	14.5695	65.9794	68.0412
NP 660088.1						100	62.5	68.6747	96.1165	11.3402	11.3402
NP 660089.1							100	77.1084	62.5	14.5833	14.5833
NP 932061.1								100	68.6747	14.4578	14.4578
XP 011535075.1									100	12.3711	12.3711
XP 016876801.1										100	100
XP 016876802.1											100

Table A5. The parameter Similarity of the HELIOS method in the quantitative measurement of homology, assuming the *Jaspar PWMs Resource, JASPAR Predicted Transcription Factor Targets Dataset, MAX Gene Set* [3].

	NP 001257997.1	NP 001257998.1	NP 001307344.1	NP 002373.3	NP 660087.1	NP 660088.1	NP 660089.1	NP 932061.1	XP 011535075.1	XP 016876801.1	XP 016876802.1
NP 001257997.1	100	78.3784	24.1379	66.6667	66.6667	66.6667	100	68.6747	66.6667	24.1379	24.1379
NP 001257998.1		100	22.973	71.6216	71.6216	71.6216	74.3243	100	71.6216	22.973	22.973
NP 001307344.1			100	72.1649	72.1649	25.7732	27.0833	25.3012	24.7423	100	100
NP 002373.3				100	100	99.0291	67.7083	74.6988	77.1242	72.1649	72.1649
NP 660087.1					100	90.2913	59.375	36.1446	32.4503	72.1649	71.134
NP 660088.1						100	69.7917	74.6988	96.1165	24.7423	24.7423
NP 660089.1							100	79.5181	69.7917	27.0833	27.0833
NP 932061.1								100	74.6988	25.3012	25.3012
XP 011535075.1									100	24.7423	24.7423
XP 016876801.1										100	100
XP 016876802.1											100

Table A6. The parameter Alignment Score of the HELIOS method in the quantitative measurement of homology, assuming the *Jaspar PWMs Resource, JASPAR Predicted Transcription Factor Targets Dataset, MAX Gene Set* [3].

	NP 001257997.1	NP 001257998.1	NP 001307344.1	NP 002373.3	NP 660087.1	NP 660088.1	NP 660089.1	NP 932061.1	XP 011535075.1	XP 016876801.1	XP 016876802.1
NP 001257997.1	469	222.5	-105.5	162	176	190.5	455	195	165.5	-105.5	-105.5
NP 001257998.1		402	-93.5	169	183	197.5	205.5	388	172.5	-93.5	-96.5
NP 001307344.1			491	225	228	-88	-100.5	-108.5	-110	491	491
NP 002373.3				809	749	467.5	211.5	229	430.5	225	227
NP 660087.1					763	407.5	158.5	-29.5	-33.5	227.5	230.5
NP 660088.1						529	250.5	257.5	473.5	-94	-94
NP 660089.1							515	268.5	225.5	-100.5	-100.5
NP 932061.1								448	232.5	-108.5	-108.5
XP 011535075.1									792	-110	-110
XP 016876801.1										491	491
XP 016876802.1											491

Table A7. The parameter Identity of the Smith-Waterman algorithm in the quantitative measurement of homology, assuming the *Jaspar PWMs Resource, JASPAR Predicted Transcription Factor Targets Dataset, MAX Gene Set* [3].

	NP 001257997.1	NP 001257998.1	NP 001307344.1	NP 002373.3	NP 660087.1	NP 660088.1	NP 660089.1	NP 932061.1	XP 011535075.1	XP 016876801.1	XP 016876802.1
NP 001257997.1	100	70.2703	12.6437	44.8276	55.1724	44.8276	89.6552	51.8072	44.8276	12.6437	12.6437
NP 001257998.1		100	14.8649	52.7027	64.8649	52.7027	58.1081	87.8378	52.7027	14.8649	14.8649
NP 001307344.1			100	62.8866	62.8866	11.3402	10.4167	12.0482	11.3402	100	100
NP 002373.3				100	94.0397	95.1456	59.375	68.6747	66.6667	63.9175	62.8866
NP 660087.1					100	77.6699	40.625	46.988	56.2914	62.8866	62.8866
NP 660088.1						100	59.375	68.6747	96.1165	11.3402	11.3402
NP 660089.1							100	73.494	59.375	10.4167	10.4167
NP 932061.1								100	68.6747	12.0482	12.0482
XP 011535075.1									100	11.3402	11.3402
XP 016876801.1										100	100
XP 016876802.1											100

Table A8. The parameter Similarity of the Smith-Waterman algorithm in the quantitative measurement of homology, assuming the *Jaspar PWMs Resource, JASPAR Predicted Transcription Factor Targets Dataset, MAX Gene Set* [3].

	NP 001257997.1	NP 001257998.1	NP 001307344.1	NP 002373.3	NP 660087.1	NP 660088.1	NP 660089.1	NP 932061.1	XP 011535075.1	XP 016876801.1	XP 016876802.1
NP 001257997.1	100	74.3243	31.0345	57.4713	63.2184	57.4713	94.2529	60.241	57.4713	31.0345	31.0345
NP 001257998.1		100	29.7297	64.8649	71.6216	64.8649	67.5676	93.2432	64.8649	29.7297	29.7297
NP 001307344.1			100	68.0412	68.0412	29.8969	29.1667	27.7108	29.8969	100	100
NP 002373.3				100	96.6887	96.1165	66.6667	74.6988	72.549	67.0103	68.0412
NP 660087.1					100	82.5243	52.0833	57.8313	64.9007	68.0412	68.0412
NP 660088.1						100	66.6667	74.6988	96.1165	29.8969	29.8969
NP 660089.1							100	77.1084	66.6667	29.1667	29.1667
NP 932061.1								100	74.6988	27.7108	27.7108
XP 011535075.1									100	29.8969	29.8969
XP 016876801.1										100	100
XP 016876802.1											100

Table A9. The parameter Alignment Score of the Smith-Waterman algorithm in the quantitative measurement of homology, assuming the *Jaspar PWMs Resource, JASPAR Predicted Transcription Factor Targets Dataset, MAX Gene Set* [3].

	NP 001257997.1	NP 001257998.1	NP 001307344.1	NP 002373.3	NP 660087.1	NP 660088.1	NP 660089.1	NP 932061.1	XP 011535075.1	XP 016876801.1	XP 016876802.1
NP 001257997.1	469	209.5	-56	89.5	154.5	118	404	144.5	93	-56	-56
NP 001257998.1		402	-63.5	118	183	146.5	144.5	337	121.5	-63.5	-63.5
NP 001307344.1			491	214	218.5	-64.5	-71.5	-79	-89.5	491	491
NP 002373.3				809	698	462	200.5	229	455	216	214
NP 660087.1					763	351	89.5	118	351	218.5	218.5
NP 660088.1						529	229	257.5	473.5	-64.5	-64.5
NP 660089.1							515	255.5	204	-71.5	-71.5
NP 932061.1								448	232.5	-79	-79
XP 011535075.1									792	-89.5	-89.5
XP 016876801.1										491	491
XP 016876802.1											491

Table A10. The parameter Identity of the Needleman-Wunsch algorithm in the quantitative measurement of homology, assuming the *Jaspar PWMs Resource, JASPAR Predicted Transcription Factor Targets Dataset, MAX Gene Set* [3].

	NP 001257997.1	NP 001257998.1	NP 001307344.1	NP 002373.3	NP 660087.1	NP 660088.1	NP 660089.1	NP 932061.1	XP 011535075.1	XP 016876801.1	XP 016876802.1
NP 001257997.1	100	70.2703	11.4943	44.8276	55.1724	44.8276	89.6552	51.8072	44.8276	11.4943	11.4943
NP 001257998.1		100	13.5135	52.7027	64.8649	52.7027	58.1081	87.8378	52.7027	13.5135	13.5135
NP 001307344.1			100	62.8866	62.8866	11.3402	9.375	10.8434	11.3402	100	100
NP 002373.3				100	94.0397	95.1456	59.375	68.6747	64.0523	62.8866	62.8866
NP 660087.1					100	77.6699	40.625	46.988	52.9801	62.8866	62.8866
NP 660088.1						100	59.375	68.6747	96.1165	11.3402	11.3402
NP 660089.1							100	73.494	59.375	9.375	9.375
NP 932061.1								100	68.6747	10.8434	10.8434
XP 011535075.1									100	11.3402	11.3402
XP 016876801.1										100	100
XP 016876802.1											100

Table A11. The parameter Similarity of the Needleman-Wunsch algorithm in the quantitative measurement of homology, assuming the *Jaspar PWMs Resource, JASPAR Predicted Transcription Factor Targets Dataset, MAX Gene Set* [3].

	NP 001257997.1	NP 001257998.1	NP 001307344.1	NP 002373.3	NP 660087.1	NP 660088.1	NP 660089.1	NP 932061.1	XP 011535075.1	XP 016876801.1	XP 016876802.1
NP 001257997.1	100	72.973	18.3908	49.4253	55.1724	49.4253	94.2529	59.0361	49.4253	18.3908	18.3908
NP 001257998.1		100	21.6216	63.5135	70.2703	63.5135	66.2162	93.2432	63.5135	21.6216	21.6216
NP 001307344.1			100	62.8866	62.8866	19.5876	15.625	18.0723	19.5876	100	100
NP 002373.3				100	96.6887	95.1456	59.375	73.494	64.0523	62.8866	62.8866
NP 660087.1					100	81.5534	44.7917	56.6265	55.6291	62.8866	62.8866
NP 660088.1						100	59.375	73.494	96.1165	19.5876	19.5876
NP 660089.1							100	75.9036	59.375	15.625	15.625
NP 932061.1								100	73.494	18.0723	18.0723
XP 011535075.1									100	19.5876	19.5876
XP 016876801.1										100	100
XP 016876802.1											100

Table A12. The parameter Alignment Score of the Needleman-Wunsch algorithm in the quantitative measurement of homology, assuming the *Jaspar PWMs Resource, JASPAR Predicted Transcription Factor Targets Dataset, MAX Gene Set* [3].

	NP 001257997.1	NP 001257998.1	NP 001307344.1	NP 002373.3	NP 660087.1	NP 660088.1	NP 660089.1	NP 932061.1	XP 011535075.1	XP 016876801.1	XP 016876802.1
NP 001257997.1	469	242	26	201	250	201	420	193	201	26	26
NP 001257998.1		402	26	206	255	206	193	353	206	26	26
NP 001307344.1			491	302	302	18	20	20	18	491	491
NP 002373.3				809	714	505	296	301	505	302	302
NP 660087.1					763	410	201	206	410	302	302
NP 660088.1						529	296	301	511	18	18
NP 660089.1							515	288	296	20	20
NP 932061.1								448	301	20	20
XP 011535075.1									792	18	18
XP 016876801.1										491	491
XP 016876802.1											491

Table A13. The parameter Identity of the BLAST in the quantitative measurement of homology, assuming the *Jaspar PWMs Resource, JASPAR Predicted Transcription Factor Targets Dataset, MAX Gene Set* [3].

	NP 001257997.1	NP 001257998.1	NP 001307344.1	NP 002373.3	NP 660087.1	NP 660088.1	NP 660089.1	NP 932061.1	XP 011535075.1	XP 016876801.1	XP 016876802.1
NP 001257997.1	100	72.973	8.046	60.9195	60.9195	60.9195	100	65.0602	60.9195	8.046	8.046
NP 001257998.1		100	5.4054	67.5676	67.5676	67.5676	72.973	100	67.5676	5.4054	5.4054
NP 001307344.1			100	67.0103	67.0103	4.1237	7.2917	4.8193	10.3093	100	100
NP 002373.3				100	100	95.1456	64.5833	71.0843	67.9739	67.0103	67.0103
NP 660087.1					100	86.4078	55.2083	60.241	62.9139	67.0103	67.0103
NP 660088.1						100	64.5833	71.0843	96.1165	4.1237	4.1237
NP 660089.1							100	75.9036	64.5833	7.2917	7.2917
NP 932061.1								100	71.0843	4.8193	4.8193
XP 011535075.1									100	10.3093	10.3093
XP 016876801.1										100	100
XP 016876802.1											100

Table A14. The parameter Similarity of the BLAST in the quantitative measurement of homology, assuming the *Jaspar PWMs Resource, JASPAR Predicted Transcription Factor Targets Dataset, MAX Gene Set* [3].

	NP 001257997.1	NP 001257998.1	NP 001307344.1	NP 002373.3	NP 660087.1	NP 660088.1	NP 660089.1	NP 932061.1	XP 011535075.1	XP 016876801.1	XP 016876802.1
NP 001257997.1	100	77.027	14.9425	67.8161	67.8161	67.8161	100	68.6747	67.8161	14.9425	14.9425
NP 001257998.1		100	8.1081	75.6757	75.6757	75.6757	77.027	100	75.6757	8.1081	8.1081
NP 001307344.1			100	72.1649	72.1649	9.2784	13.5417	7.2289	23.7113	100	100
NP 002373.3				100	100	95.1456	70.8333	78.3133	73.8562	72.1649	72.1649
NP 660087.1					100	86.4078	61.4583	67.4699	68.8742	72.1649	72.1649
NP 660088.1						100	70.8333	78.3133	96.1165	9.2784	9.2784
NP 660089.1							100	79.5181	70.8333	13.5417	13.5417
NP 932061.1								100	78.3133	7.2289	7.2289
XP 011535075.1									100	23.7113	23.7113
XP 016876801.1										100	100
XP 016876802.1											100

Table A15. The parameter Alignment Score of the BLAST in the quantitative measurement of homology, assuming the *Jaspar PWMs Resource, JASPAR Predicted Transcription Factor Targets Dataset, MAX Gene Set* [3].

	NP 001257997.1	NP 001257998.1	NP 001307344.1	NP 002373.3	NP 660087.1	NP 660088.1	NP 660089.1	NP 932061.1	XP 011535075.1	XP 016876801.1	XP 016876802.1
NP 001257997.1	469	225	-89	178.5	192.5	207	455	211	182	-89	-89
NP 001257998.1		402	-102.5	178.5	192.5	207	211	388	182	-102.5	-102.5
NP 001307344.1			491	232.5	237	-107.5	-93.5	-107	-97.5	491	491
NP 002373.3				809	749	454.5	238.5	238.5	465.5	232.5	232.5
NP 660087.1					763	394.5	178.5	178.5	405.5	237	237
NP 660088.1						529	267	267	473.5	-107.5	-107.5
NP 660089.1							515	271	242	-93.5	-93.5
NP 932061.1								448	242	-107	-107
XP 011535075.1									792	-97.5	-97.5
XP 016876801.1										491	491
XP 016876802.1											491

Table A16. The parameter Identity of the ClustalW in the quantitative measurement of homology, assuming the *Jaspar PWMs Resource, JASPAR Predicted Transcription Factor Targets Dataset, MAX Gene Set* [3].

	NP 001257997.1	NP 001257998.1	NP 001307344.1	NP 002373.3	NP 660087.1	NP 660088.1	NP 660089.1	NP 932061.1	XP 011535075.1	XP 016876801.1	XP 016876802.1
NP 001257997.1	100	71.6216	12.6437	62.069	62.069	62.069	100	63.8554	62.069	12.6437	12.6437
NP 001257998.1		100	17.5676	66.2162	66.2162	66.2162	71.6216	100	66.2162	17.5676	17.5676
NP 001307344.1			100	13.4021	13.4021	13.4021	11.4583	15.6627	13.4021	100	100
NP 002373.3				100	100	95.1456	65.625	69.8795	66.6667	13.4021	13.4021
NP 660087.1					100	86.4078	56.25	59.0361	61.5894	13.4021	13.4021
NP 660088.1						100	65.625	69.8795	98.0583	13.4021	13.4021
NP 660089.1							100	74.6988	65.625	11.4583	11.4583
NP 932061.1								100	69.8795	15.6627	15.6627
XP 011535075.1									100	13.4021	13.4021
XP 016876801.1										100	100
XP 016876802.1											100

Table A17. The parameter Similarity of the ClustalW in the quantitative measurement of homology, assuming the *Jaspar PWMs Resource, JASPAR Predicted Transcription Factor Targets Dataset, MAX Gene Set* [3].

	NP 001257997.1	NP 001257998.1	NP 001307344.1	NP 002373.3	NP 660087.1	NP 660088.1	NP 660089.1	NP 932061.1	XP 011535075.1	XP 016876801.1	XP 016876802.1
NP 001257997.1	100	75.6757	25.2874	66.6667	66.6667	66.6667	100	67.4699	66.6667	25.2874	25.2874
NP 001257998.1		100	29.7297	68.9189	68.9189	68.9189	75.6757	100	68.9189	29.7297	29.7297
NP 001307344.1			100	26.8041	24.7423	26.8041	25	28.9157	26.8041	100	100
NP 002373.3				100	100	96.1165	69.7917	72.2892	72.549	26.8041	26.8041
NP 660087.1					100	87.3786	60.4167	61.4458	67.5497	24.7423	24.7423
NP 660088.1						100	69.7917	72.2892	99.0291	26.8041	26.8041
NP 660089.1							100	78.3133	69.7917	25	25
NP 932061.1								100	72.2892	28.9157	28.9157
XP 011535075.1									100	26.8041	26.8041
XP 016876801.1										100	100
XP 016876802.1											100

Table A18. The parameter Alignment Score of the ClustalW in the quantitative measurement of homology, assuming the *Jaspar PWMs Resource, JASPAR Predicted Transcription Factor Targets Dataset, MAX Gene Set* [3].

	NP 001257997.1	NP 001257998.1	NP 001307344.1	NP 002373.3	NP 660087.1	NP 660088.1	NP 660089.1	NP 932061.1	XP 011535075.1	XP 016876801.1	XP 016876802.1
NP 001257997.1	469	214	-62	183	197	211.5	455	200	186.5	-62	-62
NP 001257998.1		402	-77.5	139.5	153.5	168	200	388	143	-77.5	-77.5
NP 001307344.1			491	-85.5	-88.5	-57	-59	-74.5	-82	491	491
NP 002373.3				809	749	452.5	243	199.5	455	-85.5	-85.5
NP 660087.1					763	392.5	183	139.5	395	-88.5	-88.5
NP 660088.1						529	271.5	228	477	-57	-57
NP 660089.1							515	260	246.5	-59	-59
NP 932061.1								448	203	-74.5	-74.5
XP 011535075.1									792	-82	-82
XP 016876801.1										491	491
XP 016876802.1											491

Table A19. The parameter Identity of the ClustalΩ in the quantitative measurement of homology, assuming the *Jaspar PWMs Resource, JASPAR Predicted Transcription Factor Targets Dataset, MAX Gene Set* [3].

	NP 001257997.1	NP 001257998.1	NP 001307344.1	NP 002373.3	NP 660087.1	NP 660088.1	NP 660089.1	NP 932061.1	XP 011535075.1	XP 016876801.1	XP 016876802.1
NP 001257997.1	100	71.6216	1.1494	58.6207	59.7701	57.4713	98.8506	62.6506	58.6207	1.1494	1.1494
NP 001257998.1		100	2.7027	64.8649	66.2162	64.8649	70.2703	98.6486	64.8649	2.7027	2.7027
NP 001307344.1			100	58.7629	58.7629	2.0619	1.0417	2.4096	10.3093	100	100
NP 002373.3				100	99.3377	95.1456	63.5417	69.8795	66.6667	58.7629	58.7629
NP 660087.1					100	85.4369	53.125	57.8313	60.9272	58.7629	58.7629
NP 660088.1						100	62.5	69.8795	96.1165	2.0619	2.0619
NP 660089.1							100	74.6988	63.5417	1.0417	1.0417
NP 932061.1								100	69.8795	2.4096	2.4096
XP 011535075.1									100	10.3093	10.3093
XP 016876801.1										100	100
XP 016876802.1											100

Table A20. The parameter Similarity of the ClustalΩ in the quantitative measurement of homology, assuming the *Jaspar PWMs Resource, JASPAR Predicted Transcription Factor Targets Dataset, MAX Gene Set* [3].

	NP 001257997.1	NP 001257998.1	NP 001307344.1	NP 002373.3	NP 660087.1	NP 660088.1	NP 660089.1	NP 932061.1	XP 011535075.1	XP 016876801.1	XP 016876802.1
NP 001257997.1	100	74.3243	5.7471	65.5172	66.6667	64.3678	98.8506	65.0602	65.5172	5.7471	5.7471
NP 001257998.1		100	4.0541	72.973	74.3243	72.973	72.973	98.6486	72.973	4.0541	4.0541
NP 001307344.1			100	67.0103	67.0103	9.2784	5.2083	3.6145	27.8351	100	100
NP 002373.3				100	99.3377	96.1165	69.7917	77.1084	74.5098	67.0103	67.0103
NP 660087.1					100	86.4078	59.375	65.0602	68.8742	67.0103	67.0103
NP 660088.1						100	68.75	77.1084	96.1165	9.2784	9.2784
NP 660089.1							100	77.1084	69.7917	5.2083	5.2083
NP 932061.1								100	77.1084	3.6145	3.6145
XP 011535075.1									100	27.8351	27.8351
XP 016876801.1										100	100
XP 016876802.1											100

Table A21. The parameter Alignment Score of the ClustalΩ in the quantitative measurement of homology, assuming the *Jaspar PWMs Resource, JASPAR Predicted Transcription Factor Targets Dataset, MAX Gene Set* [3].

	NP 001257997.1	NP 001257998.1	NP 001307344.1	NP 002373.3	NP 660087.1	NP 660088.1	NP 660089.1	NP 932061.1	XP 011535075.1	XP 016876801.1	XP 016876802.1
NP 001257997.1	469	221.5	-132.5	141.5	163.5	166	447	199.5	132.5	-132.5	-132.5
NP 001257998.1		402	-106.5	153	175	181.5	199.5	380	156.5	-106.5	-106.5
NP 001307344.1			491	185.5	190	-116	-137	-111	-88.5	491	491
NP 002373.3				809	741	462	209.5	221	452	185.5	185.5
NP 660087.1					763	394	141.5	153	384	190	190
NP 660088.1						529	234	249.5	473.5	-116	-116
NP 660089.1							515	267.5	200.5	-137	-137
NP 932061.1								448	224.5	-111	-111
XP 011535075.1									792	-88.5	-88.5
XP 016876801.1										491	491
XP 016876802.1											491

Table A22. The parameter Identity of the MUSCLE in the quantitative measurement of homology, assuming the *Jaspar PWMs Resource, JASPAR Predicted Transcription Factor Targets Dataset, MAX Gene Set* [3].

	NP 001257997.1	NP 001257998.1	NP 001307344.1	NP 002373.3	NP 660087.1	NP 660088.1	NP 660089.1	NP 932061.1	XP 011535075.1	XP 016876801.1	XP 016876802.1
NP 001257997.1	100	71.6216	8.046	62.069	62.069	62.069	100	63.8554	62.069	8.046	8.046
NP 001257998.1		100	9.4595	68.9189	68.9189	68.9189	71.6216	100	68.9189	9.4595	9.4595
NP 001307344.1			100	51.5464	51.5464	11.3402	7.2917	8.4337	13.4021	100	100
NP 002373.3				100	100	98.0583	65.625	72.2892	67.3203	51.5464	51.5464
NP 660087.1					100	89.3204	56.25	61.4458	62.2517	51.5464	51.5464
NP 660088.1						100	65.625	72.2892	98.0583	11.3402	11.3402
NP 660089.1							100	74.6988	65.625	7.2917	7.2917
NP 932061.1								100	72.2892	8.4337	8.4337
XP 011535075.1									100	13.4021	13.4021
XP 016876801.1										100	100
XP 016876802.1											100

Table A23. The parameter Similarity of the MUSCLE in the quantitative measurement of homology, assuming the *Jaspar PWMs Resource, JASPAR Predicted Transcription Factor Targets Dataset, MAX Gene Set* [3].

	NP 001257997.1	NP 001257998.1	NP 001307344.1	NP 002373.3	NP 660087.1	NP 660088.1	NP 660089.1	NP 932061.1	XP 011535075.1	XP 016876801.1	XP 016876802.1
NP 001257997.1	100	74.3243	19.5402	67.8161	67.8161	67.8161	100	66.2651	67.8161	19.5402	19.5402
NP 001257998.1		100	21.6216	75.6757	75.6757	75.6757	74.3243	100	75.6757	21.6216	21.6216
NP 001307344.1			100	63.9175	63.9175	23.7113	17.7083	19.2771	29.8969	100	100
NP 002373.3				100	100	99.0291	70.8333	78.3133	73.2026	63.9175	63.9175
NP 660087.1					100	90.2913	61.4583	67.4699	68.2119	63.9175	63.9175
NP 660088.1						100	70.8333	78.3133	99.0291	23.7113	23.7113
NP 660089.1							100	77.1084	70.8333	17.7083	17.7083
NP 932061.1								100	78.3133	19.2771	19.2771
XP 011535075.1									100	29.8969	29.8969
XP 016876801.1										100	100
XP 016876802.1											100

Table A24. The parameter Alignment Score of the MUSCLE in the quantitative measurement of homology, assuming the *Jaspar PWMs Resource, JASPAR Predicted Transcription Factor Targets Dataset, MAX Gene Set* [3].

	NP 001257997.1	NP 001257998.1	NP 001307344.1	NP 002373.3	NP 660087.1	NP 660088.1	NP 660089.1	NP 932061.1	XP 011535075.1	XP 016876801.1	XP 016876802.1
NP 001257997.1	469	221.5	-87	183	197	211.5	455	207.5	186.5	-87	-87
NP 001257998.1		402	-78	169	183	197.5	207.5	388	172.5	-78	-78
NP 001307344.1			491	164	168.5	-51	-91.5	-82.5	-57	491	491
NP 002373.3				809	749	467.5	243	229	464	164	164
NP 660087.1					763	407.5	183	169	404	168.5	168.5
NP 660088.1						529	271.5	257.5	477	-51	-51
NP 660089.1							515	267.5	246.5	-91.5	-91.5
NP 932061.1								448	232.5	-82.5	-82.5
XP 011535075.1									792	-57	-57
XP 016876801.1										491	491
XP 016876802.1											491

Table A25. The parameter Identity of the T-Coffee in the quantitative measurement of homology, assuming the *Jaspar PWMs Resource, JASPAR Predicted Transcription Factor Targets Dataset, MAX Gene Set* [3].

	NP 001257997.1	NP 001257998.1	NP 001307344.1	NP 002373.3	NP 660087.1	NP 660088.1	NP 660089.1	NP 932061.1	XP 011535075.1	XP 016876801.1	XP 016876802.1
NP 001257997.1	100	71.6216	14.9425	60.9195	60.9195	60.9195	100	63.8554	60.9195	14.9425	14.9425
NP 001257998.1		100	20.2703	67.5676	67.5676	67.5676	71.6216	100	67.5676	20.2703	20.2703
NP 001307344.1			100	17.5258	17.5258	16.4948	13.5417	18.0723	16.4948	100	100
NP 002373.3				100	100	95.1456	64.5833	71.0843	69.281	17.5258	17.5258
NP 660087.1					100	86.4078	55.2083	60.241	64.2384	17.5258	17.5258
NP 660088.1						100	64.5833	71.0843	98.0583	16.4948	16.4948
NP 660089.1							100	74.6988	64.5833	13.5417	13.5417
NP 932061.1								100	71.0843	18.0723	18.0723
XP 011535075.1									100	16.4948	16.4948
XP 016876801.1										100	100
XP 016876802.1											100

Table A26. The parameter Similarity of the T-Coffee in the quantitative measurement of homology, assuming the *Jaspar PWMs Resource, JASPAR Predicted Transcription Factor Targets Dataset, MAX Gene Set* [3].

	NP 001257997.1	NP 001257998.1	NP 001307344.1	NP 002373.3	NP 660087.1	NP 660088.1	NP 660089.1	NP 932061.1	XP 011535075.1	XP 016876801.1	XP 016876802.1
NP 001257997.1	100	74.3243	21.8391	67.8161	67.8161	67.8161	100	66.2651	67.8161	21.8391	21.8391
NP 001257998.1		100	29.7297	75.6757	75.6757	75.6757	74.3243	100	75.6757	29.7297	29.7297
NP 001307344.1			100	23.7113	23.7113	22.6804	19.7917	26.506	22.6804	100	100
NP 002373.3				100	100	96.1165	70.8333	78.3133	77.1242	23.7113	23.7113
NP 660087.1					100	87.3786	61.4583	67.4699	72.1854	23.7113	23.7113
NP 660088.1						100	70.8333	78.3133	98.0583	22.6804	22.6804
NP 660089.1							100	77.1084	70.8333	19.7917	19.7917
NP 932061.1								100	78.3133	26.506	26.506
XP 011535075.1									100	22.6804	22.6804
XP 016876801.1										100	100
XP 016876802.1											100

Table A27. The parameter Alignment Score of the T-Coffee in the quantitative measurement of homology, assuming the *Jaspar PWMs Resource, JASPAR Predicted Transcription Factor Targets Dataset, MAX Gene Set* [3].

	NP 001257997.1	NP 001257998.1	NP 001307344.1	NP 002373.3	NP 660087.1	NP 660088.1	NP 660089.1	NP 932061.1	XP 011535075.1	XP 016876801.1	XP 016876802.1
NP 001257997.1	469	220.5	-69	174	188	202.5	455	206.5	177.5	-69	-69
NP 001257998.1		402	-39.5	176.5	190.5	205	206.5	388	180	-39.5	-39.5
NP 001307344.1			491	-75.5	-61.5	-52	-83	-53.5	-77	491	491
NP 002373.3				809	749	455.5	234	236.5	478.5	-75.5	-75.5
NP 660087.1					763	395.5	174	176.5	418.5	-61.5	-61.5
NP 660088.1						529	262.5	265	474	-52	-52
NP 660089.1							515	266.5	237.5	-83	-83
NP 932061.1								448	240	-53.5	-53.5
XP 011535075.1									792	-77	-77
XP 016876801.1										491	491
XP 016876802.1											491

Table A28. The parameter Identity of the Kalign in the quantitative measurement of homology, assuming the *Jaspar PWMs Resource, JASPAR Predicted Transcription Factor Targets Dataset, MAX Gene Set* [3].

	NP 001257997.1	NP 001257998.1	NP 001307344.1	NP 002373.3	NP 660087.1	NP 660088.1	NP 660089.1	NP 932061.1	XP 011535075.1	XP 016876801.1	XP 016876802.1
NP 001257997.1	100	66.2162	6.8966	62.069	62.069	62.069	100	59.0361	62.069	6.8966	6.8966
NP 001257998.1		100	1.3514	68.9189	68.9189	68.9189	66.2162	100	68.9189	1.3514	1.3514
NP 001307344.1			100	65.9794	65.9794	8.2474	6.25	1.2048	12.3711	100	100
NP 002373.3				100	100	95.1456	65.625	72.2892	66.6667	65.9794	65.9794
NP 660087.1					100	86.4078	56.25	61.4458	61.5894	65.9794	65.9794
NP 660088.1						100	65.625	72.2892	96.1165	8.2474	8.2474
NP 660089.1							100	69.8795	65.625	6.25	6.25
NP 932061.1								100	72.2892	1.2048	1.2048
XP 011535075.1									100	12.3711	12.3711
XP 016876801.1										100	100
XP 016876802.1											100

Table A29. The parameter Similarity of the Kalign in the quantitative measurement of homology, assuming the *Jaspar PWMs Resource, JASPAR Predicted Transcription Factor Targets Dataset, MAX Gene Set* [3].

	NP 001257997.1	NP 001257998.1	NP 001307344.1	NP 002373.3	NP 660087.1	NP 660088.1	NP 660089.1	NP 932061.1	XP 011535075.1	XP 016876801.1	XP 016876802.1
NP 001257997.1	100	70.2703	16.092	68.9655	68.9655	68.9655	100	62.6506	68.9655	16.092	16.092
NP 001257998.1		100	2.7027	77.027	77.027	77.027	70.2703	100	77.027	2.7027	2.7027
NP 001307344.1			100	69.0722	69.0722	11.3402	14.5833	2.4096	22.6804	100	100
NP 002373.3				100	100	96.1165	71.875	79.5181	72.549	69.0722	69.0722
NP 660087.1					100	87.3786	62.5	68.6747	67.5497	69.0722	69.0722
NP 660088.1						100	71.875	79.5181	96.1165	11.3402	11.3402
NP 660089.1							100	73.494	71.875	14.5833	14.5833
NP 932061.1								100	79.5181	2.4096	2.4096
XP 011535075.1									100	22.6804	22.6804
XP 016876801.1										100	100
XP 016876802.1											100

Table A30. The parameter Alignment Score of the Kalign in the quantitative measurement of homology, assuming the *Jaspar PWMs Resource, JASPAR Predicted Transcription Factor Targets Dataset, MAX Gene Set* [3].

	NP 001257997.1	NP 001257998.1	NP 001307344.1	NP 002373.3	NP 660087.1	NP 660088.1	NP 660089.1	NP 932061.1	XP 011535075.1	XP 016876801.1	XP 016876802.1
NP 001257997.1	469	202	-78.5	183	197	211.5	455	188	186.5	-78.5	-78.5
NP 001257998.1		402	-103.5	177.5	191.5	206	188	388	181	-103.5	-103.5
NP 001307344.1			491	213.5	218	-91	-83	-108	-102.5	491	491
NP 002373.3				809	749	462	243	237.5	455	213.5	213.5
NP 660087.1					763	402	183	177.5	395	218	218
NP 660088.1						529	271.5	266	473.5	-91	-91
NP 660089.1							515	248	246.5	-83	-83
NP 932061.1								448	241	-108	-108
XP 011535075.1									792	-102.5	-102.5
XP 016876801.1										491	491
XP 016876802.1											491

Table A31. The parameter Identity of the MAFFT in the quantitative measurement of homology, assuming the *Jaspar PWMs Resource, JASPAR Predicted Transcription Factor Targets Dataset, MAX Gene Set* [3].

	NP 001257997.1	NP 001257998.1	NP 001307344.1	NP 002373.3	NP 660087.1	NP 660088.1	NP 660089.1	NP 932061.1	XP 011535075.1	XP 016876801.1	XP 016876802.1
NP 001257997.1	100	72.973	11.4943	60.9195	60.9195	60.9195	100	65.0602	60.9195	11.4943	11.4943
NP 001257998.1		100	12.1622	67.5676	67.5676	67.5676	72.973	100	67.5676	12.1622	12.1622
NP 001307344.1			100	54.6392	53.6082	14.433	11.4583	12.0482	17.5258	100	100
NP 002373.3				100	100	99.0291	64.5833	71.0843	71.2418	54.6392	54.6392
NP 660087.1					100	90.2913	55.2083	60.241	66.2252	53.6082	53.6082
NP 660088.1						100	64.5833	71.0843	97.0874	14.433	14.433
NP 660089.1							100	75.9036	64.5833	11.4583	11.4583
NP 932061.1								100	71.0843	12.0482	12.0482
XP 011535075.1									100	17.5258	17.5258
XP 016876801.1										100	100
XP 016876802.1											100

Table A32. The parameter Similarity of the MAFFT in the quantitative measurement of homology, assuming the *Jaspar PWMs Resource, JASPAR Predicted Transcription Factor Targets Dataset, MAX Gene Set* [3].

	NP 001257997.1	NP 001257998.1	NP 001307344.1	NP 002373.3	NP 660087.1	NP 660088.1	NP 660089.1	NP 932061.1	XP 011535075.1	XP 016876801.1	XP 016876802.1
NP 001257997.1	100	78.3784	19.5402	70.1149	70.1149	70.1149	100	69.8795	70.1149	19.5402	19.5402
NP 001257998.1		100	22.973	75.6757	75.6757	75.6757	78.3784	100	75.6757	22.973	22.973
NP 001307344.1			100	62.8866	60.8247	22.6804	19.7917	22.8916	29.8969	100	100
NP 002373.3				100	100	99.0291	72.9167	78.3133	76.4706	62.8866	62.8866
NP 660087.1					100	90.2913	63.5417	67.4699	71.5232	60.8247	60.8247
NP 660088.1						100	72.9167	78.3133	98.0583	22.6804	22.6804
NP 660089.1							100	80.7229	72.9167	19.7917	19.7917
NP 932061.1								100	78.3133	22.8916	22.8916
XP 011535075.1									100	29.8969	29.8969
XP 016876801.1										100	100
XP 016876802.1											100

Table A33. The parameter Alignment Score of the MAFFT in the quantitative measurement of homology, assuming the *Jaspar PWMs Resource, JASPAR Predicted Transcription Factor Targets Dataset, MAX Gene Set* [3].

	NP 001257997.1	NP 001257998.1	NP 001307344.1	NP 002373.3	NP 660087.1	NP 660088.1	NP 660089.1	NP 932061.1	XP 011535075.1	XP 016876801.1	XP 016876802.1
NP 001257997.1	469	226	-62	181.5	195.5	210	455	212	185	-62	-62
NP 001257998.1		402	-57.5	178.5	192.5	207	212	388	182	-57.5	-57.5
NP 001307344.1			491	159.5	159	-54.5	-61.5	-57	-62	491	491
NP 002373.3				809	749	475.5	241.5	238.5	490.5	159.5	159.5
NP 660087.1					763	415.5	181.5	178.5	430.5	159	159
NP 660088.1						529	270	267	470	-54.5	-54.5
NP 660089.1							515	272	245	-61.5	-61.5
NP 932061.1								448	242	-57	-57
XP 011535075.1									792	-62	-62
XP 016876801.1										491	491
XP 016876802.1											491

Table A34. The parameter Sensitivity (SEN) of the HELIOS method with referencing the Smith-Waterman algorithm in the accuracy measurement of classification output, assuming the *Jaspar PWMs Resource, JASPAR Predicted Transcription Factor Targets Dataset, MAX Gene Set* [3].

	NP 001257997.1	NP 001257998.1	NP 001307344.1	NP 002373.3	NP 660087.1	NP 660088.1	NP 660089.1	NP 932061.1	XP 011535075.1	XP 016876801.1	XP 016876802.1
NP 001257997.1	1	0.85135	0	0.65517	0.7931	0.65517	0.86207	0.77027	0.65517	0	0
NP 001257998.1		1	0	0.83784	1	0.83784	0.64865	0.83784	0.83784	0	0
NP 001307344.1			1	0.62887	0.62887	0	0	0	0	1	1
NP 002373.3				1	0.92053	0.97087	0.8125	1	0.64706	0.73196	0.63918
NP 660087.1					1	0.84043	0.65517	0	0.0069444	0.63918	0.63918
NP 660088.1						1	0.8125	1	1	0	0
NP 660089.1							1	0.87952	0.8125	0	0
NP 932061.1								1	1	0	0
XP 011535075.1									1	0	0
XP 016876801.1										1	1
XP 016876802.1											1

Table A35. The parameter Specification (Spec) of the HELIOS method with referencing the Smith-Waterman algorithm in the accuracy measurement of classification output, assuming the *Jaspar PWMs Resource, JASPAR Predicted Transcription Factor Targets Dataset, MAX Gene Set* [3].

	NP 001257997.1	NP 001257998.1	NP 001307344.1	NP 002373.3	NP 660087.1	NP 660088.1	NP 660089.1	NP 932061.1	XP 011535075.1	XP 016876801.1	XP 016876802.1
NP 001257997.1	1	0.9989	0.99151	0.9979	0.9987	0.99673	0.99855	0.99776	0.99781	0.99151	0.99151
NP 001257998.1		1	0.99029	0.99898	1	0.99841	0.99744	0.99802	0.99893	0.99029	0.99029
NP 001307344.1			1	0.99825	0.99828	0.99142	0.99165	0.99084	0.99424	1	1
NP 002373.3				1	0.9995	0.99982	0.99889	1	0.99799	0.9989	0.99831
NP 660087.1					1	0.99903	0.99799	0.99334	0.99368	0.99828	0.99835
NP 660088.1						1	0.99826	1	1	0.99142	0.99142
NP 660089.1							1	0.99924	0.99883	0.99165	0.99165
NP 932061.1								1	1	0.99084	0.99084
XP 011535075.1									1	0.99424	0.99424
XP 016876801.1										1	1
XP 016876802.1											1

Table A36. The parameter Accuracy (Acc) of the HELIOS method with referencing the Smith-Waterman algorithm in the accuracy measurement of classification output, assuming the *Jaspar PWMs Resource, JASPAR Predicted Transcription Factor Targets Dataset, MAX Gene Set* [3].

	NP 001257997.1	NP 001257998.1	NP 001307344.1	NP 002373.3	NP 660087.1	NP 660088.1	NP 660089.1	NP 932061.1	XP 011535075.1	XP 016876801.1	XP 016876802.1
NP 001257997.1	1	0.9972	0.98234	0.99576	0.99734	0.99342	0.99713	0.99543	0.99557	0.98234	0.98234
NP 001257998.1		1	0.98022	0.99797	1	0.99685	0.99381	0.99609	0.99788	0.98022	0.98022
NP 001307344.1			1	0.99594	0.99584	0.98288	0.98239	0.98075	0.98848	1	1
NP 002373.3				1	0.99901	0.99964	0.99772	1	0.99579	0.99723	0.99607
NP 660087.1					1	0.99807	0.99593	0.98747	0.98753	0.9959	0.99597
NP 660088.1						1	0.99646	1	1	0.98288	0.98288
NP 660089.1							1	0.99799	0.99762	0.98239	0.98239
NP 932061.1								1	1	0.98075	0.98075
XP 011535075.1									1	0.98848	0.98848
XP 016876801.1										1	1
XP 016876802.1											1

Table A37. The parameter Positive Predictive Value (PPV) of the HELIOS method with referencing the Smith-Waterman algorithm in the accuracy measurement of classification output, assuming the *Jaspar PWMs Resource, JASPAR Predicted Transcription Factor Targets Dataset, MAX Gene Set* [3].

	NP 001257997.1	NP 001257998.1	NP 001307344.1	NP 002373.3	NP 660087.1	NP 660088.1	NP 660089.1	NP 932061.1	XP 011535075.1	XP 016876801.1	XP 016876802.1
NP 001257997.1	1	0.9	0	0.66279	0.80233	0.66279	0.86207	0.78082	0.66279	0	0
NP 001257998.1		1	0	0.83784	1	0.83784	0.72727	0.83784	0.83784	0	0
NP 001307344.1			1	0.69318	0.7093	0	0	0	0	1	1
NP 002373.3				1	0.92053	0.97087	0.82105	1	0.66892	0.80682	0.70455
NP 660087.1					1	0.84043	0.66279	0	0.0068493	0.71264	0.72093
NP 660088.1						1	0.82105	1	1	0	0
NP 660089.1							1	0.92405	0.82105	0	0
NP 932061.1								1	1	0	0
XP 011535075.1									1	0	0
XP 016876801.1										1	1
XP 016876802.1											1

Table A38. The parameter Negative Predictive Value (NPV) of the HELIOS method with referencing the Smith-Waterman algorithm in the accuracy measurement of classification output, assuming the *Jaspar PWMs Resource, JASPAR Predicted Transcription Factor Targets Dataset, MAX Gene Set* [3].

	NP 001257997.1	NP 001257998.1	NP 001307344.1	NP 002373.3	NP 660087.1	NP 660088.1	NP 660089.1	NP 932061.1	XP 011535075.1	XP 016876801.1	XP 016876802.1
NP 001257997.1	1	0.99827	0.99068	0.99783	0.99862	0.99662	0.99855	0.99762	0.99773	0.99068	0.99068
NP 001257998.1		1	0.98973	0.99898	1	0.99841	0.99631	0.99802	0.99893	0.98973	0.98973
NP 001307344.1			1	0.99767	0.99753	0.99132	0.99058	0.98972	0.99417	1	1
NP 002373.3				1	0.9995	0.99982	0.99882	1	0.99778	0.99832	0.99773
NP 660087.1					1	0.99903	0.99792	0.99406	0.99377	0.9976	0.9976
NP 660088.1						1	0.99816	1	1	0.99132	0.99132
NP 660089.1							1	0.99873	0.99877	0.99058	0.99058
NP 932061.1								1	1	0.98972	0.98972
XP 011535075.1									1	0.99417	0.99417
XP 016876801.1										1	1
XP 016876802.1											1

Table A39. The parameter Matthew's Coefficient Correlation (MCC) of the HELIOS method with referencing the Smith-Waterman algorithm in the accuracy measurement of classification output, assuming the *Jaspar PWMs Resource, JASPAR Predicted Transcription Factor Targets Dataset, MAX Gene Set* [3].

	NP 001257997.1	NP 001257998.1	NP 001307344.1	NP 002373.3	NP 660087.1	NP 660088.1	NP 660089.1	NP 932061.1	XP 011535075.1	XP 016876801.1	XP 016876802.1
NP 001257997.1	1	0.87393	-0.0088969	0.65684	0.79636	0.65565	0.86062	0.77322	0.65674	-0.0088969	-0.0088969
NP 001257998.1		1	-0.0099862	0.83682	1	0.83625	0.68374	0.83586	0.83677	-0.0099862	-0.0099862
NP 001307344.1			1	0.65821	0.6658	-0.0086314	-0.0088675	-0.0097033	-0.0057944	1	1
NP 002373.3				1	0.92003	0.97069	0.81562	1	0.65578	0.7671	0.6691
NP 660087.1					1	0.83946	0.65692	-0.0062926	0.00062454	0.67287	0.67682
NP 660088.1						1	0.81498	1	1	-0.0086314	-0.0086314
NP 660089.1							1	0.9005	0.81557	-0.0088675	-0.0088675
NP 932061.1								1	1	-0.0097033	-0.0097033
XP 011535075.1									1	-0.0057944	-0.0057944
XP 016876801.1										1	1
XP 016876802.1											1

Table A40. The parameter Test's Accuracy (F-Score) of the HELIOS method with referencing the Smith-Waterman algorithm in the accuracy measurement of classification output, assuming the *Jaspar PWMs Resource, JASPAR Predicted Transcription Factor Targets Dataset, MAX Gene Set* [3].

	NP 001257997.1	NP 001257998.1	NP 001307344.1	NP 002373.3	NP 660087.1	NP 660088.1	NP 660089.1	NP 932061.1	XP 011535075.1	XP 016876801.1	XP 016876802.1
NP 001257997.1	1	0.875	0	0.65896	0.79769	0.65896	0.86207	0.77551	0.65896	0	0
NP 001257998.1		1	0	0.83784	1	0.83784	0.68571	0.83784	0.83784	0	0
NP 001307344.1			1	0.65946	0.66667	0	0	0	0	1	1
NP 002373.3				1	0.92053	0.97087	0.81675	1	0.65781	0.76757	0.67027
NP 660087.1					1	0.84043	0.65896	0	0.0068966	0.67391	0.6776
NP 660088.1						1	0.81675	1	1	0	0
NP 660089.1							1	0.90123	0.81675	0	0
NP 932061.1								1	1	0	0
XP 011535075.1									1	0	0
XP 016876801.1										1	1
XP 016876802.1											1

Table A41. The parameter Sensitivity (SEN) of the HELIOS method with referencing the Needleman-Wunsch algorithm in the accuracy measurement of classification output, assuming the *Jaspar PWMs Resource, JASPAR Predicted Transcription Factor Targets Dataset, MAX Gene Set* [3].

	NP 001257997.1	NP 001257998.1	NP 001307344.1	NP 002373.3	NP 660087.1	NP 660088.1	NP 660089.1	NP 932061.1	XP 011535075.1	XP 016876801.1	XP 016876802.1
NP 001257997.1	1	0.92308	0.34483	0.25532	1	0.25532	0.13953	0.1875	0.25532	0.34483	0.34483
NP 001257998.1		1	0.34483	0.22642	1	0.22642	0.1875	0.16438	0.22642	0.34483	0.34483
NP 001307344.1			1	0.16923	0.16923	0.27027	0.14286	0.35714	0.35135	1	1
NP 002373.3				1	0.08	1	1	1	1	0.16923	0.16923
NP 660087.1					1	0.13636	0.25532	1	1	0.16923	0.16923
NP 660088.1						1	1	1	1	0.27027	0.27027
NP 660089.1							1	0.94595	1	0.14286	0.14286
NP 932061.1								1	1	0.35714	0.35714
XP 011535075.1									1	0.35135	0.35135
XP 016876801.1										1	1
XP 016876802.1											1

Table A42. The parameter Specification (Spec) of the HELIOS method with referencing the Needleman-Wunsch algorithm in the accuracy measurement of classification output, assuming the *Jaspar PWMs Resource, JASPAR Predicted Transcription Factor Targets Dataset, MAX Gene Set* [3].

	NP 001257997.1	NP 001257998.1	NP 001307344.1	NP 002373.3	NP 660087.1	NP 660088.1	NP 660089.1	NP 932061.1	XP 011535075.1	XP 016876801.1	XP 016876802.1
NP 001257997.1	1	0.99905	0.98507	0.98797	1	0.98797	0.99111	0.9895	0.98797	0.98507	0.98507
NP 001257998.1		1	0.98507	0.98839	1	0.98839	0.99072	0.99011	0.98839	0.98507	0.98507
NP 001307344.1			1	0.99615	0.99663	0.98754	0.98278	0.98893	0.98962	1	1
NP 002373.3				1	0.99423	1	1	1	1	0.99615	0.99639
NP 660087.1					1	0.99125	0.98797	1	1	0.99663	0.99687
NP 660088.1						1	1	1	1	0.98754	0.98754
NP 660089.1							1	0.99945	1	0.98278	0.98278
NP 932061.1								1	1	0.98893	0.98893
XP 011535075.1									1	0.98962	0.98962
XP 016876801.1										1	1
XP 016876802.1											1

Table A43. The parameter Accuracy (Acc) of the HELIOS method with referencing the Needleman-Wunsch algorithm in the accuracy measurement of classification output, assuming the *Jaspar PWMs Resource*, *JASPAR Predicted Transcription Factor Targets Dataset*, *MAX Gene Set* [3].

	NP 001257997.1	NP 001257998.1	NP 001307344.1	NP 002373.3	NP 660087.1	NP 660088.1	NP 660089.1	NP 932061.1	XP 011535075.1	XP 016876801.1	XP 016876802.1
NP 001257997.1	1	0.9979	0.96444	0.97239	1	0.97239	0.98121	0.97716	0.97239	0.96444	0.96444
NP 001257998.1		1	0.96444	0.97401	1	0.97401	0.97837	0.9788	0.97401	0.96444	0.96444
NP 001307344.1			1	0.98343	0.98391	0.96964	0.95482	0.9679	0.97368	1	1
NP 002373.3				1	0.98813	1	1	1	1	0.98343	0.98367
NP 660087.1					1	0.98153	0.97239	1	1	0.98391	0.98414
NP 660088.1						1	1	1	1	0.96964	0.96964
NP 660089.1							1	0.99874	1	0.95482	0.95482
NP 932061.1								1	1	0.9679	0.9679
XP 011535075.1									1	0.97368	0.97368
XP 016876801.1										1	1
XP 016876802.1											1

Table A44. The parameter Positive Predictive Value (PPV) of the HELIOS method with referencing the Needleman-Wunsch algorithm in the accuracy measurement of classification output, assuming the *Jaspar PWMs Resource*, *JASPAR Predicted Transcription Factor Targets Dataset*, *MAX Gene Set* [3].

	NP 001257997.1	NP 001257998.1	NP 001307344.1	NP 002373.3	NP 660087.1	NP 660088.1	NP 660089.1	NP 932061.1	XP 011535075.1	XP 016876801.1	XP 016876802.1
NP 001257997.1	1	0.9375	0.43478	0.31579	1	0.31579	0.15584	0.21818	0.31579	0.43478	0.43478
NP 001257998.1		1	0.43478	0.27273	1	0.27273	0.24	0.1875	0.27273	0.43478	0.43478
NP 001307344.1			1	0.40741	0.44	0.35714	0.22222	0.52632	0.46429	1	1
NP 002373.3				1	0.085106	1	1	1	1	0.40741	0.42308
NP 660087.1					1	0.1519	0.31579	1	1	0.44	0.45833
NP 660088.1						1	1	1	1	0.35714	0.35714
NP 660089.1							1	0.9589	1	0.22222	0.22222
NP 932061.1								1	1	0.52632	0.52632
XP 011535075.1									1	0.46429	0.46429
XP 016876801.1										1	1
XP 016876802.1											1

Table A45. The parameter Negative Predictive Value (NPV) of the HELIOS method with referencing the Needleman-Wunsch algorithm in the accuracy measurement of classification output, assuming the *Jaspar PWMs Resource, JASPAR Predicted Transcription Factor Targets Dataset, MAX Gene Set* [3].

	NP 001257997.1	NP 001257998.1	NP 001307344.1	NP 002373.3	NP 660087.1	NP 660088.1	NP 660089.1	NP 932061.1	XP 011535075.1	XP 016876801.1	XP 016876802.1
NP 001257997.1	1	0.99882	0.97834	0.98388	1	0.98388	0.98989	0.98733	0.98388	0.97834	0.97834
NP 001257998.1		1	0.97834	0.98517	1	0.98517	0.98735	0.98841	0.98517	0.97834	0.97834
NP 001307344.1			1	0.98714	0.98714	0.98143	0.97084	0.9781	0.98349	1	1
NP 002373.3				1	0.99383	1	1	1	1	0.98714	0.98714
NP 660087.1					1	0.99008	0.98388	1	1	0.98714	0.98715
NP 660088.1						1	1	1	1	0.98143	0.98143
NP 660089.1							1	0.99927	1	0.97084	0.97084
NP 932061.1								1	1	0.9781	0.9781
XP 011535075.1									1	0.98349	0.98349
XP 016876801.1										1	1
XP 016876802.1											1

Table A46. The parameter Matthew's Coefficient Correlation (MCC) of the HELIOS method with referencing the Needleman-Wunsch algorithm in the accuracy measurement of classification output, assuming the *Jaspar PWMs Resource, JASPAR Predicted Transcription Factor Targets Dataset, MAX Gene Set* [3].

	NP 001257997.1	NP 001257998.1	NP 001307344.1	NP 002373.3	NP 660087.1	NP 660088.1	NP 660089.1	NP 932061.1	XP 011535075.1	XP 016876801.1	XP 016876802.1
NP 001257997.1	1	0.9292	0.36917	0.27001	1	0.27001	0.13798	0.19073	0.27001	0.36917	0.36917
NP 001257998.1		1	0.36917	0.23537	1	0.23537	0.20129	0.16485	0.23537	0.36917	0.36917
NP 001307344.1			1	0.25544	0.26617	0.29545	0.15574	0.41781	0.39074	1	1
NP 002373.3				1	0.076545	1	1	1	1	0.25544	0.26066
NP 660087.1					1	0.13461	0.27001	1	1	0.26617	0.27202
NP 660088.1						1	1	1	1	0.29545	0.29545
NP 660089.1							1	0.95176	1	0.15574	0.15574
NP 932061.1								1	1	0.41781	0.41781
XP 011535075.1									1	0.39074	0.39074
XP 016876801.1										1	1
XP 016876802.1											1

Table A47. The parameter Test's Accuracy (F-Score) of the HELIOS method with referencing the Needleman-Wunsch algorithm in the accuracy measurement of classification output, assuming the *Jaspar PWMs Resource, JASPAR Predicted Transcription Factor Targets Dataset, MAX Gene Set* [3].

	NP 001257997.1	NP 001257998.1	NP 001307344.1	NP 002373.3	NP 660087.1	NP 660088.1	NP 660089.1	NP 932061.1	XP 011535075.1	XP 016876801.1	XP 016876802.1
NP 001257997.1	1	0.93023	0.38462	0.28235	1	0.28235	0.14724	0.20168	0.28235	0.38462	0.38462
NP 001257998.1		1	0.38462	0.24742	1	0.24742	0.21053	0.17518	0.24742	0.38462	0.38462
NP 001307344.1			1	0.23913	0.24444	0.30769	0.17391	0.42553	0.4	1	1
NP 002373.3				1	0.082474	1	1	1	1	0.23913	0.24176
NP 660087.1					1	0.14371	0.28235	1	1	0.24444	0.24719
NP 660088.1						1	1	1	1	0.30769	0.30769
NP 660089.1							1	0.95238	1	0.17391	0.17391
NP 932061.1								1	1	0.42553	0.42553
XP 011535075.1									1	0.4	0.4
XP 016876801.1										1	1
XP 016876802.1											1

Table A48. The parameter Sensitivity (SEN) of the HELIOS method with referencing the BLAST in the accuracy measurement of classification output, assuming the *Jaspar PWMs Resource, JASPAR Predicted Transcription Factor Targets Dataset, MAX Gene Set* [3].

	NP 001257997.1	NP 001257998.1	NP 001307344.1	NP 002373.3	NP 660087.1	NP 660088.1	NP 660089.1	NP 932061.1	XP 011535075.1	XP 016876801.1	XP 016876802.1
NP 001257997.1	1	0.78571	0	0.76471	0.76471	0.76471	1	0.74286	0.76471	0	0
NP 001257998.1		1	0	0.83099	0.83099	0.83099	0.72857	1	0.83099	0	0
NP 001307344.1			1	0.63542	0.63542	0	0	0	0	1	1
NP 002373.3				1	1	0.9703	0.64894	0.85	0.64901	0.63542	0.64583
NP 660087.1					1	0.96739	0.64706	0.16901	0.084507	0.64583	0.64583
NP 660088.1						1	0.78723	0.85	1	0	0
NP 660089.1							1	0.78481	0.78723	0	0
NP 932061.1								1	0.85	0	0
XP 011535075.1									1	0	0
XP 016876801.1										1	1
XP 016876802.1											1

Table A49. The parameter Specification (Spec) of the HELIOS method with referencing the BLAST in the accuracy measurement of classification output, assuming the *Jaspar PWMs Resource, JASPAR Predicted Transcription Factor Targets Dataset, MAX Gene Set* [3].

	NP 001257997.1	NP 001257998.1	NP 001307344.1	NP 002373.3	NP 660087.1	NP 660088.1	NP 660089.1	NP 932061.1	XP 011535075.1	XP 016876801.1	XP 016876802.1
NP 001257997.1	1	0.99764	0.99155	0.99848	0.99839	0.99763	1	0.99706	0.99841	0.99155	0.99155
NP 001257998.1		1	0.99036	0.99873	0.99865	0.99801	0.99787	1	0.99867	0.99036	0.99036
NP 001307344.1			1	0.99825	0.99828	0.99146	0.9917	0.99091	0.99424	1	1
NP 002373.3				1	1	0.99969	0.99777	0.99886	0.99794	0.99825	0.99831
NP 660087.1					1	0.99968	0.99785	0.9943	0.99416	0.99828	0.99835
NP 660088.1						1	0.99786	0.99823	1	0.99146	0.99146
NP 660089.1							1	0.99785	0.99856	0.9917	0.9917
NP 932061.1								1	0.99881	0.99091	0.99091
XP 011535075.1									1	0.99424	0.99424
XP 016876801.1										1	1
XP 016876802.1											1

Table A50. The parameter Accuracy (Acc) of the HELIOS method with referencing the BLAST in the accuracy measurement of classification output, assuming the *Jaspar PWMs Resource, JASPAR Predicted Transcription Factor Targets Dataset, MAX Gene Set* [3].

	NP 001257997.1	NP 001257998.1	NP 001307344.1	NP 002373.3	NP 660087.1	NP 660088.1	NP 660089.1	NP 932061.1	XP 011535075.1	XP 016876801.1	XP 016876802.1
NP 001257997.1	1	0.99534	0.98744	0.99705	0.99688	0.99542	1	0.9946	0.99692	0.98744	0.98744
NP 001257998.1		1	0.98732	0.99772	0.99758	0.99646	0.99521	1	0.99762	0.98732	0.98732
NP 001307344.1			1	0.99601	0.9959	0.98779	0.98797	0.9882	0.98841	1	1
NP 002373.3				1	1	0.99951	0.99564	0.99797	0.99579	0.99601	0.99613
NP 660087.1					1	0.99949	0.99579	0.98963	0.98857	0.99597	0.99604
NP 660088.1						1	0.99585	0.99684	1	0.98779	0.98779
NP 660089.1							1	0.99573	0.99721	0.98797	0.98797
NP 932061.1								1	0.99787	0.9882	0.9882
XP 011535075.1									1	0.98841	0.98841
XP 016876801.1										1	1
XP 016876802.1											1

Table A51. The parameter Positive Predictive Value (PPV) of the HELIOS method with referencing the BLAST in the accuracy measurement of classification output, assuming the *Jaspar PWMs Resource, JASPAR Predicted Transcription Factor Targets Dataset, MAX Gene Set* [3].

	NP 001257997.1	NP 001257998.1	NP 001307344.1	NP 002373.3	NP 660087.1	NP 660088.1	NP 660089.1	NP 932061.1	XP 011535075.1	XP 016876801.1	XP 016876802.1
NP 001257997.1	1	0.78571	0	0.75581	0.75581	0.75581	1	0.71233	0.75581	0	0
NP 001257998.1		1	0	0.7973	0.7973	0.7973	0.77273	1	0.7973	0	0
NP 001307344.1			1	0.69318	0.7093	0	0	0	0	1	1
NP 002373.3				1	1	0.95146	0.64211	0.81928	0.66216	0.69318	0.70455
NP 660087.1					1	0.94681	0.63953	0.14458	0.082192	0.71264	0.72093
NP 660088.1						1	0.77895	0.81928	1	0	0
NP 660089.1							1	0.78481	0.77895	0	0
NP 932061.1								1	0.81928	0	0
XP 011535075.1									1	0	0
XP 016876801.1										1	1
XP 016876802.1											1

Table A52. The parameter Negative Predictive Value (NPV) of the HELIOS method with referencing the BLAST in the accuracy measurement of classification output, assuming the *Jaspar PWMs Resource, JASPAR Predicted Transcription Factor Targets Dataset, MAX Gene Set* [3].

	NP 001257997.1	NP 001257998.1	NP 001307344.1	NP 002373.3	NP 660087.1	NP 660088.1	NP 660089.1	NP 932061.1	XP 011535075.1	XP 016876801.1	XP 016876802.1
NP 001257997.1	1	0.99764	0.99582	0.99855	0.99847	0.99775	1	0.99748	0.99849	0.99582	0.99582
NP 001257998.1		1	0.99691	0.99898	0.99892	0.99841	0.9973	1	0.99893	0.99691	0.99691
NP 001307344.1			1	0.99773	0.9976	0.99626	0.99621	0.99724	0.9941	1	1
NP 002373.3				1	1	0.99982	0.99784	0.99909	0.99782	0.99773	0.9978
NP 660087.1					1	0.99981	0.99792	0.99526	0.99434	0.99766	0.99766
NP 660088.1						1	0.99796	0.99858	1	0.99626	0.99626
NP 660089.1							1	0.99785	0.99863	0.99621	0.99621
NP 932061.1								1	0.99905	0.99724	0.99724
XP 011535075.1									1	0.9941	0.9941
XP 016876801.1										1	1
XP 016876802.1											1

Table A53. The parameter Matthew's Coefficient Correlation (MCC) of the HELIOS method with referencing the BLAST in the accuracy measurement of classification output, assuming the *Jaspar PWMs Resource, JASPAR Predicted Transcription Factor Targets Dataset, MAX Gene Set* [3].

	NP 001257997.1	NP 001257998.1	NP 001307344.1	NP 002373.3	NP 660087.1	NP 660088.1	NP 660089.1	NP 932061.1	XP 011535075.1	XP 016876801.1	XP 016876802.1
NP 001257997.1	1	0.78336	-0.0059444	0.75877	0.75868	0.75794	1	0.72471	0.7587	-0.0059444	-0.0059444
NP 001257998.1		1	-0.0054626	0.81282	0.81275	0.81218	0.74792	1	0.81277	-0.0054626	-0.0054626
NP 001307344.1			1	0.66167	0.6693	-0.0056476	-0.0056086	-0.0050072	-0.0058281	1	1
NP 002373.3				1	1	0.96059	0.64332	0.83348	0.65344	0.66167	0.67262
NP 660087.1					1	0.95679	0.64117	0.15112	0.077593	0.6764	0.68037
NP 660088.1						1	0.78099	0.83291	1	-0.0056476	-0.0056476
NP 660089.1							1	0.78266	0.78168	-0.0056086	-0.0056086
NP 932061.1								1	0.83343	-0.0050072	-0.0050072
XP 011535075.1									1	-0.0058281	-0.0058281
XP 016876801.1										1	1
XP 016876802.1											1

Table A54. The parameter Test's Accuracy (F-Score) of the HELIOS method with referencing the BLAST in the accuracy measurement of classification output, assuming the *Jaspar PWMs Resource, JASPAR Predicted Transcription Factor Targets Dataset, MAX Gene Set* [3].

	NP 001257997.1	NP 001257998.1	NP 001307344.1	NP 002373.3	NP 660087.1	NP 660088.1	NP 660089.1	NP 932061.1	XP 011535075.1	XP 016876801.1	XP 016876802.1
NP 001257997.1	1	0.78571	0	0.76023	0.76023	0.76023	1	0.72727	0.76023	0	0
NP 001257998.1		1	0	0.81379	0.81379	0.81379	0.75	1	0.81379	0	0
NP 001307344.1			1	0.66304	0.67033	0	0	0	0	1	1
NP 002373.3				1	1	0.96078	0.6455	0.83436	0.65552	0.66304	0.67391
NP 660087.1					1	0.95699	0.64327	0.15584	0.083333	0.6776	0.68132
NP 660088.1						1	0.78307	0.83436	1	0	0
NP 660089.1							1	0.78481	0.78307	0	0
NP 932061.1								1	0.83436	0	0
XP 011535075.1									1	0	0
XP 016876801.1										1	1
XP 016876802.1											1

Table A55. The parameter Sensitivity (SEN) of the HELIOS method with referencing the ClustalW in the accuracy measurement of classification output, assuming the *Jaspar PWMs Resource, JASPAR Predicted Transcription Factor Targets Dataset, MAX Gene Set* [3].

	NP 001257997.1	NP 001257998.1	NP 001307344.1	NP 002373.3	NP 660087.1	NP 660088.1	NP 660089.1	NP 932061.1	XP 011535075.1	XP 016876801.1	XP 016876802.1
NP 001257997.1	1	0.82432	0	0.75581	0.75581	0.75581	1	0.82432	0.75581	0	0
NP 001257998.1		1	0	0.78082	0.78082	0.78082	0.77027	1	0.78082	0	0
NP 001307344.1			1	0	0.023529	0.17021	0.13684	0.13253	0.26596	1	1
NP 002373.3				1	1	0.96117	0.64211	0.80488	0.64706	0	0
NP 660087.1					1	0.95745	0.63953	0.16438	0.083333	0	0.023529
NP 660088.1						1	0.77895	0.80488	0.96117	0.17021	0.17021
NP 660089.1							1	0.84337	0.77895	0.13684	0.13684
NP 932061.1								1	0.80488	0.13253	0.13253
XP 011535075.1									1	0.26596	0.26596
XP 016876801.1										1	1
XP 016876802.1											1

Table A56. The parameter Specification (Spec) of the HELIOS method with referencing the ClustalW in the accuracy measurement of classification output, assuming the *Jaspar PWMs Resource, JASPAR Predicted Transcription Factor Targets Dataset, MAX Gene Set* [3].

	NP 001257997.1	NP 001257998.1	NP 001307344.1	NP 002373.3	NP 660087.1	NP 660088.1	NP 660089.1	NP 932061.1	XP 011535075.1	XP 016876801.1	XP 016876802.1
NP 001257997.1	1	0.99859	0.9915	0.99848	0.99839	0.99763	1	0.99832	0.99841	0.9915	0.9915
NP 001257998.1		1	0.99029	0.99856	0.99847	0.99775	0.99872	1	0.99849	0.99029	0.99029
NP 001307344.1			1	0.9943	0.99423	0.99303	0.99306	0.99222	0.99593	1	1
NP 002373.3				1	1	0.99976	0.99777	0.99871	0.99799	0.9943	0.9943
NP 660087.1					1	0.99974	0.99785	0.9943	0.99416	0.99403	0.99423
NP 660088.1						1	0.99786	0.99799	0.99974	0.99303	0.99303
NP 660089.1							1	0.99886	0.99856	0.99306	0.99306
NP 932061.1								1	0.99865	0.99222	0.99222
XP 011535075.1									1	0.99593	0.99593
XP 016876801.1										1	1
XP 016876802.1											1

Table A57. The parameter Accuracy (Acc) of the HELIOS method with referencing the ClustalW in the accuracy measurement of classification output, assuming the *Jaspar PWMs Resource, JASPAR Predicted Transcription Factor Targets Dataset, MAX Gene Set* [3].

	NP 001257997.1	NP 001257998.1	NP 001307344.1	NP 002373.3	NP 660087.1	NP 660088.1	NP 660089.1	NP 932061.1	XP 011535075.1	XP 016876801.1	XP 016876802.1
NP 001257997.1	1	0.99658	0.9814	0.99698	0.9968	0.99531	1	0.99654	0.99684	0.9814	0.9814
NP 001257998.1		1	0.98008	0.99721	0.99705	0.99567	0.99634	1	0.99709	0.98008	0.98008
NP 001307344.1			1	0.98827	0.9886	0.98529	0.98432	0.98336	0.99131	1	1
NP 002373.3				1	1	0.99951	0.99557	0.99752	0.99579	0.98827	0.98827
NP 660087.1					1	0.99949	0.99572	0.98947	0.98849	0.98826	0.9886
NP 660088.1						1	0.99575	0.99614	0.99949	0.98529	0.98529
NP 660089.1							1	0.99724	0.99714	0.98432	0.98432
NP 932061.1								1	0.9974	0.98336	0.98336
XP 011535075.1									1	0.99131	0.99131
XP 016876801.1										1	1
XP 016876802.1											1

Table A58. The parameter Positive Predictive Value (PPV) of the HELIOS method with referencing the ClustalW in the accuracy measurement of classification output, assuming the *Jaspar PWMs Resource, JASPAR Predicted Transcription Factor Targets Dataset, MAX Gene Set* [3].

	NP 001257997.1	NP 001257998.1	NP 001307344.1	NP 002373.3	NP 660087.1	NP 660088.1	NP 660089.1	NP 932061.1	XP 011535075.1	XP 016876801.1	XP 016876802.1
NP 001257997.1	1	0.87143	0	0.75581	0.75581	0.75581	1	0.83562	0.75581	0	0
NP 001257998.1		1	0	0.77027	0.77027	0.77027	0.86364	1	0.77027	0	0
NP 001307344.1			1	0	0.023256	0.18824	0.16883	0.15068	0.29412	1	1
NP 002373.3				1	1	0.96117	0.64211	0.79518	0.66892	0	0
NP 660087.1					1	0.95745	0.63953	0.14458	0.082192	0	0.023256
NP 660088.1						1	0.77895	0.79518	0.96117	0.18824	0.18824
NP 660089.1							1	0.88608	0.77895	0.16883	0.16883
NP 932061.1								1	0.79518	0.15068	0.15068
XP 011535075.1									1	0.29412	0.29412
XP 016876801.1										1	1
XP 016876802.1											1

Table A59. The parameter Negative Predictive Value (NPV) of the HELIOS method with referencing the ClustalW in the accuracy measurement of classification output, assuming the *Jaspar PWMs Resource, JASPAR Predicted Transcription Factor Targets Dataset, MAX Gene Set* [3].

	NP 001257997.1	NP 001257998.1	NP 001307344.1	NP 002373.3	NP 660087.1	NP 660088.1	NP 660089.1	NP 932061.1	XP 011535075.1	XP 016876801.1	XP 016876802.1
NP 001257997.1	1	0.99796	0.98972	0.99848	0.99839	0.99763	1	0.99818	0.99841	0.98972	0.98972
NP 001257998.1		1	0.98959	0.99864	0.99856	0.99788	0.99758	1	0.99858	0.98959	0.98959
NP 001307344.1			1	0.99391	0.9943	0.99213	0.99112	0.99098	0.99532	1	1
NP 002373.3				1	1	0.99976	0.99777	0.99879	0.99778	0.99391	0.99391
NP 660087.1					1	0.99974	0.99785	0.9951	0.99425	0.99416	0.9943
NP 660088.1						1	0.99786	0.99811	0.99974	0.99213	0.99213
NP 660089.1							1	0.99835	0.99856	0.99112	0.99112
NP 932061.1								1	0.99873	0.99098	0.99098
XP 011535075.1									1	0.99532	0.99532
XP 016876801.1										1	1
XP 016876802.1											1

Table A60. The parameter Matthew's Coefficient Correlation (MCC) of the HELIOS method with referencing the ClustalW in the accuracy measurement of classification output, assuming the *Jaspar PWMs Resource, JASPAR Predicted Transcription Factor Targets Dataset, MAX Gene Set* [3].

	NP 001257997.1	NP 001257998.1	NP 001307344.1	NP 002373.3	NP 660087.1	NP 660088.1	NP 660089.1	NP 932061.1	XP 011535075.1	XP 016876801.1	XP 016876802.1
NP 001257997.1	1	0.84583	-0.0093464	0.7543	0.7542	0.75345	1	0.8282	0.75423	-0.0093464	-0.0093464
NP 001257998.1		1	-0.010055	0.77413	0.77404	0.77334	0.8138	1	0.77406	-0.010055	-0.010055
NP 001307344.1			1	-0.0058948	0.017658	0.17159	0.14414	0.13294	0.27532	1	1
NP 002373.3				1	1	0.96092	0.63988	0.79876	0.65578	-0.0058948	-0.0058948
NP 660087.1					1	0.95719	0.63738	0.14888	0.076968	-0.0059058	0.017658
NP 660088.1						1	0.7768	0.79807	0.96091	0.17159	0.17159
NP 660089.1							1	0.86307	0.77751	0.14414	0.14414
NP 932061.1								1	0.79871	0.13294	0.13294
XP 011535075.1									1	0.27532	0.27532
XP 016876801.1										1	1
XP 016876802.1											1

Table A61. The parameter Test's Accuracy (F-Score) of the HELIOS method with referencing the ClustalW in the accuracy measurement of classification output, assuming the *Jaspar PWMs Resource, JASPAR Predicted Transcription Factor Targets Dataset, MAX Gene Set* [3].

	NP 001257997.1	NP 001257998.1	NP 001307344.1	NP 002373.3	NP 660087.1	NP 660088.1	NP 660089.1	NP 932061.1	XP 011535075.1	XP 016876801.1	XP 016876802.1
NP 001257997.1	1	0.84722	0	0.75581	0.75581	0.75581	1	0.82993	0.75581	0	0
NP 001257998.1		1	0	0.77551	0.77551	0.77551	0.81429	1	0.77551	0	0
NP 001307344.1			1	0	0.023392	0.17877	0.15116	0.14103	0.27933	1	1
NP 002373.3				1	1	0.96117	0.64211	0.8	0.65781	0	0
NP 660087.1					1	0.95745	0.63953	0.15385	0.082759	0	0.023392
NP 660088.1						1	0.77895	0.8	0.96117	0.17877	0.17877
NP 660089.1							1	0.8642	0.77895	0.15116	0.15116
NP 932061.1								1	0.8	0.14103	0.14103
XP 011535075.1									1	0.27933	0.27933
XP 016876801.1										1	1
XP 016876802.1											1

Table A62. The parameter Sensitivity (SEN) of the HELIOS method with referencing the ClustalΩ in the accuracy measurement of classification output, assuming the *Jaspar PWMs Resource, JASPAR Predicted Transcription Factor Targets Dataset, MAX Gene Set* [3].

	NP 001257997.1	NP 001257998.1	NP 001307344.1	NP 002373.3	NP 660087.1	NP 660088.1	NP 660089.1	NP 932061.1	XP 011535075.1	XP 016876801.1	XP 016876802.1
NP 001257997.1	1	0.76389	0	0.68675	0.6988	0.68675	0.98851	0.70833	0.65517	0	0
NP 001257998.1		1	0	0.78378	0.7973	0.78378	0.69444	0.98649	0.78378	0	0
NP 001307344.1			1	0.61628	0.61628	0	0	0	0	1	1
NP 002373.3				1	0.99338	0.97087	0.72826	0.81928	0.69718	0.61628	0.61628
NP 660087.1					1	0.95745	0.75904	0.27027	0.082707	0.61628	0.61628
NP 660088.1						1	0.72826	0.81928	1	0	0
NP 660089.1							1	0.79012	0.69792	0	0
NP 932061.1								1	0.81928	0	0
XP 011535075.1									1	0	0
XP 016876801.1										1	1
XP 016876802.1											1

Table A63. The parameter Specification (Spec) of the HELIOS method with referencing the ClustalΩ in the accuracy measurement of classification output, assuming the *Jaspar PWMs Resource, JASPAR Predicted Transcription Factor Targets Dataset, MAX Gene Set* [3].

	NP 001257997.1	NP 001257998.1	NP 001307344.1	NP 002373.3	NP 660087.1	NP 660088.1	NP 660089.1	NP 932061.1	XP 011535075.1	XP 016876801.1	XP 016876802.1
NP 001257997.1	1	0.99764	0.99156	0.9979	0.99786	0.99673	0.99988	0.99692	0.99781	0.99156	0.99156
NP 001257998.1		1	0.99037	0.99864	0.99865	0.99788	0.99772	0.99984	0.99858	0.99037	0.99037
NP 001307344.1			1	0.99773	0.99773	0.99147	0.99171	0.99092	0.99424	1	1
NP 002373.3				1	0.99996	0.99982	0.99817	0.99886	0.99799	0.99773	0.99773
NP 660087.1					1	0.99974	0.9984	0.99494	0.99412	0.99766	0.99773
NP 660088.1						1	0.99714	0.99823	1	0.99147	0.99147
NP 660089.1							1	0.9981	0.99808	0.99171	0.99171
NP 932061.1								1	0.99881	0.99092	0.99092
XP 011535075.1									1	0.99424	0.99424
XP 016876801.1										1	1
XP 016876802.1											1

Table A64. The parameter Accuracy (Acc) of the HELIOS method with referencing the ClustalΩ in the accuracy measurement of classification output, assuming the *Jaspar PWMs Resource, JASPAR Predicted Transcription Factor Targets Dataset, MAX Gene Set* [3].

	NP 001257997.1	NP 001257998.1	NP 001307344.1	NP 002373.3	NP 660087.1	NP 660088.1	NP 660089.1	NP 932061.1	XP 011535075.1	XP 016876801.1	XP 016876802.1
NP 001257997.1	1	0.99503	0.98851	0.99605	0.99597	0.99386	0.99976	0.99405	0.99557	0.98851	0.98851
NP 001257998.1		1	0.98872	0.9973	0.99732	0.9958	0.99465	0.99967	0.99717	0.98872	0.98872
NP 001307344.1			1	0.99562	0.99549	0.98859	0.98894	0.98944	0.98895	1	1
NP 002373.3				1	0.99992	0.99964	0.99655	0.99774	0.99624	0.99562	0.99562
NP 660087.1					1	0.99949	0.99703	0.99066	0.98888	0.99543	0.99549
NP 660088.1						1	0.99464	0.99649	1	0.98859	0.98859
NP 660089.1							1	0.99598	0.99612	0.98894	0.98894
NP 932061.1								1	0.99764	0.98944	0.98944
XP 011535075.1									1	0.98895	0.98895
XP 016876801.1										1	1
XP 016876802.1											1

Table A65. The parameter Positive Predictive Value (PPV) of the HELIOS method with referencing the ClustalΩ in the accuracy measurement of classification output, assuming the *Jaspar PWMs Resource, JASPAR Predicted Transcription Factor Targets Dataset, MAX Gene Set* [3].

	NP 001257997.1	NP 001257998.1	NP 001307344.1	NP 002373.3	NP 660087.1	NP 660088.1	NP 660089.1	NP 932061.1	XP 011535075.1	XP 016876801.1	XP 016876802.1
NP 001257997.1	1	0.78571	0	0.66279	0.67442	0.66279	0.98851	0.69863	0.66279	0	0
NP 001257998.1		1	0	0.78378	0.7973	0.78378	0.75758	0.98649	0.78378	0	0
NP 001307344.1			1	0.60227	0.61628	0	0	0	0	1	1
NP 002373.3				1	0.99338	0.97087	0.70526	0.81928	0.66892	0.60227	0.60227
NP 660087.1					1	0.95745	0.73256	0.24096	0.075342	0.6092	0.61628
NP 660088.1						1	0.70526	0.81928	1	0	0
NP 660089.1							1	0.81013	0.70526	0	0
NP 932061.1								1	0.81928	0	0
XP 011535075.1									1	0	0
XP 016876801.1										1	1
XP 016876802.1											1

Table A66. The parameter Negative Predictive Value (NPV) of the HELIOS method with referencing the ClustalΩ in the accuracy measurement of classification output, assuming the *Jaspar PWMs Resource, JASPAR Predicted Transcription Factor Targets Dataset, MAX Gene Set* [3].

	NP 001257997.1	NP 001257998.1	NP 001307344.1	NP 002373.3	NP 660087.1	NP 660088.1	NP 660089.1	NP 932061.1	XP 011535075.1	XP 016876801.1	XP 016876802.1
NP 001257997.1	1	0.99733	0.99689	0.99812	0.99808	0.99707	0.99988	0.99706	0.99773	0.99689	0.99689
NP 001257998.1		1	0.99831	0.99864	0.99865	0.99788	0.99687	0.99984	0.99858	0.99831	0.99831
NP 001307344.1			1	0.99786	0.99773	0.99707	0.99718	0.9985	0.99465	1	1
NP 002373.3				1	0.99996	0.99982	0.99836	0.99886	0.99823	0.99786	0.99786
NP 660087.1					1	0.99974	0.99861	0.99566	0.99469	0.99773	0.99773
NP 660088.1						1	0.99745	0.99823	1	0.99707	0.99707
NP 660089.1							1	0.99785	0.99801	0.99718	0.99718
NP 932061.1								1	0.99881	0.9985	0.9985
XP 011535075.1									1	0.99465	0.99465
XP 016876801.1										1	1
XP 016876802.1											1

Table A67. The parameter Matthew's Coefficient Correlation (MCC) of the HELIOS method with referencing the ClustalΩ in the accuracy measurement of classification output, assuming the *Jaspar PWMs Resource, JASPAR Predicted Transcription Factor Targets Dataset, MAX Gene Set* [3].

	NP 001257997.1	NP 001257998.1	NP 001307344.1	NP 002373.3	NP 660087.1	NP 660088.1	NP 660089.1	NP 932061.1	XP 011535075.1	XP 016876801.1	XP 016876802.1
NP 001257997.1	1	0.77221	-0.0051207	0.67268	0.68447	0.67157	0.98838	0.70046	0.65674	-0.0051207	-0.0051207
NP 001257998.1		1	-0.0040316	0.78242	0.79595	0.78166	0.72264	0.98632	0.78236	-0.0040316	-0.0040316
NP 001307344.1			1	0.60703	0.61401	-0.0049979	-0.0048317	-0.0036958	-0.0055522	1	1
NP 002373.3				1	0.99334	0.97069	0.71494	0.81814	0.68102	0.60703	0.60703
NP 660087.1					1	0.95719	0.74419	0.25051	0.07335	0.61043	0.61401
NP 660088.1						1	0.71397	0.81751	1	-0.0049979	-0.0049979
NP 660089.1							1	0.79804	0.69963	-0.0048317	-0.0048317
NP 932061.1								1	0.81809	-0.0036958	-0.0036958
XP 011535075.1									1	-0.0055522	-0.0055522
XP 016876801.1										1	1
XP 016876802.1											1

Table A68. The parameter Test's Accuracy (F-Score) of the HELIOS method with referencing the ClustalΩ in the accuracy measurement of classification output, assuming the *Jaspar PWMs Resource, JASPAR Predicted Transcription Factor Targets Dataset, MAX Gene Set* [3].

	NP 001257997.1	NP 001257998.1	NP 001307344.1	NP 002373.3	NP 660087.1	NP 660088.1	NP 660089.1	NP 932061.1	XP 011535075.1	XP 016876801.1	XP 016876802.1
NP 001257997.1	1	0.77465	0	0.67456	0.68639	0.67456	0.98851	0.70345	0.65896	0	0
NP 001257998.1		1	0	0.78378	0.7973	0.78378	0.72464	0.98649	0.78378	0	0
NP 001307344.1			1	0.6092	0.61628	0	0	0	0	1	1
NP 002373.3				1	0.99338	0.97087	0.71658	0.81928	0.68276	0.6092	0.6092
NP 660087.1					1	0.95745	0.74556	0.25478	0.078853	0.61272	0.61628
NP 660088.1						1	0.71658	0.81928	1	0	0
NP 660089.1							1	0.8	0.70157	0	0
NP 932061.1								1	0.81928	0	0
XP 011535075.1									1	0	0
XP 016876801.1										1	1
XP 016876802.1											1

Table A69. The parameter Sensitivity (SEN) of the HELIOS method with referencing the MUSCLE in the accuracy measurement of classification output, assuming the *Jaspar PWMs Resource, JASPAR Predicted Transcription Factor Targets Dataset, MAX Gene Set* [3].

	NP 001257997.1	NP 001257998.1	NP 001307344.1	NP 002373.3	NP 660087.1	NP 660088.1	NP 660089.1	NP 932061.1	XP 011535075.1	XP 016876801.1	XP 016876802.1
NP 001257997.1	1	0.76389	0	0.7907	0.7907	0.7907	1	0.72222	0.7907	0	0
NP 001257998.1		1	0	0.71831	0.71831	0.71831	0.70833	1	0.71831	0	0
NP 001307344.1			1	0.43299	0.43299	0	0	0	0	1	1
NP 002373.3				1	1	0.96117	0.67368	0.75	0.66667	0.43299	0.43299
NP 660087.1					1	0.95745	0.67442	0.16901	0.083333	0.43299	0.43299
NP 660088.1						1	0.81053	0.75	0.96117	0	0
NP 660089.1							1	0.79012	0.81053	0	0
NP 932061.1								1	0.75	0	0
XP 011535075.1									1	0	0
XP 016876801.1										1	1
XP 016876802.1											1

Table A70. The parameter Specification (Spec) of the HELIOS method with referencing the MUSCLE in the accuracy measurement of classification output, assuming the *Jaspar PWMs Resource, JASPAR Predicted Transcription Factor Targets Dataset, MAX Gene Set* [3].

	NP 001257997.1	NP 001257998.1	NP 001307344.1	NP 002373.3	NP 660087.1	NP 660088.1	NP 660089.1	NP 932061.1	XP 011535075.1	XP 016876801.1	XP 016876802.1
NP 001257997.1	1	0.99764	0.99153	0.9987	0.99862	0.99797	1	0.99706	0.99864	0.99153	0.99153
NP 001257998.1		1	0.99031	0.99805	0.99793	0.99695	0.99787	1	0.99796	0.99031	0.99031
NP 001307344.1			1	0.99702	0.99698	0.99144	0.99168	0.99087	0.99424	1	1
NP 002373.3				1	1	0.99976	0.99797	0.99826	0.99811	0.99702	0.99702
NP 660087.1					1	0.99974	0.99806	0.9943	0.99416	0.99691	0.99698
NP 660088.1						1	0.99816	0.99728	0.99974	0.99144	0.99144
NP 660089.1							1	0.9981	0.99877	0.99168	0.99168
NP 932061.1								1	0.99818	0.99087	0.99087
XP 011535075.1									1	0.99424	0.99424
XP 016876801.1										1	1
XP 016876802.1											1

Table A71. The parameter Accuracy (Acc) of the HELIOS method with referencing the MUSCLE in the accuracy measurement of classification output, assuming the *Jaspar PWMs Resource*, *JASPAR Predicted Transcription Factor Targets Dataset*, *MAX Gene Set* [3].

	NP 001257997.1	NP 001257998.1	NP 001307344.1	NP 002373.3	NP 660087.1	NP 660088.1	NP 660089.1	NP 932061.1	XP 011535075.1	XP 016876801.1	XP 016876802.1
NP 001257997.1	1	0.99503	0.98507	0.99741	0.99726	0.99598	1	0.99432	0.9973	0.98507	0.98507
NP 001257998.1		1	0.98272	0.99637	0.99615	0.99436	0.99493	1	0.9962	0.98272	0.98272
NP 001307344.1			1	0.99349	0.99324	0.98559	0.98582	0.9841	0.98821	1	1
NP 002373.3				1	1	0.99951	0.99596	0.99676	0.99604	0.99349	0.99349
NP 660087.1					1	0.99949	0.99614	0.98963	0.98849	0.99317	0.99324
NP 660088.1						1	0.99636	0.99497	0.99949	0.98559	0.98559
NP 660089.1							1	0.99598	0.99755	0.98582	0.98582
NP 932061.1								1	0.99661	0.9841	0.9841
XP 011535075.1									1	0.98821	0.98821
XP 016876801.1										1	1
XP 016876802.1											1

Table A72. The parameter Positive Predictive Value (PPV) of the HELIOS method with referencing the MUSCLE in the accuracy measurement of classification output, assuming the *Jaspar PWMs Resource*, *JASPAR Predicted Transcription Factor Targets Dataset*, *MAX Gene Set* [3].

	NP 001257997.1	NP 001257998.1	NP 001307344.1	NP 002373.3	NP 660087.1	NP 660088.1	NP 660089.1	NP 932061.1	XP 011535075.1	XP 016876801.1	XP 016876802.1
NP 001257997.1	1	0.78571	0	0.7907	0.7907	0.7907	1	0.71233	0.7907	0	0
NP 001257998.1		1	0	0.68919	0.68919	0.68919	0.77273	1	0.68919	0	0
NP 001307344.1			1	0.47727	0.48837	0	0	0	0	1	1
NP 002373.3				1	1	0.96117	0.67368	0.72289	0.68919	0.47727	0.47727
NP 660087.1					1	0.95745	0.67442	0.14458	0.082192	0.48276	0.48837
NP 660088.1						1	0.81053	0.72289	0.96117	0	0
NP 660089.1							1	0.81013	0.81053	0	0
NP 932061.1								1	0.72289	0	0
XP 011535075.1									1	0	0
XP 016876801.1										1	1
XP 016876802.1											1

Table A73. The parameter Negative Predictive Value (NPV) of the HELIOS method with referencing the MUSCLE in the accuracy measurement of classification output, assuming the *Jaspar PWMs Resource, JASPAR Predicted Transcription Factor Targets Dataset, MAX Gene Set* [3].

	NP 001257997.1	NP 001257998.1	NP 001307344.1	NP 002373.3	NP 660087.1	NP 660088.1	NP 660089.1	NP 932061.1	XP 011535075.1	XP 016876801.1	XP 016876802.1
NP 001257997.1	1	0.99733	0.99343	0.9987	0.99862	0.99797	1	0.9972	0.99864	0.99343	0.99343
NP 001257998.1		1	0.99226	0.9983	0.9982	0.99735	0.99702	1	0.99822	0.99226	0.99226
NP 001307344.1			1	0.99644	0.99622	0.99404	0.99404	0.99311	0.9939	1	1
NP 002373.3				1	1	0.99976	0.99797	0.99848	0.9979	0.99644	0.99644
NP 660087.1					1	0.99974	0.99806	0.99526	0.99425	0.99622	0.99622
NP 660088.1						1	0.99816	0.99764	0.99974	0.99404	0.99404
NP 660089.1							1	0.99785	0.99877	0.99404	0.99404
NP 932061.1								1	0.99841	0.99311	0.99311
XP 011535075.1									1	0.9939	0.9939
XP 016876801.1										1	1
XP 016876802.1											1

Table A74. The parameter Matthew's Coefficient Correlation (MCC) of the HELIOS method with referencing the MUSCLE in the accuracy measurement of classification output, assuming the *Jaspar PWMs Resource, JASPAR Predicted Transcription Factor Targets Dataset, MAX Gene Set* [3].

	NP 001257997.1	NP 001257998.1	NP 001307344.1	NP 002373.3	NP 660087.1	NP 660088.1	NP 660089.1	NP 932061.1	XP 011535075.1	XP 016876801.1	XP 016876802.1
NP 001257997.1	1	0.77221	-0.0074606	0.7894	0.78932	0.78867	1	0.71439	0.78934	-0.0074606	-0.0074606
NP 001257998.1		1	-0.0086571	0.70177	0.70166	0.70075	0.73729	1	0.70169	-0.0086571	-0.0086571
NP 001307344.1			1	0.45133	0.45646	-0.0071395	-0.0070384	-0.0079334	-0.0059284	1	1
NP 002373.3				1	1	0.96092	0.67165	0.73469	0.67584	0.45133	0.45133
NP 660087.1					1	0.95719	0.67248	0.15112	0.076968	0.45378	0.45646
NP 660088.1						1	0.80869	0.73378	0.96091	-0.0071395	-0.0071395
NP 660089.1							1	0.79804	0.80929	-0.0070384	-0.0070384
NP 932061.1								1	0.73462	-0.0079334	-0.0079334
XP 011535075.1									1	-0.0059284	-0.0059284
XP 016876801.1										1	1
XP 016876802.1											1

Table A75. The parameter Test's Accuracy (F-Score) of the HELIOS method with referencing the MUSCLE in the accuracy measurement of classification output, assuming the *Jaspar PWMs Resource, JASPAR Predicted Transcription Factor Targets Dataset, MAX Gene Set* [3].

	NP 001257997.1	NP 001257998.1	NP 001307344.1	NP 002373.3	NP 660087.1	NP 660088.1	NP 660089.1	NP 932061.1	XP 011535075.1	XP 016876801.1	XP 016876802.1
NP 001257997.1	1	0.77465	0	0.7907	0.7907	0.7907	1	0.71724	0.7907	0	0
NP 001257998.1		1	0	0.70345	0.70345	0.70345	0.73913	1	0.70345	0	0
NP 001307344.1			1	0.45405	0.45902	0	0	0	0	1	1
NP 002373.3				1	1	0.96117	0.67368	0.7362	0.67774	0.45405	0.45405
NP 660087.1					1	0.95745	0.67442	0.15584	0.082759	0.45652	0.45902
NP 660088.1						1	0.81053	0.7362	0.96117	0	0
NP 660089.1							1	0.8	0.81053	0	0
NP 932061.1								1	0.7362	0	0
XP 011535075.1									1	0	0
XP 016876801.1										1	1
XP 016876802.1											1

Table A76. The parameter Sensitivity (SEN) of the HELIOS method with referencing the T-Coffee in the accuracy measurement of classification output, assuming the *Jaspar PWMs Resource, JASPAR Predicted Transcription Factor Targets Dataset, MAX Gene Set* [3].

	NP 001257997.1	NP 001257998.1	NP 001307344.1	NP 002373.3	NP 660087.1	NP 660088.1	NP 660089.1	NP 932061.1	XP 011535075.1	XP 016876801.1	XP 016876802.1
NP 001257997.1	1	0.76389	0.087719	0.86047	0.86047	0.86047	1	0.72222	0.86047	0.087719	0.087719
NP 001257998.1		1	0.087719	0.80282	0.80282	0.80282	0.70833	1	0.80282	0.087719	0.087719
NP 001307344.1			1	0.10345	0.10345	0.087719	0.087719	0.087719	0.087719	1	1
NP 002373.3				1	1	0.97059	0.73684	0.825	0.70213	0.10345	0.10345
NP 660087.1					1	0.96774	0.74419	0.16901	0.090909	0.10345	0.10345
NP 660088.1						1	0.87368	0.825	0.97087	0.087719	0.087719
NP 660089.1							1	0.79012	0.87368	0.087719	0.087719
NP 932061.1								1	0.825	0.087719	0.087719
XP 011535075.1									1	0.087719	0.087719
XP 016876801.1										1	1
XP 016876802.1											1

Table A77. The parameter Specification (Spec) of the HELIOS method with referencing the T-Coffee in the accuracy measurement of classification output, assuming the *Jaspar PWMs Resource, JASPAR Predicted Transcription Factor Targets Dataset, MAX Gene Set* [3].

	NP 001257997.1	NP 001257998.1	NP 001307344.1	NP 002373.3	NP 660087.1	NP 660088.1	NP 660089.1	NP 932061.1	XP 011535075.1	XP 016876801.1	XP 016876802.1
NP 001257997.1	1	0.99764	0.99213	0.99913	0.99908	0.99865	1	0.99706	0.99909	0.99213	0.99213
NP 001257998.1		1	0.99101	0.99856	0.99847	0.99775	0.99787	1	0.99849	0.99101	0.99101
NP 001307344.1			1	0.9947	0.99452	0.99195	0.99222	0.99149	0.99459	1	1
NP 002373.3				1	1	0.99976	0.99836	0.99871	0.99799	0.9947	0.9947
NP 660087.1					1	0.99974	0.99847	0.9943	0.99417	0.99445	0.99452
NP 660088.1						1	0.99877	0.99799	0.99981	0.99195	0.99195
NP 660089.1							1	0.9981	0.99918	0.99222	0.99222
NP 932061.1								1	0.99865	0.99149	0.99149
XP 011535075.1									1	0.99459	0.99459
XP 016876801.1										1	1
XP 016876802.1											1

Table A78. The parameter Accuracy (Acc) of the HELIOS method with referencing the T-Coffee in the accuracy measurement of classification output, assuming the *Jaspar PWMs Resource, JASPAR Predicted Transcription Factor Targets Dataset, MAX Gene Set* [3].

	NP 001257997.1	NP 001257998.1	NP 001307344.1	NP 002373.3	NP 660087.1	NP 660088.1	NP 660089.1	NP 932061.1	XP 011535075.1	XP 016876801.1	XP 016876802.1
NP 001257997.1	1	0.99503	0.98602	0.99828	0.99817	0.99732	1	0.99432	0.9982	0.98602	0.98602
NP 001257998.1		1	0.98384	0.99738	0.99723	0.99593	0.99493	1	0.99726	0.98384	0.98384
NP 001307344.1			1	0.99137	0.99099	0.98679	0.98668	0.9851	0.99111	1	1
NP 002373.3				1	1	0.99958	0.99674	0.99767	0.99628	0.99137	0.99137
NP 660087.1					1	0.99955	0.99696	0.98963	0.98901	0.99092	0.99099
NP 660088.1						1	0.99757	0.99637	0.99962	0.98679	0.98679
NP 660089.1							1	0.99598	0.99837	0.98668	0.98668
NP 932061.1								1	0.99756	0.9851	0.9851
XP 011535075.1									1	0.99111	0.99111
XP 016876801.1										1	1
XP 016876802.1											1

Table A79. The parameter Positive Predictive Value (PPV) of the HELIOS method with referencing the T-Coffee in the accuracy measurement of classification output, assuming the *Jaspar PWMs Resource, JASPAR Predicted Transcription Factor Targets Dataset, MAX Gene Set* [3].

	NP 001257997.1	NP 001257998.1	NP 001307344.1	NP 002373.3	NP 660087.1	NP 660088.1	NP 660089.1	NP 932061.1	XP 011535075.1	XP 016876801.1	XP 016876802.1
NP 001257997.1	1	0.78571	0.070423	0.86047	0.86047	0.86047	1	0.71233	0.86047	0.070423	0.070423
NP 001257998.1		1	0.072464	0.77027	0.77027	0.77027	0.77273	1	0.77027	0.072464	0.072464
NP 001307344.1			1	0.068182	0.069767	0.058824	0.064935	0.068493	0.058824	1	1
NP 002373.3				1	1	0.96117	0.73684	0.79518	0.66892	0.068182	0.068182
NP 660087.1					1	0.95745	0.74419	0.14458	0.082192	0.068966	0.069767
NP 660088.1						1	0.87368	0.79518	0.97087	0.058824	0.058824
NP 660089.1							1	0.81013	0.87368	0.064935	0.064935
NP 932061.1								1	0.79518	0.068493	0.068493
XP 011535075.1									1	0.058824	0.058824
XP 016876801.1										1	1
XP 016876802.1											1

Table A80. The parameter Negative Predictive Value (NPV) of the HELIOS method with referencing the T-Coffee in the accuracy measurement of classification output, assuming the *Jaspar PWMs Resource, JASPAR Predicted Transcription Factor Targets Dataset, MAX Gene Set* [3].

	NP 001257997.1	NP 001257998.1	NP 001307344.1	NP 002373.3	NP 660087.1	NP 660088.1	NP 660089.1	NP 932061.1	XP 011535075.1	XP 016876801.1	XP 016876802.1
NP 001257997.1	1	0.99733	0.99379	0.99913	0.99908	0.99865	1	0.9972	0.99909	0.99379	0.99379
NP 001257998.1		1	0.99269	0.99881	0.99874	0.99815	0.99702	1	0.99876	0.99269	0.99269
NP 001307344.1			1	0.99663	0.99643	0.99475	0.99437	0.99348	0.99648	1	1
NP 002373.3				1	1	0.99982	0.99836	0.99894	0.99827	0.99663	0.99663
NP 660087.1					1	0.99981	0.99847	0.99526	0.99477	0.99643	0.99643
NP 660088.1						1	0.99877	0.99835	0.99981	0.99475	0.99475
NP 660089.1							1	0.99785	0.99918	0.99437	0.99437
NP 932061.1								1	0.99889	0.99348	0.99348
XP 011535075.1									1	0.99648	0.99648
XP 016876801.1										1	1
XP 016876802.1											1

Table A81. The parameter Matthew's Coefficient Correlation (MCC) of the HELIOS method with referencing the T-Coffee in the accuracy measurement of classification output, assuming the *Jaspar PWMs Resource, JASPAR Predicted Transcription Factor Targets Dataset, MAX Gene Set* [3].

	NP 001257997.1	NP 001257998.1	NP 001307344.1	NP 002373.3	NP 660087.1	NP 660088.1	NP 660089.1	NP 932061.1	XP 011535075.1	XP 016876801.1	XP 016876802.1
NP 001257997.1	1	0.77221	0.071601	0.8596	0.85955	0.85911	1	0.71439	0.85956	0.071601	0.071601
NP 001257998.1		1	0.071619	0.78506	0.78498	0.78433	0.73729	1	0.785	0.071619	0.071619
NP 001307344.1			1	0.079756	0.080529	0.06533	0.068853	0.070066	0.067466	1	1
NP 002373.3				1	1	0.96565	0.7352	0.80878	0.68346	0.079756	0.079756
NP 660087.1					1	0.96235	0.74266	0.15112	0.080919	0.080011	0.080529
NP 660088.1						1	0.87246	0.80813	0.97068	0.06533	0.06533
NP 660089.1							1	0.79804	0.87286	0.068853	0.068853
NP 932061.1								1	0.80873	0.070066	0.070066
XP 011535075.1									1	0.067466	0.067466
XP 016876801.1										1	1
XP 016876802.1											1

Table A82. The parameter Test's Accuracy (F-Score) of the HELIOS method with referencing the T-Coffee in the accuracy measurement of classification output, assuming the *Jaspar PWMs Resource, JASPAR Predicted Transcription Factor Targets Dataset, MAX Gene Set* [3].

	NP 001257997.1	NP 001257998.1	NP 001307344.1	NP 002373.3	NP 660087.1	NP 660088.1	NP 660089.1	NP 932061.1	XP 011535075.1	XP 016876801.1	XP 016876802.1
NP 001257997.1	1	0.77465	0.078125	0.86047	0.86047	0.86047	1	0.71724	0.86047	0.078125	0.078125
NP 001257998.1		1	0.079365	0.78621	0.78621	0.78621	0.73913	1	0.78621	0.079365	0.079365
NP 001307344.1			1	0.082192	0.083333	0.070423	0.074627	0.076923	0.070423	1	1
NP 002373.3				1	1	0.96585	0.73684	0.80982	0.68512	0.082192	0.082192
NP 660087.1					1	0.96257	0.74419	0.15584	0.086331	0.082759	0.083333
NP 660088.1						1	0.87368	0.80982	0.97087	0.070423	0.070423
NP 660089.1							1	0.8	0.87368	0.074627	0.074627
NP 932061.1								1	0.80982	0.076923	0.076923
XP 011535075.1									1	0.070423	0.070423
XP 016876801.1										1	1
XP 016876802.1											1

Table A83. The parameter Sensitivity (SEN) of the HELIOS method with referencing the Kalign in the accuracy measurement of classification output, assuming the *Jaspar PWMs Resource, JASPAR Predicted Transcription Factor Targets Dataset, MAX Gene Set* [3].

	NP 001257997.1	NP 001257998.1	NP 001307344.1	NP 002373.3	NP 660087.1	NP 660088.1	NP 660089.1	NP 932061.1	XP 011535075.1	XP 016876801.1	XP 016876802.1
NP 001257997.1	1	0.7027	0	0.82558	0.82558	0.82558	1	0.7027	0.82558	0	0
NP 001257998.1		1	0	0.73973	0.73973	0.73973	0.64865	1	0.73973	0	0
NP 001307344.1			1	0.58333	0.58333	0	0	0	0	1	1
NP 002373.3				1	1	0.97087	0.70526	0.76829	0.64706	0.58333	0.58333
NP 660087.1					1	0.96809	0.7093	0.16438	0.083333	0.58333	0.58333
NP 660088.1						1	0.84211	0.76829	1	0	0
NP 660089.1							1	0.73494	0.84211	0	0
NP 932061.1								1	0.76829	0	0
XP 011535075.1									1	0	0
XP 016876801.1										1	1
XP 016876802.1											1

Table A84. The parameter Specification (Spec) of the HELIOS method with referencing the Kalign in the accuracy measurement of classification output, assuming the *Jaspar PWMs Resource, JASPAR Predicted Transcription Factor Targets Dataset, MAX Gene Set* [3].

	NP 001257997.1	NP 001257998.1	NP 001307344.1	NP 002373.3	NP 660087.1	NP 660088.1	NP 660089.1	NP 932061.1	XP 011535075.1	XP 016876801.1	XP 016876802.1
NP 001257997.1	1	0.99717	0.99155	0.99892	0.99885	0.99831	1	0.99706	0.99887	0.99155	0.99155
NP 001257998.1		1	0.99035	0.9983	0.9982	0.99735	0.99744	1	0.99822	0.99035	0.99035
NP 001307344.1			1	0.99793	0.99794	0.99146	0.9917	0.9909	0.99424	1	1
NP 002373.3				1	1	0.99982	0.99817	0.99848	0.99799	0.99793	0.99793
NP 660087.1					1	0.99981	0.99827	0.9943	0.99416	0.99787	0.99794
NP 660088.1						1	0.99847	0.99764	1	0.99146	0.99146
NP 660089.1							1	0.99772	0.99897	0.9917	0.9917
NP 932061.1								1	0.99841	0.9909	0.9909
XP 011535075.1									1	0.99424	0.99424
XP 016876801.1										1	1
XP 016876802.1											1

Table A85. The parameter Accuracy (Acc) of the HELIOS method with referencing the Kalign in the accuracy measurement of classification output, assuming the *Jaspar PWMs Resource, JASPAR Predicted Transcription Factor Targets Dataset*, *MAX Gene Set* [3].

	NP 001257997.1	NP 001257998.1	NP 001307344.1	NP 002373.3	NP 660087.1	NP 660088.1	NP 660089.1	NP 932061.1	XP 011535075.1	XP 016876801.1	XP 016876802.1
NP 001257997.1	1	0.99379	0.98697	0.99784	0.99772	0.99665	1	0.99405	0.99775	0.98697	0.98697
NP 001257998.1		1	0.98635	0.99671	0.99651	0.99488	0.99381	1	0.99656	0.98635	0.98635
NP 001307344.1			1	0.99536	0.99522	0.98749	0.98754	0.98733	0.98828	1	1
NP 002373.3				1	1	0.99964	0.99635	0.99706	0.99579	0.99536	0.99536
NP 660087.1					1	0.99961	0.99655	0.98947	0.98849	0.99515	0.99522
NP 660088.1						1	0.99697	0.99544	1	0.98749	0.98749
NP 660089.1							1	0.99498	0.99796	0.98754	0.98754
NP 932061.1								1	0.99693	0.98733	0.98733
XP 011535075.1									1	0.98828	0.98828
XP 016876801.1										1	1
XP 016876802.1											1

Table A86. The parameter Positive Predictive Value (PPV) of the HELIOS method with referencing the Kalign in the accuracy measurement of classification output, assuming the *Jaspar PWMs Resource, JASPAR Predicted Transcription Factor Targets Dataset*, *MAX Gene Set* [3].

	NP 001257997.1	NP 001257998.1	NP 001307344.1	NP 002373.3	NP 660087.1	NP 660088.1	NP 660089.1	NP 932061.1	XP 011535075.1	XP 016876801.1	XP 016876802.1
NP 001257997.1	1	0.74286	0	0.82558	0.82558	0.82558	1	0.71233	0.82558	0	0
NP 001257998.1		1	0	0.72973	0.72973	0.72973	0.72727	1	0.72973	0	0
NP 001307344.1			1	0.63636	0.65116	0	0	0	0	1	1
NP 002373.3				1	1	0.97087	0.70526	0.75904	0.66892	0.63636	0.63636
NP 660087.1					1	0.96809	0.7093	0.14458	0.082192	0.64368	0.65116
NP 660088.1						1	0.84211	0.75904	1	0	0
NP 660089.1							1	0.77215	0.84211	0	0
NP 932061.1								1	0.75904	0	0
XP 011535075.1									1	0	0
XP 016876801.1										1	1
XP 016876802.1											1

Table A87. The parameter Negative Predictive Value (NPV) of the HELIOS method with referencing the Kalign in the accuracy measurement of classification output, assuming the *Jaspar PWMs Resource, JASPAR Predicted Transcription Factor Targets Dataset, MAX Gene Set* [3].

	NP 001257997.1	NP 001257998.1	NP 001307344.1	NP 002373.3	NP 660087.1	NP 660088.1	NP 660089.1	NP 932061.1	XP 011535075.1	XP 016876801.1	XP 016876802.1
NP 001257997.1	1	0.99655	0.99534	0.99892	0.99885	0.99831	1	0.99692	0.99887	0.99534	0.99534
NP 001257998.1		1	0.99592	0.99839	0.99829	0.99748	0.99631	1	0.99831	0.99592	0.99592
NP 001307344.1			1	0.99741	0.99725	0.99596	0.99578	0.99637	0.99397	1	1
NP 002373.3				1	1	0.99982	0.99817	0.99856	0.99778	0.99741	0.99741
NP 660087.1					1	0.99981	0.99827	0.9951	0.99425	0.99725	0.99725
NP 660088.1						1	0.99847	0.99776	1	0.99596	0.99596
NP 660089.1							1	0.99721	0.99897	0.99578	0.99578
NP 932061.1								1	0.99849	0.99637	0.99637
XP 011535075.1									1	0.99397	0.99397
XP 016876801.1										1	1
XP 016876802.1											1

Table A88. The parameter Matthew's Coefficient Correlation (MCC) of the HELIOS method with referencing the Kalign in the accuracy measurement of classification output, assuming the *Jaspar PWMs Resource, JASPAR Predicted Transcription Factor Targets Dataset, MAX Gene Set* [3].

	NP 001257997.1	NP 001257998.1	NP 001307344.1	NP 002373.3	NP 660087.1	NP 660088.1	NP 660089.1	NP 932061.1	XP 011535075.1	XP 016876801.1	XP 016876802.1
NP 001257997.1	1	0.71937	-0.0062764	0.8245	0.82443	0.82389	1	0.70449	0.82445	-0.0062764	-0.0062764
NP 001257998.1		1	-0.0062748	0.73305	0.73295	0.73213	0.68374	1	0.73298	-0.0062748	-0.0062748
NP 001307344.1			1	0.60695	0.61393	-0.005873	-0.0059217	-0.0057514	-0.0058951	1	1
NP 002373.3				1	1	0.97069	0.70343	0.76217	0.65578	0.60695	0.60695
NP 660087.1					1	0.96789	0.70757	0.14888	0.076968	0.61034	0.61393
NP 660088.1						1	0.84057	0.76135	1	-0.005873	-0.005873
NP 660089.1							1	0.75079	0.84108	-0.0059217	-0.0059217
NP 932061.1								1	0.76211	-0.0057514	-0.0057514
XP 011535075.1									1	-0.0058951	-0.0058951
XP 016876801.1										1	1
XP 016876802.1											1

Table A89. The parameter Test's Accuracy (F-Score) of the HELIOS method with referencing the Kalign in the accuracy measurement of classification output, assuming the *Jaspar PWMs Resource, JASPAR Predicted Transcription Factor Targets Dataset, MAX Gene Set* [3].

	NP 001257997.1	NP 001257998.1	NP 001307344.1	NP 002373.3	NP 660087.1	NP 660088.1	NP 660089.1	NP 932061.1	XP 011535075.1	XP 016876801.1	XP 016876802.1
NP 001257997.1	1	0.72222	0	0.82558	0.82558	0.82558	1	0.70748	0.82558	0	0
NP 001257998.1		1	0	0.73469	0.73469	0.73469	0.68571	1	0.73469	0	0
NP 001307344.1			1	0.6087	0.61538	0	0	0	0	1	1
NP 002373.3				1	1	0.97087	0.70526	0.76364	0.65781	0.6087	0.6087
NP 660087.1					1	0.96809	0.7093	0.15385	0.082759	0.61202	0.61538
NP 660088.1						1	0.84211	0.76364	1	0	0
NP 660089.1							1	0.75309	0.84211	0	0
NP 932061.1								1	0.76364	0	0
XP 011535075.1									1	0	0
XP 016876801.1										1	1
XP 016876802.1											1

Table A90. The parameter Sensitivity (SEN) of the HELIOS method with referencing the MAFFT in the accuracy measurement of classification output, assuming the *Jaspar PWMs Resource, JASPAR Predicted Transcription Factor Targets Dataset, MAX Gene Set* [3].

	NP 001257997.1	NP 001257998.1	NP 001307344.1	NP 002373.3	NP 660087.1	NP 660088.1	NP 660089.1	NP 932061.1	XP 011535075.1	XP 016876801.1	XP 016876802.1
NP 001257997.1	1	0.84286	0.06	0.81176	0.81176	0.81176	1	0.8	0.81176	0.06	0.06
NP 001257998.1		1	0.06	0.83099	0.83099	0.83099	0.72857	1	0.83099	0.06	0.06
NP 001307344.1			1	0.47917	0.50538	0.068966	0.056604	0.056604	0.035714	1	1
NP 002373.3				1	1	0.95146	0.69149	0.85	0.71127	0.47917	0.47917
NP 660087.1					1	0.94681	0.69412	0.16901	0.090226	0.49462	0.49462
NP 660088.1						1	0.82979	0.85	0.95146	0.051724	0.051724
NP 660089.1							1	0.83544	0.82979	0.056604	0.056604
NP 932061.1								1	0.85	0.056604	0.056604
XP 011535075.1									1	0.035714	0.035714
XP 016876801.1										1	1
XP 016876802.1											1

Table A91. The parameter Specification (Spec) of the HELIOS method with referencing the MAFFT in the accuracy measurement of classification output, assuming the *Jaspar PWMs Resource, JASPAR Predicted Transcription Factor Targets Dataset, MAX Gene Set* [3].

	NP 001257997.1	NP 001257998.1	NP 001307344.1	NP 002373.3	NP 660087.1	NP 660088.1	NP 660089.1	NP 932061.1	XP 011535075.1	XP 016876801.1	XP 016876802.1
NP 001257997.1	1	0.99827	0.99189	0.99877	0.9987	0.99808	1	0.99762	0.99871	0.99189	0.99189
NP 001257998.1		1	0.99074	0.99873	0.99865	0.99801	0.99787	1	0.99867	0.99074	0.99074
NP 001307344.1			1	0.99728	0.99732	0.99185	0.99201	0.99125	0.99444	1	1
NP 002373.3				1	1	0.99969	0.99803	0.99886	0.99807	0.99728	0.99728
NP 660087.1					1	0.99968	0.99813	0.9943	0.99417	0.99718	0.99725
NP 660088.1						1	0.99826	0.99823	0.99968	0.99174	0.99174
NP 660089.1							1	0.99835	0.99884	0.99201	0.99201
NP 932061.1								1	0.99881	0.99125	0.99125
XP 011535075.1									1	0.99444	0.99444
XP 016876801.1										1	1
XP 016876802.1											1

Table A92. The parameter Accuracy (Acc) of the HELIOS method with referencing the MAFFT in the accuracy measurement of classification output, assuming the *Jaspar PWMs Resource, JASPAR Predicted Transcription Factor Targets Dataset, MAX Gene Set* [3].

	NP 001257997.1	NP 001257998.1	NP 001307344.1	NP 002373.3	NP 660087.1	NP 660088.1	NP 660089.1	NP 932061.1	XP 011535075.1	XP 016876801.1	XP 016876802.1
NP 001257997.1	1	0.99658	0.98637	0.99763	0.99749	0.99632	1	0.99571	0.99752	0.98637	0.98637
NP 001257998.1		1	0.98426	0.99772	0.99758	0.99646	0.99521	1	0.99762	0.98426	0.98426
NP 001307344.1			1	0.99407	0.9942	0.98649	0.98668	0.9851	0.98902	1	1
NP 002373.3				1	1	0.99939	0.99616	0.99797	0.99641	0.99407	0.99407
NP 660087.1					1	0.99936	0.99634	0.98963	0.98896	0.99399	0.99406
NP 660088.1						1	0.99666	0.99684	0.99937	0.98629	0.98629
NP 660089.1							1	0.99674	0.99775	0.98668	0.98668
NP 932061.1								1	0.99787	0.9851	0.9851
XP 011535075.1									1	0.98902	0.98902
XP 016876801.1										1	1
XP 016876802.1											1

Table A93. The parameter Positive Predictive Value (PPV) of the HELIOS method with referencing the MAFFT in the accuracy measurement of classification output, assuming the *Jaspar PWMs Resource, JASPAR Predicted Transcription Factor Targets Dataset, MAX Gene Set* [3].

	NP 001257997.1	NP 001257998.1	NP 001307344.1	NP 002373.3	NP 660087.1	NP 660088.1	NP 660089.1	NP 932061.1	XP 011535075.1	XP 016876801.1	XP 016876802.1
NP 001257997.1	1	0.84286	0.042254	0.80233	0.80233	0.80233	1	0.76712	0.80233	0.042254	0.042254
NP 001257998.1		1	0.043478	0.7973	0.7973	0.7973	0.77273	1	0.7973	0.043478	0.043478
NP 001307344.1			1	0.52273	0.54651	0.047059	0.038961	0.041096	0.035294	1	1
NP 002373.3				1	1	0.95146	0.68421	0.81928	0.68243	0.52273	0.52273
NP 660087.1					1	0.94681	0.68605	0.14458	0.082192	0.52874	0.53488
NP 660088.1						1	0.82105	0.81928	0.95146	0.035294	0.035294
NP 660089.1							1	0.83544	0.82105	0.038961	0.038961
NP 932061.1								1	0.81928	0.041096	0.041096
XP 011535075.1									1	0.035294	0.035294
XP 016876801.1										1	1
XP 016876802.1											1

Table A94. The parameter Negative Predictive Value (NPV) of the HELIOS method with referencing the MAFFT in the accuracy measurement of classification output, assuming the *Jaspar PWMs Resource, JASPAR Predicted Transcription Factor Targets Dataset, MAX Gene Set* [3].

	NP 001257997.1	NP 001257998.1	NP 001307344.1	NP 002373.3	NP 660087.1	NP 660088.1	NP 660089.1	NP 932061.1	XP 011535075.1	XP 016876801.1	XP 016876802.1
NP 001257997.1	1	0.99827	0.99438	0.99884	0.99877	0.9982	1	0.99804	0.99879	0.99438	0.99438
NP 001257998.1		1	0.99339	0.99898	0.99892	0.99841	0.9973	1	0.99893	0.99339	0.99339
NP 001307344.1			1	0.99676	0.99684	0.99455	0.99459	0.99373	0.99451	1	1
NP 002373.3				1	1	0.99969	0.9981	0.99909	0.99831	0.99676	0.99676
NP 660087.1					1	0.99968	0.9982	0.99526	0.99473	0.99677	0.99677
NP 660088.1						1	0.99837	0.99858	0.99968	0.99445	0.99445
NP 660089.1							1	0.99835	0.9989	0.99459	0.99459
NP 932061.1								1	0.99905	0.99373	0.99373
XP 011535075.1									1	0.99451	0.99451
XP 016876801.1										1	1
XP 016876802.1											1

Table A95. The parameter Matthew's Coefficient Correlation (MCC) of the HELIOS method with referencing the MAFFT in the accuracy measurement of classification output, assuming the *Jaspar PWMs Resource, JASPAR Predicted Transcription Factor Targets Dataset*, *MAX Gene Set* [3].

	NP 001257997.1	NP 001257998.1	NP 001307344.1	NP 002373.3	NP 660087.1	NP 660088.1	NP 660089.1	NP 932061.1	XP 011535075.1	XP 016876801.1	XP 016876802.1
NP 001257997.1	1	0.84113	0.043603	0.80584	0.80577	0.80517	1	0.78123	0.80578	0.043603	0.043603
NP 001257998.1		1	0.043251	0.81282	0.81275	0.81218	0.74792	1	0.81277	0.043251	0.043251
NP 001307344.1			1	0.4975	0.52263	0.050301	0.040383	0.040824	0.029981	1	1
NP 002373.3				1	1	0.95115	0.68591	0.83348	0.69489	0.4975	0.4975
NP 660087.1					1	0.94649	0.68823	0.15112	0.08057	0.50838	0.51138
NP 660088.1						1	0.82372	0.83291	0.95114	0.035956	0.035956
NP 660089.1							1	0.8338	0.82428	0.040383	0.040383
NP 932061.1								1	0.83343	0.040824	0.040824
XP 011535075.1									1	0.029981	0.029981
XP 016876801.1										1	1
XP 016876802.1											1

Table A96. The parameter Test's Accuracy (F-Score) of the HELIOS method with referencing the MAFFT in the accuracy measurement of classification output, assuming the *Jaspar PWMs Resource, JASPAR Predicted Transcription Factor Targets Dataset*, *MAX Gene Set* [3].

	NP 001257997.1	NP 001257998.1	NP 001307344.1	NP 002373.3	NP 660087.1	NP 660088.1	NP 660089.1	NP 932061.1	XP 011535075.1	XP 016876801.1	XP 016876802.1
NP 001257997.1	1	0.84286	0.049587	0.80702	0.80702	0.80702	1	0.78322	0.80702	0.049587	0.049587
NP 001257998.1		1	0.05042	0.81379	0.81379	0.81379	0.75	1	0.81379	0.05042	0.05042
NP 001307344.1			1	0.5	0.52514	0.055944	0.046154	0.047619	0.035503	1	1
NP 002373.3				1	1	0.95146	0.68783	0.83436	0.69655	0.5	0.5
NP 660087.1					1	0.94681	0.69006	0.15584	0.086022	0.51111	0.51397
NP 660088.1						1	0.8254	0.83436	0.95146	0.041958	0.041958
NP 660089.1							1	0.83544	0.8254	0.046154	0.046154
NP 932061.1								1	0.83436	0.047619	0.047619
XP 011535075.1									1	0.035503	0.035503
XP 016876801.1										1	1
XP 016876802.1											1
